# Supplementary material for: Comparative transcriptomic analysis reveals the cold acclimation during chilling stress in sensitive and resistant passion fruit (Passiflora edulis) cultivars
Source: PeerJ. 2021 Mar 3;9:e10977. doi: 10.7717/peerj.10977 (PMC7936571; doi:10.7717/peerj.10977)
Supplement: Supplemental Information 1 [file peerj-09-10977-s001.docx]

**Supplementary table 1 Details of primers used for RT-qPCR in this study**

| Gene ID | Gene annotation | Database | Forward sequence (5-3’) | Reverse sequence (5-3’) |
| --- | --- | --- | --- | --- |
| *TRINITY_DN8803_c0_g1_i1* | auxin-responsive protein | KEGG | AGCAGAACAGATACGAGGAACT | GAGGCATGTAAGAGGATCAGGA |
| *TRINITY_DN39089_c1_g3_i1* | palmitoyl-protein thioesterase | KEGG | ACAAATGAGCCGTCTGGGTAG | CGCTTCAGCAGCCTAGAGAAT |
| *TRINITY_DN19358_c0_g2_i1* | protein phosphorylation | GO | TTTGGGCAGAGTCCGAACAG | CACCAGGTCCTTGGCACTT |
| *TRINITY_DN25050_c0_g1_i1* | calcium-binding protein | KEGG | GTCCACCATCCAGTCTCACAT | CGAACACCTTGAATGCCTCTG |
| *TRINITY_DN26890_c0_g1_i3* | protein phosphorylation | GO | TAGCCGAATTAGGAGCACCAG | CGAGGAGAAGCCACAACCAT |
| *TRINITY_DN38693_c3_g9_i1* | serine/threonine-protein phosphatase 5 | KEGG | CCGTTCTGGGCTAGGGTTT | CCGCTTAATTGCTCGTGGTT |
| *TRINITY_DN35488_c0_g2_i1* | starch and sucrose metabolism | KEGG | GAGAGCAGAGCGTACCACAT | GACGGCGGAGTACACCATTA |
| *TRINITY_DN30661_c0_g1_i2* | fatty acid metabolic process | GO | GCGTAGGTCAATGTCAGTAGCA | GCATCAAGAGGCAGAGGAAGG |
| *TRINITY_DN36596_c0_g1_i2* | photosynthesis | KEGG | GCTTCCTACTCCTTCCGATTGT | TGCCTTGAGACCAGTGAATGAA |
| *TRINITY_DN38940_c0_g1_i1* | plant hormone signal transduction | KEGG | CTTACTGCCACTGCCATTGC | TACTCATCCTGCGTGAAGAACA |
| *TRINITY_DN24790_c0_g2_i2* | ABA responsive element binding factor | KEGG | TGTCTCCTCCGCACTCCATAT | CAGCCGTCTGTCTACTCATTGA |
| *TRINITY_DN30916_c0_g2_i3* | MAPK signaling pathway | KEGG | GCGGCTTTGTAGGCTGTGA | CCATCTGCGACGGTATCTTGA |
| *TRINITY_DN38458_c1_g4_i1* | trehalose biosynthetic proces | GO | TGCTTGGCGTTGATGATATGGA | TCTCAGCCTGGACTTCCTCAA |
| *TRINITY_DN21214_c0_g2_i2* | plant hormone signal transduction | KEGG | ACACTTGAACTGGTACAGGAGA | CACAGATAGAGCAGCAGAGACT |
| *TRINITY_DN26855_c1_g3_i1* | auxin-responsive protein | KEGG | TCTTCTCTTGAACACGCTCTGA | GATGCCATTGATGTGTCCTCTG |
| *HIS* | histone H3.3 |  | AGAGCCATGCAGTGTTGGCA | CTTGGCGTGGATGGCACAGA |
