# Supplementary material for: Comparative transcriptomic analysis reveals the cold acclimation during chilling stress in sensitive and resistant passion fruit (Passiflora edulis) cultivars
Source: PeerJ. 2021 Mar 3;9:e10977. doi: 10.7717/peerj.10977 (PMC7936571; doi:10.7717/peerj.10977)
Supplement: Supplemental Information 4 [file peerj-09-10977-s004.docx]

**Supplementary table 4 GO enrichment analysis in A vs B**

**1-BP**

| GO ID | Term | Group A | | | | |  | Group B | | | |
| --- | --- | --- | --- | --- | --- | --- | --- | --- | --- | --- | --- |
|  |  | Annotated nuigene number | Significant unigene number | Expected | Classic Fisher | FDR |  | Significant unigene number | Expected | Classic Fisher | FDR |
| GO:0006468 | protein phosphorylation | 1052 | 92 | 77.46 | 0.04196 | 0.449759556 |  | 153 | 107.62 | 1.90E-06 | 0.000535556 |
| GO:0016310 | phosphorylation | 1154 | 97 | 84.97 | 0.08486 | 0.794223689 |  | 158 | 118.06 | 4.10E-05 | 0.002195778 |
| GO:0050896 | response to stimulus | 761 | 67 | 56.03 | 0.06773 | 0.646452673 |  | 102 | 77.85 | 0.00217 | 0.06748 |
| GO:0006629 | lipid metabolic process | 343 | 33 | 25.26 | 0.06765 | 0.646452673 |  | 52 | 35.09 | 0.00232 | 0.06989 |
| GO:0042221 | response to chemical | 151 | 16 | 11.12 | 0.08913 | 0.826166538 |  | 29 | 15.45 | 0.0006 | 0.025147826 |
| GO:0006855 | drug transmembrane transport | 56 | 5 | 4.12 | 0.39611 | 1 |  | 14 | 5.73 | 0.0012 | 0.039889655 |
| GO:0015893 | drug transport | 56 | 5 | 4.12 | 0.39611 | 1 |  | 14 | 5.73 | 0.0012 | 0.039889655 |
| GO:0042493 | response to drug | 56 | 5 | 4.12 | 0.39611 | 1 |  | 14 | 5.73 | 0.0012 | 0.039889655 |
| GO:0008037 | cell recognition | 45 | 9 | 3.31 | 0.00485 | 0.111319048 |  | 16 | 4.6 | 4.70E-06 | 0.000535556 |
| GO:0044706 | multi-multicellular organism process | 45 | 9 | 3.31 | 0.00485 | 0.111319048 |  | 16 | 4.6 | 4.70E-06 | 0.000535556 |
| GO:0009875 | pollen-pistil interaction | 45 | 9 | 3.31 | 0.00485 | 0.111319048 |  | 16 | 4.6 | 4.70E-06 | 0.000535556 |
| GO:0009856 | pollination | 45 | 9 | 3.31 | 0.00485 | 0.111319048 |  | 16 | 4.6 | 4.70E-06 | 0.000535556 |
| GO:0048544 | recognition of pollen | 45 | 9 | 3.31 | 0.00485 | 0.111319048 |  | 16 | 4.6 | 4.70E-06 | 0.000535556 |
| GO:0051704 | multi-organism process | 61 | 12 | 4.49 | 0.00141 | 0.050342222 |  | 19 | 6.24 | 5.90E-06 | 0.00056876 |
| GO:0022414 | reproductive process | 51 | 10 | 3.76 | 0.00354 | 0.095061111 |  | 17 | 5.22 | 6.50E-06 | 0.000569636 |
| GO:0000003 | reproduction | 52 | 10 | 3.83 | 0.0041 | 0.106821622 |  | 17 | 5.32 | 8.80E-06 | 0.000605943 |
| GO:0032501 | multicellular organismal process | 67 | 10 | 4.93 | 0.02389 | 0.319860556 |  | 17 | 6.85 | 0.00031 | 0.013583636 |
| GO:0030244 | cellulose biosynthetic process | 50 | 9 | 3.68 | 0.00986 | 0.16388 |  | 13 | 5.12 | 0.0012 | 0.039889655 |
| GO:0030243 | cellulose metabolic process | 50 | 9 | 3.68 | 0.00986 | 0.16388 |  | 13 | 5.12 | 0.0012 | 0.039889655 |

**2-CC**

| GO ID | Term | Group A | | | | |  | Group B | | | |
| --- | --- | --- | --- | --- | --- | --- | --- | --- | --- | --- | --- |
|  |  | Annotated nuigene number | Significant unigene number | Expected | Classic Fisher | FDR |  | Significant unigene number | Expected | Classic Fisher | FDR |
| GO:0016020 | membrane | 2437 | 140 | 148.2 | 0.884 | 1 |  | 213 | 185.41 | 0.00032 | 0.0768 |
| GO:0031224 | intrinsic component of membrane | 1070 | 70 | 65.07 | 0.2516 | 1 |  | 99 | 81.41 | 0.0114 | 0.5488 |
| GO:0016021 | integral component of membrane | 1052 | 69 | 63.98 | 0.2459 | 1 |  | 97 | 80.04 | 0.01372 | 0.5488 |
| GO:0009522 | photosystem I | 17 | 4 | 1.03 | 0.0169 | 0.554666667 |  | 5 | 1.29 | 0.00711 | 0.5488 |
| GO:0009538 | photosystem I reaction center | 13 | 4 | 0.79 | 0.0062 | 0.248 |  | 4 | 0.99 | 0.01354 | 0.5488 |

**3-MF**

| GO ID | Term | Group A | | | | |  | Group B | | | |
| --- | --- | --- | --- | --- | --- | --- | --- | --- | --- | --- | --- |
|  |  | Annotated nuigene number | Significant unigene number | Expected | Classic Fisher | FDR |  | Significant unigene number | Expected | Classic Fisher | FDR |
| GO.ID | Term | Annotated | Significant | Expected | classicFisher | fdr_MF |  | Significant | Expected | classicFisher | fdr_MF |
| GO:0140096 | catalytic activity, acting on a protein | 1737 | 123 | 130.63 | 0.78366 | 1 |  | 200 | 179.06 | 0.04419 | 0.420454853 |
| GO:0016772 | transferase activity, transferring phosp... | 1491 | 103 | 112.13 | 0.84102 | 1 |  | 173 | 153.7 | 0.04704 | 0.441085217 |
| GO:0016301 | kinase activity | 1216 | 95 | 91.45 | 0.35999 | 1 |  | 162 | 125.35 | 0.00028 | 0.0072464 |
| GO:0016773 | phosphotransferase activity, alcohol gro... | 1256 | 98 | 94.46 | 0.3623 | 1 |  | 165 | 129.48 | 0.00048 | 0.010708966 |
| GO:0004672 | protein kinase activity | 1103 | 92 | 82.95 | 0.15492 | 0.922420183 |  | 154 | 113.7 | 4.00E-05 | 0.0016175 |
| GO:0043169 | cation binding | 1409 | 146 | 105.96 | 2.80E-05 | 0.000696769 |  | 202 | 145.25 | 3.30E-07 | 3.56E-05 |
| GO:0046872 | metal ion binding | 1393 | 146 | 104.76 | 1.50E-05 | 0.000421957 |  | 201 | 143.6 | 2.20E-07 | 2.85E-05 |
| GO:0140110 | transcription regulator activity | 418 | 26 | 31.44 | 0.86969 | 1 |  | 67 | 43.09 | 0.00016 | 0.005176 |
| GO:0003700 | DNA binding transcription factor activit... | 395 | 24 | 29.71 | 0.88769 | 1 |  | 64 | 40.72 | 0.00016 | 0.005176 |
| GO:0016491 | oxidoreductase activity | 1582 | 167 | 118.97 | 2.20E-06 | 8.90E-05 |  | 198 | 163.08 | 0.00159 | 0.028575833 |
| GO:0046914 | transition metal ion binding | 901 | 110 | 67.76 | 1.90E-07 | 9.46E-06 |  | 132 | 92.88 | 1.50E-05 | 0.000647 |
| GO:0005509 | calcium ion binding | 208 | 15 | 15.64 | 0.60592 | 1 |  | 34 | 21.44 | 0.00449 | 0.07085439 |
| GO:0043565 | sequence-specific DNA binding | 158 | 17 | 11.88 | 0.08549 | 0.691546322 |  | 31 | 16.29 | 0.00032 | 0.007668148 |
| GO:0016702 | oxidoreductase activity, acting on singl... | 46 | 3 | 3.46 | 0.68273 | 1 |  | 16 | 6.08 | 0.00022 | 0.00647 |
| GO:0016746 | transferase activity, transferring acyl ... | 254 | 24 | 19.1 | 0.14595 | 0.890845755 |  | 37 | 26.18 | 0.01936 | 0.223677143 |
| GO:0016705 | oxidoreductase activity, acting on paire... | 334 | 66 | 25.12 | 2.40E-13 | 7.76E-11 |  | 78 | 34.43 | 2.30E-12 | 1.49E-09 |
| GO:0016747 | transferase activity, transferring acyl ... | 182 | 16 | 13.69 | 0.29411 | 1 |  | 28 | 18.76 | 0.02013 | 0.224553621 |
| GO:0051213 | dioxygenase activity | 46 | 3 | 3.46 | 0.68273 | 1 |  | 14 | 4.74 | 0.00014 | 0.005032222 |
| GO:0015297 | antiporter activity | 98 | 11 | 7.37 | 0.11758 | 0.818002796 |  | 21 | 10.1 | 0.00087 | 0.018763 |
| GO:0016788 | hydrolase activity, acting on ester bond... | 402 | 50 | 30.23 | 0.00029 | 0.006052581 |  | 60 | 41.44 | 0.00209 | 0.036546757 |
| GO:0016757 | transferase activity, transferring glyco... | 462 | 57 | 34.74 | 0.00014 | 0.003019333 |  | 67 | 47.63 | 0.00247 | 0.042055 |
| GO:0015291 | secondary active transmembrane transport... | 122 | 11 | 9.17 | 0.31043 | 1 |  | 21 | 12.58 | 0.01286 | 0.160754615 |
| GO:0015238 | drug transmembrane transporter activity | 56 | 5 | 4.21 | 0.41399 | 1 |  | 14 | 5.77 | 0.0013 | 0.024031429 |
| GO:0090484 | drug transporter activity | 56 | 5 | 4.21 | 0.41399 | 1 |  | 14 | 5.77 | 0.0013 | 0.024031429 |
| GO:0016701 | oxidoreductase activity, acting on singl... | 59 | 6 | 4.44 | 0.28222 | 1 |  | 14 | 4.74 | 0.00014 | 0.005032222 |
| GO:0016758 | transferase activity, transferring hexos... | 360 | 51 | 27.07 | 8.20E-06 | 0.000241155 |  | 59 | 37.11 | 0.00021 | 0.00647 |
| GO:0016798 | hydrolase activity, acting on glycosyl b... | 440 | 58 | 33.09 | 1.90E-05 | 0.000512208 |  | 66 | 45.36 | 0.00113 | 0.022154848 |
| GO:0020037 | heme binding | 389 | 72 | 29.25 | 5.60E-13 | 1.21E-10 |  | 78 | 40.1 | 5.20E-09 | 1.12E-06 |
| GO:0046906 | tetrapyrrole binding | 392 | 72 | 29.48 | 8.30E-13 | 1.34E-10 |  | 78 | 40.41 | 7.40E-09 | 1.20E-06 |
| GO:0016717 | oxidoreductase activity, acting on paire... | 24 | 3 | 1.8 | 0.26839 | 1 |  | 9 | 2.47 | 0.0004 | 0.009242857 |
| GO:0004674 | protein serine/threonine kinase activity | 34 | 2 | 2.56 | 0.73653 | 1 |  | 8 | 3.5 | 0.01981 | 0.224553621 |
| GO:0005506 | iron ion binding | 323 | 65 | 24.29 | 1.50E-13 | 7.76E-11 |  | 70 | 33.3 | 1.10E-09 | 3.56E-07 |
| GO:0004553 | hydrolase activity, hydrolyzing O-glycos... | 399 | 53 | 30.01 | 3.40E-05 | 0.000814741 |  | 58 | 41.13 | 0.00441 | 0.07085439 |
| GO:0005516 | calmodulin binding | 30 | 3 | 2.26 | 0.39494 | 1 |  | 8 | 3.09 | 0.00924 | 0.1206008 |
| GO:0030234 | enzyme regulator activity | 186 | 33 | 13.99 | 3.00E-06 | 0.000114176 |  | 28 | 19.17 | 0.0261 | 0.276831148 |
| GO:0015036 | disulfide oxidoreductase activity | 43 | 4 | 3.23 | 0.40701 | 1 |  | 9 | 4.43 | 0.02881 | 0.286770308 |
| GO:0015035 | protein disulfide oxidoreductase activit... | 43 | 4 | 3.23 | 0.40701 | 1 |  | 9 | 4.43 | 0.02881 | 0.286770308 |
| GO:0016760 | cellulose synthase (UDP-forming) activit... | 49 | 9 | 3.69 | 0.00992 | 0.156542439 |  | 13 | 5.05 | 0.00106 | 0.021431875 |
| GO:0016759 | cellulose synthase activity | 49 | 9 | 3.69 | 0.00992 | 0.156542439 |  | 13 | 5.05 | 0.00106 | 0.021431875 |
| GO:0001871 | pattern binding | 23 | 2 | 1.73 | 0.52484 | 1 |  | 6 | 2.37 | 0.02577 | 0.276831148 |
| GO:0030247 | polysaccharide binding | 23 | 2 | 1.73 | 0.52484 | 1 |  | 6 | 2.37 | 0.02577 | 0.276831148 |
| GO:0004857 | enzyme inhibitor activity | 117 | 28 | 8.8 | 2.80E-08 | 1.51E-06 |  | 25 | 12.06 | 0.00031 | 0.007668148 |
| GO:0004866 | endopeptidase inhibitor activity | 60 | 16 | 4.51 | 5.90E-06 | 0.000181776 |  | 13 | 6.19 | 0.00719 | 0.103376222 |
| GO:0061135 | endopeptidase regulator activity | 60 | 16 | 4.51 | 5.90E-06 | 0.000181776 |  | 13 | 6.19 | 0.00719 | 0.103376222 |
| GO:0030414 | peptidase inhibitor activity | 60 | 16 | 4.51 | 5.90E-06 | 0.000181776 |  | 13 | 6.19 | 0.00719 | 0.103376222 |
| GO:0061134 | peptidase regulator activity | 60 | 16 | 4.51 | 5.90E-06 | 0.000181776 |  | 13 | 6.19 | 0.00719 | 0.103376222 |
| GO:0016799 | hydrolase activity, hydrolyzing N-glycos... | 20 | 3 | 1.5 | 0.18694 | 1 |  | 6 | 2.06 | 0.01292 | 0.160754615 |
| GO:0003968 | RNA-directed 5'-3' RNA polymerase activi... | 21 | 3 | 1.58 | 0.20673 | 1 |  | 6 | 2.16 | 0.01655 | 0.202034906 |
| GO:0019900 | kinase binding | 5 | 1 | 0.38 | 0.3236 | 1 |  | 3 | 0.52 | 0.00932 | 0.1206008 |
| GO:0019901 | protein kinase binding | 5 | 1 | 0.38 | 0.3236 | 1 |  | 3 | 0.52 | 0.00932 | 0.1206008 |
| GO:0046527 | glucosyltransferase activity | 96 | 19 | 7.22 | 8.20E-05 | 0.001829448 |  | 17 | 9.9 | 0.01821 | 0.214215818 |
| GO:0004523 | RNA-DNA hybrid ribonuclease activity | 91 | 27 | 6.84 | 3.00E-10 | 3.88E-08 |  | 26 | 9.38 | 9.40E-07 | 7.60E-05 |
| GO:0004540 | ribonuclease activity | 124 | 30 | 9.33 | 7.00E-09 | 4.53E-07 |  | 31 | 12.78 | 2.20E-06 | 0.0001294 |
| GO:0004568 | chitinase activity | 27 | 13 | 2.03 | 1.70E-08 | 1.00E-06 |  | 12 | 2.78 | 5.50E-06 | 0.000273731 |
| GO:0004418 | hydroxymethylbilane synthase activity | 5 | 2 | 0.38 | 0.04849 | 0.522883833 |  | 3 | 0.52 | 0.00932 | 0.1206008 |
| GO:0030597 | RNA glycosylase activity | 7 | 2 | 0.53 | 0.09212 | 0.691546322 |  | 3 | 0.72 | 0.02783 | 0.285809683 |
| GO:0030598 | rRNA N-glycosylase activity | 7 | 2 | 0.53 | 0.09212 | 0.691546322 |  | 3 | 0.72 | 0.02783 | 0.285809683 |
| GO:0004521 | endoribonuclease activity | 113 | 30 | 8.5 | 6.40E-10 | 6.90E-08 |  | 30 | 11.65 | 8.00E-07 | 7.39E-05 |
| GO:0016893 | endonuclease activity, active with eithe... | 99 | 27 | 7.45 | 2.40E-09 | 1.73E-07 |  | 27 | 10.21 | 1.60E-06 | 0.00010352 |
| GO:0016891 | endoribonuclease activity, producing 5'-... | 99 | 27 | 7.45 | 2.40E-09 | 1.73E-07 |  | 27 | 10.21 | 1.60E-06 | 0.00010352 |
| GO:0004519 | endonuclease activity | 125 | 31 | 9.4 | 2.10E-09 | 1.73E-07 |  | 31 | 12.89 | 2.60E-06 | 0.000140183 |
| GO:0008061 | chitin binding | 32 | 13 | 2.41 | 2.10E-07 | 9.71E-06 |  | 13 | 3.3 | 7.50E-06 | 0.000346607 |
| GO:0004518 | nuclease activity | 170 | 33 | 12.78 | 3.70E-07 | 1.60E-05 |  | 33 | 17.52 | 0.00025 | 0.006739583 |
| GO:0004819 | glutamine-tRNA ligase activity | 5 | 3 | 0.38 | 0.00378 | 0.071931176 |  | 3 | 0.52 | 0.00932 | 0.1206008 |
| GO:0004061 | arylformamidase activity | 6 | 3 | 0.45 | 0.00714 | 0.124853514 |  | 3 | 0.62 | 0.01721 | 0.206201296 |
| GO:0016843 | amine-lyase activity | 8 | 3 | 0.6 | 0.01783 | 0.256355778 |  | 3 | 0.82 | 0.04118 | 0.397663582 |
| GO:0016844 | strictosidine synthase activity | 8 | 3 | 0.6 | 0.01783 | 0.256355778 |  | 3 | 0.82 | 0.04118 | 0.397663582 |
