# Supplementary material for: Comparative transcriptomic analysis reveals the cold acclimation during chilling stress in sensitive and resistant passion fruit (Passiflora edulis) cultivars
Source: PeerJ. 2021 Mar 3;9:e10977. doi: 10.7717/peerj.10977 (PMC7936571; doi:10.7717/peerj.10977)
Supplement: Supplemental Information 5 [file peerj-09-10977-s005.docx]

**Supplementary table 5 Highly expressed unigene sets**

| Gene_ID | Module | KME |
| --- | --- | --- |
| TRINITY_DN32883_c1_g4_i1 | black | 0.9940875 |
| TRINITY_DN27433_c0_g1_i2 | black | 0.9927759 |
| TRINITY_DN34111_c1_g1_i1 | black | 0.9903968 |
| TRINITY_DN22937_c0_g4_i1 | black | 0.9885131 |
| TRINITY_DN33031_c0_g1_i1 | black | 0.9875659 |
| TRINITY_DN24978_c0_g2_i1 | black | 0.986303 |
| TRINITY_DN38936_c0_g1_i1 | black | 0.9846654 |
| TRINITY_DN69117_c0_g1_i1 | black | 0.9834449 |
| TRINITY_DN39127_c1_g10_i1 | black | 0.9827546 |
| TRINITY_DN30197_c0_g1_i3 | black | 0.9813841 |
| TRINITY_DN29304_c0_g3_i1 | black | 0.9811728 |
| TRINITY_DN34618_c1_g9_i1 | black | 0.9796289 |
| TRINITY_DN39106_c1_g2_i6 | black | 0.9770519 |
| TRINITY_DN39165_c1_g6_i6 | black | 0.9762605 |
| TRINITY_DN26920_c0_g1_i4 | black | 0.9756251 |
| TRINITY_DN35406_c2_g1_i3 | black | 0.9747631 |
| TRINITY_DN36064_c0_g3_i3 | black | 0.9738637 |
| TRINITY_DN37319_c0_g1_i2 | black | 0.9736888 |
| TRINITY_DN36083_c0_g1_i3 | black | 0.9734002 |
| TRINITY_DN39275_c3_g1_i1 | black | 0.9731032 |
| TRINITY_DN20407_c0_g2_i1 | black | 0.9708269 |
| TRINITY_DN23479_c0_g1_i1 | black | 0.9707866 |
| TRINITY_DN23814_c0_g1_i1 | black | 0.9706574 |
| TRINITY_DN24961_c0_g1_i1 | black | 0.9704825 |
| TRINITY_DN20903_c0_g1_i3 | black | 0.9703899 |
| TRINITY_DN37614_c1_g6_i1 | black | 0.9695526 |
| TRINITY_DN37459_c0_g3_i2 | black | 0.9690473 |
| TRINITY_DN37770_c1_g2_i1 | black | 0.9687504 |
| TRINITY_DN29304_c0_g2_i1 | black | 0.968629 |
| TRINITY_DN29980_c0_g2_i1 | black | 0.9685518 |
| TRINITY_DN39325_c4_g4_i3 | black | 0.9680147 |
| TRINITY_DN38310_c0_g3_i1 | black | 0.9677275 |
| TRINITY_DN23956_c0_g2_i5 | black | 0.966867 |
| TRINITY_DN38365_c1_g4_i1 | black | 0.9665138 |
| TRINITY_DN32062_c1_g1_i1 | black | 0.9663656 |
| TRINITY_DN31476_c1_g1_i3 | black | 0.9659113 |
| TRINITY_DN37411_c4_g7_i2 | black | 0.9653954 |
| TRINITY_DN30568_c0_g3_i2 | black | 0.9651023 |
| TRINITY_DN30054_c0_g4_i6 | black | 0.9648681 |
| TRINITY_DN30887_c0_g1_i3 | black | 0.9647811 |
| TRINITY_DN31902_c1_g1_i2 | black | 0.9639337 |
| TRINITY_DN36007_c0_g1_i1 | black | 0.9638289 |
| TRINITY_DN38951_c1_g2_i1 | black | 0.9638208 |
| TRINITY_DN37750_c0_g7_i1 | black | 0.9637623 |
| TRINITY_DN32412_c0_g1_i10 | black | 0.9612801 |
| TRINITY_DN35672_c0_g2_i1 | black | 0.9606677 |
| TRINITY_DN38057_c0_g9_i1 | black | 0.9601715 |
| TRINITY_DN36409_c0_g1_i4 | black | 0.9589634 |
| TRINITY_DN30074_c1_g27_i1 | black | 0.9586467 |
| TRINITY_DN18966_c0_g1_i1 | black | 0.9583833 |
| TRINITY_DN39294_c6_g12_i5 | black | 0.9581044 |
| TRINITY_DN33151_c0_g1_i15 | black | 0.9577486 |
| TRINITY_DN30229_c0_g2_i1 | black | 0.9574561 |
| TRINITY_DN33944_c1_g2_i1 | black | 0.9565324 |
| TRINITY_DN37614_c0_g1_i2 | black | 0.9564561 |
| TRINITY_DN32924_c1_g6_i2 | black | 0.9561215 |
| TRINITY_DN37884_c0_g4_i1 | black | 0.9560816 |
| TRINITY_DN29539_c0_g4_i1 | black | 0.9558477 |
| TRINITY_DN33128_c0_g1_i4 | black | 0.9553933 |
| TRINITY_DN27304_c0_g4_i1 | black | 0.955347 |
| TRINITY_DN31476_c1_g7_i1 | black | 0.9552502 |
| TRINITY_DN24686_c0_g1_i1 | black | 0.9545149 |
| TRINITY_DN38614_c0_g1_i13 | black | 0.9534311 |
| TRINITY_DN25671_c0_g4_i1 | black | 0.9524508 |
| TRINITY_DN38586_c3_g7_i1 | black | 0.9521545 |
| TRINITY_DN39325_c4_g2_i4 | black | 0.9519807 |
| TRINITY_DN28161_c0_g1_i1 | black | 0.9517538 |
| TRINITY_DN35012_c1_g2_i2 | black | 0.9496084 |
| TRINITY_DN34338_c0_g1_i2 | black | 0.9494323 |
| TRINITY_DN22771_c1_g2_i1 | black | 0.948903 |
| TRINITY_DN25919_c1_g1_i3 | black | 0.9486164 |
| TRINITY_DN32295_c0_g3_i6 | black | 0.9480803 |
| TRINITY_DN36235_c0_g1_i4 | black | 0.9479861 |
| TRINITY_DN32334_c0_g1_i3 | black | 0.9478557 |
| TRINITY_DN38492_c1_g4_i1 | black | 0.9472182 |
| TRINITY_DN39096_c1_g1_i2 | black | 0.9456833 |
| TRINITY_DN38712_c2_g6_i1 | black | 0.9456024 |
| TRINITY_DN39147_c0_g2_i1 | black | 0.9453899 |
| TRINITY_DN22051_c0_g2_i1 | black | 0.9447722 |
| TRINITY_DN28947_c1_g1_i3 | black | 0.9437864 |
| TRINITY_DN31158_c0_g1_i3 | black | 0.943663 |
| TRINITY_DN28396_c0_g1_i2 | black | 0.9431476 |
| TRINITY_DN34351_c0_g1_i3 | black | 0.9430133 |
| TRINITY_DN27882_c0_g1_i3 | black | 0.9421037 |
| TRINITY_DN33416_c0_g4_i6 | black | 0.9412884 |
| TRINITY_DN25402_c0_g2_i2 | black | 0.9412468 |
| TRINITY_DN36605_c1_g5_i5 | black | 0.9412161 |
| TRINITY_DN21660_c0_g3_i1 | black | 0.9410499 |
| TRINITY_DN35677_c1_g7_i1 | black | 0.9404365 |
| TRINITY_DN28206_c0_g1_i8 | black | 0.9402243 |
| TRINITY_DN33922_c1_g1_i1 | black | 0.9393096 |
| TRINITY_DN23028_c0_g2_i3 | black | 0.9384327 |
| TRINITY_DN25724_c0_g1_i2 | black | 0.9372624 |
| TRINITY_DN26364_c0_g2_i1 | black | 0.9372477 |
| TRINITY_DN18571_c0_g1_i1 | black | 0.936997 |
| TRINITY_DN35610_c1_g2_i1 | black | 0.9365576 |
| TRINITY_DN32947_c1_g2_i1 | black | 0.9356879 |
| TRINITY_DN36432_c0_g5_i1 | black | 0.9352363 |
| TRINITY_DN28019_c0_g1_i2 | black | 0.9339897 |
| TRINITY_DN26920_c0_g3_i1 | black | 0.9339703 |
| TRINITY_DN34704_c5_g5_i4 | black | 0.9335595 |
| TRINITY_DN35085_c1_g3_i8 | black | 0.9332864 |
| TRINITY_DN37621_c0_g3_i1 | black | 0.9325922 |
| TRINITY_DN15984_c0_g1_i1 | black | 0.931093 |
| TRINITY_DN25433_c0_g1_i1 | black | 0.9308009 |
| TRINITY_DN36595_c1_g2_i4 | black | 0.9304725 |
| TRINITY_DN22288_c0_g1_i1 | black | 0.9299753 |
| TRINITY_DN38496_c1_g7_i1 | black | 0.928991 |
| TRINITY_DN31476_c1_g6_i2 | black | 0.9287046 |
| TRINITY_DN22638_c0_g2_i2 | black | 0.9283005 |
| TRINITY_DN38992_c0_g2_i3 | black | 0.9282396 |
| TRINITY_DN33481_c0_g1_i12 | black | 0.9263978 |
| TRINITY_DN39343_c18_g20_i1 | black | 0.9263018 |
| TRINITY_DN23045_c0_g2_i1 | black | 0.9259758 |
| TRINITY_DN32057_c0_g2_i10 | black | 0.9254453 |
| TRINITY_DN28451_c0_g1_i10 | black | 0.925085 |
| TRINITY_DN36424_c0_g1_i2 | black | 0.9246689 |
| TRINITY_DN23347_c0_g1_i1 | black | 0.9242588 |
| TRINITY_DN32566_c1_g2_i2 | black | 0.9232403 |
| TRINITY_DN23808_c0_g2_i1 | black | 0.9230094 |
| TRINITY_DN36755_c0_g3_i1 | black | 0.9226003 |
| TRINITY_DN32058_c0_g2_i3 | black | 0.9217764 |
| TRINITY_DN38226_c0_g2_i1 | black | 0.9203847 |
| TRINITY_DN62688_c0_g1_i1 | black | 0.9200974 |
| TRINITY_DN25934_c0_g1_i1 | black | 0.9197009 |
| TRINITY_DN35672_c0_g1_i1 | black | 0.9194343 |
| TRINITY_DN22179_c0_g1_i1 | black | 0.9190608 |
| TRINITY_DN38254_c1_g6_i1 | black | 0.9190591 |
| TRINITY_DN39294_c6_g2_i1 | black | 0.9181736 |
| TRINITY_DN35110_c0_g3_i1 | black | 0.9171386 |
| TRINITY_DN31454_c0_g6_i1 | black | 0.9157476 |
| TRINITY_DN35056_c1_g2_i3 | black | 0.9155567 |
| TRINITY_DN29515_c1_g1_i1 | black | 0.9154234 |
| TRINITY_DN33305_c0_g5_i1 | black | 0.9152653 |
| TRINITY_DN37767_c0_g6_i1 | black | 0.9148736 |
| TRINITY_DN37758_c1_g2_i8 | black | 0.91365 |
| TRINITY_DN29353_c0_g1_i2 | black | 0.9136186 |
| TRINITY_DN37611_c0_g2_i2 | black | 0.9133031 |
| TRINITY_DN38701_c1_g7_i1 | black | 0.9130982 |
| TRINITY_DN16380_c0_g2_i1 | black | 0.9123361 |
| TRINITY_DN22042_c0_g2_i1 | black | 0.9116232 |
| TRINITY_DN34909_c2_g2_i2 | black | 0.9107969 |
| TRINITY_DN35535_c1_g14_i10 | black | 0.9101191 |
| TRINITY_DN35457_c3_g3_i1 | black | 0.908473 |
| TRINITY_DN36114_c0_g2_i1 | black | 0.9075536 |
| TRINITY_DN28290_c0_g1_i8 | black | 0.9065452 |
| TRINITY_DN38826_c0_g4_i1 | black | 0.9062459 |
| TRINITY_DN38408_c3_g1_i24 | black | 0.9033482 |
| TRINITY_DN26713_c0_g2_i1 | black | 0.9015796 |
| TRINITY_DN37690_c0_g4_i8 | black | 0.9011977 |
| TRINITY_DN21995_c0_g3_i1 | black | 0.9002646 |
| TRINITY_DN63254_c0_g1_i1 | black | 0.8993004 |
| TRINITY_DN36813_c1_g3_i1 | black | 0.8988159 |
| TRINITY_DN38286_c0_g2_i1 | black | 0.8987329 |
| TRINITY_DN29985_c0_g1_i1 | black | 0.8979393 |
| TRINITY_DN30011_c3_g13_i1 | black | 0.8978884 |
| TRINITY_DN33642_c0_g1_i1 | black | 0.8972253 |
| TRINITY_DN29833_c0_g1_i2 | black | 0.8963808 |
| TRINITY_DN30644_c0_g4_i1 | black | 0.8960923 |
| TRINITY_DN1982_c0_g1_i1 | black | 0.8950953 |
| TRINITY_DN33986_c0_g2_i1 | black | 0.8934396 |
| TRINITY_DN27367_c0_g4_i1 | black | 0.893065 |
| TRINITY_DN22435_c0_g1_i1 | black | 0.8924826 |
| TRINITY_DN27034_c0_g2_i2 | black | 0.8919971 |
| TRINITY_DN38113_c1_g6_i1 | black | 0.8914841 |
| TRINITY_DN63786_c0_g1_i1 | black | 0.8913572 |
| TRINITY_DN38386_c0_g4_i1 | black | 0.8909824 |
| TRINITY_DN23391_c0_g2_i1 | black | 0.8907614 |
| TRINITY_DN39237_c4_g4_i1 | black | 0.8906248 |
| TRINITY_DN33909_c0_g2_i8 | black | 0.8898285 |
| TRINITY_DN38464_c0_g2_i1 | black | 0.8892116 |
| TRINITY_DN31207_c0_g1_i2 | black | 0.8885907 |
| TRINITY_DN4782_c0_g3_i1 | black | 0.8883742 |
| TRINITY_DN34503_c1_g2_i1 | black | 0.8883543 |
| TRINITY_DN38977_c0_g1_i1 | black | 0.8880122 |
| TRINITY_DN38040_c0_g3_i5 | black | 0.8866612 |
| TRINITY_DN34071_c2_g5_i2 | black | 0.8865383 |
| TRINITY_DN32936_c0_g1_i3 | black | 0.8860566 |
| TRINITY_DN37671_c1_g4_i1 | black | 0.8847059 |
| TRINITY_DN36822_c2_g2_i13 | black | 0.8845877 |
| TRINITY_DN22081_c0_g2_i1 | black | 0.8831261 |
| TRINITY_DN27217_c0_g1_i1 | black | 0.8826462 |
| TRINITY_DN9781_c0_g1_i1 | black | 0.8821821 |
| TRINITY_DN23434_c0_g3_i1 | black | 0.8817797 |
| TRINITY_DN32488_c0_g2_i1 | black | 0.8811976 |
| TRINITY_DN34344_c1_g2_i1 | black | 0.8800764 |
| TRINITY_DN31660_c0_g1_i11 | black | 0.8793551 |
| TRINITY_DN36043_c0_g1_i1 | black | 0.8788205 |
| TRINITY_DN16400_c0_g3_i2 | black | 0.8787616 |
| TRINITY_DN38280_c0_g2_i4 | black | 0.8780603 |
| TRINITY_DN36827_c0_g1_i4 | black | 0.8764863 |
| TRINITY_DN32740_c0_g4_i3 | black | 0.8759933 |
| TRINITY_DN33172_c0_g1_i4 | black | 0.875606 |
| TRINITY_DN31067_c0_g1_i4 | black | 0.8747894 |
| TRINITY_DN36269_c0_g8_i1 | black | 0.8747424 |
| TRINITY_DN25864_c0_g2_i1 | black | 0.8746281 |
| TRINITY_DN33873_c0_g2_i9 | black | 0.8742706 |
| TRINITY_DN32334_c0_g3_i2 | black | 0.8738859 |
| TRINITY_DN37306_c0_g6_i2 | black | 0.8737058 |
| TRINITY_DN23919_c0_g1_i2 | black | 0.8732471 |
| TRINITY_DN38084_c0_g1_i8 | black | 0.8719048 |
| TRINITY_DN28427_c0_g1_i2 | black | 0.8710513 |
| TRINITY_DN36154_c0_g3_i7 | black | 0.8709893 |
| TRINITY_DN25347_c0_g1_i2 | black | 0.8706106 |
| TRINITY_DN36065_c0_g5_i5 | black | 0.8704233 |
| TRINITY_DN24584_c0_g5_i1 | black | 0.8701922 |
| TRINITY_DN36082_c0_g3_i1 | black | 0.8696531 |
| TRINITY_DN24480_c0_g1_i1 | black | 0.8693192 |
| TRINITY_DN21780_c0_g2_i1 | black | 0.8686224 |
| TRINITY_DN34930_c0_g6_i1 | black | 0.8680544 |
| TRINITY_DN31773_c3_g9_i1 | black | 0.8680221 |
| TRINITY_DN36534_c2_g4_i4 | black | 0.8679007 |
| TRINITY_DN4346_c0_g1_i1 | black | 0.8662683 |
| TRINITY_DN32314_c0_g1_i1 | black | 0.8657375 |
| TRINITY_DN35677_c1_g6_i1 | black | 0.8656055 |
| TRINITY_DN38569_c0_g3_i1 | black | 0.8652447 |
| TRINITY_DN31956_c1_g4_i3 | black | 0.8642206 |
| TRINITY_DN37771_c0_g2_i7 | black | 0.8637458 |
| TRINITY_DN26454_c0_g1_i2 | black | 0.8632201 |
| TRINITY_DN21029_c0_g1_i1 | black | 0.8620637 |
| TRINITY_DN24994_c0_g1_i1 | black | 0.8620132 |
| TRINITY_DN40764_c0_g1_i1 | black | 0.861413 |
| TRINITY_DN37910_c0_g2_i3 | black | 0.8613587 |
| TRINITY_DN67451_c0_g1_i1 | black | 0.8606151 |
| TRINITY_DN38681_c1_g2_i4 | black | 0.8586007 |
| TRINITY_DN16402_c0_g2_i1 | black | 0.857597 |
| TRINITY_DN35387_c2_g18_i2 | black | 0.8574155 |
| TRINITY_DN27512_c0_g1_i3 | black | 0.8561465 |
| TRINITY_DN37285_c0_g6_i5 | black | 0.8560255 |
| TRINITY_DN38980_c0_g5_i1 | black | 0.8535848 |
| TRINITY_DN25649_c0_g1_i1 | black | 0.8515315 |
| TRINITY_DN34470_c0_g4_i1 | black | 0.8510749 |
| TRINITY_DN9110_c0_g3_i1 | black | 0.848957 |
| TRINITY_DN30971_c0_g3_i1 | black | 0.8487687 |
| TRINITY_DN36325_c0_g2_i1 | black | 0.8479839 |
| TRINITY_DN17612_c0_g1_i1 | black | 0.8478951 |
| TRINITY_DN22340_c0_g1_i1 | black | 0.8477009 |
| TRINITY_DN33236_c0_g3_i1 | black | 0.8474172 |
| TRINITY_DN17128_c0_g2_i1 | black | 0.8473346 |
| TRINITY_DN22954_c0_g1_i1 | black | 0.8462656 |
| TRINITY_DN37983_c1_g2_i1 | black | 0.845545 |
| TRINITY_DN33147_c1_g1_i4 | black | 0.8447941 |
| TRINITY_DN38356_c0_g5_i1 | black | 0.8444 |
| TRINITY_DN38649_c1_g1_i1 | black | 0.8442631 |
| TRINITY_DN31066_c0_g4_i4 | black | 0.8431019 |
| TRINITY_DN24693_c0_g1_i1 | black | 0.8423204 |
| TRINITY_DN23972_c0_g1_i1 | black | 0.8420051 |
| TRINITY_DN24090_c0_g1_i2 | black | 0.8419484 |
| TRINITY_DN39327_c7_g13_i1 | black | 0.8417477 |
| TRINITY_DN27091_c1_g1_i1 | black | 0.8415685 |
| TRINITY_DN35964_c0_g7_i9 | black | 0.841517 |
| TRINITY_DN32566_c1_g1_i4 | black | 0.8413619 |
| TRINITY_DN35878_c3_g7_i3 | black | 0.8413102 |
| TRINITY_DN27158_c0_g2_i1 | black | 0.8411109 |
| TRINITY_DN18705_c0_g1_i2 | black | 0.8406722 |
| TRINITY_DN26969_c0_g1_i1 | black | 0.8405679 |
| TRINITY_DN33832_c0_g1_i2 | black | 0.8400915 |
| TRINITY_DN36051_c0_g11_i1 | black | 0.8398545 |
| TRINITY_DN39309_c2_g3_i4 | black | 0.8398435 |
| TRINITY_DN25669_c0_g2_i1 | black | 0.8392771 |
| TRINITY_DN36570_c1_g3_i1 | black | 0.8378155 |
| TRINITY_DN25052_c0_g3_i1 | black | 0.8377505 |
| TRINITY_DN19816_c0_g1_i1 | black | 0.8372199 |
| TRINITY_DN25675_c0_g1_i1 | black | 0.8369748 |
| TRINITY_DN31540_c1_g1_i15 | black | 0.836609 |
| TRINITY_DN34123_c1_g2_i1 | black | 0.8360581 |
| TRINITY_DN36332_c0_g10_i1 | black | 0.8329879 |
| TRINITY_DN9110_c0_g4_i1 | black | 0.8329405 |
| TRINITY_DN18466_c0_g1_i1 | black | 0.8323832 |
| TRINITY_DN18764_c0_g1_i1 | black | 0.8309907 |
| TRINITY_DN39293_c3_g4_i1 | black | 0.8306443 |
| TRINITY_DN25643_c0_g1_i1 | black | 0.8284702 |
| TRINITY_DN37723_c0_g1_i16 | black | 0.8267719 |
| TRINITY_DN28419_c1_g5_i5 | black | 0.8266109 |
| TRINITY_DN24101_c0_g1_i3 | black | 0.8259634 |
| TRINITY_DN27106_c0_g3_i1 | black | 0.824936 |
| TRINITY_DN33845_c0_g3_i3 | black | 0.8243074 |
| TRINITY_DN35916_c1_g1_i21 | black | 0.8240144 |
| TRINITY_DN32992_c0_g1_i7 | black | 0.8236265 |
| TRINITY_DN70545_c0_g1_i1 | black | 0.8234732 |
| TRINITY_DN4173_c0_g1_i1 | black | 0.8213815 |
| TRINITY_DN32125_c0_g9_i2 | black | 0.8213302 |
| TRINITY_DN23481_c0_g1_i2 | black | 0.821094 |
| TRINITY_DN24883_c0_g1_i1 | black | 0.8201307 |
| TRINITY_DN30282_c0_g2_i1 | black | 0.8197778 |
| TRINITY_DN36284_c0_g1_i4 | black | 0.8192945 |
| TRINITY_DN28013_c0_g1_i2 | black | 0.8189222 |
| TRINITY_DN34714_c2_g1_i1 | black | 0.8168744 |
| TRINITY_DN25633_c0_g1_i1 | black | 0.8154307 |
| TRINITY_DN36860_c0_g4_i1 | black | 0.8139835 |
| TRINITY_DN39129_c2_g6_i1 | black | 0.8106567 |
| TRINITY_DN34082_c0_g3_i1 | black | 0.8098507 |
| TRINITY_DN35400_c0_g4_i1 | black | 0.8091213 |
| TRINITY_DN28655_c0_g1_i4 | black | 0.8088415 |
| TRINITY_DN36082_c0_g9_i1 | black | 0.8080375 |
| TRINITY_DN38066_c4_g1_i2 | black | 0.8074047 |
| TRINITY_DN28877_c0_g1_i3 | black | 0.8069064 |
| TRINITY_DN23389_c1_g2_i1 | black | 0.8047685 |
| TRINITY_DN37513_c0_g2_i4 | black | 0.8043651 |
| TRINITY_DN22947_c0_g1_i1 | black | 0.804342 |
| TRINITY_DN36671_c0_g2_i17 | black | 0.8034879 |
| TRINITY_DN38731_c4_g6_i2 | black | 0.8022743 |
| TRINITY_DN70436_c0_g1_i1 | black | 0.8019858 |
| TRINITY_DN30843_c0_g6_i2 | black | 0.801517 |
| TRINITY_DN31593_c3_g2_i1 | black | 0.8014206 |
| TRINITY_DN30858_c0_g9_i1 | black | 0.8011086 |
| TRINITY_DN35633_c0_g1_i3 | black | 0.8000648 |
| TRINITY_DN26770_c0_g2_i1 | black | 0.7990961 |
| TRINITY_DN26448_c0_g3_i1 | black | 0.7990499 |
| TRINITY_DN36434_c0_g4_i1 | black | 0.7977046 |
| TRINITY_DN2748_c0_g1_i1 | black | 0.7973447 |
| TRINITY_DN8521_c0_g1_i1 | black | 0.7972872 |
| TRINITY_DN31214_c0_g1_i1 | black | 0.7965433 |
| TRINITY_DN32886_c1_g1_i2 | black | 0.7957389 |
| TRINITY_DN38632_c0_g2_i1 | black | 0.7954578 |
| TRINITY_DN35159_c0_g1_i1 | black | 0.7950451 |
| TRINITY_DN38017_c0_g5_i3 | black | 0.7946083 |
| TRINITY_DN39040_c2_g2_i4 | black | 0.7944427 |
| TRINITY_DN26865_c0_g1_i4 | black | 0.7939071 |
| TRINITY_DN39340_c19_g10_i1 | black | 0.7929264 |
| TRINITY_DN27125_c0_g1_i1 | black | 0.7927001 |
| TRINITY_DN35955_c3_g5_i1 | black | 0.7921371 |
| TRINITY_DN25763_c0_g1_i1 | black | 0.7910939 |
| TRINITY_DN5243_c0_g1_i1 | black | 0.7893443 |
| TRINITY_DN34412_c0_g2_i1 | black | 0.7881927 |
| TRINITY_DN26058_c0_g1_i1 | black | 0.7881708 |
| TRINITY_DN58449_c0_g2_i1 | black | 0.7880828 |
| TRINITY_DN36228_c1_g1_i1 | black | 0.7876532 |
| TRINITY_DN40385_c0_g1_i1 | black | 0.7848065 |
| TRINITY_DN25229_c0_g3_i1 | black | 0.7843534 |
| TRINITY_DN45305_c0_g1_i1 | black | 0.7841349 |
| TRINITY_DN35213_c2_g3_i1 | black | 0.7826658 |
| TRINITY_DN32831_c0_g3_i4 | black | 0.7807963 |
| TRINITY_DN30661_c1_g2_i1 | black | 0.7799245 |
| TRINITY_DN38748_c0_g2_i1 | black | 0.7785122 |
| TRINITY_DN21231_c0_g1_i1 | black | 0.7770218 |
| TRINITY_DN34947_c0_g4_i1 | black | 0.7755389 |
| TRINITY_DN35575_c0_g2_i2 | black | 0.7750395 |
| TRINITY_DN52115_c0_g1_i1 | black | 0.773015 |
| TRINITY_DN25864_c0_g3_i2 | black | 0.7710114 |
| TRINITY_DN30033_c0_g1_i1 | black | 0.77095 |
| TRINITY_DN34976_c0_g1_i1 | black | 0.7705365 |
| TRINITY_DN26755_c0_g1_i1 | black | 0.7697767 |
| TRINITY_DN37605_c0_g1_i2 | black | 0.7687034 |
| TRINITY_DN13677_c0_g2_i1 | black | 0.768169 |
| TRINITY_DN38523_c0_g3_i1 | black | 0.767684 |
| TRINITY_DN35086_c1_g1_i1 | black | 0.7676499 |
| TRINITY_DN31084_c0_g1_i4 | black | 0.7672089 |
| TRINITY_DN39338_c8_g1_i6 | black | 0.7668229 |
| TRINITY_DN21104_c0_g1_i1 | black | 0.7652755 |
| TRINITY_DN24547_c0_g1_i1 | black | 0.7651251 |
| TRINITY_DN22925_c0_g1_i1 | black | 0.7646376 |
| TRINITY_DN37589_c0_g1_i1 | black | 0.7628378 |
| TRINITY_DN25274_c0_g1_i1 | black | 0.7619708 |
| TRINITY_DN34356_c0_g1_i5 | black | 0.7567307 |
| TRINITY_DN1091_c0_g1_i1 | black | 0.7562011 |
| TRINITY_DN38135_c2_g5_i1 | black | 0.7541808 |
| TRINITY_DN32285_c0_g3_i2 | black | 0.7505567 |
| TRINITY_DN67504_c0_g1_i1 | black | 0.7491009 |
| TRINITY_DN22509_c0_g4_i1 | black | 0.74753 |
| TRINITY_DN37088_c0_g2_i1 | black | 0.7432266 |
| TRINITY_DN24821_c0_g2_i1 | black | 0.7392888 |
| TRINITY_DN34374_c1_g1_i2 | black | 0.7392116 |
| TRINITY_DN19358_c0_g1_i1 | black | 0.737175 |
| TRINITY_DN34999_c0_g4_i1 | black | 0.7360424 |
| TRINITY_DN70271_c0_g1_i1 | black | 0.7332207 |
| TRINITY_DN34049_c2_g2_i1 | black | 0.7305138 |
| TRINITY_DN37762_c2_g1_i1 | black | 0.7268366 |
| TRINITY_DN22351_c0_g1_i1 | black | 0.7212474 |
| TRINITY_DN39180_c8_g8_i1 | black | 0.7189605 |
| TRINITY_DN34343_c0_g1_i2 | black | 0.7154152 |
| TRINITY_DN30152_c0_g3_i1 | black | 0.7127728 |
| TRINITY_DN20943_c0_g1_i1 | black | 0.7098155 |
| TRINITY_DN32494_c2_g10_i3 | black | 0.7028968 |
| TRINITY_DN26133_c0_g5_i1 | black | 0.7003445 |
| TRINITY_DN29241_c0_g1_i1 | black | 0.6960916 |
| TRINITY_DN19646_c0_g1_i1 | black | 0.6866614 |
| TRINITY_DN190_c0_g1_i1 | black | 0.6826789 |
| TRINITY_DN34374_c1_g2_i1 | black | 0.6807245 |
| TRINITY_DN23229_c0_g3_i1 | black | 0.656456 |
| TRINITY_DN48305_c0_g1_i1 | black | 0.6432697 |
| TRINITY_DN28053_c0_g2_i1 | black | -0.635462 |
| TRINITY_DN35456_c2_g4_i1 | black | -0.636138 |
| TRINITY_DN16283_c0_g2_i1 | black | -0.673148 |
| TRINITY_DN35213_c2_g1_i1 | black | -0.688364 |
| TRINITY_DN25411_c0_g1_i1 | black | -0.69763 |
| TRINITY_DN31741_c0_g2_i1 | black | -0.705583 |
| TRINITY_DN32348_c0_g2_i1 | black | -0.713313 |
| TRINITY_DN38898_c1_g2_i1 | black | -0.719543 |
| TRINITY_DN38699_c0_g8_i1 | black | -0.721678 |
| TRINITY_DN50746_c0_g2_i1 | black | -0.735082 |
| TRINITY_DN39315_c0_g4_i2 | black | -0.746315 |
| TRINITY_DN34640_c0_g2_i9 | black | -0.747997 |
| TRINITY_DN36047_c0_g1_i1 | black | -0.751043 |
| TRINITY_DN39254_c0_g1_i1 | black | -0.764386 |
| TRINITY_DN25231_c0_g2_i1 | black | -0.769546 |
| TRINITY_DN21176_c0_g4_i1 | black | -0.775314 |
| TRINITY_DN35058_c0_g1_i3 | black | -0.776683 |
| TRINITY_DN22435_c0_g2_i1 | black | -0.777934 |
| TRINITY_DN32690_c0_g1_i3 | black | -0.778579 |
| TRINITY_DN27393_c0_g1_i3 | black | -0.779779 |
| TRINITY_DN39313_c1_g11_i8 | black | -0.780665 |
| TRINITY_DN23834_c0_g1_i1 | black | -0.781784 |
| TRINITY_DN19536_c0_g1_i1 | black | -0.784007 |
| TRINITY_DN26375_c0_g1_i1 | black | -0.786277 |
| TRINITY_DN12662_c0_g1_i1 | black | -0.790414 |
| TRINITY_DN38861_c1_g4_i1 | black | -0.792678 |
| TRINITY_DN20017_c0_g1_i1 | black | -0.793515 |
| TRINITY_DN25027_c0_g1_i1 | black | -0.798703 |
| TRINITY_DN39101_c2_g4_i1 | black | -0.798865 |
| TRINITY_DN39045_c2_g2_i2 | black | -0.799074 |
| TRINITY_DN36490_c1_g1_i8 | black | -0.803589 |
| TRINITY_DN38034_c0_g4_i1 | black | -0.806152 |
| TRINITY_DN36914_c0_g2_i1 | black | -0.806475 |
| TRINITY_DN18800_c0_g1_i2 | black | -0.809101 |
| TRINITY_DN25240_c0_g1_i1 | black | -0.81381 |
| TRINITY_DN28754_c0_g1_i3 | black | -0.819437 |
| TRINITY_DN39190_c2_g2_i4 | black | -0.819489 |
| TRINITY_DN34778_c0_g7_i2 | black | -0.822413 |
| TRINITY_DN38712_c2_g5_i4 | black | -0.825415 |
| TRINITY_DN28028_c0_g1_i4 | black | -0.827021 |
| TRINITY_DN12815_c0_g1_i1 | black | -0.830579 |
| TRINITY_DN38570_c1_g2_i2 | black | -0.831837 |
| TRINITY_DN39292_c4_g3_i1 | black | -0.833833 |
| TRINITY_DN36532_c2_g2_i1 | black | -0.834272 |
| TRINITY_DN33107_c0_g4_i1 | black | -0.841118 |
| TRINITY_DN37081_c1_g1_i6 | black | -0.841327 |
| TRINITY_DN38888_c1_g2_i1 | black | -0.842211 |
| TRINITY_DN37859_c0_g1_i2 | black | -0.842966 |
| TRINITY_DN23704_c0_g1_i1 | black | -0.843609 |
| TRINITY_DN36108_c0_g1_i2 | black | -0.844327 |
| TRINITY_DN22437_c0_g1_i1 | black | -0.852577 |
| TRINITY_DN36019_c0_g4_i7 | black | -0.854556 |
| TRINITY_DN29139_c0_g1_i2 | black | -0.855841 |
| TRINITY_DN38876_c0_g9_i1 | black | -0.860052 |
| TRINITY_DN31908_c0_g1_i1 | black | -0.863387 |
| TRINITY_DN34355_c0_g1_i2 | black | -0.864222 |
| TRINITY_DN37736_c1_g4_i4 | black | -0.875076 |
| TRINITY_DN38339_c1_g3_i1 | black | -0.875452 |
| TRINITY_DN37282_c0_g2_i2 | black | -0.877442 |
| TRINITY_DN32005_c0_g1_i9 | black | -0.880135 |
| TRINITY_DN39205_c1_g7_i2 | black | -0.881098 |
| TRINITY_DN39283_c3_g3_i1 | black | -0.883836 |
| TRINITY_DN38540_c1_g6_i3 | black | -0.887597 |
| TRINITY_DN36552_c0_g1_i3 | black | -0.890307 |
| TRINITY_DN37799_c1_g3_i2 | black | -0.891047 |
| TRINITY_DN24771_c0_g1_i1 | black | -0.891168 |
| TRINITY_DN37912_c0_g1_i3 | black | -0.893121 |
| TRINITY_DN36958_c0_g1_i1 | black | -0.89663 |
| TRINITY_DN36663_c1_g3_i1 | black | -0.897785 |
| TRINITY_DN39207_c0_g2_i2 | black | -0.897906 |
| TRINITY_DN56010_c0_g1_i1 | black | -0.899419 |
| TRINITY_DN22603_c0_g1_i1 | black | -0.906142 |
| TRINITY_DN31919_c0_g3_i1 | black | -0.912639 |
| TRINITY_DN23761_c0_g1_i1 | black | -0.912646 |
| TRINITY_DN38653_c0_g1_i1 | black | -0.917733 |
| TRINITY_DN35668_c0_g1_i6 | black | -0.918654 |
| TRINITY_DN28999_c0_g1_i3 | black | -0.924155 |
| TRINITY_DN38489_c0_g4_i1 | black | -0.925521 |
| TRINITY_DN35360_c0_g4_i1 | black | -0.931437 |
| TRINITY_DN35464_c0_g2_i1 | black | -0.93271 |
| TRINITY_DN30262_c0_g2_i1 | black | -0.933689 |
| TRINITY_DN35448_c0_g1_i2 | black | -0.934428 |
| TRINITY_DN38457_c0_g3_i1 | black | -0.936773 |
| TRINITY_DN38246_c0_g5_i1 | black | -0.937508 |
| TRINITY_DN38770_c1_g3_i26 | black | -0.93831 |
| TRINITY_DN39111_c2_g1_i2 | black | -0.943557 |
| TRINITY_DN27540_c0_g1_i2 | black | -0.944644 |
| TRINITY_DN34595_c0_g2_i2 | black | -0.946409 |
| TRINITY_DN36114_c0_g3_i1 | black | -0.952424 |
| TRINITY_DN25516_c0_g1_i1 | black | -0.954423 |
| TRINITY_DN29153_c0_g1_i5 | black | -0.955582 |
| TRINITY_DN38748_c0_g4_i1 | black | -0.959776 |
| TRINITY_DN38685_c1_g3_i11 | black | -0.964814 |
| TRINITY_DN35625_c0_g1_i29 | black | -0.96568 |
| TRINITY_DN33085_c1_g1_i1 | blue | 0.9966179 |
| TRINITY_DN32404_c0_g3_i3 | blue | 0.994852 |
| TRINITY_DN32149_c2_g1_i1 | blue | 0.9939013 |
| TRINITY_DN36418_c0_g1_i6 | blue | 0.9935909 |
| TRINITY_DN35064_c1_g2_i3 | blue | 0.9934829 |
| TRINITY_DN32199_c0_g2_i1 | blue | 0.9924277 |
| TRINITY_DN38735_c1_g1_i1 | blue | 0.992376 |
| TRINITY_DN35825_c0_g2_i8 | blue | 0.99206 |
| TRINITY_DN31900_c0_g1_i6 | blue | 0.9916523 |
| TRINITY_DN32502_c0_g1_i3 | blue | 0.9915949 |
| TRINITY_DN38074_c0_g1_i6 | blue | 0.9914151 |
| TRINITY_DN25506_c0_g2_i2 | blue | 0.9909955 |
| TRINITY_DN35164_c0_g1_i6 | blue | 0.99084 |
| TRINITY_DN34373_c1_g2_i25 | blue | 0.9903264 |
| TRINITY_DN28683_c0_g1_i3 | blue | 0.9901885 |
| TRINITY_DN34248_c0_g5_i1 | blue | 0.9900747 |
| TRINITY_DN39068_c0_g3_i3 | blue | 0.9900658 |
| TRINITY_DN20223_c0_g1_i1 | blue | 0.9898311 |
| TRINITY_DN30931_c2_g2_i1 | blue | 0.9896457 |
| TRINITY_DN34210_c0_g2_i1 | blue | 0.9895113 |
| TRINITY_DN35898_c1_g7_i2 | blue | 0.9893572 |
| TRINITY_DN37321_c0_g1_i2 | blue | 0.9891491 |
| TRINITY_DN38532_c0_g4_i1 | blue | 0.9891278 |
| TRINITY_DN32702_c0_g1_i7 | blue | 0.9886104 |
| TRINITY_DN35886_c0_g3_i2 | blue | 0.9885691 |
| TRINITY_DN30442_c0_g4_i4 | blue | 0.9884371 |
| TRINITY_DN26082_c0_g2_i1 | blue | 0.9883556 |
| TRINITY_DN36558_c0_g1_i7 | blue | 0.9883346 |
| TRINITY_DN28567_c0_g1_i6 | blue | 0.9880171 |
| TRINITY_DN38609_c0_g1_i8 | blue | 0.9879225 |
| TRINITY_DN36790_c1_g6_i1 | blue | 0.9878983 |
| TRINITY_DN36519_c0_g2_i8 | blue | 0.9878082 |
| TRINITY_DN35898_c1_g13_i1 | blue | 0.9877661 |
| TRINITY_DN29659_c0_g1_i3 | blue | 0.9874622 |
| TRINITY_DN31995_c2_g1_i3 | blue | 0.9873941 |
| TRINITY_DN38058_c0_g1_i1 | blue | 0.9873932 |
| TRINITY_DN23939_c0_g1_i1 | blue | 0.9870666 |
| TRINITY_DN24315_c0_g1_i1 | blue | 0.9869472 |
| TRINITY_DN35773_c1_g2_i9 | blue | 0.9868185 |
| TRINITY_DN33169_c0_g1_i1 | blue | 0.9867336 |
| TRINITY_DN36907_c0_g1_i3 | blue | 0.9866737 |
| TRINITY_DN34866_c1_g2_i16 | blue | 0.9866667 |
| TRINITY_DN38885_c1_g2_i1 | blue | 0.9866392 |
| TRINITY_DN17242_c0_g1_i1 | blue | 0.9865292 |
| TRINITY_DN29101_c0_g2_i1 | blue | 0.9861204 |
| TRINITY_DN34045_c1_g3_i1 | blue | 0.9858737 |
| TRINITY_DN38340_c0_g3_i1 | blue | 0.98577 |
| TRINITY_DN27746_c0_g1_i1 | blue | 0.9857029 |
| TRINITY_DN28295_c0_g1_i4 | blue | 0.9856565 |
| TRINITY_DN33847_c1_g1_i3 | blue | 0.9856332 |
| TRINITY_DN34277_c0_g2_i1 | blue | 0.9856304 |
| TRINITY_DN28120_c0_g1_i2 | blue | 0.9855622 |
| TRINITY_DN32543_c0_g3_i4 | blue | 0.9855423 |
| TRINITY_DN32756_c0_g2_i1 | blue | 0.9855175 |
| TRINITY_DN32302_c1_g2_i1 | blue | 0.9854108 |
| TRINITY_DN33060_c0_g1_i1 | blue | 0.9853223 |
| TRINITY_DN29879_c0_g2_i1 | blue | 0.9853095 |
| TRINITY_DN28149_c0_g2_i3 | blue | 0.9851515 |
| TRINITY_DN37104_c0_g2_i1 | blue | 0.9851505 |
| TRINITY_DN31869_c0_g3_i1 | blue | 0.9850679 |
| TRINITY_DN28043_c0_g1_i1 | blue | 0.9848741 |
| TRINITY_DN35726_c1_g4_i6 | blue | 0.9848687 |
| TRINITY_DN38882_c2_g1_i3 | blue | 0.9848579 |
| TRINITY_DN34136_c0_g2_i3 | blue | 0.9848295 |
| TRINITY_DN31357_c0_g1_i3 | blue | 0.9848265 |
| TRINITY_DN31367_c0_g1_i9 | blue | 0.98481 |
| TRINITY_DN37364_c1_g2_i2 | blue | 0.9847432 |
| TRINITY_DN31936_c2_g4_i3 | blue | 0.984715 |
| TRINITY_DN31247_c0_g1_i3 | blue | 0.984643 |
| TRINITY_DN31481_c1_g2_i1 | blue | 0.9845441 |
| TRINITY_DN36737_c0_g2_i1 | blue | 0.9845178 |
| TRINITY_DN30958_c0_g1_i1 | blue | 0.9842676 |
| TRINITY_DN30763_c0_g1_i1 | blue | 0.9842379 |
| TRINITY_DN28763_c0_g2_i3 | blue | 0.9841732 |
| TRINITY_DN33925_c0_g2_i1 | blue | 0.9841603 |
| TRINITY_DN30885_c0_g1_i6 | blue | 0.9841075 |
| TRINITY_DN37380_c0_g3_i3 | blue | 0.984092 |
| TRINITY_DN32210_c2_g1_i2 | blue | 0.9840909 |
| TRINITY_DN36307_c0_g2_i2 | blue | 0.9840589 |
| TRINITY_DN34767_c1_g1_i7 | blue | 0.9840061 |
| TRINITY_DN27187_c0_g3_i1 | blue | 0.9839634 |
| TRINITY_DN36546_c0_g3_i14 | blue | 0.9839176 |
| TRINITY_DN39164_c0_g2_i14 | blue | 0.9837937 |
| TRINITY_DN31923_c0_g1_i8 | blue | 0.9837861 |
| TRINITY_DN29987_c0_g1_i1 | blue | 0.9837343 |
| TRINITY_DN36830_c0_g4_i6 | blue | 0.9836721 |
| TRINITY_DN38548_c1_g2_i2 | blue | 0.9836459 |
| TRINITY_DN37248_c0_g1_i1 | blue | 0.9835849 |
| TRINITY_DN35836_c1_g1_i2 | blue | 0.9833779 |
| TRINITY_DN30072_c0_g1_i3 | blue | 0.9831774 |
| TRINITY_DN39283_c3_g9_i3 | blue | 0.9831386 |
| TRINITY_DN38574_c0_g4_i3 | blue | 0.9831346 |
| TRINITY_DN38402_c1_g2_i6 | blue | 0.9831346 |
| TRINITY_DN38258_c0_g1_i1 | blue | 0.9831245 |
| TRINITY_DN39192_c3_g1_i1 | blue | 0.9829557 |
| TRINITY_DN30897_c0_g1_i2 | blue | 0.9829033 |
| TRINITY_DN39139_c3_g1_i1 | blue | 0.9828407 |
| TRINITY_DN38566_c1_g1_i5 | blue | 0.9827315 |
| TRINITY_DN37707_c0_g3_i2 | blue | 0.9825115 |
| TRINITY_DN20125_c0_g3_i1 | blue | 0.982475 |
| TRINITY_DN34553_c0_g3_i14 | blue | 0.9824401 |
| TRINITY_DN38819_c0_g3_i1 | blue | 0.9821843 |
| TRINITY_DN37289_c0_g1_i9 | blue | 0.9821753 |
| TRINITY_DN34242_c0_g1_i2 | blue | 0.9821276 |
| TRINITY_DN37779_c1_g1_i2 | blue | 0.981925 |
| TRINITY_DN36638_c0_g1_i8 | blue | 0.9818182 |
| TRINITY_DN32342_c1_g1_i2 | blue | 0.981793 |
| TRINITY_DN30114_c1_g1_i6 | blue | 0.9814418 |
| TRINITY_DN37852_c1_g3_i1 | blue | 0.9812808 |
| TRINITY_DN34966_c0_g2_i6 | blue | 0.9812209 |
| TRINITY_DN35872_c3_g2_i1 | blue | 0.981046 |
| TRINITY_DN35064_c1_g1_i8 | blue | 0.9810399 |
| TRINITY_DN33965_c0_g2_i7 | blue | 0.9810294 |
| TRINITY_DN36357_c0_g1_i1 | blue | 0.9810225 |
| TRINITY_DN34360_c0_g3_i1 | blue | 0.9809762 |
| TRINITY_DN34404_c0_g3_i3 | blue | 0.9808622 |
| TRINITY_DN36214_c0_g1_i6 | blue | 0.9807241 |
| TRINITY_DN38185_c2_g1_i17 | blue | 0.9806204 |
| TRINITY_DN51225_c0_g1_i1 | blue | 0.9803401 |
| TRINITY_DN27431_c0_g1_i1 | blue | 0.9802323 |
| TRINITY_DN29085_c0_g3_i1 | blue | 0.9798731 |
| TRINITY_DN22463_c0_g1_i2 | blue | 0.9798588 |
| TRINITY_DN33380_c0_g1_i3 | blue | 0.979858 |
| TRINITY_DN34224_c3_g2_i1 | blue | 0.9798231 |
| TRINITY_DN34302_c0_g1_i4 | blue | 0.9797783 |
| TRINITY_DN38779_c0_g2_i7 | blue | 0.9797108 |
| TRINITY_DN33635_c0_g1_i2 | blue | 0.9796764 |
| TRINITY_DN34960_c1_g1_i2 | blue | 0.9796527 |
| TRINITY_DN39138_c1_g8_i1 | blue | 0.9796047 |
| TRINITY_DN28197_c0_g2_i2 | blue | 0.9795424 |
| TRINITY_DN29962_c0_g1_i9 | blue | 0.9795321 |
| TRINITY_DN37842_c1_g1_i1 | blue | 0.9795114 |
| TRINITY_DN32509_c0_g1_i3 | blue | 0.9794786 |
| TRINITY_DN33134_c0_g1_i2 | blue | 0.9794473 |
| TRINITY_DN31845_c0_g1_i5 | blue | 0.9794118 |
| TRINITY_DN27637_c0_g5_i1 | blue | 0.9793751 |
| TRINITY_DN37996_c1_g1_i1 | blue | 0.9791726 |
| TRINITY_DN37012_c0_g7_i5 | blue | 0.979021 |
| TRINITY_DN34786_c1_g1_i3 | blue | 0.9790155 |
| TRINITY_DN31430_c0_g1_i3 | blue | 0.9790005 |
| TRINITY_DN37889_c0_g2_i4 | blue | 0.9789924 |
| TRINITY_DN32833_c2_g1_i1 | blue | 0.9789439 |
| TRINITY_DN29279_c0_g3_i5 | blue | 0.9788986 |
| TRINITY_DN30393_c0_g1_i2 | blue | 0.9787746 |
| TRINITY_DN39004_c1_g2_i10 | blue | 0.9786228 |
| TRINITY_DN34070_c0_g1_i12 | blue | 0.9784874 |
| TRINITY_DN35487_c0_g1_i3 | blue | 0.9784062 |
| TRINITY_DN35657_c0_g2_i3 | blue | 0.9783374 |
| TRINITY_DN27307_c0_g2_i1 | blue | 0.9782863 |
| TRINITY_DN33451_c1_g1_i10 | blue | 0.9782753 |
| TRINITY_DN37084_c0_g1_i1 | blue | 0.9781862 |
| TRINITY_DN37641_c0_g1_i2 | blue | 0.9780119 |
| TRINITY_DN33724_c0_g4_i1 | blue | 0.9779335 |
| TRINITY_DN32669_c0_g1_i1 | blue | 0.9779207 |
| TRINITY_DN29363_c0_g2_i2 | blue | 0.9778808 |
| TRINITY_DN23921_c0_g1_i2 | blue | 0.9775378 |
| TRINITY_DN37997_c2_g1_i1 | blue | 0.9775096 |
| TRINITY_DN50686_c0_g1_i1 | blue | 0.9774157 |
| TRINITY_DN38922_c1_g2_i7 | blue | 0.9774146 |
| TRINITY_DN26678_c0_g5_i1 | blue | 0.9772125 |
| TRINITY_DN32637_c0_g1_i1 | blue | 0.9771719 |
| TRINITY_DN36544_c1_g2_i8 | blue | 0.9771431 |
| TRINITY_DN30354_c0_g1_i1 | blue | 0.9771135 |
| TRINITY_DN32649_c0_g3_i8 | blue | 0.9770224 |
| TRINITY_DN31861_c0_g1_i4 | blue | 0.9768741 |
| TRINITY_DN38490_c0_g1_i1 | blue | 0.976874 |
| TRINITY_DN34084_c1_g1_i9 | blue | 0.9767943 |
| TRINITY_DN31688_c1_g1_i1 | blue | 0.9767432 |
| TRINITY_DN38189_c2_g4_i3 | blue | 0.9765565 |
| TRINITY_DN34770_c1_g2_i1 | blue | 0.9764459 |
| TRINITY_DN28743_c0_g1_i2 | blue | 0.9763914 |
| TRINITY_DN34536_c1_g2_i1 | blue | 0.9763773 |
| TRINITY_DN31607_c0_g5_i2 | blue | 0.9763473 |
| TRINITY_DN38364_c0_g6_i4 | blue | 0.9763359 |
| TRINITY_DN26984_c0_g1_i5 | blue | 0.9762653 |
| TRINITY_DN36626_c0_g1_i1 | blue | 0.9760842 |
| TRINITY_DN28965_c0_g2_i6 | blue | 0.9759496 |
| TRINITY_DN33730_c0_g1_i2 | blue | 0.9759476 |
| TRINITY_DN35852_c1_g2_i1 | blue | 0.975756 |
| TRINITY_DN35435_c0_g2_i1 | blue | 0.9756662 |
| TRINITY_DN38622_c0_g5_i6 | blue | 0.9756204 |
| TRINITY_DN38885_c1_g5_i11 | blue | 0.9755996 |
| TRINITY_DN37877_c0_g4_i7 | blue | 0.9755146 |
| TRINITY_DN36067_c0_g1_i1 | blue | 0.9755119 |
| TRINITY_DN37001_c0_g3_i6 | blue | 0.9752077 |
| TRINITY_DN36221_c0_g1_i2 | blue | 0.9750741 |
| TRINITY_DN33270_c0_g1_i3 | blue | 0.9748737 |
| TRINITY_DN34519_c0_g6_i3 | blue | 0.9748496 |
| TRINITY_DN30060_c0_g1_i1 | blue | 0.9748478 |
| TRINITY_DN29199_c1_g1_i5 | blue | 0.9748472 |
| TRINITY_DN32999_c1_g1_i4 | blue | 0.974767 |
| TRINITY_DN34344_c1_g6_i1 | blue | 0.9745924 |
| TRINITY_DN34491_c1_g1_i1 | blue | 0.9745166 |
| TRINITY_DN37274_c3_g11_i2 | blue | 0.9743706 |
| TRINITY_DN37342_c0_g1_i9 | blue | 0.9743396 |
| TRINITY_DN32290_c1_g3_i7 | blue | 0.9743237 |
| TRINITY_DN35933_c1_g1_i2 | blue | 0.9742769 |
| TRINITY_DN27742_c0_g1_i1 | blue | 0.9742576 |
| TRINITY_DN32119_c0_g1_i1 | blue | 0.9742566 |
| TRINITY_DN31279_c0_g1_i1 | blue | 0.9742106 |
| TRINITY_DN36932_c0_g5_i1 | blue | 0.9740925 |
| TRINITY_DN30827_c1_g1_i6 | blue | 0.9739137 |
| TRINITY_DN34687_c2_g1_i7 | blue | 0.9737545 |
| TRINITY_DN35603_c0_g2_i1 | blue | 0.9737213 |
| TRINITY_DN35541_c0_g1_i7 | blue | 0.9733975 |
| TRINITY_DN38258_c1_g1_i9 | blue | 0.9733695 |
| TRINITY_DN35176_c0_g1_i11 | blue | 0.9732224 |
| TRINITY_DN20723_c0_g1_i1 | blue | 0.9731475 |
| TRINITY_DN36622_c0_g2_i5 | blue | 0.9731093 |
| TRINITY_DN34181_c0_g2_i2 | blue | 0.9730755 |
| TRINITY_DN33617_c0_g1_i6 | blue | 0.9730492 |
| TRINITY_DN33305_c0_g2_i6 | blue | 0.9730189 |
| TRINITY_DN35154_c0_g3_i1 | blue | 0.9729926 |
| TRINITY_DN32779_c0_g1_i3 | blue | 0.9729848 |
| TRINITY_DN28878_c0_g1_i1 | blue | 0.9728967 |
| TRINITY_DN38680_c0_g3_i15 | blue | 0.97284 |
| TRINITY_DN36615_c0_g6_i9 | blue | 0.9727355 |
| TRINITY_DN31869_c0_g5_i3 | blue | 0.9727291 |
| TRINITY_DN28446_c0_g1_i3 | blue | 0.9727156 |
| TRINITY_DN36137_c0_g1_i1 | blue | 0.9726785 |
| TRINITY_DN36491_c0_g1_i1 | blue | 0.9726391 |
| TRINITY_DN35569_c1_g3_i14 | blue | 0.9726379 |
| TRINITY_DN24424_c0_g1_i1 | blue | 0.9725935 |
| TRINITY_DN37996_c1_g2_i1 | blue | 0.9725554 |
| TRINITY_DN19828_c0_g1_i1 | blue | 0.9725056 |
| TRINITY_DN36607_c0_g2_i2 | blue | 0.9724305 |
| TRINITY_DN36438_c0_g1_i1 | blue | 0.9723911 |
| TRINITY_DN36249_c0_g1_i12 | blue | 0.9723124 |
| TRINITY_DN27694_c0_g1_i9 | blue | 0.9722987 |
| TRINITY_DN34348_c0_g1_i3 | blue | 0.9721921 |
| TRINITY_DN33158_c1_g6_i1 | blue | 0.9721881 |
| TRINITY_DN37823_c1_g1_i2 | blue | 0.9721435 |
| TRINITY_DN27607_c0_g2_i1 | blue | 0.9721268 |
| TRINITY_DN37315_c1_g4_i1 | blue | 0.9721114 |
| TRINITY_DN29678_c0_g1_i3 | blue | 0.9720803 |
| TRINITY_DN31260_c1_g2_i5 | blue | 0.9720677 |
| TRINITY_DN35056_c2_g4_i1 | blue | 0.9720275 |
| TRINITY_DN36855_c0_g1_i4 | blue | 0.9720141 |
| TRINITY_DN21050_c0_g1_i2 | blue | 0.9717947 |
| TRINITY_DN35406_c4_g3_i5 | blue | 0.9717356 |
| TRINITY_DN27974_c0_g1_i2 | blue | 0.9717356 |
| TRINITY_DN38566_c0_g1_i2 | blue | 0.9716114 |
| TRINITY_DN29591_c0_g2_i1 | blue | 0.9716022 |
| TRINITY_DN38384_c0_g1_i5 | blue | 0.9715859 |
| TRINITY_DN33411_c0_g2_i6 | blue | 0.9715564 |
| TRINITY_DN27991_c0_g2_i1 | blue | 0.9715334 |
| TRINITY_DN34329_c0_g4_i5 | blue | 0.9715045 |
| TRINITY_DN33408_c0_g1_i6 | blue | 0.9714838 |
| TRINITY_DN37842_c1_g4_i2 | blue | 0.9713127 |
| TRINITY_DN33005_c0_g4_i12 | blue | 0.9712786 |
| TRINITY_DN33204_c1_g1_i12 | blue | 0.9712683 |
| TRINITY_DN31036_c0_g1_i4 | blue | 0.9711682 |
| TRINITY_DN33174_c0_g2_i5 | blue | 0.9710012 |
| TRINITY_DN34047_c0_g2_i8 | blue | 0.9709383 |
| TRINITY_DN30785_c0_g1_i2 | blue | 0.9708507 |
| TRINITY_DN33356_c0_g1_i14 | blue | 0.9708351 |
| TRINITY_DN38201_c0_g1_i4 | blue | 0.9708317 |
| TRINITY_DN35341_c1_g2_i5 | blue | 0.9707931 |
| TRINITY_DN28477_c0_g1_i1 | blue | 0.9706883 |
| TRINITY_DN20938_c0_g1_i1 | blue | 0.9704723 |
| TRINITY_DN23882_c1_g1_i1 | blue | 0.9704603 |
| TRINITY_DN35534_c0_g1_i5 | blue | 0.9704443 |
| TRINITY_DN16248_c0_g1_i1 | blue | 0.9703724 |
| TRINITY_DN33213_c0_g2_i2 | blue | 0.9703609 |
| TRINITY_DN28432_c0_g1_i1 | blue | 0.9703371 |
| TRINITY_DN31858_c0_g1_i1 | blue | 0.9703111 |
| TRINITY_DN26104_c0_g1_i1 | blue | 0.9701349 |
| TRINITY_DN36486_c0_g1_i1 | blue | 0.9701141 |
| TRINITY_DN38761_c2_g1_i3 | blue | 0.9701096 |
| TRINITY_DN33376_c0_g1_i2 | blue | 0.9697905 |
| TRINITY_DN36646_c1_g1_i5 | blue | 0.9697495 |
| TRINITY_DN32035_c0_g1_i2 | blue | 0.969625 |
| TRINITY_DN34135_c0_g1_i2 | blue | 0.9696121 |
| TRINITY_DN26276_c0_g3_i1 | blue | 0.9695354 |
| TRINITY_DN19398_c0_g1_i2 | blue | 0.9694987 |
| TRINITY_DN31580_c2_g11_i1 | blue | 0.9694953 |
| TRINITY_DN30158_c0_g1_i2 | blue | 0.9694321 |
| TRINITY_DN30031_c0_g1_i1 | blue | 0.9693572 |
| TRINITY_DN33042_c1_g7_i1 | blue | 0.9693326 |
| TRINITY_DN26847_c0_g1_i3 | blue | 0.9692451 |
| TRINITY_DN35050_c0_g1_i2 | blue | 0.9692049 |
| TRINITY_DN37915_c0_g4_i8 | blue | 0.9691819 |
| TRINITY_DN22829_c0_g1_i1 | blue | 0.9691308 |
| TRINITY_DN38395_c3_g1_i11 | blue | 0.9690341 |
| TRINITY_DN30768_c0_g1_i7 | blue | 0.9690203 |
| TRINITY_DN29845_c0_g1_i9 | blue | 0.9690039 |
| TRINITY_DN33810_c2_g2_i6 | blue | 0.9688959 |
| TRINITY_DN27205_c0_g2_i1 | blue | 0.9688649 |
| TRINITY_DN32761_c0_g1_i2 | blue | 0.9687703 |
| TRINITY_DN34118_c0_g1_i8 | blue | 0.9685961 |
| TRINITY_DN38731_c3_g1_i1 | blue | 0.9685437 |
| TRINITY_DN36179_c1_g1_i3 | blue | 0.9685042 |
| TRINITY_DN29306_c0_g3_i3 | blue | 0.9684102 |
| TRINITY_DN30475_c0_g1_i5 | blue | 0.9683972 |
| TRINITY_DN29932_c0_g1_i1 | blue | 0.9683861 |
| TRINITY_DN38249_c2_g4_i1 | blue | 0.9682607 |
| TRINITY_DN38955_c1_g1_i2 | blue | 0.9682393 |
| TRINITY_DN34911_c0_g3_i2 | blue | 0.9680897 |
| TRINITY_DN38189_c2_g3_i5 | blue | 0.9680865 |
| TRINITY_DN35940_c1_g1_i7 | blue | 0.968078 |
| TRINITY_DN27885_c0_g1_i1 | blue | 0.9680286 |
| TRINITY_DN34973_c0_g2_i3 | blue | 0.9679323 |
| TRINITY_DN37032_c0_g3_i3 | blue | 0.9679314 |
| TRINITY_DN30022_c0_g1_i8 | blue | 0.9678562 |
| TRINITY_DN33555_c0_g1_i2 | blue | 0.9678249 |
| TRINITY_DN34658_c1_g1_i12 | blue | 0.9678074 |
| TRINITY_DN37300_c1_g1_i8 | blue | 0.9676449 |
| TRINITY_DN34590_c1_g1_i4 | blue | 0.9676407 |
| TRINITY_DN25758_c0_g1_i1 | blue | 0.9673502 |
| TRINITY_DN25585_c0_g1_i2 | blue | 0.9672536 |
| TRINITY_DN33863_c0_g1_i4 | blue | 0.9670908 |
| TRINITY_DN37755_c0_g1_i3 | blue | 0.9669867 |
| TRINITY_DN25789_c0_g1_i2 | blue | 0.9669642 |
| TRINITY_DN37344_c1_g1_i4 | blue | 0.9667669 |
| TRINITY_DN29130_c0_g1_i1 | blue | 0.9667585 |
| TRINITY_DN38515_c0_g1_i4 | blue | 0.9667565 |
| TRINITY_DN23779_c0_g1_i1 | blue | 0.966561 |
| TRINITY_DN37220_c0_g1_i1 | blue | 0.9665557 |
| TRINITY_DN35518_c0_g1_i13 | blue | 0.9665467 |
| TRINITY_DN35675_c0_g1_i3 | blue | 0.9663973 |
| TRINITY_DN30824_c0_g1_i2 | blue | 0.9663854 |
| TRINITY_DN33832_c0_g2_i7 | blue | 0.9662774 |
| TRINITY_DN36328_c0_g1_i2 | blue | 0.9661769 |
| TRINITY_DN34796_c1_g1_i1 | blue | 0.9661341 |
| TRINITY_DN33266_c0_g1_i1 | blue | 0.9661299 |
| TRINITY_DN36065_c0_g1_i11 | blue | 0.9660458 |
| TRINITY_DN26836_c0_g1_i1 | blue | 0.9660427 |
| TRINITY_DN39021_c0_g1_i1 | blue | 0.9659184 |
| TRINITY_DN31557_c0_g1_i1 | blue | 0.965891 |
| TRINITY_DN26833_c0_g2_i1 | blue | 0.9658868 |
| TRINITY_DN36943_c1_g1_i2 | blue | 0.9658574 |
| TRINITY_DN38567_c0_g6_i1 | blue | 0.9658426 |
| TRINITY_DN30485_c0_g2_i3 | blue | 0.9657979 |
| TRINITY_DN34725_c0_g1_i1 | blue | 0.9656394 |
| TRINITY_DN27853_c0_g2_i1 | blue | 0.9656123 |
| TRINITY_DN35844_c0_g3_i1 | blue | 0.9652252 |
| TRINITY_DN30944_c1_g3_i2 | blue | 0.96522 |
| TRINITY_DN25973_c0_g1_i2 | blue | 0.9652095 |
| TRINITY_DN33497_c0_g1_i2 | blue | 0.9651397 |
| TRINITY_DN37240_c0_g1_i8 | blue | 0.9649708 |
| TRINITY_DN35528_c0_g2_i1 | blue | 0.9649582 |
| TRINITY_DN36319_c0_g3_i4 | blue | 0.9649161 |
| TRINITY_DN30525_c0_g1_i2 | blue | 0.964876 |
| TRINITY_DN34179_c0_g1_i2 | blue | 0.9648297 |
| TRINITY_DN59556_c0_g1_i1 | blue | 0.9647953 |
| TRINITY_DN32098_c0_g3_i3 | blue | 0.9647403 |
| TRINITY_DN29064_c0_g1_i2 | blue | 0.9647266 |
| TRINITY_DN36910_c0_g4_i1 | blue | 0.9647056 |
| TRINITY_DN31494_c0_g1_i1 | blue | 0.9646642 |
| TRINITY_DN28718_c0_g1_i2 | blue | 0.9645994 |
| TRINITY_DN34957_c0_g2_i3 | blue | 0.9645392 |
| TRINITY_DN37834_c0_g1_i15 | blue | 0.9645274 |
| TRINITY_DN34759_c0_g1_i10 | blue | 0.9644358 |
| TRINITY_DN38018_c0_g1_i1 | blue | 0.9643903 |
| TRINITY_DN39062_c3_g4_i3 | blue | 0.964349 |
| TRINITY_DN35116_c1_g1_i5 | blue | 0.9642318 |
| TRINITY_DN33173_c0_g1_i1 | blue | 0.9639534 |
| TRINITY_DN28908_c0_g3_i1 | blue | 0.9639472 |
| TRINITY_DN38249_c0_g2_i4 | blue | 0.963927 |
| TRINITY_DN30282_c0_g1_i5 | blue | 0.9639082 |
| TRINITY_DN38484_c0_g4_i3 | blue | 0.963693 |
| TRINITY_DN33574_c0_g1_i2 | blue | 0.9636808 |
| TRINITY_DN33518_c1_g1_i4 | blue | 0.963527 |
| TRINITY_DN39180_c8_g2_i7 | blue | 0.96329 |
| TRINITY_DN30444_c0_g1_i1 | blue | 0.9632747 |
| TRINITY_DN32759_c0_g1_i2 | blue | 0.9632064 |
| TRINITY_DN34915_c0_g1_i4 | blue | 0.9630683 |
| TRINITY_DN31841_c0_g1_i8 | blue | 0.9629463 |
| TRINITY_DN34420_c1_g3_i4 | blue | 0.9628752 |
| TRINITY_DN33738_c0_g4_i4 | blue | 0.9627323 |
| TRINITY_DN35047_c0_g1_i1 | blue | 0.9626901 |
| TRINITY_DN27278_c0_g1_i1 | blue | 0.962604 |
| TRINITY_DN35250_c0_g1_i5 | blue | 0.9625877 |
| TRINITY_DN33667_c0_g1_i3 | blue | 0.9625494 |
| TRINITY_DN35373_c1_g12_i3 | blue | 0.9625108 |
| TRINITY_DN35256_c0_g2_i8 | blue | 0.9624734 |
| TRINITY_DN27550_c0_g1_i1 | blue | 0.9624412 |
| TRINITY_DN26692_c0_g1_i2 | blue | 0.9623589 |
| TRINITY_DN34869_c1_g1_i2 | blue | 0.9623474 |
| TRINITY_DN33829_c0_g1_i1 | blue | 0.9623316 |
| TRINITY_DN33424_c0_g2_i7 | blue | 0.9622203 |
| TRINITY_DN36079_c0_g2_i2 | blue | 0.9621362 |
| TRINITY_DN35218_c1_g1_i3 | blue | 0.9621002 |
| TRINITY_DN37618_c0_g4_i2 | blue | 0.9619712 |
| TRINITY_DN30303_c1_g2_i1 | blue | 0.9618661 |
| TRINITY_DN35807_c0_g1_i4 | blue | 0.9617921 |
| TRINITY_DN36542_c0_g2_i1 | blue | 0.9615298 |
| TRINITY_DN29211_c0_g1_i2 | blue | 0.9615025 |
| TRINITY_DN28311_c0_g3_i1 | blue | 0.9614297 |
| TRINITY_DN36897_c0_g1_i1 | blue | 0.9612833 |
| TRINITY_DN34950_c0_g2_i3 | blue | 0.9610415 |
| TRINITY_DN32249_c0_g1_i3 | blue | 0.9610205 |
| TRINITY_DN32841_c2_g1_i5 | blue | 0.9608132 |
| TRINITY_DN38324_c0_g1_i1 | blue | 0.9605002 |
| TRINITY_DN35030_c0_g3_i2 | blue | 0.9603826 |
| TRINITY_DN33449_c0_g1_i13 | blue | 0.960365 |
| TRINITY_DN15737_c0_g2_i1 | blue | 0.9602588 |
| TRINITY_DN34756_c0_g2_i13 | blue | 0.9602404 |
| TRINITY_DN38935_c0_g3_i20 | blue | 0.9601847 |
| TRINITY_DN38429_c10_g1_i1 | blue | 0.9600502 |
| TRINITY_DN36359_c3_g3_i1 | blue | 0.9600079 |
| TRINITY_DN23928_c0_g1_i2 | blue | 0.9600018 |
| TRINITY_DN35744_c0_g1_i1 | blue | 0.959952 |
| TRINITY_DN35084_c0_g1_i4 | blue | 0.9597948 |
| TRINITY_DN32988_c0_g1_i1 | blue | 0.9597786 |
| TRINITY_DN27289_c2_g10_i1 | blue | 0.9595806 |
| TRINITY_DN28997_c0_g1_i6 | blue | 0.9595232 |
| TRINITY_DN32547_c1_g1_i1 | blue | 0.9595104 |
| TRINITY_DN35572_c0_g8_i2 | blue | 0.9592592 |
| TRINITY_DN33886_c0_g1_i3 | blue | 0.9592352 |
| TRINITY_DN21668_c0_g1_i1 | blue | 0.9592301 |
| TRINITY_DN37542_c0_g6_i4 | blue | 0.9592137 |
| TRINITY_DN30709_c0_g1_i1 | blue | 0.9591999 |
| TRINITY_DN30289_c0_g1_i6 | blue | 0.9591775 |
| TRINITY_DN30417_c0_g1_i1 | blue | 0.9591508 |
| TRINITY_DN28769_c0_g1_i2 | blue | 0.9590716 |
| TRINITY_DN36482_c1_g3_i5 | blue | 0.9589006 |
| TRINITY_DN36616_c2_g4_i5 | blue | 0.9588072 |
| TRINITY_DN38536_c0_g6_i2 | blue | 0.9587314 |
| TRINITY_DN34884_c1_g1_i1 | blue | 0.9586596 |
| TRINITY_DN30946_c0_g1_i8 | blue | 0.9585139 |
| TRINITY_DN34226_c0_g1_i4 | blue | 0.9584895 |
| TRINITY_DN29055_c0_g1_i2 | blue | 0.9584656 |
| TRINITY_DN37019_c1_g2_i5 | blue | 0.9584631 |
| TRINITY_DN25899_c0_g1_i5 | blue | 0.9584603 |
| TRINITY_DN27609_c0_g2_i2 | blue | 0.95846 |
| TRINITY_DN37459_c0_g4_i2 | blue | 0.9584501 |
| TRINITY_DN37420_c3_g1_i6 | blue | 0.9583831 |
| TRINITY_DN35268_c0_g2_i3 | blue | 0.9583201 |
| TRINITY_DN33534_c0_g1_i6 | blue | 0.9583053 |
| TRINITY_DN31146_c0_g1_i1 | blue | 0.9582733 |
| TRINITY_DN19953_c0_g1_i1 | blue | 0.9581344 |
| TRINITY_DN30399_c0_g1_i2 | blue | 0.9580533 |
| TRINITY_DN7987_c0_g1_i1 | blue | 0.958052 |
| TRINITY_DN28993_c0_g1_i3 | blue | 0.958012 |
| TRINITY_DN31526_c0_g1_i1 | blue | 0.9579829 |
| TRINITY_DN35592_c0_g1_i1 | blue | 0.9579549 |
| TRINITY_DN31911_c2_g2_i1 | blue | 0.9578737 |
| TRINITY_DN32282_c0_g2_i1 | blue | 0.9578732 |
| TRINITY_DN36762_c0_g2_i5 | blue | 0.9577855 |
| TRINITY_DN27988_c0_g1_i1 | blue | 0.9577275 |
| TRINITY_DN28103_c0_g1_i1 | blue | 0.9577133 |
| TRINITY_DN37972_c0_g1_i9 | blue | 0.9577108 |
| TRINITY_DN38616_c2_g9_i1 | blue | 0.9577033 |
| TRINITY_DN36626_c0_g2_i3 | blue | 0.9576834 |
| TRINITY_DN36099_c3_g2_i6 | blue | 0.9576809 |
| TRINITY_DN30865_c0_g1_i1 | blue | 0.9576021 |
| TRINITY_DN35013_c0_g2_i1 | blue | 0.9574857 |
| TRINITY_DN37308_c1_g3_i4 | blue | 0.9574384 |
| TRINITY_DN33904_c0_g1_i1 | blue | 0.9574281 |
| TRINITY_DN32404_c0_g1_i1 | blue | 0.9573134 |
| TRINITY_DN38793_c0_g3_i5 | blue | 0.9572682 |
| TRINITY_DN30446_c0_g1_i1 | blue | 0.9572295 |
| TRINITY_DN31834_c0_g1_i5 | blue | 0.9571604 |
| TRINITY_DN22433_c0_g2_i1 | blue | 0.9571539 |
| TRINITY_DN36950_c0_g2_i3 | blue | 0.9571286 |
| TRINITY_DN28793_c0_g1_i1 | blue | 0.9570382 |
| TRINITY_DN33354_c0_g1_i12 | blue | 0.9568623 |
| TRINITY_DN25764_c0_g1_i2 | blue | 0.9567726 |
| TRINITY_DN30121_c0_g1_i1 | blue | 0.9566562 |
| TRINITY_DN38985_c1_g12_i1 | blue | 0.9565839 |
| TRINITY_DN30594_c1_g1_i4 | blue | 0.9565838 |
| TRINITY_DN36822_c1_g1_i5 | blue | 0.9564612 |
| TRINITY_DN38083_c1_g2_i22 | blue | 0.9564339 |
| TRINITY_DN22988_c0_g2_i1 | blue | 0.9564247 |
| TRINITY_DN35841_c1_g2_i1 | blue | 0.9563356 |
| TRINITY_DN37892_c0_g2_i16 | blue | 0.9563084 |
| TRINITY_DN39307_c2_g2_i2 | blue | 0.9562991 |
| TRINITY_DN33217_c1_g3_i3 | blue | 0.956206 |
| TRINITY_DN37398_c3_g8_i2 | blue | 0.9561354 |
| TRINITY_DN33583_c0_g6_i1 | blue | 0.9560741 |
| TRINITY_DN26522_c0_g1_i1 | blue | 0.956055 |
| TRINITY_DN31300_c0_g4_i1 | blue | 0.9557199 |
| TRINITY_DN36525_c0_g2_i1 | blue | 0.9557016 |
| TRINITY_DN34336_c0_g2_i16 | blue | 0.9556784 |
| TRINITY_DN34366_c0_g1_i8 | blue | 0.9556092 |
| TRINITY_DN31465_c0_g1_i2 | blue | 0.9555361 |
| TRINITY_DN31359_c0_g1_i1 | blue | 0.9555329 |
| TRINITY_DN35041_c0_g1_i1 | blue | 0.9555169 |
| TRINITY_DN35852_c1_g1_i2 | blue | 0.9554104 |
| TRINITY_DN30293_c0_g2_i2 | blue | 0.9553155 |
| TRINITY_DN31300_c0_g5_i1 | blue | 0.9552458 |
| TRINITY_DN30881_c0_g1_i1 | blue | 0.9552263 |
| TRINITY_DN34962_c0_g3_i1 | blue | 0.9552235 |
| TRINITY_DN30585_c0_g1_i3 | blue | 0.9551839 |
| TRINITY_DN39107_c2_g1_i2 | blue | 0.9551794 |
| TRINITY_DN35696_c0_g1_i3 | blue | 0.9551451 |
| TRINITY_DN29546_c0_g1_i3 | blue | 0.9551065 |
| TRINITY_DN26795_c0_g1_i1 | blue | 0.9551043 |
| TRINITY_DN29611_c0_g1_i1 | blue | 0.9550748 |
| TRINITY_DN31408_c0_g1_i1 | blue | 0.9550471 |
| TRINITY_DN31250_c0_g3_i4 | blue | 0.954981 |
| TRINITY_DN33448_c0_g1_i2 | blue | 0.9549428 |
| TRINITY_DN31580_c2_g9_i1 | blue | 0.9548752 |
| TRINITY_DN31796_c0_g1_i5 | blue | 0.9548525 |
| TRINITY_DN35270_c0_g2_i2 | blue | 0.9548404 |
| TRINITY_DN32041_c0_g1_i6 | blue | 0.9548292 |
| TRINITY_DN31971_c0_g1_i4 | blue | 0.9547706 |
| TRINITY_DN24402_c0_g1_i1 | blue | 0.9546467 |
| TRINITY_DN34758_c0_g7_i4 | blue | 0.9546435 |
| TRINITY_DN34077_c1_g4_i5 | blue | 0.9546268 |
| TRINITY_DN38327_c1_g1_i7 | blue | 0.9546002 |
| TRINITY_DN33597_c1_g1_i4 | blue | 0.9544723 |
| TRINITY_DN32136_c0_g2_i5 | blue | 0.954374 |
| TRINITY_DN35560_c1_g1_i1 | blue | 0.9543433 |
| TRINITY_DN34638_c0_g2_i5 | blue | 0.9543392 |
| TRINITY_DN38380_c1_g3_i12 | blue | 0.954335 |
| TRINITY_DN28071_c0_g1_i3 | blue | 0.9543225 |
| TRINITY_DN37211_c1_g2_i1 | blue | 0.9543 |
| TRINITY_DN37862_c2_g1_i2 | blue | 0.9542874 |
| TRINITY_DN35612_c0_g1_i13 | blue | 0.9542831 |
| TRINITY_DN34371_c3_g2_i2 | blue | 0.954187 |
| TRINITY_DN36757_c0_g2_i2 | blue | 0.9541347 |
| TRINITY_DN33949_c0_g1_i1 | blue | 0.9540216 |
| TRINITY_DN34843_c1_g8_i7 | blue | 0.9539541 |
| TRINITY_DN35389_c0_g2_i1 | blue | 0.9539252 |
| TRINITY_DN27762_c0_g1_i1 | blue | 0.9538806 |
| TRINITY_DN36039_c1_g8_i1 | blue | 0.9538442 |
| TRINITY_DN33824_c1_g1_i1 | blue | 0.9536949 |
| TRINITY_DN26063_c0_g1_i1 | blue | 0.9536437 |
| TRINITY_DN34403_c0_g1_i1 | blue | 0.9535819 |
| TRINITY_DN35508_c0_g1_i6 | blue | 0.9535734 |
| TRINITY_DN38827_c1_g1_i4 | blue | 0.9534713 |
| TRINITY_DN25521_c0_g1_i1 | blue | 0.9533696 |
| TRINITY_DN32188_c0_g1_i1 | blue | 0.953295 |
| TRINITY_DN20307_c0_g1_i3 | blue | 0.9531969 |
| TRINITY_DN30943_c0_g1_i1 | blue | 0.9531411 |
| TRINITY_DN37329_c0_g1_i1 | blue | 0.9530225 |
| TRINITY_DN27137_c0_g1_i2 | blue | 0.9529938 |
| TRINITY_DN31891_c1_g1_i12 | blue | 0.95296 |
| TRINITY_DN31708_c0_g1_i1 | blue | 0.9528782 |
| TRINITY_DN25037_c0_g1_i2 | blue | 0.9528465 |
| TRINITY_DN35203_c0_g4_i1 | blue | 0.9527404 |
| TRINITY_DN31811_c0_g1_i1 | blue | 0.9527365 |
| TRINITY_DN21661_c0_g1_i1 | blue | 0.9526944 |
| TRINITY_DN38058_c0_g2_i1 | blue | 0.9526941 |
| TRINITY_DN27241_c0_g1_i1 | blue | 0.9526077 |
| TRINITY_DN27638_c0_g1_i1 | blue | 0.9525172 |
| TRINITY_DN29896_c0_g1_i1 | blue | 0.9525065 |
| TRINITY_DN33336_c1_g2_i2 | blue | 0.952354 |
| TRINITY_DN37002_c1_g2_i4 | blue | 0.9523455 |
| TRINITY_DN32386_c0_g1_i8 | blue | 0.9522672 |
| TRINITY_DN29568_c0_g3_i3 | blue | 0.9520972 |
| TRINITY_DN29838_c0_g1_i5 | blue | 0.9520312 |
| TRINITY_DN15976_c0_g2_i1 | blue | 0.9519745 |
| TRINITY_DN35922_c0_g7_i6 | blue | 0.9519267 |
| TRINITY_DN27586_c0_g1_i1 | blue | 0.9518642 |
| TRINITY_DN37122_c0_g2_i7 | blue | 0.9518456 |
| TRINITY_DN28228_c0_g1_i2 | blue | 0.9517125 |
| TRINITY_DN38572_c0_g11_i3 | blue | 0.9516709 |
| TRINITY_DN35486_c0_g3_i9 | blue | 0.9516356 |
| TRINITY_DN27773_c0_g2_i3 | blue | 0.9515082 |
| TRINITY_DN35469_c0_g1_i4 | blue | 0.9514864 |
| TRINITY_DN33464_c0_g2_i2 | blue | 0.9514387 |
| TRINITY_DN37648_c0_g8_i9 | blue | 0.9513471 |
| TRINITY_DN36116_c0_g1_i2 | blue | 0.9513304 |
| TRINITY_DN35841_c1_g1_i15 | blue | 0.951099 |
| TRINITY_DN32146_c1_g1_i1 | blue | 0.9510969 |
| TRINITY_DN34169_c2_g1_i1 | blue | 0.9510755 |
| TRINITY_DN33112_c2_g1_i7 | blue | 0.9508269 |
| TRINITY_DN29687_c0_g1_i4 | blue | 0.9508149 |
| TRINITY_DN30442_c0_g2_i1 | blue | 0.9507217 |
| TRINITY_DN32491_c0_g2_i2 | blue | 0.9507091 |
| TRINITY_DN30147_c0_g2_i4 | blue | 0.9506383 |
| TRINITY_DN34604_c1_g1_i3 | blue | 0.9505898 |
| TRINITY_DN39220_c6_g2_i1 | blue | 0.9505861 |
| TRINITY_DN31740_c0_g1_i3 | blue | 0.9505145 |
| TRINITY_DN28537_c0_g1_i8 | blue | 0.9502079 |
| TRINITY_DN33992_c1_g3_i2 | blue | 0.9500213 |
| TRINITY_DN28077_c0_g4_i1 | blue | 0.9499415 |
| TRINITY_DN38612_c1_g1_i7 | blue | 0.9499262 |
| TRINITY_DN32874_c0_g1_i1 | blue | 0.9498574 |
| TRINITY_DN30735_c0_g1_i1 | blue | 0.9498187 |
| TRINITY_DN29427_c0_g1_i5 | blue | 0.949784 |
| TRINITY_DN27904_c0_g1_i1 | blue | 0.9497388 |
| TRINITY_DN30801_c1_g2_i2 | blue | 0.9495342 |
| TRINITY_DN32245_c0_g2_i2 | blue | 0.9494684 |
| TRINITY_DN29530_c2_g3_i1 | blue | 0.9494579 |
| TRINITY_DN36169_c0_g1_i3 | blue | 0.9494523 |
| TRINITY_DN23033_c0_g2_i1 | blue | 0.9493706 |
| TRINITY_DN37010_c0_g1_i1 | blue | 0.9493051 |
| TRINITY_DN32142_c0_g1_i7 | blue | 0.9492864 |
| TRINITY_DN29692_c0_g1_i1 | blue | 0.9491596 |
| TRINITY_DN72636_c0_g1_i1 | blue | 0.949133 |
| TRINITY_DN34678_c2_g1_i5 | blue | 0.9490682 |
| TRINITY_DN35132_c0_g1_i2 | blue | 0.9490258 |
| TRINITY_DN26626_c0_g1_i1 | blue | 0.9489549 |
| TRINITY_DN26341_c0_g1_i1 | blue | 0.948811 |
| TRINITY_DN28411_c0_g1_i1 | blue | 0.9487884 |
| TRINITY_DN29873_c1_g1_i4 | blue | 0.9487731 |
| TRINITY_DN35406_c5_g1_i2 | blue | 0.9487581 |
| TRINITY_DN36493_c0_g5_i3 | blue | 0.948751 |
| TRINITY_DN33612_c0_g1_i14 | blue | 0.9487391 |
| TRINITY_DN27717_c0_g1_i1 | blue | 0.9486749 |
| TRINITY_DN23377_c0_g1_i3 | blue | 0.9486592 |
| TRINITY_DN38147_c0_g2_i6 | blue | 0.9486387 |
| TRINITY_DN34059_c0_g2_i12 | blue | 0.9486018 |
| TRINITY_DN36627_c0_g1_i4 | blue | 0.9485947 |
| TRINITY_DN34770_c1_g1_i2 | blue | 0.9485661 |
| TRINITY_DN31965_c1_g1_i4 | blue | 0.9485407 |
| TRINITY_DN39329_c13_g4_i1 | blue | 0.9484564 |
| TRINITY_DN34660_c0_g1_i1 | blue | 0.9484477 |
| TRINITY_DN29257_c1_g1_i1 | blue | 0.9484354 |
| TRINITY_DN33565_c0_g2_i1 | blue | 0.9484125 |
| TRINITY_DN32713_c0_g1_i1 | blue | 0.9483574 |
| TRINITY_DN34133_c0_g2_i5 | blue | 0.9483544 |
| TRINITY_DN31023_c0_g1_i1 | blue | 0.948352 |
| TRINITY_DN28831_c0_g1_i1 | blue | 0.9482727 |
| TRINITY_DN39265_c2_g1_i5 | blue | 0.9481868 |
| TRINITY_DN31481_c0_g2_i2 | blue | 0.9481595 |
| TRINITY_DN37825_c0_g1_i3 | blue | 0.9481211 |
| TRINITY_DN26972_c0_g1_i1 | blue | 0.9480458 |
| TRINITY_DN27004_c0_g1_i3 | blue | 0.9480201 |
| TRINITY_DN39180_c6_g1_i1 | blue | 0.9479406 |
| TRINITY_DN38121_c2_g1_i11 | blue | 0.9479371 |
| TRINITY_DN35552_c0_g2_i1 | blue | 0.9478468 |
| TRINITY_DN28398_c0_g1_i6 | blue | 0.9478227 |
| TRINITY_DN36607_c0_g6_i2 | blue | 0.9477378 |
| TRINITY_DN34150_c1_g1_i5 | blue | 0.9477308 |
| TRINITY_DN30700_c0_g2_i2 | blue | 0.9476766 |
| TRINITY_DN36039_c1_g5_i1 | blue | 0.9476135 |
| TRINITY_DN32468_c0_g1_i2 | blue | 0.9473452 |
| TRINITY_DN37473_c0_g2_i2 | blue | 0.9473382 |
| TRINITY_DN35004_c0_g2_i2 | blue | 0.9472168 |
| TRINITY_DN38267_c1_g1_i2 | blue | 0.9471128 |
| TRINITY_DN31228_c0_g1_i2 | blue | 0.9470796 |
| TRINITY_DN27827_c0_g1_i2 | blue | 0.9470514 |
| TRINITY_DN28399_c1_g1_i1 | blue | 0.9470374 |
| TRINITY_DN25576_c0_g1_i1 | blue | 0.9469833 |
| TRINITY_DN37025_c0_g1_i8 | blue | 0.9469748 |
| TRINITY_DN36952_c0_g8_i5 | blue | 0.9469385 |
| TRINITY_DN38985_c1_g4_i1 | blue | 0.9468651 |
| TRINITY_DN32359_c0_g1_i1 | blue | 0.9468256 |
| TRINITY_DN33840_c0_g4_i6 | blue | 0.9467994 |
| TRINITY_DN30430_c0_g1_i7 | blue | 0.9467538 |
| TRINITY_DN27217_c0_g3_i2 | blue | 0.9467209 |
| TRINITY_DN37890_c0_g1_i1 | blue | 0.9466678 |
| TRINITY_DN36661_c1_g4_i1 | blue | 0.9466546 |
| TRINITY_DN25230_c1_g1_i1 | blue | 0.9465727 |
| TRINITY_DN39089_c1_g2_i2 | blue | 0.946542 |
| TRINITY_DN26907_c0_g2_i8 | blue | 0.9464999 |
| TRINITY_DN25690_c0_g1_i1 | blue | 0.9463074 |
| TRINITY_DN33330_c0_g2_i1 | blue | 0.9462283 |
| TRINITY_DN26541_c0_g1_i3 | blue | 0.9462066 |
| TRINITY_DN32901_c0_g3_i7 | blue | 0.9461745 |
| TRINITY_DN38336_c1_g3_i8 | blue | 0.9461083 |
| TRINITY_DN35773_c0_g1_i1 | blue | 0.9459461 |
| TRINITY_DN28528_c0_g1_i1 | blue | 0.9459123 |
| TRINITY_DN29644_c0_g1_i1 | blue | 0.9458664 |
| TRINITY_DN38520_c2_g1_i8 | blue | 0.9458255 |
| TRINITY_DN35134_c0_g5_i3 | blue | 0.9457265 |
| TRINITY_DN32580_c0_g2_i1 | blue | 0.9454897 |
| TRINITY_DN27410_c0_g1_i3 | blue | 0.9454435 |
| TRINITY_DN38540_c1_g1_i8 | blue | 0.9452627 |
| TRINITY_DN29500_c0_g1_i1 | blue | 0.9452084 |
| TRINITY_DN32531_c0_g1_i1 | blue | 0.9451815 |
| TRINITY_DN33725_c0_g1_i9 | blue | 0.9451459 |
| TRINITY_DN28932_c0_g1_i1 | blue | 0.9450759 |
| TRINITY_DN28986_c0_g1_i4 | blue | 0.9450504 |
| TRINITY_DN18388_c0_g1_i1 | blue | 0.9449559 |
| TRINITY_DN35730_c0_g1_i1 | blue | 0.9448675 |
| TRINITY_DN16642_c0_g1_i1 | blue | 0.9447457 |
| TRINITY_DN37266_c0_g2_i13 | blue | 0.9446394 |
| TRINITY_DN39329_c12_g3_i1 | blue | 0.9445875 |
| TRINITY_DN30621_c0_g1_i1 | blue | 0.9445523 |
| TRINITY_DN36734_c0_g1_i4 | blue | 0.9443988 |
| TRINITY_DN38922_c1_g6_i1 | blue | 0.9443924 |
| TRINITY_DN23799_c0_g2_i1 | blue | 0.9442715 |
| TRINITY_DN34817_c0_g2_i1 | blue | 0.9441966 |
| TRINITY_DN33112_c1_g2_i2 | blue | 0.9441871 |
| TRINITY_DN23735_c0_g4_i1 | blue | 0.9441854 |
| TRINITY_DN38699_c0_g4_i1 | blue | 0.9441741 |
| TRINITY_DN31223_c0_g1_i2 | blue | 0.9440989 |
| TRINITY_DN35004_c0_g5_i1 | blue | 0.9440626 |
| TRINITY_DN35693_c0_g3_i2 | blue | 0.9440258 |
| TRINITY_DN35883_c1_g11_i2 | blue | 0.9440036 |
| TRINITY_DN36546_c0_g1_i1 | blue | 0.9439544 |
| TRINITY_DN23644_c0_g1_i1 | blue | 0.943931 |
| TRINITY_DN35564_c0_g1_i1 | blue | 0.9438339 |
| TRINITY_DN35754_c3_g1_i4 | blue | 0.9438295 |
| TRINITY_DN31198_c0_g1_i2 | blue | 0.9437573 |
| TRINITY_DN34039_c0_g3_i2 | blue | 0.9437567 |
| TRINITY_DN27416_c0_g3_i2 | blue | 0.9437391 |
| TRINITY_DN38005_c1_g3_i2 | blue | 0.9436279 |
| TRINITY_DN30369_c0_g2_i2 | blue | 0.9435646 |
| TRINITY_DN38858_c1_g2_i6 | blue | 0.9435393 |
| TRINITY_DN36807_c1_g1_i11 | blue | 0.943391 |
| TRINITY_DN30247_c0_g1_i3 | blue | 0.94337 |
| TRINITY_DN37217_c0_g1_i8 | blue | 0.9432317 |
| TRINITY_DN36088_c0_g2_i1 | blue | 0.9430746 |
| TRINITY_DN33071_c5_g1_i1 | blue | 0.9430299 |
| TRINITY_DN27030_c0_g3_i2 | blue | 0.9429777 |
| TRINITY_DN29103_c0_g3_i1 | blue | 0.9429612 |
| TRINITY_DN24337_c0_g1_i1 | blue | 0.9428729 |
| TRINITY_DN33909_c0_g1_i3 | blue | 0.9428305 |
| TRINITY_DN27079_c0_g1_i1 | blue | 0.9428275 |
| TRINITY_DN32390_c0_g1_i2 | blue | 0.9428026 |
| TRINITY_DN28487_c0_g1_i3 | blue | 0.9427507 |
| TRINITY_DN26082_c0_g3_i1 | blue | 0.9427082 |
| TRINITY_DN31830_c1_g1_i6 | blue | 0.9426157 |
| TRINITY_DN22846_c0_g1_i1 | blue | 0.9425472 |
| TRINITY_DN22799_c0_g2_i1 | blue | 0.942472 |
| TRINITY_DN19501_c0_g1_i1 | blue | 0.9423455 |
| TRINITY_DN27818_c0_g1_i1 | blue | 0.9423301 |
| TRINITY_DN25493_c0_g1_i5 | blue | 0.9422122 |
| TRINITY_DN38218_c1_g3_i9 | blue | 0.9422067 |
| TRINITY_DN37797_c1_g1_i1 | blue | 0.9421398 |
| TRINITY_DN29421_c0_g2_i2 | blue | 0.9420355 |
| TRINITY_DN31423_c0_g2_i1 | blue | 0.9419472 |
| TRINITY_DN35056_c2_g2_i1 | blue | 0.9418255 |
| TRINITY_DN19524_c0_g1_i1 | blue | 0.9417972 |
| TRINITY_DN36229_c4_g6_i1 | blue | 0.9417836 |
| TRINITY_DN38391_c1_g5_i2 | blue | 0.9416224 |
| TRINITY_DN26906_c1_g1_i11 | blue | 0.9415598 |
| TRINITY_DN32282_c0_g1_i1 | blue | 0.9412709 |
| TRINITY_DN36916_c0_g2_i9 | blue | 0.9411568 |
| TRINITY_DN36970_c0_g1_i5 | blue | 0.9410114 |
| TRINITY_DN25544_c0_g1_i1 | blue | 0.9409959 |
| TRINITY_DN31963_c0_g1_i2 | blue | 0.9409671 |
| TRINITY_DN33111_c0_g1_i1 | blue | 0.9409647 |
| TRINITY_DN39116_c3_g1_i1 | blue | 0.940942 |
| TRINITY_DN32725_c0_g1_i1 | blue | 0.9407047 |
| TRINITY_DN37285_c0_g1_i2 | blue | 0.9406895 |
| TRINITY_DN29759_c1_g1_i1 | blue | 0.9404368 |
| TRINITY_DN33889_c1_g2_i1 | blue | 0.9403787 |
| TRINITY_DN25249_c0_g2_i2 | blue | 0.9402853 |
| TRINITY_DN32818_c0_g1_i4 | blue | 0.9402838 |
| TRINITY_DN34901_c1_g3_i2 | blue | 0.9401685 |
| TRINITY_DN35114_c1_g1_i2 | blue | 0.939938 |
| TRINITY_DN34270_c1_g1_i1 | blue | 0.9398274 |
| TRINITY_DN30220_c0_g1_i2 | blue | 0.9398252 |
| TRINITY_DN30347_c1_g2_i1 | blue | 0.9397531 |
| TRINITY_DN37641_c1_g2_i2 | blue | 0.9396924 |
| TRINITY_DN30428_c0_g3_i1 | blue | 0.93967 |
| TRINITY_DN38889_c1_g1_i3 | blue | 0.9393757 |
| TRINITY_DN35022_c0_g1_i2 | blue | 0.9393577 |
| TRINITY_DN26678_c0_g2_i1 | blue | 0.9392973 |
| TRINITY_DN33201_c0_g1_i1 | blue | 0.9392518 |
| TRINITY_DN34922_c1_g1_i2 | blue | 0.9392352 |
| TRINITY_DN33440_c0_g1_i1 | blue | 0.9392214 |
| TRINITY_DN29484_c0_g1_i2 | blue | 0.9391697 |
| TRINITY_DN27288_c0_g1_i1 | blue | 0.9391565 |
| TRINITY_DN30907_c0_g1_i2 | blue | 0.9390282 |
| TRINITY_DN33801_c0_g1_i2 | blue | 0.9388035 |
| TRINITY_DN34808_c0_g2_i4 | blue | 0.9388024 |
| TRINITY_DN22047_c0_g1_i1 | blue | 0.938795 |
| TRINITY_DN34896_c0_g3_i1 | blue | 0.9387352 |
| TRINITY_DN23831_c0_g1_i1 | blue | 0.9386428 |
| TRINITY_DN31741_c0_g1_i6 | blue | 0.9386326 |
| TRINITY_DN33249_c0_g2_i2 | blue | 0.9385718 |
| TRINITY_DN35011_c0_g3_i2 | blue | 0.9384164 |
| TRINITY_DN37493_c0_g2_i2 | blue | 0.9383927 |
| TRINITY_DN34762_c0_g2_i8 | blue | 0.9383398 |
| TRINITY_DN33684_c0_g1_i3 | blue | 0.9383263 |
| TRINITY_DN33639_c0_g1_i3 | blue | 0.9381931 |
| TRINITY_DN39238_c1_g4_i5 | blue | 0.9381698 |
| TRINITY_DN24718_c0_g1_i1 | blue | 0.9381492 |
| TRINITY_DN38985_c1_g2_i1 | blue | 0.9381099 |
| TRINITY_DN35738_c0_g3_i8 | blue | 0.9380651 |
| TRINITY_DN34775_c1_g3_i1 | blue | 0.937973 |
| TRINITY_DN36171_c0_g1_i6 | blue | 0.9379635 |
| TRINITY_DN33640_c1_g1_i2 | blue | 0.9379309 |
| TRINITY_DN34629_c0_g2_i3 | blue | 0.9378811 |
| TRINITY_DN30151_c0_g1_i2 | blue | 0.9378421 |
| TRINITY_DN37712_c2_g2_i3 | blue | 0.9377361 |
| TRINITY_DN37489_c2_g12_i1 | blue | 0.9377025 |
| TRINITY_DN35131_c1_g2_i1 | blue | 0.9374421 |
| TRINITY_DN29010_c0_g1_i4 | blue | 0.9374039 |
| TRINITY_DN32251_c0_g2_i1 | blue | 0.9374024 |
| TRINITY_DN22798_c0_g1_i1 | blue | 0.9372533 |
| TRINITY_DN32601_c0_g2_i2 | blue | 0.937226 |
| TRINITY_DN30484_c0_g1_i1 | blue | 0.9372 |
| TRINITY_DN23735_c0_g3_i1 | blue | 0.9370015 |
| TRINITY_DN29132_c0_g1_i5 | blue | 0.9368966 |
| TRINITY_DN29041_c0_g1_i1 | blue | 0.9368725 |
| TRINITY_DN36849_c0_g1_i2 | blue | 0.9367408 |
| TRINITY_DN37262_c0_g5_i1 | blue | 0.9366833 |
| TRINITY_DN35455_c0_g1_i2 | blue | 0.9365767 |
| TRINITY_DN36305_c1_g1_i10 | blue | 0.936557 |
| TRINITY_DN37232_c1_g2_i2 | blue | 0.9364231 |
| TRINITY_DN33284_c0_g1_i7 | blue | 0.9364046 |
| TRINITY_DN27751_c0_g1_i2 | blue | 0.9362898 |
| TRINITY_DN35113_c0_g1_i5 | blue | 0.9361189 |
| TRINITY_DN33357_c0_g1_i1 | blue | 0.9359433 |
| TRINITY_DN36924_c0_g1_i1 | blue | 0.9359344 |
| TRINITY_DN33902_c1_g2_i1 | blue | 0.9357628 |
| TRINITY_DN37493_c0_g1_i1 | blue | 0.9356575 |
| TRINITY_DN19450_c0_g1_i1 | blue | 0.9356537 |
| TRINITY_DN36574_c1_g3_i1 | blue | 0.9356028 |
| TRINITY_DN26340_c0_g1_i2 | blue | 0.9355825 |
| TRINITY_DN37258_c3_g1_i33 | blue | 0.9355425 |
| TRINITY_DN23747_c0_g1_i1 | blue | 0.9353304 |
| TRINITY_DN20930_c0_g1_i1 | blue | 0.9352505 |
| TRINITY_DN33764_c0_g1_i1 | blue | 0.935236 |
| TRINITY_DN28654_c0_g1_i2 | blue | 0.9351382 |
| TRINITY_DN36847_c0_g4_i3 | blue | 0.9351186 |
| TRINITY_DN51615_c0_g1_i1 | blue | 0.9350715 |
| TRINITY_DN35433_c0_g1_i2 | blue | 0.9350522 |
| TRINITY_DN29443_c0_g1_i3 | blue | 0.9350168 |
| TRINITY_DN28891_c0_g1_i1 | blue | 0.9349711 |
| TRINITY_DN28393_c0_g1_i1 | blue | 0.934877 |
| TRINITY_DN37679_c0_g5_i4 | blue | 0.9346792 |
| TRINITY_DN34264_c1_g1_i5 | blue | 0.9345658 |
| TRINITY_DN38379_c1_g1_i1 | blue | 0.9343161 |
| TRINITY_DN24555_c0_g1_i1 | blue | 0.9342717 |
| TRINITY_DN33453_c0_g1_i1 | blue | 0.9342541 |
| TRINITY_DN36810_c0_g2_i5 | blue | 0.9342305 |
| TRINITY_DN23137_c0_g1_i1 | blue | 0.934139 |
| TRINITY_DN35885_c0_g2_i19 | blue | 0.9340441 |
| TRINITY_DN38431_c2_g2_i8 | blue | 0.9339886 |
| TRINITY_DN26877_c0_g2_i1 | blue | 0.9338699 |
| TRINITY_DN25067_c0_g1_i2 | blue | 0.9337952 |
| TRINITY_DN37791_c2_g10_i1 | blue | 0.9337055 |
| TRINITY_DN35693_c0_g1_i4 | blue | 0.9336781 |
| TRINITY_DN26174_c0_g1_i1 | blue | 0.9336103 |
| TRINITY_DN28078_c0_g1_i4 | blue | 0.933578 |
| TRINITY_DN27908_c0_g1_i1 | blue | 0.9335137 |
| TRINITY_DN24564_c0_g2_i1 | blue | 0.9334618 |
| TRINITY_DN27944_c0_g1_i1 | blue | 0.9333357 |
| TRINITY_DN33770_c0_g1_i1 | blue | 0.9333014 |
| TRINITY_DN33820_c0_g2_i5 | blue | 0.9331177 |
| TRINITY_DN37953_c0_g1_i4 | blue | 0.933093 |
| TRINITY_DN37461_c0_g1_i2 | blue | 0.933043 |
| TRINITY_DN33954_c1_g1_i1 | blue | 0.9329874 |
| TRINITY_DN29215_c0_g1_i3 | blue | 0.9329823 |
| TRINITY_DN38496_c0_g1_i1 | blue | 0.9328594 |
| TRINITY_DN38579_c1_g1_i2 | blue | 0.9328099 |
| TRINITY_DN32247_c0_g1_i5 | blue | 0.9325729 |
| TRINITY_DN34889_c0_g1_i3 | blue | 0.9324791 |
| TRINITY_DN32735_c0_g1_i4 | blue | 0.9324115 |
| TRINITY_DN29760_c0_g1_i11 | blue | 0.9323802 |
| TRINITY_DN31202_c0_g1_i1 | blue | 0.9323397 |
| TRINITY_DN34083_c0_g2_i2 | blue | 0.9322781 |
| TRINITY_DN33365_c0_g1_i4 | blue | 0.9322518 |
| TRINITY_DN33456_c0_g1_i2 | blue | 0.9322301 |
| TRINITY_DN20778_c0_g1_i1 | blue | 0.9320314 |
| TRINITY_DN25831_c0_g1_i1 | blue | 0.9320066 |
| TRINITY_DN29988_c0_g1_i1 | blue | 0.9317963 |
| TRINITY_DN36095_c0_g2_i1 | blue | 0.9317788 |
| TRINITY_DN32186_c0_g1_i4 | blue | 0.931704 |
| TRINITY_DN25031_c0_g1_i1 | blue | 0.9316093 |
| TRINITY_DN36312_c0_g1_i4 | blue | 0.9315269 |
| TRINITY_DN38352_c1_g1_i4 | blue | 0.9314261 |
| TRINITY_DN22891_c0_g2_i1 | blue | 0.9312267 |
| TRINITY_DN34731_c0_g2_i9 | blue | 0.9312009 |
| TRINITY_DN32400_c0_g3_i1 | blue | 0.9310548 |
| TRINITY_DN30295_c1_g1_i2 | blue | 0.931019 |
| TRINITY_DN37241_c0_g1_i1 | blue | 0.9310163 |
| TRINITY_DN27575_c0_g1_i1 | blue | 0.9309641 |
| TRINITY_DN29613_c0_g1_i1 | blue | 0.9309131 |
| TRINITY_DN20400_c0_g1_i1 | blue | 0.9309083 |
| TRINITY_DN30555_c0_g1_i2 | blue | 0.9308707 |
| TRINITY_DN25003_c0_g1_i1 | blue | 0.9308643 |
| TRINITY_DN35152_c0_g4_i3 | blue | 0.9307677 |
| TRINITY_DN27510_c0_g1_i1 | blue | 0.9306828 |
| TRINITY_DN34201_c0_g1_i1 | blue | 0.9305937 |
| TRINITY_DN25923_c0_g1_i3 | blue | 0.9305917 |
| TRINITY_DN34681_c0_g5_i11 | blue | 0.930549 |
| TRINITY_DN28616_c0_g1_i1 | blue | 0.9303549 |
| TRINITY_DN39153_c3_g2_i2 | blue | 0.9299134 |
| TRINITY_DN30550_c0_g2_i7 | blue | 0.9297775 |
| TRINITY_DN36648_c1_g4_i1 | blue | 0.9297653 |
| TRINITY_DN18601_c0_g3_i1 | blue | 0.9294355 |
| TRINITY_DN36948_c0_g1_i1 | blue | 0.9292895 |
| TRINITY_DN35966_c0_g6_i1 | blue | 0.929284 |
| TRINITY_DN31044_c0_g1_i3 | blue | 0.9290709 |
| TRINITY_DN40470_c0_g1_i1 | blue | 0.9289059 |
| TRINITY_DN31010_c0_g1_i1 | blue | 0.9287395 |
| TRINITY_DN22773_c0_g1_i1 | blue | 0.9287386 |
| TRINITY_DN35241_c0_g1_i3 | blue | 0.9287362 |
| TRINITY_DN26151_c0_g1_i4 | blue | 0.9286668 |
| TRINITY_DN38186_c0_g2_i10 | blue | 0.9286369 |
| TRINITY_DN36120_c2_g1_i1 | blue | 0.928608 |
| TRINITY_DN30214_c0_g1_i3 | blue | 0.9285648 |
| TRINITY_DN38349_c0_g2_i2 | blue | 0.9285117 |
| TRINITY_DN29730_c0_g1_i1 | blue | 0.9283743 |
| TRINITY_DN31081_c0_g4_i4 | blue | 0.9283523 |
| TRINITY_DN25171_c0_g1_i3 | blue | 0.9282907 |
| TRINITY_DN35704_c1_g1_i2 | blue | 0.9282453 |
| TRINITY_DN23859_c0_g2_i1 | blue | 0.9282301 |
| TRINITY_DN36932_c0_g3_i1 | blue | 0.9282171 |
| TRINITY_DN25908_c0_g1_i1 | blue | 0.928136 |
| TRINITY_DN29266_c0_g1_i3 | blue | 0.928127 |
| TRINITY_DN37231_c0_g3_i4 | blue | 0.9280606 |
| TRINITY_DN33729_c0_g1_i2 | blue | 0.9279339 |
| TRINITY_DN35004_c0_g1_i9 | blue | 0.9279333 |
| TRINITY_DN27184_c0_g1_i1 | blue | 0.9278134 |
| TRINITY_DN8723_c0_g1_i1 | blue | 0.9277827 |
| TRINITY_DN35601_c1_g1_i1 | blue | 0.9277723 |
| TRINITY_DN35301_c1_g1_i1 | blue | 0.9277603 |
| TRINITY_DN31233_c0_g1_i1 | blue | 0.9277226 |
| TRINITY_DN31481_c1_g1_i1 | blue | 0.927719 |
| TRINITY_DN37177_c1_g1_i22 | blue | 0.9276781 |
| TRINITY_DN35307_c0_g1_i1 | blue | 0.927648 |
| TRINITY_DN25163_c0_g2_i1 | blue | 0.927616 |
| TRINITY_DN24998_c0_g1_i1 | blue | 0.9275685 |
| TRINITY_DN32986_c1_g1_i1 | blue | 0.9275053 |
| TRINITY_DN32586_c0_g1_i1 | blue | 0.9274147 |
| TRINITY_DN38063_c0_g1_i4 | blue | 0.9273941 |
| TRINITY_DN34939_c0_g2_i14 | blue | 0.9273247 |
| TRINITY_DN38558_c0_g1_i7 | blue | 0.9272939 |
| TRINITY_DN23087_c0_g2_i1 | blue | 0.9271941 |
| TRINITY_DN34146_c0_g1_i1 | blue | 0.9271593 |
| TRINITY_DN33579_c0_g3_i4 | blue | 0.9270554 |
| TRINITY_DN32970_c1_g1_i3 | blue | 0.9269425 |
| TRINITY_DN37016_c0_g1_i5 | blue | 0.9269349 |
| TRINITY_DN25939_c0_g1_i2 | blue | 0.9268316 |
| TRINITY_DN37488_c0_g5_i3 | blue | 0.9268006 |
| TRINITY_DN36541_c0_g1_i8 | blue | 0.9266218 |
| TRINITY_DN25979_c0_g1_i1 | blue | 0.9265377 |
| TRINITY_DN30558_c0_g1_i3 | blue | 0.9263636 |
| TRINITY_DN33166_c0_g1_i6 | blue | 0.9262974 |
| TRINITY_DN33955_c0_g1_i3 | blue | 0.9262043 |
| TRINITY_DN30005_c0_g1_i6 | blue | 0.9261392 |
| TRINITY_DN33906_c0_g3_i1 | blue | 0.9260971 |
| TRINITY_DN28912_c0_g2_i1 | blue | 0.9259737 |
| TRINITY_DN26545_c0_g3_i1 | blue | 0.9259166 |
| TRINITY_DN27884_c0_g1_i1 | blue | 0.9258761 |
| TRINITY_DN52087_c0_g1_i1 | blue | 0.9257427 |
| TRINITY_DN26108_c0_g1_i1 | blue | 0.9257299 |
| TRINITY_DN24991_c0_g2_i2 | blue | 0.925641 |
| TRINITY_DN35325_c0_g1_i4 | blue | 0.9255413 |
| TRINITY_DN33844_c0_g5_i1 | blue | 0.9254381 |
| TRINITY_DN29244_c0_g3_i1 | blue | 0.9254347 |
| TRINITY_DN34412_c0_g3_i3 | blue | 0.9253338 |
| TRINITY_DN38127_c1_g3_i1 | blue | 0.9252826 |
| TRINITY_DN31306_c0_g1_i3 | blue | 0.9252154 |
| TRINITY_DN32654_c0_g3_i1 | blue | 0.9252138 |
| TRINITY_DN30411_c1_g1_i1 | blue | 0.9250709 |
| TRINITY_DN36341_c2_g5_i6 | blue | 0.9249888 |
| TRINITY_DN39329_c14_g5_i2 | blue | 0.9248274 |
| TRINITY_DN36448_c0_g1_i3 | blue | 0.9248162 |
| TRINITY_DN22743_c0_g1_i1 | blue | 0.9247299 |
| TRINITY_DN17129_c0_g2_i1 | blue | 0.9246929 |
| TRINITY_DN29879_c0_g3_i6 | blue | 0.9246814 |
| TRINITY_DN34876_c0_g1_i3 | blue | 0.9245882 |
| TRINITY_DN31635_c0_g1_i6 | blue | 0.9242748 |
| TRINITY_DN38247_c0_g3_i2 | blue | 0.9240986 |
| TRINITY_DN28863_c0_g2_i1 | blue | 0.9240899 |
| TRINITY_DN25412_c0_g2_i1 | blue | 0.9240421 |
| TRINITY_DN37329_c1_g2_i1 | blue | 0.9240015 |
| TRINITY_DN39299_c4_g3_i4 | blue | 0.9238368 |
| TRINITY_DN28252_c1_g1_i1 | blue | 0.9236383 |
| TRINITY_DN27889_c0_g1_i2 | blue | 0.9234582 |
| TRINITY_DN36958_c1_g1_i14 | blue | 0.9232084 |
| TRINITY_DN23168_c0_g1_i1 | blue | 0.9231228 |
| TRINITY_DN36402_c1_g4_i1 | blue | 0.9230408 |
| TRINITY_DN26725_c0_g1_i1 | blue | 0.9228913 |
| TRINITY_DN30767_c0_g1_i3 | blue | 0.9226641 |
| TRINITY_DN20966_c0_g1_i2 | blue | 0.9226411 |
| TRINITY_DN39142_c0_g2_i1 | blue | 0.9225101 |
| TRINITY_DN32400_c0_g1_i1 | blue | 0.9223435 |
| TRINITY_DN39060_c0_g1_i1 | blue | 0.9222626 |
| TRINITY_DN34446_c0_g2_i1 | blue | 0.922245 |
| TRINITY_DN24246_c0_g1_i1 | blue | 0.9222253 |
| TRINITY_DN39220_c6_g12_i2 | blue | 0.9222178 |
| TRINITY_DN64689_c0_g1_i1 | blue | 0.9216635 |
| TRINITY_DN30105_c1_g2_i5 | blue | 0.9215011 |
| TRINITY_DN35733_c0_g1_i2 | blue | 0.9213922 |
| TRINITY_DN34153_c0_g1_i8 | blue | 0.9213001 |
| TRINITY_DN38653_c0_g2_i7 | blue | 0.9212974 |
| TRINITY_DN39191_c3_g5_i2 | blue | 0.9212006 |
| TRINITY_DN33210_c0_g2_i2 | blue | 0.9211431 |
| TRINITY_DN38829_c0_g2_i1 | blue | 0.9207085 |
| TRINITY_DN34891_c0_g3_i1 | blue | 0.9206974 |
| TRINITY_DN36508_c0_g1_i9 | blue | 0.9205179 |
| TRINITY_DN26701_c0_g3_i1 | blue | 0.9204377 |
| TRINITY_DN33577_c1_g1_i2 | blue | 0.9204238 |
| TRINITY_DN30554_c0_g1_i4 | blue | 0.9202149 |
| TRINITY_DN32551_c0_g1_i1 | blue | 0.9201732 |
| TRINITY_DN29901_c0_g1_i2 | blue | 0.9200502 |
| TRINITY_DN34519_c0_g10_i1 | blue | 0.920024 |
| TRINITY_DN29081_c0_g1_i2 | blue | 0.9199937 |
| TRINITY_DN64889_c0_g1_i1 | blue | 0.9199735 |
| TRINITY_DN35653_c1_g1_i5 | blue | 0.9198863 |
| TRINITY_DN34898_c0_g4_i4 | blue | 0.9198815 |
| TRINITY_DN19088_c0_g2_i2 | blue | 0.9198516 |
| TRINITY_DN35934_c0_g4_i5 | blue | 0.9197754 |
| TRINITY_DN38493_c0_g2_i5 | blue | 0.9197714 |
| TRINITY_DN35087_c0_g1_i1 | blue | 0.9197401 |
| TRINITY_DN24765_c0_g3_i1 | blue | 0.9197256 |
| TRINITY_DN37563_c0_g3_i9 | blue | 0.9197195 |
| TRINITY_DN10835_c0_g1_i1 | blue | 0.919449 |
| TRINITY_DN37648_c0_g2_i1 | blue | 0.9193457 |
| TRINITY_DN35244_c0_g2_i10 | blue | 0.919271 |
| TRINITY_DN28822_c0_g1_i3 | blue | 0.9192377 |
| TRINITY_DN33266_c0_g2_i1 | blue | 0.9190969 |
| TRINITY_DN34421_c0_g1_i1 | blue | 0.9190864 |
| TRINITY_DN38731_c4_g5_i6 | blue | 0.9189671 |
| TRINITY_DN26299_c0_g1_i1 | blue | 0.91859 |
| TRINITY_DN39309_c2_g5_i1 | blue | 0.9184461 |
| TRINITY_DN22333_c0_g2_i1 | blue | 0.9184263 |
| TRINITY_DN35002_c0_g1_i9 | blue | 0.9182333 |
| TRINITY_DN33245_c0_g1_i14 | blue | 0.9182176 |
| TRINITY_DN33874_c0_g3_i3 | blue | 0.9181557 |
| TRINITY_DN34949_c0_g4_i4 | blue | 0.918066 |
| TRINITY_DN37635_c0_g7_i2 | blue | 0.9180227 |
| TRINITY_DN23451_c0_g1_i1 | blue | 0.9179404 |
| TRINITY_DN37230_c0_g1_i3 | blue | 0.9178094 |
| TRINITY_DN30256_c0_g1_i2 | blue | 0.9177669 |
| TRINITY_DN36393_c0_g4_i1 | blue | 0.9177595 |
| TRINITY_DN38465_c0_g2_i3 | blue | 0.9177386 |
| TRINITY_DN17667_c0_g1_i1 | blue | 0.9176597 |
| TRINITY_DN34782_c0_g1_i1 | blue | 0.9176273 |
| TRINITY_DN30224_c1_g4_i1 | blue | 0.9175382 |
| TRINITY_DN29069_c0_g1_i1 | blue | 0.9171746 |
| TRINITY_DN39023_c0_g1_i1 | blue | 0.9170725 |
| TRINITY_DN33987_c0_g2_i2 | blue | 0.9170686 |
| TRINITY_DN31108_c0_g1_i1 | blue | 0.9170308 |
| TRINITY_DN37531_c0_g1_i2 | blue | 0.9168186 |
| TRINITY_DN30801_c1_g1_i1 | blue | 0.9167426 |
| TRINITY_DN31193_c0_g1_i2 | blue | 0.9165818 |
| TRINITY_DN38234_c0_g1_i1 | blue | 0.9165794 |
| TRINITY_DN37995_c2_g1_i4 | blue | 0.9164974 |
| TRINITY_DN39996_c0_g1_i1 | blue | 0.9162927 |
| TRINITY_DN38782_c1_g1_i10 | blue | 0.9162156 |
| TRINITY_DN35726_c1_g3_i6 | blue | 0.9157818 |
| TRINITY_DN23671_c0_g1_i1 | blue | 0.9155568 |
| TRINITY_DN38532_c0_g5_i1 | blue | 0.9152303 |
| TRINITY_DN31218_c0_g1_i9 | blue | 0.9150874 |
| TRINITY_DN26657_c0_g1_i1 | blue | 0.9150043 |
| TRINITY_DN38178_c0_g1_i1 | blue | 0.9147803 |
| TRINITY_DN29842_c0_g1_i7 | blue | 0.9146934 |
| TRINITY_DN37652_c1_g3_i2 | blue | 0.9146107 |
| TRINITY_DN38923_c0_g1_i3 | blue | 0.9145982 |
| TRINITY_DN23729_c0_g1_i1 | blue | 0.9144224 |
| TRINITY_DN38731_c4_g7_i1 | blue | 0.9143871 |
| TRINITY_DN37497_c1_g1_i3 | blue | 0.9141707 |
| TRINITY_DN27204_c0_g1_i1 | blue | 0.9139729 |
| TRINITY_DN37105_c0_g1_i3 | blue | 0.9139361 |
| TRINITY_DN35889_c0_g1_i1 | blue | 0.9139323 |
| TRINITY_DN28547_c0_g1_i1 | blue | 0.9138369 |
| TRINITY_DN22283_c0_g2_i3 | blue | 0.9135953 |
| TRINITY_DN27594_c0_g1_i1 | blue | 0.9135941 |
| TRINITY_DN36000_c1_g1_i1 | blue | 0.9135272 |
| TRINITY_DN37211_c1_g1_i5 | blue | 0.9134341 |
| TRINITY_DN33397_c1_g1_i1 | blue | 0.9133874 |
| TRINITY_DN38574_c0_g3_i3 | blue | 0.9133521 |
| TRINITY_DN30672_c0_g3_i1 | blue | 0.9133206 |
| TRINITY_DN30807_c0_g1_i1 | blue | 0.9132202 |
| TRINITY_DN33935_c0_g8_i2 | blue | 0.9132192 |
| TRINITY_DN36750_c2_g3_i2 | blue | 0.9131819 |
| TRINITY_DN35727_c0_g1_i6 | blue | 0.9131231 |
| TRINITY_DN35080_c0_g2_i1 | blue | 0.9129797 |
| TRINITY_DN23261_c0_g1_i1 | blue | 0.9129557 |
| TRINITY_DN39029_c0_g1_i8 | blue | 0.9126988 |
| TRINITY_DN39220_c6_g13_i1 | blue | 0.9123053 |
| TRINITY_DN39444_c0_g1_i1 | blue | 0.9121854 |
| TRINITY_DN36953_c1_g1_i5 | blue | 0.9121671 |
| TRINITY_DN32830_c0_g2_i2 | blue | 0.9121617 |
| TRINITY_DN26808_c0_g2_i3 | blue | 0.9119253 |
| TRINITY_DN36831_c0_g2_i2 | blue | 0.9119097 |
| TRINITY_DN30737_c0_g1_i3 | blue | 0.9117032 |
| TRINITY_DN32591_c0_g1_i7 | blue | 0.9116888 |
| TRINITY_DN35679_c1_g2_i4 | blue | 0.9116755 |
| TRINITY_DN39329_c13_g8_i1 | blue | 0.9112998 |
| TRINITY_DN24774_c0_g1_i1 | blue | 0.9111161 |
| TRINITY_DN22632_c0_g1_i1 | blue | 0.9110613 |
| TRINITY_DN34057_c0_g1_i1 | blue | 0.9109352 |
| TRINITY_DN35516_c0_g1_i1 | blue | 0.9105346 |
| TRINITY_DN28573_c0_g1_i2 | blue | 0.9104617 |
| TRINITY_DN31430_c0_g2_i2 | blue | 0.9102493 |
| TRINITY_DN33577_c1_g5_i1 | blue | 0.9101604 |
| TRINITY_DN28925_c0_g1_i1 | blue | 0.9100152 |
| TRINITY_DN33378_c1_g1_i3 | blue | 0.9099316 |
| TRINITY_DN34577_c0_g1_i4 | blue | 0.9098505 |
| TRINITY_DN28325_c0_g1_i1 | blue | 0.9095493 |
| TRINITY_DN28631_c0_g3_i6 | blue | 0.9093187 |
| TRINITY_DN37919_c1_g3_i3 | blue | 0.9089844 |
| TRINITY_DN37262_c0_g1_i3 | blue | 0.9089743 |
| TRINITY_DN34330_c1_g4_i1 | blue | 0.9088728 |
| TRINITY_DN26263_c0_g1_i2 | blue | 0.9087486 |
| TRINITY_DN31768_c0_g1_i1 | blue | 0.9086881 |
| TRINITY_DN35432_c0_g2_i1 | blue | 0.908629 |
| TRINITY_DN16475_c0_g1_i1 | blue | 0.9086063 |
| TRINITY_DN26532_c0_g1_i3 | blue | 0.9085859 |
| TRINITY_DN22819_c0_g2_i1 | blue | 0.9083154 |
| TRINITY_DN26597_c0_g2_i1 | blue | 0.9081311 |
| TRINITY_DN20081_c0_g1_i1 | blue | 0.9080515 |
| TRINITY_DN29454_c0_g1_i1 | blue | 0.9077405 |
| TRINITY_DN27094_c0_g1_i5 | blue | 0.9076528 |
| TRINITY_DN32734_c0_g1_i1 | blue | 0.9074879 |
| TRINITY_DN34038_c0_g1_i1 | blue | 0.9072416 |
| TRINITY_DN34412_c0_g1_i5 | blue | 0.9071147 |
| TRINITY_DN23676_c0_g1_i1 | blue | 0.9069664 |
| TRINITY_DN34652_c0_g1_i3 | blue | 0.9069532 |
| TRINITY_DN38680_c0_g2_i1 | blue | 0.9069444 |
| TRINITY_DN34164_c0_g1_i1 | blue | 0.9068569 |
| TRINITY_DN30973_c1_g2_i1 | blue | 0.9067995 |
| TRINITY_DN35613_c0_g1_i1 | blue | 0.9067928 |
| TRINITY_DN31469_c0_g1_i2 | blue | 0.9066877 |
| TRINITY_DN31177_c1_g2_i1 | blue | 0.9064176 |
| TRINITY_DN35142_c0_g6_i6 | blue | 0.906382 |
| TRINITY_DN24902_c0_g1_i4 | blue | 0.9062627 |
| TRINITY_DN32207_c0_g1_i5 | blue | 0.90613 |
| TRINITY_DN29395_c0_g1_i1 | blue | 0.9060037 |
| TRINITY_DN33604_c0_g1_i1 | blue | 0.9059804 |
| TRINITY_DN26154_c1_g5_i1 | blue | 0.9057517 |
| TRINITY_DN33577_c1_g7_i2 | blue | 0.9057016 |
| TRINITY_DN37178_c1_g4_i1 | blue | 0.9056568 |
| TRINITY_DN28911_c0_g1_i4 | blue | 0.9055668 |
| TRINITY_DN23346_c0_g1_i1 | blue | 0.9052459 |
| TRINITY_DN31242_c0_g1_i2 | blue | 0.9049374 |
| TRINITY_DN27484_c0_g1_i2 | blue | 0.9048751 |
| TRINITY_DN39060_c0_g6_i3 | blue | 0.90472 |
| TRINITY_DN32109_c0_g2_i1 | blue | 0.9045669 |
| TRINITY_DN23059_c0_g1_i1 | blue | 0.904504 |
| TRINITY_DN27616_c0_g1_i5 | blue | 0.9043365 |
| TRINITY_DN34129_c0_g2_i1 | blue | 0.9043007 |
| TRINITY_DN26446_c0_g2_i1 | blue | 0.9042999 |
| TRINITY_DN30920_c0_g1_i1 | blue | 0.9041901 |
| TRINITY_DN34304_c0_g2_i4 | blue | 0.9041692 |
| TRINITY_DN31568_c0_g1_i1 | blue | 0.9040241 |
| TRINITY_DN28164_c1_g2_i1 | blue | 0.9038158 |
| TRINITY_DN37385_c0_g3_i1 | blue | 0.9037972 |
| TRINITY_DN35744_c0_g2_i1 | blue | 0.9037053 |
| TRINITY_DN33577_c1_g8_i2 | blue | 0.9036069 |
| TRINITY_DN32361_c0_g3_i1 | blue | 0.9035728 |
| TRINITY_DN33812_c0_g1_i1 | blue | 0.9035619 |
| TRINITY_DN35679_c0_g2_i2 | blue | 0.9035619 |
| TRINITY_DN29659_c0_g2_i1 | blue | 0.9035438 |
| TRINITY_DN29703_c0_g1_i1 | blue | 0.9034487 |
| TRINITY_DN38782_c1_g3_i1 | blue | 0.9032001 |
| TRINITY_DN39160_c0_g4_i1 | blue | 0.9029023 |
| TRINITY_DN38961_c0_g5_i4 | blue | 0.9028193 |
| TRINITY_DN31560_c0_g4_i5 | blue | 0.9028039 |
| TRINITY_DN24665_c0_g1_i2 | blue | 0.9026881 |
| TRINITY_DN28755_c0_g1_i2 | blue | 0.9025076 |
| TRINITY_DN31245_c2_g1_i7 | blue | 0.9023639 |
| TRINITY_DN28809_c1_g1_i1 | blue | 0.9023583 |
| TRINITY_DN25090_c0_g2_i1 | blue | 0.9021384 |
| TRINITY_DN2348_c0_g1_i1 | blue | 0.9020954 |
| TRINITY_DN35800_c0_g2_i6 | blue | 0.9019145 |
| TRINITY_DN38697_c0_g2_i7 | blue | 0.9017757 |
| TRINITY_DN33748_c1_g1_i3 | blue | 0.9017545 |
| TRINITY_DN23550_c0_g1_i1 | blue | 0.9017184 |
| TRINITY_DN29762_c0_g1_i4 | blue | 0.901577 |
| TRINITY_DN38616_c2_g4_i1 | blue | 0.9015227 |
| TRINITY_DN34329_c0_g1_i7 | blue | 0.9015205 |
| TRINITY_DN21847_c0_g1_i1 | blue | 0.9015179 |
| TRINITY_DN34672_c0_g1_i9 | blue | 0.9011928 |
| TRINITY_DN28872_c0_g1_i1 | blue | 0.9011774 |
| TRINITY_DN22465_c0_g1_i1 | blue | 0.9011418 |
| TRINITY_DN39068_c0_g18_i1 | blue | 0.901104 |
| TRINITY_DN56212_c0_g1_i1 | blue | 0.9010758 |
| TRINITY_DN33148_c0_g2_i1 | blue | 0.9009597 |
| TRINITY_DN23800_c0_g1_i1 | blue | 0.9006276 |
| TRINITY_DN37671_c0_g1_i3 | blue | 0.9005429 |
| TRINITY_DN33491_c0_g3_i1 | blue | 0.9003741 |
| TRINITY_DN31128_c0_g3_i2 | blue | 0.9001689 |
| TRINITY_DN37791_c2_g5_i1 | blue | 0.9001067 |
| TRINITY_DN35491_c1_g1_i1 | blue | 0.9000297 |
| TRINITY_DN27227_c0_g1_i1 | blue | 0.8998461 |
| TRINITY_DN15979_c0_g2_i1 | blue | 0.8998357 |
| TRINITY_DN38644_c0_g1_i2 | blue | 0.89977 |
| TRINITY_DN36860_c0_g1_i12 | blue | 0.8997412 |
| TRINITY_DN31509_c0_g1_i1 | blue | 0.8995905 |
| TRINITY_DN30238_c0_g1_i1 | blue | 0.8995158 |
| TRINITY_DN28736_c0_g1_i1 | blue | 0.8994101 |
| TRINITY_DN33012_c1_g5_i1 | blue | 0.8993497 |
| TRINITY_DN33294_c0_g2_i1 | blue | 0.8990438 |
| TRINITY_DN22871_c0_g1_i1 | blue | 0.8990317 |
| TRINITY_DN37967_c3_g1_i4 | blue | 0.8989968 |
| TRINITY_DN35278_c1_g3_i1 | blue | 0.8988833 |
| TRINITY_DN31577_c1_g2_i1 | blue | 0.8988737 |
| TRINITY_DN22527_c1_g2_i1 | blue | 0.8987328 |
| TRINITY_DN37339_c0_g1_i1 | blue | 0.8986461 |
| TRINITY_DN30482_c0_g3_i7 | blue | 0.8986268 |
| TRINITY_DN23646_c0_g1_i1 | blue | 0.8986101 |
| TRINITY_DN39307_c2_g3_i1 | blue | 0.8985829 |
| TRINITY_DN33158_c1_g1_i2 | blue | 0.898581 |
| TRINITY_DN35098_c0_g2_i2 | blue | 0.8984721 |
| TRINITY_DN25733_c0_g3_i1 | blue | 0.8983681 |
| TRINITY_DN37262_c0_g2_i1 | blue | 0.8982408 |
| TRINITY_DN24002_c0_g1_i2 | blue | 0.8981654 |
| TRINITY_DN38631_c1_g2_i1 | blue | 0.8981192 |
| TRINITY_DN37299_c0_g1_i3 | blue | 0.8979767 |
| TRINITY_DN3210_c0_g1_i1 | blue | 0.8978275 |
| TRINITY_DN30597_c0_g2_i1 | blue | 0.8976551 |
| TRINITY_DN35007_c1_g1_i2 | blue | 0.8976255 |
| TRINITY_DN28735_c0_g1_i1 | blue | 0.8974504 |
| TRINITY_DN35965_c0_g1_i1 | blue | 0.8974308 |
| TRINITY_DN31344_c0_g2_i3 | blue | 0.8974044 |
| TRINITY_DN37976_c0_g2_i1 | blue | 0.8973655 |
| TRINITY_DN38037_c0_g4_i1 | blue | 0.8973299 |
| TRINITY_DN16602_c0_g1_i1 | blue | 0.8973237 |
| TRINITY_DN32881_c0_g2_i1 | blue | 0.8972464 |
| TRINITY_DN33947_c0_g3_i1 | blue | 0.897 |
| TRINITY_DN38722_c0_g1_i8 | blue | 0.8969255 |
| TRINITY_DN34590_c2_g2_i2 | blue | 0.8969145 |
| TRINITY_DN26937_c0_g1_i1 | blue | 0.8966356 |
| TRINITY_DN27285_c0_g1_i3 | blue | 0.896613 |
| TRINITY_DN38731_c4_g2_i1 | blue | 0.8964175 |
| TRINITY_DN39083_c0_g7_i1 | blue | 0.8962092 |
| TRINITY_DN37383_c0_g2_i1 | blue | 0.8961948 |
| TRINITY_DN36161_c0_g1_i9 | blue | 0.8960262 |
| TRINITY_DN36639_c1_g1_i2 | blue | 0.8958812 |
| TRINITY_DN19715_c0_g3_i1 | blue | 0.8956174 |
| TRINITY_DN25679_c0_g1_i1 | blue | 0.8955557 |
| TRINITY_DN27646_c0_g1_i3 | blue | 0.8955488 |
| TRINITY_DN25272_c0_g1_i1 | blue | 0.8954842 |
| TRINITY_DN31212_c0_g1_i2 | blue | 0.8952272 |
| TRINITY_DN39028_c0_g4_i2 | blue | 0.8944864 |
| TRINITY_DN33379_c0_g1_i3 | blue | 0.8944757 |
| TRINITY_DN26978_c0_g1_i1 | blue | 0.8944173 |
| TRINITY_DN28467_c0_g1_i1 | blue | 0.8943876 |
| TRINITY_DN18967_c0_g3_i1 | blue | 0.894145 |
| TRINITY_DN37493_c0_g3_i3 | blue | 0.8935011 |
| TRINITY_DN34147_c0_g1_i1 | blue | 0.8933305 |
| TRINITY_DN36000_c1_g3_i3 | blue | 0.8932077 |
| TRINITY_DN33417_c0_g2_i1 | blue | 0.8930831 |
| TRINITY_DN27396_c0_g2_i1 | blue | 0.8930699 |
| TRINITY_DN19010_c0_g1_i1 | blue | 0.8925098 |
| TRINITY_DN32385_c0_g1_i6 | blue | 0.8923474 |
| TRINITY_DN31769_c0_g1_i1 | blue | 0.8922293 |
| TRINITY_DN24397_c0_g3_i1 | blue | 0.8921792 |
| TRINITY_DN37208_c0_g5_i2 | blue | 0.8920619 |
| TRINITY_DN34519_c0_g2_i1 | blue | 0.8920299 |
| TRINITY_DN38378_c0_g1_i8 | blue | 0.891937 |
| TRINITY_DN38703_c0_g1_i1 | blue | 0.8918651 |
| TRINITY_DN25426_c0_g1_i5 | blue | 0.8918632 |
| TRINITY_DN27187_c0_g4_i1 | blue | 0.8917015 |
| TRINITY_DN22365_c0_g1_i1 | blue | 0.891698 |
| TRINITY_DN28164_c1_g1_i1 | blue | 0.8916799 |
| TRINITY_DN32100_c0_g1_i4 | blue | 0.8915061 |
| TRINITY_DN24202_c0_g1_i1 | blue | 0.8912188 |
| TRINITY_DN28565_c0_g1_i3 | blue | 0.891028 |
| TRINITY_DN33251_c0_g2_i1 | blue | 0.891018 |
| TRINITY_DN29311_c0_g2_i1 | blue | 0.8909561 |
| TRINITY_DN270_c0_g1_i1 | blue | 0.8908356 |
| TRINITY_DN33991_c0_g1_i2 | blue | 0.8908138 |
| TRINITY_DN36402_c0_g2_i1 | blue | 0.890548 |
| TRINITY_DN12926_c0_g2_i1 | blue | 0.8905153 |
| TRINITY_DN33741_c0_g3_i1 | blue | 0.890452 |
| TRINITY_DN28318_c0_g1_i1 | blue | 0.8904474 |
| TRINITY_DN33697_c0_g1_i2 | blue | 0.8904327 |
| TRINITY_DN34784_c0_g1_i5 | blue | 0.8902569 |
| TRINITY_DN34971_c0_g1_i2 | blue | 0.8902547 |
| TRINITY_DN24880_c0_g1_i2 | blue | 0.8901265 |
| TRINITY_DN34430_c0_g3_i1 | blue | 0.8899811 |
| TRINITY_DN38839_c0_g4_i5 | blue | 0.8891828 |
| TRINITY_DN13600_c0_g1_i1 | blue | 0.8891721 |
| TRINITY_DN29223_c0_g1_i1 | blue | 0.8890791 |
| TRINITY_DN30664_c0_g1_i1 | blue | 0.8890718 |
| TRINITY_DN37006_c13_g2_i1 | blue | 0.8890607 |
| TRINITY_DN37053_c0_g7_i3 | blue | 0.8888978 |
| TRINITY_DN39269_c5_g6_i9 | blue | 0.8887626 |
| TRINITY_DN33043_c0_g1_i3 | blue | 0.8885996 |
| TRINITY_DN24587_c0_g1_i1 | blue | 0.8885197 |
| TRINITY_DN38144_c1_g1_i12 | blue | 0.8885195 |
| TRINITY_DN30979_c0_g1_i1 | blue | 0.888364 |
| TRINITY_DN38733_c1_g1_i4 | blue | 0.8883608 |
| TRINITY_DN32751_c0_g1_i3 | blue | 0.8883369 |
| TRINITY_DN39006_c0_g2_i1 | blue | 0.8879459 |
| TRINITY_DN39023_c0_g10_i1 | blue | 0.8878732 |
| TRINITY_DN26281_c0_g1_i2 | blue | 0.8877579 |
| TRINITY_DN26255_c0_g1_i3 | blue | 0.8876093 |
| TRINITY_DN33238_c1_g1_i2 | blue | 0.8875583 |
| TRINITY_DN23735_c0_g2_i1 | blue | 0.8875055 |
| TRINITY_DN35390_c2_g1_i9 | blue | 0.8874948 |
| TRINITY_DN37960_c1_g5_i1 | blue | 0.8874284 |
| TRINITY_DN37178_c1_g2_i1 | blue | 0.8873407 |
| TRINITY_DN38749_c0_g1_i5 | blue | 0.8869129 |
| TRINITY_DN28696_c1_g1_i1 | blue | 0.8867623 |
| TRINITY_DN33535_c0_g1_i4 | blue | 0.8866079 |
| TRINITY_DN32615_c0_g2_i3 | blue | 0.8865347 |
| TRINITY_DN31459_c0_g1_i3 | blue | 0.8862474 |
| TRINITY_DN25739_c0_g1_i1 | blue | 0.8860498 |
| TRINITY_DN35140_c1_g1_i1 | blue | 0.8858241 |
| TRINITY_DN25580_c0_g1_i1 | blue | 0.8856947 |
| TRINITY_DN29668_c0_g1_i2 | blue | 0.8855536 |
| TRINITY_DN39220_c6_g9_i1 | blue | 0.8854833 |
| TRINITY_DN36803_c0_g1_i1 | blue | 0.8854125 |
| TRINITY_DN26356_c0_g2_i1 | blue | 0.8853216 |
| TRINITY_DN39308_c11_g3_i4 | blue | 0.8851436 |
| TRINITY_DN33941_c1_g1_i1 | blue | 0.8850813 |
| TRINITY_DN38211_c0_g3_i1 | blue | 0.8848574 |
| TRINITY_DN31300_c0_g7_i2 | blue | 0.8847702 |
| TRINITY_DN31458_c0_g2_i3 | blue | 0.8841333 |
| TRINITY_DN38468_c0_g1_i1 | blue | 0.8840841 |
| TRINITY_DN28793_c0_g2_i2 | blue | 0.884075 |
| TRINITY_DN35001_c0_g1_i5 | blue | 0.8840496 |
| TRINITY_DN27252_c0_g1_i1 | blue | 0.883963 |
| TRINITY_DN33718_c0_g1_i12 | blue | 0.8835117 |
| TRINITY_DN18624_c0_g2_i1 | blue | 0.8833504 |
| TRINITY_DN31645_c0_g1_i1 | blue | 0.8833367 |
| TRINITY_DN68661_c0_g1_i1 | blue | 0.883324 |
| TRINITY_DN38538_c0_g3_i2 | blue | 0.8829557 |
| TRINITY_DN33452_c0_g2_i1 | blue | 0.882883 |
| TRINITY_DN23703_c0_g1_i1 | blue | 0.8826653 |
| TRINITY_DN35638_c1_g2_i3 | blue | 0.8826191 |
| TRINITY_DN39307_c2_g5_i1 | blue | 0.8823213 |
| TRINITY_DN37376_c0_g1_i3 | blue | 0.8820479 |
| TRINITY_DN37976_c0_g1_i1 | blue | 0.8818512 |
| TRINITY_DN27968_c0_g1_i2 | blue | 0.8816744 |
| TRINITY_DN67389_c0_g1_i1 | blue | 0.8813958 |
| TRINITY_DN31771_c1_g5_i1 | blue | 0.8813247 |
| TRINITY_DN39257_c1_g4_i3 | blue | 0.8810523 |
| TRINITY_DN24030_c0_g1_i1 | blue | 0.8810296 |
| TRINITY_DN31851_c1_g1_i1 | blue | 0.8810296 |
| TRINITY_DN23746_c0_g1_i3 | blue | 0.8808778 |
| TRINITY_DN21960_c0_g1_i1 | blue | 0.8808105 |
| TRINITY_DN28083_c0_g1_i1 | blue | 0.8807697 |
| TRINITY_DN38015_c0_g1_i3 | blue | 0.8807017 |
| TRINITY_DN27826_c0_g1_i1 | blue | 0.880665 |
| TRINITY_DN38444_c3_g1_i1 | blue | 0.8806436 |
| TRINITY_DN37018_c0_g2_i3 | blue | 0.8806436 |
| TRINITY_DN31296_c0_g1_i2 | blue | 0.8806321 |
| TRINITY_DN31785_c0_g1_i1 | blue | 0.8806303 |
| TRINITY_DN36754_c0_g3_i10 | blue | 0.8804547 |
| TRINITY_DN38799_c0_g2_i4 | blue | 0.8802859 |
| TRINITY_DN23346_c0_g2_i1 | blue | 0.8802668 |
| TRINITY_DN40269_c0_g1_i1 | blue | 0.8799598 |
| TRINITY_DN32120_c1_g1_i2 | blue | 0.8799153 |
| TRINITY_DN38273_c0_g1_i2 | blue | 0.8798491 |
| TRINITY_DN27838_c0_g2_i2 | blue | 0.8798083 |
| TRINITY_DN36526_c0_g7_i11 | blue | 0.8797284 |
| TRINITY_DN34084_c1_g2_i1 | blue | 0.8795215 |
| TRINITY_DN36248_c0_g1_i1 | blue | 0.8794268 |
| TRINITY_DN38981_c0_g1_i1 | blue | 0.8792709 |
| TRINITY_DN36266_c0_g3_i3 | blue | 0.8790938 |
| TRINITY_DN33953_c0_g1_i1 | blue | 0.8790621 |
| TRINITY_DN28216_c0_g2_i1 | blue | 0.8788835 |
| TRINITY_DN32833_c2_g4_i1 | blue | 0.8787047 |
| TRINITY_DN24397_c0_g2_i1 | blue | 0.878678 |
| TRINITY_DN35762_c1_g10_i7 | blue | 0.8786677 |
| TRINITY_DN25873_c0_g1_i1 | blue | 0.8785016 |
| TRINITY_DN38731_c4_g8_i1 | blue | 0.8782917 |
| TRINITY_DN22819_c0_g1_i1 | blue | 0.8781994 |
| TRINITY_DN32006_c0_g2_i2 | blue | 0.877307 |
| TRINITY_DN26065_c0_g1_i1 | blue | 0.8768155 |
| TRINITY_DN24845_c0_g1_i1 | blue | 0.8764357 |
| TRINITY_DN27779_c0_g1_i3 | blue | 0.8763787 |
| TRINITY_DN27261_c0_g1_i1 | blue | 0.8761756 |
| TRINITY_DN37515_c1_g1_i11 | blue | 0.8761481 |
| TRINITY_DN39336_c14_g7_i2 | blue | 0.8761454 |
| TRINITY_DN38872_c0_g1_i2 | blue | 0.8760189 |
| TRINITY_DN37485_c0_g2_i1 | blue | 0.8759617 |
| TRINITY_DN33077_c0_g1_i1 | blue | 0.8756512 |
| TRINITY_DN25187_c0_g2_i1 | blue | 0.8755736 |
| TRINITY_DN39160_c0_g1_i4 | blue | 0.8754809 |
| TRINITY_DN33304_c0_g1_i4 | blue | 0.8753614 |
| TRINITY_DN32206_c0_g1_i1 | blue | 0.8752935 |
| TRINITY_DN36966_c0_g2_i4 | blue | 0.8745711 |
| TRINITY_DN22771_c1_g3_i1 | blue | 0.8743668 |
| TRINITY_DN36155_c0_g1_i1 | blue | 0.8741251 |
| TRINITY_DN38171_c0_g2_i1 | blue | 0.873843 |
| TRINITY_DN24955_c0_g2_i1 | blue | 0.8738092 |
| TRINITY_DN36672_c0_g2_i1 | blue | 0.873798 |
| TRINITY_DN10292_c0_g1_i1 | blue | 0.8732137 |
| TRINITY_DN61735_c0_g1_i1 | blue | 0.8731867 |
| TRINITY_DN38365_c1_g1_i1 | blue | 0.8731381 |
| TRINITY_DN34647_c0_g3_i1 | blue | 0.8728513 |
| TRINITY_DN35486_c0_g1_i1 | blue | 0.8726491 |
| TRINITY_DN33594_c0_g1_i5 | blue | 0.8724533 |
| TRINITY_DN29173_c0_g2_i2 | blue | 0.8723891 |
| TRINITY_DN33039_c0_g1_i6 | blue | 0.8721222 |
| TRINITY_DN28379_c0_g1_i2 | blue | 0.8720769 |
| TRINITY_DN33385_c0_g2_i1 | blue | 0.8719829 |
| TRINITY_DN24081_c0_g1_i1 | blue | 0.8718979 |
| TRINITY_DN8650_c0_g1_i1 | blue | 0.8716763 |
| TRINITY_DN36564_c0_g3_i1 | blue | 0.8714083 |
| TRINITY_DN36484_c0_g2_i2 | blue | 0.8712615 |
| TRINITY_DN27757_c0_g1_i1 | blue | 0.8708061 |
| TRINITY_DN39077_c0_g2_i3 | blue | 0.8700665 |
| TRINITY_DN36648_c1_g2_i2 | blue | 0.8700051 |
| TRINITY_DN26493_c0_g1_i2 | blue | 0.8699818 |
| TRINITY_DN29883_c0_g1_i1 | blue | 0.8699297 |
| TRINITY_DN25451_c0_g1_i1 | blue | 0.86965 |
| TRINITY_DN27237_c0_g1_i2 | blue | 0.8696311 |
| TRINITY_DN29636_c0_g1_i2 | blue | 0.869452 |
| TRINITY_DN27089_c0_g1_i1 | blue | 0.8694325 |
| TRINITY_DN25329_c0_g5_i1 | blue | 0.8694007 |
| TRINITY_DN36028_c2_g1_i1 | blue | 0.869375 |
| TRINITY_DN26884_c0_g1_i1 | blue | 0.869244 |
| TRINITY_DN32024_c0_g2_i2 | blue | 0.8691511 |
| TRINITY_DN31824_c0_g4_i2 | blue | 0.8690282 |
| TRINITY_DN39023_c0_g5_i2 | blue | 0.8686942 |
| TRINITY_DN24377_c0_g1_i1 | blue | 0.8686812 |
| TRINITY_DN39337_c3_g6_i1 | blue | 0.868585 |
| TRINITY_DN25660_c0_g1_i1 | blue | 0.8685213 |
| TRINITY_DN32173_c0_g1_i1 | blue | 0.8682606 |
| TRINITY_DN35278_c2_g1_i2 | blue | 0.8676684 |
| TRINITY_DN32501_c0_g2_i1 | blue | 0.8673961 |
| TRINITY_DN29416_c0_g3_i1 | blue | 0.8663415 |
| TRINITY_DN46237_c0_g1_i1 | blue | 0.8663367 |
| TRINITY_DN28059_c0_g1_i1 | blue | 0.8662594 |
| TRINITY_DN37041_c0_g2_i1 | blue | 0.8662452 |
| TRINITY_DN11625_c0_g1_i1 | blue | 0.8657878 |
| TRINITY_DN33649_c0_g2_i1 | blue | 0.8655043 |
| TRINITY_DN22419_c0_g1_i1 | blue | 0.8654938 |
| TRINITY_DN28613_c1_g1_i3 | blue | 0.8654394 |
| TRINITY_DN38005_c1_g1_i1 | blue | 0.8648492 |
| TRINITY_DN30689_c0_g2_i1 | blue | 0.8648382 |
| TRINITY_DN31130_c0_g1_i2 | blue | 0.8646768 |
| TRINITY_DN36333_c1_g2_i1 | blue | 0.8644776 |
| TRINITY_DN32470_c1_g3_i1 | blue | 0.8642922 |
| TRINITY_DN35261_c0_g3_i1 | blue | 0.8640712 |
| TRINITY_DN36527_c0_g3_i10 | blue | 0.8636184 |
| TRINITY_DN38532_c0_g1_i1 | blue | 0.863487 |
| TRINITY_DN31573_c2_g1_i1 | blue | 0.8630957 |
| TRINITY_DN25329_c0_g8_i1 | blue | 0.863086 |
| TRINITY_DN36178_c0_g12_i1 | blue | 0.8630809 |
| TRINITY_DN28015_c0_g1_i1 | blue | 0.8624713 |
| TRINITY_DN29947_c0_g2_i1 | blue | 0.8623848 |
| TRINITY_DN35908_c0_g1_i1 | blue | 0.862264 |
| TRINITY_DN32981_c1_g1_i8 | blue | 0.8619743 |
| TRINITY_DN36002_c0_g9_i1 | blue | 0.8616274 |
| TRINITY_DN4862_c0_g2_i1 | blue | 0.8616022 |
| TRINITY_DN37314_c1_g3_i1 | blue | 0.8605525 |
| TRINITY_DN32523_c1_g1_i5 | blue | 0.860493 |
| TRINITY_DN35614_c0_g1_i2 | blue | 0.8604519 |
| TRINITY_DN16600_c0_g2_i1 | blue | 0.8602073 |
| TRINITY_DN32772_c0_g2_i1 | blue | 0.860159 |
| TRINITY_DN27024_c0_g1_i2 | blue | 0.8599527 |
| TRINITY_DN24785_c0_g1_i2 | blue | 0.8598284 |
| TRINITY_DN32783_c1_g2_i2 | blue | 0.8597996 |
| TRINITY_DN27494_c1_g1_i1 | blue | 0.8597853 |
| TRINITY_DN38731_c4_g3_i1 | blue | 0.8594668 |
| TRINITY_DN21084_c0_g2_i1 | blue | 0.859465 |
| TRINITY_DN31835_c0_g4_i2 | blue | 0.8593027 |
| TRINITY_DN31234_c0_g1_i1 | blue | 0.8592974 |
| TRINITY_DN27101_c0_g1_i1 | blue | 0.8589644 |
| TRINITY_DN27038_c0_g2_i2 | blue | 0.858957 |
| TRINITY_DN32904_c0_g1_i1 | blue | 0.8589281 |
| TRINITY_DN35846_c1_g5_i2 | blue | 0.8583169 |
| TRINITY_DN36705_c2_g1_i9 | blue | 0.8577663 |
| TRINITY_DN32595_c1_g2_i3 | blue | 0.8575761 |
| TRINITY_DN28362_c0_g1_i2 | blue | 0.8572566 |
| TRINITY_DN35185_c0_g7_i1 | blue | 0.8571712 |
| TRINITY_DN36407_c1_g5_i1 | blue | 0.857124 |
| TRINITY_DN35592_c0_g2_i1 | blue | 0.8571041 |
| TRINITY_DN26653_c0_g1_i1 | blue | 0.8565163 |
| TRINITY_DN33928_c1_g1_i1 | blue | 0.856372 |
| TRINITY_DN34630_c0_g2_i4 | blue | 0.8562332 |
| TRINITY_DN25951_c0_g1_i1 | blue | 0.8558916 |
| TRINITY_DN33817_c1_g2_i1 | blue | 0.8556737 |
| TRINITY_DN32548_c0_g1_i3 | blue | 0.8555299 |
| TRINITY_DN30500_c0_g1_i1 | blue | 0.8554921 |
| TRINITY_DN33966_c0_g1_i4 | blue | 0.8553755 |
| TRINITY_DN38397_c0_g5_i10 | blue | 0.8553287 |
| TRINITY_DN38088_c0_g1_i1 | blue | 0.8550539 |
| TRINITY_DN35528_c0_g1_i1 | blue | 0.8549476 |
| TRINITY_DN30917_c0_g6_i1 | blue | 0.8542974 |
| TRINITY_DN12002_c0_g1_i1 | blue | 0.8541165 |
| TRINITY_DN36936_c0_g3_i1 | blue | 0.8539979 |
| TRINITY_DN34663_c0_g3_i2 | blue | 0.8536355 |
| TRINITY_DN28445_c0_g1_i1 | blue | 0.8527743 |
| TRINITY_DN34349_c0_g1_i1 | blue | 0.8527272 |
| TRINITY_DN7699_c0_g2_i1 | blue | 0.8525766 |
| TRINITY_DN17116_c0_g2_i1 | blue | 0.8524578 |
| TRINITY_DN36532_c1_g2_i2 | blue | 0.852379 |
| TRINITY_DN32173_c0_g10_i4 | blue | 0.8523497 |
| TRINITY_DN36953_c1_g2_i1 | blue | 0.8519793 |
| TRINITY_DN27542_c0_g1_i1 | blue | 0.8519072 |
| TRINITY_DN21807_c0_g1_i1 | blue | 0.851819 |
| TRINITY_DN31578_c0_g1_i1 | blue | 0.8517672 |
| TRINITY_DN29272_c1_g1_i2 | blue | 0.8516458 |
| TRINITY_DN32874_c0_g2_i1 | blue | 0.8512934 |
| TRINITY_DN22821_c0_g1_i1 | blue | 0.8511876 |
| TRINITY_DN28179_c0_g1_i1 | blue | 0.8510028 |
| TRINITY_DN37079_c0_g2_i1 | blue | 0.8509261 |
| TRINITY_DN25682_c0_g1_i1 | blue | 0.8505963 |
| TRINITY_DN16810_c0_g1_i1 | blue | 0.8505007 |
| TRINITY_DN36976_c0_g1_i1 | blue | 0.8503242 |
| TRINITY_DN39237_c4_g5_i2 | blue | 0.8502988 |
| TRINITY_DN35497_c0_g3_i1 | blue | 0.8501079 |
| TRINITY_DN32519_c2_g2_i1 | blue | 0.8495269 |
| TRINITY_DN22164_c0_g1_i1 | blue | 0.8491165 |
| TRINITY_DN38406_c0_g5_i1 | blue | 0.8488759 |
| TRINITY_DN18730_c0_g1_i1 | blue | 0.8484322 |
| TRINITY_DN34833_c0_g1_i1 | blue | 0.8479424 |
| TRINITY_DN38985_c1_g5_i1 | blue | 0.8475549 |
| TRINITY_DN30655_c0_g1_i5 | blue | 0.847508 |
| TRINITY_DN22373_c0_g1_i3 | blue | 0.8474457 |
| TRINITY_DN38445_c3_g3_i3 | blue | 0.8473795 |
| TRINITY_DN45308_c0_g1_i1 | blue | 0.8472486 |
| TRINITY_DN27665_c0_g1_i1 | blue | 0.8471203 |
| TRINITY_DN28390_c0_g2_i1 | blue | 0.8470361 |
| TRINITY_DN30439_c0_g1_i2 | blue | 0.8469065 |
| TRINITY_DN39329_c14_g9_i2 | blue | 0.8467526 |
| TRINITY_DN24556_c0_g1_i3 | blue | 0.8464444 |
| TRINITY_DN30453_c0_g1_i2 | blue | 0.8462474 |
| TRINITY_DN37614_c1_g5_i2 | blue | 0.8460048 |
| TRINITY_DN27572_c0_g2_i1 | blue | 0.8456839 |
| TRINITY_DN38577_c1_g1_i1 | blue | 0.8454271 |
| TRINITY_DN36888_c0_g2_i1 | blue | 0.8452657 |
| TRINITY_DN28745_c0_g1_i1 | blue | 0.8452279 |
| TRINITY_DN35141_c0_g1_i4 | blue | 0.845073 |
| TRINITY_DN33044_c0_g3_i2 | blue | 0.8448513 |
| TRINITY_DN34180_c0_g4_i1 | blue | 0.8447517 |
| TRINITY_DN28965_c0_g1_i1 | blue | 0.8445994 |
| TRINITY_DN26076_c1_g2_i1 | blue | 0.844482 |
| TRINITY_DN20981_c0_g1_i1 | blue | 0.8444219 |
| TRINITY_DN23942_c0_g1_i1 | blue | 0.8441564 |
| TRINITY_DN39220_c6_g1_i1 | blue | 0.8441326 |
| TRINITY_DN37525_c3_g1_i6 | blue | 0.8438303 |
| TRINITY_DN35203_c0_g2_i2 | blue | 0.8434959 |
| TRINITY_DN35045_c0_g1_i1 | blue | 0.8432675 |
| TRINITY_DN33943_c0_g2_i1 | blue | 0.8432148 |
| TRINITY_DN24299_c0_g1_i1 | blue | 0.8431882 |
| TRINITY_DN35107_c0_g1_i1 | blue | 0.8431869 |
| TRINITY_DN25707_c0_g2_i1 | blue | 0.8430329 |
| TRINITY_DN29150_c0_g1_i1 | blue | 0.842945 |
| TRINITY_DN31674_c0_g4_i1 | blue | 0.8428955 |
| TRINITY_DN20534_c0_g2_i1 | blue | 0.8428462 |
| TRINITY_DN25466_c0_g1_i1 | blue | 0.8427553 |
| TRINITY_DN33046_c0_g1_i1 | blue | 0.8424729 |
| TRINITY_DN38234_c0_g2_i1 | blue | 0.8423362 |
| TRINITY_DN5539_c0_g1_i1 | blue | 0.8423151 |
| TRINITY_DN33971_c1_g1_i2 | blue | 0.8420928 |
| TRINITY_DN31300_c0_g1_i1 | blue | 0.8418869 |
| TRINITY_DN36576_c0_g3_i3 | blue | 0.8417051 |
| TRINITY_DN38442_c7_g13_i1 | blue | 0.8416729 |
| TRINITY_DN25509_c0_g2_i3 | blue | 0.8415955 |
| TRINITY_DN25733_c0_g4_i1 | blue | 0.841429 |
| TRINITY_DN32554_c0_g1_i1 | blue | 0.8414088 |
| TRINITY_DN22682_c0_g1_i1 | blue | 0.8408927 |
| TRINITY_DN13471_c0_g1_i1 | blue | 0.8408113 |
| TRINITY_DN39144_c2_g14_i1 | blue | 0.8401809 |
| TRINITY_DN35584_c1_g1_i1 | blue | 0.84 |
| TRINITY_DN19069_c0_g1_i1 | blue | 0.8395748 |
| TRINITY_DN39238_c1_g9_i3 | blue | 0.8393722 |
| TRINITY_DN39251_c0_g2_i2 | blue | 0.8392721 |
| TRINITY_DN37840_c0_g1_i1 | blue | 0.8391492 |
| TRINITY_DN34725_c0_g2_i1 | blue | 0.8390609 |
| TRINITY_DN28130_c0_g1_i4 | blue | 0.8389263 |
| TRINITY_DN30457_c0_g2_i1 | blue | 0.8384884 |
| TRINITY_DN36683_c0_g1_i8 | blue | 0.8383832 |
| TRINITY_DN22257_c0_g2_i1 | blue | 0.8380185 |
| TRINITY_DN36148_c1_g2_i8 | blue | 0.836946 |
| TRINITY_DN37088_c0_g1_i2 | blue | 0.8368853 |
| TRINITY_DN32328_c0_g1_i4 | blue | 0.836744 |
| TRINITY_DN21904_c0_g1_i1 | blue | 0.8367091 |
| TRINITY_DN27491_c0_g2_i4 | blue | 0.8366764 |
| TRINITY_DN25131_c0_g1_i1 | blue | 0.8366336 |
| TRINITY_DN37180_c0_g1_i1 | blue | 0.8365202 |
| TRINITY_DN34143_c1_g2_i1 | blue | 0.8365173 |
| TRINITY_DN34689_c1_g1_i3 | blue | 0.8363557 |
| TRINITY_DN28229_c0_g10_i2 | blue | 0.8356004 |
| TRINITY_DN37765_c0_g1_i2 | blue | 0.8350593 |
| TRINITY_DN25961_c0_g3_i2 | blue | 0.8350488 |
| TRINITY_DN31093_c0_g1_i1 | blue | 0.835029 |
| TRINITY_DN35612_c0_g2_i1 | blue | 0.8348569 |
| TRINITY_DN32242_c0_g2_i3 | blue | 0.8346334 |
| TRINITY_DN21043_c0_g1_i1 | blue | 0.8343567 |
| TRINITY_DN30787_c0_g1_i1 | blue | 0.8342242 |
| TRINITY_DN37353_c1_g1_i1 | blue | 0.8334236 |
| TRINITY_DN38365_c1_g2_i1 | blue | 0.8333597 |
| TRINITY_DN29979_c0_g1_i2 | blue | 0.8333283 |
| TRINITY_DN22858_c0_g1_i1 | blue | 0.833076 |
| TRINITY_DN32622_c0_g2_i3 | blue | 0.8329893 |
| TRINITY_DN18799_c0_g1_i1 | blue | 0.8322522 |
| TRINITY_DN22007_c0_g2_i1 | blue | 0.8320728 |
| TRINITY_DN15769_c0_g1_i1 | blue | 0.8314039 |
| TRINITY_DN38554_c0_g3_i2 | blue | 0.8312608 |
| TRINITY_DN27853_c0_g1_i1 | blue | 0.830933 |
| TRINITY_DN35194_c0_g1_i4 | blue | 0.8309186 |
| TRINITY_DN36039_c1_g7_i1 | blue | 0.8308821 |
| TRINITY_DN34147_c0_g2_i5 | blue | 0.8307013 |
| TRINITY_DN22527_c1_g1_i1 | blue | 0.8303898 |
| TRINITY_DN37892_c1_g3_i1 | blue | 0.8303299 |
| TRINITY_DN35137_c2_g1_i2 | blue | 0.8302285 |
| TRINITY_DN69722_c0_g1_i1 | blue | 0.8300119 |
| TRINITY_DN20631_c0_g1_i1 | blue | 0.8296083 |
| TRINITY_DN25543_c0_g1_i1 | blue | 0.8293988 |
| TRINITY_DN28155_c0_g3_i1 | blue | 0.8292391 |
| TRINITY_DN27552_c0_g1_i1 | blue | 0.8291542 |
| TRINITY_DN36356_c0_g2_i2 | blue | 0.828375 |
| TRINITY_DN35211_c0_g2_i3 | blue | 0.8272064 |
| TRINITY_DN26099_c0_g1_i1 | blue | 0.825868 |
| TRINITY_DN33211_c3_g4_i1 | blue | 0.8257267 |
| TRINITY_DN35429_c0_g1_i5 | blue | 0.8255287 |
| TRINITY_DN20253_c0_g1_i1 | blue | 0.8247743 |
| TRINITY_DN45037_c0_g1_i1 | blue | 0.8246171 |
| TRINITY_DN37262_c0_g7_i1 | blue | 0.8242089 |
| TRINITY_DN24154_c0_g1_i1 | blue | 0.8241005 |
| TRINITY_DN23157_c0_g3_i1 | blue | 0.8241004 |
| TRINITY_DN37644_c1_g1_i5 | blue | 0.8239818 |
| TRINITY_DN30829_c0_g1_i1 | blue | 0.8235691 |
| TRINITY_DN36339_c2_g2_i3 | blue | 0.8234848 |
| TRINITY_DN8043_c0_g1_i1 | blue | 0.8229688 |
| TRINITY_DN31093_c0_g2_i1 | blue | 0.8224137 |
| TRINITY_DN36787_c1_g4_i1 | blue | 0.8223284 |
| TRINITY_DN20038_c0_g2_i1 | blue | 0.8221016 |
| TRINITY_DN35827_c0_g3_i2 | blue | 0.8215932 |
| TRINITY_DN23223_c0_g2_i1 | blue | 0.8214236 |
| TRINITY_DN22527_c1_g4_i1 | blue | 0.821137 |
| TRINITY_DN39314_c3_g3_i1 | blue | 0.8208313 |
| TRINITY_DN37307_c0_g3_i1 | blue | 0.8207573 |
| TRINITY_DN16559_c0_g1_i1 | blue | 0.8205067 |
| TRINITY_DN32822_c0_g2_i3 | blue | 0.8195208 |
| TRINITY_DN34801_c1_g5_i6 | blue | 0.8194317 |
| TRINITY_DN29258_c0_g2_i3 | blue | 0.8193474 |
| TRINITY_DN25397_c0_g1_i1 | blue | 0.8192753 |
| TRINITY_DN20035_c0_g1_i1 | blue | 0.8191498 |
| TRINITY_DN27525_c0_g1_i2 | blue | 0.8187405 |
| TRINITY_DN20567_c0_g1_i1 | blue | 0.818451 |
| TRINITY_DN33015_c0_g1_i3 | blue | 0.8182281 |
| TRINITY_DN27515_c0_g1_i1 | blue | 0.8179633 |
| TRINITY_DN35592_c0_g3_i2 | blue | 0.8176636 |
| TRINITY_DN29101_c0_g1_i3 | blue | 0.8173615 |
| TRINITY_DN30347_c1_g3_i6 | blue | 0.8171302 |
| TRINITY_DN26927_c0_g1_i5 | blue | 0.8171239 |
| TRINITY_DN35198_c0_g8_i1 | blue | 0.8162034 |
| TRINITY_DN29072_c0_g1_i1 | blue | 0.8158865 |
| TRINITY_DN26606_c0_g1_i1 | blue | 0.8153795 |
| TRINITY_DN33030_c0_g1_i2 | blue | 0.8148683 |
| TRINITY_DN39148_c0_g2_i1 | blue | 0.8132067 |
| TRINITY_DN31689_c0_g1_i1 | blue | 0.8130653 |
| TRINITY_DN36413_c0_g4_i9 | blue | 0.8127933 |
| TRINITY_DN33248_c0_g1_i1 | blue | 0.812741 |
| TRINITY_DN37772_c0_g1_i4 | blue | 0.8127357 |
| TRINITY_DN37488_c0_g3_i1 | blue | 0.8125808 |
| TRINITY_DN39023_c0_g6_i1 | blue | 0.8118605 |
| TRINITY_DN38339_c1_g5_i8 | blue | 0.811803 |
| TRINITY_DN31744_c0_g1_i2 | blue | 0.8110294 |
| TRINITY_DN37551_c0_g1_i1 | blue | 0.8109966 |
| TRINITY_DN36465_c0_g4_i2 | blue | 0.810636 |
| TRINITY_DN27767_c0_g1_i1 | blue | 0.810339 |
| TRINITY_DN33882_c0_g2_i7 | blue | 0.8099668 |
| TRINITY_DN61302_c0_g1_i1 | blue | 0.8088381 |
| TRINITY_DN38566_c0_g2_i1 | blue | 0.8088291 |
| TRINITY_DN37709_c1_g1_i5 | blue | 0.8087945 |
| TRINITY_DN20970_c0_g1_i1 | blue | 0.8087544 |
| TRINITY_DN64609_c0_g1_i1 | blue | 0.8081812 |
| TRINITY_DN36032_c0_g1_i3 | blue | 0.8080583 |
| TRINITY_DN24434_c0_g1_i1 | blue | 0.80802 |
| TRINITY_DN37152_c0_g5_i1 | blue | 0.8078617 |
| TRINITY_DN35786_c2_g2_i1 | blue | 0.8073687 |
| TRINITY_DN28734_c0_g2_i16 | blue | 0.807041 |
| TRINITY_DN46346_c0_g1_i1 | blue | 0.806773 |
| TRINITY_DN25818_c0_g1_i2 | blue | 0.8064958 |
| TRINITY_DN29302_c0_g1_i1 | blue | 0.8057894 |
| TRINITY_DN30601_c0_g1_i2 | blue | 0.8057515 |
| TRINITY_DN39129_c2_g2_i4 | blue | 0.8054037 |
| TRINITY_DN34939_c0_g3_i1 | blue | 0.805193 |
| TRINITY_DN26230_c0_g1_i1 | blue | 0.8051157 |
| TRINITY_DN25570_c0_g3_i2 | blue | 0.8046982 |
| TRINITY_DN28996_c0_g1_i2 | blue | 0.8046476 |
| TRINITY_DN24452_c0_g1_i1 | blue | 0.8045032 |
| TRINITY_DN20885_c0_g2_i1 | blue | 0.8037023 |
| TRINITY_DN16167_c0_g1_i1 | blue | 0.8037009 |
| TRINITY_DN32023_c0_g1_i7 | blue | 0.8032276 |
| TRINITY_DN38503_c1_g1_i2 | blue | 0.803163 |
| TRINITY_DN38980_c1_g6_i9 | blue | 0.8031104 |
| TRINITY_DN22801_c0_g1_i1 | blue | 0.8030977 |
| TRINITY_DN30509_c0_g1_i3 | blue | 0.8027732 |
| TRINITY_DN25029_c0_g1_i1 | blue | 0.8022902 |
| TRINITY_DN21303_c0_g1_i1 | blue | 0.8022704 |
| TRINITY_DN34625_c0_g1_i1 | blue | 0.8021421 |
| TRINITY_DN26781_c0_g1_i3 | blue | 0.8019257 |
| TRINITY_DN27143_c0_g1_i1 | blue | 0.8019235 |
| TRINITY_DN40190_c0_g1_i1 | blue | 0.8018748 |
| TRINITY_DN31340_c0_g1_i2 | blue | 0.8017256 |
| TRINITY_DN37667_c1_g1_i7 | blue | 0.8015749 |
| TRINITY_DN26006_c0_g1_i1 | blue | 0.7995911 |
| TRINITY_DN24183_c0_g1_i2 | blue | 0.7993465 |
| TRINITY_DN19789_c0_g2_i1 | blue | 0.7992965 |
| TRINITY_DN39082_c1_g10_i2 | blue | 0.7976035 |
| TRINITY_DN29924_c0_g1_i2 | blue | 0.7974436 |
| TRINITY_DN33119_c0_g1_i6 | blue | 0.7970107 |
| TRINITY_DN25013_c0_g1_i1 | blue | 0.7969091 |
| TRINITY_DN23593_c0_g1_i1 | blue | 0.7968038 |
| TRINITY_DN20815_c0_g1_i1 | blue | 0.7959584 |
| TRINITY_DN21863_c0_g1_i1 | blue | 0.7955274 |
| TRINITY_DN18172_c0_g1_i1 | blue | 0.7953363 |
| TRINITY_DN37650_c2_g2_i1 | blue | 0.7951602 |
| TRINITY_DN26848_c0_g1_i1 | blue | 0.7942522 |
| TRINITY_DN22975_c0_g1_i1 | blue | 0.7941346 |
| TRINITY_DN27220_c0_g2_i1 | blue | 0.794085 |
| TRINITY_DN34536_c1_g3_i3 | blue | 0.7938735 |
| TRINITY_DN26913_c1_g1_i1 | blue | 0.7935564 |
| TRINITY_DN27461_c2_g3_i1 | blue | 0.7933311 |
| TRINITY_DN36700_c0_g1_i4 | blue | 0.7925595 |
| TRINITY_DN33413_c0_g1_i2 | blue | 0.7923493 |
| TRINITY_DN34888_c0_g2_i2 | blue | 0.792339 |
| TRINITY_DN37791_c2_g6_i1 | blue | 0.7921446 |
| TRINITY_DN23797_c0_g1_i2 | blue | 0.7919394 |
| TRINITY_DN25850_c0_g1_i1 | blue | 0.7917925 |
| TRINITY_DN35995_c0_g1_i5 | blue | 0.7913788 |
| TRINITY_DN32892_c1_g1_i1 | blue | 0.7913618 |
| TRINITY_DN37973_c0_g1_i1 | blue | 0.791139 |
| TRINITY_DN35052_c0_g2_i9 | blue | 0.7903078 |
| TRINITY_DN31289_c0_g1_i1 | blue | 0.7896542 |
| TRINITY_DN35382_c0_g1_i2 | blue | 0.7894042 |
| TRINITY_DN21825_c0_g3_i1 | blue | 0.7892744 |
| TRINITY_DN36255_c0_g1_i2 | blue | 0.7890456 |
| TRINITY_DN37522_c0_g3_i3 | blue | 0.7889771 |
| TRINITY_DN63022_c0_g1_i1 | blue | 0.7888995 |
| TRINITY_DN25807_c0_g1_i1 | blue | 0.7882091 |
| TRINITY_DN29016_c0_g1_i5 | blue | 0.7879618 |
| TRINITY_DN20927_c0_g1_i1 | blue | 0.7878539 |
| TRINITY_DN27467_c0_g1_i2 | blue | 0.7872535 |
| TRINITY_DN22444_c0_g1_i1 | blue | 0.7872437 |
| TRINITY_DN19534_c0_g3_i1 | blue | 0.7864923 |
| TRINITY_DN34827_c1_g6_i1 | blue | 0.786352 |
| TRINITY_DN27692_c0_g1_i1 | blue | 0.7860209 |
| TRINITY_DN35122_c0_g1_i3 | blue | 0.7851954 |
| TRINITY_DN32337_c0_g1_i2 | blue | 0.7851783 |
| TRINITY_DN30912_c0_g1_i10 | blue | 0.784325 |
| TRINITY_DN18574_c0_g1_i1 | blue | 0.7834954 |
| TRINITY_DN26735_c0_g3_i2 | blue | 0.7825874 |
| TRINITY_DN28001_c0_g1_i1 | blue | 0.7823477 |
| TRINITY_DN38599_c2_g5_i8 | blue | 0.7821386 |
| TRINITY_DN38601_c2_g3_i1 | blue | 0.7819873 |
| TRINITY_DN28353_c0_g1_i1 | blue | 0.781654 |
| TRINITY_DN33214_c3_g1_i8 | blue | 0.7807214 |
| TRINITY_DN38613_c2_g1_i2 | blue | 0.780226 |
| TRINITY_DN37636_c0_g1_i4 | blue | 0.7797175 |
| TRINITY_DN27181_c0_g2_i1 | blue | 0.7790918 |
| TRINITY_DN24824_c0_g1_i1 | blue | 0.7785819 |
| TRINITY_DN36849_c1_g1_i3 | blue | 0.7775437 |
| TRINITY_DN29394_c0_g1_i4 | blue | 0.7772052 |
| TRINITY_DN11183_c0_g1_i1 | blue | 0.7772015 |
| TRINITY_DN37594_c1_g4_i1 | blue | 0.777007 |
| TRINITY_DN22773_c0_g2_i1 | blue | 0.7768421 |
| TRINITY_DN37445_c0_g3_i3 | blue | 0.7764011 |
| TRINITY_DN3153_c0_g1_i1 | blue | 0.7763267 |
| TRINITY_DN34707_c0_g1_i1 | blue | 0.7758143 |
| TRINITY_DN28958_c0_g2_i1 | blue | 0.7752683 |
| TRINITY_DN26583_c0_g4_i1 | blue | 0.7751675 |
| TRINITY_DN23359_c0_g1_i1 | blue | 0.7749705 |
| TRINITY_DN24989_c0_g1_i1 | blue | 0.7745762 |
| TRINITY_DN22641_c0_g1_i1 | blue | 0.774402 |
| TRINITY_DN38176_c1_g12_i2 | blue | 0.7743236 |
| TRINITY_DN26002_c0_g1_i1 | blue | 0.7739706 |
| TRINITY_DN15392_c0_g2_i1 | blue | 0.7739146 |
| TRINITY_DN30822_c1_g1_i5 | blue | 0.7732477 |
| TRINITY_DN29685_c2_g2_i7 | blue | 0.773065 |
| TRINITY_DN28212_c0_g1_i1 | blue | 0.7728991 |
| TRINITY_DN12806_c0_g1_i1 | blue | 0.7728844 |
| TRINITY_DN37973_c1_g2_i1 | blue | 0.7726093 |
| TRINITY_DN9916_c0_g1_i1 | blue | 0.772202 |
| TRINITY_DN7673_c0_g1_i1 | blue | 0.7714187 |
| TRINITY_DN4482_c0_g2_i1 | blue | 0.7712863 |
| TRINITY_DN26165_c0_g1_i2 | blue | 0.7712712 |
| TRINITY_DN38908_c1_g2_i3 | blue | 0.7711336 |
| TRINITY_DN23621_c0_g1_i1 | blue | 0.7705612 |
| TRINITY_DN12596_c0_g1_i1 | blue | 0.7704064 |
| TRINITY_DN32150_c1_g1_i1 | blue | 0.7698421 |
| TRINITY_DN9150_c0_g1_i1 | blue | 0.7694029 |
| TRINITY_DN21995_c0_g1_i1 | blue | 0.7691882 |
| TRINITY_DN31546_c0_g1_i1 | blue | 0.769041 |
| TRINITY_DN624_c0_g1_i1 | blue | 0.7679683 |
| TRINITY_DN9261_c0_g1_i1 | blue | 0.7662983 |
| TRINITY_DN25210_c0_g2_i1 | blue | 0.7660015 |
| TRINITY_DN38936_c0_g9_i1 | blue | 0.7656466 |
| TRINITY_DN37469_c1_g3_i1 | blue | 0.7655387 |
| TRINITY_DN1911_c0_g1_i1 | blue | 0.7651105 |
| TRINITY_DN23178_c0_g3_i1 | blue | 0.7644085 |
| TRINITY_DN46108_c0_g1_i1 | blue | 0.7643909 |
| TRINITY_DN16667_c0_g4_i1 | blue | 0.7640959 |
| TRINITY_DN37234_c2_g1_i1 | blue | 0.7638431 |
| TRINITY_DN34376_c0_g1_i2 | blue | 0.7638195 |
| TRINITY_DN67410_c0_g1_i1 | blue | 0.7636746 |
| TRINITY_DN19039_c0_g1_i1 | blue | 0.7636091 |
| TRINITY_DN34842_c0_g2_i1 | blue | 0.7634603 |
| TRINITY_DN30378_c0_g1_i1 | blue | 0.7632541 |
| TRINITY_DN25172_c0_g3_i1 | blue | 0.7628427 |
| TRINITY_DN30711_c2_g1_i1 | blue | 0.7627459 |
| TRINITY_DN39311_c5_g4_i3 | blue | 0.7621436 |
| TRINITY_DN22118_c0_g3_i1 | blue | 0.7618706 |
| TRINITY_DN14417_c0_g1_i1 | blue | 0.7616 |
| TRINITY_DN38737_c0_g1_i1 | blue | 0.7614872 |
| TRINITY_DN26616_c0_g2_i1 | blue | 0.7614113 |
| TRINITY_DN30671_c0_g7_i1 | blue | 0.7605382 |
| TRINITY_DN23309_c0_g1_i2 | blue | 0.7605073 |
| TRINITY_DN23197_c0_g1_i1 | blue | 0.7602118 |
| TRINITY_DN32898_c0_g1_i4 | blue | 0.7601762 |
| TRINITY_DN32059_c1_g2_i3 | blue | 0.7601198 |
| TRINITY_DN39217_c0_g1_i1 | blue | 0.7584526 |
| TRINITY_DN14223_c0_g2_i1 | blue | 0.7582376 |
| TRINITY_DN30249_c0_g1_i1 | blue | 0.7581997 |
| TRINITY_DN33611_c2_g3_i4 | blue | 0.758181 |
| TRINITY_DN26346_c0_g1_i1 | blue | 0.7568468 |
| TRINITY_DN20803_c0_g1_i1 | blue | 0.7565778 |
| TRINITY_DN24548_c0_g1_i1 | blue | 0.7564835 |
| TRINITY_DN16936_c0_g1_i1 | blue | 0.7561315 |
| TRINITY_DN19842_c0_g2_i1 | blue | 0.7545994 |
| TRINITY_DN25599_c0_g2_i1 | blue | 0.7545797 |
| TRINITY_DN35517_c0_g3_i2 | blue | 0.7543997 |
| TRINITY_DN38013_c2_g3_i1 | blue | 0.7540154 |
| TRINITY_DN25863_c0_g1_i1 | blue | 0.7537263 |
| TRINITY_DN32691_c0_g4_i3 | blue | 0.7535316 |
| TRINITY_DN64707_c0_g1_i1 | blue | 0.7508761 |
| TRINITY_DN34101_c0_g2_i10 | blue | 0.7497188 |
| TRINITY_DN29543_c0_g1_i3 | blue | 0.7487262 |
| TRINITY_DN21048_c0_g1_i2 | blue | 0.7478543 |
| TRINITY_DN28935_c0_g1_i2 | blue | 0.7473023 |
| TRINITY_DN36957_c0_g1_i6 | blue | 0.7472541 |
| TRINITY_DN23637_c0_g1_i1 | blue | 0.7469 |
| TRINITY_DN37308_c1_g2_i3 | blue | 0.746055 |
| TRINITY_DN23931_c0_g1_i1 | blue | 0.7458171 |
| TRINITY_DN38429_c12_g1_i2 | blue | 0.7457368 |
| TRINITY_DN37332_c1_g3_i1 | blue | 0.7454691 |
| TRINITY_DN39276_c2_g6_i1 | blue | 0.7450149 |
| TRINITY_DN20473_c0_g1_i1 | blue | 0.7439856 |
| TRINITY_DN1745_c0_g1_i1 | blue | 0.7436403 |
| TRINITY_DN35012_c3_g1_i2 | blue | 0.743474 |
| TRINITY_DN27283_c0_g1_i1 | blue | 0.7433337 |
| TRINITY_DN38985_c1_g14_i1 | blue | 0.7421531 |
| TRINITY_DN18072_c0_g1_i1 | blue | 0.742088 |
| TRINITY_DN10359_c0_g1_i1 | blue | 0.7416459 |
| TRINITY_DN35846_c1_g6_i1 | blue | 0.7412968 |
| TRINITY_DN22283_c0_g1_i1 | blue | 0.7402542 |
| TRINITY_DN4594_c0_g1_i1 | blue | 0.7401776 |
| TRINITY_DN30164_c0_g1_i1 | blue | 0.7401695 |
| TRINITY_DN23331_c0_g1_i1 | blue | 0.7400737 |
| TRINITY_DN33435_c1_g4_i1 | blue | 0.73959 |
| TRINITY_DN41662_c0_g1_i1 | blue | 0.738408 |
| TRINITY_DN24068_c0_g1_i1 | blue | 0.7377081 |
| TRINITY_DN39194_c4_g5_i2 | blue | 0.736688 |
| TRINITY_DN27223_c0_g1_i2 | blue | 0.7360024 |
| TRINITY_DN32360_c1_g4_i1 | blue | 0.7345509 |
| TRINITY_DN27576_c0_g1_i2 | blue | 0.7331193 |
| TRINITY_DN20322_c0_g1_i1 | blue | 0.7330171 |
| TRINITY_DN32552_c0_g3_i3 | blue | 0.7328152 |
| TRINITY_DN116_c0_g2_i1 | blue | 0.7325876 |
| TRINITY_DN13040_c0_g1_i1 | blue | 0.7318954 |
| TRINITY_DN33914_c1_g3_i1 | blue | 0.7318625 |
| TRINITY_DN38755_c1_g7_i4 | blue | 0.7317995 |
| TRINITY_DN27150_c0_g1_i3 | blue | 0.73174 |
| TRINITY_DN28647_c0_g1_i1 | blue | 0.7315774 |
| TRINITY_DN28212_c0_g2_i1 | blue | 0.731409 |
| TRINITY_DN38573_c0_g3_i2 | blue | 0.7305164 |
| TRINITY_DN39082_c1_g1_i7 | blue | 0.729787 |
| TRINITY_DN31376_c0_g2_i6 | blue | 0.7297143 |
| TRINITY_DN22557_c0_g1_i1 | blue | 0.7293517 |
| TRINITY_DN22440_c0_g1_i1 | blue | 0.7292966 |
| TRINITY_DN14542_c0_g1_i1 | blue | 0.7291207 |
| TRINITY_DN29501_c0_g1_i1 | blue | 0.7289456 |
| TRINITY_DN25021_c0_g1_i1 | blue | 0.728462 |
| TRINITY_DN21462_c0_g1_i1 | blue | 0.7267331 |
| TRINITY_DN15653_c0_g1_i1 | blue | 0.7254509 |
| TRINITY_DN38862_c1_g3_i2 | blue | 0.7252882 |
| TRINITY_DN21156_c0_g1_i1 | blue | 0.7249882 |
| TRINITY_DN67542_c0_g1_i1 | blue | 0.7249575 |
| TRINITY_DN32997_c0_g1_i10 | blue | 0.7245912 |
| TRINITY_DN43686_c0_g1_i1 | blue | 0.7240042 |
| TRINITY_DN27690_c0_g5_i1 | blue | 0.7238565 |
| TRINITY_DN27446_c0_g1_i3 | blue | 0.722744 |
| TRINITY_DN29726_c0_g1_i2 | blue | 0.721056 |
| TRINITY_DN25627_c0_g1_i1 | blue | 0.7198192 |
| TRINITY_DN15992_c0_g1_i1 | blue | 0.7195476 |
| TRINITY_DN29562_c1_g4_i1 | blue | 0.7195171 |
| TRINITY_DN37186_c2_g2_i1 | blue | 0.7193592 |
| TRINITY_DN31758_c1_g13_i1 | blue | 0.7193201 |
| TRINITY_DN28873_c0_g2_i2 | blue | 0.7192976 |
| TRINITY_DN34822_c0_g3_i2 | blue | 0.7186803 |
| TRINITY_DN26513_c0_g1_i2 | blue | 0.7180957 |
| TRINITY_DN67834_c0_g1_i1 | blue | 0.7173826 |
| TRINITY_DN56936_c0_g1_i1 | blue | 0.7166817 |
| TRINITY_DN32862_c0_g1_i10 | blue | 0.7161267 |
| TRINITY_DN28934_c0_g1_i8 | blue | 0.7159939 |
| TRINITY_DN23923_c0_g2_i1 | blue | 0.7154683 |
| TRINITY_DN21482_c0_g2_i1 | blue | 0.7153848 |
| TRINITY_DN26239_c0_g1_i4 | blue | 0.7151694 |
| TRINITY_DN22782_c0_g1_i2 | blue | 0.7144587 |
| TRINITY_DN26727_c0_g1_i1 | blue | 0.7137781 |
| TRINITY_DN38442_c7_g10_i1 | blue | 0.7136773 |
| TRINITY_DN33374_c0_g2_i4 | blue | 0.7130362 |
| TRINITY_DN65877_c0_g1_i1 | blue | 0.7129664 |
| TRINITY_DN37869_c0_g1_i1 | blue | 0.711239 |
| TRINITY_DN35463_c1_g2_i4 | blue | 0.7111704 |
| TRINITY_DN26033_c0_g1_i1 | blue | 0.7108818 |
| TRINITY_DN38714_c0_g1_i7 | blue | 0.7096428 |
| TRINITY_DN34995_c0_g2_i3 | blue | 0.708361 |
| TRINITY_DN47714_c0_g1_i1 | blue | 0.7075323 |
| TRINITY_DN38226_c0_g4_i1 | blue | 0.7070389 |
| TRINITY_DN37720_c1_g1_i9 | blue | 0.7068732 |
| TRINITY_DN29440_c0_g1_i2 | blue | 0.7068649 |
| TRINITY_DN37799_c1_g1_i2 | blue | 0.7067331 |
| TRINITY_DN37923_c2_g2_i5 | blue | 0.7065153 |
| TRINITY_DN24839_c0_g1_i2 | blue | 0.7061958 |
| TRINITY_DN32991_c0_g4_i1 | blue | 0.7048408 |
| TRINITY_DN38755_c1_g4_i2 | blue | 0.7047599 |
| TRINITY_DN21791_c0_g2_i1 | blue | 0.7042287 |
| TRINITY_DN38442_c7_g16_i1 | blue | 0.7041944 |
| TRINITY_DN35951_c0_g1_i1 | blue | 0.7041559 |
| TRINITY_DN67967_c0_g1_i1 | blue | 0.7038543 |
| TRINITY_DN23670_c0_g1_i1 | blue | 0.7033367 |
| TRINITY_DN18676_c0_g1_i1 | blue | 0.702751 |
| TRINITY_DN28661_c0_g1_i1 | blue | 0.7025308 |
| TRINITY_DN37306_c0_g7_i1 | blue | 0.7022763 |
| TRINITY_DN34574_c1_g1_i1 | blue | 0.7022268 |
| TRINITY_DN26665_c0_g1_i1 | blue | 0.7021704 |
| TRINITY_DN25904_c0_g1_i1 | blue | 0.7015653 |
| TRINITY_DN44843_c0_g1_i1 | blue | 0.7013239 |
| TRINITY_DN46613_c0_g1_i1 | blue | 0.7007103 |
| TRINITY_DN34891_c0_g1_i1 | blue | 0.6997599 |
| TRINITY_DN29966_c1_g1_i1 | blue | 0.6996627 |
| TRINITY_DN45429_c0_g1_i1 | blue | 0.6990162 |
| TRINITY_DN62222_c0_g1_i1 | blue | 0.6971604 |
| TRINITY_DN16880_c0_g1_i1 | blue | 0.694664 |
| TRINITY_DN20890_c0_g1_i1 | blue | 0.6930068 |
| TRINITY_DN57402_c0_g1_i1 | blue | 0.6923623 |
| TRINITY_DN37246_c2_g2_i1 | blue | 0.6920616 |
| TRINITY_DN21856_c0_g1_i1 | blue | 0.6917304 |
| TRINITY_DN39329_c12_g2_i1 | blue | 0.6912101 |
| TRINITY_DN39747_c0_g1_i1 | blue | 0.6905396 |
| TRINITY_DN38872_c1_g3_i15 | blue | 0.6896949 |
| TRINITY_DN30652_c0_g1_i1 | blue | 0.6890121 |
| TRINITY_DN35517_c0_g2_i1 | blue | 0.6882723 |
| TRINITY_DN19772_c0_g1_i1 | blue | 0.688078 |
| TRINITY_DN28791_c0_g1_i1 | blue | 0.6870889 |
| TRINITY_DN36138_c0_g2_i1 | blue | 0.6868887 |
| TRINITY_DN24382_c0_g1_i1 | blue | 0.6866629 |
| TRINITY_DN38461_c1_g5_i1 | blue | 0.6860419 |
| TRINITY_DN38877_c0_g1_i1 | blue | 0.6858305 |
| TRINITY_DN10498_c0_g1_i1 | blue | 0.6856951 |
| TRINITY_DN4253_c0_g2_i1 | blue | 0.6841613 |
| TRINITY_DN15594_c0_g1_i1 | blue | 0.6839767 |
| TRINITY_DN27260_c0_g1_i1 | blue | 0.6837594 |
| TRINITY_DN22057_c0_g1_i1 | blue | 0.683652 |
| TRINITY_DN22578_c0_g1_i1 | blue | 0.6822057 |
| TRINITY_DN18678_c0_g1_i1 | blue | 0.6817082 |
| TRINITY_DN37791_c2_g8_i8 | blue | 0.680929 |
| TRINITY_DN30294_c0_g1_i2 | blue | 0.6806189 |
| TRINITY_DN22238_c0_g2_i1 | blue | 0.6788595 |
| TRINITY_DN29102_c0_g3_i1 | blue | 0.6776988 |
| TRINITY_DN13984_c0_g2_i1 | blue | 0.6767928 |
| TRINITY_DN35095_c2_g2_i1 | blue | 0.675699 |
| TRINITY_DN26203_c0_g1_i1 | blue | 0.6754606 |
| TRINITY_DN17153_c0_g1_i1 | blue | 0.6749289 |
| TRINITY_DN36529_c1_g2_i4 | blue | 0.6741491 |
| TRINITY_DN71233_c0_g1_i1 | blue | 0.6741052 |
| TRINITY_DN36705_c1_g2_i2 | blue | 0.673348 |
| TRINITY_DN42443_c0_g1_i1 | blue | 0.6728877 |
| TRINITY_DN11852_c0_g1_i1 | blue | 0.6721052 |
| TRINITY_DN30788_c0_g1_i1 | blue | 0.6715597 |
| TRINITY_DN901_c0_g1_i1 | blue | 0.6711423 |
| TRINITY_DN36705_c1_g1_i1 | blue | 0.6704463 |
| TRINITY_DN25657_c0_g1_i1 | blue | 0.6702179 |
| TRINITY_DN21772_c0_g1_i1 | blue | 0.6698728 |
| TRINITY_DN38750_c1_g3_i1 | blue | 0.667234 |
| TRINITY_DN32936_c1_g1_i2 | blue | 0.6667825 |
| TRINITY_DN16225_c0_g1_i1 | blue | 0.6666069 |
| TRINITY_DN39125_c0_g2_i1 | blue | 0.6665122 |
| TRINITY_DN33381_c0_g1_i7 | blue | 0.6650641 |
| TRINITY_DN22468_c0_g1_i2 | blue | 0.6649987 |
| TRINITY_DN36706_c0_g1_i1 | blue | 0.6640899 |
| TRINITY_DN32864_c2_g2_i3 | blue | 0.6635486 |
| TRINITY_DN39418_c0_g1_i1 | blue | 0.6632637 |
| TRINITY_DN48137_c0_g1_i1 | blue | 0.6607652 |
| TRINITY_DN6662_c0_g1_i1 | blue | 0.6607474 |
| TRINITY_DN56624_c0_g1_i1 | blue | 0.6606896 |
| TRINITY_DN4727_c0_g1_i1 | blue | 0.6589332 |
| TRINITY_DN25990_c1_g3_i1 | blue | 0.6583468 |
| TRINITY_DN37680_c0_g2_i15 | blue | 0.6564407 |
| TRINITY_DN37281_c0_g1_i1 | blue | 0.6563851 |
| TRINITY_DN25457_c0_g1_i1 | blue | 0.6559378 |
| TRINITY_DN38295_c3_g6_i1 | blue | 0.6539954 |
| TRINITY_DN38408_c0_g1_i1 | blue | 0.6537497 |
| TRINITY_DN23643_c0_g1_i1 | blue | 0.6531639 |
| TRINITY_DN32608_c0_g3_i1 | blue | 0.6505138 |
| TRINITY_DN16112_c0_g1_i1 | blue | 0.6500271 |
| TRINITY_DN46964_c0_g1_i1 | blue | 0.6492141 |
| TRINITY_DN37392_c0_g2_i1 | blue | 0.6470308 |
| TRINITY_DN39082_c1_g2_i1 | blue | 0.6465275 |
| TRINITY_DN22523_c0_g1_i1 | blue | 0.6454828 |
| TRINITY_DN22164_c0_g3_i1 | blue | 0.6454555 |
| TRINITY_DN33842_c0_g1_i1 | blue | 0.6453686 |
| TRINITY_DN37491_c0_g6_i2 | blue | 0.6442416 |
| TRINITY_DN62676_c0_g1_i1 | blue | 0.6426343 |
| TRINITY_DN26408_c0_g2_i1 | blue | 0.6425329 |
| TRINITY_DN39330_c1_g6_i2 | blue | 0.6423517 |
| TRINITY_DN34909_c2_g1_i1 | blue | 0.6407934 |
| TRINITY_DN32982_c0_g1_i2 | blue | 0.6401762 |
| TRINITY_DN37808_c0_g2_i1 | blue | 0.6397174 |
| TRINITY_DN38578_c0_g6_i1 | blue | 0.6385898 |
| TRINITY_DN30589_c0_g2_i1 | blue | 0.6372906 |
| TRINITY_DN19107_c0_g1_i1 | blue | 0.6368796 |
| TRINITY_DN9311_c0_g1_i1 | blue | 0.636394 |
| TRINITY_DN37694_c2_g2_i2 | blue | 0.6348839 |
| TRINITY_DN22582_c0_g3_i1 | blue | 0.6343356 |
| TRINITY_DN39129_c2_g5_i2 | blue | 0.6335527 |
| TRINITY_DN19486_c0_g2_i1 | blue | 0.633399 |
| TRINITY_DN18140_c0_g1_i1 | blue | 0.6333974 |
| TRINITY_DN38991_c0_g5_i1 | blue | 0.6323125 |
| TRINITY_DN39637_c0_g1_i1 | blue | 0.6313829 |
| TRINITY_DN29858_c1_g1_i1 | blue | 0.6269824 |
| TRINITY_DN37117_c0_g5_i1 | blue | 0.6269533 |
| TRINITY_DN34922_c2_g3_i1 | blue | 0.6247036 |
| TRINITY_DN31828_c0_g1_i1 | blue | 0.6241622 |
| TRINITY_DN36079_c0_g6_i3 | blue | 0.6237688 |
| TRINITY_DN19802_c0_g2_i1 | blue | 0.6236721 |
| TRINITY_DN9072_c0_g1_i1 | blue | 0.6224074 |
| TRINITY_DN23474_c0_g1_i1 | blue | 0.6215372 |
| TRINITY_DN22921_c0_g2_i1 | blue | 0.619394 |
| TRINITY_DN25961_c0_g2_i1 | blue | 0.6189426 |
| TRINITY_DN30445_c0_g3_i1 | blue | 0.618856 |
| TRINITY_DN45913_c0_g1_i1 | blue | 0.6187937 |
| TRINITY_DN28141_c0_g3_i1 | blue | 0.6174613 |
| TRINITY_DN22499_c0_g2_i1 | blue | 0.6161834 |
| TRINITY_DN37481_c1_g2_i1 | blue | 0.6156326 |
| TRINITY_DN46557_c0_g1_i1 | blue | 0.6149674 |
| TRINITY_DN25834_c0_g1_i1 | blue | 0.6147409 |
| TRINITY_DN53787_c0_g1_i1 | blue | 0.6146039 |
| TRINITY_DN36089_c0_g1_i1 | blue | 0.6123771 |
| TRINITY_DN31471_c0_g1_i5 | blue | 0.6116119 |
| TRINITY_DN39186_c4_g1_i1 | blue | 0.610844 |
| TRINITY_DN22582_c0_g1_i1 | blue | 0.6077974 |
| TRINITY_DN26883_c1_g1_i3 | blue | 0.6072024 |
| TRINITY_DN67910_c0_g1_i1 | blue | 0.6068436 |
| TRINITY_DN37787_c1_g2_i6 | blue | 0.606619 |
| TRINITY_DN30348_c0_g3_i9 | blue | 0.6054273 |
| TRINITY_DN56649_c0_g1_i1 | blue | 0.6045906 |
| TRINITY_DN25122_c0_g1_i1 | blue | 0.6035086 |
| TRINITY_DN34151_c0_g2_i1 | blue | 0.6032911 |
| TRINITY_DN30329_c0_g1_i1 | blue | 0.6030209 |
| TRINITY_DN34304_c0_g10_i1 | blue | 0.5993806 |
| TRINITY_DN42610_c0_g1_i1 | blue | 0.5987897 |
| TRINITY_DN38551_c1_g6_i1 | blue | 0.5981815 |
| TRINITY_DN28433_c0_g1_i1 | blue | 0.5977669 |
| TRINITY_DN38489_c0_g3_i7 | blue | 0.5971399 |
| TRINITY_DN64866_c0_g1_i1 | blue | 0.5967374 |
| TRINITY_DN16178_c0_g1_i1 | blue | 0.5962202 |
| TRINITY_DN25110_c0_g1_i2 | blue | 0.5931342 |
| TRINITY_DN27245_c0_g2_i2 | blue | 0.5927361 |
| TRINITY_DN20990_c0_g2_i1 | blue | 0.591464 |
| TRINITY_DN39238_c0_g1_i1 | blue | 0.5910355 |
| TRINITY_DN28876_c0_g2_i1 | blue | 0.5906531 |
| TRINITY_DN27721_c0_g1_i2 | blue | 0.5898664 |
| TRINITY_DN25648_c0_g1_i1 | blue | 0.5893031 |
| TRINITY_DN26181_c0_g2_i1 | blue | 0.5892661 |
| TRINITY_DN20870_c0_g1_i1 | blue | 0.587035 |
| TRINITY_DN69484_c0_g1_i1 | blue | 0.5861983 |
| TRINITY_DN31409_c0_g3_i1 | blue | 0.5851218 |
| TRINITY_DN62446_c0_g1_i1 | blue | 0.5835934 |
| TRINITY_DN38513_c0_g1_i1 | blue | 0.583064 |
| TRINITY_DN27551_c0_g1_i1 | blue | 0.5825862 |
| TRINITY_DN16469_c0_g1_i1 | blue | 0.5823106 |
| TRINITY_DN38407_c0_g1_i1 | blue | 0.5803129 |
| TRINITY_DN23548_c0_g1_i2 | blue | 0.5802715 |
| TRINITY_DN60044_c0_g1_i1 | blue | 0.5796732 |
| TRINITY_DN23770_c0_g1_i6 | blue | 0.5791525 |
| TRINITY_DN19216_c0_g1_i1 | blue | 0.5786576 |
| TRINITY_DN35929_c0_g2_i4 | blue | 0.5784171 |
| TRINITY_DN25797_c0_g1_i4 | blue | 0.5778763 |
| TRINITY_DN27181_c0_g1_i1 | blue | 0.5765973 |
| TRINITY_DN20648_c0_g1_i1 | blue | 0.5764548 |
| TRINITY_DN37356_c1_g2_i2 | blue | 0.5759861 |
| TRINITY_DN18581_c0_g1_i1 | blue | 0.5716919 |
| TRINITY_DN21456_c0_g2_i1 | blue | 0.5716706 |
| TRINITY_DN1240_c0_g1_i1 | blue | 0.5715366 |
| TRINITY_DN26760_c0_g1_i1 | blue | 0.5714318 |
| TRINITY_DN61983_c0_g1_i1 | blue | 0.5713328 |
| TRINITY_DN58522_c0_g1_i1 | blue | 0.5711787 |
| TRINITY_DN24861_c0_g1_i1 | blue | 0.5698213 |
| TRINITY_DN22081_c0_g1_i1 | blue | 0.5687557 |
| TRINITY_DN28937_c0_g1_i1 | blue | 0.5687458 |
| TRINITY_DN36883_c1_g1_i1 | blue | 0.5671117 |
| TRINITY_DN21008_c0_g1_i1 | blue | 0.5661325 |
| TRINITY_DN67366_c0_g1_i1 | blue | 0.564472 |
| TRINITY_DN23100_c0_g1_i1 | blue | 0.5644118 |
| TRINITY_DN36115_c2_g3_i1 | blue | 0.5636366 |
| TRINITY_DN50712_c0_g1_i1 | blue | 0.5628189 |
| TRINITY_DN12008_c0_g1_i1 | blue | 0.5620601 |
| TRINITY_DN34922_c2_g2_i1 | blue | 0.5612578 |
| TRINITY_DN27658_c0_g1_i1 | blue | 0.5589411 |
| TRINITY_DN5268_c0_g1_i1 | blue | 0.5584821 |
| TRINITY_DN21545_c0_g1_i1 | blue | 0.5571799 |
| TRINITY_DN21644_c0_g1_i1 | blue | 0.5551295 |
| TRINITY_DN30505_c0_g1_i1 | blue | 0.5544512 |
| TRINITY_DN28499_c0_g2_i1 | blue | 0.5541978 |
| TRINITY_DN35483_c1_g2_i3 | blue | 0.5540561 |
| TRINITY_DN37380_c0_g2_i5 | blue | 0.5538026 |
| TRINITY_DN20461_c0_g1_i1 | blue | 0.5536807 |
| TRINITY_DN31558_c1_g2_i1 | blue | 0.5529883 |
| TRINITY_DN38511_c2_g1_i1 | blue | 0.5525031 |
| TRINITY_DN9116_c0_g1_i1 | blue | 0.5499658 |
| TRINITY_DN37193_c0_g3_i1 | blue | 0.5499315 |
| TRINITY_DN23681_c0_g1_i1 | blue | 0.5498943 |
| TRINITY_DN20967_c0_g2_i1 | blue | 0.5498578 |
| TRINITY_DN23815_c0_g1_i3 | blue | 0.5490757 |
| TRINITY_DN20803_c0_g2_i1 | blue | 0.5477641 |
| TRINITY_DN23017_c0_g1_i4 | blue | 0.5476304 |
| TRINITY_DN38442_c6_g5_i1 | blue | 0.5474891 |
| TRINITY_DN30896_c0_g2_i1 | blue | 0.5470667 |
| TRINITY_DN39708_c0_g1_i1 | blue | 0.545821 |
| TRINITY_DN18611_c0_g1_i1 | blue | 0.5457449 |
| TRINITY_DN21071_c0_g1_i1 | blue | 0.545596 |
| TRINITY_DN35526_c2_g1_i1 | blue | 0.5446652 |
| TRINITY_DN25583_c0_g1_i1 | blue | 0.5433036 |
| TRINITY_DN27921_c0_g4_i1 | blue | 0.542613 |
| TRINITY_DN27424_c0_g1_i1 | blue | 0.5421004 |
| TRINITY_DN62183_c0_g1_i1 | blue | 0.5418405 |
| TRINITY_DN29684_c4_g8_i1 | blue | 0.541147 |
| TRINITY_DN21964_c0_g3_i1 | blue | 0.5407499 |
| TRINITY_DN32494_c2_g6_i5 | blue | 0.5401995 |
| TRINITY_DN29582_c0_g1_i1 | blue | 0.5391676 |
| TRINITY_DN62722_c0_g1_i1 | blue | 0.5387839 |
| TRINITY_DN34101_c0_g1_i5 | blue | 0.5384618 |
| TRINITY_DN38876_c0_g3_i1 | blue | 0.5380627 |
| TRINITY_DN63333_c0_g1_i1 | blue | 0.536703 |
| TRINITY_DN13824_c0_g2_i1 | blue | 0.5359552 |
| TRINITY_DN24770_c0_g1_i1 | blue | 0.534365 |
| TRINITY_DN42275_c0_g1_i1 | blue | 0.5337341 |
| TRINITY_DN23621_c1_g1_i1 | blue | 0.5332017 |
| TRINITY_DN26028_c1_g1_i2 | blue | 0.5317363 |
| TRINITY_DN29156_c0_g1_i3 | blue | 0.5314054 |
| TRINITY_DN31843_c0_g2_i2 | blue | 0.5309545 |
| TRINITY_DN13573_c0_g1_i1 | blue | 0.5308882 |
| TRINITY_DN61897_c0_g1_i1 | blue | 0.5307752 |
| TRINITY_DN8867_c0_g1_i1 | blue | 0.5302744 |
| TRINITY_DN18659_c0_g1_i1 | blue | 0.5291589 |
| TRINITY_DN24675_c0_g1_i1 | blue | 0.5285457 |
| TRINITY_DN18260_c0_g1_i1 | blue | 0.5283047 |
| TRINITY_DN27382_c1_g1_i1 | blue | 0.528067 |
| TRINITY_DN23553_c0_g1_i2 | blue | 0.5272997 |
| TRINITY_DN28355_c1_g1_i1 | blue | 0.5272148 |
| TRINITY_DN45389_c0_g1_i1 | blue | 0.5271978 |
| TRINITY_DN24252_c0_g1_i1 | blue | 0.5269251 |
| TRINITY_DN9164_c0_g1_i1 | blue | 0.5252087 |
| TRINITY_DN17167_c0_g1_i1 | blue | 0.5250732 |
| TRINITY_DN22779_c0_g2_i1 | blue | 0.5248451 |
| TRINITY_DN62266_c0_g1_i1 | blue | 0.5243215 |
| TRINITY_DN12970_c0_g1_i1 | blue | 0.5232462 |
| TRINITY_DN35230_c0_g3_i3 | blue | 0.5226481 |
| TRINITY_DN67500_c0_g1_i1 | blue | 0.522512 |
| TRINITY_DN34194_c1_g2_i1 | blue | 0.5223742 |
| TRINITY_DN56871_c0_g1_i1 | blue | 0.5222902 |
| TRINITY_DN18646_c0_g2_i1 | blue | 0.5215866 |
| TRINITY_DN34071_c2_g1_i1 | blue | 0.5211316 |
| TRINITY_DN23530_c0_g1_i1 | blue | 0.5203959 |
| TRINITY_DN25863_c0_g3_i1 | blue | 0.5203877 |
| TRINITY_DN37892_c1_g5_i1 | blue | 0.5201772 |
| TRINITY_DN44815_c0_g1_i1 | blue | 0.5181817 |
| TRINITY_DN30079_c0_g2_i2 | blue | 0.518082 |
| TRINITY_DN56289_c0_g1_i1 | blue | 0.5180343 |
| TRINITY_DN25332_c0_g1_i1 | blue | 0.5174059 |
| TRINITY_DN51019_c0_g1_i1 | blue | 0.5166455 |
| TRINITY_DN29684_c4_g6_i1 | blue | 0.515826 |
| TRINITY_DN11262_c0_g1_i1 | blue | 0.5142824 |
| TRINITY_DN23657_c0_g1_i1 | blue | 0.5141708 |
| TRINITY_DN40171_c0_g1_i1 | blue | 0.5141689 |
| TRINITY_DN28355_c2_g1_i3 | blue | 0.5139315 |
| TRINITY_DN26206_c0_g4_i1 | blue | 0.5132536 |
| TRINITY_DN35035_c0_g1_i1 | blue | 0.5115834 |
| TRINITY_DN23982_c0_g1_i1 | blue | 0.510839 |
| TRINITY_DN23748_c0_g3_i1 | blue | 0.5104997 |
| TRINITY_DN21277_c0_g1_i1 | blue | 0.509531 |
| TRINITY_DN24346_c0_g1_i1 | blue | 0.5089949 |
| TRINITY_DN25593_c0_g2_i1 | blue | 0.508928 |
| TRINITY_DN8888_c0_g1_i1 | blue | 0.5088636 |
| TRINITY_DN41039_c0_g1_i1 | blue | 0.508599 |
| TRINITY_DN21013_c0_g1_i1 | blue | 0.5068893 |
| TRINITY_DN32872_c0_g1_i6 | blue | 0.5053959 |
| TRINITY_DN8782_c0_g1_i1 | blue | 0.5042744 |
| TRINITY_DN35343_c0_g6_i2 | blue | 0.5041402 |
| TRINITY_DN21201_c0_g2_i1 | blue | 0.5007924 |
| TRINITY_DN55935_c0_g1_i1 | blue | 0.5005609 |
| TRINITY_DN23128_c0_g1_i1 | blue | 0.5003189 |
| TRINITY_DN56956_c0_g1_i1 | blue | 0.4998106 |
| TRINITY_DN28313_c0_g1_i3 | blue | 0.4989285 |
| TRINITY_DN21094_c0_g1_i1 | blue | 0.4981654 |
| TRINITY_DN4276_c0_g1_i1 | blue | 0.4979918 |
| TRINITY_DN52411_c0_g1_i1 | blue | 0.4977371 |
| TRINITY_DN3333_c0_g1_i1 | blue | 0.4959905 |
| TRINITY_DN16263_c0_g1_i1 | blue | 0.4957137 |
| TRINITY_DN45166_c0_g1_i1 | blue | 0.4955601 |
| TRINITY_DN24578_c0_g1_i1 | blue | 0.4920767 |
| TRINITY_DN28080_c0_g1_i2 | blue | 0.4919887 |
| TRINITY_DN27951_c0_g3_i1 | blue | 0.4918048 |
| TRINITY_DN22962_c0_g2_i1 | blue | 0.4911851 |
| TRINITY_DN33301_c0_g5_i1 | blue | 0.4887466 |
| TRINITY_DN22138_c0_g1_i2 | blue | 0.4882446 |
| TRINITY_DN19397_c0_g1_i1 | blue | 0.4874036 |
| TRINITY_DN34364_c1_g1_i1 | blue | 0.4860583 |
| TRINITY_DN39109_c1_g3_i1 | blue | 0.4856123 |
| TRINITY_DN27614_c1_g1_i3 | blue | 0.4851064 |
| TRINITY_DN3447_c0_g1_i1 | blue | 0.4823341 |
| TRINITY_DN17466_c0_g1_i1 | blue | 0.4822602 |
| TRINITY_DN25628_c0_g1_i1 | blue | 0.4817707 |
| TRINITY_DN22893_c0_g1_i1 | blue | 0.4817667 |
| TRINITY_DN26583_c0_g7_i1 | blue | 0.4816178 |
| TRINITY_DN22007_c0_g1_i1 | blue | 0.4813808 |
| TRINITY_DN33019_c0_g1_i1 | blue | 0.4810377 |
| TRINITY_DN26784_c0_g1_i1 | blue | 0.4792267 |
| TRINITY_DN6486_c0_g1_i1 | blue | 0.4779985 |
| TRINITY_DN19627_c0_g1_i1 | blue | 0.477828 |
| TRINITY_DN16828_c0_g1_i1 | blue | 0.4744462 |
| TRINITY_DN40354_c0_g1_i1 | blue | 0.4742826 |
| TRINITY_DN25596_c0_g1_i1 | blue | 0.4728685 |
| TRINITY_DN62978_c0_g1_i1 | blue | 0.4721517 |
| TRINITY_DN25850_c0_g4_i1 | blue | 0.4720634 |
| TRINITY_DN17197_c0_g1_i1 | blue | 0.471256 |
| TRINITY_DN21932_c0_g1_i1 | blue | 0.4709219 |
| TRINITY_DN32251_c0_g1_i2 | blue | 0.4697177 |
| TRINITY_DN38521_c0_g1_i2 | blue | 0.4686379 |
| TRINITY_DN19891_c0_g2_i1 | blue | 0.4684633 |
| TRINITY_DN24810_c0_g1_i1 | blue | 0.4684463 |
| TRINITY_DN31299_c0_g1_i1 | blue | 0.4678709 |
| TRINITY_DN21677_c0_g1_i1 | blue | 0.4655521 |
| TRINITY_DN41855_c0_g1_i1 | blue | 0.4643518 |
| TRINITY_DN23582_c0_g2_i1 | blue | 0.4635359 |
| TRINITY_DN1858_c0_g1_i1 | blue | 0.4634449 |
| TRINITY_DN24068_c0_g2_i1 | blue | 0.4625927 |
| TRINITY_DN18976_c0_g1_i1 | blue | 0.4613885 |
| TRINITY_DN31672_c0_g1_i4 | blue | 0.4612989 |
| TRINITY_DN45501_c0_g1_i1 | blue | 0.4606603 |
| TRINITY_DN16464_c0_g2_i1 | blue | 0.4605841 |
| TRINITY_DN71430_c0_g1_i1 | blue | 0.4604526 |
| TRINITY_DN39379_c0_g1_i1 | blue | 0.4583927 |
| TRINITY_DN38578_c0_g2_i2 | blue | 0.4576796 |
| TRINITY_DN29502_c0_g1_i6 | blue | 0.4574041 |
| TRINITY_DN19213_c0_g1_i1 | blue | 0.4549417 |
| TRINITY_DN33566_c0_g1_i1 | blue | 0.4539307 |
| TRINITY_DN32613_c0_g1_i3 | blue | 0.4520822 |
| TRINITY_DN4569_c0_g1_i1 | blue | 0.4515105 |
| TRINITY_DN17020_c0_g1_i1 | blue | 0.4513807 |
| TRINITY_DN21727_c0_g1_i1 | blue | 0.4503366 |
| TRINITY_DN17044_c0_g1_i1 | blue | 0.4489704 |
| TRINITY_DN17091_c0_g1_i1 | blue | 0.4488174 |
| TRINITY_DN17090_c0_g2_i1 | blue | 0.4473318 |
| TRINITY_DN33789_c0_g8_i1 | blue | 0.4464429 |
| TRINITY_DN22216_c0_g2_i1 | blue | 0.4451349 |
| TRINITY_DN31192_c0_g4_i1 | blue | 0.4440181 |
| TRINITY_DN25856_c0_g2_i2 | blue | 0.4439794 |
| TRINITY_DN46180_c0_g1_i1 | blue | 0.4433486 |
| TRINITY_DN63884_c0_g1_i1 | blue | 0.4406838 |
| TRINITY_DN39060_c0_g10_i1 | blue | 0.4401985 |
| TRINITY_DN22234_c0_g1_i1 | blue | 0.4386546 |
| TRINITY_DN19596_c0_g2_i1 | blue | 0.4376561 |
| TRINITY_DN26120_c0_g1_i1 | blue | 0.4376087 |
| TRINITY_DN7415_c0_g1_i1 | blue | 0.4363154 |
| TRINITY_DN59442_c0_g1_i1 | blue | 0.4360389 |
| TRINITY_DN17216_c0_g3_i1 | blue | 0.435863 |
| TRINITY_DN68388_c0_g1_i1 | blue | 0.4357654 |
| TRINITY_DN38723_c1_g1_i1 | blue | 0.4338051 |
| TRINITY_DN31950_c0_g11_i1 | blue | 0.4333793 |
| TRINITY_DN38176_c1_g4_i2 | blue | 0.4321783 |
| TRINITY_DN19526_c0_g2_i1 | blue | 0.4318386 |
| TRINITY_DN22809_c0_g1_i1 | blue | 0.4304706 |
| TRINITY_DN39861_c0_g1_i1 | blue | 0.4304412 |
| TRINITY_DN23621_c0_g2_i1 | blue | 0.4288089 |
| TRINITY_DN27221_c0_g1_i1 | blue | 0.4276515 |
| TRINITY_DN18921_c0_g1_i1 | blue | 0.4272464 |
| TRINITY_DN16891_c0_g1_i1 | blue | 0.426625 |
| TRINITY_DN22792_c0_g1_i1 | blue | 0.4265035 |
| TRINITY_DN30190_c1_g4_i1 | blue | 0.4255832 |
| TRINITY_DN34304_c0_g4_i1 | blue | 0.4251603 |
| TRINITY_DN24040_c0_g1_i1 | blue | 0.4241039 |
| TRINITY_DN72079_c0_g1_i1 | blue | 0.4238497 |
| TRINITY_DN69174_c0_g1_i1 | blue | 0.4222522 |
| TRINITY_DN17287_c0_g2_i1 | blue | 0.4212425 |
| TRINITY_DN33927_c0_g1_i1 | blue | 0.4209307 |
| TRINITY_DN24065_c0_g1_i1 | blue | 0.4206637 |
| TRINITY_DN28072_c0_g1_i1 | blue | 0.4196306 |
| TRINITY_DN24147_c0_g1_i2 | blue | 0.4190102 |
| TRINITY_DN23883_c0_g2_i2 | blue | 0.4150623 |
| TRINITY_DN8963_c0_g1_i1 | blue | 0.412991 |
| TRINITY_DN22249_c0_g1_i2 | blue | 0.4126663 |
| TRINITY_DN49725_c0_g2_i1 | blue | 0.4126604 |
| TRINITY_DN39779_c0_g1_i1 | blue | 0.4092473 |
| TRINITY_DN47513_c0_g1_i1 | blue | 0.4092288 |
| TRINITY_DN2054_c0_g1_i1 | blue | 0.4079731 |
| TRINITY_DN51533_c0_g1_i1 | blue | 0.4061001 |
| TRINITY_DN17422_c0_g3_i1 | blue | 0.404087 |
| TRINITY_DN29684_c3_g2_i1 | blue | 0.3956959 |
| TRINITY_DN32823_c1_g4_i3 | blue | 0.3939902 |
| TRINITY_DN36692_c0_g4_i7 | blue | 0.3938766 |
| TRINITY_DN23209_c0_g1_i1 | blue | 0.3938379 |
| TRINITY_DN27600_c0_g1_i2 | blue | 0.3902021 |
| TRINITY_DN20762_c0_g3_i1 | blue | 0.3896427 |
| TRINITY_DN53299_c0_g1_i1 | blue | 0.3895198 |
| TRINITY_DN2158_c0_g1_i1 | blue | 0.3892464 |
| TRINITY_DN55067_c0_g1_i1 | blue | 0.3890874 |
| TRINITY_DN26932_c0_g1_i1 | blue | 0.3884948 |
| TRINITY_DN25120_c0_g1_i1 | blue | 0.3878878 |
| TRINITY_DN69050_c0_g2_i1 | blue | 0.3876975 |
| TRINITY_DN19469_c0_g1_i1 | blue | 0.3872984 |
| TRINITY_DN24888_c0_g1_i1 | blue | 0.3863957 |
| TRINITY_DN52097_c0_g1_i1 | blue | 0.3724701 |
| TRINITY_DN38787_c0_g1_i2 | blue | 0.3716668 |
| TRINITY_DN34754_c0_g1_i2 | blue | 0.3688239 |
| TRINITY_DN24900_c0_g1_i1 | blue | 0.3606944 |
| TRINITY_DN37370_c0_g1_i1 | blue | 0.3604293 |
| TRINITY_DN39335_c10_g5_i1 | blue | 0.3527825 |
| TRINITY_DN39335_c10_g6_i5 | blue | 0.3514986 |
| TRINITY_DN32471_c0_g2_i1 | blue | 0.3380794 |
| TRINITY_DN23838_c0_g4_i1 | blue | 0.3224951 |
| TRINITY_DN27925_c0_g1_i1 | blue | 0.3207007 |
| TRINITY_DN34522_c2_g1_i3 | blue | 0.2791141 |
| TRINITY_DN35239_c4_g3_i1 | blue | 0.2755604 |
| TRINITY_DN23597_c0_g1_i1 | blue | -0.181962 |
| TRINITY_DN25361_c0_g1_i1 | blue | -0.193663 |
| TRINITY_DN38048_c2_g10_i5 | blue | -0.234253 |
| TRINITY_DN35967_c1_g2_i1 | blue | -0.261672 |
| TRINITY_DN38354_c0_g1_i2 | blue | -0.268347 |
| TRINITY_DN25696_c0_g1_i1 | blue | -0.317469 |
| TRINITY_DN33478_c0_g4_i1 | blue | -0.317897 |
| TRINITY_DN26962_c0_g1_i2 | blue | -0.337058 |
| TRINITY_DN48198_c0_g1_i1 | blue | -0.352 |
| TRINITY_DN35526_c2_g14_i4 | blue | -0.359806 |
| TRINITY_DN39060_c0_g3_i1 | blue | -0.364637 |
| TRINITY_DN25498_c0_g2_i2 | blue | -0.373525 |
| TRINITY_DN23113_c0_g1_i1 | blue | -0.40838 |
| TRINITY_DN31424_c0_g1_i2 | blue | -0.414448 |
| TRINITY_DN36588_c4_g1_i8 | blue | -0.415724 |
| TRINITY_DN29499_c0_g2_i1 | blue | -0.419321 |
| TRINITY_DN31150_c0_g2_i2 | blue | -0.422164 |
| TRINITY_DN17029_c0_g1_i1 | blue | -0.42233 |
| TRINITY_DN34824_c1_g1_i3 | blue | -0.428396 |
| TRINITY_DN35362_c0_g5_i1 | blue | -0.428835 |
| TRINITY_DN33491_c0_g4_i2 | blue | -0.429858 |
| TRINITY_DN23587_c0_g1_i1 | blue | -0.435836 |
| TRINITY_DN27076_c0_g2_i1 | blue | -0.445149 |
| TRINITY_DN35285_c0_g1_i2 | blue | -0.454449 |
| TRINITY_DN247_c0_g1_i1 | blue | -0.458834 |
| TRINITY_DN37035_c2_g2_i3 | blue | -0.45963 |
| TRINITY_DN30926_c0_g2_i3 | blue | -0.461206 |
| TRINITY_DN17417_c0_g2_i1 | blue | -0.462433 |
| TRINITY_DN26024_c0_g1_i1 | blue | -0.46269 |
| TRINITY_DN21282_c0_g1_i1 | blue | -0.468538 |
| TRINITY_DN20861_c0_g1_i1 | blue | -0.47603 |
| TRINITY_DN34963_c0_g1_i3 | blue | -0.476264 |
| TRINITY_DN31024_c0_g1_i1 | blue | -0.481788 |
| TRINITY_DN35458_c0_g3_i8 | blue | -0.488916 |
| TRINITY_DN21353_c0_g1_i1 | blue | -0.493459 |
| TRINITY_DN37466_c1_g8_i1 | blue | -0.498471 |
| TRINITY_DN28942_c0_g1_i1 | blue | -0.499161 |
| TRINITY_DN32966_c0_g2_i9 | blue | -0.50074 |
| TRINITY_DN27888_c0_g1_i4 | blue | -0.500806 |
| TRINITY_DN39057_c0_g3_i1 | blue | -0.501247 |
| TRINITY_DN36944_c0_g2_i2 | blue | -0.503847 |
| TRINITY_DN39317_c6_g16_i1 | blue | -0.504323 |
| TRINITY_DN39266_c2_g1_i2 | blue | -0.506123 |
| TRINITY_DN20480_c0_g2_i1 | blue | -0.506245 |
| TRINITY_DN45163_c0_g1_i1 | blue | -0.507771 |
| TRINITY_DN31144_c0_g1_i1 | blue | -0.513697 |
| TRINITY_DN35393_c0_g2_i3 | blue | -0.513993 |
| TRINITY_DN32851_c1_g3_i1 | blue | -0.516141 |
| TRINITY_DN26105_c0_g2_i2 | blue | -0.520766 |
| TRINITY_DN37200_c1_g2_i2 | blue | -0.52283 |
| TRINITY_DN18270_c0_g2_i1 | blue | -0.523157 |
| TRINITY_DN26832_c0_g1_i6 | blue | -0.525744 |
| TRINITY_DN34261_c1_g5_i1 | blue | -0.527372 |
| TRINITY_DN31105_c0_g1_i1 | blue | -0.531615 |
| TRINITY_DN38616_c1_g1_i2 | blue | -0.53379 |
| TRINITY_DN39321_c2_g8_i4 | blue | -0.545459 |
| TRINITY_DN30947_c0_g1_i3 | blue | -0.55273 |
| TRINITY_DN24809_c0_g1_i1 | blue | -0.553361 |
| TRINITY_DN19827_c0_g1_i1 | blue | -0.55441 |
| TRINITY_DN32272_c1_g2_i1 | blue | -0.55579 |
| TRINITY_DN18599_c0_g1_i1 | blue | -0.558689 |
| TRINITY_DN27543_c0_g1_i2 | blue | -0.559457 |
| TRINITY_DN28489_c0_g1_i4 | blue | -0.560011 |
| TRINITY_DN555_c0_g1_i1 | blue | -0.560564 |
| TRINITY_DN12263_c0_g1_i1 | blue | -0.560842 |
| TRINITY_DN2956_c0_g2_i1 | blue | -0.560876 |
| TRINITY_DN17163_c0_g1_i1 | blue | -0.562854 |
| TRINITY_DN26624_c0_g1_i1 | blue | -0.565188 |
| TRINITY_DN36775_c0_g2_i3 | blue | -0.566266 |
| TRINITY_DN67575_c0_g1_i1 | blue | -0.570181 |
| TRINITY_DN35751_c2_g3_i1 | blue | -0.570685 |
| TRINITY_DN27421_c0_g1_i2 | blue | -0.57102 |
| TRINITY_DN32403_c0_g2_i3 | blue | -0.572658 |
| TRINITY_DN67561_c0_g1_i1 | blue | -0.573102 |
| TRINITY_DN24286_c0_g1_i1 | blue | -0.57361 |
| TRINITY_DN28575_c0_g1_i3 | blue | -0.577292 |
| TRINITY_DN39230_c3_g5_i2 | blue | -0.578319 |
| TRINITY_DN67479_c0_g1_i1 | blue | -0.578717 |
| TRINITY_DN25775_c0_g1_i1 | blue | -0.578742 |
| TRINITY_DN37549_c0_g1_i4 | blue | -0.57969 |
| TRINITY_DN51793_c0_g1_i1 | blue | -0.581174 |
| TRINITY_DN35165_c0_g5_i4 | blue | -0.582162 |
| TRINITY_DN22436_c0_g1_i1 | blue | -0.583106 |
| TRINITY_DN19567_c0_g1_i1 | blue | -0.583355 |
| TRINITY_DN32805_c0_g2_i3 | blue | -0.583498 |
| TRINITY_DN29516_c0_g2_i10 | blue | -0.583746 |
| TRINITY_DN38643_c0_g2_i2 | blue | -0.584116 |
| TRINITY_DN30288_c0_g3_i1 | blue | -0.584384 |
| TRINITY_DN34705_c0_g2_i1 | blue | -0.585109 |
| TRINITY_DN26068_c0_g1_i1 | blue | -0.585137 |
| TRINITY_DN17897_c0_g1_i1 | blue | -0.586148 |
| TRINITY_DN52341_c0_g1_i1 | blue | -0.586253 |
| TRINITY_DN34065_c0_g1_i1 | blue | -0.587191 |
| TRINITY_DN26346_c0_g4_i1 | blue | -0.588632 |
| TRINITY_DN35374_c0_g6_i1 | blue | -0.591894 |
| TRINITY_DN37756_c1_g2_i2 | blue | -0.592722 |
| TRINITY_DN18507_c0_g2_i1 | blue | -0.593368 |
| TRINITY_DN30069_c0_g1_i3 | blue | -0.594478 |
| TRINITY_DN35165_c0_g6_i1 | blue | -0.594636 |
| TRINITY_DN62995_c0_g1_i1 | blue | -0.598749 |
| TRINITY_DN24808_c0_g1_i1 | blue | -0.599166 |
| TRINITY_DN21200_c0_g1_i1 | blue | -0.599185 |
| TRINITY_DN35904_c0_g2_i5 | blue | -0.600533 |
| TRINITY_DN26240_c0_g1_i3 | blue | -0.600658 |
| TRINITY_DN21770_c0_g1_i1 | blue | -0.6012 |
| TRINITY_DN25112_c0_g1_i2 | blue | -0.605047 |
| TRINITY_DN25264_c0_g2_i1 | blue | -0.605465 |
| TRINITY_DN18203_c0_g1_i1 | blue | -0.605671 |
| TRINITY_DN18329_c0_g3_i1 | blue | -0.60625 |
| TRINITY_DN29964_c0_g1_i1 | blue | -0.608339 |
| TRINITY_DN23689_c0_g1_i1 | blue | -0.608886 |
| TRINITY_DN32677_c1_g3_i1 | blue | -0.61034 |
| TRINITY_DN22982_c0_g1_i1 | blue | -0.610607 |
| TRINITY_DN36865_c3_g6_i3 | blue | -0.610724 |
| TRINITY_DN28686_c0_g1_i1 | blue | -0.611374 |
| TRINITY_DN32501_c0_g1_i1 | blue | -0.614463 |
| TRINITY_DN2602_c0_g1_i1 | blue | -0.615377 |
| TRINITY_DN38287_c0_g3_i2 | blue | -0.616954 |
| TRINITY_DN26458_c0_g2_i2 | blue | -0.617458 |
| TRINITY_DN12684_c0_g1_i1 | blue | -0.617482 |
| TRINITY_DN39285_c1_g4_i1 | blue | -0.618514 |
| TRINITY_DN32798_c0_g1_i1 | blue | -0.618705 |
| TRINITY_DN31267_c0_g2_i2 | blue | -0.620521 |
| TRINITY_DN13499_c0_g1_i1 | blue | -0.62088 |
| TRINITY_DN10015_c0_g1_i1 | blue | -0.621695 |
| TRINITY_DN23375_c0_g1_i1 | blue | -0.621975 |
| TRINITY_DN17858_c0_g1_i1 | blue | -0.624114 |
| TRINITY_DN16757_c0_g3_i1 | blue | -0.624755 |
| TRINITY_DN39995_c0_g1_i1 | blue | -0.627316 |
| TRINITY_DN34124_c0_g1_i1 | blue | -0.627485 |
| TRINITY_DN8445_c0_g2_i1 | blue | -0.627891 |
| TRINITY_DN23878_c0_g1_i1 | blue | -0.628418 |
| TRINITY_DN33897_c0_g2_i1 | blue | -0.628644 |
| TRINITY_DN22330_c0_g1_i1 | blue | -0.63021 |
| TRINITY_DN24340_c0_g1_i1 | blue | -0.630803 |
| TRINITY_DN20474_c0_g2_i1 | blue | -0.632502 |
| TRINITY_DN34639_c2_g3_i3 | blue | -0.633248 |
| TRINITY_DN9030_c0_g1_i1 | blue | -0.633302 |
| TRINITY_DN23685_c0_g1_i1 | blue | -0.637443 |
| TRINITY_DN17532_c0_g1_i1 | blue | -0.637762 |
| TRINITY_DN839_c0_g1_i1 | blue | -0.637937 |
| TRINITY_DN18127_c0_g2_i1 | blue | -0.638045 |
| TRINITY_DN69193_c0_g1_i1 | blue | -0.640925 |
| TRINITY_DN9556_c0_g1_i1 | blue | -0.641391 |
| TRINITY_DN26747_c0_g1_i3 | blue | -0.641475 |
| TRINITY_DN33719_c0_g1_i2 | blue | -0.641926 |
| TRINITY_DN20023_c0_g1_i1 | blue | -0.641938 |
| TRINITY_DN22156_c0_g1_i1 | blue | -0.642162 |
| TRINITY_DN51418_c0_g1_i1 | blue | -0.644333 |
| TRINITY_DN69154_c0_g1_i1 | blue | -0.644365 |
| TRINITY_DN46132_c0_g1_i1 | blue | -0.645764 |
| TRINITY_DN68370_c0_g1_i1 | blue | -0.645912 |
| TRINITY_DN24192_c0_g1_i2 | blue | -0.646181 |
| TRINITY_DN38860_c1_g5_i1 | blue | -0.646524 |
| TRINITY_DN39211_c3_g1_i2 | blue | -0.647781 |
| TRINITY_DN26346_c0_g3_i1 | blue | -0.648176 |
| TRINITY_DN52105_c0_g1_i1 | blue | -0.648298 |
| TRINITY_DN30314_c0_g1_i3 | blue | -0.64888 |
| TRINITY_DN67538_c0_g1_i1 | blue | -0.648929 |
| TRINITY_DN6991_c0_g1_i1 | blue | -0.649274 |
| TRINITY_DN36513_c0_g2_i1 | blue | -0.650005 |
| TRINITY_DN37991_c1_g2_i7 | blue | -0.650034 |
| TRINITY_DN25586_c0_g1_i1 | blue | -0.651095 |
| TRINITY_DN38891_c0_g1_i1 | blue | -0.651854 |
| TRINITY_DN68555_c0_g1_i1 | blue | -0.651908 |
| TRINITY_DN29237_c0_g1_i1 | blue | -0.652754 |
| TRINITY_DN18523_c0_g1_i1 | blue | -0.653299 |
| TRINITY_DN21582_c0_g3_i2 | blue | -0.653335 |
| TRINITY_DN30090_c0_g1_i2 | blue | -0.653586 |
| TRINITY_DN34623_c0_g1_i2 | blue | -0.653752 |
| TRINITY_DN31424_c0_g2_i2 | blue | -0.656864 |
| TRINITY_DN42579_c0_g1_i1 | blue | -0.656887 |
| TRINITY_DN6507_c0_g1_i1 | blue | -0.658309 |
| TRINITY_DN35945_c0_g2_i4 | blue | -0.659104 |
| TRINITY_DN8272_c0_g1_i1 | blue | -0.659106 |
| TRINITY_DN40055_c0_g1_i1 | blue | -0.660714 |
| TRINITY_DN28249_c0_g1_i3 | blue | -0.660817 |
| TRINITY_DN30834_c0_g1_i1 | blue | -0.661249 |
| TRINITY_DN29283_c0_g1_i3 | blue | -0.66176 |
| TRINITY_DN67757_c0_g1_i1 | blue | -0.662328 |
| TRINITY_DN18329_c0_g2_i1 | blue | -0.662428 |
| TRINITY_DN30961_c0_g1_i1 | blue | -0.6633 |
| TRINITY_DN65088_c0_g1_i1 | blue | -0.664393 |
| TRINITY_DN24094_c1_g3_i1 | blue | -0.664407 |
| TRINITY_DN62765_c0_g1_i1 | blue | -0.664794 |
| TRINITY_DN25033_c0_g2_i1 | blue | -0.665119 |
| TRINITY_DN19518_c0_g2_i1 | blue | -0.665156 |
| TRINITY_DN22354_c0_g1_i2 | blue | -0.666193 |
| TRINITY_DN38227_c0_g1_i8 | blue | -0.667487 |
| TRINITY_DN19325_c0_g1_i1 | blue | -0.667945 |
| TRINITY_DN24338_c0_g1_i1 | blue | -0.667973 |
| TRINITY_DN33011_c0_g1_i5 | blue | -0.668164 |
| TRINITY_DN13711_c0_g1_i1 | blue | -0.66922 |
| TRINITY_DN38875_c2_g2_i4 | blue | -0.670082 |
| TRINITY_DN35787_c1_g1_i3 | blue | -0.670704 |
| TRINITY_DN51259_c0_g1_i1 | blue | -0.671514 |
| TRINITY_DN23782_c0_g2_i1 | blue | -0.672838 |
| TRINITY_DN30557_c0_g2_i1 | blue | -0.673327 |
| TRINITY_DN17578_c0_g1_i1 | blue | -0.673341 |
| TRINITY_DN938_c0_g2_i1 | blue | -0.673644 |
| TRINITY_DN14995_c0_g2_i1 | blue | -0.674051 |
| TRINITY_DN57969_c0_g1_i1 | blue | -0.674171 |
| TRINITY_DN28080_c0_g2_i1 | blue | -0.67432 |
| TRINITY_DN30683_c0_g2_i4 | blue | -0.675326 |
| TRINITY_DN72156_c0_g1_i1 | blue | -0.675978 |
| TRINITY_DN11579_c0_g1_i1 | blue | -0.675986 |
| TRINITY_DN21199_c0_g3_i1 | blue | -0.676663 |
| TRINITY_DN25053_c0_g2_i1 | blue | -0.677581 |
| TRINITY_DN26097_c0_g1_i2 | blue | -0.677619 |
| TRINITY_DN20557_c0_g2_i1 | blue | -0.678334 |
| TRINITY_DN61926_c0_g1_i1 | blue | -0.67873 |
| TRINITY_DN26700_c0_g7_i1 | blue | -0.678824 |
| TRINITY_DN1907_c0_g1_i1 | blue | -0.67959 |
| TRINITY_DN28909_c0_g2_i1 | blue | -0.680167 |
| TRINITY_DN25662_c0_g2_i1 | blue | -0.680221 |
| TRINITY_DN50591_c0_g1_i1 | blue | -0.680276 |
| TRINITY_DN23405_c0_g1_i1 | blue | -0.68071 |
| TRINITY_DN37003_c0_g1_i4 | blue | -0.680789 |
| TRINITY_DN50625_c0_g1_i1 | blue | -0.68161 |
| TRINITY_DN13951_c0_g1_i1 | blue | -0.68217 |
| TRINITY_DN30120_c0_g1_i5 | blue | -0.682435 |
| TRINITY_DN11473_c0_g1_i1 | blue | -0.682568 |
| TRINITY_DN26137_c0_g1_i1 | blue | -0.682598 |
| TRINITY_DN37273_c0_g1_i1 | blue | -0.682708 |
| TRINITY_DN38649_c1_g5_i1 | blue | -0.68273 |
| TRINITY_DN17165_c0_g2_i1 | blue | -0.682927 |
| TRINITY_DN28962_c0_g1_i1 | blue | -0.683653 |
| TRINITY_DN29358_c0_g1_i4 | blue | -0.684092 |
| TRINITY_DN25756_c0_g2_i1 | blue | -0.68423 |
| TRINITY_DN35658_c0_g2_i2 | blue | -0.684636 |
| TRINITY_DN10721_c0_g1_i1 | blue | -0.684952 |
| TRINITY_DN19699_c0_g2_i1 | blue | -0.685187 |
| TRINITY_DN39870_c0_g1_i1 | blue | -0.685323 |
| TRINITY_DN10003_c0_g1_i1 | blue | -0.687315 |
| TRINITY_DN70861_c0_g1_i1 | blue | -0.687474 |
| TRINITY_DN39750_c0_g1_i1 | blue | -0.687982 |
| TRINITY_DN26694_c0_g1_i1 | blue | -0.688516 |
| TRINITY_DN17230_c0_g2_i1 | blue | -0.68865 |
| TRINITY_DN22695_c0_g2_i1 | blue | -0.688942 |
| TRINITY_DN59224_c0_g1_i1 | blue | -0.689026 |
| TRINITY_DN37262_c0_g6_i1 | blue | -0.68903 |
| TRINITY_DN36233_c0_g1_i2 | blue | -0.689141 |
| TRINITY_DN31132_c0_g1_i2 | blue | -0.689142 |
| TRINITY_DN38355_c0_g3_i1 | blue | -0.689496 |
| TRINITY_DN68925_c0_g1_i1 | blue | -0.691289 |
| TRINITY_DN39166_c1_g2_i3 | blue | -0.691328 |
| TRINITY_DN30070_c0_g1_i2 | blue | -0.692086 |
| TRINITY_DN67381_c0_g1_i1 | blue | -0.692139 |
| TRINITY_DN20984_c0_g1_i1 | blue | -0.692259 |
| TRINITY_DN70131_c0_g1_i1 | blue | -0.692803 |
| TRINITY_DN17193_c0_g1_i1 | blue | -0.693835 |
| TRINITY_DN27880_c0_g1_i2 | blue | -0.694157 |
| TRINITY_DN18515_c0_g2_i1 | blue | -0.694333 |
| TRINITY_DN31309_c0_g1_i1 | blue | -0.694956 |
| TRINITY_DN21646_c0_g1_i1 | blue | -0.695234 |
| TRINITY_DN34525_c0_g1_i2 | blue | -0.695464 |
| TRINITY_DN13520_c0_g1_i1 | blue | -0.695975 |
| TRINITY_DN35936_c0_g2_i2 | blue | -0.696179 |
| TRINITY_DN32125_c0_g10_i1 | blue | -0.696967 |
| TRINITY_DN13791_c0_g1_i1 | blue | -0.697047 |
| TRINITY_DN894_c0_g1_i1 | blue | -0.697293 |
| TRINITY_DN52863_c0_g1_i1 | blue | -0.69881 |
| TRINITY_DN2633_c0_g2_i1 | blue | -0.699829 |
| TRINITY_DN38975_c0_g1_i1 | blue | -0.700515 |
| TRINITY_DN28006_c0_g2_i2 | blue | -0.70086 |
| TRINITY_DN697_c0_g1_i1 | blue | -0.70092 |
| TRINITY_DN26286_c0_g1_i1 | blue | -0.701328 |
| TRINITY_DN23201_c0_g2_i1 | blue | -0.702183 |
| TRINITY_DN27463_c0_g1_i2 | blue | -0.702214 |
| TRINITY_DN50585_c0_g1_i1 | blue | -0.702517 |
| TRINITY_DN17843_c0_g2_i1 | blue | -0.702594 |
| TRINITY_DN4235_c0_g1_i1 | blue | -0.703193 |
| TRINITY_DN62485_c0_g1_i1 | blue | -0.7036 |
| TRINITY_DN38892_c1_g2_i1 | blue | -0.704535 |
| TRINITY_DN34618_c1_g5_i3 | blue | -0.704626 |
| TRINITY_DN38853_c1_g1_i1 | blue | -0.705691 |
| TRINITY_DN41973_c0_g1_i1 | blue | -0.706472 |
| TRINITY_DN23206_c0_g1_i1 | blue | -0.706991 |
| TRINITY_DN25476_c0_g1_i1 | blue | -0.70812 |
| TRINITY_DN26992_c0_g1_i3 | blue | -0.708817 |
| TRINITY_DN56640_c0_g1_i1 | blue | -0.708992 |
| TRINITY_DN55918_c0_g1_i1 | blue | -0.709889 |
| TRINITY_DN33709_c2_g3_i2 | blue | -0.709967 |
| TRINITY_DN25188_c0_g2_i1 | blue | -0.710526 |
| TRINITY_DN14335_c0_g1_i1 | blue | -0.710642 |
| TRINITY_DN39237_c5_g8_i1 | blue | -0.711158 |
| TRINITY_DN29733_c0_g1_i1 | blue | -0.711378 |
| TRINITY_DN24920_c0_g1_i4 | blue | -0.711452 |
| TRINITY_DN38698_c0_g2_i5 | blue | -0.712561 |
| TRINITY_DN29286_c0_g1_i1 | blue | -0.712615 |
| TRINITY_DN56267_c0_g1_i1 | blue | -0.71322 |
| TRINITY_DN20741_c0_g1_i1 | blue | -0.713352 |
| TRINITY_DN38045_c0_g1_i1 | blue | -0.71352 |
| TRINITY_DN39080_c1_g2_i2 | blue | -0.713628 |
| TRINITY_DN16807_c0_g1_i1 | blue | -0.715158 |
| TRINITY_DN35197_c1_g6_i1 | blue | -0.71516 |
| TRINITY_DN10174_c0_g1_i1 | blue | -0.715204 |
| TRINITY_DN36367_c0_g7_i1 | blue | -0.715559 |
| TRINITY_DN19300_c0_g1_i1 | blue | -0.715584 |
| TRINITY_DN39525_c0_g1_i1 | blue | -0.715991 |
| TRINITY_DN56706_c0_g1_i1 | blue | -0.716081 |
| TRINITY_DN38791_c3_g5_i2 | blue | -0.716726 |
| TRINITY_DN42233_c0_g1_i1 | blue | -0.71742 |
| TRINITY_DN63689_c0_g1_i1 | blue | -0.717482 |
| TRINITY_DN11363_c0_g2_i1 | blue | -0.717898 |
| TRINITY_DN20904_c0_g2_i1 | blue | -0.718302 |
| TRINITY_DN41254_c0_g1_i1 | blue | -0.718479 |
| TRINITY_DN44895_c0_g1_i1 | blue | -0.718557 |
| TRINITY_DN43350_c0_g1_i1 | blue | -0.718909 |
| TRINITY_DN15370_c0_g1_i1 | blue | -0.719503 |
| TRINITY_DN25839_c0_g1_i1 | blue | -0.720532 |
| TRINITY_DN8887_c0_g1_i1 | blue | -0.721058 |
| TRINITY_DN28054_c0_g7_i2 | blue | -0.721075 |
| TRINITY_DN36925_c0_g2_i1 | blue | -0.721865 |
| TRINITY_DN37815_c0_g1_i5 | blue | -0.722094 |
| TRINITY_DN70888_c0_g1_i1 | blue | -0.722197 |
| TRINITY_DN29667_c4_g6_i1 | blue | -0.722285 |
| TRINITY_DN63221_c0_g1_i1 | blue | -0.722829 |
| TRINITY_DN62365_c0_g1_i1 | blue | -0.723093 |
| TRINITY_DN56054_c0_g1_i1 | blue | -0.723116 |
| TRINITY_DN28054_c0_g4_i1 | blue | -0.723404 |
| TRINITY_DN57491_c0_g1_i1 | blue | -0.723902 |
| TRINITY_DN51120_c0_g1_i1 | blue | -0.724077 |
| TRINITY_DN26307_c0_g2_i2 | blue | -0.724247 |
| TRINITY_DN24579_c0_g2_i3 | blue | -0.724582 |
| TRINITY_DN23773_c0_g1_i1 | blue | -0.725195 |
| TRINITY_DN56113_c0_g1_i1 | blue | -0.725204 |
| TRINITY_DN9762_c0_g1_i1 | blue | -0.725225 |
| TRINITY_DN27856_c0_g2_i1 | blue | -0.726103 |
| TRINITY_DN36382_c0_g2_i1 | blue | -0.726215 |
| TRINITY_DN25781_c0_g1_i1 | blue | -0.726316 |
| TRINITY_DN39888_c0_g1_i1 | blue | -0.726635 |
| TRINITY_DN70843_c0_g1_i1 | blue | -0.727106 |
| TRINITY_DN4624_c0_g2_i1 | blue | -0.727341 |
| TRINITY_DN1658_c0_g1_i1 | blue | -0.727748 |
| TRINITY_DN24954_c0_g1_i1 | blue | -0.728134 |
| TRINITY_DN38279_c0_g4_i1 | blue | -0.728201 |
| TRINITY_DN33403_c0_g1_i2 | blue | -0.72837 |
| TRINITY_DN67868_c0_g1_i1 | blue | -0.729334 |
| TRINITY_DN290_c0_g1_i1 | blue | -0.73041 |
| TRINITY_DN38896_c2_g1_i4 | blue | -0.730631 |
| TRINITY_DN13380_c0_g1_i1 | blue | -0.73109 |
| TRINITY_DN20112_c0_g1_i1 | blue | -0.731574 |
| TRINITY_DN68503_c0_g1_i1 | blue | -0.732712 |
| TRINITY_DN20564_c0_g1_i2 | blue | -0.732836 |
| TRINITY_DN22621_c0_g2_i2 | blue | -0.733164 |
| TRINITY_DN54410_c0_g1_i1 | blue | -0.733844 |
| TRINITY_DN39180_c7_g13_i1 | blue | -0.73464 |
| TRINITY_DN62039_c0_g1_i1 | blue | -0.734884 |
| TRINITY_DN1855_c0_g1_i1 | blue | -0.735215 |
| TRINITY_DN19916_c0_g1_i1 | blue | -0.735372 |
| TRINITY_DN5845_c0_g1_i1 | blue | -0.735737 |
| TRINITY_DN22414_c0_g2_i1 | blue | -0.736423 |
| TRINITY_DN33881_c0_g1_i1 | blue | -0.736441 |
| TRINITY_DN38467_c0_g1_i1 | blue | -0.736469 |
| TRINITY_DN41567_c0_g1_i1 | blue | -0.736495 |
| TRINITY_DN22443_c0_g1_i1 | blue | -0.737387 |
| TRINITY_DN10452_c0_g1_i1 | blue | -0.737471 |
| TRINITY_DN38765_c0_g1_i1 | blue | -0.73768 |
| TRINITY_DN38979_c1_g1_i1 | blue | -0.737812 |
| TRINITY_DN26557_c0_g1_i1 | blue | -0.73848 |
| TRINITY_DN21641_c0_g1_i1 | blue | -0.738765 |
| TRINITY_DN26482_c0_g2_i2 | blue | -0.738822 |
| TRINITY_DN28991_c0_g1_i5 | blue | -0.739039 |
| TRINITY_DN16389_c0_g1_i1 | blue | -0.73937 |
| TRINITY_DN62456_c0_g1_i1 | blue | -0.74017 |
| TRINITY_DN31517_c0_g3_i3 | blue | -0.740874 |
| TRINITY_DN69830_c0_g1_i1 | blue | -0.74101 |
| TRINITY_DN4308_c0_g1_i1 | blue | -0.741056 |
| TRINITY_DN21834_c0_g1_i1 | blue | -0.741321 |
| TRINITY_DN284_c0_g1_i1 | blue | -0.74162 |
| TRINITY_DN39824_c0_g1_i1 | blue | -0.741973 |
| TRINITY_DN22366_c0_g2_i1 | blue | -0.742489 |
| TRINITY_DN39305_c3_g5_i1 | blue | -0.742819 |
| TRINITY_DN9175_c0_g1_i1 | blue | -0.743322 |
| TRINITY_DN4242_c0_g1_i1 | blue | -0.743347 |
| TRINITY_DN44932_c0_g1_i1 | blue | -0.744101 |
| TRINITY_DN70218_c0_g1_i1 | blue | -0.744169 |
| TRINITY_DN29913_c0_g1_i2 | blue | -0.744237 |
| TRINITY_DN63073_c0_g1_i1 | blue | -0.74426 |
| TRINITY_DN26484_c0_g1_i1 | blue | -0.744306 |
| TRINITY_DN11169_c0_g2_i1 | blue | -0.744339 |
| TRINITY_DN67737_c0_g1_i1 | blue | -0.744782 |
| TRINITY_DN32628_c0_g1_i1 | blue | -0.745032 |
| TRINITY_DN61477_c0_g1_i1 | blue | -0.745147 |
| TRINITY_DN23903_c0_g1_i1 | blue | -0.745554 |
| TRINITY_DN35147_c0_g1_i1 | blue | -0.745731 |
| TRINITY_DN12095_c0_g1_i1 | blue | -0.746585 |
| TRINITY_DN54236_c0_g1_i1 | blue | -0.746712 |
| TRINITY_DN31823_c0_g1_i19 | blue | -0.746924 |
| TRINITY_DN29441_c0_g2_i2 | blue | -0.747275 |
| TRINITY_DN39309_c2_g8_i1 | blue | -0.747291 |
| TRINITY_DN56389_c0_g1_i1 | blue | -0.747359 |
| TRINITY_DN36182_c0_g1_i1 | blue | -0.747459 |
| TRINITY_DN70732_c0_g1_i1 | blue | -0.748106 |
| TRINITY_DN22931_c0_g2_i1 | blue | -0.749217 |
| TRINITY_DN34540_c0_g4_i3 | blue | -0.749252 |
| TRINITY_DN78_c0_g2_i1 | blue | -0.749397 |
| TRINITY_DN32942_c0_g4_i4 | blue | -0.749574 |
| TRINITY_DN35318_c0_g1_i2 | blue | -0.750519 |
| TRINITY_DN18226_c0_g1_i1 | blue | -0.751017 |
| TRINITY_DN38686_c1_g1_i3 | blue | -0.751165 |
| TRINITY_DN33701_c1_g2_i2 | blue | -0.751279 |
| TRINITY_DN36243_c0_g2_i1 | blue | -0.751375 |
| TRINITY_DN33701_c1_g5_i3 | blue | -0.751441 |
| TRINITY_DN37120_c0_g1_i17 | blue | -0.751517 |
| TRINITY_DN39230_c3_g2_i1 | blue | -0.751802 |
| TRINITY_DN38948_c0_g3_i1 | blue | -0.752217 |
| TRINITY_DN16628_c0_g1_i1 | blue | -0.752265 |
| TRINITY_DN34132_c1_g1_i6 | blue | -0.752434 |
| TRINITY_DN38458_c1_g3_i1 | blue | -0.75264 |
| TRINITY_DN52016_c0_g1_i1 | blue | -0.752693 |
| TRINITY_DN68239_c0_g1_i1 | blue | -0.752833 |
| TRINITY_DN24313_c0_g3_i1 | blue | -0.752839 |
| TRINITY_DN55953_c0_g1_i1 | blue | -0.753146 |
| TRINITY_DN40988_c0_g1_i1 | blue | -0.753685 |
| TRINITY_DN62152_c0_g1_i1 | blue | -0.753832 |
| TRINITY_DN17367_c0_g3_i1 | blue | -0.753846 |
| TRINITY_DN6994_c0_g1_i1 | blue | -0.754023 |
| TRINITY_DN36039_c0_g1_i1 | blue | -0.754238 |
| TRINITY_DN24240_c0_g2_i1 | blue | -0.75433 |
| TRINITY_DN45721_c0_g1_i1 | blue | -0.754725 |
| TRINITY_DN27153_c0_g1_i2 | blue | -0.754835 |
| TRINITY_DN29787_c0_g1_i1 | blue | -0.754927 |
| TRINITY_DN32345_c0_g2_i1 | blue | -0.755054 |
| TRINITY_DN61779_c0_g1_i1 | blue | -0.755201 |
| TRINITY_DN35756_c1_g2_i1 | blue | -0.755576 |
| TRINITY_DN37366_c0_g1_i9 | blue | -0.75577 |
| TRINITY_DN69370_c0_g1_i1 | blue | -0.755955 |
| TRINITY_DN30719_c0_g1_i3 | blue | -0.756318 |
| TRINITY_DN11294_c0_g1_i1 | blue | -0.756348 |
| TRINITY_DN34162_c0_g1_i1 | blue | -0.757398 |
| TRINITY_DN21886_c0_g1_i1 | blue | -0.757527 |
| TRINITY_DN35300_c0_g4_i5 | blue | -0.757537 |
| TRINITY_DN19098_c0_g2_i1 | blue | -0.75803 |
| TRINITY_DN38687_c0_g1_i2 | blue | -0.758271 |
| TRINITY_DN21161_c0_g2_i2 | blue | -0.758373 |
| TRINITY_DN14015_c0_g2_i1 | blue | -0.758471 |
| TRINITY_DN27072_c0_g1_i1 | blue | -0.758803 |
| TRINITY_DN34886_c0_g1_i1 | blue | -0.75906 |
| TRINITY_DN69481_c0_g1_i1 | blue | -0.759157 |
| TRINITY_DN18736_c0_g2_i1 | blue | -0.75923 |
| TRINITY_DN29052_c0_g1_i5 | blue | -0.759247 |
| TRINITY_DN19125_c0_g2_i1 | blue | -0.759364 |
| TRINITY_DN25130_c0_g1_i1 | blue | -0.759687 |
| TRINITY_DN25203_c0_g1_i1 | blue | -0.759741 |
| TRINITY_DN28021_c0_g1_i1 | blue | -0.759929 |
| TRINITY_DN24226_c0_g2_i1 | blue | -0.760715 |
| TRINITY_DN18147_c0_g1_i1 | blue | -0.761065 |
| TRINITY_DN51042_c0_g1_i1 | blue | -0.762587 |
| TRINITY_DN68571_c0_g1_i1 | blue | -0.76299 |
| TRINITY_DN38606_c0_g3_i1 | blue | -0.763077 |
| TRINITY_DN30084_c0_g1_i2 | blue | -0.763086 |
| TRINITY_DN23740_c0_g1_i1 | blue | -0.7634 |
| TRINITY_DN30471_c0_g2_i3 | blue | -0.763451 |
| TRINITY_DN32337_c0_g2_i1 | blue | -0.763882 |
| TRINITY_DN26765_c0_g1_i1 | blue | -0.763943 |
| TRINITY_DN17217_c0_g1_i1 | blue | -0.763951 |
| TRINITY_DN35421_c1_g1_i5 | blue | -0.763968 |
| TRINITY_DN19933_c0_g1_i1 | blue | -0.764185 |
| TRINITY_DN9076_c0_g2_i1 | blue | -0.764222 |
| TRINITY_DN35676_c0_g2_i3 | blue | -0.76477 |
| TRINITY_DN21085_c0_g1_i1 | blue | -0.764854 |
| TRINITY_DN43386_c0_g1_i1 | blue | -0.764933 |
| TRINITY_DN35285_c0_g3_i1 | blue | -0.765309 |
| TRINITY_DN11957_c0_g2_i1 | blue | -0.765599 |
| TRINITY_DN30993_c0_g1_i1 | blue | -0.765647 |
| TRINITY_DN28678_c0_g1_i3 | blue | -0.765671 |
| TRINITY_DN29748_c1_g1_i5 | blue | -0.765977 |
| TRINITY_DN27072_c1_g1_i1 | blue | -0.767087 |
| TRINITY_DN31602_c0_g2_i1 | blue | -0.767142 |
| TRINITY_DN63476_c0_g1_i1 | blue | -0.767535 |
| TRINITY_DN20934_c0_g1_i1 | blue | -0.767587 |
| TRINITY_DN8925_c0_g1_i1 | blue | -0.768091 |
| TRINITY_DN16767_c0_g1_i1 | blue | -0.768104 |
| TRINITY_DN4255_c0_g1_i1 | blue | -0.768134 |
| TRINITY_DN25698_c0_g1_i1 | blue | -0.768263 |
| TRINITY_DN22751_c0_g1_i1 | blue | -0.768642 |
| TRINITY_DN8824_c0_g1_i1 | blue | -0.768933 |
| TRINITY_DN46230_c0_g1_i1 | blue | -0.768955 |
| TRINITY_DN28507_c0_g2_i1 | blue | -0.768971 |
| TRINITY_DN4170_c0_g1_i1 | blue | -0.76902 |
| TRINITY_DN39770_c0_g1_i1 | blue | -0.769134 |
| TRINITY_DN19202_c0_g1_i1 | blue | -0.76922 |
| TRINITY_DN23542_c0_g1_i1 | blue | -0.769297 |
| TRINITY_DN68311_c0_g1_i1 | blue | -0.769948 |
| TRINITY_DN41612_c0_g1_i1 | blue | -0.770125 |
| TRINITY_DN32882_c0_g1_i1 | blue | -0.77016 |
| TRINITY_DN28405_c0_g1_i1 | blue | -0.770167 |
| TRINITY_DN16581_c0_g3_i1 | blue | -0.77049 |
| TRINITY_DN13943_c0_g2_i1 | blue | -0.771484 |
| TRINITY_DN37647_c2_g2_i2 | blue | -0.7716 |
| TRINITY_DN45869_c0_g1_i1 | blue | -0.771744 |
| TRINITY_DN4952_c0_g1_i1 | blue | -0.771831 |
| TRINITY_DN29968_c2_g2_i1 | blue | -0.772188 |
| TRINITY_DN37268_c0_g2_i4 | blue | -0.772253 |
| TRINITY_DN32036_c0_g2_i2 | blue | -0.772624 |
| TRINITY_DN20687_c0_g1_i1 | blue | -0.772653 |
| TRINITY_DN37320_c0_g1_i12 | blue | -0.772922 |
| TRINITY_DN25638_c0_g1_i1 | blue | -0.773054 |
| TRINITY_DN45666_c0_g1_i1 | blue | -0.773718 |
| TRINITY_DN25157_c0_g1_i2 | blue | -0.775011 |
| TRINITY_DN44936_c0_g1_i1 | blue | -0.775738 |
| TRINITY_DN17286_c0_g1_i1 | blue | -0.775747 |
| TRINITY_DN70151_c0_g1_i1 | blue | -0.776112 |
| TRINITY_DN25219_c0_g1_i1 | blue | -0.777316 |
| TRINITY_DN18726_c0_g1_i1 | blue | -0.777737 |
| TRINITY_DN56481_c0_g1_i1 | blue | -0.777897 |
| TRINITY_DN34841_c2_g1_i4 | blue | -0.77825 |
| TRINITY_DN21320_c0_g1_i1 | blue | -0.77842 |
| TRINITY_DN18084_c0_g1_i1 | blue | -0.778511 |
| TRINITY_DN40250_c0_g1_i1 | blue | -0.778924 |
| TRINITY_DN47333_c0_g1_i1 | blue | -0.7791 |
| TRINITY_DN29982_c0_g1_i1 | blue | -0.779396 |
| TRINITY_DN39720_c0_g1_i1 | blue | -0.779537 |
| TRINITY_DN37870_c1_g9_i4 | blue | -0.779545 |
| TRINITY_DN57514_c0_g1_i1 | blue | -0.779691 |
| TRINITY_DN9070_c0_g1_i1 | blue | -0.779778 |
| TRINITY_DN33701_c1_g3_i4 | blue | -0.780256 |
| TRINITY_DN67854_c0_g1_i1 | blue | -0.781078 |
| TRINITY_DN37273_c3_g1_i1 | blue | -0.781249 |
| TRINITY_DN38195_c0_g2_i1 | blue | -0.782453 |
| TRINITY_DN24590_c0_g1_i2 | blue | -0.782803 |
| TRINITY_DN26187_c0_g1_i5 | blue | -0.782887 |
| TRINITY_DN38142_c1_g3_i1 | blue | -0.782893 |
| TRINITY_DN33825_c0_g1_i8 | blue | -0.783116 |
| TRINITY_DN49211_c0_g1_i1 | blue | -0.783296 |
| TRINITY_DN69375_c0_g1_i1 | blue | -0.783421 |
| TRINITY_DN46821_c0_g1_i1 | blue | -0.783447 |
| TRINITY_DN28511_c0_g1_i2 | blue | -0.783742 |
| TRINITY_DN27472_c0_g6_i1 | blue | -0.783945 |
| TRINITY_DN62786_c0_g1_i1 | blue | -0.783947 |
| TRINITY_DN253_c0_g1_i1 | blue | -0.784209 |
| TRINITY_DN9147_c0_g1_i1 | blue | -0.784318 |
| TRINITY_DN13376_c0_g1_i1 | blue | -0.784727 |
| TRINITY_DN17161_c0_g2_i1 | blue | -0.785726 |
| TRINITY_DN24707_c0_g1_i1 | blue | -0.786209 |
| TRINITY_DN17262_c0_g1_i1 | blue | -0.78624 |
| TRINITY_DN36367_c0_g2_i1 | blue | -0.786289 |
| TRINITY_DN56619_c0_g1_i1 | blue | -0.786487 |
| TRINITY_DN18665_c0_g2_i1 | blue | -0.787282 |
| TRINITY_DN57707_c0_g1_i1 | blue | -0.787808 |
| TRINITY_DN39190_c1_g2_i2 | blue | -0.788009 |
| TRINITY_DN33310_c0_g1_i1 | blue | -0.788137 |
| TRINITY_DN47542_c0_g1_i1 | blue | -0.788593 |
| TRINITY_DN2015_c0_g1_i1 | blue | -0.789031 |
| TRINITY_DN21552_c0_g1_i2 | blue | -0.789088 |
| TRINITY_DN62691_c0_g1_i1 | blue | -0.789206 |
| TRINITY_DN39249_c3_g1_i4 | blue | -0.789212 |
| TRINITY_DN11831_c0_g2_i1 | blue | -0.789684 |
| TRINITY_DN29966_c1_g4_i2 | blue | -0.789795 |
| TRINITY_DN8789_c0_g1_i1 | blue | -0.790021 |
| TRINITY_DN33320_c2_g1_i2 | blue | -0.790085 |
| TRINITY_DN27828_c0_g1_i1 | blue | -0.790161 |
| TRINITY_DN50941_c0_g1_i1 | blue | -0.790846 |
| TRINITY_DN34812_c1_g1_i1 | blue | -0.790941 |
| TRINITY_DN27464_c0_g2_i2 | blue | -0.791157 |
| TRINITY_DN62675_c0_g1_i1 | blue | -0.791689 |
| TRINITY_DN13562_c0_g1_i1 | blue | -0.791844 |
| TRINITY_DN35685_c1_g1_i3 | blue | -0.792301 |
| TRINITY_DN36974_c0_g1_i11 | blue | -0.792791 |
| TRINITY_DN32593_c0_g2_i4 | blue | -0.792863 |
| TRINITY_DN5208_c0_g1_i1 | blue | -0.792904 |
| TRINITY_DN39552_c0_g1_i1 | blue | -0.793151 |
| TRINITY_DN41164_c0_g1_i1 | blue | -0.793389 |
| TRINITY_DN3068_c0_g1_i1 | blue | -0.793526 |
| TRINITY_DN25047_c0_g2_i1 | blue | -0.793785 |
| TRINITY_DN38605_c1_g7_i1 | blue | -0.794161 |
| TRINITY_DN69916_c0_g1_i1 | blue | -0.794222 |
| TRINITY_DN68721_c0_g1_i1 | blue | -0.794475 |
| TRINITY_DN34685_c0_g1_i1 | blue | -0.794973 |
| TRINITY_DN25779_c0_g1_i1 | blue | -0.795916 |
| TRINITY_DN30905_c0_g2_i1 | blue | -0.795994 |
| TRINITY_DN58190_c0_g1_i1 | blue | -0.79615 |
| TRINITY_DN28795_c0_g1_i2 | blue | -0.79622 |
| TRINITY_DN38192_c1_g2_i1 | blue | -0.796254 |
| TRINITY_DN50430_c0_g1_i1 | blue | -0.796563 |
| TRINITY_DN51082_c0_g1_i1 | blue | -0.796646 |
| TRINITY_DN8616_c0_g2_i1 | blue | -0.796761 |
| TRINITY_DN4345_c0_g1_i1 | blue | -0.797011 |
| TRINITY_DN51462_c0_g1_i1 | blue | -0.797261 |
| TRINITY_DN33646_c0_g1_i2 | blue | -0.797385 |
| TRINITY_DN30626_c0_g1_i3 | blue | -0.797641 |
| TRINITY_DN62259_c0_g1_i1 | blue | -0.797643 |
| TRINITY_DN53137_c0_g1_i1 | blue | -0.797652 |
| TRINITY_DN61817_c0_g1_i1 | blue | -0.797783 |
| TRINITY_DN50611_c0_g1_i1 | blue | -0.798553 |
| TRINITY_DN70470_c0_g1_i1 | blue | -0.798609 |
| TRINITY_DN37570_c0_g2_i1 | blue | -0.798694 |
| TRINITY_DN32265_c1_g6_i2 | blue | -0.798922 |
| TRINITY_DN57197_c0_g1_i1 | blue | -0.798925 |
| TRINITY_DN70487_c0_g1_i1 | blue | -0.799036 |
| TRINITY_DN30810_c0_g2_i1 | blue | -0.799334 |
| TRINITY_DN63149_c0_g1_i1 | blue | -0.80008 |
| TRINITY_DN959_c0_g1_i1 | blue | -0.800294 |
| TRINITY_DN36557_c0_g1_i9 | blue | -0.800677 |
| TRINITY_DN907_c0_g1_i1 | blue | -0.801192 |
| TRINITY_DN20666_c0_g2_i1 | blue | -0.801436 |
| TRINITY_DN69237_c0_g1_i1 | blue | -0.801516 |
| TRINITY_DN39887_c0_g1_i1 | blue | -0.801547 |
| TRINITY_DN31938_c2_g2_i1 | blue | -0.801855 |
| TRINITY_DN40914_c0_g1_i1 | blue | -0.802052 |
| TRINITY_DN37918_c0_g3_i4 | blue | -0.802055 |
| TRINITY_DN21916_c0_g3_i1 | blue | -0.802236 |
| TRINITY_DN20195_c0_g1_i1 | blue | -0.802942 |
| TRINITY_DN22167_c0_g1_i1 | blue | -0.802942 |
| TRINITY_DN59236_c0_g1_i1 | blue | -0.802966 |
| TRINITY_DN56104_c0_g1_i1 | blue | -0.803002 |
| TRINITY_DN62097_c0_g1_i1 | blue | -0.803278 |
| TRINITY_DN26385_c0_g2_i1 | blue | -0.803351 |
| TRINITY_DN9763_c0_g1_i1 | blue | -0.803452 |
| TRINITY_DN56944_c0_g1_i1 | blue | -0.803538 |
| TRINITY_DN39990_c0_g1_i1 | blue | -0.803768 |
| TRINITY_DN62649_c0_g1_i1 | blue | -0.804222 |
| TRINITY_DN25810_c0_g1_i4 | blue | -0.804703 |
| TRINITY_DN35649_c1_g3_i1 | blue | -0.804727 |
| TRINITY_DN13611_c0_g1_i1 | blue | -0.804825 |
| TRINITY_DN34158_c0_g3_i1 | blue | -0.805213 |
| TRINITY_DN29348_c0_g1_i1 | blue | -0.805332 |
| TRINITY_DN29983_c0_g2_i1 | blue | -0.805631 |
| TRINITY_DN45265_c0_g1_i1 | blue | -0.805873 |
| TRINITY_DN38041_c0_g3_i1 | blue | -0.805875 |
| TRINITY_DN45323_c0_g1_i1 | blue | -0.805878 |
| TRINITY_DN16898_c0_g1_i1 | blue | -0.806256 |
| TRINITY_DN2509_c0_g1_i1 | blue | -0.806669 |
| TRINITY_DN57121_c0_g1_i1 | blue | -0.807068 |
| TRINITY_DN55941_c0_g1_i1 | blue | -0.807137 |
| TRINITY_DN31312_c0_g1_i8 | blue | -0.807288 |
| TRINITY_DN36631_c0_g4_i2 | blue | -0.807424 |
| TRINITY_DN24668_c0_g1_i1 | blue | -0.808077 |
| TRINITY_DN69601_c0_g1_i1 | blue | -0.808097 |
| TRINITY_DN8710_c0_g1_i1 | blue | -0.808231 |
| TRINITY_DN2367_c0_g1_i1 | blue | -0.808254 |
| TRINITY_DN25836_c0_g1_i3 | blue | -0.808448 |
| TRINITY_DN35278_c3_g1_i2 | blue | -0.808499 |
| TRINITY_DN36858_c0_g2_i21 | blue | -0.808811 |
| TRINITY_DN837_c0_g1_i1 | blue | -0.808884 |
| TRINITY_DN39318_c2_g1_i1 | blue | -0.809292 |
| TRINITY_DN68502_c0_g1_i1 | blue | -0.809567 |
| TRINITY_DN17234_c0_g2_i1 | blue | -0.809777 |
| TRINITY_DN32658_c0_g1_i2 | blue | -0.80984 |
| TRINITY_DN34581_c0_g3_i1 | blue | -0.810121 |
| TRINITY_DN63276_c0_g1_i1 | blue | -0.810325 |
| TRINITY_DN50597_c0_g1_i1 | blue | -0.810447 |
| TRINITY_DN45543_c0_g1_i1 | blue | -0.810644 |
| TRINITY_DN36041_c0_g1_i11 | blue | -0.810776 |
| TRINITY_DN30961_c0_g2_i1 | blue | -0.811131 |
| TRINITY_DN32038_c0_g2_i2 | blue | -0.811943 |
| TRINITY_DN29219_c0_g2_i4 | blue | -0.812054 |
| TRINITY_DN25858_c1_g2_i1 | blue | -0.812113 |
| TRINITY_DN33644_c1_g2_i1 | blue | -0.812197 |
| TRINITY_DN33335_c0_g1_i2 | blue | -0.81223 |
| TRINITY_DN29296_c0_g1_i1 | blue | -0.812894 |
| TRINITY_DN4361_c0_g1_i1 | blue | -0.813062 |
| TRINITY_DN56577_c0_g1_i1 | blue | -0.813699 |
| TRINITY_DN26186_c0_g1_i3 | blue | -0.813736 |
| TRINITY_DN71849_c0_g1_i1 | blue | -0.814018 |
| TRINITY_DN52883_c0_g1_i1 | blue | -0.814216 |
| TRINITY_DN13436_c0_g1_i1 | blue | -0.814312 |
| TRINITY_DN34158_c0_g1_i19 | blue | -0.814339 |
| TRINITY_DN13634_c0_g1_i1 | blue | -0.814416 |
| TRINITY_DN36318_c2_g3_i1 | blue | -0.814585 |
| TRINITY_DN46231_c0_g1_i1 | blue | -0.814632 |
| TRINITY_DN25977_c0_g1_i2 | blue | -0.814634 |
| TRINITY_DN36395_c5_g1_i1 | blue | -0.814887 |
| TRINITY_DN71200_c0_g1_i1 | blue | -0.814926 |
| TRINITY_DN28859_c0_g2_i1 | blue | -0.814962 |
| TRINITY_DN35623_c0_g2_i3 | blue | -0.815045 |
| TRINITY_DN16345_c0_g1_i1 | blue | -0.815587 |
| TRINITY_DN67497_c0_g1_i1 | blue | -0.815892 |
| TRINITY_DN1005_c0_g1_i1 | blue | -0.816018 |
| TRINITY_DN30452_c0_g1_i1 | blue | -0.816322 |
| TRINITY_DN32239_c2_g1_i4 | blue | -0.816522 |
| TRINITY_DN19913_c0_g3_i1 | blue | -0.816531 |
| TRINITY_DN25251_c0_g1_i1 | blue | -0.816672 |
| TRINITY_DN56087_c0_g1_i1 | blue | -0.816679 |
| TRINITY_DN35523_c0_g2_i1 | blue | -0.816706 |
| TRINITY_DN916_c0_g1_i1 | blue | -0.816937 |
| TRINITY_DN44930_c0_g1_i1 | blue | -0.817311 |
| TRINITY_DN29801_c0_g1_i3 | blue | -0.817484 |
| TRINITY_DN63493_c0_g1_i1 | blue | -0.817584 |
| TRINITY_DN38043_c0_g1_i1 | blue | -0.817644 |
| TRINITY_DN8432_c0_g1_i1 | blue | -0.817703 |
| TRINITY_DN3708_c0_g1_i1 | blue | -0.817725 |
| TRINITY_DN46308_c0_g1_i1 | blue | -0.817852 |
| TRINITY_DN17496_c0_g2_i1 | blue | -0.817976 |
| TRINITY_DN38951_c1_g9_i9 | blue | -0.818052 |
| TRINITY_DN13034_c0_g2_i1 | blue | -0.818146 |
| TRINITY_DN8475_c0_g1_i1 | blue | -0.818188 |
| TRINITY_DN36780_c0_g3_i1 | blue | -0.818237 |
| TRINITY_DN28673_c1_g1_i2 | blue | -0.818358 |
| TRINITY_DN67662_c0_g1_i1 | blue | -0.818392 |
| TRINITY_DN28029_c0_g6_i4 | blue | -0.818553 |
| TRINITY_DN45290_c0_g1_i1 | blue | -0.818798 |
| TRINITY_DN34532_c0_g3_i1 | blue | -0.818924 |
| TRINITY_DN18907_c0_g1_i1 | blue | -0.819299 |
| TRINITY_DN29380_c0_g1_i3 | blue | -0.819469 |
| TRINITY_DN21344_c0_g2_i1 | blue | -0.819761 |
| TRINITY_DN37606_c1_g2_i1 | blue | -0.820245 |
| TRINITY_DN12779_c0_g2_i1 | blue | -0.820878 |
| TRINITY_DN37547_c0_g1_i2 | blue | -0.821034 |
| TRINITY_DN24730_c0_g1_i2 | blue | -0.821656 |
| TRINITY_DN45118_c0_g1_i1 | blue | -0.822493 |
| TRINITY_DN24725_c0_g1_i1 | blue | -0.822576 |
| TRINITY_DN4323_c0_g2_i1 | blue | -0.822611 |
| TRINITY_DN17796_c0_g2_i1 | blue | -0.822723 |
| TRINITY_DN69006_c0_g1_i1 | blue | -0.82286 |
| TRINITY_DN63921_c0_g1_i1 | blue | -0.823054 |
| TRINITY_DN18024_c0_g1_i1 | blue | -0.82334 |
| TRINITY_DN47137_c0_g1_i1 | blue | -0.823613 |
| TRINITY_DN26018_c0_g1_i1 | blue | -0.823847 |
| TRINITY_DN21721_c0_g1_i1 | blue | -0.824041 |
| TRINITY_DN45828_c0_g1_i1 | blue | -0.824145 |
| TRINITY_DN40235_c0_g1_i1 | blue | -0.824606 |
| TRINITY_DN27175_c0_g1_i1 | blue | -0.824608 |
| TRINITY_DN22103_c0_g1_i1 | blue | -0.824931 |
| TRINITY_DN39102_c0_g1_i8 | blue | -0.825124 |
| TRINITY_DN8983_c0_g1_i1 | blue | -0.825483 |
| TRINITY_DN34441_c1_g2_i5 | blue | -0.826063 |
| TRINITY_DN38534_c2_g1_i1 | blue | -0.826089 |
| TRINITY_DN31982_c0_g1_i3 | blue | -0.826173 |
| TRINITY_DN29519_c0_g1_i2 | blue | -0.826223 |
| TRINITY_DN56753_c0_g1_i1 | blue | -0.82643 |
| TRINITY_DN18977_c0_g1_i1 | blue | -0.827097 |
| TRINITY_DN32406_c0_g2_i5 | blue | -0.827502 |
| TRINITY_DN26965_c0_g1_i3 | blue | -0.827893 |
| TRINITY_DN51246_c0_g1_i1 | blue | -0.827955 |
| TRINITY_DN50835_c0_g1_i1 | blue | -0.828091 |
| TRINITY_DN26521_c0_g1_i1 | blue | -0.828283 |
| TRINITY_DN39087_c0_g2_i5 | blue | -0.828359 |
| TRINITY_DN23222_c0_g1_i1 | blue | -0.828397 |
| TRINITY_DN45229_c0_g1_i1 | blue | -0.828864 |
| TRINITY_DN57425_c0_g1_i1 | blue | -0.829065 |
| TRINITY_DN26546_c0_g1_i2 | blue | -0.829183 |
| TRINITY_DN27776_c0_g1_i1 | blue | -0.829193 |
| TRINITY_DN39310_c0_g6_i1 | blue | -0.829217 |
| TRINITY_DN38151_c1_g2_i2 | blue | -0.829237 |
| TRINITY_DN29026_c0_g2_i1 | blue | -0.829309 |
| TRINITY_DN29242_c0_g1_i1 | blue | -0.829541 |
| TRINITY_DN6695_c0_g1_i1 | blue | -0.82971 |
| TRINITY_DN64341_c0_g1_i1 | blue | -0.829727 |
| TRINITY_DN23219_c0_g1_i1 | blue | -0.829983 |
| TRINITY_DN45366_c0_g1_i1 | blue | -0.830221 |
| TRINITY_DN2552_c0_g1_i1 | blue | -0.830361 |
| TRINITY_DN52304_c0_g1_i1 | blue | -0.830584 |
| TRINITY_DN67285_c0_g1_i1 | blue | -0.830649 |
| TRINITY_DN56487_c0_g1_i1 | blue | -0.830739 |
| TRINITY_DN32430_c0_g2_i6 | blue | -0.830882 |
| TRINITY_DN34608_c0_g1_i5 | blue | -0.831399 |
| TRINITY_DN68400_c0_g1_i1 | blue | -0.831576 |
| TRINITY_DN33835_c0_g2_i5 | blue | -0.831652 |
| TRINITY_DN33316_c1_g8_i1 | blue | -0.831703 |
| TRINITY_DN39264_c0_g1_i1 | blue | -0.831709 |
| TRINITY_DN16700_c0_g1_i1 | blue | -0.831883 |
| TRINITY_DN36762_c0_g1_i1 | blue | -0.832426 |
| TRINITY_DN45349_c0_g1_i1 | blue | -0.832959 |
| TRINITY_DN37201_c1_g1_i14 | blue | -0.833035 |
| TRINITY_DN305_c0_g1_i1 | blue | -0.833061 |
| TRINITY_DN12101_c0_g1_i1 | blue | -0.833414 |
| TRINITY_DN61771_c0_g1_i1 | blue | -0.833626 |
| TRINITY_DN11251_c0_g1_i1 | blue | -0.833725 |
| TRINITY_DN38809_c1_g2_i1 | blue | -0.833937 |
| TRINITY_DN38338_c1_g5_i9 | blue | -0.834443 |
| TRINITY_DN7268_c0_g1_i1 | blue | -0.835155 |
| TRINITY_DN39420_c0_g1_i1 | blue | -0.835272 |
| TRINITY_DN5810_c0_g1_i1 | blue | -0.836123 |
| TRINITY_DN35245_c0_g1_i4 | blue | -0.836277 |
| TRINITY_DN38807_c0_g5_i1 | blue | -0.836295 |
| TRINITY_DN45471_c0_g1_i1 | blue | -0.83642 |
| TRINITY_DN26307_c0_g1_i3 | blue | -0.836948 |
| TRINITY_DN30574_c0_g2_i1 | blue | -0.837418 |
| TRINITY_DN46212_c0_g1_i1 | blue | -0.837434 |
| TRINITY_DN3226_c0_g1_i1 | blue | -0.837442 |
| TRINITY_DN36234_c0_g4_i5 | blue | -0.837505 |
| TRINITY_DN30515_c0_g1_i2 | blue | -0.83761 |
| TRINITY_DN50505_c0_g1_i1 | blue | -0.838079 |
| TRINITY_DN62868_c0_g1_i1 | blue | -0.838254 |
| TRINITY_DN21759_c0_g1_i1 | blue | -0.838613 |
| TRINITY_DN31964_c0_g1_i2 | blue | -0.838822 |
| TRINITY_DN30100_c0_g3_i1 | blue | -0.838912 |
| TRINITY_DN58124_c0_g1_i1 | blue | -0.839819 |
| TRINITY_DN26155_c0_g1_i5 | blue | -0.839924 |
| TRINITY_DN15605_c0_g1_i1 | blue | -0.840118 |
| TRINITY_DN27739_c0_g1_i1 | blue | -0.840311 |
| TRINITY_DN36387_c0_g1_i7 | blue | -0.840439 |
| TRINITY_DN38308_c1_g1_i1 | blue | -0.841423 |
| TRINITY_DN37654_c0_g1_i2 | blue | -0.841666 |
| TRINITY_DN30443_c0_g1_i2 | blue | -0.842488 |
| TRINITY_DN34371_c2_g3_i1 | blue | -0.84267 |
| TRINITY_DN37293_c1_g1_i1 | blue | -0.842706 |
| TRINITY_DN26767_c0_g1_i1 | blue | -0.842822 |
| TRINITY_DN35283_c0_g4_i2 | blue | -0.842899 |
| TRINITY_DN9037_c0_g1_i1 | blue | -0.84305 |
| TRINITY_DN69141_c0_g1_i1 | blue | -0.843263 |
| TRINITY_DN30862_c0_g3_i2 | blue | -0.843565 |
| TRINITY_DN30116_c0_g1_i1 | blue | -0.843582 |
| TRINITY_DN58942_c0_g1_i1 | blue | -0.843845 |
| TRINITY_DN37714_c0_g2_i1 | blue | -0.84412 |
| TRINITY_DN23610_c0_g1_i1 | blue | -0.844202 |
| TRINITY_DN35428_c0_g6_i1 | blue | -0.844332 |
| TRINITY_DN35539_c1_g1_i2 | blue | -0.844757 |
| TRINITY_DN27023_c0_g1_i1 | blue | -0.844773 |
| TRINITY_DN62478_c0_g1_i1 | blue | -0.844828 |
| TRINITY_DN4379_c0_g2_i1 | blue | -0.844968 |
| TRINITY_DN33803_c1_g2_i1 | blue | -0.845031 |
| TRINITY_DN27729_c0_g1_i1 | blue | -0.84524 |
| TRINITY_DN24710_c0_g1_i1 | blue | -0.845709 |
| TRINITY_DN64704_c0_g1_i1 | blue | -0.846131 |
| TRINITY_DN27800_c0_g1_i1 | blue | -0.84653 |
| TRINITY_DN31222_c0_g2_i7 | blue | -0.84674 |
| TRINITY_DN25298_c0_g1_i1 | blue | -0.847406 |
| TRINITY_DN16357_c0_g2_i1 | blue | -0.847993 |
| TRINITY_DN13635_c0_g1_i1 | blue | -0.848299 |
| TRINITY_DN39847_c0_g1_i1 | blue | -0.848478 |
| TRINITY_DN24383_c0_g1_i1 | blue | -0.848596 |
| TRINITY_DN34526_c0_g1_i2 | blue | -0.848703 |
| TRINITY_DN24056_c0_g1_i1 | blue | -0.84886 |
| TRINITY_DN24607_c0_g1_i2 | blue | -0.8489 |
| TRINITY_DN36785_c1_g1_i5 | blue | -0.848949 |
| TRINITY_DN62411_c0_g1_i1 | blue | -0.849133 |
| TRINITY_DN51211_c0_g1_i1 | blue | -0.849526 |
| TRINITY_DN33834_c0_g1_i3 | blue | -0.849635 |
| TRINITY_DN68412_c0_g1_i1 | blue | -0.849654 |
| TRINITY_DN26915_c0_g2_i1 | blue | -0.849864 |
| TRINITY_DN36244_c1_g3_i1 | blue | -0.849926 |
| TRINITY_DN36414_c0_g2_i1 | blue | -0.850072 |
| TRINITY_DN35722_c0_g1_i1 | blue | -0.850105 |
| TRINITY_DN52120_c0_g1_i1 | blue | -0.850106 |
| TRINITY_DN29828_c0_g1_i1 | blue | -0.850325 |
| TRINITY_DN31364_c1_g1_i2 | blue | -0.850391 |
| TRINITY_DN37948_c1_g3_i1 | blue | -0.850444 |
| TRINITY_DN40351_c0_g1_i1 | blue | -0.850491 |
| TRINITY_DN35717_c0_g2_i3 | blue | -0.850703 |
| TRINITY_DN37338_c0_g1_i1 | blue | -0.850964 |
| TRINITY_DN18270_c0_g1_i1 | blue | -0.851668 |
| TRINITY_DN29575_c0_g1_i2 | blue | -0.851974 |
| TRINITY_DN45756_c0_g1_i1 | blue | -0.852181 |
| TRINITY_DN19364_c0_g1_i1 | blue | -0.852222 |
| TRINITY_DN68405_c0_g1_i1 | blue | -0.852504 |
| TRINITY_DN56724_c0_g1_i1 | blue | -0.852789 |
| TRINITY_DN31310_c0_g2_i3 | blue | -0.853087 |
| TRINITY_DN33646_c0_g2_i4 | blue | -0.853213 |
| TRINITY_DN33549_c0_g1_i5 | blue | -0.853537 |
| TRINITY_DN46468_c0_g1_i1 | blue | -0.853595 |
| TRINITY_DN62407_c0_g1_i1 | blue | -0.854073 |
| TRINITY_DN28807_c0_g1_i3 | blue | -0.854076 |
| TRINITY_DN36780_c0_g5_i3 | blue | -0.854523 |
| TRINITY_DN24741_c0_g1_i3 | blue | -0.854525 |
| TRINITY_DN50443_c0_g1_i1 | blue | -0.854588 |
| TRINITY_DN32849_c0_g1_i1 | blue | -0.854654 |
| TRINITY_DN16581_c0_g1_i1 | blue | -0.854676 |
| TRINITY_DN61695_c0_g1_i1 | blue | -0.854742 |
| TRINITY_DN45442_c0_g1_i1 | blue | -0.854852 |
| TRINITY_DN37373_c1_g1_i2 | blue | -0.854875 |
| TRINITY_DN36691_c1_g1_i1 | blue | -0.854969 |
| TRINITY_DN38014_c1_g1_i1 | blue | -0.855008 |
| TRINITY_DN35008_c1_g1_i1 | blue | -0.855174 |
| TRINITY_DN33138_c0_g1_i3 | blue | -0.855261 |
| TRINITY_DN46810_c0_g1_i1 | blue | -0.855569 |
| TRINITY_DN25793_c0_g1_i2 | blue | -0.855586 |
| TRINITY_DN38544_c0_g5_i1 | blue | -0.855749 |
| TRINITY_DN30331_c0_g1_i1 | blue | -0.856403 |
| TRINITY_DN25463_c0_g1_i2 | blue | -0.856482 |
| TRINITY_DN59152_c0_g1_i1 | blue | -0.856569 |
| TRINITY_DN21570_c0_g2_i1 | blue | -0.85672 |
| TRINITY_DN38237_c0_g4_i2 | blue | -0.857035 |
| TRINITY_DN30949_c0_g1_i6 | blue | -0.857267 |
| TRINITY_DN34175_c0_g1_i11 | blue | -0.857285 |
| TRINITY_DN39672_c0_g1_i1 | blue | -0.85732 |
| TRINITY_DN9788_c0_g1_i1 | blue | -0.857502 |
| TRINITY_DN38502_c0_g2_i1 | blue | -0.857761 |
| TRINITY_DN8363_c0_g1_i1 | blue | -0.857783 |
| TRINITY_DN38727_c0_g1_i14 | blue | -0.857949 |
| TRINITY_DN5684_c0_g1_i1 | blue | -0.858193 |
| TRINITY_DN26213_c0_g1_i3 | blue | -0.858295 |
| TRINITY_DN38809_c1_g1_i3 | blue | -0.858908 |
| TRINITY_DN32202_c0_g2_i1 | blue | -0.858969 |
| TRINITY_DN36419_c2_g1_i2 | blue | -0.859259 |
| TRINITY_DN33353_c0_g1_i1 | blue | -0.859349 |
| TRINITY_DN22052_c0_g4_i1 | blue | -0.859398 |
| TRINITY_DN51619_c0_g1_i1 | blue | -0.859728 |
| TRINITY_DN30853_c2_g1_i1 | blue | -0.859921 |
| TRINITY_DN37079_c0_g3_i4 | blue | -0.860182 |
| TRINITY_DN27067_c0_g1_i2 | blue | -0.860269 |
| TRINITY_DN29738_c0_g1_i3 | blue | -0.860934 |
| TRINITY_DN37333_c3_g3_i2 | blue | -0.861055 |
| TRINITY_DN39236_c0_g4_i2 | blue | -0.861177 |
| TRINITY_DN56483_c0_g1_i1 | blue | -0.861358 |
| TRINITY_DN63489_c0_g1_i1 | blue | -0.861546 |
| TRINITY_DN37249_c2_g1_i1 | blue | -0.861815 |
| TRINITY_DN27025_c0_g1_i1 | blue | -0.862148 |
| TRINITY_DN36915_c0_g1_i1 | blue | -0.862581 |
| TRINITY_DN67546_c0_g1_i1 | blue | -0.862977 |
| TRINITY_DN34785_c0_g1_i12 | blue | -0.863013 |
| TRINITY_DN31165_c0_g1_i3 | blue | -0.863146 |
| TRINITY_DN56746_c0_g1_i1 | blue | -0.863606 |
| TRINITY_DN207_c0_g1_i1 | blue | -0.86389 |
| TRINITY_DN45371_c0_g1_i1 | blue | -0.864104 |
| TRINITY_DN43253_c0_g1_i1 | blue | -0.864142 |
| TRINITY_DN41230_c0_g1_i1 | blue | -0.864431 |
| TRINITY_DN38598_c1_g1_i2 | blue | -0.864791 |
| TRINITY_DN35101_c0_g1_i8 | blue | -0.865017 |
| TRINITY_DN38103_c2_g1_i1 | blue | -0.865432 |
| TRINITY_DN31003_c0_g2_i11 | blue | -0.865473 |
| TRINITY_DN59386_c0_g1_i1 | blue | -0.865566 |
| TRINITY_DN69779_c0_g1_i1 | blue | -0.865969 |
| TRINITY_DN39024_c1_g1_i6 | blue | -0.866024 |
| TRINITY_DN29202_c0_g1_i2 | blue | -0.866028 |
| TRINITY_DN36335_c0_g1_i11 | blue | -0.866359 |
| TRINITY_DN34142_c0_g1_i9 | blue | -0.866797 |
| TRINITY_DN38969_c1_g1_i3 | blue | -0.867174 |
| TRINITY_DN36850_c0_g2_i18 | blue | -0.86747 |
| TRINITY_DN8025_c0_g1_i1 | blue | -0.867605 |
| TRINITY_DN33459_c0_g1_i2 | blue | -0.867959 |
| TRINITY_DN57067_c0_g1_i1 | blue | -0.868052 |
| TRINITY_DN38242_c1_g3_i3 | blue | -0.868205 |
| TRINITY_DN35030_c1_g7_i2 | blue | -0.868705 |
| TRINITY_DN36918_c0_g1_i15 | blue | -0.868736 |
| TRINITY_DN34659_c0_g1_i7 | blue | -0.869144 |
| TRINITY_DN33559_c0_g1_i2 | blue | -0.869249 |
| TRINITY_DN37664_c0_g7_i3 | blue | -0.869469 |
| TRINITY_DN37296_c1_g2_i6 | blue | -0.86965 |
| TRINITY_DN39305_c0_g1_i1 | blue | -0.869832 |
| TRINITY_DN68065_c0_g1_i1 | blue | -0.869837 |
| TRINITY_DN45302_c0_g1_i1 | blue | -0.870087 |
| TRINITY_DN37742_c0_g1_i1 | blue | -0.870288 |
| TRINITY_DN35883_c0_g3_i1 | blue | -0.870339 |
| TRINITY_DN37463_c0_g1_i19 | blue | -0.870688 |
| TRINITY_DN56354_c0_g1_i1 | blue | -0.870703 |
| TRINITY_DN50881_c0_g1_i1 | blue | -0.871665 |
| TRINITY_DN37568_c1_g3_i16 | blue | -0.871827 |
| TRINITY_DN31897_c0_g1_i1 | blue | -0.87201 |
| TRINITY_DN71014_c0_g1_i1 | blue | -0.872483 |
| TRINITY_DN31294_c1_g1_i3 | blue | -0.872588 |
| TRINITY_DN26332_c0_g2_i2 | blue | -0.872675 |
| TRINITY_DN37300_c0_g3_i2 | blue | -0.873442 |
| TRINITY_DN23410_c0_g2_i1 | blue | -0.873464 |
| TRINITY_DN35395_c0_g1_i12 | blue | -0.873698 |
| TRINITY_DN32259_c0_g1_i1 | blue | -0.87398 |
| TRINITY_DN34589_c0_g2_i1 | blue | -0.874345 |
| TRINITY_DN38542_c0_g2_i3 | blue | -0.874622 |
| TRINITY_DN23052_c0_g1_i1 | blue | -0.874938 |
| TRINITY_DN28796_c0_g1_i1 | blue | -0.875203 |
| TRINITY_DN40632_c0_g1_i1 | blue | -0.875406 |
| TRINITY_DN11831_c0_g1_i1 | blue | -0.875437 |
| TRINITY_DN33116_c1_g2_i3 | blue | -0.875488 |
| TRINITY_DN39116_c2_g1_i2 | blue | -0.875561 |
| TRINITY_DN35051_c0_g4_i3 | blue | -0.875649 |
| TRINITY_DN57017_c0_g1_i1 | blue | -0.875663 |
| TRINITY_DN57753_c0_g1_i1 | blue | -0.875729 |
| TRINITY_DN63822_c0_g1_i1 | blue | -0.876101 |
| TRINITY_DN61039_c0_g1_i1 | blue | -0.876266 |
| TRINITY_DN34760_c1_g1_i6 | blue | -0.876281 |
| TRINITY_DN62185_c0_g1_i1 | blue | -0.876305 |
| TRINITY_DN34547_c0_g3_i4 | blue | -0.876504 |
| TRINITY_DN16454_c0_g2_i1 | blue | -0.876551 |
| TRINITY_DN61994_c0_g1_i1 | blue | -0.876986 |
| TRINITY_DN16876_c0_g1_i1 | blue | -0.877232 |
| TRINITY_DN31527_c0_g1_i2 | blue | -0.877538 |
| TRINITY_DN5410_c0_g1_i1 | blue | -0.877726 |
| TRINITY_DN32138_c0_g1_i2 | blue | -0.878069 |
| TRINITY_DN26147_c0_g2_i1 | blue | -0.878467 |
| TRINITY_DN30175_c0_g1_i2 | blue | -0.879218 |
| TRINITY_DN51387_c0_g1_i1 | blue | -0.879888 |
| TRINITY_DN38242_c1_g1_i6 | blue | -0.879919 |
| TRINITY_DN25671_c0_g2_i1 | blue | -0.879998 |
| TRINITY_DN29645_c2_g2_i5 | blue | -0.880143 |
| TRINITY_DN34982_c0_g1_i7 | blue | -0.880146 |
| TRINITY_DN1483_c0_g1_i1 | blue | -0.880354 |
| TRINITY_DN20857_c0_g2_i1 | blue | -0.880381 |
| TRINITY_DN36085_c0_g3_i1 | blue | -0.880505 |
| TRINITY_DN25242_c0_g1_i1 | blue | -0.880933 |
| TRINITY_DN37858_c0_g1_i1 | blue | -0.880964 |
| TRINITY_DN37219_c0_g2_i4 | blue | -0.881208 |
| TRINITY_DN35467_c0_g1_i2 | blue | -0.881486 |
| TRINITY_DN23611_c0_g1_i1 | blue | -0.881686 |
| TRINITY_DN42261_c0_g1_i1 | blue | -0.881706 |
| TRINITY_DN38967_c2_g7_i1 | blue | -0.881835 |
| TRINITY_DN38204_c0_g2_i8 | blue | -0.88195 |
| TRINITY_DN38649_c1_g2_i1 | blue | -0.881959 |
| TRINITY_DN29189_c0_g3_i1 | blue | -0.882131 |
| TRINITY_DN30660_c0_g1_i1 | blue | -0.882186 |
| TRINITY_DN37675_c1_g2_i18 | blue | -0.88226 |
| TRINITY_DN13589_c0_g1_i1 | blue | -0.882329 |
| TRINITY_DN36166_c0_g1_i1 | blue | -0.882431 |
| TRINITY_DN61992_c0_g1_i1 | blue | -0.882503 |
| TRINITY_DN26672_c0_g1_i1 | blue | -0.883979 |
| TRINITY_DN31846_c0_g2_i2 | blue | -0.884619 |
| TRINITY_DN36251_c0_g1_i8 | blue | -0.884623 |
| TRINITY_DN40657_c0_g1_i1 | blue | -0.884865 |
| TRINITY_DN33679_c1_g3_i8 | blue | -0.885043 |
| TRINITY_DN38435_c0_g4_i1 | blue | -0.885051 |
| TRINITY_DN36447_c2_g1_i3 | blue | -0.885988 |
| TRINITY_DN32269_c0_g1_i4 | blue | -0.886177 |
| TRINITY_DN27331_c1_g2_i1 | blue | -0.886248 |
| TRINITY_DN35713_c0_g1_i15 | blue | -0.886314 |
| TRINITY_DN31799_c0_g2_i11 | blue | -0.886316 |
| TRINITY_DN19046_c0_g1_i1 | blue | -0.886505 |
| TRINITY_DN26000_c1_g2_i1 | blue | -0.887086 |
| TRINITY_DN4203_c0_g3_i1 | blue | -0.887238 |
| TRINITY_DN24673_c0_g2_i1 | blue | -0.887241 |
| TRINITY_DN34706_c0_g2_i2 | blue | -0.887361 |
| TRINITY_DN35048_c0_g1_i8 | blue | -0.887488 |
| TRINITY_DN40315_c0_g1_i1 | blue | -0.887635 |
| TRINITY_DN29761_c0_g1_i1 | blue | -0.88776 |
| TRINITY_DN36782_c4_g2_i1 | blue | -0.888046 |
| TRINITY_DN34795_c0_g2_i3 | blue | -0.888706 |
| TRINITY_DN31907_c0_g2_i1 | blue | -0.888856 |
| TRINITY_DN33038_c0_g1_i1 | blue | -0.888882 |
| TRINITY_DN28231_c0_g1_i3 | blue | -0.889014 |
| TRINITY_DN19634_c0_g1_i2 | blue | -0.889131 |
| TRINITY_DN67317_c0_g1_i1 | blue | -0.889515 |
| TRINITY_DN24619_c0_g1_i2 | blue | -0.889658 |
| TRINITY_DN29592_c0_g1_i2 | blue | -0.889724 |
| TRINITY_DN24250_c0_g1_i2 | blue | -0.890342 |
| TRINITY_DN25409_c0_g1_i2 | blue | -0.891053 |
| TRINITY_DN15585_c0_g1_i1 | blue | -0.891711 |
| TRINITY_DN67970_c0_g1_i1 | blue | -0.89178 |
| TRINITY_DN1001_c0_g1_i1 | blue | -0.891992 |
| TRINITY_DN27814_c0_g1_i3 | blue | -0.892016 |
| TRINITY_DN44993_c0_g1_i1 | blue | -0.892022 |
| TRINITY_DN7655_c0_g1_i1 | blue | -0.892214 |
| TRINITY_DN52475_c0_g1_i1 | blue | -0.892302 |
| TRINITY_DN36034_c1_g2_i3 | blue | -0.892719 |
| TRINITY_DN37527_c2_g2_i14 | blue | -0.892725 |
| TRINITY_DN36457_c1_g1_i1 | blue | -0.892799 |
| TRINITY_DN23508_c0_g2_i2 | blue | -0.892859 |
| TRINITY_DN36244_c1_g2_i4 | blue | -0.892956 |
| TRINITY_DN36987_c0_g1_i20 | blue | -0.893033 |
| TRINITY_DN38625_c0_g1_i1 | blue | -0.893062 |
| TRINITY_DN67436_c0_g1_i1 | blue | -0.893126 |
| TRINITY_DN37041_c0_g1_i1 | blue | -0.893579 |
| TRINITY_DN40430_c0_g1_i1 | blue | -0.894699 |
| TRINITY_DN38909_c1_g1_i9 | blue | -0.894986 |
| TRINITY_DN16539_c0_g1_i1 | blue | -0.895235 |
| TRINITY_DN34589_c0_g1_i2 | blue | -0.895837 |
| TRINITY_DN36940_c0_g1_i17 | blue | -0.895929 |
| TRINITY_DN35952_c1_g1_i4 | blue | -0.896484 |
| TRINITY_DN58847_c0_g1_i1 | blue | -0.896562 |
| TRINITY_DN30968_c0_g2_i1 | blue | -0.896811 |
| TRINITY_DN10085_c0_g1_i1 | blue | -0.896861 |
| TRINITY_DN37247_c0_g2_i20 | blue | -0.897288 |
| TRINITY_DN38155_c0_g1_i7 | blue | -0.897339 |
| TRINITY_DN17587_c0_g1_i1 | blue | -0.897411 |
| TRINITY_DN25622_c0_g1_i1 | blue | -0.897635 |
| TRINITY_DN16979_c0_g1_i1 | blue | -0.898037 |
| TRINITY_DN36509_c0_g3_i3 | blue | -0.898204 |
| TRINITY_DN67720_c0_g1_i1 | blue | -0.898328 |
| TRINITY_DN24039_c0_g1_i1 | blue | -0.898355 |
| TRINITY_DN22789_c0_g1_i1 | blue | -0.89845 |
| TRINITY_DN26428_c0_g3_i2 | blue | -0.898995 |
| TRINITY_DN33427_c0_g1_i5 | blue | -0.899142 |
| TRINITY_DN27095_c0_g1_i3 | blue | -0.899239 |
| TRINITY_DN35129_c0_g1_i11 | blue | -0.899321 |
| TRINITY_DN28188_c0_g1_i1 | blue | -0.899336 |
| TRINITY_DN33495_c0_g1_i15 | blue | -0.899363 |
| TRINITY_DN19647_c0_g2_i1 | blue | -0.899909 |
| TRINITY_DN30533_c0_g1_i5 | blue | -0.900021 |
| TRINITY_DN61864_c0_g1_i1 | blue | -0.900066 |
| TRINITY_DN35335_c2_g1_i4 | blue | -0.900306 |
| TRINITY_DN38901_c0_g1_i2 | blue | -0.900629 |
| TRINITY_DN28057_c0_g1_i1 | blue | -0.900871 |
| TRINITY_DN37569_c0_g7_i3 | blue | -0.901454 |
| TRINITY_DN29783_c1_g3_i2 | blue | -0.901933 |
| TRINITY_DN36474_c0_g1_i1 | blue | -0.901941 |
| TRINITY_DN34338_c0_g2_i1 | blue | -0.902076 |
| TRINITY_DN26259_c0_g1_i1 | blue | -0.902081 |
| TRINITY_DN33157_c0_g1_i1 | blue | -0.902222 |
| TRINITY_DN37660_c0_g1_i4 | blue | -0.902298 |
| TRINITY_DN27356_c0_g2_i1 | blue | -0.902357 |
| TRINITY_DN23600_c0_g1_i2 | blue | -0.902695 |
| TRINITY_DN24363_c0_g1_i1 | blue | -0.902699 |
| TRINITY_DN36801_c0_g1_i7 | blue | -0.902882 |
| TRINITY_DN31057_c0_g1_i1 | blue | -0.903397 |
| TRINITY_DN67420_c0_g1_i1 | blue | -0.904035 |
| TRINITY_DN36008_c0_g3_i1 | blue | -0.904812 |
| TRINITY_DN31887_c0_g1_i13 | blue | -0.904875 |
| TRINITY_DN34529_c0_g1_i17 | blue | -0.905499 |
| TRINITY_DN30155_c0_g1_i2 | blue | -0.905529 |
| TRINITY_DN27714_c0_g2_i2 | blue | -0.906092 |
| TRINITY_DN34674_c0_g1_i6 | blue | -0.90629 |
| TRINITY_DN32875_c0_g1_i4 | blue | -0.906435 |
| TRINITY_DN35395_c0_g2_i1 | blue | -0.907162 |
| TRINITY_DN23072_c0_g1_i1 | blue | -0.907212 |
| TRINITY_DN35046_c0_g3_i1 | blue | -0.907746 |
| TRINITY_DN16827_c0_g1_i1 | blue | -0.907825 |
| TRINITY_DN40624_c0_g1_i1 | blue | -0.90796 |
| TRINITY_DN29560_c0_g1_i7 | blue | -0.908214 |
| TRINITY_DN17136_c0_g1_i2 | blue | -0.908479 |
| TRINITY_DN29481_c0_g1_i1 | blue | -0.908494 |
| TRINITY_DN31873_c0_g1_i2 | blue | -0.90872 |
| TRINITY_DN28860_c0_g2_i1 | blue | -0.909124 |
| TRINITY_DN35378_c0_g1_i3 | blue | -0.909169 |
| TRINITY_DN35860_c3_g2_i1 | blue | -0.909181 |
| TRINITY_DN32172_c0_g1_i5 | blue | -0.909217 |
| TRINITY_DN29263_c0_g1_i6 | blue | -0.909356 |
| TRINITY_DN38589_c0_g4_i2 | blue | -0.909411 |
| TRINITY_DN64497_c0_g1_i1 | blue | -0.909644 |
| TRINITY_DN35880_c0_g1_i12 | blue | -0.9097 |
| TRINITY_DN31517_c0_g1_i8 | blue | -0.909979 |
| TRINITY_DN34841_c2_g2_i3 | blue | -0.910165 |
| TRINITY_DN39048_c0_g1_i13 | blue | -0.910325 |
| TRINITY_DN39146_c1_g2_i15 | blue | -0.910369 |
| TRINITY_DN36850_c0_g1_i10 | blue | -0.910429 |
| TRINITY_DN37160_c1_g3_i2 | blue | -0.910865 |
| TRINITY_DN41171_c0_g1_i1 | blue | -0.91099 |
| TRINITY_DN35445_c0_g3_i17 | blue | -0.911028 |
| TRINITY_DN25154_c0_g1_i2 | blue | -0.911094 |
| TRINITY_DN38821_c3_g2_i3 | blue | -0.911234 |
| TRINITY_DN38429_c16_g1_i1 | blue | -0.911384 |
| TRINITY_DN34431_c0_g1_i1 | blue | -0.911465 |
| TRINITY_DN67519_c0_g1_i1 | blue | -0.911468 |
| TRINITY_DN26968_c0_g1_i1 | blue | -0.911716 |
| TRINITY_DN67934_c0_g1_i1 | blue | -0.911828 |
| TRINITY_DN63215_c0_g1_i1 | blue | -0.912027 |
| TRINITY_DN22169_c0_g1_i1 | blue | -0.912503 |
| TRINITY_DN36572_c0_g2_i3 | blue | -0.912619 |
| TRINITY_DN37238_c1_g1_i4 | blue | -0.913091 |
| TRINITY_DN38575_c0_g2_i2 | blue | -0.913097 |
| TRINITY_DN38966_c0_g1_i4 | blue | -0.913781 |
| TRINITY_DN35163_c2_g4_i1 | blue | -0.91412 |
| TRINITY_DN31916_c0_g1_i2 | blue | -0.914187 |
| TRINITY_DN26793_c0_g1_i4 | blue | -0.914703 |
| TRINITY_DN46573_c0_g1_i1 | blue | -0.914861 |
| TRINITY_DN38728_c0_g3_i3 | blue | -0.915106 |
| TRINITY_DN33136_c1_g1_i1 | blue | -0.915135 |
| TRINITY_DN37624_c0_g3_i1 | blue | -0.915219 |
| TRINITY_DN38722_c1_g1_i6 | blue | -0.916173 |
| TRINITY_DN35490_c0_g5_i3 | blue | -0.916286 |
| TRINITY_DN32072_c1_g1_i2 | blue | -0.916742 |
| TRINITY_DN33836_c0_g1_i9 | blue | -0.916759 |
| TRINITY_DN35160_c0_g1_i3 | blue | -0.917049 |
| TRINITY_DN30944_c2_g2_i2 | blue | -0.917059 |
| TRINITY_DN39737_c0_g1_i1 | blue | -0.917111 |
| TRINITY_DN38813_c0_g1_i6 | blue | -0.917205 |
| TRINITY_DN38415_c1_g4_i3 | blue | -0.917858 |
| TRINITY_DN17134_c0_g4_i1 | blue | -0.918131 |
| TRINITY_DN31291_c0_g1_i13 | blue | -0.918384 |
| TRINITY_DN35281_c0_g1_i3 | blue | -0.918402 |
| TRINITY_DN11190_c0_g1_i1 | blue | -0.918965 |
| TRINITY_DN67554_c0_g1_i1 | blue | -0.919059 |
| TRINITY_DN35238_c0_g1_i7 | blue | -0.919397 |
| TRINITY_DN35377_c0_g2_i1 | blue | -0.919441 |
| TRINITY_DN38728_c0_g4_i5 | blue | -0.919612 |
| TRINITY_DN36103_c0_g1_i1 | blue | -0.92039 |
| TRINITY_DN28617_c1_g1_i2 | blue | -0.920413 |
| TRINITY_DN36625_c0_g1_i5 | blue | -0.920414 |
| TRINITY_DN31975_c0_g1_i4 | blue | -0.921011 |
| TRINITY_DN37558_c0_g1_i1 | blue | -0.921226 |
| TRINITY_DN34152_c0_g2_i2 | blue | -0.921237 |
| TRINITY_DN28243_c0_g1_i2 | blue | -0.921355 |
| TRINITY_DN39098_c2_g1_i16 | blue | -0.921401 |
| TRINITY_DN31430_c0_g3_i5 | blue | -0.921559 |
| TRINITY_DN37195_c0_g3_i1 | blue | -0.921763 |
| TRINITY_DN36506_c0_g1_i3 | blue | -0.922009 |
| TRINITY_DN31115_c0_g2_i2 | blue | -0.922104 |
| TRINITY_DN28670_c1_g3_i1 | blue | -0.922236 |
| TRINITY_DN38651_c0_g1_i4 | blue | -0.92233 |
| TRINITY_DN32241_c0_g1_i1 | blue | -0.92233 |
| TRINITY_DN37606_c1_g4_i15 | blue | -0.922601 |
| TRINITY_DN30074_c1_g9_i1 | blue | -0.922711 |
| TRINITY_DN32969_c0_g1_i5 | blue | -0.922763 |
| TRINITY_DN34761_c1_g2_i2 | blue | -0.922882 |
| TRINITY_DN36625_c0_g3_i3 | blue | -0.923121 |
| TRINITY_DN38539_c0_g1_i4 | blue | -0.923161 |
| TRINITY_DN10390_c0_g1_i1 | blue | -0.92333 |
| TRINITY_DN34051_c0_g1_i7 | blue | -0.923352 |
| TRINITY_DN34308_c0_g1_i1 | blue | -0.923516 |
| TRINITY_DN30757_c1_g1_i2 | blue | -0.924079 |
| TRINITY_DN31040_c0_g1_i4 | blue | -0.924186 |
| TRINITY_DN17032_c0_g1_i1 | blue | -0.92422 |
| TRINITY_DN37152_c0_g2_i2 | blue | -0.924448 |
| TRINITY_DN37505_c0_g3_i1 | blue | -0.924471 |
| TRINITY_DN37226_c0_g3_i18 | blue | -0.924668 |
| TRINITY_DN38128_c1_g1_i10 | blue | -0.924789 |
| TRINITY_DN39170_c0_g1_i8 | blue | -0.925042 |
| TRINITY_DN27053_c0_g2_i1 | blue | -0.92527 |
| TRINITY_DN37570_c0_g1_i3 | blue | -0.92528 |
| TRINITY_DN35340_c1_g3_i1 | blue | -0.92533 |
| TRINITY_DN30724_c0_g1_i2 | blue | -0.925904 |
| TRINITY_DN32621_c0_g1_i3 | blue | -0.926392 |
| TRINITY_DN25227_c0_g2_i1 | blue | -0.926582 |
| TRINITY_DN39068_c0_g19_i1 | blue | -0.926778 |
| TRINITY_DN37839_c2_g4_i2 | blue | -0.926871 |
| TRINITY_DN35102_c0_g1_i7 | blue | -0.926992 |
| TRINITY_DN37425_c0_g1_i2 | blue | -0.927092 |
| TRINITY_DN36593_c0_g4_i1 | blue | -0.927627 |
| TRINITY_DN37222_c0_g1_i1 | blue | -0.927912 |
| TRINITY_DN36838_c0_g1_i5 | blue | -0.927953 |
| TRINITY_DN35471_c0_g2_i1 | blue | -0.928094 |
| TRINITY_DN32178_c0_g2_i2 | blue | -0.928101 |
| TRINITY_DN36726_c0_g3_i2 | blue | -0.928118 |
| TRINITY_DN38908_c0_g1_i1 | blue | -0.928287 |
| TRINITY_DN29726_c1_g2_i2 | blue | -0.928592 |
| TRINITY_DN37750_c0_g1_i3 | blue | -0.928847 |
| TRINITY_DN35063_c2_g4_i5 | blue | -0.929161 |
| TRINITY_DN36115_c2_g1_i8 | blue | -0.930402 |
| TRINITY_DN39314_c1_g1_i1 | blue | -0.93049 |
| TRINITY_DN39247_c1_g4_i10 | blue | -0.930529 |
| TRINITY_DN40744_c0_g1_i1 | blue | -0.930562 |
| TRINITY_DN33514_c0_g5_i1 | blue | -0.931595 |
| TRINITY_DN39224_c0_g3_i4 | blue | -0.931947 |
| TRINITY_DN33117_c0_g2_i2 | blue | -0.932238 |
| TRINITY_DN34732_c1_g1_i4 | blue | -0.932299 |
| TRINITY_DN32354_c0_g1_i1 | blue | -0.932669 |
| TRINITY_DN35731_c1_g1_i11 | blue | -0.932784 |
| TRINITY_DN30157_c0_g1_i5 | blue | -0.933205 |
| TRINITY_DN37600_c1_g4_i3 | blue | -0.933295 |
| TRINITY_DN34124_c1_g6_i8 | blue | -0.933366 |
| TRINITY_DN30233_c0_g1_i2 | blue | -0.933387 |
| TRINITY_DN25624_c0_g1_i4 | blue | -0.933405 |
| TRINITY_DN32620_c0_g1_i14 | blue | -0.933484 |
| TRINITY_DN35760_c0_g1_i4 | blue | -0.933536 |
| TRINITY_DN36152_c1_g1_i3 | blue | -0.933569 |
| TRINITY_DN25349_c0_g1_i2 | blue | -0.933582 |
| TRINITY_DN38602_c0_g1_i14 | blue | -0.933595 |
| TRINITY_DN38836_c0_g1_i4 | blue | -0.933666 |
| TRINITY_DN34991_c0_g1_i1 | blue | -0.93376 |
| TRINITY_DN35074_c1_g3_i2 | blue | -0.933792 |
| TRINITY_DN27504_c0_g2_i1 | blue | -0.933837 |
| TRINITY_DN35328_c2_g3_i2 | blue | -0.933848 |
| TRINITY_DN17327_c0_g1_i1 | blue | -0.934001 |
| TRINITY_DN36448_c1_g1_i4 | blue | -0.934347 |
| TRINITY_DN38668_c0_g2_i1 | blue | -0.934893 |
| TRINITY_DN36158_c0_g1_i2 | blue | -0.935173 |
| TRINITY_DN35026_c0_g1_i13 | blue | -0.935234 |
| TRINITY_DN39264_c4_g2_i1 | blue | -0.93562 |
| TRINITY_DN38690_c0_g3_i3 | blue | -0.935681 |
| TRINITY_DN32336_c0_g1_i13 | blue | -0.935756 |
| TRINITY_DN26900_c0_g1_i4 | blue | -0.935977 |
| TRINITY_DN33837_c0_g1_i6 | blue | -0.937371 |
| TRINITY_DN38516_c0_g5_i30 | blue | -0.93758 |
| TRINITY_DN24916_c0_g1_i2 | blue | -0.937681 |
| TRINITY_DN34735_c0_g1_i11 | blue | -0.937997 |
| TRINITY_DN37069_c0_g3_i1 | blue | -0.938127 |
| TRINITY_DN25373_c0_g2_i4 | blue | -0.938209 |
| TRINITY_DN32757_c0_g1_i16 | blue | -0.938225 |
| TRINITY_DN34453_c0_g2_i6 | blue | -0.938479 |
| TRINITY_DN33226_c0_g1_i4 | blue | -0.93874 |
| TRINITY_DN29680_c0_g1_i2 | blue | -0.939183 |
| TRINITY_DN39163_c0_g1_i1 | blue | -0.939913 |
| TRINITY_DN35866_c2_g1_i2 | blue | -0.940462 |
| TRINITY_DN26796_c0_g1_i2 | blue | -0.940713 |
| TRINITY_DN35076_c0_g4_i1 | blue | -0.941192 |
| TRINITY_DN30065_c0_g1_i7 | blue | -0.941837 |
| TRINITY_DN28544_c0_g2_i1 | blue | -0.942766 |
| TRINITY_DN38798_c1_g2_i1 | blue | -0.942867 |
| TRINITY_DN37664_c0_g2_i5 | blue | -0.942949 |
| TRINITY_DN34742_c1_g1_i2 | blue | -0.943146 |
| TRINITY_DN36906_c0_g2_i1 | blue | -0.944139 |
| TRINITY_DN29955_c0_g1_i4 | blue | -0.94441 |
| TRINITY_DN28830_c0_g1_i1 | blue | -0.944532 |
| TRINITY_DN26840_c0_g1_i1 | blue | -0.945668 |
| TRINITY_DN29221_c0_g2_i5 | blue | -0.945924 |
| TRINITY_DN35736_c0_g2_i3 | blue | -0.946008 |
| TRINITY_DN29286_c0_g2_i2 | blue | -0.946363 |
| TRINITY_DN28017_c1_g1_i2 | blue | -0.946497 |
| TRINITY_DN29989_c0_g1_i7 | blue | -0.946836 |
| TRINITY_DN25228_c0_g1_i1 | blue | -0.947407 |
| TRINITY_DN34654_c0_g2_i2 | blue | -0.947437 |
| TRINITY_DN27534_c0_g1_i2 | blue | -0.948332 |
| TRINITY_DN36874_c1_g1_i5 | blue | -0.94884 |
| TRINITY_DN35016_c0_g1_i19 | blue | -0.948843 |
| TRINITY_DN30365_c0_g1_i3 | blue | -0.949331 |
| TRINITY_DN37639_c1_g2_i2 | blue | -0.949676 |
| TRINITY_DN36297_c0_g2_i1 | blue | -0.949956 |
| TRINITY_DN34425_c1_g4_i1 | blue | -0.9504 |
| TRINITY_DN26756_c0_g1_i1 | blue | -0.951202 |
| TRINITY_DN36040_c0_g1_i1 | blue | -0.951916 |
| TRINITY_DN38075_c0_g1_i2 | blue | -0.952767 |
| TRINITY_DN37738_c0_g2_i3 | blue | -0.953834 |
| TRINITY_DN28381_c0_g1_i1 | blue | -0.954711 |
| TRINITY_DN37677_c2_g1_i1 | blue | -0.955296 |
| TRINITY_DN37686_c0_g1_i1 | blue | -0.955386 |
| TRINITY_DN37923_c1_g4_i2 | blue | -0.956143 |
| TRINITY_DN34790_c3_g1_i4 | blue | -0.957865 |
| TRINITY_DN26941_c0_g1_i3 | blue | -0.958172 |
| TRINITY_DN27517_c0_g2_i5 | blue | -0.959522 |
| TRINITY_DN37639_c0_g2_i1 | blue | -0.961189 |
| TRINITY_DN37933_c0_g1_i1 | blue | -0.961969 |
| TRINITY_DN29750_c0_g1_i4 | blue | -0.962002 |
| TRINITY_DN36738_c0_g2_i1 | blue | -0.962741 |
| TRINITY_DN32093_c0_g1_i1 | blue | -0.96308 |
| TRINITY_DN34365_c0_g1_i6 | blue | -0.963562 |
| TRINITY_DN37750_c0_g4_i1 | blue | -0.964241 |
| TRINITY_DN32623_c0_g2_i1 | blue | -0.964555 |
| TRINITY_DN23390_c0_g1_i1 | blue | -0.964595 |
| TRINITY_DN33040_c0_g1_i8 | blue | -0.964682 |
| TRINITY_DN38040_c0_g4_i32 | blue | -0.969111 |
| TRINITY_DN56272_c0_g1_i1 | blue | -0.969727 |
| TRINITY_DN37987_c2_g2_i4 | blue | -0.970027 |
| TRINITY_DN35304_c0_g1_i18 | blue | -0.972312 |
| TRINITY_DN31802_c0_g1_i4 | blue | -0.976573 |
| TRINITY_DN38444_c4_g1_i6 | blue | -0.980608 |
| TRINITY_DN35306_c0_g2_i4 | blue | -0.986077 |
| TRINITY_DN35245_c1_g1_i3 | brown | 0.9961793 |
| TRINITY_DN38511_c1_g1_i2 | brown | 0.9946431 |
| TRINITY_DN39179_c0_g1_i5 | brown | 0.9929332 |
| TRINITY_DN36332_c0_g11_i1 | brown | 0.9920358 |
| TRINITY_DN36813_c0_g2_i1 | brown | 0.9915437 |
| TRINITY_DN32039_c0_g2_i2 | brown | 0.9915239 |
| TRINITY_DN38037_c0_g1_i5 | brown | 0.9900617 |
| TRINITY_DN26298_c0_g2_i1 | brown | 0.9899628 |
| TRINITY_DN38944_c0_g2_i2 | brown | 0.9898175 |
| TRINITY_DN37004_c0_g1_i3 | brown | 0.9889631 |
| TRINITY_DN37187_c1_g3_i2 | brown | 0.9870302 |
| TRINITY_DN37131_c0_g2_i1 | brown | 0.9865105 |
| TRINITY_DN38907_c0_g3_i1 | brown | 0.9861472 |
| TRINITY_DN25564_c0_g1_i1 | brown | 0.9859182 |
| TRINITY_DN30326_c0_g2_i1 | brown | 0.9856896 |
| TRINITY_DN34886_c0_g2_i1 | brown | 0.9849325 |
| TRINITY_DN33214_c1_g2_i1 | brown | 0.9843833 |
| TRINITY_DN34946_c0_g6_i1 | brown | 0.9829703 |
| TRINITY_DN35366_c0_g3_i1 | brown | 0.9824562 |
| TRINITY_DN37187_c1_g2_i1 | brown | 0.9824133 |
| TRINITY_DN38577_c0_g1_i3 | brown | 0.9822465 |
| TRINITY_DN21193_c0_g1_i1 | brown | 0.9821474 |
| TRINITY_DN38944_c0_g4_i1 | brown | 0.9815113 |
| TRINITY_DN37770_c1_g1_i1 | brown | 0.9814193 |
| TRINITY_DN36248_c0_g2_i1 | brown | 0.9813616 |
| TRINITY_DN36269_c0_g7_i1 | brown | 0.9807623 |
| TRINITY_DN17075_c0_g3_i1 | brown | 0.9802632 |
| TRINITY_DN38956_c0_g1_i1 | brown | 0.9797585 |
| TRINITY_DN37004_c0_g2_i2 | brown | 0.9795059 |
| TRINITY_DN16494_c0_g1_i1 | brown | 0.9791219 |
| TRINITY_DN38338_c1_g1_i1 | brown | 0.9790972 |
| TRINITY_DN31736_c0_g1_i5 | brown | 0.979089 |
| TRINITY_DN34683_c0_g3_i4 | brown | 0.9788884 |
| TRINITY_DN18595_c0_g2_i1 | brown | 0.9783482 |
| TRINITY_DN29840_c0_g1_i6 | brown | 0.9781346 |
| TRINITY_DN34383_c0_g1_i6 | brown | 0.9776352 |
| TRINITY_DN38498_c0_g1_i1 | brown | 0.9774678 |
| TRINITY_DN18973_c0_g1_i1 | brown | 0.9774539 |
| TRINITY_DN37133_c0_g2_i1 | brown | 0.9774535 |
| TRINITY_DN39007_c0_g3_i6 | brown | 0.9771908 |
| TRINITY_DN33478_c0_g2_i3 | brown | 0.9767354 |
| TRINITY_DN33283_c1_g1_i1 | brown | 0.976717 |
| TRINITY_DN26277_c0_g1_i2 | brown | 0.9762104 |
| TRINITY_DN37896_c0_g2_i1 | brown | 0.9761587 |
| TRINITY_DN24968_c0_g2_i1 | brown | 0.9759432 |
| TRINITY_DN27936_c0_g1_i3 | brown | 0.9759401 |
| TRINITY_DN36909_c0_g1_i1 | brown | 0.9757772 |
| TRINITY_DN38888_c2_g5_i1 | brown | 0.9757409 |
| TRINITY_DN39278_c3_g3_i1 | brown | 0.9757001 |
| TRINITY_DN39278_c3_g1_i8 | brown | 0.9756131 |
| TRINITY_DN34098_c0_g7_i1 | brown | 0.9755745 |
| TRINITY_DN39043_c5_g10_i1 | brown | 0.9753865 |
| TRINITY_DN34098_c0_g4_i2 | brown | 0.9750706 |
| TRINITY_DN33554_c0_g3_i1 | brown | 0.9747856 |
| TRINITY_DN34886_c0_g4_i1 | brown | 0.9747081 |
| TRINITY_DN37786_c0_g3_i2 | brown | 0.9746486 |
| TRINITY_DN16569_c0_g1_i1 | brown | 0.9745966 |
| TRINITY_DN28153_c0_g1_i1 | brown | 0.9741296 |
| TRINITY_DN38578_c0_g1_i1 | brown | 0.974075 |
| TRINITY_DN32550_c0_g2_i1 | brown | 0.973741 |
| TRINITY_DN22937_c0_g3_i1 | brown | 0.9731101 |
| TRINITY_DN33558_c0_g1_i11 | brown | 0.9728903 |
| TRINITY_DN38862_c1_g1_i3 | brown | 0.9727782 |
| TRINITY_DN26892_c0_g1_i1 | brown | 0.9724845 |
| TRINITY_DN23126_c0_g2_i1 | brown | 0.9722596 |
| TRINITY_DN37811_c1_g4_i2 | brown | 0.9721771 |
| TRINITY_DN37741_c0_g1_i2 | brown | 0.9718176 |
| TRINITY_DN33659_c0_g3_i1 | brown | 0.9708343 |
| TRINITY_DN37029_c0_g7_i2 | brown | 0.9708164 |
| TRINITY_DN37246_c1_g4_i1 | brown | 0.9707627 |
| TRINITY_DN31555_c0_g3_i1 | brown | 0.9702129 |
| TRINITY_DN38338_c1_g3_i1 | brown | 0.969833 |
| TRINITY_DN28077_c0_g3_i1 | brown | 0.9695037 |
| TRINITY_DN21995_c0_g2_i1 | brown | 0.9694696 |
| TRINITY_DN28074_c0_g1_i2 | brown | 0.969431 |
| TRINITY_DN37359_c0_g3_i1 | brown | 0.9691252 |
| TRINITY_DN39068_c0_g15_i1 | brown | 0.9691062 |
| TRINITY_DN19358_c0_g2_i1 | brown | 0.9688095 |
| TRINITY_DN38578_c0_g5_i1 | brown | 0.9686785 |
| TRINITY_DN27868_c0_g2_i2 | brown | 0.9683772 |
| TRINITY_DN16368_c0_g3_i1 | brown | 0.9678756 |
| TRINITY_DN39256_c0_g4_i1 | brown | 0.9678057 |
| TRINITY_DN36607_c0_g5_i1 | brown | 0.9677039 |
| TRINITY_DN38688_c1_g1_i2 | brown | 0.9676435 |
| TRINITY_DN39309_c1_g1_i1 | brown | 0.9674946 |
| TRINITY_DN36204_c0_g1_i3 | brown | 0.9674398 |
| TRINITY_DN37469_c1_g4_i1 | brown | 0.9670805 |
| TRINITY_DN38689_c1_g1_i1 | brown | 0.9670151 |
| TRINITY_DN37897_c0_g1_i8 | brown | 0.9670008 |
| TRINITY_DN39237_c5_g12_i2 | brown | 0.9669765 |
| TRINITY_DN44816_c0_g1_i1 | brown | 0.9664997 |
| TRINITY_DN39289_c2_g1_i1 | brown | 0.9663565 |
| TRINITY_DN37319_c0_g8_i1 | brown | 0.9663494 |
| TRINITY_DN36836_c1_g2_i1 | brown | 0.9659557 |
| TRINITY_DN35359_c0_g3_i1 | brown | 0.9657451 |
| TRINITY_DN37416_c0_g2_i8 | brown | 0.9656865 |
| TRINITY_DN24069_c0_g1_i1 | brown | 0.965468 |
| TRINITY_DN22288_c0_g2_i1 | brown | 0.9650666 |
| TRINITY_DN33748_c0_g1_i2 | brown | 0.9649975 |
| TRINITY_DN38863_c1_g4_i1 | brown | 0.9644947 |
| TRINITY_DN39340_c22_g12_i1 | brown | 0.9637027 |
| TRINITY_DN29985_c0_g4_i1 | brown | 0.9635015 |
| TRINITY_DN36522_c1_g1_i1 | brown | 0.9632617 |
| TRINITY_DN29835_c0_g3_i1 | brown | 0.9631436 |
| TRINITY_DN33081_c0_g8_i3 | brown | 0.9625243 |
| TRINITY_DN39090_c1_g2_i6 | brown | 0.9622949 |
| TRINITY_DN29812_c0_g2_i5 | brown | 0.9621066 |
| TRINITY_DN26022_c0_g3_i1 | brown | 0.9620019 |
| TRINITY_DN22937_c0_g1_i1 | brown | 0.9619281 |
| TRINITY_DN31190_c0_g1_i1 | brown | 0.9617913 |
| TRINITY_DN39006_c0_g1_i1 | brown | 0.9616628 |
| TRINITY_DN56891_c0_g1_i1 | brown | 0.9615606 |
| TRINITY_DN36051_c0_g4_i1 | brown | 0.9612868 |
| TRINITY_DN38474_c0_g3_i5 | brown | 0.9611455 |
| TRINITY_DN37205_c0_g5_i1 | brown | 0.9611438 |
| TRINITY_DN30722_c0_g14_i1 | brown | 0.9609198 |
| TRINITY_DN20555_c0_g1_i1 | brown | 0.9608572 |
| TRINITY_DN31758_c1_g15_i1 | brown | 0.9606278 |
| TRINITY_DN36751_c0_g1_i3 | brown | 0.9604525 |
| TRINITY_DN38907_c1_g6_i1 | brown | 0.9604202 |
| TRINITY_DN14199_c0_g1_i1 | brown | 0.9603423 |
| TRINITY_DN22684_c0_g1_i1 | brown | 0.9600647 |
| TRINITY_DN33031_c0_g3_i1 | brown | 0.9596915 |
| TRINITY_DN34156_c1_g4_i2 | brown | 0.9596514 |
| TRINITY_DN11252_c0_g1_i1 | brown | 0.9594993 |
| TRINITY_DN24366_c0_g2_i1 | brown | 0.9592858 |
| TRINITY_DN36672_c1_g1_i1 | brown | 0.9586949 |
| TRINITY_DN31991_c0_g1_i6 | brown | 0.9583844 |
| TRINITY_DN31957_c1_g5_i2 | brown | 0.9583561 |
| TRINITY_DN38561_c0_g3_i1 | brown | 0.9580245 |
| TRINITY_DN36415_c0_g4_i1 | brown | 0.9577025 |
| TRINITY_DN41770_c0_g1_i1 | brown | 0.9571403 |
| TRINITY_DN37594_c0_g1_i1 | brown | 0.9569271 |
| TRINITY_DN34038_c0_g3_i1 | brown | 0.9568755 |
| TRINITY_DN31462_c0_g4_i1 | brown | 0.9568281 |
| TRINITY_DN13487_c0_g2_i1 | brown | 0.9565226 |
| TRINITY_DN30963_c0_g2_i1 | brown | 0.9564839 |
| TRINITY_DN30288_c0_g1_i1 | brown | 0.9558602 |
| TRINITY_DN36961_c0_g1_i2 | brown | 0.9554991 |
| TRINITY_DN26402_c0_g1_i1 | brown | 0.9553503 |
| TRINITY_DN34052_c0_g2_i2 | brown | 0.9549006 |
| TRINITY_DN20135_c0_g1_i1 | brown | 0.9547695 |
| TRINITY_DN24539_c0_g2_i1 | brown | 0.954597 |
| TRINITY_DN22839_c0_g1_i1 | brown | 0.9544144 |
| TRINITY_DN39130_c0_g4_i1 | brown | 0.9543116 |
| TRINITY_DN38279_c0_g18_i1 | brown | 0.9542169 |
| TRINITY_DN53097_c0_g2_i1 | brown | 0.9537865 |
| TRINITY_DN29226_c0_g2_i1 | brown | 0.9537358 |
| TRINITY_DN38079_c0_g3_i1 | brown | 0.9536829 |
| TRINITY_DN24394_c0_g1_i1 | brown | 0.9535675 |
| TRINITY_DN21363_c0_g4_i1 | brown | 0.9535114 |
| TRINITY_DN37057_c0_g3_i1 | brown | 0.9534931 |
| TRINITY_DN34627_c0_g2_i1 | brown | 0.953376 |
| TRINITY_DN31901_c0_g1_i3 | brown | 0.9532529 |
| TRINITY_DN31061_c0_g2_i1 | brown | 0.9529953 |
| TRINITY_DN38323_c2_g4_i1 | brown | 0.9527814 |
| TRINITY_DN37318_c2_g6_i3 | brown | 0.9526212 |
| TRINITY_DN38390_c0_g1_i10 | brown | 0.9525398 |
| TRINITY_DN35148_c0_g1_i2 | brown | 0.9523255 |
| TRINITY_DN31667_c0_g6_i1 | brown | 0.9522594 |
| TRINITY_DN37573_c0_g1_i1 | brown | 0.9519429 |
| TRINITY_DN35264_c0_g1_i10 | brown | 0.9519318 |
| TRINITY_DN21438_c0_g1_i1 | brown | 0.9517192 |
| TRINITY_DN34164_c1_g2_i2 | brown | 0.9514631 |
| TRINITY_DN36149_c3_g4_i2 | brown | 0.9514342 |
| TRINITY_DN22803_c0_g1_i1 | brown | 0.9511733 |
| TRINITY_DN37164_c0_g3_i1 | brown | 0.9509107 |
| TRINITY_DN19375_c0_g1_i1 | brown | 0.950874 |
| TRINITY_DN38693_c3_g10_i1 | brown | 0.9508399 |
| TRINITY_DN33446_c3_g4_i7 | brown | 0.9503585 |
| TRINITY_DN37164_c0_g1_i5 | brown | 0.9502783 |
| TRINITY_DN38254_c1_g3_i1 | brown | 0.9502472 |
| TRINITY_DN25671_c0_g1_i1 | brown | 0.9502217 |
| TRINITY_DN38888_c2_g21_i1 | brown | 0.9501802 |
| TRINITY_DN36424_c0_g5_i1 | brown | 0.9501592 |
| TRINITY_DN39215_c0_g1_i1 | brown | 0.9498793 |
| TRINITY_DN33732_c1_g1_i1 | brown | 0.9491441 |
| TRINITY_DN28533_c0_g6_i1 | brown | 0.9485421 |
| TRINITY_DN37469_c1_g5_i1 | brown | 0.9483686 |
| TRINITY_DN19496_c0_g2_i1 | brown | 0.9482111 |
| TRINITY_DN37063_c0_g4_i1 | brown | 0.9480767 |
| TRINITY_DN39205_c1_g8_i1 | brown | 0.9479666 |
| TRINITY_DN29745_c0_g2_i1 | brown | 0.9479482 |
| TRINITY_DN38913_c1_g10_i3 | brown | 0.9478844 |
| TRINITY_DN39068_c0_g13_i1 | brown | 0.9477937 |
| TRINITY_DN33706_c0_g1_i2 | brown | 0.94766 |
| TRINITY_DN23961_c0_g2_i1 | brown | 0.9476535 |
| TRINITY_DN39200_c1_g3_i1 | brown | 0.9474038 |
| TRINITY_DN28649_c0_g2_i1 | brown | 0.9472172 |
| TRINITY_DN28533_c0_g4_i1 | brown | 0.9468179 |
| TRINITY_DN26143_c0_g1_i1 | brown | 0.9467312 |
| TRINITY_DN39084_c2_g4_i1 | brown | 0.9464603 |
| TRINITY_DN37416_c0_g7_i1 | brown | 0.9463458 |
| TRINITY_DN36391_c1_g1_i6 | brown | 0.9463074 |
| TRINITY_DN36402_c3_g2_i1 | brown | 0.9462396 |
| TRINITY_DN39058_c1_g5_i2 | brown | 0.946214 |
| TRINITY_DN35356_c0_g2_i1 | brown | 0.9462094 |
| TRINITY_DN31139_c1_g1_i1 | brown | 0.9461533 |
| TRINITY_DN33760_c3_g1_i1 | brown | 0.9457157 |
| TRINITY_DN35984_c1_g2_i1 | brown | 0.9455125 |
| TRINITY_DN38535_c0_g12_i2 | brown | 0.9452893 |
| TRINITY_DN39296_c4_g2_i1 | brown | 0.9452131 |
| TRINITY_DN38956_c0_g4_i1 | brown | 0.945142 |
| TRINITY_DN35963_c0_g7_i4 | brown | 0.9449216 |
| TRINITY_DN28811_c0_g1_i1 | brown | 0.9448398 |
| TRINITY_DN27524_c0_g1_i1 | brown | 0.9448299 |
| TRINITY_DN35640_c2_g2_i1 | brown | 0.9448024 |
| TRINITY_DN37386_c1_g3_i1 | brown | 0.944798 |
| TRINITY_DN35463_c1_g7_i1 | brown | 0.9446893 |
| TRINITY_DN32090_c1_g3_i3 | brown | 0.9446526 |
| TRINITY_DN18568_c0_g3_i1 | brown | 0.9441658 |
| TRINITY_DN37006_c13_g1_i1 | brown | 0.9438723 |
| TRINITY_DN31773_c1_g2_i1 | brown | 0.9437026 |
| TRINITY_DN37052_c0_g2_i1 | brown | 0.9436297 |
| TRINITY_DN22495_c0_g1_i1 | brown | 0.9435558 |
| TRINITY_DN39083_c0_g6_i2 | brown | 0.9434937 |
| TRINITY_DN36286_c0_g4_i1 | brown | 0.9433059 |
| TRINITY_DN25018_c0_g2_i1 | brown | 0.9431123 |
| TRINITY_DN51344_c0_g1_i1 | brown | 0.9423646 |
| TRINITY_DN38511_c1_g4_i1 | brown | 0.9423347 |
| TRINITY_DN26755_c0_g2_i1 | brown | 0.9422891 |
| TRINITY_DN17681_c0_g2_i1 | brown | 0.9418384 |
| TRINITY_DN36577_c1_g2_i2 | brown | 0.9417014 |
| TRINITY_DN31134_c0_g1_i1 | brown | 0.9412258 |
| TRINITY_DN38263_c0_g1_i4 | brown | 0.9409414 |
| TRINITY_DN39205_c1_g9_i1 | brown | 0.9408677 |
| TRINITY_DN32560_c1_g1_i1 | brown | 0.9404366 |
| TRINITY_DN30876_c1_g8_i2 | brown | 0.9400805 |
| TRINITY_DN38984_c3_g1_i1 | brown | 0.9399705 |
| TRINITY_DN32665_c0_g1_i3 | brown | 0.9399693 |
| TRINITY_DN23415_c0_g1_i8 | brown | 0.9399609 |
| TRINITY_DN30346_c0_g1_i16 | brown | 0.939824 |
| TRINITY_DN35695_c0_g2_i5 | brown | 0.9397401 |
| TRINITY_DN36178_c0_g2_i6 | brown | 0.9396917 |
| TRINITY_DN24625_c0_g3_i1 | brown | 0.9396455 |
| TRINITY_DN20058_c0_g1_i1 | brown | 0.9395483 |
| TRINITY_DN27091_c1_g2_i1 | brown | 0.9393901 |
| TRINITY_DN27341_c0_g1_i1 | brown | 0.9391754 |
| TRINITY_DN28926_c0_g1_i2 | brown | 0.9390715 |
| TRINITY_DN33163_c0_g1_i1 | brown | 0.9390529 |
| TRINITY_DN16874_c0_g4_i1 | brown | 0.9388725 |
| TRINITY_DN20088_c0_g3_i1 | brown | 0.9388411 |
| TRINITY_DN27918_c0_g1_i7 | brown | 0.9388016 |
| TRINITY_DN17314_c0_g1_i1 | brown | 0.9385174 |
| TRINITY_DN38693_c3_g9_i1 | brown | 0.9380425 |
| TRINITY_DN33214_c2_g1_i4 | brown | 0.937972 |
| TRINITY_DN35977_c0_g20_i1 | brown | 0.9374822 |
| TRINITY_DN22598_c0_g1_i1 | brown | 0.9370357 |
| TRINITY_DN29807_c0_g1_i1 | brown | 0.9368999 |
| TRINITY_DN28649_c0_g3_i1 | brown | 0.9365333 |
| TRINITY_DN36060_c0_g1_i1 | brown | 0.9364472 |
| TRINITY_DN52613_c0_g1_i1 | brown | 0.9363822 |
| TRINITY_DN35440_c0_g2_i1 | brown | 0.935997 |
| TRINITY_DN27260_c0_g3_i1 | brown | 0.9359142 |
| TRINITY_DN30674_c0_g1_i6 | brown | 0.9357165 |
| TRINITY_DN27927_c0_g1_i1 | brown | 0.9357091 |
| TRINITY_DN32319_c0_g2_i1 | brown | 0.9356568 |
| TRINITY_DN38511_c1_g2_i1 | brown | 0.935338 |
| TRINITY_DN35698_c0_g1_i1 | brown | 0.9352654 |
| TRINITY_DN30250_c0_g1_i2 | brown | 0.9352632 |
| TRINITY_DN22371_c0_g1_i3 | brown | 0.9351607 |
| TRINITY_DN24720_c0_g2_i1 | brown | 0.9349367 |
| TRINITY_DN38025_c3_g3_i1 | brown | 0.9344035 |
| TRINITY_DN39256_c0_g3_i5 | brown | 0.9338741 |
| TRINITY_DN31077_c1_g1_i11 | brown | 0.9334524 |
| TRINITY_DN24470_c0_g4_i1 | brown | 0.9334509 |
| TRINITY_DN36026_c0_g7_i1 | brown | 0.9333351 |
| TRINITY_DN26140_c0_g2_i1 | brown | 0.9329863 |
| TRINITY_DN37695_c1_g2_i7 | brown | 0.9327131 |
| TRINITY_DN37751_c2_g3_i1 | brown | 0.9322381 |
| TRINITY_DN27179_c0_g1_i2 | brown | 0.9322073 |
| TRINITY_DN39172_c2_g2_i1 | brown | 0.9320525 |
| TRINITY_DN36996_c0_g2_i8 | brown | 0.9318988 |
| TRINITY_DN38979_c1_g3_i1 | brown | 0.9316904 |
| TRINITY_DN39136_c1_g2_i3 | brown | 0.9313783 |
| TRINITY_DN39187_c0_g2_i1 | brown | 0.9312915 |
| TRINITY_DN26096_c0_g2_i1 | brown | 0.9309498 |
| TRINITY_DN38146_c1_g6_i8 | brown | 0.9305202 |
| TRINITY_DN37287_c1_g1_i17 | brown | 0.9297299 |
| TRINITY_DN25676_c0_g1_i1 | brown | 0.9293997 |
| TRINITY_DN39289_c3_g4_i1 | brown | 0.9292841 |
| TRINITY_DN26331_c0_g1_i4 | brown | 0.9291781 |
| TRINITY_DN39227_c1_g4_i1 | brown | 0.9291031 |
| TRINITY_DN21011_c0_g1_i1 | brown | 0.9290793 |
| TRINITY_DN22225_c0_g1_i1 | brown | 0.9290214 |
| TRINITY_DN25589_c0_g2_i1 | brown | 0.9289206 |
| TRINITY_DN31298_c1_g1_i1 | brown | 0.9287064 |
| TRINITY_DN22941_c0_g1_i3 | brown | 0.9284222 |
| TRINITY_DN11128_c0_g1_i1 | brown | 0.9281695 |
| TRINITY_DN34264_c1_g2_i4 | brown | 0.9278903 |
| TRINITY_DN37386_c1_g5_i2 | brown | 0.9275884 |
| TRINITY_DN20310_c0_g2_i1 | brown | 0.927309 |
| TRINITY_DN17361_c0_g2_i1 | brown | 0.9270083 |
| TRINITY_DN34946_c0_g2_i1 | brown | 0.9269901 |
| TRINITY_DN21367_c0_g2_i1 | brown | 0.9269187 |
| TRINITY_DN34103_c0_g1_i2 | brown | 0.9267439 |
| TRINITY_DN38500_c0_g1_i11 | brown | 0.9266404 |
| TRINITY_DN29142_c0_g1_i2 | brown | 0.9260004 |
| TRINITY_DN36269_c0_g6_i1 | brown | 0.925894 |
| TRINITY_DN35871_c0_g4_i6 | brown | 0.9258447 |
| TRINITY_DN35457_c3_g12_i2 | brown | 0.9256392 |
| TRINITY_DN38047_c0_g1_i1 | brown | 0.9255322 |
| TRINITY_DN36581_c3_g8_i1 | brown | 0.9254335 |
| TRINITY_DN37701_c0_g1_i8 | brown | 0.9252974 |
| TRINITY_DN36185_c2_g9_i1 | brown | 0.9252459 |
| TRINITY_DN33109_c0_g4_i1 | brown | 0.9251823 |
| TRINITY_DN45664_c0_g1_i1 | brown | 0.9249859 |
| TRINITY_DN38913_c1_g1_i1 | brown | 0.9246945 |
| TRINITY_DN34812_c1_g2_i1 | brown | 0.9246659 |
| TRINITY_DN30011_c3_g12_i1 | brown | 0.924359 |
| TRINITY_DN24813_c0_g2_i1 | brown | 0.9240147 |
| TRINITY_DN33456_c0_g3_i1 | brown | 0.9238306 |
| TRINITY_DN33102_c1_g2_i15 | brown | 0.923614 |
| TRINITY_DN23342_c0_g2_i1 | brown | 0.9236073 |
| TRINITY_DN22258_c0_g2_i1 | brown | 0.9235569 |
| TRINITY_DN35457_c2_g2_i1 | brown | 0.9235077 |
| TRINITY_DN34878_c2_g3_i11 | brown | 0.9234327 |
| TRINITY_DN24796_c0_g1_i3 | brown | 0.9232348 |
| TRINITY_DN57573_c0_g1_i1 | brown | 0.9228788 |
| TRINITY_DN24706_c0_g1_i1 | brown | 0.9228471 |
| TRINITY_DN22051_c0_g1_i1 | brown | 0.922789 |
| TRINITY_DN37104_c0_g1_i2 | brown | 0.9225222 |
| TRINITY_DN39137_c1_g2_i1 | brown | 0.9223405 |
| TRINITY_DN36797_c4_g1_i6 | brown | 0.9221663 |
| TRINITY_DN29080_c0_g2_i1 | brown | 0.9220968 |
| TRINITY_DN26948_c0_g1_i1 | brown | 0.9219828 |
| TRINITY_DN25854_c0_g3_i1 | brown | 0.9213473 |
| TRINITY_DN288_c0_g1_i1 | brown | 0.9211633 |
| TRINITY_DN34904_c0_g1_i2 | brown | 0.9208471 |
| TRINITY_DN34941_c0_g3_i1 | brown | 0.9207599 |
| TRINITY_DN37868_c2_g2_i1 | brown | 0.9207199 |
| TRINITY_DN36314_c0_g3_i1 | brown | 0.9204849 |
| TRINITY_DN25741_c0_g1_i1 | brown | 0.9204259 |
| TRINITY_DN36496_c1_g3_i1 | brown | 0.9201882 |
| TRINITY_DN27159_c0_g2_i1 | brown | 0.9201485 |
| TRINITY_DN33278_c1_g8_i1 | brown | 0.9201195 |
| TRINITY_DN36028_c1_g4_i1 | brown | 0.920081 |
| TRINITY_DN17474_c0_g2_i1 | brown | 0.919919 |
| TRINITY_DN4249_c0_g1_i1 | brown | 0.9199182 |
| TRINITY_DN25276_c0_g1_i2 | brown | 0.9197198 |
| TRINITY_DN34025_c0_g1_i1 | brown | 0.9194098 |
| TRINITY_DN21248_c0_g2_i1 | brown | 0.9193586 |
| TRINITY_DN31815_c0_g1_i4 | brown | 0.9191946 |
| TRINITY_DN36574_c1_g2_i1 | brown | 0.9186957 |
| TRINITY_DN23410_c0_g1_i1 | brown | 0.9186398 |
| TRINITY_DN35925_c2_g2_i3 | brown | 0.91859 |
| TRINITY_DN37974_c0_g4_i1 | brown | 0.918354 |
| TRINITY_DN32025_c0_g1_i1 | brown | 0.9182659 |
| TRINITY_DN38888_c2_g7_i2 | brown | 0.918222 |
| TRINITY_DN32934_c1_g4_i8 | brown | 0.9180941 |
| TRINITY_DN31607_c0_g1_i1 | brown | 0.9179336 |
| TRINITY_DN34873_c0_g2_i4 | brown | 0.9176304 |
| TRINITY_DN38918_c3_g1_i4 | brown | 0.9175842 |
| TRINITY_DN37859_c0_g2_i2 | brown | 0.9172469 |
| TRINITY_DN38573_c0_g2_i1 | brown | 0.9169693 |
| TRINITY_DN27402_c0_g1_i1 | brown | 0.9169135 |
| TRINITY_DN23897_c0_g1_i1 | brown | 0.9167849 |
| TRINITY_DN39289_c3_g1_i5 | brown | 0.9167801 |
| TRINITY_DN36094_c1_g2_i2 | brown | 0.9159125 |
| TRINITY_DN39208_c0_g8_i1 | brown | 0.9157523 |
| TRINITY_DN37477_c0_g1_i1 | brown | 0.9156278 |
| TRINITY_DN16960_c0_g2_i2 | brown | 0.9152344 |
| TRINITY_DN35899_c0_g9_i1 | brown | 0.9151742 |
| TRINITY_DN29509_c0_g2_i1 | brown | 0.9150409 |
| TRINITY_DN68713_c0_g1_i1 | brown | 0.9149987 |
| TRINITY_DN29995_c0_g3_i2 | brown | 0.9148448 |
| TRINITY_DN23761_c0_g2_i1 | brown | 0.9146517 |
| TRINITY_DN38701_c1_g8_i1 | brown | 0.9146505 |
| TRINITY_DN31349_c0_g2_i2 | brown | 0.9145186 |
| TRINITY_DN37734_c1_g2_i1 | brown | 0.9144863 |
| TRINITY_DN16222_c0_g2_i1 | brown | 0.9144191 |
| TRINITY_DN37977_c0_g2_i2 | brown | 0.9143684 |
| TRINITY_DN33464_c0_g3_i1 | brown | 0.9140379 |
| TRINITY_DN37386_c1_g7_i1 | brown | 0.913918 |
| TRINITY_DN36463_c0_g4_i4 | brown | 0.9138331 |
| TRINITY_DN31991_c0_g2_i1 | brown | 0.9137781 |
| TRINITY_DN37882_c0_g5_i1 | brown | 0.9136566 |
| TRINITY_DN33267_c0_g1_i1 | brown | 0.9136156 |
| TRINITY_DN38752_c0_g6_i4 | brown | 0.9134463 |
| TRINITY_DN38195_c0_g1_i1 | brown | 0.9133216 |
| TRINITY_DN31924_c0_g3_i1 | brown | 0.9131747 |
| TRINITY_DN30281_c0_g1_i1 | brown | 0.9131082 |
| TRINITY_DN29414_c0_g3_i1 | brown | 0.9129251 |
| TRINITY_DN38098_c0_g1_i1 | brown | 0.9129088 |
| TRINITY_DN24951_c0_g1_i1 | brown | 0.9125309 |
| TRINITY_DN38379_c1_g6_i1 | brown | 0.9123395 |
| TRINITY_DN27697_c0_g4_i1 | brown | 0.9117502 |
| TRINITY_DN20855_c0_g1_i1 | brown | 0.9115809 |
| TRINITY_DN21789_c0_g1_i1 | brown | 0.9115661 |
| TRINITY_DN35400_c0_g1_i1 | brown | 0.9115038 |
| TRINITY_DN31115_c0_g1_i1 | brown | 0.9113807 |
| TRINITY_DN31094_c0_g2_i1 | brown | 0.9113488 |
| TRINITY_DN33121_c2_g1_i3 | brown | 0.9112588 |
| TRINITY_DN25342_c0_g4_i2 | brown | 0.9112299 |
| TRINITY_DN24322_c0_g1_i1 | brown | 0.910728 |
| TRINITY_DN21681_c0_g2_i1 | brown | 0.9106185 |
| TRINITY_DN22363_c0_g1_i1 | brown | 0.9103593 |
| TRINITY_DN20220_c0_g2_i1 | brown | 0.9098004 |
| TRINITY_DN25078_c0_g2_i1 | brown | 0.9097906 |
| TRINITY_DN22777_c0_g2_i1 | brown | 0.9094836 |
| TRINITY_DN39052_c1_g1_i2 | brown | 0.9093981 |
| TRINITY_DN35776_c3_g3_i2 | brown | 0.9092987 |
| TRINITY_DN20244_c0_g1_i1 | brown | 0.9089718 |
| TRINITY_DN39068_c0_g20_i1 | brown | 0.9086831 |
| TRINITY_DN39322_c2_g3_i1 | brown | 0.9086488 |
| TRINITY_DN33054_c0_g1_i1 | brown | 0.9085801 |
| TRINITY_DN37149_c0_g1_i4 | brown | 0.9084566 |
| TRINITY_DN37663_c1_g6_i2 | brown | 0.9078113 |
| TRINITY_DN35147_c0_g5_i1 | brown | 0.9077621 |
| TRINITY_DN28037_c0_g2_i1 | brown | 0.9076502 |
| TRINITY_DN38452_c1_g3_i4 | brown | 0.9072579 |
| TRINITY_DN39208_c0_g5_i7 | brown | 0.907224 |
| TRINITY_DN32289_c0_g1_i14 | brown | 0.9072028 |
| TRINITY_DN33220_c0_g1_i4 | brown | 0.9071848 |
| TRINITY_DN27086_c0_g1_i1 | brown | 0.9071657 |
| TRINITY_DN32544_c0_g8_i1 | brown | 0.907089 |
| TRINITY_DN31160_c0_g2_i1 | brown | 0.9068732 |
| TRINITY_DN35682_c0_g1_i6 | brown | 0.9067124 |
| TRINITY_DN28077_c0_g1_i1 | brown | 0.9067054 |
| TRINITY_DN39057_c0_g13_i1 | brown | 0.906581 |
| TRINITY_DN24978_c0_g1_i1 | brown | 0.9064054 |
| TRINITY_DN30226_c0_g1_i1 | brown | 0.9063896 |
| TRINITY_DN37029_c0_g5_i2 | brown | 0.9061391 |
| TRINITY_DN35457_c3_g13_i1 | brown | 0.9055962 |
| TRINITY_DN37173_c1_g1_i1 | brown | 0.9055267 |
| TRINITY_DN45248_c0_g1_i1 | brown | 0.9054357 |
| TRINITY_DN27710_c1_g1_i1 | brown | 0.9054156 |
| TRINITY_DN25732_c0_g1_i1 | brown | 0.9052657 |
| TRINITY_DN20407_c0_g1_i1 | brown | 0.9051467 |
| TRINITY_DN36253_c0_g2_i1 | brown | 0.9051045 |
| TRINITY_DN32126_c0_g1_i1 | brown | 0.9049542 |
| TRINITY_DN18951_c0_g3_i1 | brown | 0.9047379 |
| TRINITY_DN32544_c0_g3_i1 | brown | 0.9045962 |
| TRINITY_DN24838_c0_g2_i1 | brown | 0.9044586 |
| TRINITY_DN28087_c1_g1_i1 | brown | 0.9044475 |
| TRINITY_DN36349_c1_g1_i2 | brown | 0.9041654 |
| TRINITY_DN32752_c0_g3_i3 | brown | 0.9040789 |
| TRINITY_DN35198_c0_g12_i1 | brown | 0.9039597 |
| TRINITY_DN36314_c0_g1_i3 | brown | 0.9038399 |
| TRINITY_DN38551_c1_g7_i2 | brown | 0.9036095 |
| TRINITY_DN37100_c1_g1_i1 | brown | 0.9035108 |
| TRINITY_DN26595_c0_g1_i2 | brown | 0.9033787 |
| TRINITY_DN38343_c1_g5_i1 | brown | 0.9033735 |
| TRINITY_DN38310_c0_g2_i1 | brown | 0.9031402 |
| TRINITY_DN39294_c6_g3_i1 | brown | 0.902992 |
| TRINITY_DN38936_c0_g5_i1 | brown | 0.9029805 |
| TRINITY_DN39317_c6_g2_i1 | brown | 0.9027569 |
| TRINITY_DN38786_c0_g2_i5 | brown | 0.902504 |
| TRINITY_DN38678_c1_g1_i2 | brown | 0.9020819 |
| TRINITY_DN33056_c0_g1_i1 | brown | 0.9020564 |
| TRINITY_DN35897_c2_g3_i1 | brown | 0.9018918 |
| TRINITY_DN33629_c0_g1_i13 | brown | 0.9018033 |
| TRINITY_DN68854_c0_g1_i1 | brown | 0.9015412 |
| TRINITY_DN31688_c1_g2_i2 | brown | 0.9014959 |
| TRINITY_DN39172_c2_g3_i1 | brown | 0.9014163 |
| TRINITY_DN30689_c0_g1_i3 | brown | 0.9013475 |
| TRINITY_DN21120_c0_g1_i1 | brown | 0.9012067 |
| TRINITY_DN39200_c1_g8_i3 | brown | 0.9010067 |
| TRINITY_DN25370_c0_g3_i3 | brown | 0.9009604 |
| TRINITY_DN57261_c0_g1_i1 | brown | 0.9009578 |
| TRINITY_DN33578_c0_g1_i4 | brown | 0.9004342 |
| TRINITY_DN39321_c2_g5_i1 | brown | 0.900037 |
| TRINITY_DN22438_c0_g1_i1 | brown | 0.9000261 |
| TRINITY_DN29835_c0_g4_i1 | brown | 0.8998944 |
| TRINITY_DN30738_c2_g5_i2 | brown | 0.8998741 |
| TRINITY_DN17116_c0_g3_i1 | brown | 0.8998725 |
| TRINITY_DN38863_c1_g9_i2 | brown | 0.8997663 |
| TRINITY_DN18655_c0_g1_i1 | brown | 0.8996704 |
| TRINITY_DN32544_c0_g4_i1 | brown | 0.8988629 |
| TRINITY_DN28215_c1_g2_i1 | brown | 0.8988251 |
| TRINITY_DN32298_c0_g1_i2 | brown | 0.8987898 |
| TRINITY_DN31847_c0_g4_i1 | brown | 0.8983481 |
| TRINITY_DN21758_c0_g1_i1 | brown | 0.8982292 |
| TRINITY_DN31133_c0_g2_i1 | brown | 0.8981953 |
| TRINITY_DN26780_c0_g1_i1 | brown | 0.8980478 |
| TRINITY_DN36768_c0_g2_i15 | brown | 0.8980403 |
| TRINITY_DN37540_c0_g6_i1 | brown | 0.8978598 |
| TRINITY_DN35142_c1_g1_i2 | brown | 0.897763 |
| TRINITY_DN35987_c0_g2_i2 | brown | 0.8977136 |
| TRINITY_DN35521_c0_g1_i13 | brown | 0.8975814 |
| TRINITY_DN39045_c1_g1_i1 | brown | 0.8975473 |
| TRINITY_DN35526_c2_g9_i1 | brown | 0.8974317 |
| TRINITY_DN39200_c1_g7_i1 | brown | 0.8972482 |
| TRINITY_DN33211_c3_g3_i6 | brown | 0.8970735 |
| TRINITY_DN32077_c0_g1_i1 | brown | 0.8970015 |
| TRINITY_DN39205_c1_g11_i1 | brown | 0.8968089 |
| TRINITY_DN58688_c0_g1_i1 | brown | 0.8964693 |
| TRINITY_DN37786_c0_g2_i4 | brown | 0.8963959 |
| TRINITY_DN32200_c1_g4_i1 | brown | 0.8963262 |
| TRINITY_DN33922_c1_g6_i1 | brown | 0.895996 |
| TRINITY_DN28614_c0_g1_i2 | brown | 0.8958375 |
| TRINITY_DN35640_c1_g2_i1 | brown | 0.8958339 |
| TRINITY_DN12934_c0_g1_i1 | brown | 0.8958176 |
| TRINITY_DN25458_c0_g1_i4 | brown | 0.8955001 |
| TRINITY_DN37029_c0_g4_i1 | brown | 0.8954359 |
| TRINITY_DN35400_c0_g3_i1 | brown | 0.895435 |
| TRINITY_DN16713_c0_g1_i1 | brown | 0.8953631 |
| TRINITY_DN30891_c0_g1_i1 | brown | 0.8953512 |
| TRINITY_DN27036_c0_g1_i1 | brown | 0.8951351 |
| TRINITY_DN33862_c0_g1_i1 | brown | 0.8951173 |
| TRINITY_DN19911_c0_g2_i1 | brown | 0.8950326 |
| TRINITY_DN34451_c0_g2_i1 | brown | 0.8948891 |
| TRINITY_DN40044_c0_g1_i1 | brown | 0.8947123 |
| TRINITY_DN35359_c0_g5_i7 | brown | 0.8946968 |
| TRINITY_DN39057_c0_g12_i2 | brown | 0.8944346 |
| TRINITY_DN38985_c1_g1_i1 | brown | 0.8943131 |
| TRINITY_DN31625_c0_g2_i1 | brown | 0.8942014 |
| TRINITY_DN16580_c0_g1_i1 | brown | 0.8941358 |
| TRINITY_DN14607_c0_g1_i1 | brown | 0.8940725 |
| TRINITY_DN38582_c0_g3_i5 | brown | 0.8939839 |
| TRINITY_DN38427_c0_g4_i1 | brown | 0.8939295 |
| TRINITY_DN39125_c0_g13_i1 | brown | 0.8936399 |
| TRINITY_DN18520_c0_g2_i1 | brown | 0.8932936 |
| TRINITY_DN28087_c0_g1_i1 | brown | 0.8929952 |
| TRINITY_DN22663_c0_g2_i1 | brown | 0.8929762 |
| TRINITY_DN19038_c0_g1_i1 | brown | 0.8928359 |
| TRINITY_DN36577_c1_g3_i4 | brown | 0.8928341 |
| TRINITY_DN21104_c0_g2_i1 | brown | 0.8926788 |
| TRINITY_DN39198_c4_g7_i6 | brown | 0.892253 |
| TRINITY_DN33447_c2_g3_i1 | brown | 0.8921559 |
| TRINITY_DN37903_c0_g1_i7 | brown | 0.8919463 |
| TRINITY_DN29984_c1_g3_i1 | brown | 0.8918205 |
| TRINITY_DN24206_c0_g1_i6 | brown | 0.8914403 |
| TRINITY_DN17691_c0_g1_i1 | brown | 0.8913519 |
| TRINITY_DN39022_c2_g4_i1 | brown | 0.891095 |
| TRINITY_DN24306_c0_g1_i1 | brown | 0.8910108 |
| TRINITY_DN20460_c0_g1_i1 | brown | 0.8903647 |
| TRINITY_DN32674_c1_g1_i3 | brown | 0.8902834 |
| TRINITY_DN29892_c0_g1_i2 | brown | 0.8902788 |
| TRINITY_DN33007_c3_g3_i1 | brown | 0.8901758 |
| TRINITY_DN33301_c0_g3_i6 | brown | 0.8898473 |
| TRINITY_DN38689_c1_g5_i1 | brown | 0.889723 |
| TRINITY_DN22261_c0_g1_i1 | brown | 0.8895834 |
| TRINITY_DN35135_c0_g5_i2 | brown | 0.8890309 |
| TRINITY_DN37672_c2_g1_i2 | brown | 0.8886298 |
| TRINITY_DN33255_c0_g1_i8 | brown | 0.8883608 |
| TRINITY_DN25794_c0_g1_i1 | brown | 0.8882246 |
| TRINITY_DN25308_c0_g1_i4 | brown | 0.8882092 |
| TRINITY_DN38412_c0_g1_i2 | brown | 0.8882044 |
| TRINITY_DN31833_c1_g3_i1 | brown | 0.8881516 |
| TRINITY_DN37614_c1_g8_i1 | brown | 0.8878575 |
| TRINITY_DN28486_c0_g1_i2 | brown | 0.8878329 |
| TRINITY_DN32845_c0_g2_i1 | brown | 0.8877266 |
| TRINITY_DN37169_c0_g1_i7 | brown | 0.8876016 |
| TRINITY_DN34632_c0_g5_i1 | brown | 0.8872952 |
| TRINITY_DN27651_c0_g2_i2 | brown | 0.8871998 |
| TRINITY_DN38323_c2_g9_i1 | brown | 0.8868883 |
| TRINITY_DN4455_c0_g1_i1 | brown | 0.8867118 |
| TRINITY_DN20774_c0_g3_i1 | brown | 0.8866463 |
| TRINITY_DN1311_c0_g1_i1 | brown | 0.8864257 |
| TRINITY_DN31147_c0_g3_i1 | brown | 0.886345 |
| TRINITY_DN21143_c0_g2_i2 | brown | 0.8863398 |
| TRINITY_DN39326_c1_g2_i1 | brown | 0.886334 |
| TRINITY_DN39339_c8_g6_i1 | brown | 0.8862768 |
| TRINITY_DN34503_c1_g1_i2 | brown | 0.8862522 |
| TRINITY_DN33406_c0_g3_i2 | brown | 0.8861472 |
| TRINITY_DN31321_c0_g2_i1 | brown | 0.8861244 |
| TRINITY_DN45417_c0_g1_i1 | brown | 0.8858477 |
| TRINITY_DN28988_c0_g1_i1 | brown | 0.8857887 |
| TRINITY_DN27728_c0_g1_i2 | brown | 0.885747 |
| TRINITY_DN31043_c0_g1_i2 | brown | 0.8857027 |
| TRINITY_DN37246_c1_g3_i2 | brown | 0.8856574 |
| TRINITY_DN32625_c0_g1_i3 | brown | 0.8856459 |
| TRINITY_DN31667_c0_g2_i1 | brown | 0.8853014 |
| TRINITY_DN31728_c2_g4_i1 | brown | 0.8852887 |
| TRINITY_DN31687_c1_g1_i1 | brown | 0.8850886 |
| TRINITY_DN39025_c0_g2_i12 | brown | 0.8850671 |
| TRINITY_DN34149_c0_g5_i1 | brown | 0.8850498 |
| TRINITY_DN17412_c0_g2_i1 | brown | 0.8850343 |
| TRINITY_DN33437_c0_g2_i1 | brown | 0.8846492 |
| TRINITY_DN30766_c0_g2_i1 | brown | 0.8844019 |
| TRINITY_DN15408_c0_g1_i1 | brown | 0.8836842 |
| TRINITY_DN24867_c0_g1_i1 | brown | 0.883563 |
| TRINITY_DN28247_c0_g2_i10 | brown | 0.8832257 |
| TRINITY_DN18760_c0_g3_i1 | brown | 0.883132 |
| TRINITY_DN24500_c1_g2_i1 | brown | 0.8831174 |
| TRINITY_DN30212_c0_g2_i1 | brown | 0.8830955 |
| TRINITY_DN36402_c3_g3_i3 | brown | 0.8830335 |
| TRINITY_DN3959_c0_g2_i1 | brown | 0.8829953 |
| TRINITY_DN36241_c2_g2_i1 | brown | 0.8829497 |
| TRINITY_DN38536_c0_g2_i1 | brown | 0.8827361 |
| TRINITY_DN35624_c0_g2_i6 | brown | 0.8826717 |
| TRINITY_DN38578_c0_g3_i1 | brown | 0.882063 |
| TRINITY_DN31874_c1_g1_i1 | brown | 0.8819383 |
| TRINITY_DN39043_c5_g7_i2 | brown | 0.8817511 |
| TRINITY_DN29539_c0_g1_i1 | brown | 0.8816479 |
| TRINITY_DN27669_c0_g2_i2 | brown | 0.8815711 |
| TRINITY_DN31758_c1_g4_i2 | brown | 0.8815169 |
| TRINITY_DN32258_c0_g1_i3 | brown | 0.881383 |
| TRINITY_DN25542_c0_g1_i1 | brown | 0.8811865 |
| TRINITY_DN20384_c0_g1_i1 | brown | 0.8810768 |
| TRINITY_DN31111_c1_g2_i4 | brown | 0.8808996 |
| TRINITY_DN34890_c1_g1_i2 | brown | 0.8808842 |
| TRINITY_DN36862_c4_g1_i1 | brown | 0.8808579 |
| TRINITY_DN35356_c0_g4_i1 | brown | 0.8807882 |
| TRINITY_DN22390_c0_g3_i1 | brown | 0.8805015 |
| TRINITY_DN17928_c0_g3_i1 | brown | 0.8801942 |
| TRINITY_DN36038_c1_g2_i1 | brown | 0.8801756 |
| TRINITY_DN39060_c0_g11_i1 | brown | 0.8800831 |
| TRINITY_DN38805_c0_g1_i10 | brown | 0.8798906 |
| TRINITY_DN17921_c0_g1_i1 | brown | 0.8796396 |
| TRINITY_DN37668_c0_g1_i13 | brown | 0.8794831 |
| TRINITY_DN28656_c0_g1_i1 | brown | 0.8794638 |
| TRINITY_DN35572_c0_g7_i3 | brown | 0.8794629 |
| TRINITY_DN34032_c0_g1_i7 | brown | 0.8790707 |
| TRINITY_DN25108_c0_g1_i1 | brown | 0.8785538 |
| TRINITY_DN34156_c1_g6_i1 | brown | 0.8785099 |
| TRINITY_DN28187_c0_g1_i3 | brown | 0.8784437 |
| TRINITY_DN29564_c0_g1_i1 | brown | 0.8781314 |
| TRINITY_DN29482_c0_g3_i1 | brown | 0.8776882 |
| TRINITY_DN24389_c0_g1_i1 | brown | 0.8775983 |
| TRINITY_DN24341_c0_g1_i1 | brown | 0.8772394 |
| TRINITY_DN31111_c2_g1_i2 | brown | 0.8771933 |
| TRINITY_DN35468_c0_g14_i2 | brown | 0.8768923 |
| TRINITY_DN27315_c0_g1_i1 | brown | 0.8767721 |
| TRINITY_DN37672_c1_g1_i1 | brown | 0.8766011 |
| TRINITY_DN37356_c1_g4_i1 | brown | 0.8763522 |
| TRINITY_DN35481_c1_g3_i3 | brown | 0.8762421 |
| TRINITY_DN35155_c0_g2_i4 | brown | 0.8760987 |
| TRINITY_DN31244_c0_g1_i1 | brown | 0.875884 |
| TRINITY_DN59502_c0_g1_i1 | brown | 0.8753685 |
| TRINITY_DN27785_c0_g2_i1 | brown | 0.8752861 |
| TRINITY_DN18521_c0_g1_i1 | brown | 0.8746513 |
| TRINITY_DN31728_c2_g3_i1 | brown | 0.8745862 |
| TRINITY_DN35252_c2_g2_i1 | brown | 0.8745773 |
| TRINITY_DN38251_c2_g7_i2 | brown | 0.8745261 |
| TRINITY_DN25587_c0_g1_i1 | brown | 0.8744137 |
| TRINITY_DN2748_c0_g2_i1 | brown | 0.8743825 |
| TRINITY_DN24972_c0_g1_i1 | brown | 0.87423 |
| TRINITY_DN32707_c0_g3_i1 | brown | 0.8741843 |
| TRINITY_DN31322_c1_g2_i1 | brown | 0.8739954 |
| TRINITY_DN33877_c0_g2_i1 | brown | 0.8739069 |
| TRINITY_DN35296_c0_g1_i3 | brown | 0.8737444 |
| TRINITY_DN29944_c0_g2_i1 | brown | 0.8737021 |
| TRINITY_DN21218_c0_g1_i2 | brown | 0.8735997 |
| TRINITY_DN36607_c0_g7_i1 | brown | 0.8731682 |
| TRINITY_DN1808_c1_g1_i1 | brown | 0.8727743 |
| TRINITY_DN16446_c0_g1_i1 | brown | 0.8726731 |
| TRINITY_DN26422_c0_g1_i2 | brown | 0.8726118 |
| TRINITY_DN32723_c0_g1_i1 | brown | 0.8725766 |
| TRINITY_DN29431_c0_g1_i3 | brown | 0.8723374 |
| TRINITY_DN36198_c1_g1_i2 | brown | 0.8722227 |
| TRINITY_DN34435_c0_g1_i2 | brown | 0.8721796 |
| TRINITY_DN35548_c0_g1_i1 | brown | 0.8718527 |
| TRINITY_DN31667_c0_g4_i2 | brown | 0.8717645 |
| TRINITY_DN37476_c0_g2_i1 | brown | 0.8716497 |
| TRINITY_DN38951_c1_g1_i1 | brown | 0.8714129 |
| TRINITY_DN34513_c0_g2_i1 | brown | 0.8713377 |
| TRINITY_DN30876_c1_g10_i2 | brown | 0.8709712 |
| TRINITY_DN27042_c1_g2_i1 | brown | 0.870946 |
| TRINITY_DN38295_c3_g10_i1 | brown | 0.870327 |
| TRINITY_DN35526_c2_g6_i1 | brown | 0.8702733 |
| TRINITY_DN18444_c0_g3_i1 | brown | 0.8702545 |
| TRINITY_DN22052_c0_g2_i1 | brown | 0.8701874 |
| TRINITY_DN34129_c0_g3_i1 | brown | 0.8692992 |
| TRINITY_DN2310_c0_g1_i1 | brown | 0.8691558 |
| TRINITY_DN34778_c0_g4_i1 | brown | 0.8691384 |
| TRINITY_DN30403_c1_g4_i4 | brown | 0.8690875 |
| TRINITY_DN20227_c0_g1_i1 | brown | 0.86848 |
| TRINITY_DN23411_c0_g1_i1 | brown | 0.8683595 |
| TRINITY_DN28268_c0_g9_i1 | brown | 0.8683587 |
| TRINITY_DN9197_c0_g1_i1 | brown | 0.868287 |
| TRINITY_DN37541_c1_g1_i12 | brown | 0.8681676 |
| TRINITY_DN29386_c0_g1_i1 | brown | 0.8679336 |
| TRINITY_DN37588_c0_g1_i2 | brown | 0.86775 |
| TRINITY_DN54659_c0_g1_i1 | brown | 0.8674521 |
| TRINITY_DN38586_c3_g3_i1 | brown | 0.867381 |
| TRINITY_DN25671_c0_g3_i1 | brown | 0.8668248 |
| TRINITY_DN51145_c0_g1_i1 | brown | 0.8667474 |
| TRINITY_DN33163_c0_g3_i1 | brown | 0.8666714 |
| TRINITY_DN19317_c0_g1_i1 | brown | 0.866579 |
| TRINITY_DN36600_c0_g1_i1 | brown | 0.8665013 |
| TRINITY_DN38841_c0_g8_i1 | brown | 0.8663349 |
| TRINITY_DN23434_c0_g4_i1 | brown | 0.8662064 |
| TRINITY_DN37617_c0_g12_i1 | brown | 0.8659683 |
| TRINITY_DN35456_c2_g6_i1 | brown | 0.8659066 |
| TRINITY_DN22627_c0_g1_i1 | brown | 0.8658798 |
| TRINITY_DN38411_c0_g2_i1 | brown | 0.8656974 |
| TRINITY_DN51649_c0_g1_i1 | brown | 0.8654251 |
| TRINITY_DN38685_c1_g1_i1 | brown | 0.8653309 |
| TRINITY_DN39130_c0_g1_i2 | brown | 0.8653028 |
| TRINITY_DN26026_c0_g3_i1 | brown | 0.8652184 |
| TRINITY_DN14523_c0_g1_i1 | brown | 0.8650933 |
| TRINITY_DN39205_c1_g2_i1 | brown | 0.8650126 |
| TRINITY_DN34618_c1_g6_i1 | brown | 0.8649757 |
| TRINITY_DN20164_c0_g2_i1 | brown | 0.8649457 |
| TRINITY_DN12853_c0_g1_i1 | brown | 0.8644582 |
| TRINITY_DN25869_c0_g1_i1 | brown | 0.8643851 |
| TRINITY_DN23794_c0_g1_i1 | brown | 0.8641187 |
| TRINITY_DN37620_c3_g1_i1 | brown | 0.863917 |
| TRINITY_DN33077_c1_g4_i1 | brown | 0.8637388 |
| TRINITY_DN32128_c0_g1_i5 | brown | 0.8636693 |
| TRINITY_DN31422_c1_g1_i2 | brown | 0.8635716 |
| TRINITY_DN38830_c1_g7_i1 | brown | 0.863347 |
| TRINITY_DN29653_c0_g1_i2 | brown | 0.8633242 |
| TRINITY_DN22150_c0_g3_i1 | brown | 0.863224 |
| TRINITY_DN9125_c0_g1_i1 | brown | 0.8631553 |
| TRINITY_DN28414_c0_g7_i1 | brown | 0.8631135 |
| TRINITY_DN26586_c0_g1_i2 | brown | 0.8630601 |
| TRINITY_DN38689_c1_g3_i1 | brown | 0.8630028 |
| TRINITY_DN16384_c0_g2_i1 | brown | 0.8627141 |
| TRINITY_DN37018_c0_g4_i5 | brown | 0.8627106 |
| TRINITY_DN21226_c0_g1_i2 | brown | 0.8626317 |
| TRINITY_DN36407_c1_g3_i19 | brown | 0.8623767 |
| TRINITY_DN36047_c1_g2_i3 | brown | 0.8620107 |
| TRINITY_DN30063_c0_g1_i1 | brown | 0.861472 |
| TRINITY_DN30843_c0_g19_i1 | brown | 0.8613878 |
| TRINITY_DN33526_c0_g2_i3 | brown | 0.8613462 |
| TRINITY_DN29142_c0_g2_i5 | brown | 0.860574 |
| TRINITY_DN36019_c0_g5_i4 | brown | 0.8604203 |
| TRINITY_DN38807_c0_g2_i1 | brown | 0.8603335 |
| TRINITY_DN27750_c0_g5_i1 | brown | 0.8601267 |
| TRINITY_DN30876_c1_g1_i2 | brown | 0.859981 |
| TRINITY_DN32879_c1_g1_i1 | brown | 0.8598193 |
| TRINITY_DN31563_c0_g1_i1 | brown | 0.8596367 |
| TRINITY_DN26966_c0_g1_i1 | brown | 0.8595387 |
| TRINITY_DN38543_c1_g8_i2 | brown | 0.8593478 |
| TRINITY_DN33830_c0_g1_i1 | brown | 0.8592999 |
| TRINITY_DN22520_c0_g1_i1 | brown | 0.8592979 |
| TRINITY_DN39137_c3_g3_i2 | brown | 0.8592735 |
| TRINITY_DN37750_c0_g2_i1 | brown | 0.8587781 |
| TRINITY_DN21230_c0_g1_i1 | brown | 0.8586006 |
| TRINITY_DN57627_c0_g1_i1 | brown | 0.8585607 |
| TRINITY_DN38299_c2_g5_i1 | brown | 0.8580897 |
| TRINITY_DN36593_c0_g9_i1 | brown | 0.8578925 |
| TRINITY_DN23203_c0_g1_i1 | brown | 0.8578096 |
| TRINITY_DN28470_c0_g1_i3 | brown | 0.8576305 |
| TRINITY_DN22134_c0_g1_i1 | brown | 0.8574589 |
| TRINITY_DN32623_c0_g5_i1 | brown | 0.857435 |
| TRINITY_DN32954_c0_g1_i2 | brown | 0.8572361 |
| TRINITY_DN32042_c0_g1_i3 | brown | 0.8571988 |
| TRINITY_DN32047_c0_g6_i1 | brown | 0.8571561 |
| TRINITY_DN34261_c1_g10_i1 | brown | 0.8571436 |
| TRINITY_DN31118_c0_g1_i2 | brown | 0.8570164 |
| TRINITY_DN35818_c0_g1_i6 | brown | 0.8569772 |
| TRINITY_DN35851_c3_g1_i3 | brown | 0.8569186 |
| TRINITY_DN9253_c0_g1_i1 | brown | 0.8568173 |
| TRINITY_DN39285_c1_g8_i1 | brown | 0.8568028 |
| TRINITY_DN38112_c0_g3_i1 | brown | 0.8565351 |
| TRINITY_DN25858_c1_g3_i1 | brown | 0.8563493 |
| TRINITY_DN16034_c0_g1_i1 | brown | 0.8562935 |
| TRINITY_DN37809_c0_g6_i1 | brown | 0.8560613 |
| TRINITY_DN25757_c0_g1_i1 | brown | 0.8559312 |
| TRINITY_DN38419_c1_g1_i6 | brown | 0.8558183 |
| TRINITY_DN35036_c1_g1_i3 | brown | 0.8557414 |
| TRINITY_DN28050_c0_g1_i6 | brown | 0.8556037 |
| TRINITY_DN39125_c0_g4_i5 | brown | 0.8555926 |
| TRINITY_DN36465_c0_g5_i1 | brown | 0.855571 |
| TRINITY_DN23517_c0_g1_i2 | brown | 0.8547246 |
| TRINITY_DN38251_c1_g2_i5 | brown | 0.8546798 |
| TRINITY_DN37196_c0_g2_i2 | brown | 0.854575 |
| TRINITY_DN28412_c0_g2_i1 | brown | 0.8543 |
| TRINITY_DN30843_c0_g3_i1 | brown | 0.8539538 |
| TRINITY_DN38113_c1_g1_i1 | brown | 0.8539283 |
| TRINITY_DN21196_c0_g2_i1 | brown | 0.853631 |
| TRINITY_DN28679_c0_g1_i1 | brown | 0.853098 |
| TRINITY_DN39261_c3_g1_i1 | brown | 0.8529633 |
| TRINITY_DN25541_c1_g3_i1 | brown | 0.8529282 |
| TRINITY_DN38295_c0_g2_i1 | brown | 0.8524256 |
| TRINITY_DN34020_c0_g1_i10 | brown | 0.852348 |
| TRINITY_DN20291_c0_g1_i1 | brown | 0.8521627 |
| TRINITY_DN31240_c0_g6_i1 | brown | 0.852028 |
| TRINITY_DN48590_c0_g1_i1 | brown | 0.851825 |
| TRINITY_DN26802_c0_g1_i1 | brown | 0.8516853 |
| TRINITY_DN38355_c0_g5_i1 | brown | 0.8512829 |
| TRINITY_DN26386_c0_g1_i4 | brown | 0.8511725 |
| TRINITY_DN33510_c0_g3_i7 | brown | 0.8504588 |
| TRINITY_DN35846_c1_g4_i1 | brown | 0.8501975 |
| TRINITY_DN24953_c0_g1_i1 | brown | 0.8501104 |
| TRINITY_DN57092_c0_g1_i1 | brown | 0.849383 |
| TRINITY_DN23968_c0_g3_i1 | brown | 0.8492724 |
| TRINITY_DN34395_c0_g4_i1 | brown | 0.8491579 |
| TRINITY_DN39180_c8_g16_i1 | brown | 0.8491326 |
| TRINITY_DN26851_c0_g1_i5 | brown | 0.8489652 |
| TRINITY_DN33914_c1_g2_i1 | brown | 0.8485257 |
| TRINITY_DN37668_c0_g2_i1 | brown | 0.8484669 |
| TRINITY_DN26133_c0_g2_i1 | brown | 0.8482939 |
| TRINITY_DN39175_c0_g2_i1 | brown | 0.8471541 |
| TRINITY_DN38788_c0_g4_i2 | brown | 0.847065 |
| TRINITY_DN17602_c0_g1_i1 | brown | 0.8468923 |
| TRINITY_DN26127_c0_g1_i1 | brown | 0.8468169 |
| TRINITY_DN35348_c0_g1_i1 | brown | 0.8466213 |
| TRINITY_DN27722_c1_g1_i4 | brown | 0.845973 |
| TRINITY_DN28765_c0_g1_i3 | brown | 0.8459121 |
| TRINITY_DN10882_c0_g1_i1 | brown | 0.8453449 |
| TRINITY_DN12429_c0_g2_i1 | brown | 0.8453155 |
| TRINITY_DN34904_c0_g2_i1 | brown | 0.8450679 |
| TRINITY_DN36052_c1_g1_i9 | brown | 0.8445961 |
| TRINITY_DN29301_c0_g1_i1 | brown | 0.8437658 |
| TRINITY_DN35722_c0_g2_i12 | brown | 0.8432867 |
| TRINITY_DN16174_c0_g2_i1 | brown | 0.8432009 |
| TRINITY_DN44405_c0_g1_i1 | brown | 0.8428097 |
| TRINITY_DN36154_c0_g4_i1 | brown | 0.8425659 |
| TRINITY_DN34088_c0_g2_i2 | brown | 0.8422886 |
| TRINITY_DN39220_c6_g6_i2 | brown | 0.8422236 |
| TRINITY_DN36596_c0_g1_i2 | brown | 0.8421468 |
| TRINITY_DN25876_c0_g3_i1 | brown | 0.8418831 |
| TRINITY_DN35289_c0_g2_i5 | brown | 0.8418641 |
| TRINITY_DN37096_c0_g5_i1 | brown | 0.8417424 |
| TRINITY_DN22418_c0_g2_i1 | brown | 0.8410554 |
| TRINITY_DN22672_c0_g1_i1 | brown | 0.8410196 |
| TRINITY_DN30074_c1_g5_i1 | brown | 0.8405034 |
| TRINITY_DN22363_c0_g3_i1 | brown | 0.8401462 |
| TRINITY_DN34887_c1_g1_i10 | brown | 0.8399003 |
| TRINITY_DN35649_c0_g4_i1 | brown | 0.8398667 |
| TRINITY_DN25340_c0_g1_i1 | brown | 0.8398189 |
| TRINITY_DN33518_c1_g2_i5 | brown | 0.8397035 |
| TRINITY_DN20009_c0_g2_i1 | brown | 0.8396805 |
| TRINITY_DN20435_c0_g1_i1 | brown | 0.8396302 |
| TRINITY_DN37945_c1_g1_i1 | brown | 0.8395498 |
| TRINITY_DN8397_c0_g1_i1 | brown | 0.8394678 |
| TRINITY_DN35991_c0_g1_i1 | brown | 0.839388 |
| TRINITY_DN22206_c0_g4_i1 | brown | 0.8391891 |
| TRINITY_DN35750_c0_g1_i2 | brown | 0.8390254 |
| TRINITY_DN27620_c0_g3_i1 | brown | 0.8388398 |
| TRINITY_DN39098_c1_g2_i4 | brown | 0.8387318 |
| TRINITY_DN17717_c0_g1_i1 | brown | 0.838718 |
| TRINITY_DN25527_c0_g1_i1 | brown | 0.8381173 |
| TRINITY_DN34970_c0_g3_i6 | brown | 0.8377977 |
| TRINITY_DN36819_c0_g1_i3 | brown | 0.8366935 |
| TRINITY_DN22201_c0_g1_i1 | brown | 0.8363172 |
| TRINITY_DN26778_c0_g1_i1 | brown | 0.8362647 |
| TRINITY_DN31833_c1_g1_i17 | brown | 0.8362334 |
| TRINITY_DN33189_c0_g3_i1 | brown | 0.8360719 |
| TRINITY_DN9087_c0_g3_i1 | brown | 0.8354665 |
| TRINITY_DN37997_c1_g1_i3 | brown | 0.8352699 |
| TRINITY_DN52520_c0_g1_i1 | brown | 0.8351197 |
| TRINITY_DN23185_c0_g2_i1 | brown | 0.8350827 |
| TRINITY_DN22153_c0_g1_i1 | brown | 0.8349014 |
| TRINITY_DN16461_c0_g2_i1 | brown | 0.8346642 |
| TRINITY_DN28787_c0_g2_i2 | brown | 0.8346439 |
| TRINITY_DN36314_c0_g2_i4 | brown | 0.8345831 |
| TRINITY_DN29788_c0_g1_i1 | brown | 0.8345592 |
| TRINITY_DN25284_c0_g1_i1 | brown | 0.8338733 |
| TRINITY_DN21977_c0_g1_i2 | brown | 0.8338121 |
| TRINITY_DN38860_c1_g4_i5 | brown | 0.8336859 |
| TRINITY_DN39205_c1_g6_i1 | brown | 0.8336083 |
| TRINITY_DN32577_c0_g5_i1 | brown | 0.8334714 |
| TRINITY_DN34890_c1_g2_i3 | brown | 0.8332705 |
| TRINITY_DN35488_c0_g2_i1 | brown | 0.832988 |
| TRINITY_DN31814_c1_g3_i3 | brown | 0.8329529 |
| TRINITY_DN37983_c2_g1_i4 | brown | 0.8325042 |
| TRINITY_DN23691_c0_g1_i1 | brown | 0.8324441 |
| TRINITY_DN37736_c0_g3_i1 | brown | 0.8320512 |
| TRINITY_DN24851_c0_g1_i1 | brown | 0.8318011 |
| TRINITY_DN27291_c0_g3_i1 | brown | 0.8310127 |
| TRINITY_DN36444_c0_g2_i3 | brown | 0.8309279 |
| TRINITY_DN35537_c1_g5_i8 | brown | 0.8307808 |
| TRINITY_DN38489_c0_g2_i4 | brown | 0.8307734 |
| TRINITY_DN23801_c0_g1_i1 | brown | 0.8306828 |
| TRINITY_DN33531_c0_g3_i1 | brown | 0.8304738 |
| TRINITY_DN34767_c0_g1_i1 | brown | 0.8304073 |
| TRINITY_DN38272_c1_g3_i6 | brown | 0.8299482 |
| TRINITY_DN32311_c0_g2_i5 | brown | 0.829673 |
| TRINITY_DN28186_c0_g2_i2 | brown | 0.8294697 |
| TRINITY_DN35671_c0_g5_i1 | brown | 0.8294359 |
| TRINITY_DN26771_c0_g1_i1 | brown | 0.8291818 |
| TRINITY_DN35247_c0_g2_i1 | brown | 0.829122 |
| TRINITY_DN18484_c0_g2_i1 | brown | 0.8287906 |
| TRINITY_DN35561_c0_g2_i1 | brown | 0.8279042 |
| TRINITY_DN34780_c0_g1_i1 | brown | 0.8270241 |
| TRINITY_DN39326_c10_g16_i1 | brown | 0.8265331 |
| TRINITY_DN39704_c0_g1_i1 | brown | 0.8262527 |
| TRINITY_DN20965_c0_g1_i1 | brown | 0.8261443 |
| TRINITY_DN14725_c0_g1_i1 | brown | 0.8261224 |
| TRINITY_DN41617_c0_g1_i1 | brown | 0.8260554 |
| TRINITY_DN34496_c0_g6_i1 | brown | 0.8258317 |
| TRINITY_DN35155_c0_g1_i6 | brown | 0.8257813 |
| TRINITY_DN10328_c0_g1_i1 | brown | 0.8252669 |
| TRINITY_DN37725_c0_g2_i2 | brown | 0.8247711 |
| TRINITY_DN27389_c0_g1_i4 | brown | 0.8243967 |
| TRINITY_DN35785_c0_g1_i1 | brown | 0.8243108 |
| TRINITY_DN30154_c0_g1_i2 | brown | 0.8239768 |
| TRINITY_DN18769_c0_g2_i1 | brown | 0.8236789 |
| TRINITY_DN16114_c0_g1_i1 | brown | 0.8235007 |
| TRINITY_DN17433_c0_g1_i1 | brown | 0.8234626 |
| TRINITY_DN29402_c0_g1_i8 | brown | 0.8233282 |
| TRINITY_DN38649_c1_g3_i1 | brown | 0.823327 |
| TRINITY_DN68944_c0_g1_i1 | brown | 0.8231684 |
| TRINITY_DN38000_c0_g3_i1 | brown | 0.8230916 |
| TRINITY_DN25722_c0_g1_i1 | brown | 0.8229709 |
| TRINITY_DN23986_c0_g4_i1 | brown | 0.8221626 |
| TRINITY_DN31951_c0_g6_i1 | brown | 0.8221465 |
| TRINITY_DN34038_c0_g2_i1 | brown | 0.8220465 |
| TRINITY_DN37615_c1_g1_i7 | brown | 0.821964 |
| TRINITY_DN24148_c0_g1_i1 | brown | 0.8217475 |
| TRINITY_DN38295_c3_g3_i10 | brown | 0.8217171 |
| TRINITY_DN39182_c1_g4_i1 | brown | 0.821642 |
| TRINITY_DN19897_c0_g1_i1 | brown | 0.8216131 |
| TRINITY_DN24306_c0_g2_i1 | brown | 0.8214681 |
| TRINITY_DN33180_c2_g1_i4 | brown | 0.8212399 |
| TRINITY_DN30580_c0_g2_i4 | brown | 0.8208482 |
| TRINITY_DN37162_c0_g4_i1 | brown | 0.8193941 |
| TRINITY_DN22509_c0_g1_i1 | brown | 0.8192571 |
| TRINITY_DN18055_c0_g1_i1 | brown | 0.8180284 |
| TRINITY_DN25669_c0_g4_i1 | brown | 0.817001 |
| TRINITY_DN31773_c2_g3_i1 | brown | 0.8164642 |
| TRINITY_DN46289_c0_g1_i1 | brown | 0.8161542 |
| TRINITY_DN24708_c0_g1_i1 | brown | 0.8158953 |
| TRINITY_DN28308_c0_g1_i1 | brown | 0.8158818 |
| TRINITY_DN28439_c0_g6_i1 | brown | 0.8157965 |
| TRINITY_DN38057_c0_g5_i1 | brown | 0.8153681 |
| TRINITY_DN32255_c0_g2_i4 | brown | 0.8147591 |
| TRINITY_DN25593_c0_g3_i1 | brown | 0.814588 |
| TRINITY_DN33376_c0_g3_i3 | brown | 0.8143496 |
| TRINITY_DN33851_c0_g2_i1 | brown | 0.8136342 |
| TRINITY_DN24664_c0_g1_i1 | brown | 0.8132573 |
| TRINITY_DN31099_c0_g1_i1 | brown | 0.8123916 |
| TRINITY_DN30189_c0_g4_i1 | brown | 0.8123333 |
| TRINITY_DN28186_c0_g1_i1 | brown | 0.8122707 |
| TRINITY_DN35324_c0_g6_i1 | brown | 0.8116915 |
| TRINITY_DN35895_c0_g2_i1 | brown | 0.8107029 |
| TRINITY_DN25285_c0_g1_i1 | brown | 0.8103235 |
| TRINITY_DN20669_c0_g1_i2 | brown | 0.8096409 |
| TRINITY_DN38182_c0_g2_i1 | brown | 0.8090163 |
| TRINITY_DN26629_c0_g1_i2 | brown | 0.8087711 |
| TRINITY_DN39200_c1_g4_i1 | brown | 0.8083966 |
| TRINITY_DN25787_c0_g2_i1 | brown | 0.8083699 |
| TRINITY_DN37800_c0_g2_i4 | brown | 0.8082362 |
| TRINITY_DN19530_c0_g3_i1 | brown | 0.8081851 |
| TRINITY_DN24150_c0_g1_i1 | brown | 0.8080178 |
| TRINITY_DN32431_c0_g1_i1 | brown | 0.8078864 |
| TRINITY_DN23606_c0_g1_i1 | brown | 0.8076151 |
| TRINITY_DN26890_c0_g1_i3 | brown | 0.8070027 |
| TRINITY_DN38940_c0_g1_i1 | brown | 0.8058472 |
| TRINITY_DN37501_c0_g1_i3 | brown | 0.8058452 |
| TRINITY_DN39310_c0_g5_i3 | brown | 0.805622 |
| TRINITY_DN20085_c0_g1_i1 | brown | 0.8050079 |
| TRINITY_DN37356_c1_g1_i1 | brown | 0.8047536 |
| TRINITY_DN34156_c1_g2_i1 | brown | 0.8045778 |
| TRINITY_DN39076_c5_g4_i1 | brown | 0.8038448 |
| TRINITY_DN38951_c1_g6_i1 | brown | 0.8036179 |
| TRINITY_DN24360_c0_g1_i1 | brown | 0.8016169 |
| TRINITY_DN41952_c0_g1_i1 | brown | 0.801605 |
| TRINITY_DN16528_c0_g1_i1 | brown | 0.8014279 |
| TRINITY_DN38788_c0_g2_i1 | brown | 0.7999647 |
| TRINITY_DN27085_c0_g1_i1 | brown | 0.7995863 |
| TRINITY_DN28526_c0_g1_i1 | brown | 0.7994199 |
| TRINITY_DN31908_c0_g2_i2 | brown | 0.7993367 |
| TRINITY_DN29214_c0_g1_i1 | brown | 0.7990626 |
| TRINITY_DN19618_c0_g1_i1 | brown | 0.7989859 |
| TRINITY_DN20783_c0_g1_i1 | brown | 0.7989439 |
| TRINITY_DN16461_c0_g3_i1 | brown | 0.7986698 |
| TRINITY_DN35135_c0_g3_i1 | brown | 0.7984319 |
| TRINITY_DN31213_c0_g4_i1 | brown | 0.7982688 |
| TRINITY_DN22331_c0_g1_i1 | brown | 0.7975987 |
| TRINITY_DN28722_c1_g1_i1 | brown | 0.7973923 |
| TRINITY_DN23279_c0_g1_i2 | brown | 0.7969675 |
| TRINITY_DN4582_c0_g1_i1 | brown | 0.7967956 |
| TRINITY_DN20882_c0_g1_i1 | brown | 0.7959235 |
| TRINITY_DN27035_c0_g2_i4 | brown | 0.7957722 |
| TRINITY_DN29048_c0_g5_i1 | brown | 0.7956691 |
| TRINITY_DN39300_c6_g9_i1 | brown | 0.7951925 |
| TRINITY_DN19950_c0_g1_i1 | brown | 0.7937423 |
| TRINITY_DN33532_c0_g1_i4 | brown | 0.7935971 |
| TRINITY_DN22225_c0_g3_i1 | brown | 0.7927671 |
| TRINITY_DN765_c0_g1_i1 | brown | 0.7906471 |
| TRINITY_DN20542_c1_g1_i1 | brown | 0.7905495 |
| TRINITY_DN28201_c0_g1_i5 | brown | 0.7898871 |
| TRINITY_DN34215_c2_g2_i1 | brown | 0.7898524 |
| TRINITY_DN34543_c0_g1_i1 | brown | 0.7898248 |
| TRINITY_DN56249_c0_g1_i1 | brown | 0.7895801 |
| TRINITY_DN50770_c0_g1_i1 | brown | 0.7891472 |
| TRINITY_DN25999_c0_g3_i2 | brown | 0.7890745 |
| TRINITY_DN35900_c0_g1_i4 | brown | 0.7877299 |
| TRINITY_DN39211_c1_g3_i1 | brown | 0.7858238 |
| TRINITY_DN34770_c1_g3_i1 | brown | 0.7857535 |
| TRINITY_DN30281_c0_g2_i1 | brown | 0.7856564 |
| TRINITY_DN39206_c4_g2_i10 | brown | 0.7847804 |
| TRINITY_DN36581_c3_g15_i1 | brown | 0.7841201 |
| TRINITY_DN31235_c0_g1_i1 | brown | 0.7838373 |
| TRINITY_DN31824_c0_g2_i1 | brown | 0.7836946 |
| TRINITY_DN38295_c3_g2_i1 | brown | 0.7828613 |
| TRINITY_DN31648_c0_g3_i1 | brown | 0.7828144 |
| TRINITY_DN36146_c0_g1_i1 | brown | 0.7827106 |
| TRINITY_DN24794_c1_g2_i1 | brown | 0.7825434 |
| TRINITY_DN34300_c2_g1_i21 | brown | 0.7825187 |
| TRINITY_DN9811_c0_g2_i1 | brown | 0.7815648 |
| TRINITY_DN39261_c3_g2_i2 | brown | 0.7814452 |
| TRINITY_DN35956_c0_g2_i2 | brown | 0.7810927 |
| TRINITY_DN29942_c0_g3_i2 | brown | 0.7808087 |
| TRINITY_DN40138_c0_g1_i1 | brown | 0.7801942 |
| TRINITY_DN28054_c0_g1_i1 | brown | 0.7800569 |
| TRINITY_DN38047_c1_g1_i1 | brown | 0.7798552 |
| TRINITY_DN4349_c0_g1_i1 | brown | 0.778074 |
| TRINITY_DN22106_c0_g2_i1 | brown | 0.7768539 |
| TRINITY_DN34714_c3_g1_i2 | brown | 0.7762637 |
| TRINITY_DN34398_c0_g1_i9 | brown | 0.7761734 |
| TRINITY_DN32118_c1_g1_i2 | brown | 0.7758233 |
| TRINITY_DN26303_c0_g1_i2 | brown | 0.7742968 |
| TRINITY_DN34650_c0_g2_i1 | brown | 0.7741251 |
| TRINITY_DN22435_c0_g3_i1 | brown | 0.7740197 |
| TRINITY_DN34283_c0_g1_i3 | brown | 0.773427 |
| TRINITY_DN23989_c0_g1_i2 | brown | 0.7731684 |
| TRINITY_DN11264_c0_g2_i1 | brown | 0.7730729 |
| TRINITY_DN30181_c0_g3_i1 | brown | 0.7728854 |
| TRINITY_DN21509_c0_g2_i1 | brown | 0.7726691 |
| TRINITY_DN64964_c0_g1_i1 | brown | 0.772599 |
| TRINITY_DN28029_c0_g5_i1 | brown | 0.7718611 |
| TRINITY_DN37029_c0_g6_i1 | brown | 0.7716976 |
| TRINITY_DN70777_c0_g1_i1 | brown | 0.771561 |
| TRINITY_DN24639_c0_g1_i1 | brown | 0.7712388 |
| TRINITY_DN32984_c0_g2_i1 | brown | 0.7689196 |
| TRINITY_DN19103_c0_g2_i1 | brown | 0.7677624 |
| TRINITY_DN18511_c0_g1_i1 | brown | 0.767419 |
| TRINITY_DN31790_c0_g1_i3 | brown | 0.7671838 |
| TRINITY_DN28551_c0_g3_i3 | brown | 0.7660775 |
| TRINITY_DN21554_c0_g2_i2 | brown | 0.7655335 |
| TRINITY_DN22273_c0_g1_i1 | brown | 0.7647565 |
| TRINITY_DN42720_c0_g1_i1 | brown | 0.7640575 |
| TRINITY_DN32283_c0_g1_i13 | brown | 0.763814 |
| TRINITY_DN45509_c0_g1_i1 | brown | 0.7637825 |
| TRINITY_DN35976_c0_g3_i1 | brown | 0.762969 |
| TRINITY_DN71747_c0_g1_i1 | brown | 0.7629638 |
| TRINITY_DN20767_c0_g2_i1 | brown | 0.7613033 |
| TRINITY_DN39208_c0_g1_i1 | brown | 0.7610749 |
| TRINITY_DN12944_c0_g1_i1 | brown | 0.7606084 |
| TRINITY_DN35095_c0_g1_i1 | brown | 0.7602741 |
| TRINITY_DN30011_c4_g1_i2 | brown | 0.7596192 |
| TRINITY_DN20898_c0_g1_i3 | brown | 0.7595578 |
| TRINITY_DN20604_c0_g2_i1 | brown | 0.7585956 |
| TRINITY_DN37489_c2_g8_i1 | brown | 0.7575436 |
| TRINITY_DN38681_c1_g4_i1 | brown | 0.7559265 |
| TRINITY_DN28806_c0_g1_i5 | brown | 0.7559133 |
| TRINITY_DN39282_c2_g4_i6 | brown | 0.7554699 |
| TRINITY_DN22769_c0_g2_i2 | brown | 0.7554221 |
| TRINITY_DN58449_c0_g1_i1 | brown | 0.7550631 |
| TRINITY_DN39241_c3_g3_i1 | brown | 0.7545334 |
| TRINITY_DN34385_c2_g8_i1 | brown | 0.7539273 |
| TRINITY_DN32353_c0_g1_i1 | brown | 0.7538094 |
| TRINITY_DN23860_c0_g1_i3 | brown | 0.7533835 |
| TRINITY_DN37921_c2_g1_i6 | brown | 0.7513284 |
| TRINITY_DN20594_c0_g1_i1 | brown | 0.7512682 |
| TRINITY_DN34361_c0_g2_i1 | brown | 0.7473591 |
| TRINITY_DN40911_c0_g1_i1 | brown | 0.7468636 |
| TRINITY_DN21101_c0_g1_i1 | brown | 0.7440666 |
| TRINITY_DN37370_c0_g2_i3 | brown | 0.7419991 |
| TRINITY_DN31297_c0_g1_i1 | brown | 0.7417185 |
| TRINITY_DN8803_c0_g1_i1 | brown | 0.7389712 |
| TRINITY_DN9125_c0_g2_i1 | brown | 0.7389531 |
| TRINITY_DN29760_c0_g2_i1 | brown | 0.7387452 |
| TRINITY_DN23454_c0_g3_i1 | brown | 0.7382931 |
| TRINITY_DN39199_c1_g2_i1 | brown | 0.7381247 |
| TRINITY_DN10339_c0_g2_i1 | brown | 0.7373585 |
| TRINITY_DN41739_c0_g1_i1 | brown | 0.7373321 |
| TRINITY_DN36993_c0_g2_i1 | brown | 0.7372522 |
| TRINITY_DN37922_c0_g2_i7 | brown | 0.7357336 |
| TRINITY_DN28461_c0_g1_i1 | brown | 0.735515 |
| TRINITY_DN20467_c0_g1_i1 | brown | 0.7334021 |
| TRINITY_DN62647_c0_g1_i1 | brown | 0.7330672 |
| TRINITY_DN27747_c0_g2_i1 | brown | 0.7274537 |
| TRINITY_DN35179_c0_g1_i3 | brown | 0.7243452 |
| TRINITY_DN36952_c0_g11_i1 | brown | 0.7218264 |
| TRINITY_DN17888_c0_g1_i1 | brown | 0.7208866 |
| TRINITY_DN27102_c0_g1_i1 | brown | 0.7207896 |
| TRINITY_DN25126_c0_g1_i1 | brown | 0.7197809 |
| TRINITY_DN33995_c0_g4_i1 | brown | 0.7195455 |
| TRINITY_DN39069_c3_g23_i1 | brown | 0.717545 |
| TRINITY_DN51379_c0_g1_i1 | brown | 0.7165063 |
| TRINITY_DN35893_c0_g2_i2 | brown | 0.715614 |
| TRINITY_DN25484_c0_g2_i1 | brown | 0.7137599 |
| TRINITY_DN57619_c0_g1_i1 | brown | 0.712347 |
| TRINITY_DN28730_c0_g2_i1 | brown | 0.7121417 |
| TRINITY_DN35090_c0_g2_i1 | brown | 0.711395 |
| TRINITY_DN32346_c0_g1_i2 | brown | 0.7107417 |
| TRINITY_DN25477_c0_g1_i1 | brown | 0.709331 |
| TRINITY_DN21681_c0_g3_i1 | brown | 0.7058726 |
| TRINITY_DN39657_c0_g1_i1 | brown | 0.7043972 |
| TRINITY_DN32356_c0_g1_i7 | brown | 0.7028101 |
| TRINITY_DN23913_c0_g1_i1 | brown | 0.702784 |
| TRINITY_DN36998_c0_g2_i1 | brown | 0.7023946 |
| TRINITY_DN36707_c0_g1_i6 | brown | 0.6994455 |
| TRINITY_DN31431_c0_g1_i4 | brown | 0.6944457 |
| TRINITY_DN24700_c0_g1_i1 | brown | 0.6931128 |
| TRINITY_DN27039_c0_g2_i1 | brown | 0.6882772 |
| TRINITY_DN36581_c3_g17_i1 | brown | 0.681719 |
| TRINITY_DN36546_c0_g2_i4 | brown | 0.6808565 |
| TRINITY_DN39164_c0_g3_i1 | brown | 0.6778286 |
| TRINITY_DN21363_c0_g3_i1 | brown | 0.6778104 |
| TRINITY_DN27366_c0_g4_i1 | brown | 0.6721722 |
| TRINITY_DN27949_c0_g1_i1 | brown | 0.6687632 |
| TRINITY_DN33183_c1_g2_i2 | brown | 0.6677521 |
| TRINITY_DN23264_c0_g2_i1 | brown | 0.6676643 |
| TRINITY_DN30634_c0_g1_i1 | brown | 0.6645957 |
| TRINITY_DN32847_c0_g2_i2 | brown | 0.661415 |
| TRINITY_DN35417_c0_g2_i1 | brown | 0.6534723 |
| TRINITY_DN18980_c0_g1_i1 | brown | 0.6478023 |
| TRINITY_DN29416_c0_g2_i2 | brown | 0.6358977 |
| TRINITY_DN36718_c0_g3_i1 | brown | 0.6322275 |
| TRINITY_DN17648_c0_g1_i1 | brown | 0.6305712 |
| TRINITY_DN30843_c0_g13_i1 | brown | 0.6131992 |
| TRINITY_DN18691_c0_g1_i1 | brown | 0.6099038 |
| TRINITY_DN28518_c0_g1_i2 | brown | 0.6060372 |
| TRINITY_DN17558_c0_g3_i1 | brown | 0.5992959 |
| TRINITY_DN27930_c0_g1_i1 | brown | 0.596395 |
| TRINITY_DN36126_c1_g1_i8 | brown | 0.5963795 |
| TRINITY_DN22978_c0_g2_i1 | brown | 0.5868992 |
| TRINITY_DN1516_c0_g1_i1 | brown | 0.4332665 |
| TRINITY_DN54114_c0_g1_i1 | brown | -0.430729 |
| TRINITY_DN51062_c0_g1_i1 | brown | -0.485144 |
| TRINITY_DN28360_c0_g1_i1 | brown | -0.518065 |
| TRINITY_DN65519_c0_g1_i1 | brown | -0.518098 |
| TRINITY_DN26032_c0_g1_i1 | brown | -0.535555 |
| TRINITY_DN38750_c0_g1_i9 | brown | -0.555313 |
| TRINITY_DN45048_c0_g1_i1 | brown | -0.560811 |
| TRINITY_DN26770_c0_g4_i1 | brown | -0.573114 |
| TRINITY_DN34084_c1_g5_i1 | brown | -0.573834 |
| TRINITY_DN28481_c0_g1_i1 | brown | -0.58216 |
| TRINITY_DN33293_c0_g3_i14 | brown | -0.594577 |
| TRINITY_DN28848_c0_g1_i3 | brown | -0.596969 |
| TRINITY_DN19152_c0_g1_i1 | brown | -0.601085 |
| TRINITY_DN37060_c0_g2_i3 | brown | -0.602988 |
| TRINITY_DN20034_c0_g1_i1 | brown | -0.605754 |
| TRINITY_DN32371_c0_g2_i7 | brown | -0.606251 |
| TRINITY_DN29954_c0_g1_i1 | brown | -0.615063 |
| TRINITY_DN24387_c0_g1_i1 | brown | -0.624421 |
| TRINITY_DN32702_c0_g2_i5 | brown | -0.634376 |
| TRINITY_DN23186_c0_g3_i1 | brown | -0.636438 |
| TRINITY_DN26892_c0_g5_i1 | brown | -0.638502 |
| TRINITY_DN49615_c0_g1_i1 | brown | -0.638671 |
| TRINITY_DN27962_c1_g1_i1 | brown | -0.639141 |
| TRINITY_DN67331_c0_g1_i1 | brown | -0.64322 |
| TRINITY_DN50450_c0_g1_i1 | brown | -0.644249 |
| TRINITY_DN31018_c0_g1_i2 | brown | -0.648322 |
| TRINITY_DN35947_c2_g2_i12 | brown | -0.649518 |
| TRINITY_DN29803_c0_g1_i1 | brown | -0.64964 |
| TRINITY_DN34107_c0_g1_i1 | brown | -0.649831 |
| TRINITY_DN16864_c0_g2_i1 | brown | -0.651147 |
| TRINITY_DN37093_c1_g2_i1 | brown | -0.654282 |
| TRINITY_DN27461_c2_g1_i1 | brown | -0.656354 |
| TRINITY_DN34503_c1_g4_i1 | brown | -0.657838 |
| TRINITY_DN63969_c0_g1_i1 | brown | -0.658786 |
| TRINITY_DN27920_c0_g1_i2 | brown | -0.661653 |
| TRINITY_DN37411_c4_g1_i1 | brown | -0.667012 |
| TRINITY_DN26192_c0_g1_i1 | brown | -0.667387 |
| TRINITY_DN26266_c0_g1_i1 | brown | -0.670924 |
| TRINITY_DN25019_c0_g1_i1 | brown | -0.678841 |
| TRINITY_DN35030_c1_g2_i2 | brown | -0.679325 |
| TRINITY_DN28481_c0_g2_i2 | brown | -0.682027 |
| TRINITY_DN27163_c0_g2_i3 | brown | -0.682137 |
| TRINITY_DN25776_c0_g1_i1 | brown | -0.682157 |
| TRINITY_DN36434_c0_g6_i1 | brown | -0.68363 |
| TRINITY_DN32942_c0_g3_i2 | brown | -0.684031 |
| TRINITY_DN35987_c0_g4_i1 | brown | -0.686655 |
| TRINITY_DN27815_c0_g2_i4 | brown | -0.692315 |
| TRINITY_DN25030_c0_g1_i1 | brown | -0.695277 |
| TRINITY_DN27684_c0_g1_i4 | brown | -0.696398 |
| TRINITY_DN13989_c0_g1_i1 | brown | -0.696617 |
| TRINITY_DN33522_c0_g1_i1 | brown | -0.700406 |
| TRINITY_DN22331_c0_g3_i1 | brown | -0.703461 |
| TRINITY_DN34704_c1_g2_i1 | brown | -0.70566 |
| TRINITY_DN67343_c0_g1_i1 | brown | -0.705899 |
| TRINITY_DN40848_c0_g1_i1 | brown | -0.706137 |
| TRINITY_DN36885_c1_g6_i1 | brown | -0.706854 |
| TRINITY_DN27102_c0_g2_i1 | brown | -0.707036 |
| TRINITY_DN25009_c0_g2_i2 | brown | -0.707199 |
| TRINITY_DN34304_c0_g6_i2 | brown | -0.711781 |
| TRINITY_DN35261_c0_g1_i18 | brown | -0.714115 |
| TRINITY_DN23507_c0_g1_i1 | brown | -0.715105 |
| TRINITY_DN33406_c0_g2_i2 | brown | -0.715338 |
| TRINITY_DN19912_c0_g1_i1 | brown | -0.715697 |
| TRINITY_DN25736_c0_g1_i1 | brown | -0.71585 |
| TRINITY_DN20242_c0_g2_i2 | brown | -0.716206 |
| TRINITY_DN16820_c0_g1_i1 | brown | -0.717133 |
| TRINITY_DN55969_c0_g1_i1 | brown | -0.718126 |
| TRINITY_DN25782_c0_g1_i1 | brown | -0.719049 |
| TRINITY_DN37255_c2_g1_i1 | brown | -0.72107 |
| TRINITY_DN24603_c0_g1_i1 | brown | -0.72231 |
| TRINITY_DN31388_c1_g3_i3 | brown | -0.722401 |
| TRINITY_DN31271_c0_g2_i1 | brown | -0.723146 |
| TRINITY_DN30802_c0_g9_i1 | brown | -0.723377 |
| TRINITY_DN35090_c0_g10_i1 | brown | -0.726225 |
| TRINITY_DN36331_c1_g4_i2 | brown | -0.726344 |
| TRINITY_DN19700_c0_g1_i1 | brown | -0.72684 |
| TRINITY_DN25312_c0_g1_i1 | brown | -0.731204 |
| TRINITY_DN38963_c0_g1_i10 | brown | -0.734037 |
| TRINITY_DN32235_c0_g2_i1 | brown | -0.74071 |
| TRINITY_DN28041_c0_g5_i1 | brown | -0.74077 |
| TRINITY_DN34693_c0_g1_i4 | brown | -0.7415 |
| TRINITY_DN37791_c2_g11_i1 | brown | -0.743669 |
| TRINITY_DN36339_c2_g1_i5 | brown | -0.744139 |
| TRINITY_DN10990_c0_g1_i1 | brown | -0.746037 |
| TRINITY_DN19905_c0_g1_i1 | brown | -0.746878 |
| TRINITY_DN37093_c1_g1_i3 | brown | -0.751076 |
| TRINITY_DN31902_c1_g2_i2 | brown | -0.75127 |
| TRINITY_DN27913_c0_g2_i1 | brown | -0.751648 |
| TRINITY_DN16592_c0_g2_i1 | brown | -0.752245 |
| TRINITY_DN19036_c0_g1_i1 | brown | -0.752948 |
| TRINITY_DN28374_c1_g1_i1 | brown | -0.753451 |
| TRINITY_DN13727_c0_g1_i1 | brown | -0.755024 |
| TRINITY_DN24641_c0_g1_i1 | brown | -0.755436 |
| TRINITY_DN29210_c0_g2_i1 | brown | -0.7565 |
| TRINITY_DN26470_c0_g1_i2 | brown | -0.758833 |
| TRINITY_DN35327_c1_g1_i4 | brown | -0.758898 |
| TRINITY_DN34121_c0_g4_i2 | brown | -0.76003 |
| TRINITY_DN31031_c0_g1_i1 | brown | -0.761878 |
| TRINITY_DN265_c0_g1_i1 | brown | -0.763057 |
| TRINITY_DN32844_c0_g2_i3 | brown | -0.765423 |
| TRINITY_DN31616_c1_g1_i1 | brown | -0.765895 |
| TRINITY_DN83_c0_g2_i1 | brown | -0.769065 |
| TRINITY_DN62333_c0_g1_i1 | brown | -0.77297 |
| TRINITY_DN24088_c0_g1_i1 | brown | -0.772983 |
| TRINITY_DN38845_c2_g3_i5 | brown | -0.773934 |
| TRINITY_DN36695_c0_g7_i1 | brown | -0.775881 |
| TRINITY_DN37067_c0_g1_i9 | brown | -0.776669 |
| TRINITY_DN35532_c2_g2_i13 | brown | -0.776789 |
| TRINITY_DN37672_c0_g1_i1 | brown | -0.776979 |
| TRINITY_DN23285_c0_g1_i1 | brown | -0.778281 |
| TRINITY_DN39321_c0_g1_i5 | brown | -0.780819 |
| TRINITY_DN25523_c0_g2_i1 | brown | -0.782484 |
| TRINITY_DN33912_c0_g2_i1 | brown | -0.78311 |
| TRINITY_DN21750_c1_g1_i1 | brown | -0.783354 |
| TRINITY_DN35844_c0_g2_i1 | brown | -0.784256 |
| TRINITY_DN39218_c1_g7_i1 | brown | -0.784866 |
| TRINITY_DN33004_c1_g18_i2 | brown | -0.785105 |
| TRINITY_DN19200_c0_g1_i1 | brown | -0.786516 |
| TRINITY_DN38235_c0_g2_i1 | brown | -0.788214 |
| TRINITY_DN37132_c0_g1_i1 | brown | -0.789692 |
| TRINITY_DN33789_c0_g11_i1 | brown | -0.790183 |
| TRINITY_DN20574_c0_g1_i1 | brown | -0.790309 |
| TRINITY_DN24461_c0_g4_i1 | brown | -0.792941 |
| TRINITY_DN36781_c0_g4_i1 | brown | -0.79304 |
| TRINITY_DN32716_c0_g1_i7 | brown | -0.793328 |
| TRINITY_DN22403_c0_g2_i1 | brown | -0.793946 |
| TRINITY_DN29741_c0_g1_i3 | brown | -0.796407 |
| TRINITY_DN36284_c2_g4_i3 | brown | -0.799999 |
| TRINITY_DN29012_c0_g1_i1 | brown | -0.802791 |
| TRINITY_DN38776_c1_g2_i5 | brown | -0.804457 |
| TRINITY_DN38849_c0_g2_i8 | brown | -0.805525 |
| TRINITY_DN38492_c1_g5_i3 | brown | -0.805787 |
| TRINITY_DN37679_c0_g6_i2 | brown | -0.806095 |
| TRINITY_DN19019_c0_g1_i1 | brown | -0.80659 |
| TRINITY_DN37692_c0_g1_i2 | brown | -0.807163 |
| TRINITY_DN20249_c0_g2_i1 | brown | -0.807252 |
| TRINITY_DN28135_c0_g1_i1 | brown | -0.807944 |
| TRINITY_DN27034_c0_g1_i1 | brown | -0.809703 |
| TRINITY_DN36570_c1_g2_i1 | brown | -0.810637 |
| TRINITY_DN21345_c0_g1_i1 | brown | -0.811624 |
| TRINITY_DN37329_c1_g5_i1 | brown | -0.812643 |
| TRINITY_DN34627_c0_g1_i1 | brown | -0.814286 |
| TRINITY_DN30679_c0_g2_i1 | brown | -0.814679 |
| TRINITY_DN12228_c0_g1_i1 | brown | -0.815287 |
| TRINITY_DN38027_c0_g2_i1 | brown | -0.815629 |
| TRINITY_DN37344_c0_g2_i3 | brown | -0.816732 |
| TRINITY_DN30900_c0_g1_i1 | brown | -0.81786 |
| TRINITY_DN38135_c2_g7_i3 | brown | -0.818529 |
| TRINITY_DN39294_c6_g8_i1 | brown | -0.819042 |
| TRINITY_DN13729_c0_g1_i1 | brown | -0.819256 |
| TRINITY_DN29742_c0_g4_i1 | brown | -0.819292 |
| TRINITY_DN39276_c2_g7_i1 | brown | -0.819705 |
| TRINITY_DN33848_c0_g2_i2 | brown | -0.820199 |
| TRINITY_DN32442_c0_g2_i1 | brown | -0.822199 |
| TRINITY_DN38910_c1_g1_i2 | brown | -0.822327 |
| TRINITY_DN35805_c0_g1_i4 | brown | -0.822857 |
| TRINITY_DN39064_c1_g1_i1 | brown | -0.823985 |
| TRINITY_DN23166_c0_g1_i1 | brown | -0.824503 |
| TRINITY_DN36038_c1_g1_i1 | brown | -0.824543 |
| TRINITY_DN36941_c0_g1_i1 | brown | -0.825362 |
| TRINITY_DN36082_c0_g7_i1 | brown | -0.825409 |
| TRINITY_DN22808_c0_g1_i2 | brown | -0.827229 |
| TRINITY_DN29581_c0_g1_i7 | brown | -0.827246 |
| TRINITY_DN24345_c0_g2_i1 | brown | -0.82736 |
| TRINITY_DN27548_c0_g1_i1 | brown | -0.828728 |
| TRINITY_DN38678_c2_g3_i1 | brown | -0.829443 |
| TRINITY_DN32474_c0_g1_i1 | brown | -0.830136 |
| TRINITY_DN32196_c1_g2_i3 | brown | -0.831256 |
| TRINITY_DN36228_c1_g3_i1 | brown | -0.833055 |
| TRINITY_DN31603_c0_g2_i1 | brown | -0.835343 |
| TRINITY_DN33363_c0_g3_i1 | brown | -0.836153 |
| TRINITY_DN26022_c0_g1_i1 | brown | -0.836305 |
| TRINITY_DN16368_c0_g1_i1 | brown | -0.836858 |
| TRINITY_DN25374_c0_g3_i1 | brown | -0.838126 |
| TRINITY_DN39314_c5_g5_i2 | brown | -0.838688 |
| TRINITY_DN38776_c1_g3_i5 | brown | -0.839137 |
| TRINITY_DN37989_c0_g1_i3 | brown | -0.842214 |
| TRINITY_DN36591_c1_g3_i17 | brown | -0.842298 |
| TRINITY_DN38286_c0_g3_i1 | brown | -0.842318 |
| TRINITY_DN31245_c1_g1_i1 | brown | -0.842672 |
| TRINITY_DN32881_c0_g1_i2 | brown | -0.843106 |
| TRINITY_DN31427_c0_g1_i3 | brown | -0.843498 |
| TRINITY_DN33333_c1_g1_i10 | brown | -0.84365 |
| TRINITY_DN10308_c0_g2_i1 | brown | -0.844585 |
| TRINITY_DN36795_c0_g2_i1 | brown | -0.844803 |
| TRINITY_DN39255_c2_g2_i1 | brown | -0.845001 |
| TRINITY_DN56796_c0_g1_i1 | brown | -0.845031 |
| TRINITY_DN9081_c0_g1_i1 | brown | -0.845051 |
| TRINITY_DN4521_c0_g2_i1 | brown | -0.845551 |
| TRINITY_DN33840_c0_g2_i1 | brown | -0.847379 |
| TRINITY_DN32924_c1_g4_i4 | brown | -0.847739 |
| TRINITY_DN35753_c2_g9_i5 | brown | -0.847971 |
| TRINITY_DN20696_c0_g1_i2 | brown | -0.848294 |
| TRINITY_DN38574_c0_g1_i6 | brown | -0.850044 |
| TRINITY_DN29742_c0_g3_i3 | brown | -0.850249 |
| TRINITY_DN37580_c0_g2_i8 | brown | -0.850251 |
| TRINITY_DN38301_c2_g4_i1 | brown | -0.850281 |
| TRINITY_DN31896_c1_g3_i1 | brown | -0.850322 |
| TRINITY_DN37799_c1_g2_i1 | brown | -0.850861 |
| TRINITY_DN30802_c0_g8_i1 | brown | -0.852439 |
| TRINITY_DN27126_c0_g1_i2 | brown | -0.852882 |
| TRINITY_DN25647_c0_g2_i1 | brown | -0.852952 |
| TRINITY_DN37173_c0_g1_i5 | brown | -0.853019 |
| TRINITY_DN37736_c0_g4_i1 | brown | -0.853035 |
| TRINITY_DN26427_c0_g1_i1 | brown | -0.853248 |
| TRINITY_DN28255_c0_g1_i8 | brown | -0.85326 |
| TRINITY_DN21997_c0_g1_i1 | brown | -0.853265 |
| TRINITY_DN32511_c0_g2_i6 | brown | -0.853634 |
| TRINITY_DN37689_c0_g1_i1 | brown | -0.853973 |
| TRINITY_DN22727_c0_g1_i2 | brown | -0.854141 |
| TRINITY_DN28239_c0_g1_i4 | brown | -0.854913 |
| TRINITY_DN31311_c0_g3_i1 | brown | -0.855083 |
| TRINITY_DN37348_c0_g2_i10 | brown | -0.855478 |
| TRINITY_DN29667_c4_g7_i1 | brown | -0.856206 |
| TRINITY_DN38285_c1_g4_i1 | brown | -0.856885 |
| TRINITY_DN30419_c0_g3_i1 | brown | -0.857923 |
| TRINITY_DN27466_c0_g1_i1 | brown | -0.858473 |
| TRINITY_DN37567_c3_g2_i9 | brown | -0.859326 |
| TRINITY_DN38561_c0_g1_i1 | brown | -0.85994 |
| TRINITY_DN36163_c1_g4_i11 | brown | -0.8602 |
| TRINITY_DN33710_c2_g1_i6 | brown | -0.860311 |
| TRINITY_DN35004_c0_g3_i6 | brown | -0.860826 |
| TRINITY_DN45142_c0_g1_i1 | brown | -0.861135 |
| TRINITY_DN60007_c0_g1_i1 | brown | -0.862041 |
| TRINITY_DN37799_c1_g7_i1 | brown | -0.862809 |
| TRINITY_DN24430_c0_g1_i1 | brown | -0.863488 |
| TRINITY_DN38955_c0_g2_i5 | brown | -0.86398 |
| TRINITY_DN31048_c0_g1_i4 | brown | -0.864256 |
| TRINITY_DN31647_c0_g2_i1 | brown | -0.864936 |
| TRINITY_DN22344_c0_g3_i1 | brown | -0.865164 |
| TRINITY_DN33283_c3_g1_i2 | brown | -0.865368 |
| TRINITY_DN37294_c0_g2_i8 | brown | -0.866046 |
| TRINITY_DN36178_c0_g1_i2 | brown | -0.866824 |
| TRINITY_DN38446_c1_g2_i2 | brown | -0.86755 |
| TRINITY_DN19550_c0_g1_i1 | brown | -0.867601 |
| TRINITY_DN35185_c0_g5_i1 | brown | -0.867715 |
| TRINITY_DN38619_c0_g2_i1 | brown | -0.868558 |
| TRINITY_DN38752_c0_g2_i1 | brown | -0.86868 |
| TRINITY_DN39327_c7_g18_i1 | brown | -0.868733 |
| TRINITY_DN30802_c0_g13_i1 | brown | -0.869205 |
| TRINITY_DN34155_c1_g3_i4 | brown | -0.869491 |
| TRINITY_DN25256_c0_g1_i1 | brown | -0.87267 |
| TRINITY_DN13487_c0_g1_i1 | brown | -0.872988 |
| TRINITY_DN31342_c0_g1_i16 | brown | -0.875158 |
| TRINITY_DN32494_c2_g15_i3 | brown | -0.875358 |
| TRINITY_DN18852_c0_g1_i1 | brown | -0.876023 |
| TRINITY_DN38103_c1_g2_i1 | brown | -0.876236 |
| TRINITY_DN32031_c1_g1_i1 | brown | -0.87638 |
| TRINITY_DN34260_c0_g2_i11 | brown | -0.87639 |
| TRINITY_DN24470_c0_g3_i1 | brown | -0.876453 |
| TRINITY_DN37843_c1_g3_i19 | brown | -0.876718 |
| TRINITY_DN39341_c9_g11_i1 | brown | -0.877163 |
| TRINITY_DN29825_c0_g1_i7 | brown | -0.877306 |
| TRINITY_DN21367_c0_g1_i1 | brown | -0.877539 |
| TRINITY_DN36148_c1_g1_i5 | brown | -0.877983 |
| TRINITY_DN36074_c0_g1_i1 | brown | -0.878552 |
| TRINITY_DN38888_c2_g16_i1 | brown | -0.878648 |
| TRINITY_DN30853_c1_g1_i2 | brown | -0.878714 |
| TRINITY_DN33371_c2_g1_i1 | brown | -0.878737 |
| TRINITY_DN23053_c0_g1_i1 | brown | -0.878984 |
| TRINITY_DN38556_c1_g2_i3 | brown | -0.87905 |
| TRINITY_DN27006_c0_g1_i1 | brown | -0.879917 |
| TRINITY_DN34669_c0_g1_i5 | brown | -0.881552 |
| TRINITY_DN30549_c0_g1_i3 | brown | -0.881588 |
| TRINITY_DN32975_c0_g1_i4 | brown | -0.882258 |
| TRINITY_DN36093_c0_g1_i6 | brown | -0.882458 |
| TRINITY_DN39159_c0_g3_i1 | brown | -0.883013 |
| TRINITY_DN39141_c0_g1_i5 | brown | -0.883316 |
| TRINITY_DN30644_c0_g2_i1 | brown | -0.883359 |
| TRINITY_DN18627_c0_g1_i1 | brown | -0.883751 |
| TRINITY_DN35841_c0_g1_i1 | brown | -0.884925 |
| TRINITY_DN27488_c0_g1_i1 | brown | -0.885458 |
| TRINITY_DN38279_c0_g5_i1 | brown | -0.886727 |
| TRINITY_DN35379_c0_g5_i1 | brown | -0.88682 |
| TRINITY_DN25256_c0_g3_i1 | brown | -0.88846 |
| TRINITY_DN20785_c0_g1_i1 | brown | -0.888723 |
| TRINITY_DN34738_c0_g1_i5 | brown | -0.889015 |
| TRINITY_DN38235_c0_g6_i1 | brown | -0.889269 |
| TRINITY_DN35428_c0_g7_i1 | brown | -0.890852 |
| TRINITY_DN32963_c0_g4_i1 | brown | -0.892455 |
| TRINITY_DN33288_c0_g1_i1 | brown | -0.892978 |
| TRINITY_DN28668_c0_g1_i2 | brown | -0.893321 |
| TRINITY_DN19420_c0_g1_i2 | brown | -0.893477 |
| TRINITY_DN39343_c19_g8_i2 | brown | -0.894174 |
| TRINITY_DN34050_c0_g2_i6 | brown | -0.894299 |
| TRINITY_DN30329_c1_g6_i2 | brown | -0.894327 |
| TRINITY_DN36395_c4_g2_i2 | brown | -0.894757 |
| TRINITY_DN29320_c0_g3_i1 | brown | -0.895316 |
| TRINITY_DN32021_c0_g2_i2 | brown | -0.896462 |
| TRINITY_DN27582_c0_g1_i5 | brown | -0.896636 |
| TRINITY_DN1978_c0_g1_i1 | brown | -0.897667 |
| TRINITY_DN38439_c1_g4_i6 | brown | -0.897669 |
| TRINITY_DN38689_c1_g6_i1 | brown | -0.897811 |
| TRINITY_DN23126_c0_g1_i1 | brown | -0.898207 |
| TRINITY_DN31177_c0_g1_i1 | brown | -0.898298 |
| TRINITY_DN35086_c1_g3_i3 | brown | -0.898385 |
| TRINITY_DN18595_c0_g1_i1 | brown | -0.899131 |
| TRINITY_DN36087_c0_g4_i8 | brown | -0.89918 |
| TRINITY_DN37306_c0_g5_i1 | brown | -0.899223 |
| TRINITY_DN37862_c2_g2_i5 | brown | -0.899363 |
| TRINITY_DN33233_c0_g2_i1 | brown | -0.899728 |
| TRINITY_DN30644_c0_g1_i1 | brown | -0.900201 |
| TRINITY_DN25206_c0_g2_i3 | brown | -0.90118 |
| TRINITY_DN28599_c0_g2_i1 | brown | -0.901865 |
| TRINITY_DN37457_c1_g7_i8 | brown | -0.902908 |
| TRINITY_DN37843_c1_g4_i1 | brown | -0.903184 |
| TRINITY_DN28272_c0_g1_i1 | brown | -0.903259 |
| TRINITY_DN34996_c0_g2_i2 | brown | -0.904133 |
| TRINITY_DN39311_c5_g7_i1 | brown | -0.90416 |
| TRINITY_DN48408_c0_g1_i1 | brown | -0.904584 |
| TRINITY_DN34139_c0_g1_i1 | brown | -0.905591 |
| TRINITY_DN39331_c7_g3_i1 | brown | -0.906022 |
| TRINITY_DN29959_c0_g2_i2 | brown | -0.906314 |
| TRINITY_DN39110_c0_g1_i14 | brown | -0.906386 |
| TRINITY_DN35147_c0_g3_i1 | brown | -0.906492 |
| TRINITY_DN38286_c0_g1_i1 | brown | -0.907556 |
| TRINITY_DN35213_c1_g2_i1 | brown | -0.907703 |
| TRINITY_DN44819_c0_g1_i1 | brown | -0.908391 |
| TRINITY_DN24625_c0_g4_i1 | brown | -0.908521 |
| TRINITY_DN36205_c0_g2_i12 | brown | -0.910001 |
| TRINITY_DN19160_c0_g1_i1 | brown | -0.91012 |
| TRINITY_DN26251_c0_g1_i2 | brown | -0.910438 |
| TRINITY_DN17128_c0_g1_i1 | brown | -0.91057 |
| TRINITY_DN36321_c1_g4_i5 | brown | -0.910614 |
| TRINITY_DN36064_c0_g2_i3 | brown | -0.911024 |
| TRINITY_DN38760_c1_g2_i9 | brown | -0.91129 |
| TRINITY_DN14199_c0_g2_i1 | brown | -0.911344 |
| TRINITY_DN29320_c0_g2_i1 | brown | -0.911931 |
| TRINITY_DN29943_c0_g1_i7 | brown | -0.912934 |
| TRINITY_DN25891_c0_g1_i1 | brown | -0.914172 |
| TRINITY_DN24763_c0_g1_i2 | brown | -0.914471 |
| TRINITY_DN25519_c0_g1_i1 | brown | -0.914489 |
| TRINITY_DN25858_c1_g1_i1 | brown | -0.915701 |
| TRINITY_DN38243_c0_g2_i5 | brown | -0.915985 |
| TRINITY_DN38907_c0_g2_i1 | brown | -0.916045 |
| TRINITY_DN19049_c0_g1_i4 | brown | -0.916163 |
| TRINITY_DN28463_c0_g1_i10 | brown | -0.916427 |
| TRINITY_DN38409_c0_g4_i1 | brown | -0.917166 |
| TRINITY_DN37068_c2_g16_i1 | brown | -0.917653 |
| TRINITY_DN30268_c2_g5_i1 | brown | -0.917988 |
| TRINITY_DN31809_c0_g1_i3 | brown | -0.91845 |
| TRINITY_DN38298_c0_g9_i1 | brown | -0.918809 |
| TRINITY_DN38845_c2_g6_i1 | brown | -0.91953 |
| TRINITY_DN29186_c0_g2_i2 | brown | -0.920049 |
| TRINITY_DN31147_c0_g5_i1 | brown | -0.920603 |
| TRINITY_DN38689_c1_g4_i1 | brown | -0.920603 |
| TRINITY_DN30661_c0_g1_i2 | brown | -0.922692 |
| TRINITY_DN28772_c0_g1_i10 | brown | -0.923001 |
| TRINITY_DN36807_c0_g1_i1 | brown | -0.923519 |
| TRINITY_DN26883_c0_g1_i1 | brown | -0.923705 |
| TRINITY_DN27226_c0_g2_i3 | brown | -0.925077 |
| TRINITY_DN32924_c1_g5_i2 | brown | -0.925473 |
| TRINITY_DN37553_c2_g14_i2 | brown | -0.926947 |
| TRINITY_DN22379_c0_g1_i1 | brown | -0.927614 |
| TRINITY_DN33739_c0_g1_i1 | brown | -0.927929 |
| TRINITY_DN31933_c0_g2_i1 | brown | -0.928228 |
| TRINITY_DN39339_c7_g6_i2 | brown | -0.928417 |
| TRINITY_DN24963_c0_g1_i3 | brown | -0.928521 |
| TRINITY_DN31847_c0_g2_i1 | brown | -0.928938 |
| TRINITY_DN19023_c0_g1_i1 | brown | -0.929096 |
| TRINITY_DN34050_c0_g3_i1 | brown | -0.92998 |
| TRINITY_DN23280_c0_g2_i1 | brown | -0.930305 |
| TRINITY_DN38983_c1_g3_i1 | brown | -0.930329 |
| TRINITY_DN34946_c0_g5_i2 | brown | -0.931012 |
| TRINITY_DN28077_c0_g2_i1 | brown | -0.931018 |
| TRINITY_DN39292_c4_g4_i1 | brown | -0.93106 |
| TRINITY_DN34510_c0_g1_i1 | brown | -0.931178 |
| TRINITY_DN38752_c0_g5_i1 | brown | -0.932247 |
| TRINITY_DN38020_c1_g3_i1 | brown | -0.932451 |
| TRINITY_DN36722_c0_g1_i6 | brown | -0.93439 |
| TRINITY_DN21378_c0_g1_i2 | brown | -0.934726 |
| TRINITY_DN17681_c0_g5_i1 | brown | -0.934815 |
| TRINITY_DN17565_c0_g2_i1 | brown | -0.934917 |
| TRINITY_DN27552_c0_g3_i1 | brown | -0.93603 |
| TRINITY_DN38693_c3_g8_i1 | brown | -0.936593 |
| TRINITY_DN34333_c0_g4_i5 | brown | -0.936858 |
| TRINITY_DN36883_c1_g8_i1 | brown | -0.937217 |
| TRINITY_DN39134_c5_g1_i6 | brown | -0.937363 |
| TRINITY_DN37533_c3_g1_i1 | brown | -0.937408 |
| TRINITY_DN38956_c0_g3_i1 | brown | -0.937821 |
| TRINITY_DN29713_c0_g1_i1 | brown | -0.937999 |
| TRINITY_DN37924_c0_g3_i1 | brown | -0.938853 |
| TRINITY_DN38036_c1_g1_i5 | brown | -0.940186 |
| TRINITY_DN35687_c1_g1_i1 | brown | -0.940779 |
| TRINITY_DN32746_c0_g1_i11 | brown | -0.940804 |
| TRINITY_DN37487_c0_g5_i1 | brown | -0.941183 |
| TRINITY_DN35557_c0_g2_i3 | brown | -0.942535 |
| TRINITY_DN34946_c0_g4_i1 | brown | -0.94304 |
| TRINITY_DN23761_c0_g3_i1 | brown | -0.944034 |
| TRINITY_DN28607_c0_g1_i1 | brown | -0.944329 |
| TRINITY_DN27059_c0_g1_i1 | brown | -0.94446 |
| TRINITY_DN34935_c0_g2_i1 | brown | -0.944916 |
| TRINITY_DN6340_c0_g2_i1 | brown | -0.945356 |
| TRINITY_DN31657_c0_g1_i2 | brown | -0.945419 |
| TRINITY_DN39292_c4_g1_i4 | brown | -0.949401 |
| TRINITY_DN30184_c0_g1_i2 | brown | -0.949643 |
| TRINITY_DN27706_c0_g1_i1 | brown | -0.949933 |
| TRINITY_DN36226_c1_g3_i2 | brown | -0.95029 |
| TRINITY_DN22363_c0_g2_i1 | brown | -0.950315 |
| TRINITY_DN35463_c1_g4_i2 | brown | -0.951233 |
| TRINITY_DN34930_c0_g1_i6 | brown | -0.951951 |
| TRINITY_DN22315_c0_g1_i1 | brown | -0.954502 |
| TRINITY_DN39035_c1_g4_i7 | brown | -0.954882 |
| TRINITY_DN27217_c0_g2_i4 | brown | -0.955039 |
| TRINITY_DN37614_c1_g1_i1 | brown | -0.956731 |
| TRINITY_DN38936_c0_g6_i3 | brown | -0.956889 |
| TRINITY_DN38409_c0_g5_i1 | brown | -0.956891 |
| TRINITY_DN35440_c0_g1_i1 | brown | -0.957596 |
| TRINITY_DN38753_c1_g5_i1 | brown | -0.958724 |
| TRINITY_DN33579_c0_g2_i1 | brown | -0.963858 |
| TRINITY_DN36054_c1_g1_i7 | brown | -0.964438 |
| TRINITY_DN24815_c0_g1_i2 | brown | -0.96514 |
| TRINITY_DN30021_c0_g1_i1 | brown | -0.966874 |
| TRINITY_DN38996_c2_g1_i1 | brown | -0.971641 |
| TRINITY_DN31967_c0_g1_i1 | green | 0.9955311 |
| TRINITY_DN34385_c2_g3_i1 | green | 0.9907007 |
| TRINITY_DN38125_c0_g2_i2 | green | 0.9897933 |
| TRINITY_DN33269_c0_g2_i4 | green | 0.9891996 |
| TRINITY_DN36396_c0_g5_i7 | green | 0.9860229 |
| TRINITY_DN36755_c0_g4_i1 | green | 0.9859165 |
| TRINITY_DN34965_c0_g2_i2 | green | 0.9853865 |
| TRINITY_DN34965_c0_g3_i1 | green | 0.9847082 |
| TRINITY_DN39065_c6_g12_i1 | green | 0.9840145 |
| TRINITY_DN39283_c3_g1_i1 | green | 0.9838688 |
| TRINITY_DN37923_c2_g1_i3 | green | 0.9833189 |
| TRINITY_DN23705_c0_g1_i1 | green | 0.9830664 |
| TRINITY_DN39051_c1_g1_i4 | green | 0.9820055 |
| TRINITY_DN26379_c0_g1_i4 | green | 0.9817415 |
| TRINITY_DN39068_c0_g25_i1 | green | 0.9810908 |
| TRINITY_DN32598_c0_g2_i1 | green | 0.9810037 |
| TRINITY_DN31454_c0_g7_i1 | green | 0.978943 |
| TRINITY_DN39283_c3_g2_i7 | green | 0.9786597 |
| TRINITY_DN38943_c1_g1_i8 | green | 0.9781683 |
| TRINITY_DN23137_c0_g2_i1 | green | 0.9774529 |
| TRINITY_DN35955_c3_g2_i1 | green | 0.9773477 |
| TRINITY_DN24925_c0_g2_i1 | green | 0.9770456 |
| TRINITY_DN20186_c0_g1_i1 | green | 0.9767971 |
| TRINITY_DN35185_c0_g1_i1 | green | 0.9764162 |
| TRINITY_DN38888_c2_g6_i3 | green | 0.9758252 |
| TRINITY_DN18550_c1_g1_i1 | green | 0.9749863 |
| TRINITY_DN31691_c1_g1_i3 | green | 0.9749033 |
| TRINITY_DN28298_c0_g4_i1 | green | 0.9741133 |
| TRINITY_DN36115_c2_g4_i1 | green | 0.9736932 |
| TRINITY_DN25329_c0_g7_i1 | green | 0.9734127 |
| TRINITY_DN35548_c0_g2_i1 | green | 0.9732694 |
| TRINITY_DN34965_c0_g5_i1 | green | 0.973259 |
| TRINITY_DN22072_c0_g1_i1 | green | 0.9731827 |
| TRINITY_DN35056_c2_g5_i1 | green | 0.9729485 |
| TRINITY_DN20099_c0_g2_i1 | green | 0.9727127 |
| TRINITY_DN36647_c0_g3_i5 | green | 0.9724248 |
| TRINITY_DN38846_c0_g1_i1 | green | 0.9718666 |
| TRINITY_DN38101_c0_g4_i1 | green | 0.9713402 |
| TRINITY_DN34182_c0_g2_i2 | green | 0.9710703 |
| TRINITY_DN38792_c1_g5_i1 | green | 0.9706421 |
| TRINITY_DN35438_c0_g1_i2 | green | 0.9706167 |
| TRINITY_DN37717_c1_g6_i2 | green | 0.9704445 |
| TRINITY_DN35220_c1_g1_i1 | green | 0.9704221 |
| TRINITY_DN35530_c1_g2_i1 | green | 0.9699667 |
| TRINITY_DN38846_c2_g4_i1 | green | 0.9698585 |
| TRINITY_DN38513_c0_g2_i3 | green | 0.9697842 |
| TRINITY_DN34748_c0_g1_i1 | green | 0.9696733 |
| TRINITY_DN28341_c0_g2_i2 | green | 0.9693557 |
| TRINITY_DN39254_c0_g2_i6 | green | 0.9691513 |
| TRINITY_DN30684_c0_g1_i2 | green | 0.9687463 |
| TRINITY_DN26785_c0_g1_i1 | green | 0.9686101 |
| TRINITY_DN35249_c0_g3_i1 | green | 0.9683155 |
| TRINITY_DN39312_c3_g4_i1 | green | 0.9681477 |
| TRINITY_DN37408_c0_g3_i1 | green | 0.9680555 |
| TRINITY_DN31163_c0_g1_i4 | green | 0.9671926 |
| TRINITY_DN35249_c0_g4_i2 | green | 0.9669468 |
| TRINITY_DN34519_c0_g9_i1 | green | 0.9667644 |
| TRINITY_DN35278_c1_g2_i1 | green | 0.9666766 |
| TRINITY_DN34482_c0_g4_i1 | green | 0.9666518 |
| TRINITY_DN28165_c0_g1_i1 | green | 0.9662782 |
| TRINITY_DN33935_c0_g5_i2 | green | 0.9662653 |
| TRINITY_DN22333_c0_g4_i1 | green | 0.9658189 |
| TRINITY_DN27808_c0_g1_i4 | green | 0.9654459 |
| TRINITY_DN25494_c0_g1_i3 | green | 0.9653585 |
| TRINITY_DN25368_c0_g1_i1 | green | 0.9652925 |
| TRINITY_DN31691_c1_g3_i1 | green | 0.9652917 |
| TRINITY_DN27986_c0_g1_i1 | green | 0.9652722 |
| TRINITY_DN35898_c1_g1_i1 | green | 0.9651782 |
| TRINITY_DN39283_c3_g8_i1 | green | 0.9651667 |
| TRINITY_DN39137_c3_g2_i3 | green | 0.9650419 |
| TRINITY_DN36345_c0_g4_i7 | green | 0.9647581 |
| TRINITY_DN34965_c0_g4_i4 | green | 0.964301 |
| TRINITY_DN22252_c0_g1_i1 | green | 0.9642346 |
| TRINITY_DN23408_c0_g1_i1 | green | 0.9642312 |
| TRINITY_DN22471_c0_g1_i1 | green | 0.9635308 |
| TRINITY_DN38678_c2_g2_i4 | green | 0.9634667 |
| TRINITY_DN39068_c0_g2_i1 | green | 0.9632822 |
| TRINITY_DN38540_c1_g2_i1 | green | 0.9632302 |
| TRINITY_DN21213_c0_g2_i1 | green | 0.9630691 |
| TRINITY_DN38979_c1_g2_i4 | green | 0.9629442 |
| TRINITY_DN36778_c0_g1_i3 | green | 0.9627979 |
| TRINITY_DN32461_c0_g1_i4 | green | 0.9627345 |
| TRINITY_DN25403_c0_g1_i1 | green | 0.962677 |
| TRINITY_DN39142_c0_g1_i1 | green | 0.9624121 |
| TRINITY_DN34635_c0_g1_i10 | green | 0.9623982 |
| TRINITY_DN28123_c0_g1_i2 | green | 0.9623101 |
| TRINITY_DN37595_c1_g2_i1 | green | 0.9622208 |
| TRINITY_DN37567_c4_g1_i1 | green | 0.9622006 |
| TRINITY_DN10835_c0_g2_i1 | green | 0.9618644 |
| TRINITY_DN33002_c0_g2_i8 | green | 0.9603497 |
| TRINITY_DN28758_c0_g3_i1 | green | 0.9602605 |
| TRINITY_DN4_c0_g1_i1 | green | 0.9602378 |
| TRINITY_DN34965_c0_g1_i1 | green | 0.9601441 |
| TRINITY_DN26106_c0_g1_i1 | green | 0.9600351 |
| TRINITY_DN32387_c0_g1_i3 | green | 0.9598476 |
| TRINITY_DN34124_c1_g5_i1 | green | 0.9595568 |
| TRINITY_DN37386_c1_g12_i1 | green | 0.9588153 |
| TRINITY_DN38755_c1_g2_i1 | green | 0.9583463 |
| TRINITY_DN20043_c0_g1_i1 | green | 0.9583211 |
| TRINITY_DN38301_c2_g3_i1 | green | 0.9579886 |
| TRINITY_DN37594_c1_g5_i1 | green | 0.9577423 |
| TRINITY_DN34043_c1_g2_i1 | green | 0.9577389 |
| TRINITY_DN19409_c0_g1_i1 | green | 0.9570756 |
| TRINITY_DN39220_c6_g7_i1 | green | 0.9569411 |
| TRINITY_DN24403_c0_g3_i1 | green | 0.9546872 |
| TRINITY_DN25329_c0_g6_i1 | green | 0.9543251 |
| TRINITY_DN21606_c0_g1_i1 | green | 0.9542584 |
| TRINITY_DN17512_c0_g1_i1 | green | 0.9540193 |
| TRINITY_DN28778_c1_g1_i2 | green | 0.9539437 |
| TRINITY_DN20075_c0_g1_i1 | green | 0.9538987 |
| TRINITY_DN33821_c0_g1_i1 | green | 0.953788 |
| TRINITY_DN23302_c0_g4_i1 | green | 0.9537692 |
| TRINITY_DN37306_c0_g8_i1 | green | 0.9532706 |
| TRINITY_DN24133_c0_g1_i1 | green | 0.9527247 |
| TRINITY_DN30944_c3_g2_i4 | green | 0.9525823 |
| TRINITY_DN37537_c0_g1_i4 | green | 0.9524644 |
| TRINITY_DN25180_c0_g1_i1 | green | 0.9520108 |
| TRINITY_DN25674_c0_g1_i2 | green | 0.951742 |
| TRINITY_DN29785_c0_g2_i1 | green | 0.9514538 |
| TRINITY_DN58694_c0_g1_i1 | green | 0.9513222 |
| TRINITY_DN32733_c0_g1_i1 | green | 0.9512325 |
| TRINITY_DN37299_c0_g3_i1 | green | 0.9511824 |
| TRINITY_DN24637_c0_g1_i1 | green | 0.9510157 |
| TRINITY_DN36515_c0_g4_i4 | green | 0.9509363 |
| TRINITY_DN38846_c2_g2_i8 | green | 0.9507547 |
| TRINITY_DN27280_c0_g2_i2 | green | 0.9507251 |
| TRINITY_DN33883_c2_g1_i1 | green | 0.950505 |
| TRINITY_DN36028_c2_g4_i1 | green | 0.9502339 |
| TRINITY_DN25643_c0_g4_i1 | green | 0.9498936 |
| TRINITY_DN21572_c0_g1_i1 | green | 0.9495588 |
| TRINITY_DN26196_c1_g7_i1 | green | 0.9494396 |
| TRINITY_DN39125_c0_g14_i1 | green | 0.9493993 |
| TRINITY_DN31800_c0_g1_i2 | green | 0.9493353 |
| TRINITY_DN39314_c5_g9_i3 | green | 0.9492672 |
| TRINITY_DN38176_c1_g13_i1 | green | 0.9490843 |
| TRINITY_DN33289_c0_g1_i5 | green | 0.9486566 |
| TRINITY_DN13609_c0_g2_i1 | green | 0.9484507 |
| TRINITY_DN36071_c0_g1_i5 | green | 0.9482519 |
| TRINITY_DN27232_c0_g2_i2 | green | 0.9481869 |
| TRINITY_DN9847_c0_g1_i1 | green | 0.9481636 |
| TRINITY_DN30271_c0_g1_i1 | green | 0.9479428 |
| TRINITY_DN39438_c0_g1_i1 | green | 0.9479316 |
| TRINITY_DN38442_c7_g12_i1 | green | 0.947782 |
| TRINITY_DN28014_c0_g1_i1 | green | 0.9476079 |
| TRINITY_DN19848_c0_g2_i1 | green | 0.947436 |
| TRINITY_DN37905_c1_g2_i5 | green | 0.9473875 |
| TRINITY_DN26596_c0_g1_i3 | green | 0.9470025 |
| TRINITY_DN38888_c2_g12_i1 | green | 0.9467382 |
| TRINITY_DN38135_c1_g1_i5 | green | 0.9466769 |
| TRINITY_DN39069_c3_g27_i1 | green | 0.9466461 |
| TRINITY_DN51595_c0_g1_i1 | green | 0.9463588 |
| TRINITY_DN25868_c0_g1_i2 | green | 0.9462063 |
| TRINITY_DN13107_c0_g1_i1 | green | 0.9457702 |
| TRINITY_DN27961_c0_g2_i3 | green | 0.9451225 |
| TRINITY_DN37525_c2_g4_i1 | green | 0.9450799 |
| TRINITY_DN38549_c0_g2_i1 | green | 0.9444627 |
| TRINITY_DN36377_c0_g3_i6 | green | 0.9443875 |
| TRINITY_DN24872_c0_g2_i1 | green | 0.9442455 |
| TRINITY_DN12831_c0_g2_i1 | green | 0.9438295 |
| TRINITY_DN24096_c0_g2_i1 | green | 0.9437065 |
| TRINITY_DN36932_c0_g8_i1 | green | 0.9436061 |
| TRINITY_DN37306_c0_g10_i1 | green | 0.9434498 |
| TRINITY_DN36577_c1_g5_i1 | green | 0.9434211 |
| TRINITY_DN39069_c3_g7_i3 | green | 0.943054 |
| TRINITY_DN27419_c0_g1_i1 | green | 0.9430485 |
| TRINITY_DN33862_c0_g2_i2 | green | 0.9430333 |
| TRINITY_DN39062_c1_g3_i1 | green | 0.9428066 |
| TRINITY_DN29947_c0_g3_i1 | green | 0.9426949 |
| TRINITY_DN25806_c0_g2_i1 | green | 0.942252 |
| TRINITY_DN25150_c0_g1_i1 | green | 0.9420812 |
| TRINITY_DN38211_c1_g2_i2 | green | 0.9417171 |
| TRINITY_DN37842_c1_g9_i1 | green | 0.9416689 |
| TRINITY_DN24020_c1_g1_i1 | green | 0.9416609 |
| TRINITY_DN20503_c0_g1_i1 | green | 0.9416122 |
| TRINITY_DN26090_c0_g1_i1 | green | 0.941092 |
| TRINITY_DN35333_c0_g4_i3 | green | 0.940629 |
| TRINITY_DN23816_c0_g2_i2 | green | 0.9405582 |
| TRINITY_DN32688_c0_g2_i1 | green | 0.9403963 |
| TRINITY_DN70881_c0_g1_i1 | green | 0.9403617 |
| TRINITY_DN35766_c0_g1_i3 | green | 0.9403433 |
| TRINITY_DN31270_c0_g1_i1 | green | 0.9401722 |
| TRINITY_DN35240_c0_g1_i9 | green | 0.9400795 |
| TRINITY_DN34739_c0_g2_i3 | green | 0.9398945 |
| TRINITY_DN17565_c0_g3_i1 | green | 0.939671 |
| TRINITY_DN35250_c0_g3_i4 | green | 0.9394196 |
| TRINITY_DN38719_c0_g2_i1 | green | 0.9393367 |
| TRINITY_DN20274_c0_g1_i1 | green | 0.9391681 |
| TRINITY_DN32094_c0_g1_i2 | green | 0.9391143 |
| TRINITY_DN16190_c0_g1_i1 | green | 0.9387716 |
| TRINITY_DN38231_c1_g9_i1 | green | 0.9383455 |
| TRINITY_DN36140_c0_g1_i1 | green | 0.9382854 |
| TRINITY_DN39200_c1_g1_i1 | green | 0.9381584 |
| TRINITY_DN39269_c5_g1_i1 | green | 0.9380255 |
| TRINITY_DN35526_c2_g11_i1 | green | 0.9376337 |
| TRINITY_DN13358_c0_g2_i1 | green | 0.9375825 |
| TRINITY_DN32900_c0_g1_i14 | green | 0.9363609 |
| TRINITY_DN38098_c0_g2_i1 | green | 0.9358253 |
| TRINITY_DN39335_c10_g15_i1 | green | 0.9350714 |
| TRINITY_DN63109_c0_g1_i1 | green | 0.9349907 |
| TRINITY_DN23868_c0_g1_i1 | green | 0.934834 |
| TRINITY_DN30411_c2_g1_i1 | green | 0.93481 |
| TRINITY_DN29235_c0_g1_i3 | green | 0.9345239 |
| TRINITY_DN36560_c0_g1_i13 | green | 0.9344899 |
| TRINITY_DN30781_c0_g1_i6 | green | 0.9336716 |
| TRINITY_DN19344_c0_g1_i4 | green | 0.9335948 |
| TRINITY_DN5390_c0_g1_i1 | green | 0.933452 |
| TRINITY_DN20351_c0_g2_i1 | green | 0.9333061 |
| TRINITY_DN38791_c3_g4_i1 | green | 0.9330987 |
| TRINITY_DN38866_c0_g6_i1 | green | 0.9329851 |
| TRINITY_DN36534_c2_g5_i4 | green | 0.9323239 |
| TRINITY_DN36885_c0_g1_i1 | green | 0.9320344 |
| TRINITY_DN16235_c0_g1_i1 | green | 0.9314786 |
| TRINITY_DN27963_c1_g2_i1 | green | 0.9314347 |
| TRINITY_DN39062_c3_g5_i3 | green | 0.9313777 |
| TRINITY_DN33982_c3_g6_i1 | green | 0.9313486 |
| TRINITY_DN67414_c0_g1_i1 | green | 0.9312976 |
| TRINITY_DN36377_c0_g2_i2 | green | 0.9311762 |
| TRINITY_DN36039_c1_g10_i1 | green | 0.9311406 |
| TRINITY_DN57276_c0_g1_i1 | green | 0.930947 |
| TRINITY_DN38551_c1_g5_i1 | green | 0.9308223 |
| TRINITY_DN35192_c0_g1_i4 | green | 0.9306104 |
| TRINITY_DN34568_c0_g1_i1 | green | 0.9305869 |
| TRINITY_DN34513_c0_g12_i2 | green | 0.9305123 |
| TRINITY_DN36835_c1_g2_i1 | green | 0.9301802 |
| TRINITY_DN32484_c0_g1_i1 | green | 0.9301448 |
| TRINITY_DN29140_c0_g2_i1 | green | 0.9300879 |
| TRINITY_DN24387_c0_g3_i1 | green | 0.9299908 |
| TRINITY_DN32935_c0_g1_i5 | green | 0.9297746 |
| TRINITY_DN39271_c6_g15_i1 | green | 0.9295634 |
| TRINITY_DN20832_c0_g2_i1 | green | 0.928831 |
| TRINITY_DN30671_c0_g2_i5 | green | 0.9286864 |
| TRINITY_DN34827_c1_g1_i1 | green | 0.9285849 |
| TRINITY_DN36759_c0_g2_i2 | green | 0.9285756 |
| TRINITY_DN33817_c1_g3_i1 | green | 0.9277127 |
| TRINITY_DN34427_c0_g1_i5 | green | 0.927487 |
| TRINITY_DN23849_c0_g1_i2 | green | 0.9274556 |
| TRINITY_DN27922_c0_g1_i1 | green | 0.9274104 |
| TRINITY_DN35961_c1_g2_i1 | green | 0.9273251 |
| TRINITY_DN30571_c0_g1_i1 | green | 0.9272954 |
| TRINITY_DN24189_c0_g3_i1 | green | 0.926985 |
| TRINITY_DN33935_c0_g3_i1 | green | 0.9268995 |
| TRINITY_DN26881_c0_g6_i1 | green | 0.9268946 |
| TRINITY_DN13842_c0_g3_i1 | green | 0.9268247 |
| TRINITY_DN36514_c0_g3_i1 | green | 0.9267607 |
| TRINITY_DN32611_c0_g1_i1 | green | 0.9266158 |
| TRINITY_DN21825_c0_g1_i1 | green | 0.9264334 |
| TRINITY_DN29684_c4_g5_i3 | green | 0.9257882 |
| TRINITY_DN36617_c0_g5_i1 | green | 0.9256242 |
| TRINITY_DN25990_c0_g1_i1 | green | 0.9255827 |
| TRINITY_DN31039_c0_g2_i2 | green | 0.9255611 |
| TRINITY_DN34896_c0_g1_i1 | green | 0.9254907 |
| TRINITY_DN29096_c0_g1_i2 | green | 0.9252451 |
| TRINITY_DN27157_c0_g1_i2 | green | 0.9252288 |
| TRINITY_DN33757_c0_g1_i4 | green | 0.9251298 |
| TRINITY_DN22582_c0_g2_i1 | green | 0.9251216 |
| TRINITY_DN39306_c0_g2_i1 | green | 0.9251021 |
| TRINITY_DN33749_c0_g2_i1 | green | 0.9250483 |
| TRINITY_DN38680_c0_g1_i1 | green | 0.9248818 |
| TRINITY_DN23978_c0_g2_i1 | green | 0.9245044 |
| TRINITY_DN23133_c0_g4_i1 | green | 0.9243151 |
| TRINITY_DN35911_c0_g1_i7 | green | 0.9238912 |
| TRINITY_DN24496_c0_g1_i2 | green | 0.9237739 |
| TRINITY_DN28908_c0_g5_i1 | green | 0.9233373 |
| TRINITY_DN32094_c0_g2_i1 | green | 0.922924 |
| TRINITY_DN29858_c0_g1_i1 | green | 0.9226503 |
| TRINITY_DN29552_c0_g1_i1 | green | 0.9226251 |
| TRINITY_DN16637_c0_g1_i1 | green | 0.9226208 |
| TRINITY_DN38986_c0_g1_i5 | green | 0.9226025 |
| TRINITY_DN21175_c0_g1_i1 | green | 0.9225503 |
| TRINITY_DN38364_c0_g4_i1 | green | 0.9222363 |
| TRINITY_DN10268_c0_g1_i1 | green | 0.9221884 |
| TRINITY_DN38301_c2_g2_i1 | green | 0.9221075 |
| TRINITY_DN38339_c1_g4_i1 | green | 0.9215572 |
| TRINITY_DN53718_c0_g1_i1 | green | 0.9215277 |
| TRINITY_DN36082_c0_g11_i1 | green | 0.9214504 |
| TRINITY_DN37333_c1_g1_i2 | green | 0.9214403 |
| TRINITY_DN38555_c1_g3_i1 | green | 0.9214149 |
| TRINITY_DN83_c0_g1_i1 | green | 0.9213645 |
| TRINITY_DN38888_c2_g18_i1 | green | 0.9210205 |
| TRINITY_DN31069_c0_g3_i1 | green | 0.9208781 |
| TRINITY_DN39321_c1_g1_i4 | green | 0.9208756 |
| TRINITY_DN24176_c0_g1_i1 | green | 0.9207106 |
| TRINITY_DN27062_c0_g2_i1 | green | 0.9207106 |
| TRINITY_DN37005_c0_g3_i1 | green | 0.9202601 |
| TRINITY_DN37591_c0_g1_i3 | green | 0.9202556 |
| TRINITY_DN39275_c3_g2_i1 | green | 0.9202113 |
| TRINITY_DN37791_c2_g9_i1 | green | 0.9202093 |
| TRINITY_DN27455_c0_g1_i1 | green | 0.9201287 |
| TRINITY_DN34466_c0_g3_i3 | green | 0.9197943 |
| TRINITY_DN39200_c1_g2_i4 | green | 0.9196615 |
| TRINITY_DN26934_c0_g1_i1 | green | 0.9195451 |
| TRINITY_DN33878_c1_g1_i1 | green | 0.9195283 |
| TRINITY_DN33762_c0_g8_i1 | green | 0.9194161 |
| TRINITY_DN27768_c0_g1_i1 | green | 0.9190751 |
| TRINITY_DN38853_c0_g1_i8 | green | 0.9189287 |
| TRINITY_DN27154_c1_g1_i1 | green | 0.9188739 |
| TRINITY_DN17818_c0_g2_i1 | green | 0.9181565 |
| TRINITY_DN24210_c0_g1_i1 | green | 0.9179644 |
| TRINITY_DN39228_c6_g1_i1 | green | 0.9179392 |
| TRINITY_DN34242_c0_g4_i1 | green | 0.917755 |
| TRINITY_DN23066_c0_g1_i2 | green | 0.9177219 |
| TRINITY_DN25999_c0_g4_i1 | green | 0.9170954 |
| TRINITY_DN30753_c0_g1_i1 | green | 0.9166938 |
| TRINITY_DN39339_c7_g9_i1 | green | 0.9162554 |
| TRINITY_DN27552_c0_g5_i1 | green | 0.9160719 |
| TRINITY_DN25991_c0_g1_i1 | green | 0.9158098 |
| TRINITY_DN26426_c0_g1_i1 | green | 0.9153876 |
| TRINITY_DN39041_c0_g1_i1 | green | 0.915239 |
| TRINITY_DN36304_c0_g5_i8 | green | 0.9151334 |
| TRINITY_DN29269_c0_g1_i4 | green | 0.9145644 |
| TRINITY_DN35339_c2_g1_i6 | green | 0.9144583 |
| TRINITY_DN22771_c1_g4_i1 | green | 0.9142411 |
| TRINITY_DN35024_c1_g2_i3 | green | 0.9140539 |
| TRINITY_DN33802_c0_g1_i15 | green | 0.9138382 |
| TRINITY_DN14947_c0_g1_i1 | green | 0.9135481 |
| TRINITY_DN24324_c0_g1_i1 | green | 0.9133596 |
| TRINITY_DN14529_c0_g1_i2 | green | 0.9132436 |
| TRINITY_DN32756_c1_g2_i1 | green | 0.9131519 |
| TRINITY_DN29452_c0_g1_i2 | green | 0.9129637 |
| TRINITY_DN22247_c0_g1_i2 | green | 0.912833 |
| TRINITY_DN26999_c0_g1_i1 | green | 0.9127399 |
| TRINITY_DN6024_c0_g1_i1 | green | 0.9127261 |
| TRINITY_DN33792_c0_g3_i6 | green | 0.9124251 |
| TRINITY_DN29601_c0_g1_i3 | green | 0.9120491 |
| TRINITY_DN34088_c0_g1_i3 | green | 0.9120045 |
| TRINITY_DN39190_c2_g4_i1 | green | 0.9119797 |
| TRINITY_DN59746_c0_g2_i1 | green | 0.9117071 |
| TRINITY_DN22705_c0_g2_i1 | green | 0.9114308 |
| TRINITY_DN31111_c1_g1_i1 | green | 0.9113863 |
| TRINITY_DN25625_c0_g2_i1 | green | 0.91117 |
| TRINITY_DN36581_c3_g18_i1 | green | 0.9108656 |
| TRINITY_DN39340_c19_g7_i1 | green | 0.9108292 |
| TRINITY_DN35628_c0_g1_i1 | green | 0.9105523 |
| TRINITY_DN34117_c0_g2_i1 | green | 0.9104677 |
| TRINITY_DN33982_c3_g2_i6 | green | 0.9103353 |
| TRINITY_DN27100_c0_g2_i4 | green | 0.9101738 |
| TRINITY_DN37525_c2_g2_i1 | green | 0.9100466 |
| TRINITY_DN19180_c0_g1_i1 | green | 0.9097066 |
| TRINITY_DN26544_c0_g1_i6 | green | 0.9096653 |
| TRINITY_DN35527_c0_g1_i1 | green | 0.9093942 |
| TRINITY_DN37591_c0_g3_i1 | green | 0.9090956 |
| TRINITY_DN23926_c0_g1_i1 | green | 0.9090374 |
| TRINITY_DN18022_c0_g1_i1 | green | 0.9088624 |
| TRINITY_DN38395_c2_g2_i1 | green | 0.9088595 |
| TRINITY_DN34122_c4_g2_i1 | green | 0.9088374 |
| TRINITY_DN27428_c0_g2_i1 | green | 0.908723 |
| TRINITY_DN36704_c0_g2_i1 | green | 0.9085691 |
| TRINITY_DN27611_c0_g1_i2 | green | 0.9082387 |
| TRINITY_DN29036_c0_g1_i5 | green | 0.9081706 |
| TRINITY_DN38693_c3_g3_i1 | green | 0.9081103 |
| TRINITY_DN36968_c0_g1_i1 | green | 0.9080039 |
| TRINITY_DN35789_c0_g3_i4 | green | 0.9079222 |
| TRINITY_DN28888_c0_g1_i2 | green | 0.9079185 |
| TRINITY_DN17128_c0_g3_i1 | green | 0.9078374 |
| TRINITY_DN22425_c0_g1_i1 | green | 0.9077118 |
| TRINITY_DN20234_c0_g3_i1 | green | 0.907615 |
| TRINITY_DN38991_c0_g4_i1 | green | 0.9071631 |
| TRINITY_DN34281_c0_g1_i1 | green | 0.9069947 |
| TRINITY_DN39329_c14_g4_i2 | green | 0.9058794 |
| TRINITY_DN23630_c0_g1_i1 | green | 0.9058212 |
| TRINITY_DN30722_c0_g10_i1 | green | 0.9055584 |
| TRINITY_DN32582_c1_g2_i1 | green | 0.9050994 |
| TRINITY_DN33639_c0_g2_i1 | green | 0.9048318 |
| TRINITY_DN33626_c0_g1_i17 | green | 0.9047032 |
| TRINITY_DN35387_c2_g3_i2 | green | 0.9046902 |
| TRINITY_DN33336_c1_g1_i3 | green | 0.9046828 |
| TRINITY_DN31950_c0_g6_i1 | green | 0.9046636 |
| TRINITY_DN27829_c0_g1_i1 | green | 0.9044996 |
| TRINITY_DN30672_c0_g2_i1 | green | 0.9039864 |
| TRINITY_DN31890_c0_g2_i1 | green | 0.9039351 |
| TRINITY_DN29552_c0_g3_i1 | green | 0.9038807 |
| TRINITY_DN32575_c0_g2_i2 | green | 0.9031344 |
| TRINITY_DN27957_c0_g1_i2 | green | 0.9030873 |
| TRINITY_DN23885_c0_g1_i1 | green | 0.9027396 |
| TRINITY_DN21076_c0_g1_i1 | green | 0.9025496 |
| TRINITY_DN30627_c0_g1_i1 | green | 0.9020009 |
| TRINITY_DN31359_c0_g2_i1 | green | 0.9015201 |
| TRINITY_DN56845_c0_g1_i1 | green | 0.9012477 |
| TRINITY_DN27018_c0_g3_i2 | green | 0.9012148 |
| TRINITY_DN26685_c0_g1_i1 | green | 0.9011894 |
| TRINITY_DN33339_c0_g1_i3 | green | 0.9009018 |
| TRINITY_DN30055_c1_g1_i4 | green | 0.8995926 |
| TRINITY_DN38364_c0_g1_i1 | green | 0.8992333 |
| TRINITY_DN32667_c0_g2_i1 | green | 0.8991729 |
| TRINITY_DN29684_c4_g3_i5 | green | 0.8981522 |
| TRINITY_DN31116_c0_g1_i2 | green | 0.8980025 |
| TRINITY_DN29751_c0_g1_i1 | green | 0.8978297 |
| TRINITY_DN21765_c0_g3_i2 | green | 0.8976921 |
| TRINITY_DN25570_c0_g5_i1 | green | 0.8972164 |
| TRINITY_DN67980_c0_g1_i1 | green | 0.8968463 |
| TRINITY_DN29618_c0_g1_i8 | green | 0.8966793 |
| TRINITY_DN21305_c0_g2_i1 | green | 0.8964799 |
| TRINITY_DN21967_c0_g1_i1 | green | 0.8964222 |
| TRINITY_DN27440_c0_g1_i1 | green | 0.8964201 |
| TRINITY_DN37799_c1_g8_i1 | green | 0.8962099 |
| TRINITY_DN17236_c0_g1_i2 | green | 0.8961355 |
| TRINITY_DN31147_c0_g2_i1 | green | 0.8959438 |
| TRINITY_DN28256_c0_g2_i1 | green | 0.8956653 |
| TRINITY_DN28879_c0_g1_i3 | green | 0.8956318 |
| TRINITY_DN27010_c0_g1_i1 | green | 0.8956157 |
| TRINITY_DN26858_c0_g2_i1 | green | 0.8954933 |
| TRINITY_DN18529_c0_g1_i1 | green | 0.8949049 |
| TRINITY_DN36778_c0_g3_i1 | green | 0.894781 |
| TRINITY_DN24987_c0_g1_i1 | green | 0.8947539 |
| TRINITY_DN37373_c0_g1_i2 | green | 0.8947147 |
| TRINITY_DN39023_c0_g2_i1 | green | 0.8944958 |
| TRINITY_DN27607_c0_g3_i1 | green | 0.8941783 |
| TRINITY_DN24727_c0_g1_i1 | green | 0.8941174 |
| TRINITY_DN30789_c0_g1_i1 | green | 0.8938832 |
| TRINITY_DN32707_c0_g2_i4 | green | 0.8937816 |
| TRINITY_DN28594_c0_g2_i1 | green | 0.8935513 |
| TRINITY_DN35387_c2_g16_i1 | green | 0.8934551 |
| TRINITY_DN24167_c0_g2_i1 | green | 0.8933508 |
| TRINITY_DN33005_c0_g2_i1 | green | 0.8930415 |
| TRINITY_DN31186_c0_g4_i3 | green | 0.8926111 |
| TRINITY_DN38986_c0_g2_i1 | green | 0.8925409 |
| TRINITY_DN34517_c0_g7_i1 | green | 0.8922995 |
| TRINITY_DN27384_c0_g1_i1 | green | 0.8920636 |
| TRINITY_DN36534_c2_g12_i1 | green | 0.8915819 |
| TRINITY_DN25374_c0_g2_i1 | green | 0.8911541 |
| TRINITY_DN20558_c0_g1_i1 | green | 0.8910086 |
| TRINITY_DN33946_c0_g5_i1 | green | 0.8909198 |
| TRINITY_DN34156_c1_g1_i1 | green | 0.8908197 |
| TRINITY_DN34704_c4_g1_i2 | green | 0.8902809 |
| TRINITY_DN25591_c0_g1_i2 | green | 0.8902158 |
| TRINITY_DN24732_c0_g1_i1 | green | 0.8900784 |
| TRINITY_DN18633_c0_g1_i1 | green | 0.8900634 |
| TRINITY_DN24037_c0_g1_i1 | green | 0.8898795 |
| TRINITY_DN30188_c0_g1_i7 | green | 0.8894869 |
| TRINITY_DN39135_c0_g3_i1 | green | 0.8893891 |
| TRINITY_DN29141_c0_g1_i2 | green | 0.8892595 |
| TRINITY_DN35326_c0_g2_i4 | green | 0.8891793 |
| TRINITY_DN20918_c0_g1_i1 | green | 0.889152 |
| TRINITY_DN39135_c0_g4_i1 | green | 0.8890572 |
| TRINITY_DN37708_c1_g3_i1 | green | 0.8890494 |
| TRINITY_DN651_c0_g1_i1 | green | 0.8887702 |
| TRINITY_DN37663_c1_g4_i1 | green | 0.8883563 |
| TRINITY_DN23778_c0_g1_i2 | green | 0.8882841 |
| TRINITY_DN23292_c0_g1_i1 | green | 0.887932 |
| TRINITY_DN39101_c3_g4_i1 | green | 0.8876681 |
| TRINITY_DN29619_c0_g1_i1 | green | 0.8873783 |
| TRINITY_DN38187_c1_g1_i4 | green | 0.8866123 |
| TRINITY_DN33724_c0_g2_i1 | green | 0.8863815 |
| TRINITY_DN61901_c0_g1_i1 | green | 0.8861108 |
| TRINITY_DN25407_c1_g3_i1 | green | 0.8860611 |
| TRINITY_DN33004_c1_g10_i1 | green | 0.8856552 |
| TRINITY_DN36430_c0_g1_i4 | green | 0.8854146 |
| TRINITY_DN16081_c0_g1_i1 | green | 0.8850563 |
| TRINITY_DN27605_c0_g1_i1 | green | 0.8849961 |
| TRINITY_DN38256_c0_g1_i1 | green | 0.884583 |
| TRINITY_DN32102_c0_g1_i1 | green | 0.8845747 |
| TRINITY_DN28665_c0_g1_i1 | green | 0.8844973 |
| TRINITY_DN9946_c0_g2_i1 | green | 0.8842087 |
| TRINITY_DN30461_c1_g1_i10 | green | 0.8834847 |
| TRINITY_DN3461_c0_g2_i1 | green | 0.8832075 |
| TRINITY_DN25213_c0_g1_i1 | green | 0.8831068 |
| TRINITY_DN30950_c1_g1_i2 | green | 0.8830096 |
| TRINITY_DN35789_c0_g2_i1 | green | 0.8824763 |
| TRINITY_DN33290_c0_g4_i1 | green | 0.8822374 |
| TRINITY_DN14246_c0_g2_i1 | green | 0.8820918 |
| TRINITY_DN29131_c0_g1_i4 | green | 0.8820172 |
| TRINITY_DN37638_c1_g3_i2 | green | 0.8816702 |
| TRINITY_DN26371_c0_g1_i2 | green | 0.8811287 |
| TRINITY_DN30944_c2_g1_i3 | green | 0.8804006 |
| TRINITY_DN28571_c0_g2_i1 | green | 0.8803579 |
| TRINITY_DN47032_c0_g1_i1 | green | 0.8803496 |
| TRINITY_DN29323_c0_g1_i2 | green | 0.8803358 |
| TRINITY_DN30544_c0_g1_i2 | green | 0.88032 |
| TRINITY_DN39135_c0_g6_i5 | green | 0.8800255 |
| TRINITY_DN37006_c8_g1_i1 | green | 0.8798336 |
| TRINITY_DN31474_c0_g1_i4 | green | 0.8796313 |
| TRINITY_DN35764_c0_g1_i1 | green | 0.8795428 |
| TRINITY_DN29002_c0_g1_i3 | green | 0.8794004 |
| TRINITY_DN26866_c0_g1_i4 | green | 0.8793468 |
| TRINITY_DN39062_c3_g2_i1 | green | 0.8776759 |
| TRINITY_DN38106_c1_g2_i4 | green | 0.8775912 |
| TRINITY_DN35975_c0_g4_i7 | green | 0.8767417 |
| TRINITY_DN28512_c0_g1_i2 | green | 0.8766371 |
| TRINITY_DN25630_c0_g1_i1 | green | 0.8764629 |
| TRINITY_DN33501_c0_g1_i1 | green | 0.8758641 |
| TRINITY_DN5986_c0_g2_i1 | green | 0.8756293 |
| TRINITY_DN24121_c0_g1_i1 | green | 0.8752718 |
| TRINITY_DN25271_c0_g1_i1 | green | 0.8749587 |
| TRINITY_DN24082_c0_g1_i1 | green | 0.8743937 |
| TRINITY_DN24465_c0_g1_i1 | green | 0.8743592 |
| TRINITY_DN38520_c1_g1_i1 | green | 0.8743297 |
| TRINITY_DN25902_c1_g1_i1 | green | 0.873766 |
| TRINITY_DN35112_c1_g5_i3 | green | 0.8735738 |
| TRINITY_DN37031_c0_g2_i2 | green | 0.8735573 |
| TRINITY_DN38931_c0_g1_i2 | green | 0.8729409 |
| TRINITY_DN36238_c0_g2_i7 | green | 0.8727966 |
| TRINITY_DN19235_c0_g1_i1 | green | 0.8725404 |
| TRINITY_DN35802_c0_g1_i1 | green | 0.8716202 |
| TRINITY_DN47531_c0_g1_i1 | green | 0.8714396 |
| TRINITY_DN26000_c1_g3_i4 | green | 0.8713146 |
| TRINITY_DN22372_c0_g2_i1 | green | 0.8712146 |
| TRINITY_DN35898_c1_g10_i1 | green | 0.8711938 |
| TRINITY_DN32425_c1_g1_i3 | green | 0.8708347 |
| TRINITY_DN25777_c0_g1_i1 | green | 0.8704273 |
| TRINITY_DN36789_c1_g3_i1 | green | 0.8694724 |
| TRINITY_DN32585_c0_g1_i7 | green | 0.8681894 |
| TRINITY_DN25570_c0_g1_i1 | green | 0.8680701 |
| TRINITY_DN39101_c2_g1_i1 | green | 0.8679922 |
| TRINITY_DN36094_c1_g1_i3 | green | 0.8672082 |
| TRINITY_DN32544_c0_g12_i1 | green | 0.8667419 |
| TRINITY_DN21968_c0_g3_i1 | green | 0.8663069 |
| TRINITY_DN33812_c0_g6_i9 | green | 0.8656771 |
| TRINITY_DN35637_c1_g2_i1 | green | 0.8656737 |
| TRINITY_DN34019_c0_g1_i1 | green | 0.8651003 |
| TRINITY_DN24266_c0_g1_i1 | green | 0.8650832 |
| TRINITY_DN32555_c0_g1_i1 | green | 0.8648554 |
| TRINITY_DN13609_c0_g1_i1 | green | 0.8645429 |
| TRINITY_DN5162_c0_g1_i1 | green | 0.8644501 |
| TRINITY_DN23739_c0_g1_i1 | green | 0.8643974 |
| TRINITY_DN5320_c0_g1_i1 | green | 0.8642403 |
| TRINITY_DN35806_c0_g2_i8 | green | 0.8639795 |
| TRINITY_DN33414_c0_g2_i1 | green | 0.8637632 |
| TRINITY_DN21986_c0_g1_i2 | green | 0.8635324 |
| TRINITY_DN33168_c1_g1_i1 | green | 0.863485 |
| TRINITY_DN36759_c0_g4_i1 | green | 0.8633094 |
| TRINITY_DN24891_c0_g1_i1 | green | 0.8631412 |
| TRINITY_DN31936_c2_g3_i1 | green | 0.8626894 |
| TRINITY_DN35561_c0_g1_i1 | green | 0.8626629 |
| TRINITY_DN36958_c1_g6_i2 | green | 0.8622478 |
| TRINITY_DN30390_c1_g1_i4 | green | 0.862062 |
| TRINITY_DN36812_c0_g2_i2 | green | 0.8620515 |
| TRINITY_DN35456_c2_g3_i2 | green | 0.8618649 |
| TRINITY_DN34182_c0_g1_i1 | green | 0.8616736 |
| TRINITY_DN37932_c3_g2_i9 | green | 0.8616472 |
| TRINITY_DN31805_c0_g1_i3 | green | 0.8614083 |
| TRINITY_DN35637_c0_g1_i2 | green | 0.8613339 |
| TRINITY_DN50992_c0_g1_i1 | green | 0.8611988 |
| TRINITY_DN34817_c0_g3_i1 | green | 0.860318 |
| TRINITY_DN33882_c0_g1_i2 | green | 0.8602044 |
| TRINITY_DN25948_c1_g1_i3 | green | 0.8601814 |
| TRINITY_DN23988_c0_g1_i1 | green | 0.8597022 |
| TRINITY_DN39315_c0_g1_i3 | green | 0.8595476 |
| TRINITY_DN36157_c1_g15_i2 | green | 0.8594695 |
| TRINITY_DN37117_c0_g3_i1 | green | 0.8579661 |
| TRINITY_DN37567_c3_g8_i1 | green | 0.8576436 |
| TRINITY_DN32050_c0_g1_i4 | green | 0.8572109 |
| TRINITY_DN37293_c2_g1_i16 | green | 0.8572031 |
| TRINITY_DN22814_c0_g1_i1 | green | 0.8568607 |
| TRINITY_DN28631_c0_g1_i2 | green | 0.8567077 |
| TRINITY_DN65795_c0_g1_i1 | green | 0.8566859 |
| TRINITY_DN31441_c0_g1_i4 | green | 0.8565441 |
| TRINITY_DN18671_c0_g2_i1 | green | 0.856462 |
| TRINITY_DN23188_c0_g1_i1 | green | 0.8559539 |
| TRINITY_DN35862_c0_g5_i3 | green | 0.8557991 |
| TRINITY_DN27708_c0_g1_i7 | green | 0.8554754 |
| TRINITY_DN26317_c0_g1_i1 | green | 0.8551987 |
| TRINITY_DN20917_c0_g3_i1 | green | 0.8551572 |
| TRINITY_DN22867_c0_g1_i2 | green | 0.8551095 |
| TRINITY_DN27756_c0_g1_i1 | green | 0.8550935 |
| TRINITY_DN30653_c0_g2_i1 | green | 0.8550556 |
| TRINITY_DN26074_c0_g1_i2 | green | 0.8545236 |
| TRINITY_DN26714_c0_g1_i1 | green | 0.85444 |
| TRINITY_DN35273_c1_g3_i1 | green | 0.8543854 |
| TRINITY_DN32381_c0_g1_i1 | green | 0.8543758 |
| TRINITY_DN31391_c0_g1_i1 | green | 0.8542148 |
| TRINITY_DN38089_c1_g3_i3 | green | 0.8541455 |
| TRINITY_DN34069_c1_g1_i8 | green | 0.8540511 |
| TRINITY_DN30639_c0_g1_i4 | green | 0.8540312 |
| TRINITY_DN31501_c0_g1_i4 | green | 0.8536997 |
| TRINITY_DN20929_c0_g2_i3 | green | 0.8535984 |
| TRINITY_DN38883_c2_g1_i2 | green | 0.8535177 |
| TRINITY_DN31071_c0_g2_i1 | green | 0.8532655 |
| TRINITY_DN34407_c0_g2_i13 | green | 0.8525627 |
| TRINITY_DN30013_c0_g1_i1 | green | 0.8523038 |
| TRINITY_DN56822_c0_g1_i1 | green | 0.8519387 |
| TRINITY_DN33883_c2_g2_i4 | green | 0.8517017 |
| TRINITY_DN36968_c3_g5_i4 | green | 0.8516309 |
| TRINITY_DN23984_c0_g1_i1 | green | 0.8506129 |
| TRINITY_DN27031_c0_g1_i1 | green | 0.8502563 |
| TRINITY_DN62219_c0_g1_i1 | green | 0.8499447 |
| TRINITY_DN39180_c8_g4_i1 | green | 0.8491721 |
| TRINITY_DN29521_c2_g2_i1 | green | 0.8491335 |
| TRINITY_DN23634_c0_g1_i1 | green | 0.8490709 |
| TRINITY_DN23975_c0_g1_i1 | green | 0.8486883 |
| TRINITY_DN35063_c1_g1_i3 | green | 0.8486595 |
| TRINITY_DN34632_c0_g3_i1 | green | 0.8483288 |
| TRINITY_DN38863_c1_g6_i3 | green | 0.8478211 |
| TRINITY_DN37787_c1_g3_i1 | green | 0.84754 |
| TRINITY_DN34235_c0_g1_i1 | green | 0.8472392 |
| TRINITY_DN36224_c0_g1_i1 | green | 0.8472096 |
| TRINITY_DN20205_c0_g1_i1 | green | 0.8463982 |
| TRINITY_DN30239_c0_g1_i7 | green | 0.8463851 |
| TRINITY_DN33329_c1_g1_i5 | green | 0.8463464 |
| TRINITY_DN29162_c0_g1_i1 | green | 0.8458708 |
| TRINITY_DN13549_c0_g1_i1 | green | 0.8456064 |
| TRINITY_DN29028_c0_g1_i1 | green | 0.8455533 |
| TRINITY_DN27308_c0_g1_i1 | green | 0.8454031 |
| TRINITY_DN39144_c2_g12_i1 | green | 0.8442515 |
| TRINITY_DN36425_c0_g1_i4 | green | 0.8442233 |
| TRINITY_DN24580_c0_g1_i1 | green | 0.8442157 |
| TRINITY_DN34809_c0_g1_i1 | green | 0.8441282 |
| TRINITY_DN6340_c0_g1_i1 | green | 0.8440626 |
| TRINITY_DN32203_c0_g4_i1 | green | 0.8438323 |
| TRINITY_DN20168_c0_g1_i1 | green | 0.8437747 |
| TRINITY_DN31452_c0_g1_i9 | green | 0.8431292 |
| TRINITY_DN16222_c0_g4_i1 | green | 0.8428547 |
| TRINITY_DN38829_c1_g4_i1 | green | 0.8427852 |
| TRINITY_DN38883_c2_g3_i4 | green | 0.8423821 |
| TRINITY_DN39125_c0_g10_i1 | green | 0.8420487 |
| TRINITY_DN31580_c2_g15_i1 | green | 0.8419863 |
| TRINITY_DN25044_c0_g1_i1 | green | 0.8419282 |
| TRINITY_DN16094_c0_g1_i2 | green | 0.8413766 |
| TRINITY_DN34775_c0_g1_i1 | green | 0.8413265 |
| TRINITY_DN24933_c0_g1_i1 | green | 0.8408806 |
| TRINITY_DN23153_c0_g1_i1 | green | 0.8405741 |
| TRINITY_DN27604_c0_g1_i1 | green | 0.8405539 |
| TRINITY_DN20662_c0_g1_i1 | green | 0.8405176 |
| TRINITY_DN57291_c0_g1_i1 | green | 0.840175 |
| TRINITY_DN31972_c1_g2_i1 | green | 0.8399744 |
| TRINITY_DN26966_c0_g2_i2 | green | 0.839921 |
| TRINITY_DN67613_c0_g1_i1 | green | 0.8398102 |
| TRINITY_DN36846_c0_g1_i2 | green | 0.83908 |
| TRINITY_DN25396_c0_g2_i1 | green | 0.8385433 |
| TRINITY_DN38496_c1_g6_i1 | green | 0.8382668 |
| TRINITY_DN24461_c0_g2_i1 | green | 0.8381151 |
| TRINITY_DN10834_c0_g1_i2 | green | 0.8379212 |
| TRINITY_DN27003_c0_g1_i2 | green | 0.8376213 |
| TRINITY_DN28484_c0_g1_i2 | green | 0.8376101 |
| TRINITY_DN28346_c0_g1_i2 | green | 0.8372728 |
| TRINITY_DN26457_c0_g1_i2 | green | 0.8370162 |
| TRINITY_DN37817_c0_g2_i12 | green | 0.8368246 |
| TRINITY_DN25534_c0_g1_i1 | green | 0.8367646 |
| TRINITY_DN37340_c0_g1_i4 | green | 0.8360582 |
| TRINITY_DN36367_c0_g3_i1 | green | 0.8355141 |
| TRINITY_DN11253_c0_g1_i1 | green | 0.8353591 |
| TRINITY_DN19682_c0_g1_i1 | green | 0.8353324 |
| TRINITY_DN29993_c0_g1_i3 | green | 0.8353164 |
| TRINITY_DN36262_c0_g5_i1 | green | 0.8349958 |
| TRINITY_DN20805_c0_g2_i1 | green | 0.8349273 |
| TRINITY_DN22284_c0_g4_i1 | green | 0.8349161 |
| TRINITY_DN23821_c0_g1_i1 | green | 0.834828 |
| TRINITY_DN31893_c0_g2_i4 | green | 0.8336378 |
| TRINITY_DN25214_c0_g1_i1 | green | 0.8334027 |
| TRINITY_DN28298_c0_g3_i1 | green | 0.8328247 |
| TRINITY_DN38141_c1_g4_i6 | green | 0.8319914 |
| TRINITY_DN19498_c0_g1_i1 | green | 0.8312079 |
| TRINITY_DN25215_c0_g1_i4 | green | 0.8309554 |
| TRINITY_DN37226_c0_g4_i1 | green | 0.8309359 |
| TRINITY_DN33075_c0_g3_i1 | green | 0.8305102 |
| TRINITY_DN23391_c0_g1_i1 | green | 0.83037 |
| TRINITY_DN18517_c0_g1_i1 | green | 0.8301817 |
| TRINITY_DN30006_c0_g2_i1 | green | 0.8295283 |
| TRINITY_DN38800_c0_g11_i4 | green | 0.8286714 |
| TRINITY_DN26025_c0_g2_i1 | green | 0.8278294 |
| TRINITY_DN28419_c1_g1_i1 | green | 0.8274134 |
| TRINITY_DN27147_c0_g1_i1 | green | 0.8267087 |
| TRINITY_DN37977_c0_g1_i5 | green | 0.8255617 |
| TRINITY_DN32697_c0_g2_i16 | green | 0.8255586 |
| TRINITY_DN27002_c0_g4_i1 | green | 0.8254562 |
| TRINITY_DN25531_c0_g1_i1 | green | 0.8246364 |
| TRINITY_DN34284_c0_g1_i6 | green | 0.8244752 |
| TRINITY_DN36742_c1_g1_i1 | green | 0.8243176 |
| TRINITY_DN27088_c0_g1_i2 | green | 0.8237984 |
| TRINITY_DN38476_c2_g4_i1 | green | 0.8231895 |
| TRINITY_DN27518_c0_g1_i2 | green | 0.8229278 |
| TRINITY_DN27041_c0_g1_i1 | green | 0.8225626 |
| TRINITY_DN39064_c1_g4_i1 | green | 0.8225261 |
| TRINITY_DN16446_c0_g3_i1 | green | 0.8222825 |
| TRINITY_DN30300_c1_g4_i6 | green | 0.8222347 |
| TRINITY_DN27783_c0_g1_i1 | green | 0.8216422 |
| TRINITY_DN20202_c0_g1_i1 | green | 0.8214149 |
| TRINITY_DN32287_c0_g1_i2 | green | 0.8201193 |
| TRINITY_DN29058_c0_g1_i1 | green | 0.819821 |
| TRINITY_DN31477_c0_g8_i1 | green | 0.8198093 |
| TRINITY_DN31061_c0_g1_i2 | green | 0.8196322 |
| TRINITY_DN36862_c4_g2_i1 | green | 0.8192742 |
| TRINITY_DN37645_c3_g4_i3 | green | 0.8184086 |
| TRINITY_DN33869_c0_g1_i1 | green | 0.8174437 |
| TRINITY_DN23976_c0_g1_i1 | green | 0.8174327 |
| TRINITY_DN39254_c0_g3_i1 | green | 0.8170709 |
| TRINITY_DN38980_c0_g1_i1 | green | 0.8170622 |
| TRINITY_DN22704_c0_g1_i1 | green | 0.8170497 |
| TRINITY_DN27723_c0_g1_i4 | green | 0.8153672 |
| TRINITY_DN34734_c0_g2_i2 | green | 0.8149981 |
| TRINITY_DN35144_c0_g1_i3 | green | 0.8145346 |
| TRINITY_DN36586_c0_g2_i5 | green | 0.8141567 |
| TRINITY_DN38436_c1_g2_i1 | green | 0.8133313 |
| TRINITY_DN35598_c0_g1_i2 | green | 0.8131843 |
| TRINITY_DN23850_c0_g3_i1 | green | 0.810606 |
| TRINITY_DN45160_c0_g1_i1 | green | 0.8098573 |
| TRINITY_DN38656_c0_g2_i11 | green | 0.8097773 |
| TRINITY_DN26216_c0_g1_i2 | green | 0.808859 |
| TRINITY_DN35468_c0_g9_i1 | green | 0.8087341 |
| TRINITY_DN34620_c0_g1_i5 | green | 0.8087222 |
| TRINITY_DN33161_c0_g3_i1 | green | 0.8083503 |
| TRINITY_DN24967_c0_g1_i1 | green | 0.8076767 |
| TRINITY_DN33244_c0_g1_i3 | green | 0.8071737 |
| TRINITY_DN37672_c2_g4_i1 | green | 0.8071462 |
| TRINITY_DN21406_c0_g1_i1 | green | 0.8071456 |
| TRINITY_DN32552_c0_g1_i1 | green | 0.8066099 |
| TRINITY_DN38808_c0_g1_i3 | green | 0.8065533 |
| TRINITY_DN31726_c0_g4_i1 | green | 0.8062716 |
| TRINITY_DN37252_c0_g1_i3 | green | 0.8055021 |
| TRINITY_DN33251_c0_g1_i1 | green | 0.8053375 |
| TRINITY_DN37953_c0_g3_i1 | green | 0.8052662 |
| TRINITY_DN31086_c0_g2_i4 | green | 0.8052411 |
| TRINITY_DN30180_c1_g2_i1 | green | 0.8049232 |
| TRINITY_DN37314_c1_g2_i1 | green | 0.8035598 |
| TRINITY_DN29030_c0_g1_i5 | green | 0.8031181 |
| TRINITY_DN29909_c0_g1_i1 | green | 0.8029199 |
| TRINITY_DN37912_c0_g2_i1 | green | 0.8020716 |
| TRINITY_DN26749_c0_g1_i1 | green | 0.801376 |
| TRINITY_DN28340_c0_g1_i1 | green | 0.7998383 |
| TRINITY_DN30671_c0_g1_i1 | green | 0.7996977 |
| TRINITY_DN39138_c1_g1_i1 | green | 0.7990975 |
| TRINITY_DN34671_c0_g1_i1 | green | 0.7990186 |
| TRINITY_DN32866_c0_g1_i2 | green | 0.7988606 |
| TRINITY_DN35486_c0_g2_i5 | green | 0.7982625 |
| TRINITY_DN25529_c0_g1_i1 | green | 0.7976749 |
| TRINITY_DN26391_c0_g2_i1 | green | 0.7975008 |
| TRINITY_DN18258_c0_g3_i1 | green | 0.7971709 |
| TRINITY_DN36995_c0_g1_i1 | green | 0.7970696 |
| TRINITY_DN36108_c0_g3_i3 | green | 0.7965988 |
| TRINITY_DN29810_c0_g1_i1 | green | 0.7964016 |
| TRINITY_DN39019_c1_g1_i6 | green | 0.796138 |
| TRINITY_DN23494_c0_g1_i1 | green | 0.7956949 |
| TRINITY_DN28480_c0_g2_i2 | green | 0.7952638 |
| TRINITY_DN15593_c0_g1_i1 | green | 0.7943984 |
| TRINITY_DN34132_c0_g3_i3 | green | 0.7942746 |
| TRINITY_DN26078_c0_g1_i1 | green | 0.7941129 |
| TRINITY_DN35295_c1_g2_i1 | green | 0.7940329 |
| TRINITY_DN33201_c0_g2_i7 | green | 0.793983 |
| TRINITY_DN29420_c1_g1_i1 | green | 0.7937566 |
| TRINITY_DN37040_c1_g1_i1 | green | 0.793098 |
| TRINITY_DN40535_c0_g1_i1 | green | 0.7927124 |
| TRINITY_DN3978_c0_g1_i1 | green | 0.792281 |
| TRINITY_DN5441_c0_g1_i1 | green | 0.7909322 |
| TRINITY_DN24011_c0_g2_i1 | green | 0.7896591 |
| TRINITY_DN36352_c1_g4_i1 | green | 0.7888064 |
| TRINITY_DN29562_c1_g6_i1 | green | 0.7881241 |
| TRINITY_DN29420_c0_g1_i1 | green | 0.7873117 |
| TRINITY_DN30677_c0_g1_i1 | green | 0.7871281 |
| TRINITY_DN36253_c0_g1_i7 | green | 0.7869526 |
| TRINITY_DN39273_c1_g4_i1 | green | 0.7863222 |
| TRINITY_DN29245_c1_g2_i1 | green | 0.784033 |
| TRINITY_DN20432_c0_g1_i1 | green | 0.7822421 |
| TRINITY_DN35526_c2_g15_i1 | green | 0.7817563 |
| TRINITY_DN33812_c0_g7_i1 | green | 0.7810498 |
| TRINITY_DN32037_c0_g1_i1 | green | 0.7809122 |
| TRINITY_DN37934_c0_g3_i6 | green | 0.7803247 |
| TRINITY_DN27615_c1_g1_i2 | green | 0.7802643 |
| TRINITY_DN22441_c0_g1_i1 | green | 0.7799285 |
| TRINITY_DN20004_c0_g1_i1 | green | 0.7799022 |
| TRINITY_DN37674_c0_g4_i1 | green | 0.7781176 |
| TRINITY_DN22923_c0_g1_i1 | green | 0.7779237 |
| TRINITY_DN14403_c0_g1_i1 | green | 0.7775423 |
| TRINITY_DN22947_c0_g2_i1 | green | 0.7774246 |
| TRINITY_DN33992_c1_g1_i1 | green | 0.7772934 |
| TRINITY_DN32664_c0_g2_i4 | green | 0.7772018 |
| TRINITY_DN51111_c0_g1_i1 | green | 0.7764504 |
| TRINITY_DN20788_c0_g1_i2 | green | 0.775026 |
| TRINITY_DN18933_c0_g1_i1 | green | 0.7749482 |
| TRINITY_DN35803_c0_g1_i8 | green | 0.7743291 |
| TRINITY_DN25520_c0_g1_i1 | green | 0.7742649 |
| TRINITY_DN37253_c1_g3_i2 | green | 0.7723008 |
| TRINITY_DN28752_c1_g2_i1 | green | 0.7721245 |
| TRINITY_DN38877_c0_g3_i1 | green | 0.7688023 |
| TRINITY_DN35456_c2_g5_i1 | green | 0.7674811 |
| TRINITY_DN37163_c0_g2_i5 | green | 0.7669805 |
| TRINITY_DN32850_c1_g3_i2 | green | 0.7668894 |
| TRINITY_DN10366_c0_g1_i1 | green | 0.7668699 |
| TRINITY_DN33509_c0_g2_i1 | green | 0.7662391 |
| TRINITY_DN31343_c0_g1_i1 | green | 0.7634304 |
| TRINITY_DN30242_c0_g2_i1 | green | 0.7631193 |
| TRINITY_DN26359_c0_g1_i1 | green | 0.7626778 |
| TRINITY_DN29292_c0_g1_i3 | green | 0.7621355 |
| TRINITY_DN23822_c0_g1_i1 | green | 0.7615751 |
| TRINITY_DN37723_c0_g2_i1 | green | 0.7605579 |
| TRINITY_DN27741_c0_g1_i1 | green | 0.7599329 |
| TRINITY_DN26960_c0_g1_i1 | green | 0.7593742 |
| TRINITY_DN26434_c0_g1_i1 | green | 0.758169 |
| TRINITY_DN35495_c0_g1_i4 | green | 0.7578508 |
| TRINITY_DN57305_c0_g1_i1 | green | 0.7575344 |
| TRINITY_DN31174_c0_g1_i2 | green | 0.7572985 |
| TRINITY_DN37255_c2_g4_i4 | green | 0.7571452 |
| TRINITY_DN25542_c0_g3_i1 | green | 0.7570535 |
| TRINITY_DN24715_c0_g1_i2 | green | 0.7567168 |
| TRINITY_DN35456_c1_g1_i1 | green | 0.7564564 |
| TRINITY_DN5927_c0_g1_i1 | green | 0.7561219 |
| TRINITY_DN35328_c1_g1_i4 | green | 0.7552938 |
| TRINITY_DN13921_c0_g1_i1 | green | 0.7544492 |
| TRINITY_DN27458_c0_g1_i1 | green | 0.7537857 |
| TRINITY_DN27002_c0_g2_i1 | green | 0.7518539 |
| TRINITY_DN39089_c1_g3_i1 | green | 0.7516903 |
| TRINITY_DN25244_c0_g1_i2 | green | 0.7503835 |
| TRINITY_DN27211_c0_g1_i1 | green | 0.750057 |
| TRINITY_DN69180_c0_g1_i1 | green | 0.7497575 |
| TRINITY_DN38511_c1_g3_i1 | green | 0.7483658 |
| TRINITY_DN35984_c1_g7_i1 | green | 0.7479856 |
| TRINITY_DN35533_c0_g5_i2 | green | 0.7475136 |
| TRINITY_DN27390_c0_g1_i2 | green | 0.7457959 |
| TRINITY_DN31926_c0_g2_i1 | green | 0.7457777 |
| TRINITY_DN22853_c0_g1_i1 | green | 0.7448702 |
| TRINITY_DN8527_c0_g1_i1 | green | 0.7445324 |
| TRINITY_DN64771_c0_g1_i1 | green | 0.7434085 |
| TRINITY_DN31163_c0_g2_i1 | green | 0.7432023 |
| TRINITY_DN33302_c1_g5_i1 | green | 0.7427013 |
| TRINITY_DN38949_c1_g5_i1 | green | 0.742673 |
| TRINITY_DN37520_c2_g3_i1 | green | 0.7402248 |
| TRINITY_DN30492_c0_g1_i1 | green | 0.7401399 |
| TRINITY_DN37800_c0_g3_i2 | green | 0.7395399 |
| TRINITY_DN31198_c0_g3_i1 | green | 0.7362892 |
| TRINITY_DN71183_c0_g1_i1 | green | 0.7348689 |
| TRINITY_DN27702_c0_g1_i1 | green | 0.7323902 |
| TRINITY_DN15778_c0_g1_i1 | green | 0.7303163 |
| TRINITY_DN16123_c0_g1_i1 | green | 0.7299295 |
| TRINITY_DN29008_c0_g1_i2 | green | 0.7261824 |
| TRINITY_DN23212_c0_g1_i1 | green | 0.7254076 |
| TRINITY_DN19774_c0_g2_i1 | green | 0.7187328 |
| TRINITY_DN39166_c1_g3_i1 | green | 0.7166825 |
| TRINITY_DN27018_c0_g2_i1 | green | 0.7152831 |
| TRINITY_DN29826_c0_g1_i3 | green | 0.7138378 |
| TRINITY_DN36534_c2_g11_i1 | green | 0.7134637 |
| TRINITY_DN31556_c0_g1_i1 | green | 0.7131235 |
| TRINITY_DN22735_c0_g1_i1 | green | 0.712148 |
| TRINITY_DN32179_c0_g1_i3 | green | 0.7089356 |
| TRINITY_DN19220_c0_g1_i1 | green | 0.7076912 |
| TRINITY_DN33264_c0_g1_i1 | green | 0.7072939 |
| TRINITY_DN20979_c0_g1_i1 | green | 0.7071426 |
| TRINITY_DN26531_c1_g1_i8 | green | 0.7021387 |
| TRINITY_DN29479_c0_g1_i1 | green | 0.7020679 |
| TRINITY_DN33736_c1_g1_i1 | green | 0.7007136 |
| TRINITY_DN27632_c0_g3_i2 | green | 0.6945269 |
| TRINITY_DN23063_c0_g1_i1 | green | 0.6941782 |
| TRINITY_DN34339_c1_g4_i1 | green | 0.6878281 |
| TRINITY_DN39191_c3_g4_i5 | green | 0.6829356 |
| TRINITY_DN31776_c0_g1_i2 | green | 0.6828759 |
| TRINITY_DN39207_c0_g3_i1 | green | 0.6744946 |
| TRINITY_DN37751_c1_g1_i2 | green | 0.6716688 |
| TRINITY_DN19368_c0_g1_i1 | green | 0.6689303 |
| TRINITY_DN28147_c0_g1_i2 | green | 0.6688964 |
| TRINITY_DN22513_c0_g1_i1 | green | 0.6684674 |
| TRINITY_DN55693_c0_g1_i1 | green | 0.6674939 |
| TRINITY_DN20423_c0_g1_i1 | green | 0.6639496 |
| TRINITY_DN32548_c0_g2_i2 | green | 0.6602643 |
| TRINITY_DN27806_c0_g1_i5 | green | 0.6517492 |
| TRINITY_DN28780_c0_g1_i1 | green | 0.6428592 |
| TRINITY_DN24294_c0_g1_i1 | green | 0.633247 |
| TRINITY_DN31929_c0_g2_i3 | green | 0.5523214 |
| TRINITY_DN34267_c1_g3_i1 | green | 0.5284839 |
| TRINITY_DN38367_c1_g4_i1 | green | -0.489033 |
| TRINITY_DN22674_c0_g2_i1 | green | -0.512561 |
| TRINITY_DN28875_c0_g2_i2 | green | -0.52536 |
| TRINITY_DN30622_c0_g1_i1 | green | -0.554667 |
| TRINITY_DN28869_c1_g4_i1 | green | -0.560259 |
| TRINITY_DN38279_c0_g6_i1 | green | -0.579279 |
| TRINITY_DN31416_c0_g1_i2 | green | -0.614234 |
| TRINITY_DN35063_c0_g1_i1 | green | -0.653556 |
| TRINITY_DN36159_c1_g1_i2 | green | -0.665407 |
| TRINITY_DN30446_c0_g2_i6 | green | -0.679014 |
| TRINITY_DN37973_c1_g6_i1 | green | -0.692086 |
| TRINITY_DN39216_c3_g8_i1 | green | -0.694074 |
| TRINITY_DN34839_c2_g2_i3 | green | -0.700493 |
| TRINITY_DN36669_c0_g1_i3 | green | -0.713795 |
| TRINITY_DN20841_c0_g1_i1 | green | -0.719576 |
| TRINITY_DN32084_c0_g2_i4 | green | -0.729426 |
| TRINITY_DN32442_c0_g1_i1 | green | -0.73359 |
| TRINITY_DN26214_c0_g1_i2 | green | -0.738011 |
| TRINITY_DN35275_c0_g3_i1 | green | -0.738935 |
| TRINITY_DN38899_c0_g1_i4 | green | -0.739889 |
| TRINITY_DN25295_c0_g1_i1 | green | -0.74038 |
| TRINITY_DN31927_c0_g2_i1 | green | -0.742378 |
| TRINITY_DN31091_c0_g2_i11 | green | -0.744729 |
| TRINITY_DN32823_c1_g5_i1 | green | -0.74952 |
| TRINITY_DN19334_c0_g1_i1 | green | -0.754048 |
| TRINITY_DN39230_c3_g6_i1 | green | -0.759689 |
| TRINITY_DN68948_c0_g1_i1 | green | -0.777068 |
| TRINITY_DN31514_c0_g1_i10 | green | -0.77707 |
| TRINITY_DN16174_c0_g1_i1 | green | -0.777326 |
| TRINITY_DN35169_c0_g1_i7 | green | -0.778306 |
| TRINITY_DN23454_c0_g2_i1 | green | -0.778625 |
| TRINITY_DN38035_c0_g1_i19 | green | -0.781058 |
| TRINITY_DN38509_c1_g3_i2 | green | -0.78164 |
| TRINITY_DN31275_c0_g1_i1 | green | -0.784254 |
| TRINITY_DN27955_c0_g1_i1 | green | -0.785794 |
| TRINITY_DN35044_c0_g1_i5 | green | -0.790898 |
| TRINITY_DN39320_c0_g3_i1 | green | -0.795827 |
| TRINITY_DN22969_c0_g1_i1 | green | -0.796794 |
| TRINITY_DN34538_c1_g9_i1 | green | -0.800521 |
| TRINITY_DN38251_c2_g6_i1 | green | -0.801071 |
| TRINITY_DN31457_c0_g1_i1 | green | -0.80351 |
| TRINITY_DN33382_c0_g1_i1 | green | -0.8064 |
| TRINITY_DN38251_c2_g5_i1 | green | -0.807549 |
| TRINITY_DN38653_c0_g3_i1 | green | -0.808531 |
| TRINITY_DN28951_c0_g1_i1 | green | -0.812102 |
| TRINITY_DN39167_c3_g1_i4 | green | -0.815458 |
| TRINITY_DN37904_c0_g2_i16 | green | -0.816338 |
| TRINITY_DN21593_c0_g1_i1 | green | -0.81654 |
| TRINITY_DN32878_c0_g2_i1 | green | -0.819164 |
| TRINITY_DN33517_c1_g2_i5 | green | -0.823373 |
| TRINITY_DN39322_c1_g3_i9 | green | -0.825047 |
| TRINITY_DN26929_c0_g1_i1 | green | -0.825094 |
| TRINITY_DN37438_c0_g1_i14 | green | -0.828452 |
| TRINITY_DN19496_c0_g1_i1 | green | -0.831372 |
| TRINITY_DN21096_c0_g2_i1 | green | -0.831885 |
| TRINITY_DN36106_c3_g1_i1 | green | -0.833653 |
| TRINITY_DN27909_c1_g1_i1 | green | -0.838391 |
| TRINITY_DN21193_c0_g2_i1 | green | -0.843161 |
| TRINITY_DN26260_c0_g1_i1 | green | -0.84919 |
| TRINITY_DN30618_c0_g1_i1 | green | -0.85405 |
| TRINITY_DN19390_c0_g1_i1 | green | -0.854187 |
| TRINITY_DN33355_c0_g1_i4 | green | -0.855592 |
| TRINITY_DN36330_c0_g4_i1 | green | -0.855666 |
| TRINITY_DN35228_c0_g2_i5 | green | -0.857381 |
| TRINITY_DN37611_c0_g1_i1 | green | -0.858884 |
| TRINITY_DN29835_c0_g1_i1 | green | -0.868565 |
| TRINITY_DN32931_c0_g1_i3 | green | -0.871357 |
| TRINITY_DN12017_c0_g1_i1 | green | -0.873105 |
| TRINITY_DN26096_c0_g1_i1 | green | -0.879219 |
| TRINITY_DN27011_c0_g1_i1 | green | -0.89109 |
| TRINITY_DN31351_c0_g2_i3 | green | -0.891999 |
| TRINITY_DN25787_c0_g4_i2 | green | -0.892147 |
| TRINITY_DN34842_c0_g1_i3 | green | -0.903777 |
| TRINITY_DN37646_c2_g1_i16 | green | -0.910527 |
| TRINITY_DN29657_c0_g1_i3 | green | -0.912694 |
| TRINITY_DN30831_c0_g1_i1 | green | -0.917863 |
| TRINITY_DN34781_c0_g1_i1 | green | -0.941283 |
| TRINITY_DN33974_c0_g3_i3 | grey | 0.9148117 |
| TRINITY_DN13512_c0_g1_i1 | grey | 0.9001073 |
| TRINITY_DN20355_c0_g1_i2 | grey | 0.8927978 |
| TRINITY_DN30445_c0_g2_i4 | grey | 0.8753149 |
| TRINITY_DN39171_c2_g8_i1 | grey | 0.8665306 |
| TRINITY_DN24626_c0_g1_i4 | grey | 0.8610267 |
| TRINITY_DN30232_c0_g1_i1 | grey | 0.8547429 |
| TRINITY_DN28335_c0_g1_i6 | grey | 0.8539645 |
| TRINITY_DN28493_c0_g2_i1 | grey | 0.8317363 |
| TRINITY_DN23774_c0_g1_i1 | grey | 0.8205157 |
| TRINITY_DN28517_c0_g1_i2 | grey | 0.8173633 |
| TRINITY_DN28634_c0_g1_i1 | grey | 0.8133313 |
| TRINITY_DN28708_c0_g1_i2 | grey | 0.8033529 |
| TRINITY_DN32494_c2_g7_i1 | grey | 0.8029679 |
| TRINITY_DN21978_c0_g1_i1 | grey | 0.800349 |
| TRINITY_DN38343_c1_g1_i1 | grey | 0.7977019 |
| TRINITY_DN22685_c0_g1_i1 | grey | 0.7921201 |
| TRINITY_DN51707_c0_g1_i1 | grey | 0.7899006 |
| TRINITY_DN34263_c0_g1_i1 | grey | 0.7828892 |
| TRINITY_DN33756_c0_g1_i2 | grey | 0.7798258 |
| TRINITY_DN39286_c4_g4_i1 | grey | 0.7620566 |
| TRINITY_DN25484_c0_g1_i1 | grey | 0.739095 |
| TRINITY_DN36588_c5_g2_i6 | grey | 0.7379018 |
| TRINITY_DN38301_c1_g6_i1 | grey | 0.7277401 |
| TRINITY_DN28112_c0_g3_i3 | grey | 0.727032 |
| TRINITY_DN4781_c0_g1_i1 | grey | 0.7230009 |
| TRINITY_DN37650_c1_g1_i1 | grey | 0.7221795 |
| TRINITY_DN39311_c4_g2_i1 | grey | 0.71348 |
| TRINITY_DN36886_c0_g1_i2 | grey | 0.7039539 |
| TRINITY_DN39161_c2_g3_i3 | grey | 0.7032826 |
| TRINITY_DN27506_c0_g1_i1 | grey | 0.6977572 |
| TRINITY_DN29787_c1_g1_i4 | grey | 0.6959718 |
| TRINITY_DN20181_c0_g1_i2 | grey | 0.6939012 |
| TRINITY_DN37243_c1_g4_i1 | grey | 0.6911128 |
| TRINITY_DN35721_c1_g2_i4 | grey | 0.6905097 |
| TRINITY_DN27355_c0_g1_i1 | grey | 0.67848 |
| TRINITY_DN34547_c0_g5_i2 | grey | 0.6768879 |
| TRINITY_DN30641_c1_g1_i4 | grey | 0.6674491 |
| TRINITY_DN39069_c3_g9_i1 | grey | 0.6613935 |
| TRINITY_DN20230_c0_g1_i1 | grey | 0.6588118 |
| TRINITY_DN25572_c0_g1_i1 | grey | 0.6484998 |
| TRINITY_DN37889_c0_g1_i1 | grey | 0.6241737 |
| TRINITY_DN25419_c0_g1_i1 | grey | 0.623567 |
| TRINITY_DN34242_c0_g2_i1 | grey | 0.6089043 |
| TRINITY_DN36667_c0_g1_i6 | grey | 0.6045997 |
| TRINITY_DN35776_c3_g1_i8 | grey | 0.5802764 |
| TRINITY_DN25614_c0_g1_i1 | grey | 0.5695357 |
| TRINITY_DN43711_c0_g1_i1 | grey | 0.5654354 |
| TRINITY_DN39271_c6_g12_i7 | grey | 0.5380407 |
| TRINITY_DN18606_c0_g1_i2 | grey | 0.5152067 |
| TRINITY_DN19091_c0_g1_i1 | grey | 0.4787215 |
| TRINITY_DN5606_c0_g1_i1 | grey | 0.4261497 |
| TRINITY_DN17358_c0_g1_i2 | grey | 0.2149613 |
| TRINITY_DN39180_c7_g3_i1 | grey | 0.1852966 |
| TRINITY_DN41075_c0_g1_i1 | grey | 0.1829061 |
| TRINITY_DN25864_c0_g1_i1 | grey | 0.0737192 |
| TRINITY_DN12831_c0_g1_i1 | grey | -0.159475 |
| TRINITY_DN21248_c0_g1_i1 | grey | -0.300907 |
| TRINITY_DN24685_c0_g2_i1 | grey | -0.380552 |
| TRINITY_DN28882_c0_g3_i1 | grey | -0.592391 |
| TRINITY_DN29950_c0_g1_i1 | grey | -0.600186 |
| TRINITY_DN30896_c0_g1_i1 | grey | -0.604216 |
| TRINITY_DN34553_c0_g2_i1 | grey | -0.614878 |
| TRINITY_DN29465_c0_g1_i1 | grey | -0.622866 |
| TRINITY_DN61753_c0_g1_i1 | grey | -0.640481 |
| TRINITY_DN35752_c1_g2_i3 | grey | -0.646416 |
| TRINITY_DN37478_c0_g1_i5 | grey | -0.711375 |
| TRINITY_DN23945_c0_g2_i1 | grey | -0.738847 |
| TRINITY_DN25932_c0_g1_i2 | grey | -0.742357 |
| TRINITY_DN39187_c1_g5_i1 | grey | -0.74809 |
| TRINITY_DN35869_c0_g1_i20 | grey | -0.758156 |
| TRINITY_DN39313_c1_g12_i1 | grey | -0.784417 |
| TRINITY_DN34023_c0_g3_i1 | grey | -0.791652 |
| TRINITY_DN36126_c1_g2_i16 | grey | -0.801886 |
| TRINITY_DN37961_c0_g2_i7 | grey | -0.802483 |
| TRINITY_DN39303_c1_g3_i1 | grey | -0.841031 |
| TRINITY_DN38068_c0_g1_i2 | grey | -0.847698 |
| TRINITY_DN37398_c3_g5_i1 | grey | -0.855828 |
| TRINITY_DN24001_c0_g1_i2 | grey | -0.860424 |
| TRINITY_DN23419_c0_g1_i4 | grey | -0.867732 |
| TRINITY_DN38275_c1_g2_i1 | grey | -0.926661 |
| TRINITY_DN37359_c0_g2_i1 | red | 0.9898075 |
| TRINITY_DN39041_c3_g1_i2 | red | 0.9897276 |
| TRINITY_DN39159_c0_g6_i16 | red | 0.987344 |
| TRINITY_DN39255_c2_g5_i1 | red | 0.9869555 |
| TRINITY_DN35059_c0_g2_i4 | red | 0.985116 |
| TRINITY_DN34503_c1_g5_i12 | red | 0.9845339 |
| TRINITY_DN38783_c0_g3_i4 | red | 0.9834264 |
| TRINITY_DN39125_c0_g5_i1 | red | 0.9831539 |
| TRINITY_DN39068_c0_g11_i1 | red | 0.982743 |
| TRINITY_DN38953_c0_g3_i5 | red | 0.9823948 |
| TRINITY_DN24872_c0_g1_i1 | red | 0.9819801 |
| TRINITY_DN26140_c0_g3_i1 | red | 0.9816935 |
| TRINITY_DN39068_c0_g24_i1 | red | 0.9784787 |
| TRINITY_DN26292_c0_g1_i1 | red | 0.9773684 |
| TRINITY_DN37083_c0_g1_i7 | red | 0.9772465 |
| TRINITY_DN28882_c0_g2_i2 | red | 0.9768421 |
| TRINITY_DN38676_c1_g2_i1 | red | 0.9763223 |
| TRINITY_DN34590_c2_g1_i1 | red | 0.9759094 |
| TRINITY_DN24387_c0_g2_i1 | red | 0.9741226 |
| TRINITY_DN23767_c0_g1_i2 | red | 0.9721738 |
| TRINITY_DN20099_c0_g1_i2 | red | 0.9708757 |
| TRINITY_DN34848_c0_g1_i3 | red | 0.970163 |
| TRINITY_DN26824_c0_g2_i1 | red | 0.9701082 |
| TRINITY_DN39255_c2_g9_i2 | red | 0.9700063 |
| TRINITY_DN25329_c0_g4_i1 | red | 0.968917 |
| TRINITY_DN25741_c0_g2_i1 | red | 0.9688463 |
| TRINITY_DN37310_c0_g1_i8 | red | 0.968626 |
| TRINITY_DN39299_c4_g2_i3 | red | 0.9675675 |
| TRINITY_DN28154_c0_g2_i1 | red | 0.9675607 |
| TRINITY_DN31999_c0_g1_i3 | red | 0.9658794 |
| TRINITY_DN38483_c1_g10_i1 | red | 0.9652255 |
| TRINITY_DN39037_c0_g1_i1 | red | 0.964168 |
| TRINITY_DN34697_c0_g1_i31 | red | 0.9637536 |
| TRINITY_DN39080_c0_g2_i1 | red | 0.9629796 |
| TRINITY_DN31950_c0_g5_i1 | red | 0.9619835 |
| TRINITY_DN38653_c0_g4_i1 | red | 0.9606444 |
| TRINITY_DN21810_c0_g2_i1 | red | 0.9600589 |
| TRINITY_DN35910_c2_g4_i2 | red | 0.959819 |
| TRINITY_DN58276_c0_g1_i1 | red | 0.9596028 |
| TRINITY_DN39237_c5_g14_i1 | red | 0.9590034 |
| TRINITY_DN21865_c0_g1_i1 | red | 0.9589633 |
| TRINITY_DN33754_c0_g1_i3 | red | 0.9589571 |
| TRINITY_DN35548_c0_g3_i1 | red | 0.9586968 |
| TRINITY_DN26000_c1_g1_i1 | red | 0.9586572 |
| TRINITY_DN39060_c0_g2_i5 | red | 0.9581862 |
| TRINITY_DN27953_c0_g5_i1 | red | 0.95804 |
| TRINITY_DN38725_c0_g1_i1 | red | 0.9574506 |
| TRINITY_DN35372_c1_g1_i7 | red | 0.9569275 |
| TRINITY_DN32153_c0_g1_i2 | red | 0.9567168 |
| TRINITY_DN33897_c0_g3_i1 | red | 0.9561063 |
| TRINITY_DN21076_c0_g2_i1 | red | 0.9555591 |
| TRINITY_DN29655_c0_g1_i1 | red | 0.9555118 |
| TRINITY_DN19725_c0_g1_i1 | red | 0.9553077 |
| TRINITY_DN31552_c0_g2_i22 | red | 0.9552593 |
| TRINITY_DN38173_c1_g7_i2 | red | 0.9552269 |
| TRINITY_DN34607_c0_g1_i3 | red | 0.9545847 |
| TRINITY_DN39127_c1_g12_i8 | red | 0.9545754 |
| TRINITY_DN36946_c0_g4_i13 | red | 0.9544777 |
| TRINITY_DN38888_c2_g17_i4 | red | 0.9539444 |
| TRINITY_DN31433_c0_g1_i1 | red | 0.9537336 |
| TRINITY_DN39138_c1_g6_i3 | red | 0.9537145 |
| TRINITY_DN36377_c0_g4_i1 | red | 0.9536183 |
| TRINITY_DN31479_c0_g1_i1 | red | 0.9518738 |
| TRINITY_DN38891_c0_g2_i2 | red | 0.9516336 |
| TRINITY_DN35622_c0_g1_i12 | red | 0.9515747 |
| TRINITY_DN36438_c0_g3_i1 | red | 0.9515487 |
| TRINITY_DN20455_c0_g1_i1 | red | 0.9515388 |
| TRINITY_DN31351_c0_g1_i1 | red | 0.9508562 |
| TRINITY_DN34260_c0_g1_i1 | red | 0.9507522 |
| TRINITY_DN30190_c2_g4_i1 | red | 0.9506078 |
| TRINITY_DN38888_c2_g8_i1 | red | 0.9505659 |
| TRINITY_DN22432_c0_g1_i1 | red | 0.950231 |
| TRINITY_DN27426_c0_g1_i1 | red | 0.9500421 |
| TRINITY_DN32860_c0_g1_i4 | red | 0.9499764 |
| TRINITY_DN39016_c3_g5_i8 | red | 0.9499645 |
| TRINITY_DN31924_c0_g4_i1 | red | 0.9498205 |
| TRINITY_DN29898_c0_g1_i1 | red | 0.9495537 |
| TRINITY_DN37079_c0_g4_i6 | red | 0.949494 |
| TRINITY_DN37567_c3_g3_i3 | red | 0.9489695 |
| TRINITY_DN35961_c1_g4_i1 | red | 0.9484808 |
| TRINITY_DN37966_c0_g1_i2 | red | 0.9483258 |
| TRINITY_DN21858_c0_g1_i1 | red | 0.9481023 |
| TRINITY_DN39341_c7_g7_i6 | red | 0.9480808 |
| TRINITY_DN33585_c0_g1_i8 | red | 0.9480655 |
| TRINITY_DN38884_c0_g3_i1 | red | 0.9478974 |
| TRINITY_DN36551_c0_g1_i1 | red | 0.9477362 |
| TRINITY_DN37951_c0_g1_i1 | red | 0.9475184 |
| TRINITY_DN22709_c0_g1_i2 | red | 0.9468979 |
| TRINITY_DN37084_c0_g3_i3 | red | 0.9465781 |
| TRINITY_DN28430_c0_g1_i1 | red | 0.9462076 |
| TRINITY_DN38884_c0_g7_i1 | red | 0.9460441 |
| TRINITY_DN28571_c0_g1_i1 | red | 0.9457207 |
| TRINITY_DN32544_c0_g10_i1 | red | 0.9457131 |
| TRINITY_DN29922_c0_g1_i3 | red | 0.9456651 |
| TRINITY_DN45311_c0_g1_i1 | red | 0.9453609 |
| TRINITY_DN22003_c0_g1_i1 | red | 0.9449655 |
| TRINITY_DN33795_c0_g1_i3 | red | 0.9446106 |
| TRINITY_DN23133_c0_g2_i1 | red | 0.9445178 |
| TRINITY_DN39343_c19_g4_i2 | red | 0.9444648 |
| TRINITY_DN4171_c0_g1_i1 | red | 0.9442371 |
| TRINITY_DN20600_c0_g1_i1 | red | 0.9441426 |
| TRINITY_DN18933_c0_g2_i1 | red | 0.9431695 |
| TRINITY_DN31249_c0_g1_i2 | red | 0.9431408 |
| TRINITY_DN39317_c6_g21_i2 | red | 0.9428385 |
| TRINITY_DN25533_c0_g1_i2 | red | 0.9422758 |
| TRINITY_DN25490_c0_g1_i1 | red | 0.9411973 |
| TRINITY_DN25808_c0_g1_i3 | red | 0.9410462 |
| TRINITY_DN31667_c0_g5_i1 | red | 0.9407714 |
| TRINITY_DN36236_c1_g2_i1 | red | 0.9404786 |
| TRINITY_DN52843_c0_g1_i1 | red | 0.9404709 |
| TRINITY_DN25798_c0_g1_i1 | red | 0.940328 |
| TRINITY_DN35526_c1_g1_i2 | red | 0.9402433 |
| TRINITY_DN38343_c1_g3_i5 | red | 0.9401865 |
| TRINITY_DN19847_c0_g1_i1 | red | 0.9401224 |
| TRINITY_DN28533_c0_g2_i1 | red | 0.9399143 |
| TRINITY_DN39271_c6_g1_i1 | red | 0.9396482 |
| TRINITY_DN39238_c1_g8_i1 | red | 0.9395459 |
| TRINITY_DN31266_c0_g1_i3 | red | 0.9392874 |
| TRINITY_DN31562_c0_g1_i2 | red | 0.9391157 |
| TRINITY_DN26920_c0_g2_i1 | red | 0.9389176 |
| TRINITY_DN26824_c0_g3_i1 | red | 0.9388027 |
| TRINITY_DN30433_c0_g4_i1 | red | 0.9381425 |
| TRINITY_DN54928_c0_g1_i1 | red | 0.9360795 |
| TRINITY_DN39220_c6_g10_i1 | red | 0.9360532 |
| TRINITY_DN38821_c3_g3_i1 | red | 0.9353371 |
| TRINITY_DN29717_c0_g1_i1 | red | 0.9344839 |
| TRINITY_DN39283_c3_g4_i2 | red | 0.9344577 |
| TRINITY_DN30845_c0_g1_i4 | red | 0.9343953 |
| TRINITY_DN31344_c0_g1_i4 | red | 0.9343264 |
| TRINITY_DN36952_c0_g12_i1 | red | 0.9334806 |
| TRINITY_DN37128_c0_g4_i2 | red | 0.933277 |
| TRINITY_DN38888_c2_g13_i1 | red | 0.9332299 |
| TRINITY_DN13626_c0_g1_i1 | red | 0.9331425 |
| TRINITY_DN33828_c0_g2_i4 | red | 0.9327855 |
| TRINITY_DN35295_c2_g1_i1 | red | 0.932775 |
| TRINITY_DN24609_c0_g2_i1 | red | 0.9326641 |
| TRINITY_DN68012_c0_g1_i1 | red | 0.9321353 |
| TRINITY_DN30826_c0_g1_i1 | red | 0.9308831 |
| TRINITY_DN32963_c0_g2_i1 | red | 0.9307343 |
| TRINITY_DN30057_c0_g1_i1 | red | 0.9307198 |
| TRINITY_DN33409_c1_g16_i1 | red | 0.9302245 |
| TRINITY_DN27195_c0_g1_i2 | red | 0.9293008 |
| TRINITY_DN36005_c0_g1_i4 | red | 0.9291499 |
| TRINITY_DN33736_c1_g4_i1 | red | 0.9290966 |
| TRINITY_DN37636_c0_g2_i1 | red | 0.9288618 |
| TRINITY_DN24855_c0_g1_i2 | red | 0.9288116 |
| TRINITY_DN38279_c0_g7_i1 | red | 0.9280412 |
| TRINITY_DN39205_c1_g1_i1 | red | 0.9280003 |
| TRINITY_DN22771_c1_g1_i1 | red | 0.9274651 |
| TRINITY_DN25753_c0_g1_i3 | red | 0.9271901 |
| TRINITY_DN39314_c5_g6_i18 | red | 0.9271749 |
| TRINITY_DN27716_c0_g1_i1 | red | 0.9270526 |
| TRINITY_DN38884_c0_g4_i1 | red | 0.9270506 |
| TRINITY_DN16158_c0_g1_i1 | red | 0.9267465 |
| TRINITY_DN26892_c0_g4_i1 | red | 0.9266755 |
| TRINITY_DN32067_c0_g4_i1 | red | 0.9262058 |
| TRINITY_DN30011_c4_g3_i1 | red | 0.9261823 |
| TRINITY_DN26632_c0_g3_i2 | red | 0.9257314 |
| TRINITY_DN36624_c0_g3_i1 | red | 0.9256927 |
| TRINITY_DN38554_c0_g1_i6 | red | 0.9255085 |
| TRINITY_DN38810_c0_g3_i1 | red | 0.92524 |
| TRINITY_DN2361_c0_g1_i1 | red | 0.9249838 |
| TRINITY_DN39088_c3_g3_i4 | red | 0.9247193 |
| TRINITY_DN39144_c2_g22_i1 | red | 0.9247166 |
| TRINITY_DN25643_c0_g2_i1 | red | 0.9246637 |
| TRINITY_DN24570_c0_g2_i1 | red | 0.9244831 |
| TRINITY_DN39251_c0_g5_i3 | red | 0.9243024 |
| TRINITY_DN35387_c2_g19_i1 | red | 0.9242578 |
| TRINITY_DN38233_c0_g4_i2 | red | 0.9239644 |
| TRINITY_DN33897_c0_g6_i1 | red | 0.9238266 |
| TRINITY_DN38690_c0_g5_i1 | red | 0.9235265 |
| TRINITY_DN971_c0_g1_i1 | red | 0.9234266 |
| TRINITY_DN22775_c0_g1_i1 | red | 0.9231647 |
| TRINITY_DN39504_c0_g1_i1 | red | 0.9222388 |
| TRINITY_DN35349_c0_g4_i6 | red | 0.9221981 |
| TRINITY_DN18291_c0_g1_i1 | red | 0.9220786 |
| TRINITY_DN16274_c0_g1_i1 | red | 0.9216169 |
| TRINITY_DN2624_c0_g1_i1 | red | 0.9210841 |
| TRINITY_DN30035_c0_g1_i1 | red | 0.9208753 |
| TRINITY_DN30360_c0_g1_i1 | red | 0.9207078 |
| TRINITY_DN25553_c0_g1_i1 | red | 0.9206512 |
| TRINITY_DN37705_c3_g2_i1 | red | 0.9202394 |
| TRINITY_DN39276_c2_g4_i1 | red | 0.9201328 |
| TRINITY_DN39286_c4_g3_i1 | red | 0.9197319 |
| TRINITY_DN32615_c0_g4_i1 | red | 0.9196191 |
| TRINITY_DN22806_c0_g2_i1 | red | 0.919363 |
| TRINITY_DN29835_c0_g5_i1 | red | 0.9191844 |
| TRINITY_DN25641_c0_g2_i1 | red | 0.9189384 |
| TRINITY_DN33860_c0_g1_i1 | red | 0.9188882 |
| TRINITY_DN17753_c0_g1_i1 | red | 0.9187817 |
| TRINITY_DN24242_c0_g1_i1 | red | 0.9182734 |
| TRINITY_DN30848_c0_g1_i1 | red | 0.9181424 |
| TRINITY_DN36082_c0_g1_i1 | red | 0.9177572 |
| TRINITY_DN28585_c0_g2_i1 | red | 0.917331 |
| TRINITY_DN25416_c0_g1_i1 | red | 0.9168269 |
| TRINITY_DN33147_c0_g3_i2 | red | 0.9164017 |
| TRINITY_DN33434_c1_g3_i2 | red | 0.9162586 |
| TRINITY_DN33851_c0_g4_i2 | red | 0.9153439 |
| TRINITY_DN36298_c0_g1_i1 | red | 0.9150507 |
| TRINITY_DN17433_c0_g3_i1 | red | 0.9147636 |
| TRINITY_DN32092_c0_g1_i8 | red | 0.9146029 |
| TRINITY_DN38557_c1_g1_i1 | red | 0.914509 |
| TRINITY_DN39055_c2_g4_i1 | red | 0.9139394 |
| TRINITY_DN20286_c0_g1_i2 | red | 0.9132415 |
| TRINITY_DN26710_c0_g1_i1 | red | 0.9125883 |
| TRINITY_DN45272_c0_g3_i1 | red | 0.9124528 |
| TRINITY_DN26881_c0_g4_i1 | red | 0.9122122 |
| TRINITY_DN24153_c0_g1_i2 | red | 0.9122076 |
| TRINITY_DN38344_c0_g1_i2 | red | 0.9121162 |
| TRINITY_DN39068_c0_g1_i1 | red | 0.9119189 |
| TRINITY_DN30975_c0_g1_i1 | red | 0.9117234 |
| TRINITY_DN32296_c0_g3_i12 | red | 0.9111384 |
| TRINITY_DN34813_c0_g5_i1 | red | 0.9108203 |
| TRINITY_DN32963_c0_g3_i1 | red | 0.9107274 |
| TRINITY_DN16576_c0_g2_i1 | red | 0.910567 |
| TRINITY_DN38936_c0_g4_i1 | red | 0.9105457 |
| TRINITY_DN30086_c0_g1_i1 | red | 0.9099806 |
| TRINITY_DN36755_c0_g2_i1 | red | 0.9094279 |
| TRINITY_DN36941_c0_g3_i1 | red | 0.90902 |
| TRINITY_DN26143_c0_g2_i1 | red | 0.908535 |
| TRINITY_DN29696_c0_g1_i4 | red | 0.9084807 |
| TRINITY_DN25643_c0_g5_i1 | red | 0.9078387 |
| TRINITY_DN38442_c7_g4_i1 | red | 0.9077597 |
| TRINITY_DN39127_c1_g8_i1 | red | 0.9074396 |
| TRINITY_DN22932_c0_g1_i5 | red | 0.9065067 |
| TRINITY_DN30843_c0_g7_i1 | red | 0.905971 |
| TRINITY_DN31950_c0_g12_i1 | red | 0.9059545 |
| TRINITY_DN33851_c0_g1_i1 | red | 0.905884 |
| TRINITY_DN35420_c0_g1_i2 | red | 0.9057144 |
| TRINITY_DN39328_c8_g2_i3 | red | 0.90559 |
| TRINITY_DN24992_c0_g1_i2 | red | 0.9055274 |
| TRINITY_DN31231_c0_g1_i2 | red | 0.905431 |
| TRINITY_DN22997_c0_g1_i1 | red | 0.9051126 |
| TRINITY_DN25398_c0_g2_i1 | red | 0.9049802 |
| TRINITY_DN8426_c0_g1_i1 | red | 0.9049793 |
| TRINITY_DN65136_c0_g1_i1 | red | 0.9039574 |
| TRINITY_DN37436_c0_g1_i4 | red | 0.9034644 |
| TRINITY_DN38557_c1_g8_i1 | red | 0.9033987 |
| TRINITY_DN36811_c1_g7_i1 | red | 0.9031809 |
| TRINITY_DN17257_c0_g2_i1 | red | 0.9029962 |
| TRINITY_DN26085_c0_g1_i9 | red | 0.9027353 |
| TRINITY_DN27296_c0_g1_i1 | red | 0.901921 |
| TRINITY_DN30074_c1_g10_i1 | red | 0.9017886 |
| TRINITY_DN18967_c0_g1_i1 | red | 0.9014808 |
| TRINITY_DN39080_c0_g3_i2 | red | 0.9012934 |
| TRINITY_DN16222_c0_g3_i1 | red | 0.901253 |
| TRINITY_DN789_c0_g2_i1 | red | 0.9010194 |
| TRINITY_DN35130_c1_g7_i2 | red | 0.9006283 |
| TRINITY_DN34149_c0_g4_i1 | red | 0.9004978 |
| TRINITY_DN21213_c0_g1_i1 | red | 0.9002068 |
| TRINITY_DN39068_c0_g8_i1 | red | 0.9001562 |
| TRINITY_DN33946_c0_g3_i1 | red | 0.9000366 |
| TRINITY_DN63773_c0_g1_i1 | red | 0.8999385 |
| TRINITY_DN24748_c0_g1_i1 | red | 0.8998589 |
| TRINITY_DN36285_c0_g3_i3 | red | 0.8996619 |
| TRINITY_DN20416_c0_g1_i1 | red | 0.8994598 |
| TRINITY_DN36060_c0_g4_i1 | red | 0.8993034 |
| TRINITY_DN44958_c0_g1_i1 | red | 0.8986418 |
| TRINITY_DN36952_c0_g1_i1 | red | 0.898293 |
| TRINITY_DN38170_c0_g1_i1 | red | 0.8981801 |
| TRINITY_DN29314_c1_g1_i3 | red | 0.8977984 |
| TRINITY_DN29059_c0_g1_i2 | red | 0.8974292 |
| TRINITY_DN25850_c0_g2_i1 | red | 0.8973786 |
| TRINITY_DN30624_c0_g1_i4 | red | 0.8973078 |
| TRINITY_DN26710_c0_g2_i1 | red | 0.8972437 |
| TRINITY_DN39062_c1_g5_i1 | red | 0.8968599 |
| TRINITY_DN29124_c0_g1_i2 | red | 0.896765 |
| TRINITY_DN16872_c0_g1_i1 | red | 0.8966507 |
| TRINITY_DN38985_c1_g11_i1 | red | 0.896605 |
| TRINITY_DN32831_c0_g8_i3 | red | 0.8965342 |
| TRINITY_DN31614_c0_g1_i2 | red | 0.8960491 |
| TRINITY_DN37996_c0_g4_i1 | red | 0.8950541 |
| TRINITY_DN29336_c1_g1_i5 | red | 0.8945958 |
| TRINITY_DN35698_c0_g2_i1 | red | 0.893957 |
| TRINITY_DN16569_c0_g2_i1 | red | 0.8938951 |
| TRINITY_DN25590_c0_g4_i1 | red | 0.8936314 |
| TRINITY_DN22238_c0_g1_i1 | red | 0.8935931 |
| TRINITY_DN23354_c1_g1_i1 | red | 0.8932002 |
| TRINITY_DN22966_c0_g1_i2 | red | 0.8922161 |
| TRINITY_DN39276_c2_g11_i1 | red | 0.8919096 |
| TRINITY_DN31190_c0_g2_i3 | red | 0.8911567 |
| TRINITY_DN36771_c0_g2_i2 | red | 0.8911346 |
| TRINITY_DN38483_c1_g6_i1 | red | 0.8904966 |
| TRINITY_DN36392_c0_g5_i2 | red | 0.8904794 |
| TRINITY_DN35530_c1_g3_i1 | red | 0.890143 |
| TRINITY_DN13733_c0_g1_i1 | red | 0.8899444 |
| TRINITY_DN14126_c0_g1_i1 | red | 0.8896359 |
| TRINITY_DN5538_c0_g2_i1 | red | 0.889219 |
| TRINITY_DN32522_c1_g1_i1 | red | 0.88863 |
| TRINITY_DN19237_c0_g1_i3 | red | 0.8878564 |
| TRINITY_DN18931_c0_g2_i1 | red | 0.8876827 |
| TRINITY_DN38840_c0_g2_i1 | red | 0.8869609 |
| TRINITY_DN21681_c0_g1_i1 | red | 0.8866936 |
| TRINITY_DN34645_c0_g1_i12 | red | 0.8858521 |
| TRINITY_DN36016_c1_g4_i4 | red | 0.885606 |
| TRINITY_DN27344_c0_g1_i1 | red | 0.885422 |
| TRINITY_DN39002_c0_g8_i1 | red | 0.8853436 |
| TRINITY_DN36754_c0_g6_i1 | red | 0.8848159 |
| TRINITY_DN39060_c0_g13_i1 | red | 0.8847514 |
| TRINITY_DN39340_c21_g1_i3 | red | 0.8846658 |
| TRINITY_DN22697_c0_g1_i1 | red | 0.8844352 |
| TRINITY_DN37294_c0_g1_i5 | red | 0.8838294 |
| TRINITY_DN33352_c0_g1_i2 | red | 0.8831274 |
| TRINITY_DN33302_c1_g1_i1 | red | 0.8830471 |
| TRINITY_DN34386_c2_g4_i1 | red | 0.8822471 |
| TRINITY_DN17050_c0_g1_i1 | red | 0.8821612 |
| TRINITY_DN29905_c0_g2_i1 | red | 0.881344 |
| TRINITY_DN28066_c0_g1_i1 | red | 0.8813396 |
| TRINITY_DN35302_c1_g1_i1 | red | 0.8810957 |
| TRINITY_DN21810_c0_g1_i1 | red | 0.8810714 |
| TRINITY_DN39065_c6_g5_i1 | red | 0.8807464 |
| TRINITY_DN24367_c0_g1_i1 | red | 0.8805991 |
| TRINITY_DN8875_c0_g1_i1 | red | 0.8801201 |
| TRINITY_DN36384_c0_g7_i1 | red | 0.8796729 |
| TRINITY_DN32751_c1_g1_i2 | red | 0.8794361 |
| TRINITY_DN29391_c1_g1_i1 | red | 0.878594 |
| TRINITY_DN39343_c18_g22_i1 | red | 0.878518 |
| TRINITY_DN19173_c0_g2_i1 | red | 0.8785065 |
| TRINITY_DN17744_c0_g2_i1 | red | 0.8783503 |
| TRINITY_DN32265_c1_g4_i1 | red | 0.8780957 |
| TRINITY_DN22127_c0_g1_i1 | red | 0.8772494 |
| TRINITY_DN38442_c6_g6_i1 | red | 0.8768995 |
| TRINITY_DN33031_c0_g6_i1 | red | 0.876805 |
| TRINITY_DN29414_c0_g2_i1 | red | 0.8766667 |
| TRINITY_DN10677_c0_g1_i1 | red | 0.8762122 |
| TRINITY_DN39016_c3_g3_i1 | red | 0.8758483 |
| TRINITY_DN26783_c0_g2_i2 | red | 0.8758464 |
| TRINITY_DN37846_c0_g1_i2 | red | 0.8756641 |
| TRINITY_DN27521_c0_g1_i3 | red | 0.8754404 |
| TRINITY_DN38487_c0_g4_i1 | red | 0.8751417 |
| TRINITY_DN63564_c0_g1_i1 | red | 0.8751386 |
| TRINITY_DN35352_c1_g1_i3 | red | 0.875083 |
| TRINITY_DN39135_c0_g2_i1 | red | 0.8746311 |
| TRINITY_DN38170_c0_g2_i9 | red | 0.8741776 |
| TRINITY_DN39336_c14_g1_i5 | red | 0.8741169 |
| TRINITY_DN25733_c0_g1_i1 | red | 0.8737079 |
| TRINITY_DN20125_c0_g2_i1 | red | 0.8734921 |
| TRINITY_DN22187_c0_g1_i1 | red | 0.8732582 |
| TRINITY_DN26628_c0_g1_i2 | red | 0.8723405 |
| TRINITY_DN28527_c0_g4_i1 | red | 0.8721057 |
| TRINITY_DN38079_c0_g4_i1 | red | 0.871764 |
| TRINITY_DN21214_c0_g2_i2 | red | 0.8710381 |
| TRINITY_DN30717_c0_g1_i2 | red | 0.8704911 |
| TRINITY_DN11859_c0_g1_i1 | red | 0.8704885 |
| TRINITY_DN37520_c2_g2_i1 | red | 0.8696426 |
| TRINITY_DN29059_c0_g2_i1 | red | 0.8693441 |
| TRINITY_DN29935_c0_g2_i2 | red | 0.8690731 |
| TRINITY_DN34941_c0_g2_i1 | red | 0.8685554 |
| TRINITY_DN32067_c0_g3_i2 | red | 0.868246 |
| TRINITY_DN34896_c0_g6_i1 | red | 0.8680469 |
| TRINITY_DN24658_c0_g2_i1 | red | 0.867933 |
| TRINITY_DN33182_c1_g1_i1 | red | 0.8675979 |
| TRINITY_DN24929_c0_g1_i1 | red | 0.8674914 |
| TRINITY_DN38024_c0_g1_i3 | red | 0.8670214 |
| TRINITY_DN38795_c1_g2_i1 | red | 0.8667309 |
| TRINITY_DN31462_c0_g5_i1 | red | 0.8667052 |
| TRINITY_DN39880_c0_g1_i1 | red | 0.8664986 |
| TRINITY_DN17681_c0_g3_i1 | red | 0.8657842 |
| TRINITY_DN23043_c0_g5_i1 | red | 0.8655282 |
| TRINITY_DN22476_c0_g1_i1 | red | 0.8654385 |
| TRINITY_DN38164_c0_g2_i1 | red | 0.8642434 |
| TRINITY_DN32686_c0_g1_i25 | red | 0.8642281 |
| TRINITY_DN27291_c0_g1_i4 | red | 0.8638976 |
| TRINITY_DN30098_c0_g1_i1 | red | 0.8638779 |
| TRINITY_DN29100_c0_g1_i1 | red | 0.8635834 |
| TRINITY_DN38607_c1_g10_i3 | red | 0.863322 |
| TRINITY_DN24968_c0_g1_i1 | red | 0.8629365 |
| TRINITY_DN39248_c2_g1_i1 | red | 0.8625862 |
| TRINITY_DN26076_c1_g1_i1 | red | 0.8625569 |
| TRINITY_DN34392_c0_g1_i3 | red | 0.862523 |
| TRINITY_DN21620_c0_g2_i1 | red | 0.8616143 |
| TRINITY_DN35361_c0_g1_i2 | red | 0.8612036 |
| TRINITY_DN35137_c1_g1_i3 | red | 0.8605534 |
| TRINITY_DN28107_c0_g1_i2 | red | 0.8603773 |
| TRINITY_DN34071_c2_g4_i10 | red | 0.859672 |
| TRINITY_DN16583_c1_g1_i1 | red | 0.8578848 |
| TRINITY_DN34778_c0_g2_i1 | red | 0.8574323 |
| TRINITY_DN52338_c0_g1_i1 | red | 0.856682 |
| TRINITY_DN2361_c0_g2_i1 | red | 0.8564064 |
| TRINITY_DN7774_c0_g2_i1 | red | 0.8563736 |
| TRINITY_DN20616_c0_g2_i1 | red | 0.8559872 |
| TRINITY_DN24669_c0_g1_i1 | red | 0.8559242 |
| TRINITY_DN39159_c0_g2_i1 | red | 0.8555686 |
| TRINITY_DN38233_c0_g3_i8 | red | 0.8527945 |
| TRINITY_DN20524_c0_g2_i1 | red | 0.8525565 |
| TRINITY_DN35984_c1_g10_i4 | red | 0.8524944 |
| TRINITY_DN38445_c3_g1_i2 | red | 0.8514094 |
| TRINITY_DN16225_c0_g2_i1 | red | 0.8513277 |
| TRINITY_DN20575_c0_g1_i1 | red | 0.8512653 |
| TRINITY_DN22646_c0_g2_i1 | red | 0.8510967 |
| TRINITY_DN38706_c0_g4_i12 | red | 0.8506968 |
| TRINITY_DN6430_c0_g2_i1 | red | 0.8502139 |
| TRINITY_DN39341_c9_g18_i2 | red | 0.8491974 |
| TRINITY_DN37459_c0_g1_i1 | red | 0.847417 |
| TRINITY_DN26416_c0_g1_i1 | red | 0.8467212 |
| TRINITY_DN36322_c0_g1_i4 | red | 0.8467146 |
| TRINITY_DN27377_c0_g1_i1 | red | 0.8459404 |
| TRINITY_DN35987_c0_g3_i1 | red | 0.8456583 |
| TRINITY_DN37317_c1_g2_i1 | red | 0.8452921 |
| TRINITY_DN25791_c0_g2_i1 | red | 0.8444417 |
| TRINITY_DN39062_c3_g6_i1 | red | 0.8443802 |
| TRINITY_DN39343_c19_g17_i1 | red | 0.8440633 |
| TRINITY_DN67600_c0_g3_i1 | red | 0.8430048 |
| TRINITY_DN19905_c0_g2_i1 | red | 0.8429043 |
| TRINITY_DN35387_c2_g14_i5 | red | 0.841542 |
| TRINITY_DN21690_c0_g1_i1 | red | 0.8409744 |
| TRINITY_DN30111_c0_g1_i1 | red | 0.8406101 |
| TRINITY_DN31773_c3_g5_i1 | red | 0.8403455 |
| TRINITY_DN39121_c0_g3_i4 | red | 0.8388928 |
| TRINITY_DN37191_c0_g3_i3 | red | 0.8355432 |
| TRINITY_DN32577_c0_g11_i1 | red | 0.8351572 |
| TRINITY_DN30294_c0_g2_i2 | red | 0.8350338 |
| TRINITY_DN37736_c1_g5_i3 | red | 0.8343158 |
| TRINITY_DN25990_c1_g1_i2 | red | 0.8335579 |
| TRINITY_DN59433_c0_g1_i1 | red | 0.8334945 |
| TRINITY_DN18258_c0_g1_i1 | red | 0.8318657 |
| TRINITY_DN25643_c0_g6_i1 | red | 0.8316691 |
| TRINITY_DN35463_c1_g6_i1 | red | 0.8315512 |
| TRINITY_DN30053_c0_g1_i5 | red | 0.8304273 |
| TRINITY_DN34582_c0_g1_i6 | red | 0.8302465 |
| TRINITY_DN36941_c0_g4_i2 | red | 0.8291021 |
| TRINITY_DN36981_c0_g3_i1 | red | 0.8289509 |
| TRINITY_DN53097_c0_g1_i1 | red | 0.8285293 |
| TRINITY_DN22499_c0_g1_i4 | red | 0.828442 |
| TRINITY_DN8408_c0_g1_i1 | red | 0.8281727 |
| TRINITY_DN38363_c0_g2_i3 | red | 0.8250603 |
| TRINITY_DN37713_c0_g1_i2 | red | 0.8246628 |
| TRINITY_DN34661_c0_g1_i3 | red | 0.8238009 |
| TRINITY_DN13716_c0_g1_i1 | red | 0.8234715 |
| TRINITY_DN35185_c0_g4_i1 | red | 0.8213707 |
| TRINITY_DN30853_c0_g2_i1 | red | 0.8212001 |
| TRINITY_DN39023_c0_g8_i1 | red | 0.821107 |
| TRINITY_DN30853_c0_g1_i1 | red | 0.8197108 |
| TRINITY_DN25090_c0_g3_i1 | red | 0.8189298 |
| TRINITY_DN21638_c0_g1_i1 | red | 0.8179427 |
| TRINITY_DN34304_c0_g9_i1 | red | 0.8167264 |
| TRINITY_DN35185_c0_g2_i1 | red | 0.8157294 |
| TRINITY_DN65444_c0_g1_i1 | red | 0.8145188 |
| TRINITY_DN19527_c0_g1_i3 | red | 0.8142293 |
| TRINITY_DN21662_c0_g3_i1 | red | 0.8136649 |
| TRINITY_DN36811_c1_g10_i1 | red | 0.8135313 |
| TRINITY_DN21914_c0_g1_i1 | red | 0.8131763 |
| TRINITY_DN38653_c0_g6_i1 | red | 0.8111612 |
| TRINITY_DN30631_c0_g1_i1 | red | 0.8105753 |
| TRINITY_DN34161_c0_g2_i5 | red | 0.8101299 |
| TRINITY_DN27212_c0_g2_i2 | red | 0.8101033 |
| TRINITY_DN28799_c0_g1_i2 | red | 0.8094793 |
| TRINITY_DN30062_c0_g1_i2 | red | 0.8090771 |
| TRINITY_DN25802_c0_g1_i1 | red | 0.8083995 |
| TRINITY_DN36465_c0_g3_i1 | red | 0.808364 |
| TRINITY_DN29451_c0_g1_i3 | red | 0.8056487 |
| TRINITY_DN30945_c0_g1_i1 | red | 0.8040511 |
| TRINITY_DN25791_c0_g1_i1 | red | 0.8019743 |
| TRINITY_DN29451_c0_g2_i1 | red | 0.8019684 |
| TRINITY_DN70786_c0_g1_i1 | red | 0.8017197 |
| TRINITY_DN38147_c0_g3_i7 | red | 0.8012739 |
| TRINITY_DN36316_c0_g1_i9 | red | 0.7986059 |
| TRINITY_DN16380_c0_g1_i1 | red | 0.7950255 |
| TRINITY_DN27624_c0_g1_i1 | red | 0.7934512 |
| TRINITY_DN33920_c1_g2_i3 | red | 0.7900155 |
| TRINITY_DN37254_c1_g3_i1 | red | 0.7892243 |
| TRINITY_DN52222_c0_g1_i1 | red | 0.7871837 |
| TRINITY_DN21903_c0_g2_i1 | red | 0.7869084 |
| TRINITY_DN39180_c7_g15_i1 | red | 0.7848186 |
| TRINITY_DN24852_c0_g6_i1 | red | 0.7834234 |
| TRINITY_DN68495_c0_g1_i1 | red | 0.7833882 |
| TRINITY_DN38621_c2_g1_i4 | red | 0.7823879 |
| TRINITY_DN38888_c2_g19_i1 | red | 0.7770758 |
| TRINITY_DN24262_c0_g1_i1 | red | 0.7757792 |
| TRINITY_DN39068_c0_g22_i1 | red | 0.7750211 |
| TRINITY_DN34912_c0_g1_i5 | red | 0.7749131 |
| TRINITY_DN24625_c0_g2_i1 | red | 0.7730038 |
| TRINITY_DN28430_c0_g2_i1 | red | 0.7716233 |
| TRINITY_DN33107_c0_g2_i1 | red | 0.7680614 |
| TRINITY_DN33144_c0_g1_i6 | red | 0.7659208 |
| TRINITY_DN27006_c0_g2_i1 | red | 0.7650808 |
| TRINITY_DN33944_c2_g1_i9 | red | 0.7645299 |
| TRINITY_DN21720_c0_g2_i1 | red | 0.7637283 |
| TRINITY_DN35964_c0_g8_i1 | red | 0.7601801 |
| TRINITY_DN4780_c0_g2_i1 | red | 0.760154 |
| TRINITY_DN34719_c0_g1_i8 | red | 0.7599792 |
| TRINITY_DN31956_c1_g1_i1 | red | 0.7587167 |
| TRINITY_DN26627_c1_g1_i2 | red | 0.7575219 |
| TRINITY_DN17319_c0_g1_i1 | red | 0.7534212 |
| TRINITY_DN28048_c0_g1_i1 | red | 0.7519186 |
| TRINITY_DN27951_c0_g2_i1 | red | 0.7501997 |
| TRINITY_DN30494_c0_g6_i1 | red | 0.7499656 |
| TRINITY_DN28856_c0_g2_i1 | red | 0.7475128 |
| TRINITY_DN29768_c0_g1_i3 | red | 0.7462752 |
| TRINITY_DN36082_c0_g12_i1 | red | 0.7459027 |
| TRINITY_DN22908_c0_g3_i1 | red | 0.7452986 |
| TRINITY_DN37439_c1_g2_i1 | red | 0.7434005 |
| TRINITY_DN18925_c0_g1_i1 | red | 0.7428973 |
| TRINITY_DN37973_c1_g1_i4 | red | 0.7368067 |
| TRINITY_DN38295_c3_g11_i1 | red | 0.7366043 |
| TRINITY_DN24430_c0_g5_i1 | red | 0.7338881 |
| TRINITY_DN38632_c0_g6_i1 | red | 0.7255606 |
| TRINITY_DN376_c0_g1_i1 | red | 0.7250017 |
| TRINITY_DN24175_c0_g2_i2 | red | 0.7243159 |
| TRINITY_DN35268_c1_g2_i2 | red | 0.7233774 |
| TRINITY_DN4655_c0_g1_i1 | red | 0.7213826 |
| TRINITY_DN26922_c0_g2_i1 | red | 0.7201812 |
| TRINITY_DN26196_c1_g2_i1 | red | 0.7147873 |
| TRINITY_DN34995_c0_g3_i1 | red | 0.7115922 |
| TRINITY_DN37071_c0_g4_i2 | red | 0.7103209 |
| TRINITY_DN38135_c2_g2_i1 | red | 0.7029264 |
| TRINITY_DN18035_c0_g1_i1 | red | 0.7015116 |
| TRINITY_DN31388_c1_g1_i1 | red | 0.6998694 |
| TRINITY_DN13632_c0_g1_i1 | red | 0.6987306 |
| TRINITY_DN39329_c14_g10_i1 | red | 0.6918918 |
| TRINITY_DN28582_c0_g1_i3 | red | 0.6894719 |
| TRINITY_DN28479_c0_g1_i3 | red | 0.6889841 |
| TRINITY_DN37339_c0_g2_i2 | red | 0.6808149 |
| TRINITY_DN26246_c0_g1_i1 | red | 0.6799143 |
| TRINITY_DN14051_c0_g1_i1 | red | 0.6796115 |
| TRINITY_DN21058_c0_g4_i1 | red | 0.6732924 |
| TRINITY_DN20097_c0_g1_i2 | red | 0.6731695 |
| TRINITY_DN16148_c0_g1_i1 | red | 0.6713652 |
| TRINITY_DN24953_c0_g3_i1 | red | 0.6686949 |
| TRINITY_DN50696_c0_g1_i1 | red | 0.6670356 |
| TRINITY_DN30352_c1_g9_i1 | red | 0.6644014 |
| TRINITY_DN17842_c0_g2_i1 | red | 0.6578073 |
| TRINITY_DN56952_c0_g1_i1 | red | 0.6565665 |
| TRINITY_DN33090_c1_g2_i1 | red | 0.6385921 |
| TRINITY_DN23986_c0_g2_i1 | red | 0.6342696 |
| TRINITY_DN17097_c0_g1_i1 | red | 0.6310834 |
| TRINITY_DN17828_c0_g1_i2 | red | 0.621444 |
| TRINITY_DN8805_c0_g1_i1 | red | 0.61053 |
| TRINITY_DN26783_c0_g4_i1 | red | 0.5789717 |
| TRINITY_DN9891_c0_g2_i1 | red | 0.572556 |
| TRINITY_DN31726_c0_g6_i1 | red | 0.5620819 |
| TRINITY_DN21363_c0_g2_i1 | red | 0.4482315 |
| TRINITY_DN32179_c0_g2_i3 | red | -0.548937 |
| TRINITY_DN34050_c0_g1_i2 | red | -0.569228 |
| TRINITY_DN5813_c0_g1_i1 | red | -0.589251 |
| TRINITY_DN33812_c0_g3_i1 | red | -0.602842 |
| TRINITY_DN34570_c0_g2_i1 | red | -0.613903 |
| TRINITY_DN21831_c0_g2_i1 | red | -0.637845 |
| TRINITY_DN37416_c0_g1_i1 | red | -0.645535 |
| TRINITY_DN26967_c0_g1_i3 | red | -0.650253 |
| TRINITY_DN20829_c0_g1_i1 | red | -0.655519 |
| TRINITY_DN25701_c0_g1_i1 | red | -0.655563 |
| TRINITY_DN25432_c0_g4_i1 | red | -0.66084 |
| TRINITY_DN38487_c0_g3_i1 | red | -0.671833 |
| TRINITY_DN26905_c0_g1_i1 | red | -0.681569 |
| TRINITY_DN31545_c1_g5_i1 | red | -0.688325 |
| TRINITY_DN34273_c0_g2_i2 | red | -0.69316 |
| TRINITY_DN34900_c0_g1_i3 | red | -0.697329 |
| TRINITY_DN23927_c0_g1_i1 | red | -0.706736 |
| TRINITY_DN51735_c0_g1_i1 | red | -0.714739 |
| TRINITY_DN32603_c0_g5_i2 | red | -0.716921 |
| TRINITY_DN39334_c7_g9_i1 | red | -0.717725 |
| TRINITY_DN44998_c0_g1_i1 | red | -0.721106 |
| TRINITY_DN34156_c0_g1_i9 | red | -0.726068 |
| TRINITY_DN32882_c0_g3_i2 | red | -0.726695 |
| TRINITY_DN872_c0_g1_i1 | red | -0.732996 |
| TRINITY_DN32973_c0_g2_i4 | red | -0.733326 |
| TRINITY_DN27750_c0_g3_i2 | red | -0.734533 |
| TRINITY_DN22749_c0_g1_i1 | red | -0.73904 |
| TRINITY_DN24472_c0_g1_i1 | red | -0.741515 |
| TRINITY_DN36132_c0_g1_i3 | red | -0.741938 |
| TRINITY_DN62946_c0_g1_i1 | red | -0.744848 |
| TRINITY_DN34096_c0_g2_i1 | red | -0.756001 |
| TRINITY_DN34028_c0_g1_i3 | red | -0.756697 |
| TRINITY_DN36163_c1_g1_i24 | red | -0.760607 |
| TRINITY_DN22822_c0_g1_i1 | red | -0.761297 |
| TRINITY_DN35862_c0_g7_i3 | red | -0.764885 |
| TRINITY_DN36709_c0_g2_i6 | red | -0.768227 |
| TRINITY_DN38158_c0_g2_i1 | red | -0.7724 |
| TRINITY_DN25437_c0_g1_i2 | red | -0.772882 |
| TRINITY_DN25491_c0_g1_i2 | red | -0.773727 |
| TRINITY_DN38907_c2_g2_i1 | red | -0.774031 |
| TRINITY_DN17433_c0_g2_i1 | red | -0.77428 |
| TRINITY_DN22082_c0_g1_i1 | red | -0.77643 |
| TRINITY_DN28860_c0_g3_i1 | red | -0.778011 |
| TRINITY_DN30477_c0_g1_i1 | red | -0.778915 |
| TRINITY_DN36811_c1_g1_i1 | red | -0.779013 |
| TRINITY_DN30928_c0_g1_i1 | red | -0.780036 |
| TRINITY_DN31812_c0_g1_i8 | red | -0.781037 |
| TRINITY_DN68158_c0_g1_i1 | red | -0.78245 |
| TRINITY_DN25656_c0_g1_i1 | red | -0.786066 |
| TRINITY_DN36765_c0_g2_i1 | red | -0.787716 |
| TRINITY_DN30916_c0_g1_i1 | red | -0.788133 |
| TRINITY_DN31821_c0_g2_i2 | red | -0.789644 |
| TRINITY_DN35783_c0_g1_i4 | red | -0.790722 |
| TRINITY_DN34049_c2_g10_i1 | red | -0.793195 |
| TRINITY_DN38693_c3_g2_i1 | red | -0.79542 |
| TRINITY_DN35611_c1_g1_i1 | red | -0.796853 |
| TRINITY_DN25436_c0_g2_i2 | red | -0.796889 |
| TRINITY_DN26608_c0_g1_i1 | red | -0.797956 |
| TRINITY_DN24915_c0_g1_i2 | red | -0.802197 |
| TRINITY_DN35731_c0_g2_i1 | red | -0.802844 |
| TRINITY_DN22311_c0_g1_i1 | red | -0.804336 |
| TRINITY_DN38205_c1_g1_i1 | red | -0.808271 |
| TRINITY_DN36812_c0_g5_i2 | red | -0.812826 |
| TRINITY_DN26566_c0_g1_i1 | red | -0.813105 |
| TRINITY_DN37009_c0_g2_i1 | red | -0.813992 |
| TRINITY_DN27672_c0_g1_i2 | red | -0.816703 |
| TRINITY_DN33924_c1_g1_i1 | red | -0.818809 |
| TRINITY_DN33857_c0_g1_i2 | red | -0.819715 |
| TRINITY_DN31094_c0_g15_i1 | red | -0.821287 |
| TRINITY_DN38240_c1_g5_i1 | red | -0.821499 |
| TRINITY_DN34615_c3_g1_i1 | red | -0.822016 |
| TRINITY_DN23552_c0_g2_i2 | red | -0.822668 |
| TRINITY_DN34251_c1_g3_i1 | red | -0.823232 |
| TRINITY_DN32047_c0_g11_i1 | red | -0.823782 |
| TRINITY_DN25982_c0_g1_i1 | red | -0.825256 |
| TRINITY_DN33290_c0_g5_i1 | red | -0.827535 |
| TRINITY_DN35237_c0_g1_i2 | red | -0.834789 |
| TRINITY_DN30753_c0_g2_i1 | red | -0.835418 |
| TRINITY_DN6844_c0_g1_i1 | red | -0.835518 |
| TRINITY_DN37710_c0_g2_i3 | red | -0.835764 |
| TRINITY_DN34592_c4_g4_i5 | red | -0.836333 |
| TRINITY_DN29666_c0_g1_i3 | red | -0.837372 |
| TRINITY_DN34527_c0_g1_i2 | red | -0.839168 |
| TRINITY_DN35214_c2_g2_i1 | red | -0.83934 |
| TRINITY_DN24058_c0_g1_i6 | red | -0.839605 |
| TRINITY_DN38988_c0_g1_i1 | red | -0.840019 |
| TRINITY_DN29672_c0_g1_i4 | red | -0.840149 |
| TRINITY_DN30755_c0_g1_i2 | red | -0.840627 |
| TRINITY_DN33715_c0_g1_i7 | red | -0.840881 |
| TRINITY_DN37006_c14_g7_i1 | red | -0.840931 |
| TRINITY_DN39131_c0_g1_i1 | red | -0.844218 |
| TRINITY_DN36065_c0_g3_i1 | red | -0.844325 |
| TRINITY_DN38200_c0_g1_i12 | red | -0.844908 |
| TRINITY_DN31934_c0_g1_i1 | red | -0.845641 |
| TRINITY_DN32760_c0_g2_i3 | red | -0.846539 |
| TRINITY_DN22254_c0_g1_i1 | red | -0.851969 |
| TRINITY_DN33450_c0_g2_i4 | red | -0.85352 |
| TRINITY_DN35468_c0_g8_i4 | red | -0.85356 |
| TRINITY_DN27995_c0_g1_i2 | red | -0.854545 |
| TRINITY_DN38711_c1_g1_i1 | red | -0.855351 |
| TRINITY_DN25389_c0_g1_i1 | red | -0.855423 |
| TRINITY_DN34361_c0_g1_i18 | red | -0.856403 |
| TRINITY_DN39069_c3_g20_i1 | red | -0.856607 |
| TRINITY_DN38788_c0_g1_i1 | red | -0.858012 |
| TRINITY_DN37824_c0_g4_i1 | red | -0.858201 |
| TRINITY_DN31761_c0_g3_i2 | red | -0.859911 |
| TRINITY_DN34637_c0_g1_i1 | red | -0.860051 |
| TRINITY_DN33959_c0_g1_i6 | red | -0.860608 |
| TRINITY_DN26412_c0_g4_i2 | red | -0.860802 |
| TRINITY_DN31202_c0_g2_i16 | red | -0.865041 |
| TRINITY_DN26604_c0_g1_i1 | red | -0.865648 |
| TRINITY_DN39122_c0_g7_i1 | red | -0.86862 |
| TRINITY_DN27697_c0_g2_i1 | red | -0.868707 |
| TRINITY_DN34650_c2_g1_i1 | red | -0.869085 |
| TRINITY_DN19374_c0_g1_i1 | red | -0.869776 |
| TRINITY_DN23813_c0_g1_i1 | red | -0.869903 |
| TRINITY_DN27954_c0_g2_i1 | red | -0.870823 |
| TRINITY_DN38974_c0_g4_i6 | red | -0.873735 |
| TRINITY_DN23034_c0_g1_i2 | red | -0.874339 |
| TRINITY_DN35611_c1_g3_i1 | red | -0.875955 |
| TRINITY_DN39058_c1_g2_i4 | red | -0.87603 |
| TRINITY_DN25640_c0_g1_i3 | red | -0.877686 |
| TRINITY_DN33620_c1_g2_i4 | red | -0.878868 |
| TRINITY_DN29816_c0_g1_i1 | red | -0.879887 |
| TRINITY_DN35814_c0_g2_i13 | red | -0.881473 |
| TRINITY_DN35680_c1_g1_i1 | red | -0.882237 |
| TRINITY_DN32276_c0_g1_i5 | red | -0.882331 |
| TRINITY_DN35318_c1_g2_i2 | red | -0.8834 |
| TRINITY_DN33784_c0_g1_i1 | red | -0.88374 |
| TRINITY_DN31606_c3_g3_i13 | red | -0.884535 |
| TRINITY_DN30854_c0_g2_i5 | red | -0.885116 |
| TRINITY_DN33396_c0_g4_i1 | red | -0.885366 |
| TRINITY_DN35782_c0_g2_i1 | red | -0.885491 |
| TRINITY_DN35444_c1_g2_i1 | red | -0.886002 |
| TRINITY_DN29158_c0_g1_i4 | red | -0.887712 |
| TRINITY_DN37508_c0_g1_i5 | red | -0.888009 |
| TRINITY_DN31268_c0_g2_i1 | red | -0.888135 |
| TRINITY_DN30892_c0_g2_i4 | red | -0.889119 |
| TRINITY_DN29749_c0_g1_i1 | red | -0.889149 |
| TRINITY_DN33659_c0_g6_i1 | red | -0.890373 |
| TRINITY_DN36812_c0_g4_i5 | red | -0.890475 |
| TRINITY_DN23657_c0_g3_i1 | red | -0.892291 |
| TRINITY_DN29699_c0_g5_i1 | red | -0.893249 |
| TRINITY_DN34994_c0_g1_i3 | red | -0.893836 |
| TRINITY_DN38425_c0_g3_i7 | red | -0.894657 |
| TRINITY_DN29871_c0_g1_i7 | red | -0.896131 |
| TRINITY_DN30944_c3_g1_i1 | red | -0.898495 |
| TRINITY_DN29376_c0_g2_i3 | red | -0.899855 |
| TRINITY_DN32424_c0_g1_i7 | red | -0.900794 |
| TRINITY_DN31725_c0_g1_i4 | red | -0.902322 |
| TRINITY_DN31554_c0_g1_i4 | red | -0.902888 |
| TRINITY_DN37005_c0_g4_i1 | red | -0.904209 |
| TRINITY_DN52026_c0_g1_i1 | red | -0.906014 |
| TRINITY_DN29991_c0_g1_i3 | red | -0.906839 |
| TRINITY_DN28977_c0_g5_i1 | red | -0.909185 |
| TRINITY_DN39241_c8_g12_i4 | red | -0.909926 |
| TRINITY_DN25917_c0_g1_i2 | red | -0.910587 |
| TRINITY_DN36513_c0_g1_i1 | red | -0.914258 |
| TRINITY_DN39238_c1_g7_i1 | red | -0.918834 |
| TRINITY_DN31252_c0_g1_i2 | red | -0.921602 |
| TRINITY_DN29674_c0_g1_i4 | red | -0.922224 |
| TRINITY_DN34035_c1_g3_i6 | red | -0.923782 |
| TRINITY_DN33419_c0_g2_i1 | red | -0.927727 |
| TRINITY_DN37891_c0_g1_i11 | red | -0.929345 |
| TRINITY_DN31063_c0_g3_i2 | red | -0.929356 |
| TRINITY_DN32490_c0_g2_i1 | red | -0.929803 |
| TRINITY_DN26589_c0_g1_i1 | red | -0.936105 |
| TRINITY_DN36812_c0_g1_i1 | red | -0.938968 |
| TRINITY_DN29332_c0_g1_i1 | red | -0.941966 |
| TRINITY_DN33935_c0_g1_i7 | red | -0.944239 |
| TRINITY_DN38846_c2_g3_i1 | red | -0.948471 |
| TRINITY_DN35191_c2_g1_i1 | red | -0.949572 |
| TRINITY_DN39207_c1_g2_i5 | red | -0.954592 |
| TRINITY_DN27123_c0_g1_i1 | red | -0.956932 |
| TRINITY_DN31566_c1_g1_i3 | red | -0.972466 |
| TRINITY_DN38048_c2_g8_i1 | red | -0.972492 |
| TRINITY_DN27619_c0_g1_i1 | turquoise | 0.993869 |
| TRINITY_DN33984_c0_g2_i16 | turquoise | 0.9926037 |
| TRINITY_DN38976_c0_g5_i13 | turquoise | 0.9917445 |
| TRINITY_DN36437_c0_g3_i6 | turquoise | 0.9913956 |
| TRINITY_DN38257_c1_g1_i5 | turquoise | 0.9912944 |
| TRINITY_DN38356_c0_g3_i1 | turquoise | 0.9898584 |
| TRINITY_DN39111_c3_g3_i2 | turquoise | 0.9896422 |
| TRINITY_DN56019_c0_g1_i1 | turquoise | 0.9884114 |
| TRINITY_DN27718_c3_g3_i1 | turquoise | 0.9879523 |
| TRINITY_DN35942_c0_g3_i13 | turquoise | 0.9879333 |
| TRINITY_DN35306_c0_g1_i15 | turquoise | 0.9872317 |
| TRINITY_DN68045_c0_g1_i1 | turquoise | 0.9871142 |
| TRINITY_DN33042_c1_g1_i1 | turquoise | 0.9864941 |
| TRINITY_DN37842_c1_g2_i8 | turquoise | 0.9860489 |
| TRINITY_DN37657_c0_g2_i4 | turquoise | 0.9855175 |
| TRINITY_DN38458_c1_g2_i4 | turquoise | 0.9854365 |
| TRINITY_DN37971_c1_g5_i1 | turquoise | 0.9849591 |
| TRINITY_DN33153_c0_g1_i3 | turquoise | 0.9848434 |
| TRINITY_DN29780_c0_g1_i1 | turquoise | 0.9845287 |
| TRINITY_DN38421_c0_g1_i14 | turquoise | 0.9842499 |
| TRINITY_DN38954_c1_g3_i2 | turquoise | 0.9842162 |
| TRINITY_DN35724_c1_g1_i17 | turquoise | 0.9840803 |
| TRINITY_DN8475_c0_g2_i1 | turquoise | 0.9836835 |
| TRINITY_DN36595_c1_g3_i1 | turquoise | 0.9834861 |
| TRINITY_DN34381_c0_g3_i1 | turquoise | 0.9833945 |
| TRINITY_DN37325_c2_g1_i4 | turquoise | 0.9833407 |
| TRINITY_DN32079_c0_g1_i4 | turquoise | 0.9830754 |
| TRINITY_DN37934_c0_g2_i8 | turquoise | 0.9829872 |
| TRINITY_DN45099_c0_g1_i1 | turquoise | 0.9829624 |
| TRINITY_DN50926_c0_g1_i1 | turquoise | 0.982502 |
| TRINITY_DN36717_c1_g1_i1 | turquoise | 0.9824811 |
| TRINITY_DN36760_c1_g1_i1 | turquoise | 0.982263 |
| TRINITY_DN32948_c0_g1_i1 | turquoise | 0.9822391 |
| TRINITY_DN35509_c0_g1_i9 | turquoise | 0.9822057 |
| TRINITY_DN33188_c0_g3_i4 | turquoise | 0.9821582 |
| TRINITY_DN31712_c0_g1_i18 | turquoise | 0.981183 |
| TRINITY_DN38718_c0_g7_i4 | turquoise | 0.9810861 |
| TRINITY_DN38675_c1_g8_i10 | turquoise | 0.9804276 |
| TRINITY_DN32851_c1_g1_i4 | turquoise | 0.9803188 |
| TRINITY_DN69438_c0_g1_i1 | turquoise | 0.9800353 |
| TRINITY_DN34409_c1_g3_i4 | turquoise | 0.9798877 |
| TRINITY_DN21497_c0_g1_i1 | turquoise | 0.979856 |
| TRINITY_DN35010_c1_g7_i1 | turquoise | 0.9797637 |
| TRINITY_DN37232_c0_g1_i1 | turquoise | 0.9796773 |
| TRINITY_DN35358_c0_g1_i8 | turquoise | 0.9795422 |
| TRINITY_DN35833_c0_g1_i24 | turquoise | 0.9794386 |
| TRINITY_DN36890_c1_g2_i1 | turquoise | 0.9790718 |
| TRINITY_DN37657_c0_g1_i4 | turquoise | 0.9790546 |
| TRINITY_DN25465_c0_g1_i1 | turquoise | 0.9788732 |
| TRINITY_DN33563_c0_g2_i10 | turquoise | 0.9788404 |
| TRINITY_DN28822_c1_g1_i2 | turquoise | 0.9787082 |
| TRINITY_DN38725_c1_g1_i1 | turquoise | 0.9785368 |
| TRINITY_DN38825_c0_g1_i6 | turquoise | 0.978181 |
| TRINITY_DN34543_c0_g2_i7 | turquoise | 0.9780138 |
| TRINITY_DN29971_c0_g4_i2 | turquoise | 0.9773101 |
| TRINITY_DN45613_c0_g1_i1 | turquoise | 0.977206 |
| TRINITY_DN34593_c0_g2_i11 | turquoise | 0.9770956 |
| TRINITY_DN38976_c0_g4_i1 | turquoise | 0.9768376 |
| TRINITY_DN26700_c0_g6_i1 | turquoise | 0.9767764 |
| TRINITY_DN30871_c0_g2_i1 | turquoise | 0.976164 |
| TRINITY_DN16467_c0_g1_i1 | turquoise | 0.9759012 |
| TRINITY_DN32588_c0_g2_i1 | turquoise | 0.9758102 |
| TRINITY_DN39264_c4_g5_i2 | turquoise | 0.9757969 |
| TRINITY_DN28196_c0_g1_i2 | turquoise | 0.9757223 |
| TRINITY_DN20774_c0_g2_i1 | turquoise | 0.9754745 |
| TRINITY_DN32457_c0_g1_i2 | turquoise | 0.9754162 |
| TRINITY_DN36979_c0_g2_i4 | turquoise | 0.9754044 |
| TRINITY_DN28415_c0_g2_i4 | turquoise | 0.9751481 |
| TRINITY_DN30740_c0_g2_i1 | turquoise | 0.9742888 |
| TRINITY_DN36145_c2_g3_i7 | turquoise | 0.974047 |
| TRINITY_DN27019_c2_g1_i1 | turquoise | 0.9739453 |
| TRINITY_DN36130_c0_g2_i8 | turquoise | 0.9738422 |
| TRINITY_DN35991_c0_g2_i3 | turquoise | 0.9737144 |
| TRINITY_DN26551_c0_g1_i1 | turquoise | 0.9735218 |
| TRINITY_DN39001_c0_g2_i14 | turquoise | 0.9734073 |
| TRINITY_DN38438_c0_g5_i13 | turquoise | 0.973336 |
| TRINITY_DN33687_c0_g3_i11 | turquoise | 0.9733346 |
| TRINITY_DN32166_c0_g1_i2 | turquoise | 0.9731514 |
| TRINITY_DN26882_c0_g1_i1 | turquoise | 0.9729126 |
| TRINITY_DN33281_c0_g1_i1 | turquoise | 0.9728206 |
| TRINITY_DN37839_c1_g1_i1 | turquoise | 0.9728108 |
| TRINITY_DN37717_c1_g2_i2 | turquoise | 0.972806 |
| TRINITY_DN39264_c4_g3_i1 | turquoise | 0.9727964 |
| TRINITY_DN33297_c0_g1_i7 | turquoise | 0.9727078 |
| TRINITY_DN34951_c0_g2_i7 | turquoise | 0.9725477 |
| TRINITY_DN31543_c0_g2_i6 | turquoise | 0.9723602 |
| TRINITY_DN36890_c1_g1_i3 | turquoise | 0.9722535 |
| TRINITY_DN36280_c0_g4_i6 | turquoise | 0.9722266 |
| TRINITY_DN27320_c0_g6_i10 | turquoise | 0.9721799 |
| TRINITY_DN38906_c2_g4_i1 | turquoise | 0.9720952 |
| TRINITY_DN24739_c0_g2_i1 | turquoise | 0.9720529 |
| TRINITY_DN50728_c0_g1_i1 | turquoise | 0.9719101 |
| TRINITY_DN37298_c0_g3_i3 | turquoise | 0.9716804 |
| TRINITY_DN36684_c1_g2_i21 | turquoise | 0.9705828 |
| TRINITY_DN25336_c0_g1_i1 | turquoise | 0.97036 |
| TRINITY_DN36311_c1_g1_i7 | turquoise | 0.9703265 |
| TRINITY_DN56436_c0_g1_i1 | turquoise | 0.9700424 |
| TRINITY_DN38103_c4_g4_i6 | turquoise | 0.9698869 |
| TRINITY_DN37893_c2_g4_i4 | turquoise | 0.9698346 |
| TRINITY_DN33583_c0_g1_i1 | turquoise | 0.9696955 |
| TRINITY_DN36390_c1_g1_i6 | turquoise | 0.9694952 |
| TRINITY_DN29436_c0_g1_i2 | turquoise | 0.9689079 |
| TRINITY_DN31316_c0_g5_i2 | turquoise | 0.9688187 |
| TRINITY_DN37235_c0_g1_i20 | turquoise | 0.9686732 |
| TRINITY_DN68100_c0_g1_i1 | turquoise | 0.9680004 |
| TRINITY_DN33010_c1_g3_i4 | turquoise | 0.9675877 |
| TRINITY_DN67486_c0_g1_i1 | turquoise | 0.9675619 |
| TRINITY_DN31692_c0_g1_i21 | turquoise | 0.9671565 |
| TRINITY_DN39337_c4_g1_i1 | turquoise | 0.9671279 |
| TRINITY_DN34307_c1_g2_i1 | turquoise | 0.9670978 |
| TRINITY_DN39194_c4_g6_i1 | turquoise | 0.9669411 |
| TRINITY_DN35314_c1_g3_i2 | turquoise | 0.9668308 |
| TRINITY_DN36010_c0_g1_i1 | turquoise | 0.9666912 |
| TRINITY_DN31671_c0_g1_i8 | turquoise | 0.9666505 |
| TRINITY_DN36593_c0_g3_i2 | turquoise | 0.966646 |
| TRINITY_DN34294_c0_g1_i7 | turquoise | 0.9665375 |
| TRINITY_DN38124_c0_g1_i1 | turquoise | 0.9664501 |
| TRINITY_DN35158_c0_g2_i3 | turquoise | 0.9663889 |
| TRINITY_DN38908_c1_g3_i1 | turquoise | 0.9663488 |
| TRINITY_DN38458_c1_g1_i1 | turquoise | 0.9663366 |
| TRINITY_DN39264_c4_g1_i6 | turquoise | 0.9661594 |
| TRINITY_DN34015_c0_g1_i5 | turquoise | 0.9661523 |
| TRINITY_DN38384_c2_g2_i2 | turquoise | 0.9660408 |
| TRINITY_DN23568_c0_g1_i1 | turquoise | 0.9660393 |
| TRINITY_DN33499_c1_g4_i5 | turquoise | 0.9660278 |
| TRINITY_DN5032_c0_g1_i1 | turquoise | 0.9660034 |
| TRINITY_DN35654_c0_g4_i1 | turquoise | 0.9653992 |
| TRINITY_DN37946_c0_g3_i6 | turquoise | 0.9653529 |
| TRINITY_DN38124_c0_g6_i1 | turquoise | 0.965319 |
| TRINITY_DN27300_c0_g1_i1 | turquoise | 0.9652767 |
| TRINITY_DN35220_c2_g1_i5 | turquoise | 0.9650605 |
| TRINITY_DN32520_c0_g4_i2 | turquoise | 0.965033 |
| TRINITY_DN35550_c0_g1_i20 | turquoise | 0.9650282 |
| TRINITY_DN37414_c1_g2_i1 | turquoise | 0.9650233 |
| TRINITY_DN33854_c1_g1_i8 | turquoise | 0.9648397 |
| TRINITY_DN21913_c0_g1_i1 | turquoise | 0.9645954 |
| TRINITY_DN33364_c0_g2_i11 | turquoise | 0.9644837 |
| TRINITY_DN36801_c1_g2_i5 | turquoise | 0.9642713 |
| TRINITY_DN37950_c1_g2_i14 | turquoise | 0.9642396 |
| TRINITY_DN31937_c1_g5_i1 | turquoise | 0.9642194 |
| TRINITY_DN33499_c1_g1_i1 | turquoise | 0.9641042 |
| TRINITY_DN70694_c0_g1_i1 | turquoise | 0.9640983 |
| TRINITY_DN34867_c0_g1_i6 | turquoise | 0.9639448 |
| TRINITY_DN52176_c0_g1_i1 | turquoise | 0.9638747 |
| TRINITY_DN33812_c0_g4_i1 | turquoise | 0.9637423 |
| TRINITY_DN24698_c0_g2_i1 | turquoise | 0.9637376 |
| TRINITY_DN23791_c0_g1_i1 | turquoise | 0.963597 |
| TRINITY_DN34111_c0_g1_i1 | turquoise | 0.9635399 |
| TRINITY_DN22287_c0_g1_i4 | turquoise | 0.9633971 |
| TRINITY_DN36096_c0_g2_i2 | turquoise | 0.9629899 |
| TRINITY_DN36521_c0_g4_i1 | turquoise | 0.9628169 |
| TRINITY_DN38952_c0_g2_i1 | turquoise | 0.962793 |
| TRINITY_DN29795_c0_g2_i1 | turquoise | 0.9627563 |
| TRINITY_DN31937_c1_g2_i1 | turquoise | 0.9627157 |
| TRINITY_DN28192_c0_g2_i10 | turquoise | 0.9626166 |
| TRINITY_DN37959_c2_g1_i4 | turquoise | 0.9623965 |
| TRINITY_DN50569_c0_g1_i1 | turquoise | 0.9623819 |
| TRINITY_DN67792_c0_g1_i1 | turquoise | 0.962364 |
| TRINITY_DN34395_c0_g2_i1 | turquoise | 0.9623542 |
| TRINITY_DN38954_c1_g1_i2 | turquoise | 0.9622993 |
| TRINITY_DN10111_c0_g1_i1 | turquoise | 0.9620475 |
| TRINITY_DN35950_c1_g1_i7 | turquoise | 0.9619629 |
| TRINITY_DN11533_c0_g1_i1 | turquoise | 0.9618838 |
| TRINITY_DN38415_c1_g3_i5 | turquoise | 0.961869 |
| TRINITY_DN30100_c0_g1_i4 | turquoise | 0.9618289 |
| TRINITY_DN30732_c0_g1_i1 | turquoise | 0.961815 |
| TRINITY_DN29607_c0_g2_i3 | turquoise | 0.961708 |
| TRINITY_DN37496_c1_g1_i19 | turquoise | 0.9616406 |
| TRINITY_DN37282_c0_g4_i5 | turquoise | 0.9616105 |
| TRINITY_DN19513_c0_g1_i1 | turquoise | 0.9609152 |
| TRINITY_DN27050_c0_g1_i1 | turquoise | 0.9609141 |
| TRINITY_DN32121_c0_g1_i3 | turquoise | 0.960727 |
| TRINITY_DN24141_c0_g1_i1 | turquoise | 0.9604643 |
| TRINITY_DN32647_c0_g1_i1 | turquoise | 0.9601665 |
| TRINITY_DN30259_c0_g1_i2 | turquoise | 0.960085 |
| TRINITY_DN34789_c1_g3_i1 | turquoise | 0.95997 |
| TRINITY_DN36096_c0_g3_i1 | turquoise | 0.9598791 |
| TRINITY_DN57622_c0_g1_i1 | turquoise | 0.9598429 |
| TRINITY_DN34218_c0_g1_i3 | turquoise | 0.9594817 |
| TRINITY_DN36572_c0_g4_i1 | turquoise | 0.9594575 |
| TRINITY_DN38309_c1_g4_i5 | turquoise | 0.9594178 |
| TRINITY_DN33553_c1_g1_i1 | turquoise | 0.9592854 |
| TRINITY_DN35073_c0_g1_i11 | turquoise | 0.959254 |
| TRINITY_DN39182_c1_g1_i1 | turquoise | 0.9591042 |
| TRINITY_DN36973_c0_g2_i4 | turquoise | 0.9590573 |
| TRINITY_DN30835_c0_g1_i3 | turquoise | 0.9588766 |
| TRINITY_DN37252_c1_g4_i20 | turquoise | 0.9588414 |
| TRINITY_DN46447_c0_g1_i1 | turquoise | 0.9587402 |
| TRINITY_DN39904_c0_g1_i1 | turquoise | 0.9586014 |
| TRINITY_DN21871_c0_g2_i1 | turquoise | 0.9585489 |
| TRINITY_DN28128_c0_g1_i2 | turquoise | 0.9584945 |
| TRINITY_DN37948_c1_g1_i1 | turquoise | 0.9582945 |
| TRINITY_DN35297_c0_g1_i4 | turquoise | 0.9582603 |
| TRINITY_DN34190_c0_g3_i9 | turquoise | 0.958193 |
| TRINITY_DN37211_c0_g1_i2 | turquoise | 0.9580918 |
| TRINITY_DN16203_c0_g2_i1 | turquoise | 0.9580152 |
| TRINITY_DN35207_c0_g2_i4 | turquoise | 0.9579452 |
| TRINITY_DN32262_c1_g1_i9 | turquoise | 0.9577975 |
| TRINITY_DN37747_c0_g1_i5 | turquoise | 0.9577528 |
| TRINITY_DN31325_c0_g1_i7 | turquoise | 0.9576762 |
| TRINITY_DN38648_c0_g1_i24 | turquoise | 0.9575449 |
| TRINITY_DN34744_c0_g1_i2 | turquoise | 0.9575182 |
| TRINITY_DN15188_c0_g1_i1 | turquoise | 0.9572467 |
| TRINITY_DN32886_c1_g2_i7 | turquoise | 0.957186 |
| TRINITY_DN25257_c0_g1_i2 | turquoise | 0.9571281 |
| TRINITY_DN33803_c1_g1_i5 | turquoise | 0.9570521 |
| TRINITY_DN26428_c0_g1_i1 | turquoise | 0.9570118 |
| TRINITY_DN2200_c0_g1_i1 | turquoise | 0.9569326 |
| TRINITY_DN39071_c0_g1_i1 | turquoise | 0.956893 |
| TRINITY_DN63752_c0_g1_i1 | turquoise | 0.9567936 |
| TRINITY_DN56300_c0_g1_i1 | turquoise | 0.95662 |
| TRINITY_DN34438_c0_g1_i8 | turquoise | 0.9565811 |
| TRINITY_DN26227_c0_g1_i1 | turquoise | 0.9562488 |
| TRINITY_DN26197_c0_g1_i1 | turquoise | 0.9561321 |
| TRINITY_DN37212_c2_g1_i13 | turquoise | 0.9558178 |
| TRINITY_DN11612_c0_g1_i1 | turquoise | 0.9556486 |
| TRINITY_DN37425_c0_g2_i22 | turquoise | 0.9555997 |
| TRINITY_DN34790_c2_g1_i4 | turquoise | 0.9555869 |
| TRINITY_DN39067_c1_g2_i1 | turquoise | 0.9554965 |
| TRINITY_DN30040_c0_g2_i3 | turquoise | 0.9554955 |
| TRINITY_DN51218_c0_g1_i1 | turquoise | 0.9554118 |
| TRINITY_DN29272_c0_g2_i1 | turquoise | 0.9553458 |
| TRINITY_DN45591_c0_g1_i1 | turquoise | 0.9552922 |
| TRINITY_DN37795_c1_g1_i4 | turquoise | 0.955277 |
| TRINITY_DN31840_c0_g1_i5 | turquoise | 0.9551827 |
| TRINITY_DN23780_c0_g3_i1 | turquoise | 0.9551723 |
| TRINITY_DN55942_c0_g1_i1 | turquoise | 0.9551562 |
| TRINITY_DN36756_c0_g1_i3 | turquoise | 0.9551052 |
| TRINITY_DN29300_c0_g2_i2 | turquoise | 0.9549033 |
| TRINITY_DN36617_c0_g4_i10 | turquoise | 0.9546454 |
| TRINITY_DN52214_c0_g1_i1 | turquoise | 0.9545458 |
| TRINITY_DN40446_c0_g1_i1 | turquoise | 0.9544364 |
| TRINITY_DN67768_c0_g1_i1 | turquoise | 0.9542905 |
| TRINITY_DN30713_c4_g10_i1 | turquoise | 0.9540653 |
| TRINITY_DN29352_c0_g1_i1 | turquoise | 0.953866 |
| TRINITY_DN13776_c0_g2_i1 | turquoise | 0.9537924 |
| TRINITY_DN30583_c0_g1_i5 | turquoise | 0.9536675 |
| TRINITY_DN57189_c0_g1_i1 | turquoise | 0.9536514 |
| TRINITY_DN34528_c0_g1_i7 | turquoise | 0.9534035 |
| TRINITY_DN64114_c0_g1_i1 | turquoise | 0.952947 |
| TRINITY_DN32773_c0_g2_i10 | turquoise | 0.9528997 |
| TRINITY_DN33891_c0_g2_i7 | turquoise | 0.9528508 |
| TRINITY_DN27822_c0_g1_i6 | turquoise | 0.9527399 |
| TRINITY_DN32578_c0_g1_i2 | turquoise | 0.9524951 |
| TRINITY_DN34326_c0_g1_i1 | turquoise | 0.9520905 |
| TRINITY_DN38209_c0_g4_i19 | turquoise | 0.9519838 |
| TRINITY_DN27116_c0_g1_i1 | turquoise | 0.951962 |
| TRINITY_DN68225_c0_g1_i1 | turquoise | 0.9519569 |
| TRINITY_DN38126_c2_g1_i1 | turquoise | 0.9518948 |
| TRINITY_DN35427_c1_g1_i3 | turquoise | 0.9518103 |
| TRINITY_DN31180_c0_g1_i5 | turquoise | 0.951763 |
| TRINITY_DN32867_c2_g2_i6 | turquoise | 0.9517052 |
| TRINITY_DN36998_c0_g4_i3 | turquoise | 0.9516582 |
| TRINITY_DN30547_c0_g1_i3 | turquoise | 0.9515647 |
| TRINITY_DN29295_c0_g1_i4 | turquoise | 0.951351 |
| TRINITY_DN37815_c0_g4_i2 | turquoise | 0.9512876 |
| TRINITY_DN37803_c0_g3_i3 | turquoise | 0.951251 |
| TRINITY_DN24980_c0_g1_i1 | turquoise | 0.9512384 |
| TRINITY_DN38934_c0_g2_i1 | turquoise | 0.9511935 |
| TRINITY_DN12282_c0_g1_i1 | turquoise | 0.9508283 |
| TRINITY_DN38126_c2_g2_i1 | turquoise | 0.9507691 |
| TRINITY_DN31687_c1_g2_i1 | turquoise | 0.9507324 |
| TRINITY_DN32524_c1_g1_i1 | turquoise | 0.9506338 |
| TRINITY_DN52976_c0_g1_i1 | turquoise | 0.9506268 |
| TRINITY_DN28192_c0_g3_i2 | turquoise | 0.950479 |
| TRINITY_DN31995_c0_g2_i1 | turquoise | 0.9503689 |
| TRINITY_DN46664_c0_g1_i1 | turquoise | 0.9503626 |
| TRINITY_DN31944_c2_g1_i10 | turquoise | 0.9502629 |
| TRINITY_DN38132_c0_g1_i9 | turquoise | 0.9502197 |
| TRINITY_DN62368_c0_g1_i1 | turquoise | 0.950211 |
| TRINITY_DN22023_c0_g1_i3 | turquoise | 0.9502061 |
| TRINITY_DN30708_c0_g2_i1 | turquoise | 0.9501292 |
| TRINITY_DN33776_c0_g1_i3 | turquoise | 0.9499745 |
| TRINITY_DN36151_c0_g2_i7 | turquoise | 0.9498968 |
| TRINITY_DN38019_c0_g2_i7 | turquoise | 0.9498139 |
| TRINITY_DN33064_c0_g1_i1 | turquoise | 0.9496694 |
| TRINITY_DN39177_c2_g3_i1 | turquoise | 0.9496557 |
| TRINITY_DN26365_c0_g1_i5 | turquoise | 0.9494952 |
| TRINITY_DN31903_c2_g3_i2 | turquoise | 0.9493146 |
| TRINITY_DN31855_c0_g2_i5 | turquoise | 0.9492855 |
| TRINITY_DN35451_c0_g1_i3 | turquoise | 0.9492834 |
| TRINITY_DN27479_c0_g4_i1 | turquoise | 0.9491628 |
| TRINITY_DN36666_c0_g1_i7 | turquoise | 0.9491497 |
| TRINITY_DN56492_c0_g1_i1 | turquoise | 0.9491463 |
| TRINITY_DN1735_c0_g2_i1 | turquoise | 0.9490562 |
| TRINITY_DN32879_c1_g2_i2 | turquoise | 0.9490077 |
| TRINITY_DN56867_c0_g1_i1 | turquoise | 0.948989 |
| TRINITY_DN4272_c0_g2_i1 | turquoise | 0.9489783 |
| TRINITY_DN28403_c0_g1_i1 | turquoise | 0.9489609 |
| TRINITY_DN24073_c0_g1_i3 | turquoise | 0.9488646 |
| TRINITY_DN32254_c0_g1_i1 | turquoise | 0.9486168 |
| TRINITY_DN35379_c0_g4_i2 | turquoise | 0.9486063 |
| TRINITY_DN37562_c0_g2_i1 | turquoise | 0.9484401 |
| TRINITY_DN36756_c0_g3_i3 | turquoise | 0.9482539 |
| TRINITY_DN37314_c1_g1_i1 | turquoise | 0.9481778 |
| TRINITY_DN32650_c0_g1_i2 | turquoise | 0.9480388 |
| TRINITY_DN35837_c2_g2_i10 | turquoise | 0.9480301 |
| TRINITY_DN35466_c0_g4_i6 | turquoise | 0.9478678 |
| TRINITY_DN31015_c0_g3_i2 | turquoise | 0.9478601 |
| TRINITY_DN35012_c0_g1_i4 | turquoise | 0.9477774 |
| TRINITY_DN38905_c0_g1_i6 | turquoise | 0.947674 |
| TRINITY_DN11339_c0_g1_i1 | turquoise | 0.9475786 |
| TRINITY_DN13911_c0_g1_i1 | turquoise | 0.9475755 |
| TRINITY_DN46893_c0_g1_i1 | turquoise | 0.9475735 |
| TRINITY_DN37276_c1_g3_i1 | turquoise | 0.947499 |
| TRINITY_DN45563_c0_g1_i1 | turquoise | 0.9469308 |
| TRINITY_DN51868_c0_g1_i1 | turquoise | 0.9465612 |
| TRINITY_DN36290_c0_g1_i2 | turquoise | 0.9464293 |
| TRINITY_DN26963_c1_g1_i1 | turquoise | 0.9463223 |
| TRINITY_DN10742_c0_g2_i1 | turquoise | 0.9460282 |
| TRINITY_DN35002_c0_g2_i1 | turquoise | 0.9455055 |
| TRINITY_DN35660_c1_g2_i18 | turquoise | 0.9454358 |
| TRINITY_DN45196_c0_g1_i1 | turquoise | 0.9453963 |
| TRINITY_DN37900_c3_g2_i1 | turquoise | 0.94533 |
| TRINITY_DN32790_c0_g2_i5 | turquoise | 0.9450454 |
| TRINITY_DN61739_c0_g1_i1 | turquoise | 0.9450062 |
| TRINITY_DN30010_c0_g1_i13 | turquoise | 0.9449775 |
| TRINITY_DN58251_c0_g1_i1 | turquoise | 0.944934 |
| TRINITY_DN39285_c1_g9_i10 | turquoise | 0.9447582 |
| TRINITY_DN56553_c0_g1_i1 | turquoise | 0.9447577 |
| TRINITY_DN28535_c0_g1_i1 | turquoise | 0.944643 |
| TRINITY_DN35073_c0_g2_i2 | turquoise | 0.9446184 |
| TRINITY_DN38181_c0_g5_i1 | turquoise | 0.9445911 |
| TRINITY_DN37343_c0_g4_i1 | turquoise | 0.9443001 |
| TRINITY_DN30938_c0_g2_i1 | turquoise | 0.9442049 |
| TRINITY_DN34501_c0_g2_i1 | turquoise | 0.9441458 |
| TRINITY_DN62801_c0_g1_i1 | turquoise | 0.943933 |
| TRINITY_DN39075_c0_g1_i17 | turquoise | 0.9439165 |
| TRINITY_DN34332_c0_g2_i1 | turquoise | 0.9438834 |
| TRINITY_DN63629_c0_g1_i1 | turquoise | 0.9437999 |
| TRINITY_DN51686_c0_g1_i1 | turquoise | 0.9437963 |
| TRINITY_DN33034_c0_g1_i2 | turquoise | 0.9435551 |
| TRINITY_DN36354_c0_g2_i1 | turquoise | 0.9434706 |
| TRINITY_DN31737_c0_g1_i4 | turquoise | 0.9433888 |
| TRINITY_DN37664_c0_g5_i1 | turquoise | 0.9431872 |
| TRINITY_DN38124_c0_g4_i4 | turquoise | 0.9431665 |
| TRINITY_DN37884_c0_g2_i1 | turquoise | 0.9428518 |
| TRINITY_DN36157_c1_g12_i11 | turquoise | 0.9428469 |
| TRINITY_DN14074_c0_g2_i1 | turquoise | 0.9427361 |
| TRINITY_DN36917_c0_g1_i6 | turquoise | 0.94273 |
| TRINITY_DN33939_c0_g2_i11 | turquoise | 0.9426129 |
| TRINITY_DN37581_c0_g2_i7 | turquoise | 0.942527 |
| TRINITY_DN36179_c0_g1_i1 | turquoise | 0.9424409 |
| TRINITY_DN33990_c1_g1_i6 | turquoise | 0.9421898 |
| TRINITY_DN31569_c0_g1_i2 | turquoise | 0.9420486 |
| TRINITY_DN56817_c0_g1_i1 | turquoise | 0.9420449 |
| TRINITY_DN57963_c0_g1_i1 | turquoise | 0.9420279 |
| TRINITY_DN38254_c1_g2_i3 | turquoise | 0.9419203 |
| TRINITY_DN16767_c0_g3_i1 | turquoise | 0.9419037 |
| TRINITY_DN29035_c0_g1_i2 | turquoise | 0.94189 |
| TRINITY_DN17364_c0_g2_i1 | turquoise | 0.9418345 |
| TRINITY_DN25293_c0_g1_i1 | turquoise | 0.9417738 |
| TRINITY_DN17945_c0_g1_i1 | turquoise | 0.9417408 |
| TRINITY_DN33911_c0_g2_i2 | turquoise | 0.9413072 |
| TRINITY_DN35365_c2_g3_i2 | turquoise | 0.9412733 |
| TRINITY_DN39402_c0_g1_i1 | turquoise | 0.9409673 |
| TRINITY_DN39177_c2_g2_i1 | turquoise | 0.9408783 |
| TRINITY_DN33717_c0_g1_i2 | turquoise | 0.9408632 |
| TRINITY_DN34555_c3_g1_i2 | turquoise | 0.9407417 |
| TRINITY_DN36865_c1_g1_i1 | turquoise | 0.9406469 |
| TRINITY_DN30830_c0_g1_i1 | turquoise | 0.9402828 |
| TRINITY_DN32929_c0_g2_i2 | turquoise | 0.9402792 |
| TRINITY_DN36028_c1_g1_i1 | turquoise | 0.940083 |
| TRINITY_DN29163_c0_g1_i4 | turquoise | 0.9400666 |
| TRINITY_DN32475_c0_g1_i4 | turquoise | 0.9400257 |
| TRINITY_DN69550_c0_g1_i1 | turquoise | 0.9399907 |
| TRINITY_DN34633_c0_g2_i2 | turquoise | 0.9397362 |
| TRINITY_DN33743_c0_g2_i1 | turquoise | 0.9396631 |
| TRINITY_DN33053_c1_g1_i1 | turquoise | 0.9396006 |
| TRINITY_DN30371_c0_g1_i1 | turquoise | 0.93958 |
| TRINITY_DN67845_c0_g1_i1 | turquoise | 0.9395517 |
| TRINITY_DN36355_c0_g2_i1 | turquoise | 0.9395361 |
| TRINITY_DN67316_c0_g1_i1 | turquoise | 0.9394552 |
| TRINITY_DN34340_c0_g1_i2 | turquoise | 0.9394238 |
| TRINITY_DN44867_c0_g1_i1 | turquoise | 0.9392428 |
| TRINITY_DN37457_c0_g1_i4 | turquoise | 0.9389109 |
| TRINITY_DN35517_c0_g4_i3 | turquoise | 0.9388605 |
| TRINITY_DN36640_c0_g3_i2 | turquoise | 0.9387608 |
| TRINITY_DN31160_c0_g1_i5 | turquoise | 0.9387526 |
| TRINITY_DN31472_c0_g1_i5 | turquoise | 0.9387495 |
| TRINITY_DN27962_c1_g2_i1 | turquoise | 0.9386874 |
| TRINITY_DN2877_c0_g1_i1 | turquoise | 0.9385957 |
| TRINITY_DN37947_c0_g2_i15 | turquoise | 0.9381712 |
| TRINITY_DN68456_c0_g1_i1 | turquoise | 0.9381168 |
| TRINITY_DN17193_c0_g2_i1 | turquoise | 0.938083 |
| TRINITY_DN36439_c0_g1_i13 | turquoise | 0.9380318 |
| TRINITY_DN38904_c1_g4_i1 | turquoise | 0.9380062 |
| TRINITY_DN34858_c0_g3_i6 | turquoise | 0.9379632 |
| TRINITY_DN33517_c1_g1_i2 | turquoise | 0.9379596 |
| TRINITY_DN20326_c0_g1_i1 | turquoise | 0.9377626 |
| TRINITY_DN30708_c0_g4_i1 | turquoise | 0.9377438 |
| TRINITY_DN38859_c0_g4_i1 | turquoise | 0.9376706 |
| TRINITY_DN24502_c0_g4_i1 | turquoise | 0.9376427 |
| TRINITY_DN37276_c1_g2_i3 | turquoise | 0.9376068 |
| TRINITY_DN30329_c1_g3_i2 | turquoise | 0.9375714 |
| TRINITY_DN34163_c2_g2_i4 | turquoise | 0.9375328 |
| TRINITY_DN29144_c0_g1_i1 | turquoise | 0.9374602 |
| TRINITY_DN34803_c1_g8_i1 | turquoise | 0.9374237 |
| TRINITY_DN42260_c0_g1_i1 | turquoise | 0.9373617 |
| TRINITY_DN34358_c0_g1_i20 | turquoise | 0.9373176 |
| TRINITY_DN61959_c0_g1_i1 | turquoise | 0.9371963 |
| TRINITY_DN36846_c0_g3_i1 | turquoise | 0.937171 |
| TRINITY_DN25588_c0_g1_i4 | turquoise | 0.9369899 |
| TRINITY_DN27507_c0_g2_i6 | turquoise | 0.9368835 |
| TRINITY_DN33761_c0_g1_i4 | turquoise | 0.9368599 |
| TRINITY_DN33973_c0_g1_i14 | turquoise | 0.9368581 |
| TRINITY_DN17480_c0_g2_i1 | turquoise | 0.9367414 |
| TRINITY_DN36115_c2_g2_i1 | turquoise | 0.9365552 |
| TRINITY_DN27668_c0_g1_i1 | turquoise | 0.9365333 |
| TRINITY_DN38331_c3_g1_i6 | turquoise | 0.9363219 |
| TRINITY_DN37924_c0_g5_i6 | turquoise | 0.9362803 |
| TRINITY_DN9442_c0_g1_i1 | turquoise | 0.9362555 |
| TRINITY_DN39151_c0_g2_i6 | turquoise | 0.9362494 |
| TRINITY_DN38260_c0_g1_i4 | turquoise | 0.9361171 |
| TRINITY_DN33097_c0_g1_i6 | turquoise | 0.9360375 |
| TRINITY_DN37381_c0_g1_i1 | turquoise | 0.93603 |
| TRINITY_DN16053_c0_g1_i1 | turquoise | 0.9359847 |
| TRINITY_DN38254_c1_g1_i1 | turquoise | 0.9358664 |
| TRINITY_DN31101_c0_g1_i11 | turquoise | 0.9357078 |
| TRINITY_DN38605_c1_g2_i8 | turquoise | 0.9356759 |
| TRINITY_DN35269_c0_g1_i10 | turquoise | 0.9356633 |
| TRINITY_DN38181_c0_g3_i8 | turquoise | 0.9354445 |
| TRINITY_DN30854_c0_g1_i1 | turquoise | 0.9352118 |
| TRINITY_DN30074_c1_g11_i1 | turquoise | 0.9351751 |
| TRINITY_DN35453_c0_g2_i4 | turquoise | 0.9351218 |
| TRINITY_DN10305_c0_g1_i1 | turquoise | 0.9350417 |
| TRINITY_DN32758_c1_g3_i2 | turquoise | 0.9349972 |
| TRINITY_DN68728_c0_g1_i1 | turquoise | 0.934909 |
| TRINITY_DN38543_c1_g7_i1 | turquoise | 0.9348043 |
| TRINITY_DN32631_c0_g1_i10 | turquoise | 0.9347678 |
| TRINITY_DN45605_c0_g1_i1 | turquoise | 0.9346862 |
| TRINITY_DN32537_c0_g1_i1 | turquoise | 0.934628 |
| TRINITY_DN37708_c0_g1_i3 | turquoise | 0.9345404 |
| TRINITY_DN56469_c0_g1_i1 | turquoise | 0.93452 |
| TRINITY_DN25577_c0_g1_i1 | turquoise | 0.9345127 |
| TRINITY_DN21894_c0_g1_i1 | turquoise | 0.9344277 |
| TRINITY_DN38850_c1_g2_i3 | turquoise | 0.9344082 |
| TRINITY_DN19069_c0_g2_i1 | turquoise | 0.9342274 |
| TRINITY_DN39192_c3_g6_i1 | turquoise | 0.934147 |
| TRINITY_DN28322_c0_g1_i2 | turquoise | 0.9340617 |
| TRINITY_DN32000_c0_g1_i1 | turquoise | 0.9340559 |
| TRINITY_DN31937_c1_g1_i1 | turquoise | 0.9340084 |
| TRINITY_DN30406_c0_g1_i5 | turquoise | 0.9339951 |
| TRINITY_DN31633_c0_g2_i1 | turquoise | 0.9338907 |
| TRINITY_DN22150_c0_g2_i1 | turquoise | 0.9338247 |
| TRINITY_DN38957_c2_g1_i1 | turquoise | 0.9337579 |
| TRINITY_DN32834_c0_g1_i7 | turquoise | 0.9337325 |
| TRINITY_DN39602_c0_g1_i1 | turquoise | 0.9337161 |
| TRINITY_DN26353_c0_g3_i2 | turquoise | 0.9336206 |
| TRINITY_DN63659_c0_g1_i1 | turquoise | 0.9335952 |
| TRINITY_DN38193_c0_g1_i4 | turquoise | 0.9333761 |
| TRINITY_DN33792_c1_g3_i2 | turquoise | 0.9333021 |
| TRINITY_DN38739_c0_g2_i4 | turquoise | 0.9331723 |
| TRINITY_DN36275_c0_g1_i6 | turquoise | 0.9330991 |
| TRINITY_DN35621_c0_g2_i1 | turquoise | 0.932956 |
| TRINITY_DN5106_c0_g1_i1 | turquoise | 0.9329541 |
| TRINITY_DN56302_c0_g1_i1 | turquoise | 0.9329376 |
| TRINITY_DN36232_c0_g3_i1 | turquoise | 0.9327281 |
| TRINITY_DN42265_c0_g1_i1 | turquoise | 0.932329 |
| TRINITY_DN30494_c0_g1_i1 | turquoise | 0.9323156 |
| TRINITY_DN38956_c1_g5_i2 | turquoise | 0.9321576 |
| TRINITY_DN36999_c0_g2_i3 | turquoise | 0.9321391 |
| TRINITY_DN26433_c0_g1_i3 | turquoise | 0.9320543 |
| TRINITY_DN37639_c1_g1_i17 | turquoise | 0.9320435 |
| TRINITY_DN35666_c0_g2_i2 | turquoise | 0.9319365 |
| TRINITY_DN64440_c0_g1_i1 | turquoise | 0.9319199 |
| TRINITY_DN37529_c1_g3_i6 | turquoise | 0.9319178 |
| TRINITY_DN15541_c0_g1_i1 | turquoise | 0.9319021 |
| TRINITY_DN29525_c0_g1_i1 | turquoise | 0.9318756 |
| TRINITY_DN36955_c1_g2_i1 | turquoise | 0.9316966 |
| TRINITY_DN37078_c0_g7_i3 | turquoise | 0.9313682 |
| TRINITY_DN61940_c0_g1_i1 | turquoise | 0.9311941 |
| TRINITY_DN17290_c0_g1_i1 | turquoise | 0.9311699 |
| TRINITY_DN36000_c1_g5_i1 | turquoise | 0.9311047 |
| TRINITY_DN38601_c0_g1_i2 | turquoise | 0.9309915 |
| TRINITY_DN313_c0_g2_i1 | turquoise | 0.9309529 |
| TRINITY_DN36844_c1_g1_i10 | turquoise | 0.9309194 |
| TRINITY_DN39330_c1_g2_i4 | turquoise | 0.9308109 |
| TRINITY_DN68778_c0_g1_i1 | turquoise | 0.93072 |
| TRINITY_DN28248_c1_g1_i3 | turquoise | 0.9304978 |
| TRINITY_DN33551_c0_g2_i1 | turquoise | 0.9304332 |
| TRINITY_DN40396_c0_g1_i1 | turquoise | 0.9303866 |
| TRINITY_DN45461_c0_g1_i1 | turquoise | 0.9303153 |
| TRINITY_DN33114_c0_g1_i1 | turquoise | 0.930178 |
| TRINITY_DN2051_c0_g1_i1 | turquoise | 0.9301758 |
| TRINITY_DN38230_c0_g1_i5 | turquoise | 0.9300598 |
| TRINITY_DN15823_c0_g2_i1 | turquoise | 0.9296611 |
| TRINITY_DN38148_c0_g2_i6 | turquoise | 0.9294516 |
| TRINITY_DN39059_c4_g10_i1 | turquoise | 0.9293594 |
| TRINITY_DN34856_c0_g3_i6 | turquoise | 0.9293086 |
| TRINITY_DN35691_c0_g2_i6 | turquoise | 0.9292942 |
| TRINITY_DN32021_c0_g1_i3 | turquoise | 0.9291074 |
| TRINITY_DN20816_c0_g1_i1 | turquoise | 0.929074 |
| TRINITY_DN33053_c0_g1_i1 | turquoise | 0.9288347 |
| TRINITY_DN31451_c0_g12_i1 | turquoise | 0.9287669 |
| TRINITY_DN280_c0_g1_i1 | turquoise | 0.9287213 |
| TRINITY_DN39221_c1_g5_i5 | turquoise | 0.9286427 |
| TRINITY_DN31336_c0_g1_i3 | turquoise | 0.9286273 |
| TRINITY_DN20638_c0_g1_i2 | turquoise | 0.9286103 |
| TRINITY_DN28815_c0_g1_i1 | turquoise | 0.9285797 |
| TRINITY_DN56562_c0_g1_i1 | turquoise | 0.9285108 |
| TRINITY_DN39558_c0_g1_i1 | turquoise | 0.9285081 |
| TRINITY_DN36631_c0_g3_i4 | turquoise | 0.9284947 |
| TRINITY_DN35252_c2_g1_i1 | turquoise | 0.9283906 |
| TRINITY_DN36670_c1_g2_i4 | turquoise | 0.9282525 |
| TRINITY_DN36178_c0_g9_i11 | turquoise | 0.9280562 |
| TRINITY_DN33243_c0_g1_i1 | turquoise | 0.9280211 |
| TRINITY_DN17892_c0_g1_i1 | turquoise | 0.9279875 |
| TRINITY_DN21537_c0_g3_i1 | turquoise | 0.9279047 |
| TRINITY_DN22267_c0_g1_i1 | turquoise | 0.9278051 |
| TRINITY_DN21894_c0_g6_i1 | turquoise | 0.9277843 |
| TRINITY_DN38894_c0_g13_i1 | turquoise | 0.9277026 |
| TRINITY_DN39472_c0_g1_i1 | turquoise | 0.9276994 |
| TRINITY_DN37447_c2_g4_i1 | turquoise | 0.9276691 |
| TRINITY_DN33203_c2_g1_i1 | turquoise | 0.9276646 |
| TRINITY_DN35562_c0_g5_i1 | turquoise | 0.927633 |
| TRINITY_DN33117_c0_g1_i11 | turquoise | 0.927602 |
| TRINITY_DN34986_c2_g1_i1 | turquoise | 0.9272669 |
| TRINITY_DN33615_c0_g1_i12 | turquoise | 0.9272317 |
| TRINITY_DN61789_c0_g1_i1 | turquoise | 0.9271298 |
| TRINITY_DN35594_c0_g1_i1 | turquoise | 0.927094 |
| TRINITY_DN29953_c0_g2_i2 | turquoise | 0.9270799 |
| TRINITY_DN31450_c0_g1_i1 | turquoise | 0.9269193 |
| TRINITY_DN42780_c0_g1_i1 | turquoise | 0.926794 |
| TRINITY_DN33198_c0_g1_i1 | turquoise | 0.926792 |
| TRINITY_DN33924_c2_g3_i4 | turquoise | 0.9267684 |
| TRINITY_DN32285_c0_g2_i3 | turquoise | 0.9265699 |
| TRINITY_DN35595_c0_g3_i2 | turquoise | 0.9263864 |
| TRINITY_DN46761_c0_g1_i1 | turquoise | 0.926219 |
| TRINITY_DN36744_c1_g1_i1 | turquoise | 0.9262096 |
| TRINITY_DN34986_c2_g5_i5 | turquoise | 0.9261632 |
| TRINITY_DN29731_c0_g2_i1 | turquoise | 0.9261307 |
| TRINITY_DN39337_c4_g5_i5 | turquoise | 0.9261237 |
| TRINITY_DN30019_c0_g1_i2 | turquoise | 0.9259458 |
| TRINITY_DN18855_c0_g1_i1 | turquoise | 0.9258876 |
| TRINITY_DN10741_c0_g1_i1 | turquoise | 0.9258267 |
| TRINITY_DN24683_c0_g1_i3 | turquoise | 0.9258049 |
| TRINITY_DN30546_c0_g1_i1 | turquoise | 0.9256938 |
| TRINITY_DN31846_c0_g1_i1 | turquoise | 0.9256301 |
| TRINITY_DN34062_c0_g1_i2 | turquoise | 0.9256008 |
| TRINITY_DN26536_c0_g1_i2 | turquoise | 0.9255294 |
| TRINITY_DN29887_c1_g1_i4 | turquoise | 0.9253113 |
| TRINITY_DN32914_c0_g1_i8 | turquoise | 0.9252546 |
| TRINITY_DN25522_c0_g2_i1 | turquoise | 0.9251386 |
| TRINITY_DN36040_c0_g2_i2 | turquoise | 0.925091 |
| TRINITY_DN37854_c1_g2_i7 | turquoise | 0.9250056 |
| TRINITY_DN37226_c0_g2_i1 | turquoise | 0.9250053 |
| TRINITY_DN33768_c0_g2_i5 | turquoise | 0.9248199 |
| TRINITY_DN32088_c0_g1_i4 | turquoise | 0.9247626 |
| TRINITY_DN23085_c0_g1_i1 | turquoise | 0.9247229 |
| TRINITY_DN35080_c0_g1_i1 | turquoise | 0.9245922 |
| TRINITY_DN24848_c0_g1_i1 | turquoise | 0.9243954 |
| TRINITY_DN33382_c0_g6_i1 | turquoise | 0.9243855 |
| TRINITY_DN69165_c0_g1_i1 | turquoise | 0.924373 |
| TRINITY_DN34258_c0_g1_i9 | turquoise | 0.9242455 |
| TRINITY_DN24921_c0_g1_i1 | turquoise | 0.9241433 |
| TRINITY_DN38466_c1_g2_i1 | turquoise | 0.9238643 |
| TRINITY_DN68302_c0_g1_i1 | turquoise | 0.923851 |
| TRINITY_DN27319_c0_g1_i2 | turquoise | 0.9237679 |
| TRINITY_DN28628_c0_g1_i4 | turquoise | 0.9237581 |
| TRINITY_DN29154_c0_g1_i1 | turquoise | 0.9237182 |
| TRINITY_DN23493_c0_g1_i1 | turquoise | 0.9236859 |
| TRINITY_DN34899_c1_g3_i4 | turquoise | 0.9236525 |
| TRINITY_DN34761_c1_g1_i12 | turquoise | 0.9236435 |
| TRINITY_DN26871_c0_g1_i1 | turquoise | 0.9236145 |
| TRINITY_DN31938_c2_g1_i1 | turquoise | 0.9234754 |
| TRINITY_DN63531_c0_g1_i1 | turquoise | 0.9234378 |
| TRINITY_DN70555_c0_g1_i1 | turquoise | 0.9231933 |
| TRINITY_DN67890_c0_g1_i1 | turquoise | 0.9231588 |
| TRINITY_DN34308_c0_g2_i1 | turquoise | 0.9230237 |
| TRINITY_DN63019_c0_g1_i1 | turquoise | 0.9229586 |
| TRINITY_DN38767_c4_g1_i4 | turquoise | 0.9228949 |
| TRINITY_DN36014_c0_g1_i2 | turquoise | 0.9228875 |
| TRINITY_DN28099_c0_g1_i3 | turquoise | 0.9228779 |
| TRINITY_DN29268_c0_g1_i7 | turquoise | 0.9228611 |
| TRINITY_DN31810_c0_g1_i1 | turquoise | 0.922782 |
| TRINITY_DN30848_c4_g2_i1 | turquoise | 0.922713 |
| TRINITY_DN37717_c1_g3_i8 | turquoise | 0.9226906 |
| TRINITY_DN38815_c1_g9_i1 | turquoise | 0.9226424 |
| TRINITY_DN56543_c0_g1_i1 | turquoise | 0.9225877 |
| TRINITY_DN38139_c0_g1_i20 | turquoise | 0.9225029 |
| TRINITY_DN56252_c0_g1_i1 | turquoise | 0.9224918 |
| TRINITY_DN36308_c0_g1_i1 | turquoise | 0.9222716 |
| TRINITY_DN31995_c1_g1_i1 | turquoise | 0.9222648 |
| TRINITY_DN31960_c0_g3_i1 | turquoise | 0.9221841 |
| TRINITY_DN45630_c0_g1_i1 | turquoise | 0.9221646 |
| TRINITY_DN25962_c0_g1_i2 | turquoise | 0.9221484 |
| TRINITY_DN68268_c0_g1_i1 | turquoise | 0.9220963 |
| TRINITY_DN33598_c1_g1_i4 | turquoise | 0.922053 |
| TRINITY_DN56691_c0_g1_i1 | turquoise | 0.9219779 |
| TRINITY_DN30124_c0_g1_i2 | turquoise | 0.921859 |
| TRINITY_DN28503_c1_g1_i1 | turquoise | 0.9217975 |
| TRINITY_DN50527_c0_g1_i1 | turquoise | 0.9217093 |
| TRINITY_DN33097_c0_g2_i1 | turquoise | 0.9217062 |
| TRINITY_DN27472_c0_g4_i4 | turquoise | 0.9216666 |
| TRINITY_DN29693_c0_g1_i1 | turquoise | 0.9215135 |
| TRINITY_DN35223_c0_g3_i11 | turquoise | 0.9214998 |
| TRINITY_DN33421_c0_g1_i14 | turquoise | 0.9214515 |
| TRINITY_DN32013_c2_g1_i1 | turquoise | 0.9214334 |
| TRINITY_DN38807_c0_g7_i6 | turquoise | 0.9214086 |
| TRINITY_DN36020_c0_g1_i2 | turquoise | 0.9213878 |
| TRINITY_DN31279_c1_g1_i3 | turquoise | 0.9213189 |
| TRINITY_DN35385_c0_g1_i2 | turquoise | 0.9212907 |
| TRINITY_DN38820_c0_g2_i14 | turquoise | 0.9212634 |
| TRINITY_DN45749_c0_g1_i1 | turquoise | 0.9211523 |
| TRINITY_DN33421_c0_g2_i5 | turquoise | 0.9210238 |
| TRINITY_DN29671_c0_g1_i1 | turquoise | 0.9210229 |
| TRINITY_DN28751_c0_g1_i2 | turquoise | 0.9210138 |
| TRINITY_DN37814_c0_g2_i12 | turquoise | 0.9208961 |
| TRINITY_DN57438_c0_g1_i1 | turquoise | 0.920774 |
| TRINITY_DN36928_c0_g1_i5 | turquoise | 0.9206773 |
| TRINITY_DN24592_c0_g2_i1 | turquoise | 0.9204408 |
| TRINITY_DN26590_c0_g1_i6 | turquoise | 0.9203587 |
| TRINITY_DN27178_c0_g1_i5 | turquoise | 0.9203245 |
| TRINITY_DN35037_c0_g1_i15 | turquoise | 0.9202033 |
| TRINITY_DN16345_c0_g2_i1 | turquoise | 0.9201213 |
| TRINITY_DN30797_c0_g1_i1 | turquoise | 0.9201082 |
| TRINITY_DN29800_c0_g1_i2 | turquoise | 0.9200504 |
| TRINITY_DN30126_c0_g1_i3 | turquoise | 0.9199941 |
| TRINITY_DN57360_c0_g1_i1 | turquoise | 0.9199744 |
| TRINITY_DN37450_c1_g2_i1 | turquoise | 0.9199658 |
| TRINITY_DN39192_c3_g7_i6 | turquoise | 0.9199379 |
| TRINITY_DN122_c0_g1_i1 | turquoise | 0.9199304 |
| TRINITY_DN28292_c0_g1_i2 | turquoise | 0.9198143 |
| TRINITY_DN4395_c0_g1_i1 | turquoise | 0.9197795 |
| TRINITY_DN18304_c0_g2_i1 | turquoise | 0.9197591 |
| TRINITY_DN28923_c0_g1_i1 | turquoise | 0.919672 |
| TRINITY_DN37325_c0_g1_i10 | turquoise | 0.9192692 |
| TRINITY_DN13647_c0_g2_i1 | turquoise | 0.9192562 |
| TRINITY_DN33937_c0_g1_i9 | turquoise | 0.9191932 |
| TRINITY_DN37792_c2_g1_i7 | turquoise | 0.9190911 |
| TRINITY_DN817_c0_g2_i1 | turquoise | 0.9190079 |
| TRINITY_DN52953_c0_g1_i1 | turquoise | 0.9189383 |
| TRINITY_DN61942_c0_g1_i1 | turquoise | 0.9188968 |
| TRINITY_DN56964_c0_g1_i1 | turquoise | 0.918823 |
| TRINITY_DN33657_c0_g1_i2 | turquoise | 0.9186701 |
| TRINITY_DN19616_c0_g2_i1 | turquoise | 0.9186486 |
| TRINITY_DN8489_c0_g1_i1 | turquoise | 0.9185653 |
| TRINITY_DN26699_c0_g1_i1 | turquoise | 0.9184813 |
| TRINITY_DN70439_c0_g1_i1 | turquoise | 0.9184625 |
| TRINITY_DN3249_c0_g1_i1 | turquoise | 0.9183928 |
| TRINITY_DN36867_c0_g2_i6 | turquoise | 0.9183503 |
| TRINITY_DN62605_c0_g1_i1 | turquoise | 0.918157 |
| TRINITY_DN24187_c0_g1_i1 | turquoise | 0.9181185 |
| TRINITY_DN9877_c0_g1_i1 | turquoise | 0.9179433 |
| TRINITY_DN67321_c0_g1_i1 | turquoise | 0.9179432 |
| TRINITY_DN26621_c0_g2_i1 | turquoise | 0.9178841 |
| TRINITY_DN34053_c1_g2_i3 | turquoise | 0.9177592 |
| TRINITY_DN39331_c7_g7_i2 | turquoise | 0.917746 |
| TRINITY_DN32806_c0_g2_i4 | turquoise | 0.917659 |
| TRINITY_DN39145_c0_g1_i1 | turquoise | 0.9176005 |
| TRINITY_DN35463_c1_g1_i2 | turquoise | 0.9174617 |
| TRINITY_DN39309_c2_g12_i2 | turquoise | 0.917255 |
| TRINITY_DN12402_c0_g1_i1 | turquoise | 0.9172295 |
| TRINITY_DN34433_c0_g1_i9 | turquoise | 0.9171346 |
| TRINITY_DN40352_c0_g1_i1 | turquoise | 0.9171181 |
| TRINITY_DN30313_c1_g6_i1 | turquoise | 0.9171025 |
| TRINITY_DN35790_c0_g1_i3 | turquoise | 0.9170187 |
| TRINITY_DN13236_c0_g1_i1 | turquoise | 0.9170021 |
| TRINITY_DN36031_c0_g1_i1 | turquoise | 0.9169395 |
| TRINITY_DN21819_c0_g1_i1 | turquoise | 0.9168889 |
| TRINITY_DN32090_c1_g2_i2 | turquoise | 0.9168797 |
| TRINITY_DN33007_c2_g1_i1 | turquoise | 0.9165935 |
| TRINITY_DN51014_c0_g1_i1 | turquoise | 0.9165289 |
| TRINITY_DN35866_c3_g1_i9 | turquoise | 0.9164063 |
| TRINITY_DN34825_c0_g2_i5 | turquoise | 0.9163525 |
| TRINITY_DN33632_c0_g3_i3 | turquoise | 0.9163327 |
| TRINITY_DN29865_c0_g1_i1 | turquoise | 0.9162159 |
| TRINITY_DN29716_c0_g1_i3 | turquoise | 0.9162061 |
| TRINITY_DN37360_c0_g6_i1 | turquoise | 0.9161468 |
| TRINITY_DN28317_c0_g1_i3 | turquoise | 0.9159376 |
| TRINITY_DN38717_c1_g3_i1 | turquoise | 0.9159287 |
| TRINITY_DN38667_c0_g3_i1 | turquoise | 0.915871 |
| TRINITY_DN37880_c1_g2_i2 | turquoise | 0.9158461 |
| TRINITY_DN36539_c0_g1_i5 | turquoise | 0.9157991 |
| TRINITY_DN35609_c0_g2_i4 | turquoise | 0.9157205 |
| TRINITY_DN18721_c0_g1_i1 | turquoise | 0.9156703 |
| TRINITY_DN47344_c0_g1_i1 | turquoise | 0.9155883 |
| TRINITY_DN28242_c0_g1_i1 | turquoise | 0.9155367 |
| TRINITY_DN35201_c1_g1_i1 | turquoise | 0.915506 |
| TRINITY_DN56329_c0_g1_i1 | turquoise | 0.9154994 |
| TRINITY_DN7150_c0_g1_i1 | turquoise | 0.9154843 |
| TRINITY_DN16227_c0_g1_i1 | turquoise | 0.9154552 |
| TRINITY_DN39837_c0_g1_i1 | turquoise | 0.9153863 |
| TRINITY_DN4645_c0_g2_i1 | turquoise | 0.9153753 |
| TRINITY_DN744_c0_g1_i1 | turquoise | 0.9153395 |
| TRINITY_DN31717_c0_g1_i3 | turquoise | 0.9152039 |
| TRINITY_DN38549_c1_g2_i1 | turquoise | 0.9151727 |
| TRINITY_DN33953_c0_g2_i1 | turquoise | 0.9151497 |
| TRINITY_DN9187_c0_g1_i1 | turquoise | 0.9151019 |
| TRINITY_DN36794_c0_g1_i11 | turquoise | 0.9150922 |
| TRINITY_DN26957_c0_g1_i1 | turquoise | 0.9150141 |
| TRINITY_DN37041_c0_g4_i3 | turquoise | 0.9149611 |
| TRINITY_DN17938_c0_g3_i1 | turquoise | 0.9149364 |
| TRINITY_DN38916_c0_g1_i11 | turquoise | 0.9147691 |
| TRINITY_DN22467_c0_g1_i1 | turquoise | 0.914683 |
| TRINITY_DN37773_c0_g1_i7 | turquoise | 0.9145429 |
| TRINITY_DN9114_c0_g2_i1 | turquoise | 0.9144478 |
| TRINITY_DN36080_c0_g1_i11 | turquoise | 0.914434 |
| TRINITY_DN36772_c0_g1_i1 | turquoise | 0.9144153 |
| TRINITY_DN36787_c0_g1_i3 | turquoise | 0.9142671 |
| TRINITY_DN49138_c0_g1_i1 | turquoise | 0.9141621 |
| TRINITY_DN29078_c0_g1_i5 | turquoise | 0.9141585 |
| TRINITY_DN31646_c0_g1_i1 | turquoise | 0.9141197 |
| TRINITY_DN67490_c0_g1_i1 | turquoise | 0.9139586 |
| TRINITY_DN33223_c0_g1_i1 | turquoise | 0.9138144 |
| TRINITY_DN36549_c0_g2_i5 | turquoise | 0.9138106 |
| TRINITY_DN56257_c0_g1_i1 | turquoise | 0.9137959 |
| TRINITY_DN25950_c0_g1_i1 | turquoise | 0.9137012 |
| TRINITY_DN27203_c0_g1_i9 | turquoise | 0.9136739 |
| TRINITY_DN38695_c0_g2_i9 | turquoise | 0.9135228 |
| TRINITY_DN36117_c0_g1_i10 | turquoise | 0.9135183 |
| TRINITY_DN67428_c0_g1_i1 | turquoise | 0.9134863 |
| TRINITY_DN46545_c0_g1_i1 | turquoise | 0.9134781 |
| TRINITY_DN39671_c0_g1_i1 | turquoise | 0.9134527 |
| TRINITY_DN32378_c0_g1_i1 | turquoise | 0.9134127 |
| TRINITY_DN68162_c0_g1_i1 | turquoise | 0.9133959 |
| TRINITY_DN34599_c0_g1_i7 | turquoise | 0.9133449 |
| TRINITY_DN36530_c1_g2_i9 | turquoise | 0.9132942 |
| TRINITY_DN27159_c0_g1_i2 | turquoise | 0.9132504 |
| TRINITY_DN15118_c0_g2_i1 | turquoise | 0.9132374 |
| TRINITY_DN25981_c0_g2_i1 | turquoise | 0.9131408 |
| TRINITY_DN32190_c0_g1_i3 | turquoise | 0.9130198 |
| TRINITY_DN4493_c0_g1_i1 | turquoise | 0.913001 |
| TRINITY_DN33517_c1_g5_i10 | turquoise | 0.9129775 |
| TRINITY_DN31758_c1_g3_i1 | turquoise | 0.9129359 |
| TRINITY_DN35236_c0_g1_i2 | turquoise | 0.9128956 |
| TRINITY_DN37360_c0_g4_i4 | turquoise | 0.9128671 |
| TRINITY_DN17362_c0_g1_i1 | turquoise | 0.9126148 |
| TRINITY_DN34037_c1_g2_i9 | turquoise | 0.912562 |
| TRINITY_DN29667_c3_g3_i2 | turquoise | 0.912515 |
| TRINITY_DN16993_c0_g2_i1 | turquoise | 0.9123644 |
| TRINITY_DN52114_c0_g1_i1 | turquoise | 0.9123579 |
| TRINITY_DN17660_c0_g1_i1 | turquoise | 0.9122483 |
| TRINITY_DN2277_c0_g2_i1 | turquoise | 0.9122414 |
| TRINITY_DN25302_c0_g1_i4 | turquoise | 0.9121683 |
| TRINITY_DN33870_c0_g1_i7 | turquoise | 0.9121429 |
| TRINITY_DN35983_c1_g4_i5 | turquoise | 0.9121074 |
| TRINITY_DN37735_c0_g3_i2 | turquoise | 0.9120679 |
| TRINITY_DN35666_c0_g1_i1 | turquoise | 0.9118931 |
| TRINITY_DN38726_c1_g2_i10 | turquoise | 0.911856 |
| TRINITY_DN27351_c0_g1_i1 | turquoise | 0.9117641 |
| TRINITY_DN37600_c1_g3_i1 | turquoise | 0.9116226 |
| TRINITY_DN33005_c0_g5_i1 | turquoise | 0.9116023 |
| TRINITY_DN28378_c0_g1_i1 | turquoise | 0.911427 |
| TRINITY_DN28501_c1_g2_i1 | turquoise | 0.9112743 |
| TRINITY_DN32871_c0_g2_i1 | turquoise | 0.911078 |
| TRINITY_DN37141_c0_g1_i2 | turquoise | 0.911019 |
| TRINITY_DN36103_c0_g2_i2 | turquoise | 0.9109718 |
| TRINITY_DN13593_c0_g1_i1 | turquoise | 0.9109085 |
| TRINITY_DN33709_c1_g1_i1 | turquoise | 0.9108298 |
| TRINITY_DN37654_c1_g2_i6 | turquoise | 0.9108174 |
| TRINITY_DN15791_c0_g1_i1 | turquoise | 0.910781 |
| TRINITY_DN5261_c0_g2_i1 | turquoise | 0.9106159 |
| TRINITY_DN32268_c0_g1_i3 | turquoise | 0.9106011 |
| TRINITY_DN36476_c1_g1_i2 | turquoise | 0.9105564 |
| TRINITY_DN34053_c0_g1_i1 | turquoise | 0.9105268 |
| TRINITY_DN33397_c2_g2_i3 | turquoise | 0.9103641 |
| TRINITY_DN67723_c0_g1_i1 | turquoise | 0.9103597 |
| TRINITY_DN38781_c0_g4_i8 | turquoise | 0.9103253 |
| TRINITY_DN33792_c1_g1_i1 | turquoise | 0.9103182 |
| TRINITY_DN38820_c0_g3_i1 | turquoise | 0.9103055 |
| TRINITY_DN39261_c3_g7_i3 | turquoise | 0.9100087 |
| TRINITY_DN62413_c0_g1_i1 | turquoise | 0.9099261 |
| TRINITY_DN35876_c0_g1_i3 | turquoise | 0.9099186 |
| TRINITY_DN56405_c0_g1_i1 | turquoise | 0.9095993 |
| TRINITY_DN33841_c1_g1_i2 | turquoise | 0.9095599 |
| TRINITY_DN30468_c0_g3_i4 | turquoise | 0.9095407 |
| TRINITY_DN28815_c0_g2_i4 | turquoise | 0.9095152 |
| TRINITY_DN37836_c1_g4_i1 | turquoise | 0.9094777 |
| TRINITY_DN37869_c2_g1_i1 | turquoise | 0.9093762 |
| TRINITY_DN32378_c0_g2_i1 | turquoise | 0.9093054 |
| TRINITY_DN9787_c0_g1_i1 | turquoise | 0.9091997 |
| TRINITY_DN34021_c0_g2_i9 | turquoise | 0.9091304 |
| TRINITY_DN37885_c0_g3_i1 | turquoise | 0.9090486 |
| TRINITY_DN16758_c0_g1_i1 | turquoise | 0.9090206 |
| TRINITY_DN5389_c0_g1_i1 | turquoise | 0.9089092 |
| TRINITY_DN34566_c0_g1_i2 | turquoise | 0.9088729 |
| TRINITY_DN36218_c0_g2_i6 | turquoise | 0.9088719 |
| TRINITY_DN37950_c1_g3_i11 | turquoise | 0.9087519 |
| TRINITY_DN38736_c0_g1_i8 | turquoise | 0.9087172 |
| TRINITY_DN56290_c0_g1_i1 | turquoise | 0.908652 |
| TRINITY_DN11458_c0_g1_i1 | turquoise | 0.9086424 |
| TRINITY_DN65474_c0_g1_i1 | turquoise | 0.908624 |
| TRINITY_DN37626_c1_g2_i8 | turquoise | 0.9085857 |
| TRINITY_DN39969_c0_g1_i1 | turquoise | 0.9084631 |
| TRINITY_DN31498_c0_g1_i2 | turquoise | 0.908418 |
| TRINITY_DN35962_c0_g8_i4 | turquoise | 0.9084 |
| TRINITY_DN19300_c0_g2_i1 | turquoise | 0.9080463 |
| TRINITY_DN36695_c0_g1_i11 | turquoise | 0.9079841 |
| TRINITY_DN31307_c0_g1_i1 | turquoise | 0.9078964 |
| TRINITY_DN40179_c0_g1_i1 | turquoise | 0.9078809 |
| TRINITY_DN24388_c0_g1_i1 | turquoise | 0.9077429 |
| TRINITY_DN33747_c1_g1_i6 | turquoise | 0.9077209 |
| TRINITY_DN67423_c0_g1_i1 | turquoise | 0.9076572 |
| TRINITY_DN35015_c0_g5_i1 | turquoise | 0.9076415 |
| TRINITY_DN58087_c0_g1_i1 | turquoise | 0.9073625 |
| TRINITY_DN19327_c0_g2_i1 | turquoise | 0.9072974 |
| TRINITY_DN29679_c0_g1_i1 | turquoise | 0.9072961 |
| TRINITY_DN35209_c1_g1_i1 | turquoise | 0.9072095 |
| TRINITY_DN38102_c0_g3_i2 | turquoise | 0.907113 |
| TRINITY_DN28206_c0_g3_i1 | turquoise | 0.9068663 |
| TRINITY_DN69587_c0_g1_i1 | turquoise | 0.9066749 |
| TRINITY_DN36650_c0_g3_i10 | turquoise | 0.9064389 |
| TRINITY_DN34903_c1_g1_i5 | turquoise | 0.9063502 |
| TRINITY_DN4344_c0_g1_i1 | turquoise | 0.9061243 |
| TRINITY_DN67904_c0_g1_i1 | turquoise | 0.9059816 |
| TRINITY_DN69238_c0_g1_i1 | turquoise | 0.9059103 |
| TRINITY_DN38316_c0_g3_i4 | turquoise | 0.9058817 |
| TRINITY_DN19135_c0_g2_i1 | turquoise | 0.9058216 |
| TRINITY_DN4516_c0_g1_i1 | turquoise | 0.9057894 |
| TRINITY_DN47461_c0_g1_i1 | turquoise | 0.905689 |
| TRINITY_DN17611_c0_g2_i1 | turquoise | 0.9056771 |
| TRINITY_DN33332_c0_g1_i2 | turquoise | 0.9056285 |
| TRINITY_DN41140_c0_g1_i1 | turquoise | 0.9053401 |
| TRINITY_DN28564_c0_g2_i2 | turquoise | 0.9052414 |
| TRINITY_DN33559_c0_g2_i14 | turquoise | 0.9051815 |
| TRINITY_DN32085_c2_g1_i5 | turquoise | 0.9050911 |
| TRINITY_DN37494_c0_g3_i8 | turquoise | 0.9050152 |
| TRINITY_DN14130_c0_g1_i1 | turquoise | 0.9049802 |
| TRINITY_DN40746_c0_g1_i1 | turquoise | 0.9049786 |
| TRINITY_DN23780_c0_g1_i1 | turquoise | 0.9049421 |
| TRINITY_DN45111_c0_g1_i1 | turquoise | 0.9048909 |
| TRINITY_DN717_c0_g1_i1 | turquoise | 0.9048593 |
| TRINITY_DN35689_c0_g1_i2 | turquoise | 0.9044875 |
| TRINITY_DN30245_c0_g2_i9 | turquoise | 0.9044234 |
| TRINITY_DN39152_c1_g1_i2 | turquoise | 0.9044106 |
| TRINITY_DN35576_c0_g1_i10 | turquoise | 0.904314 |
| TRINITY_DN38139_c0_g3_i6 | turquoise | 0.9041556 |
| TRINITY_DN57814_c0_g1_i1 | turquoise | 0.9040322 |
| TRINITY_DN39316_c1_g4_i1 | turquoise | 0.9039942 |
| TRINITY_DN1685_c0_g1_i1 | turquoise | 0.9039691 |
| TRINITY_DN32494_c1_g1_i2 | turquoise | 0.9037725 |
| TRINITY_DN46636_c0_g1_i1 | turquoise | 0.9034725 |
| TRINITY_DN16257_c0_g2_i1 | turquoise | 0.9034079 |
| TRINITY_DN39268_c1_g1_i1 | turquoise | 0.9033176 |
| TRINITY_DN36581_c3_g6_i1 | turquoise | 0.9032201 |
| TRINITY_DN21431_c0_g1_i1 | turquoise | 0.9031145 |
| TRINITY_DN19859_c0_g1_i1 | turquoise | 0.9029371 |
| TRINITY_DN45593_c0_g1_i1 | turquoise | 0.9027967 |
| TRINITY_DN37064_c2_g1_i1 | turquoise | 0.9026733 |
| TRINITY_DN38686_c1_g3_i1 | turquoise | 0.9026409 |
| TRINITY_DN45080_c0_g1_i1 | turquoise | 0.9025795 |
| TRINITY_DN9274_c0_g1_i1 | turquoise | 0.9024109 |
| TRINITY_DN33920_c1_g1_i9 | turquoise | 0.9023802 |
| TRINITY_DN337_c0_g1_i1 | turquoise | 0.9023533 |
| TRINITY_DN39044_c2_g3_i6 | turquoise | 0.9023369 |
| TRINITY_DN17054_c0_g1_i1 | turquoise | 0.9022018 |
| TRINITY_DN35804_c0_g2_i6 | turquoise | 0.9021624 |
| TRINITY_DN37739_c0_g2_i1 | turquoise | 0.9021609 |
| TRINITY_DN23015_c0_g1_i1 | turquoise | 0.9020577 |
| TRINITY_DN57281_c0_g1_i1 | turquoise | 0.9020471 |
| TRINITY_DN17485_c0_g1_i1 | turquoise | 0.9018842 |
| TRINITY_DN28175_c0_g2_i1 | turquoise | 0.9018353 |
| TRINITY_DN3579_c0_g1_i1 | turquoise | 0.9017751 |
| TRINITY_DN30019_c0_g4_i3 | turquoise | 0.9017009 |
| TRINITY_DN37534_c0_g2_i1 | turquoise | 0.9015948 |
| TRINITY_DN22863_c0_g1_i1 | turquoise | 0.9015777 |
| TRINITY_DN38383_c0_g1_i11 | turquoise | 0.9015477 |
| TRINITY_DN25105_c0_g1_i2 | turquoise | 0.901445 |
| TRINITY_DN32762_c0_g1_i5 | turquoise | 0.9013882 |
| TRINITY_DN4506_c0_g1_i1 | turquoise | 0.9013722 |
| TRINITY_DN5259_c0_g1_i1 | turquoise | 0.9013524 |
| TRINITY_DN26734_c0_g1_i1 | turquoise | 0.9013307 |
| TRINITY_DN37734_c2_g1_i3 | turquoise | 0.9013204 |
| TRINITY_DN63698_c0_g1_i1 | turquoise | 0.9013078 |
| TRINITY_DN36571_c0_g1_i24 | turquoise | 0.9012642 |
| TRINITY_DN44876_c0_g1_i1 | turquoise | 0.9011822 |
| TRINITY_DN32483_c0_g4_i2 | turquoise | 0.9010488 |
| TRINITY_DN36804_c0_g1_i1 | turquoise | 0.900972 |
| TRINITY_DN37281_c0_g4_i1 | turquoise | 0.9009305 |
| TRINITY_DN38933_c0_g1_i6 | turquoise | 0.9008898 |
| TRINITY_DN34538_c1_g6_i1 | turquoise | 0.9008891 |
| TRINITY_DN62353_c0_g1_i1 | turquoise | 0.900876 |
| TRINITY_DN46885_c0_g1_i1 | turquoise | 0.9008468 |
| TRINITY_DN18737_c0_g1_i1 | turquoise | 0.9006808 |
| TRINITY_DN595_c0_g1_i1 | turquoise | 0.9006765 |
| TRINITY_DN38904_c1_g1_i1 | turquoise | 0.9006388 |
| TRINITY_DN29670_c0_g3_i2 | turquoise | 0.9005234 |
| TRINITY_DN40956_c0_g1_i1 | turquoise | 0.9004979 |
| TRINITY_DN24411_c0_g2_i1 | turquoise | 0.9004396 |
| TRINITY_DN32003_c1_g1_i1 | turquoise | 0.9003793 |
| TRINITY_DN17314_c0_g2_i1 | turquoise | 0.9003108 |
| TRINITY_DN39293_c3_g3_i4 | turquoise | 0.9002043 |
| TRINITY_DN27680_c0_g1_i3 | turquoise | 0.900001 |
| TRINITY_DN36979_c0_g4_i9 | turquoise | 0.8998493 |
| TRINITY_DN18515_c0_g1_i1 | turquoise | 0.8998084 |
| TRINITY_DN35499_c0_g1_i2 | turquoise | 0.8996392 |
| TRINITY_DN26166_c0_g1_i3 | turquoise | 0.8996368 |
| TRINITY_DN37075_c1_g2_i2 | turquoise | 0.8996104 |
| TRINITY_DN4203_c0_g1_i1 | turquoise | 0.8995131 |
| TRINITY_DN25970_c0_g1_i3 | turquoise | 0.8993011 |
| TRINITY_DN33872_c1_g2_i4 | turquoise | 0.8991984 |
| TRINITY_DN25011_c0_g3_i1 | turquoise | 0.899163 |
| TRINITY_DN36142_c2_g1_i6 | turquoise | 0.899093 |
| TRINITY_DN38422_c1_g1_i5 | turquoise | 0.8990656 |
| TRINITY_DN31942_c0_g1_i5 | turquoise | 0.8989329 |
| TRINITY_DN35718_c0_g1_i4 | turquoise | 0.8989209 |
| TRINITY_DN6030_c0_g2_i1 | turquoise | 0.8987871 |
| TRINITY_DN33104_c1_g1_i4 | turquoise | 0.8987402 |
| TRINITY_DN45203_c0_g1_i1 | turquoise | 0.8987393 |
| TRINITY_DN39815_c0_g1_i1 | turquoise | 0.8984246 |
| TRINITY_DN35941_c0_g1_i3 | turquoise | 0.8982907 |
| TRINITY_DN34234_c0_g2_i1 | turquoise | 0.8981918 |
| TRINITY_DN6157_c0_g1_i1 | turquoise | 0.8981322 |
| TRINITY_DN37086_c0_g1_i7 | turquoise | 0.8981173 |
| TRINITY_DN51172_c0_g1_i1 | turquoise | 0.8981149 |
| TRINITY_DN11773_c0_g1_i1 | turquoise | 0.8980934 |
| TRINITY_DN38471_c0_g1_i11 | turquoise | 0.8980348 |
| TRINITY_DN33286_c0_g1_i5 | turquoise | 0.8979453 |
| TRINITY_DN37630_c1_g1_i2 | turquoise | 0.8978135 |
| TRINITY_DN36856_c0_g2_i2 | turquoise | 0.8977976 |
| TRINITY_DN17684_c0_g1_i1 | turquoise | 0.8975425 |
| TRINITY_DN38904_c1_g3_i7 | turquoise | 0.89754 |
| TRINITY_DN50823_c0_g1_i1 | turquoise | 0.8975304 |
| TRINITY_DN39109_c0_g1_i5 | turquoise | 0.8974789 |
| TRINITY_DN37343_c0_g1_i2 | turquoise | 0.8974757 |
| TRINITY_DN27171_c0_g1_i1 | turquoise | 0.8974308 |
| TRINITY_DN50990_c0_g1_i1 | turquoise | 0.897426 |
| TRINITY_DN30076_c0_g3_i3 | turquoise | 0.8973206 |
| TRINITY_DN28979_c0_g1_i1 | turquoise | 0.897317 |
| TRINITY_DN5151_c0_g1_i1 | turquoise | 0.8971872 |
| TRINITY_DN38268_c0_g7_i12 | turquoise | 0.8971254 |
| TRINITY_DN42494_c0_g1_i1 | turquoise | 0.8970797 |
[truncated: 222,664 more chars]
