# Supplementary material for: Comparative transcriptomic analysis reveals the cold acclimation during chilling stress in sensitive and resistant passion fruit (Passiflora edulis) cultivars
Source: PeerJ. 2021 Mar 3;9:e10977. doi: 10.7717/peerj.10977 (PMC7936571; doi:10.7717/peerj.10977)
Supplement: Supplemental Information 6 [file peerj-09-10977-s006.docx]

**Supplementary table 6 GO enrichment analysis in brown module**

**1-BP**

| GO ID | Term | Annotated nuigene number | Significant unigene number | Expected | Classic Fisher | FDR |
| --- | --- | --- | --- | --- | --- | --- |
| GO:0015074 | DNA integration | 112 | 16 | 3.51 | 3.70E-07 | 0.00035668 |
| GO:0006032 | chitin catabolic process | 27 | 7 | 0.85 | 1.40E-05 | 0.003052667 |
| GO:0046348 | amino sugar catabolic process | 27 | 7 | 0.85 | 1.40E-05 | 0.003052667 |
| GO:1901072 | glucosamine-containing compound cataboli... | 27 | 7 | 0.85 | 1.40E-05 | 0.003052667 |
| GO:0006030 | chitin metabolic process | 28 | 7 | 0.88 | 1.90E-05 | 0.003052667 |
| GO:1901071 | glucosamine-containing compound metaboli... | 28 | 7 | 0.88 | 1.90E-05 | 0.003052667 |
| GO:0016998 | cell wall macromolecule catabolic proces... | 31 | 7 | 0.97 | 3.80E-05 | 0.004579 |
| GO:0044036 | cell wall macromolecule metabolic proces... | 31 | 7 | 0.97 | 3.80E-05 | 0.004579 |
| GO:0006040 | amino sugar metabolic process | 33 | 7 | 1.03 | 5.90E-05 | 0.006319556 |
| GO:0006026 | aminoglycan catabolic process | 37 | 7 | 1.16 | 0.00013 | 0.012532 |
| GO:0006022 | aminoglycan metabolic process | 43 | 7 | 1.35 | 0.00034 | 0.029796364 |
| GO:1901136 | carbohydrate derivative catabolic proces... | 45 | 7 | 1.41 | 0.00045 | 0.03615 |
| GO:0006771 | riboflavin metabolic process | 15 | 4 | 0.47 | 0.00098 | 0.059045 |
| GO:0009231 | riboflavin biosynthetic process | 15 | 4 | 0.47 | 0.00098 | 0.059045 |
| GO:0042726 | flavin-containing compound metabolic pro... | 15 | 4 | 0.47 | 0.00098 | 0.059045 |
| GO:0042727 | flavin-containing compound biosynthetic ... | 15 | 4 | 0.47 | 0.00098 | 0.059045 |
| GO:0006259 | DNA metabolic process | 328 | 21 | 10.28 | 0.0015 | 0.085058824 |
| GO:0016310 | phosphorylation | 1154 | 52 | 36.16 | 0.00414 | 0.22172 |
| GO:0006468 | protein phosphorylation | 1052 | 48 | 32.97 | 0.00466 | 0.236433684 |
| GO:0009110 | vitamin biosynthetic process | 37 | 5 | 1.16 | 0.0056 | 0.257066667 |
| GO:0042364 | water-soluble vitamin biosynthetic proce... | 37 | 5 | 1.16 | 0.0056 | 0.257066667 |
| GO:0006766 | vitamin metabolic process | 38 | 5 | 1.19 | 0.00628 | 0.263213913 |
| GO:0006767 | water-soluble vitamin metabolic process | 38 | 5 | 1.19 | 0.00628 | 0.263213913 |
| GO:0006950 | response to stress | 237 | 15 | 7.43 | 0.00763 | 0.306471667 |
| GO:0006952 | defense response | 57 | 6 | 1.79 | 0.00855 | 0.31812 |
| GO:0006694 | steroid biosynthetic process | 14 | 3 | 0.44 | 0.00858 | 0.31812 |
| GO:0006425 | glutaminyl-tRNA aminoacylation | 5 | 2 | 0.16 | 0.00919 | 0.328117037 |
| GO:0008202 | steroid metabolic process | 15 | 3 | 0.47 | 0.01048 | 0.360811429 |
| GO:0050896 | response to stimulus | 761 | 35 | 23.85 | 0.01369 | 0.448581333 |
| GO:0044260 | cellular macromolecule metabolic process | 3578 | 131 | 112.12 | 0.01396 | 0.448581333 |
| GO:0071554 | cell wall organization or biogenesis | 102 | 8 | 3.2 | 0.01458 | 0.453390968 |
| GO:0009607 | response to biotic stimulus | 8 | 2 | 0.25 | 0.02419 | 0.666261714 |
| GO:0043207 | response to external biotic stimulus | 8 | 2 | 0.25 | 0.02419 | 0.666261714 |
| GO:0051707 | response to other organism | 8 | 2 | 0.25 | 0.02419 | 0.666261714 |
| GO:0098542 | defense response to other organism | 8 | 2 | 0.25 | 0.02419 | 0.666261714 |
| GO:0032268 | regulation of cellular protein metabolic... | 21 | 3 | 0.66 | 0.02668 | 0.699129524 |
| GO:0051246 | regulation of protein metabolic process | 22 | 3 | 0.69 | 0.03019 | 0.699129524 |
| GO:0015748 | organophosphate ester transport | 9 | 2 | 0.28 | 0.03046 | 0.699129524 |
| GO:0017148 | negative regulation of translation | 9 | 2 | 0.28 | 0.03046 | 0.699129524 |
| GO:0032269 | negative regulation of cellular protein ... | 9 | 2 | 0.28 | 0.03046 | 0.699129524 |
| GO:0034249 | negative regulation of cellular amide me... | 9 | 2 | 0.28 | 0.03046 | 0.699129524 |
| GO:0051248 | negative regulation of protein metabolic... | 9 | 2 | 0.28 | 0.03046 | 0.699129524 |
| GO:0006796 | phosphate-containing compound metabolic ... | 1410 | 56 | 44.18 | 0.03366 | 0.754610233 |
| GO:0006793 | phosphorus metabolic process | 1417 | 56 | 44.4 | 0.03656 | 0.800996364 |
| GO:0006555 | methionine metabolic process | 12 | 2 | 0.38 | 0.05251 | 1 |
| GO:0044237 | cellular metabolic process | 4582 | 158 | 143.58 | 0.05277 | 1 |
| GO:0071704 | organic substance metabolic process | 5449 | 185 | 170.75 | 0.05348 | 1 |
| GO:0009057 | macromolecule catabolic process | 156 | 9 | 4.89 | 0.05655 | 1 |
| GO:1901565 | organonitrogen compound catabolic proces... | 162 | 9 | 5.08 | 0.06838 | 1 |
| GO:0006464 | cellular protein modification process | 1357 | 52 | 42.52 | 0.06864 | 1 |
| GO:0036211 | protein modification process | 1357 | 52 | 42.52 | 0.06864 | 1 |
| GO:0030243 | cellulose metabolic process | 50 | 4 | 1.57 | 0.07074 | 1 |
| GO:0030244 | cellulose biosynthetic process | 50 | 4 | 1.57 | 0.07074 | 1 |
| GO:0044267 | cellular protein metabolic process | 2026 | 74 | 63.49 | 0.07756 | 1 |
| GO:0006417 | regulation of translation | 15 | 2 | 0.47 | 0.07859 | 1 |
| GO:0010608 | posttranscriptional regulation of gene e... | 15 | 2 | 0.47 | 0.07859 | 1 |
| GO:0034248 | regulation of cellular amide metabolic p... | 15 | 2 | 0.47 | 0.07859 | 1 |
| GO:0008152 | metabolic process | 7079 | 233 | 221.83 | 0.08119 | 1 |
| GO:0010558 | negative regulation of macromolecule bio... | 16 | 2 | 0.5 | 0.08801 | 1 |
| GO:0031324 | negative regulation of cellular metaboli... | 16 | 2 | 0.5 | 0.08801 | 1 |
| GO:0031327 | negative regulation of cellular biosynth... | 16 | 2 | 0.5 | 0.08801 | 1 |
| GO:0051172 | negative regulation of nitrogen compound... | 16 | 2 | 0.5 | 0.08801 | 1 |
| GO:2000113 | negative regulation of cellular macromol... | 16 | 2 | 0.5 | 0.08801 | 1 |
| GO:0009890 | negative regulation of biosynthetic proc... | 17 | 2 | 0.53 | 0.09776 | 1 |
| GO:0006096 | glycolytic process | 57 | 4 | 1.79 | 0.10281 | 1 |
| GO:0006165 | nucleoside diphosphate phosphorylation | 57 | 4 | 1.79 | 0.10281 | 1 |
| GO:0006757 | ATP generation from ADP | 57 | 4 | 1.79 | 0.10281 | 1 |
| GO:0009132 | nucleoside diphosphate metabolic process | 57 | 4 | 1.79 | 0.10281 | 1 |
| GO:0009135 | purine nucleoside diphosphate metabolic ... | 57 | 4 | 1.79 | 0.10281 | 1 |
| GO:0009179 | purine ribonucleoside diphosphate metabo... | 57 | 4 | 1.79 | 0.10281 | 1 |
| GO:0009185 | ribonucleoside diphosphate metabolic pro... | 57 | 4 | 1.79 | 0.10281 | 1 |
| GO:0046031 | ADP metabolic process | 57 | 4 | 1.79 | 0.10281 | 1 |
| GO:0046939 | nucleotide phosphorylation | 57 | 4 | 1.79 | 0.10281 | 1 |
| GO:0043412 | macromolecule modification | 1404 | 52 | 44 | 0.1087 | 1 |
| GO:1901575 | organic substance catabolic process | 288 | 13 | 9.02 | 0.11927 | 1 |
| GO:0009987 | cellular process | 5537 | 184 | 173.51 | 0.12057 | 1 |
| GO:0006629 | lipid metabolic process | 343 | 15 | 10.75 | 0.12081 | 1 |
| GO:0051704 | multi-organism process | 61 | 4 | 1.91 | 0.12365 | 1 |
| GO:0000096 | sulfur amino acid metabolic process | 20 | 2 | 0.63 | 0.12859 | 1 |
| GO:0009605 | response to external stimulus | 20 | 2 | 0.63 | 0.12859 | 1 |
| GO:0016311 | dephosphorylation | 20 | 2 | 0.63 | 0.12859 | 1 |
| GO:0051716 | cellular response to stimulus | 486 | 20 | 15.23 | 0.12866 | 1 |
| GO:0006090 | pyruvate metabolic process | 62 | 4 | 1.94 | 0.12912 | 1 |
| GO:0043170 | macromolecule metabolic process | 3924 | 133 | 122.96 | 0.12948 | 1 |
| GO:0009056 | catabolic process | 296 | 13 | 9.28 | 0.13836 | 1 |
| GO:0005975 | carbohydrate metabolic process | 784 | 30 | 24.57 | 0.14626 | 1 |
| GO:0006073 | cellular glucan metabolic process | 89 | 5 | 2.79 | 0.1467 | 1 |
| GO:0044042 | glucan metabolic process | 89 | 5 | 2.79 | 0.1467 | 1 |
| GO:0000079 | regulation of cyclin-dependent protein s... | 5 | 1 | 0.16 | 0.14719 | 1 |
| GO:0001932 | regulation of protein phosphorylation | 5 | 1 | 0.16 | 0.14719 | 1 |
| GO:0007205 | protein kinase C-activating G-protein co... | 5 | 1 | 0.16 | 0.14719 | 1 |
| GO:0015689 | molybdate ion transport | 5 | 1 | 0.16 | 0.14719 | 1 |
| GO:0043549 | regulation of kinase activity | 5 | 1 | 0.16 | 0.14719 | 1 |
| GO:0045859 | regulation of protein kinase activity | 5 | 1 | 0.16 | 0.14719 | 1 |
| GO:0051338 | regulation of transferase activity | 5 | 1 | 0.16 | 0.14719 | 1 |
| GO:0071900 | regulation of protein serine/threonine k... | 5 | 1 | 0.16 | 0.14719 | 1 |
| GO:1904029 | regulation of cyclin-dependent protein k... | 5 | 1 | 0.16 | 0.14719 | 1 |
| GO:0032787 | monocarboxylic acid metabolic process | 167 | 8 | 5.23 | 0.15383 | 1 |
| GO:0044283 | small molecule biosynthetic process | 195 | 9 | 6.11 | 0.15906 | 1 |
| GO:0031399 | regulation of protein modification proce... | 6 | 1 | 0.19 | 0.17393 | 1 |
| GO:0042325 | regulation of phosphorylation | 6 | 1 | 0.19 | 0.17393 | 1 |
| GO:0080134 | regulation of response to stress | 6 | 1 | 0.19 | 0.17393 | 1 |
| GO:0006979 | response to oxidative stress | 70 | 4 | 2.19 | 0.17623 | 1 |
| GO:0051273 | beta-glucan metabolic process | 70 | 4 | 2.19 | 0.17623 | 1 |
| GO:0051274 | beta-glucan biosynthetic process | 70 | 4 | 2.19 | 0.17623 | 1 |
| GO:0007154 | cell communication | 425 | 17 | 13.32 | 0.18017 | 1 |
| GO:0009250 | glucan biosynthetic process | 71 | 4 | 2.22 | 0.18249 | 1 |
| GO:0044264 | cellular polysaccharide metabolic proces... | 96 | 5 | 3.01 | 0.18258 | 1 |
| GO:0015979 | photosynthesis | 48 | 3 | 1.5 | 0.18984 | 1 |
| GO:0015931 | nucleobase-containing compound transport | 26 | 2 | 0.81 | 0.1953 | 1 |
| GO:0071804 | cellular potassium ion transport | 26 | 2 | 0.81 | 0.1953 | 1 |
| GO:0071805 | potassium ion transmembrane transport | 26 | 2 | 0.81 | 0.1953 | 1 |
| GO:0044248 | cellular catabolic process | 206 | 9 | 6.46 | 0.19822 | 1 |
| GO:0006955 | immune response | 7 | 1 | 0.22 | 0.19983 | 1 |
| GO:0009095 | aromatic amino acid family biosynthetic ... | 7 | 1 | 0.22 | 0.19983 | 1 |
| GO:0019220 | regulation of phosphate metabolic proces... | 7 | 1 | 0.22 | 0.19983 | 1 |
| GO:0051174 | regulation of phosphorus metabolic proce... | 7 | 1 | 0.22 | 0.19983 | 1 |
| GO:0009733 | response to auxin | 50 | 3 | 1.57 | 0.20599 | 1 |
| GO:0007165 | signal transduction | 378 | 15 | 11.85 | 0.20724 | 1 |
| GO:0009719 | response to endogenous stimulus | 75 | 4 | 2.35 | 0.20822 | 1 |
| GO:0009725 | response to hormone | 75 | 4 | 2.35 | 0.20822 | 1 |
| GO:0006281 | DNA repair | 101 | 5 | 3.16 | 0.20999 | 1 |
| GO:0023052 | signaling | 379 | 15 | 11.88 | 0.21006 | 1 |
| GO:0015939 | pantothenate metabolic process | 8 | 1 | 0.25 | 0.22493 | 1 |
| GO:0015940 | pantothenate biosynthetic process | 8 | 1 | 0.25 | 0.22493 | 1 |
| GO:0000271 | polysaccharide biosynthetic process | 78 | 4 | 2.44 | 0.22816 | 1 |
| GO:0010033 | response to organic substance | 78 | 4 | 2.44 | 0.22816 | 1 |
| GO:0019362 | pyridine nucleotide metabolic process | 78 | 4 | 2.44 | 0.22816 | 1 |
| GO:0033692 | cellular polysaccharide biosynthetic pro... | 78 | 4 | 2.44 | 0.22816 | 1 |
| GO:0046496 | nicotinamide nucleotide metabolic proces... | 78 | 4 | 2.44 | 0.22816 | 1 |
| GO:1901564 | organonitrogen compound metabolic proces... | 2895 | 97 | 90.72 | 0.22998 | 1 |
| GO:0010629 | negative regulation of gene expression | 29 | 2 | 0.91 | 0.23008 | 1 |
| GO:0006733 | oxidoreduction coenzyme metabolic proces... | 79 | 4 | 2.48 | 0.23492 | 1 |
| GO:0044238 | primary metabolic process | 5181 | 169 | 162.35 | 0.2374 | 1 |
| GO:0006974 | cellular response to DNA damage stimulus | 106 | 5 | 3.32 | 0.23864 | 1 |
| GO:0010605 | negative regulation of macromolecule met... | 30 | 2 | 0.94 | 0.24178 | 1 |
| GO:0005976 | polysaccharide metabolic process | 107 | 5 | 3.35 | 0.24449 | 1 |
| GO:0033554 | cellular response to stress | 107 | 5 | 3.35 | 0.24449 | 1 |
| GO:0019752 | carboxylic acid metabolic process | 479 | 18 | 15.01 | 0.24487 | 1 |
| GO:0072524 | pyridine-containing compound metabolic p... | 81 | 4 | 2.54 | 0.24856 | 1 |
| GO:0002376 | immune system process | 9 | 1 | 0.28 | 0.24923 | 1 |
| GO:0043436 | oxoacid metabolic process | 482 | 18 | 15.1 | 0.25312 | 1 |
| GO:0009066 | aspartate family amino acid metabolic pr... | 31 | 2 | 0.97 | 0.2535 | 1 |
| GO:0009892 | negative regulation of metabolic process | 31 | 2 | 0.97 | 0.2535 | 1 |
| GO:0006082 | organic acid metabolic process | 487 | 18 | 15.26 | 0.2671 | 1 |
| GO:0016052 | carbohydrate catabolic process | 84 | 4 | 2.63 | 0.26934 | 1 |
| GO:0003333 | amino acid transmembrane transport | 10 | 1 | 0.31 | 0.27278 | 1 |
| GO:0055114 | oxidation-reduction process | 1558 | 53 | 48.82 | 0.27612 | 1 |
| GO:0048523 | negative regulation of cellular process | 33 | 2 | 1.03 | 0.27696 | 1 |
| GO:0006807 | nitrogen compound metabolic process | 4365 | 142 | 136.78 | 0.29038 | 1 |
| GO:0072330 | monocarboxylic acid biosynthetic process | 61 | 3 | 1.91 | 0.29888 | 1 |
| GO:0043604 | amide biosynthetic process | 597 | 21 | 18.71 | 0.3223 | 1 |
| GO:0006412 | translation | 568 | 20 | 17.8 | 0.32633 | 1 |
| GO:0043043 | peptide biosynthetic process | 574 | 20 | 17.99 | 0.34358 | 1 |
| GO:0007018 | microtubule-based movement | 67 | 3 | 2.1 | 0.35072 | 1 |
| GO:0043603 | cellular amide metabolic process | 608 | 21 | 19.05 | 0.35321 | 1 |
| GO:0051726 | regulation of cell cycle | 14 | 1 | 0.44 | 0.35984 | 1 |
| GO:0006518 | peptide metabolic process | 580 | 20 | 18.18 | 0.36105 | 1 |
| GO:0006633 | fatty acid biosynthetic process | 41 | 2 | 1.28 | 0.36957 | 1 |
| GO:0006631 | fatty acid metabolic process | 70 | 3 | 2.19 | 0.37653 | 1 |
| GO:0019538 | protein metabolic process | 2338 | 76 | 73.26 | 0.37666 | 1 |
| GO:0006732 | coenzyme metabolic process | 129 | 5 | 4.04 | 0.37957 | 1 |
| GO:0006289 | nucleotide-excision repair | 15 | 1 | 0.47 | 0.37993 | 1 |
| GO:0008610 | lipid biosynthetic process | 162 | 6 | 5.08 | 0.39772 | 1 |
| GO:0030001 | metal ion transport | 163 | 6 | 5.11 | 0.40335 | 1 |
| GO:0008037 | cell recognition | 45 | 2 | 1.41 | 0.41421 | 1 |
| GO:0009856 | pollination | 45 | 2 | 1.41 | 0.41421 | 1 |
| GO:0009875 | pollen-pistil interaction | 45 | 2 | 1.41 | 0.41421 | 1 |
| GO:0044706 | multi-multicellular organism process | 45 | 2 | 1.41 | 0.41421 | 1 |
| GO:0048544 | recognition of pollen | 45 | 2 | 1.41 | 0.41421 | 1 |
| GO:1901135 | carbohydrate derivative metabolic proces... | 381 | 13 | 11.94 | 0.41673 | 1 |
| GO:0009073 | aromatic amino acid family biosynthetic ... | 17 | 1 | 0.53 | 0.41824 | 1 |
| GO:0006091 | generation of precursor metabolites and ... | 107 | 4 | 3.35 | 0.43296 | 1 |
| GO:0006928 | movement of cell or subcellular componen... | 77 | 3 | 2.41 | 0.43577 | 1 |
| GO:0006813 | potassium ion transport | 47 | 2 | 1.47 | 0.43594 | 1 |
| GO:0048519 | negative regulation of biological proces... | 48 | 2 | 1.5 | 0.44664 | 1 |
| GO:0006334 | nucleosome assembly | 19 | 1 | 0.6 | 0.45419 | 1 |
| GO:0031497 | chromatin assembly | 19 | 1 | 0.6 | 0.45419 | 1 |
| GO:0050790 | regulation of catalytic activity | 19 | 1 | 0.6 | 0.45419 | 1 |
| GO:0065004 | protein-DNA complex assembly | 19 | 1 | 0.6 | 0.45419 | 1 |
| GO:0065009 | regulation of molecular function | 19 | 1 | 0.6 | 0.45419 | 1 |
| GO:0016567 | protein ubiquitination | 49 | 2 | 1.54 | 0.45722 | 1 |
| GO:0016053 | organic acid biosynthetic process | 142 | 5 | 4.45 | 0.46029 | 1 |
| GO:0046394 | carboxylic acid biosynthetic process | 142 | 5 | 4.45 | 0.46029 | 1 |
| GO:1901566 | organonitrogen compound biosynthetic pro... | 932 | 30 | 29.21 | 0.46686 | 1 |
| GO:0032446 | protein modification by small protein co... | 50 | 2 | 1.57 | 0.46769 | 1 |
| GO:0007017 | microtubule-based process | 81 | 3 | 2.54 | 0.46868 | 1 |
| GO:0006333 | chromatin assembly or disassembly | 20 | 1 | 0.63 | 0.47133 | 1 |
| GO:0006865 | amino acid transport | 20 | 1 | 0.63 | 0.47133 | 1 |
| GO:0034728 | nucleosome organization | 20 | 1 | 0.63 | 0.47133 | 1 |
| GO:0042398 | cellular modified amino acid biosyntheti... | 20 | 1 | 0.63 | 0.47133 | 1 |
| GO:0051235 | maintenance of location | 20 | 1 | 0.63 | 0.47133 | 1 |
| GO:0071824 | protein-DNA complex subunit organization | 20 | 1 | 0.63 | 0.47133 | 1 |
| GO:0022414 | reproductive process | 51 | 2 | 1.6 | 0.47804 | 1 |
| GO:0044262 | cellular carbohydrate metabolic process | 178 | 6 | 5.58 | 0.48681 | 1 |
| GO:0000003 | reproduction | 52 | 2 | 1.63 | 0.48826 | 1 |
| GO:1901360 | organic cyclic compound metabolic proces... | 1928 | 61 | 60.42 | 0.48963 | 1 |
| GO:0034637 | cellular carbohydrate biosynthetic proce... | 117 | 4 | 3.67 | 0.50185 | 1 |
| GO:0006323 | DNA packaging | 22 | 1 | 0.69 | 0.50401 | 1 |
| GO:0010876 | lipid localization | 22 | 1 | 0.69 | 0.50401 | 1 |
| GO:0042221 | response to chemical | 151 | 5 | 4.73 | 0.51442 | 1 |
| GO:0034641 | cellular nitrogen compound metabolic pro... | 2393 | 75 | 74.99 | 0.52199 | 1 |
| GO:0050789 | regulation of biological process | 1342 | 42 | 42.05 | 0.52961 | 1 |
| GO:0006575 | cellular modified amino acid metabolic p... | 24 | 1 | 0.75 | 0.53468 | 1 |
| GO:0050794 | regulation of cellular process | 1313 | 41 | 41.14 | 0.53602 | 1 |
| GO:0000160 | phosphorelay signal transduction system | 91 | 3 | 2.85 | 0.54686 | 1 |
| GO:0046034 | ATP metabolic process | 124 | 4 | 3.89 | 0.54784 | 1 |
| GO:0098656 | anion transmembrane transport | 25 | 1 | 0.78 | 0.5493 | 1 |
| GO:1903825 | organic acid transmembrane transport | 25 | 1 | 0.78 | 0.5493 | 1 |
| GO:1905039 | carboxylic acid transmembrane transport | 25 | 1 | 0.78 | 0.5493 | 1 |
| GO:0090304 | nucleic acid metabolic process | 1547 | 48 | 48.48 | 0.55519 | 1 |
| GO:0033014 | tetrapyrrole biosynthetic process | 26 | 1 | 0.81 | 0.56346 | 1 |
| GO:0065007 | biological regulation | 1391 | 43 | 43.59 | 0.56468 | 1 |
| GO:0009144 | purine nucleoside triphosphate metabolic... | 127 | 4 | 3.98 | 0.56686 | 1 |
| GO:0009199 | ribonucleoside triphosphate metabolic pr... | 127 | 4 | 3.98 | 0.56686 | 1 |
| GO:0009205 | purine ribonucleoside triphosphate metab... | 127 | 4 | 3.98 | 0.56686 | 1 |
| GO:0009123 | nucleoside monophosphate metabolic proce... | 128 | 4 | 4.01 | 0.57311 | 1 |
| GO:0009126 | purine nucleoside monophosphate metaboli... | 128 | 4 | 4.01 | 0.57311 | 1 |
| GO:0009161 | ribonucleoside monophosphate metabolic p... | 128 | 4 | 4.01 | 0.57311 | 1 |
| GO:0009167 | purine ribonucleoside monophosphate meta... | 128 | 4 | 4.01 | 0.57311 | 1 |
| GO:0045454 | cell redox homeostasis | 95 | 3 | 2.98 | 0.5762 | 1 |
| GO:0007186 | G-protein coupled receptor signaling pat... | 27 | 1 | 0.85 | 0.57717 | 1 |
| GO:0009141 | nucleoside triphosphate metabolic proces... | 129 | 4 | 4.04 | 0.5793 | 1 |
| GO:0051186 | cofactor metabolic process | 163 | 5 | 5.11 | 0.58283 | 1 |
| GO:0006511 | ubiquitin-dependent protein catabolic pr... | 62 | 2 | 1.94 | 0.58331 | 1 |
| GO:0019941 | modification-dependent protein catabolic... | 62 | 2 | 1.94 | 0.58331 | 1 |
| GO:0043632 | modification-dependent macromolecule cat... | 62 | 2 | 1.94 | 0.58331 | 1 |
| GO:0046488 | phosphatidylinositol metabolic process | 63 | 2 | 1.97 | 0.59207 | 1 |
| GO:0044281 | small molecule metabolic process | 763 | 23 | 23.91 | 0.60978 | 1 |
| GO:0006497 | protein lipidation | 30 | 1 | 0.94 | 0.6158 | 1 |
| GO:0006505 | GPI anchor metabolic process | 30 | 1 | 0.94 | 0.6158 | 1 |
| GO:0006506 | GPI anchor biosynthetic process | 30 | 1 | 0.94 | 0.6158 | 1 |
| GO:0006661 | phosphatidylinositol biosynthetic proces... | 30 | 1 | 0.94 | 0.6158 | 1 |
| GO:0048583 | regulation of response to stimulus | 30 | 1 | 0.94 | 0.6158 | 1 |
| GO:0006650 | glycerophospholipid metabolic process | 67 | 2 | 2.1 | 0.62573 | 1 |
| GO:0032501 | multicellular organismal process | 67 | 2 | 2.1 | 0.62573 | 1 |
| GO:0007049 | cell cycle | 31 | 1 | 0.97 | 0.62787 | 1 |
| GO:0042158 | lipoprotein biosynthetic process | 31 | 1 | 0.97 | 0.62787 | 1 |
| GO:0006790 | sulfur compound metabolic process | 68 | 2 | 2.13 | 0.6338 | 1 |
| GO:0006418 | tRNA aminoacylation for protein translat... | 104 | 3 | 3.26 | 0.63772 | 1 |
| GO:0019725 | cellular homeostasis | 104 | 3 | 3.26 | 0.63772 | 1 |
| GO:0046486 | glycerolipid metabolic process | 70 | 2 | 2.19 | 0.64954 | 1 |
| GO:1901605 | alpha-amino acid metabolic process | 106 | 3 | 3.32 | 0.65052 | 1 |
| GO:0042592 | homeostatic process | 107 | 3 | 3.35 | 0.65679 | 1 |
| GO:0046474 | glycerophospholipid biosynthetic process | 34 | 1 | 1.07 | 0.66188 | 1 |
| GO:0009150 | purine ribonucleotide metabolic process | 145 | 4 | 4.54 | 0.67103 | 1 |
| GO:0009259 | ribonucleotide metabolic process | 145 | 4 | 4.54 | 0.67103 | 1 |
| GO:0009072 | aromatic amino acid family metabolic pro... | 35 | 1 | 1.1 | 0.67251 | 1 |
| GO:0033013 | tetrapyrrole metabolic process | 35 | 1 | 1.1 | 0.67251 | 1 |
| GO:0042157 | lipoprotein metabolic process | 35 | 1 | 1.1 | 0.67251 | 1 |
| GO:0046483 | heterocycle metabolic process | 1903 | 57 | 59.63 | 0.673 | 1 |
| GO:0045017 | glycerolipid biosynthetic process | 37 | 1 | 1.16 | 0.69279 | 1 |
| GO:0065008 | regulation of biological quality | 150 | 4 | 4.7 | 0.69671 | 1 |
| GO:0043038 | amino acid activation | 114 | 3 | 3.57 | 0.69845 | 1 |
| GO:0043039 | tRNA aminoacylation | 114 | 3 | 3.57 | 0.69845 | 1 |
| GO:0044271 | cellular nitrogen compound biosynthetic ... | 1658 | 49 | 51.96 | 0.69952 | 1 |
| GO:0016051 | carbohydrate biosynthetic process | 152 | 4 | 4.76 | 0.70657 | 1 |
| GO:0006006 | glucose metabolic process | 39 | 1 | 1.22 | 0.71181 | 1 |
| GO:0006352 | DNA-templated transcription, initiation | 39 | 1 | 1.22 | 0.71181 | 1 |
| GO:0006163 | purine nucleotide metabolic process | 154 | 4 | 4.83 | 0.7162 | 1 |
| GO:1901362 | organic cyclic compound biosynthetic pro... | 1109 | 32 | 34.75 | 0.71932 | 1 |
| GO:0006887 | exocytosis | 40 | 1 | 1.25 | 0.72088 | 1 |
| GO:0019693 | ribose phosphate metabolic process | 155 | 4 | 4.86 | 0.72092 | 1 |
| GO:0072521 | purine-containing compound metabolic pro... | 156 | 4 | 4.89 | 0.72559 | 1 |
| GO:0006325 | chromatin organization | 41 | 1 | 1.28 | 0.72966 | 1 |
| GO:0042545 | cell wall modification | 42 | 1 | 1.32 | 0.73817 | 1 |
| GO:0035556 | intracellular signal transduction | 122 | 3 | 3.82 | 0.74125 | 1 |
| GO:0051171 | regulation of nitrogen compound metaboli... | 820 | 23 | 25.7 | 0.74346 | 1 |
| GO:0006139 | nucleobase-containing compound metabolic... | 1785 | 52 | 55.94 | 0.74478 | 1 |
| GO:0080090 | regulation of primary metabolic process | 821 | 23 | 25.73 | 0.74553 | 1 |
| GO:0005991 | trehalose metabolic process | 43 | 1 | 1.35 | 0.74641 | 1 |
| GO:0006664 | glycolipid metabolic process | 43 | 1 | 1.35 | 0.74641 | 1 |
| GO:0009247 | glycolipid biosynthetic process | 43 | 1 | 1.35 | 0.74641 | 1 |
| GO:0046467 | membrane lipid biosynthetic process | 43 | 1 | 1.35 | 0.74641 | 1 |
| GO:0034220 | ion transmembrane transport | 161 | 4 | 5.05 | 0.74807 | 1 |
| GO:0006520 | cellular amino acid metabolic process | 271 | 7 | 8.49 | 0.75096 | 1 |
| GO:0031323 | regulation of cellular metabolic process | 825 | 23 | 25.85 | 0.75371 | 1 |
| GO:0006643 | membrane lipid metabolic process | 45 | 1 | 1.41 | 0.76212 | 1 |
| GO:0008652 | cellular amino acid biosynthetic process | 88 | 2 | 2.76 | 0.76786 | 1 |
| GO:0060255 | regulation of macromolecule metabolic pr... | 836 | 23 | 26.2 | 0.77533 | 1 |
| GO:1903509 | liposaccharide metabolic process | 47 | 1 | 1.47 | 0.77686 | 1 |
| GO:0019222 | regulation of metabolic process | 843 | 23 | 26.42 | 0.78843 | 1 |
| GO:0071103 | DNA conformation change | 49 | 1 | 1.54 | 0.7907 | 1 |
| GO:0044255 | cellular lipid metabolic process | 210 | 5 | 6.58 | 0.79266 | 1 |
| GO:0044257 | cellular protein catabolic process | 93 | 2 | 2.91 | 0.7939 | 1 |
| GO:0051603 | proteolysis involved in cellular protein... | 93 | 2 | 2.91 | 0.7939 | 1 |
| GO:0070647 | protein modification by small protein co... | 93 | 2 | 2.91 | 0.7939 | 1 |
| GO:0010556 | regulation of macromolecule biosynthetic... | 812 | 22 | 25.45 | 0.79435 | 1 |
| GO:0031326 | regulation of cellular biosynthetic proc... | 812 | 22 | 25.45 | 0.79435 | 1 |
| GO:2000112 | regulation of cellular macromolecule bio... | 812 | 22 | 25.45 | 0.79435 | 1 |
| GO:0009889 | regulation of biosynthetic process | 813 | 22 | 25.48 | 0.79618 | 1 |
| GO:0009059 | macromolecule biosynthetic process | 1723 | 49 | 53.99 | 0.79739 | 1 |
| GO:0034645 | cellular macromolecule biosynthetic proc... | 1723 | 49 | 53.99 | 0.79739 | 1 |
| GO:0030163 | protein catabolic process | 95 | 2 | 2.98 | 0.80358 | 1 |
| GO:0005984 | disaccharide metabolic process | 51 | 1 | 1.6 | 0.80367 | 1 |
| GO:0009108 | coenzyme biosynthetic process | 51 | 1 | 1.6 | 0.80367 | 1 |
| GO:0015698 | inorganic anion transport | 51 | 1 | 1.6 | 0.80367 | 1 |
| GO:0098655 | cation transmembrane transport | 136 | 3 | 4.26 | 0.8044 | 1 |
| GO:0098660 | inorganic ion transmembrane transport | 136 | 3 | 4.26 | 0.8044 | 1 |
| GO:0098662 | inorganic cation transmembrane transport | 136 | 3 | 4.26 | 0.8044 | 1 |
| GO:0006644 | phospholipid metabolic process | 96 | 2 | 3.01 | 0.80827 | 1 |
| GO:0010468 | regulation of gene expression | 823 | 22 | 25.79 | 0.81384 | 1 |
| GO:0032940 | secretion by cell | 54 | 1 | 1.69 | 0.82165 | 1 |
| GO:0046903 | secretion | 54 | 1 | 1.69 | 0.82165 | 1 |
| GO:0006725 | cellular aromatic compound metabolic pro... | 1909 | 54 | 59.82 | 0.82225 | 1 |
| GO:0006812 | cation transport | 334 | 8 | 10.47 | 0.82781 | 1 |
| GO:0006855 | drug transmembrane transport | 56 | 1 | 1.75 | 0.83271 | 1 |
| GO:0015893 | drug transport | 56 | 1 | 1.75 | 0.83271 | 1 |
| GO:0042493 | response to drug | 56 | 1 | 1.75 | 0.83271 | 1 |
| GO:0015849 | organic acid transport | 57 | 1 | 1.79 | 0.83799 | 1 |
| GO:0046942 | carboxylic acid transport | 57 | 1 | 1.79 | 0.83799 | 1 |
| GO:0009117 | nucleotide metabolic process | 189 | 4 | 5.92 | 0.84865 | 1 |
| GO:1901576 | organic substance biosynthetic process | 2200 | 62 | 68.94 | 0.84978 | 1 |
| GO:0044265 | cellular macromolecule catabolic process | 106 | 2 | 3.32 | 0.84991 | 1 |
| GO:0006753 | nucleoside phosphate metabolic process | 191 | 4 | 5.99 | 0.85433 | 1 |
| GO:0008654 | phospholipid biosynthetic process | 62 | 1 | 1.94 | 0.86196 | 1 |
| GO:0015711 | organic anion transport | 62 | 1 | 1.94 | 0.86196 | 1 |
| GO:0009311 | oligosaccharide metabolic process | 63 | 1 | 1.97 | 0.86632 | 1 |
| GO:0055085 | transmembrane transport | 1137 | 30 | 35.63 | 0.86784 | 1 |
| GO:0071555 | cell wall organization | 64 | 1 | 2.01 | 0.87053 | 1 |
| GO:0006355 | regulation of transcription, DNA-templat... | 792 | 20 | 24.82 | 0.87318 | 1 |
| GO:0051252 | regulation of RNA metabolic process | 792 | 20 | 24.82 | 0.87318 | 1 |
| GO:1903506 | regulation of nucleic acid-templated tra... | 792 | 20 | 24.82 | 0.87318 | 1 |
| GO:2001141 | regulation of RNA biosynthetic process | 792 | 20 | 24.82 | 0.87318 | 1 |
| GO:0006399 | tRNA metabolic process | 157 | 3 | 4.92 | 0.87456 | 1 |
| GO:0019318 | hexose metabolic process | 65 | 1 | 2.04 | 0.87462 | 1 |
| GO:0045229 | external encapsulating structure organiz... | 65 | 1 | 2.04 | 0.87462 | 1 |
| GO:0018130 | heterocycle biosynthetic process | 1078 | 28 | 33.78 | 0.87975 | 1 |
| GO:0019219 | regulation of nucleobase-containing comp... | 798 | 20 | 25.01 | 0.88102 | 1 |
| GO:0034622 | cellular macromolecular complex assembly | 67 | 1 | 2.1 | 0.8824 | 1 |
| GO:0006351 | transcription, DNA-templated | 908 | 23 | 28.45 | 0.88563 | 1 |
| GO:0097659 | nucleic acid-templated transcription | 908 | 23 | 28.45 | 0.88563 | 1 |
| GO:0032774 | RNA biosynthetic process | 911 | 23 | 28.55 | 0.88911 | 1 |
| GO:0005996 | monosaccharide metabolic process | 69 | 1 | 2.16 | 0.88971 | 1 |
| GO:0006486 | protein glycosylation | 70 | 1 | 2.19 | 0.89319 | 1 |
| GO:0009100 | glycoprotein metabolic process | 70 | 1 | 2.19 | 0.89319 | 1 |
| GO:0009101 | glycoprotein biosynthetic process | 70 | 1 | 2.19 | 0.89319 | 1 |
| GO:0043413 | macromolecule glycosylation | 70 | 1 | 2.19 | 0.89319 | 1 |
| GO:0015672 | monovalent inorganic cation transport | 165 | 3 | 5.17 | 0.8948 | 1 |
| GO:1901607 | alpha-amino acid biosynthetic process | 71 | 1 | 2.22 | 0.89656 | 1 |
| GO:0006820 | anion transport | 122 | 2 | 3.82 | 0.89973 | 1 |
| GO:0044249 | cellular biosynthetic process | 2158 | 59 | 67.62 | 0.90074 | 1 |
| GO:0070085 | glycosylation | 75 | 1 | 2.35 | 0.90902 | 1 |
| GO:0006811 | ion transport | 495 | 11 | 15.51 | 0.91356 | 1 |
| GO:0010467 | gene expression | 1668 | 44 | 52.27 | 0.91438 | 1 |
| GO:0019637 | organophosphate metabolic process | 303 | 6 | 9.49 | 0.91798 | 1 |
| GO:0055086 | nucleobase-containing small molecule met... | 222 | 4 | 6.96 | 0.92164 | 1 |
| GO:0051188 | cofactor biosynthetic process | 80 | 1 | 2.51 | 0.92251 | 1 |
| GO:0065003 | macromolecular complex assembly | 80 | 1 | 2.51 | 0.92251 | 1 |
| GO:0051276 | chromosome organization | 83 | 1 | 2.6 | 0.92962 | 1 |
| GO:0034660 | ncRNA metabolic process | 187 | 3 | 5.86 | 0.93622 | 1 |
| GO:0009058 | biosynthetic process | 2364 | 63 | 74.08 | 0.94384 | 1 |
| GO:0034654 | nucleobase-containing compound biosynthe... | 994 | 23 | 31.15 | 0.95646 | 1 |
| GO:0019438 | aromatic compound biosynthetic process | 1068 | 25 | 33.47 | 0.95696 | 1 |
| GO:0043933 | macromolecular complex subunit organizat... | 99 | 1 | 3.1 | 0.95792 | 1 |
| GO:0006818 | hydrogen transport | 100 | 1 | 3.13 | 0.95925 | 1 |
| GO:0015992 | proton transport | 100 | 1 | 3.13 | 0.95925 | 1 |
| GO:1902600 | hydrogen ion transmembrane transport | 100 | 1 | 3.13 | 0.95925 | 1 |
| GO:0016192 | vesicle-mediated transport | 160 | 2 | 5.01 | 0.96318 | 1 |
| GO:0071705 | nitrogen compound transport | 273 | 4 | 8.55 | 0.97424 | 1 |
| GO:1901137 | carbohydrate derivative biosynthetic pro... | 177 | 2 | 5.55 | 0.97685 | 1 |
| GO:0006886 | intracellular protein transport | 125 | 1 | 3.92 | 0.98179 | 1 |
| GO:0016070 | RNA metabolic process | 1225 | 27 | 38.39 | 0.98485 | 1 |
| GO:0022607 | cellular component assembly | 131 | 1 | 4.11 | 0.98499 | 1 |
| GO:0051179 | localization | 1741 | 41 | 54.56 | 0.98595 | 1 |
| GO:0006810 | transport | 1711 | 40 | 53.62 | 0.98688 | 1 |
| GO:0051234 | establishment of localization | 1711 | 40 | 53.62 | 0.98688 | 1 |
| GO:0034613 | cellular protein localization | 141 | 1 | 4.42 | 0.98913 | 1 |
| GO:0070727 | cellular macromolecule localization | 141 | 1 | 4.42 | 0.98913 | 1 |
| GO:0006996 | organelle organization | 144 | 1 | 4.51 | 0.99014 | 1 |
| GO:0090407 | organophosphate biosynthetic process | 151 | 1 | 4.73 | 0.99214 | 1 |
| GO:0006396 | RNA processing | 159 | 1 | 4.98 | 0.99393 | 1 |
| GO:0046907 | intracellular transport | 159 | 1 | 4.98 | 0.99393 | 1 |
| GO:0051649 | establishment of localization in cell | 159 | 1 | 4.98 | 0.99393 | 1 |
| GO:0033036 | macromolecule localization | 230 | 2 | 7.21 | 0.99479 | 1 |
| GO:0071702 | organic substance transport | 343 | 4 | 10.75 | 0.99514 | 1 |
| GO:0044085 | cellular component biogenesis | 177 | 1 | 5.55 | 0.99661 | 1 |
| GO:0051641 | cellular localization | 188 | 1 | 5.89 | 0.99763 | 1 |
| GO:0015031 | protein transport | 189 | 1 | 5.92 | 0.9977 | 1 |
| GO:0045184 | establishment of protein localization | 189 | 1 | 5.92 | 0.9977 | 1 |
| GO:0015833 | peptide transport | 197 | 1 | 6.17 | 0.99823 | 1 |
| GO:0042886 | amide transport | 197 | 1 | 6.17 | 0.99823 | 1 |
| GO:0008104 | protein localization | 205 | 1 | 6.42 | 0.99864 | 1 |
| GO:0016043 | cellular component organization | 318 | 2 | 9.96 | 0.99961 | 1 |
| GO:0006508 | proteolysis | 445 | 4 | 13.94 | 0.99965 | 1 |
| GO:0071840 | cellular component organization or bioge... | 364 | 2 | 11.41 | 0.9999 | 1 |
| GO:0008150 | biological_process | 9765 | 306 | 306 | 1 | 1 |
| GO:0000041 | transition metal ion transport | 13 | 0 | 0.41 | 1 | 1 |
| GO:0000070 | mitotic sister chromatid segregation | 7 | 0 | 0.22 | 1 | 1 |
| GO:0000097 | sulfur amino acid biosynthetic process | 12 | 0 | 0.38 | 1 | 1 |
| GO:0000105 | histidine biosynthetic process | 14 | 0 | 0.44 | 1 | 1 |
| GO:0000226 | microtubule cytoskeleton organization | 7 | 0 | 0.22 | 1 | 1 |
| GO:0000270 | peptidoglycan metabolic process | 15 | 0 | 0.47 | 1 | 1 |
| GO:0000272 | polysaccharide catabolic process | 11 | 0 | 0.34 | 1 | 1 |
| GO:0000278 | mitotic cell cycle | 10 | 0 | 0.31 | 1 | 1 |
| GO:0000280 | nuclear division | 9 | 0 | 0.28 | 1 | 1 |
| GO:0000375 | RNA splicing, via transesterification re... | 15 | 0 | 0.47 | 1 | 1 |
| GO:0000377 | RNA splicing, via transesterification re... | 15 | 0 | 0.47 | 1 | 1 |
| GO:0000398 | mRNA splicing, via spliceosome | 15 | 0 | 0.47 | 1 | 1 |
| GO:0000413 | protein peptidyl-prolyl isomerization | 46 | 0 | 1.44 | 1 | 1 |
| GO:0000726 | non-recombinational repair | 6 | 0 | 0.19 | 1 | 1 |
| GO:0000819 | sister chromatid segregation | 7 | 0 | 0.22 | 1 | 1 |
| GO:0000956 | nuclear-transcribed mRNA catabolic proce... | 6 | 0 | 0.19 | 1 | 1 |
| GO:0001510 | RNA methylation | 7 | 0 | 0.22 | 1 | 1 |
| GO:0001522 | pseudouridine synthesis | 26 | 0 | 0.81 | 1 | 1 |
| GO:0001539 | cilium or flagellum-dependent cell motil... | 9 | 0 | 0.28 | 1 | 1 |
| GO:0002790 | peptide secretion | 14 | 0 | 0.44 | 1 | 1 |
| GO:0005985 | sucrose metabolic process | 8 | 0 | 0.25 | 1 | 1 |
| GO:0005992 | trehalose biosynthetic process | 34 | 0 | 1.07 | 1 | 1 |
| GO:0006013 | mannose metabolic process | 19 | 0 | 0.6 | 1 | 1 |
| GO:0006020 | inositol metabolic process | 11 | 0 | 0.34 | 1 | 1 |
| GO:0006027 | glycosaminoglycan catabolic process | 10 | 0 | 0.31 | 1 | 1 |
| GO:0006066 | alcohol metabolic process | 23 | 0 | 0.72 | 1 | 1 |
| GO:0006071 | glycerol metabolic process | 6 | 0 | 0.19 | 1 | 1 |
| GO:0006072 | glycerol-3-phosphate metabolic process | 5 | 0 | 0.16 | 1 | 1 |
| GO:0006074 | (1->3)-beta-D-glucan metabolic process | 20 | 0 | 0.63 | 1 | 1 |
| GO:0006075 | (1->3)-beta-D-glucan biosynthetic proces... | 20 | 0 | 0.63 | 1 | 1 |
| GO:0006081 | cellular aldehyde metabolic process | 26 | 0 | 0.81 | 1 | 1 |
| GO:0006094 | gluconeogenesis | 17 | 0 | 0.53 | 1 | 1 |
| GO:0006097 | glyoxylate cycle | 9 | 0 | 0.28 | 1 | 1 |
| GO:0006098 | pentose-phosphate shunt | 10 | 0 | 0.31 | 1 | 1 |
| GO:0006099 | tricarboxylic acid cycle | 9 | 0 | 0.28 | 1 | 1 |
| GO:0006101 | citrate metabolic process | 9 | 0 | 0.28 | 1 | 1 |
| GO:0006119 | oxidative phosphorylation | 9 | 0 | 0.28 | 1 | 1 |
| GO:0006122 | mitochondrial electron transport, ubiqui... | 7 | 0 | 0.22 | 1 | 1 |
| GO:0006164 | purine nucleotide biosynthetic process | 56 | 0 | 1.75 | 1 | 1 |
| GO:0006220 | pyrimidine nucleotide metabolic process | 7 | 0 | 0.22 | 1 | 1 |
| GO:0006221 | pyrimidine nucleotide biosynthetic proce... | 7 | 0 | 0.22 | 1 | 1 |
| GO:0006260 | DNA replication | 68 | 0 | 2.13 | 1 | 1 |
| GO:0006261 | DNA-dependent DNA replication | 16 | 0 | 0.5 | 1 | 1 |
| GO:0006265 | DNA topological change | 26 | 0 | 0.81 | 1 | 1 |
| GO:0006270 | DNA replication initiation | 11 | 0 | 0.34 | 1 | 1 |
| GO:0006275 | regulation of DNA replication | 5 | 0 | 0.16 | 1 | 1 |
| GO:0006284 | base-excision repair | 16 | 0 | 0.5 | 1 | 1 |
| GO:0006298 | mismatch repair | 16 | 0 | 0.5 | 1 | 1 |
| GO:0006302 | double-strand break repair | 8 | 0 | 0.25 | 1 | 1 |
| GO:0006303 | double-strand break repair via nonhomolo... | 6 | 0 | 0.19 | 1 | 1 |
| GO:0006310 | DNA recombination | 37 | 0 | 1.16 | 1 | 1 |
| GO:0006313 | transposition, DNA-mediated | 11 | 0 | 0.34 | 1 | 1 |
| GO:0006354 | DNA-templated transcription, elongation | 9 | 0 | 0.28 | 1 | 1 |
| GO:0006357 | regulation of transcription from RNA pol... | 15 | 0 | 0.47 | 1 | 1 |
| GO:0006364 | rRNA processing | 30 | 0 | 0.94 | 1 | 1 |
| GO:0006366 | transcription from RNA polymerase II pro... | 28 | 0 | 0.88 | 1 | 1 |
| GO:0006367 | transcription initiation from RNA polyme... | 12 | 0 | 0.38 | 1 | 1 |
| GO:0006368 | transcription elongation from RNA polyme... | 8 | 0 | 0.25 | 1 | 1 |
| GO:0006378 | mRNA polyadenylation | 6 | 0 | 0.19 | 1 | 1 |
| GO:0006383 | transcription from RNA polymerase III pr... | 11 | 0 | 0.34 | 1 | 1 |
| GO:0006397 | mRNA processing | 37 | 0 | 1.16 | 1 | 1 |
| GO:0006400 | tRNA modification | 11 | 0 | 0.34 | 1 | 1 |
| GO:0006401 | RNA catabolic process | 9 | 0 | 0.28 | 1 | 1 |
| GO:0006402 | mRNA catabolic process | 7 | 0 | 0.22 | 1 | 1 |
| GO:0006413 | translational initiation | 34 | 0 | 1.07 | 1 | 1 |
| GO:0006414 | translational elongation | 17 | 0 | 0.53 | 1 | 1 |
| GO:0006415 | translational termination | 16 | 0 | 0.5 | 1 | 1 |
| GO:0006419 | alanyl-tRNA aminoacylation | 7 | 0 | 0.22 | 1 | 1 |
| GO:0006420 | arginyl-tRNA aminoacylation | 5 | 0 | 0.16 | 1 | 1 |
| GO:0006457 | protein folding | 48 | 0 | 1.5 | 1 | 1 |
| GO:0006461 | protein complex assembly | 55 | 0 | 1.72 | 1 | 1 |
| GO:0006470 | protein dephosphorylation | 8 | 0 | 0.25 | 1 | 1 |
| GO:0006473 | protein acetylation | 6 | 0 | 0.19 | 1 | 1 |
| GO:0006475 | internal protein amino acid acetylation | 5 | 0 | 0.16 | 1 | 1 |
| GO:0006479 | protein methylation | 13 | 0 | 0.41 | 1 | 1 |
| GO:0006487 | protein N-linked glycosylation | 18 | 0 | 0.56 | 1 | 1 |
| GO:0006525 | arginine metabolic process | 7 | 0 | 0.22 | 1 | 1 |
| GO:0006528 | asparagine metabolic process | 12 | 0 | 0.38 | 1 | 1 |
| GO:0006529 | asparagine biosynthetic process | 12 | 0 | 0.38 | 1 | 1 |
| GO:0006534 | cysteine metabolic process | 6 | 0 | 0.19 | 1 | 1 |
| GO:0006535 | cysteine biosynthetic process from serin... | 6 | 0 | 0.19 | 1 | 1 |
| GO:0006536 | glutamate metabolic process | 6 | 0 | 0.19 | 1 | 1 |
| GO:0006537 | glutamate biosynthetic process | 5 | 0 | 0.16 | 1 | 1 |
| GO:0006541 | glutamine metabolic process | 11 | 0 | 0.34 | 1 | 1 |
| GO:0006542 | glutamine biosynthetic process | 8 | 0 | 0.25 | 1 | 1 |
| GO:0006544 | glycine metabolic process | 5 | 0 | 0.16 | 1 | 1 |
| GO:0006546 | glycine catabolic process | 5 | 0 | 0.16 | 1 | 1 |
| GO:0006547 | histidine metabolic process | 14 | 0 | 0.44 | 1 | 1 |
| GO:0006553 | lysine metabolic process | 7 | 0 | 0.22 | 1 | 1 |
| GO:0006556 | S-adenosylmethionine biosynthetic proces... | 15 | 0 | 0.47 | 1 | 1 |
| GO:0006558 | L-phenylalanine metabolic process | 6 | 0 | 0.19 | 1 | 1 |
| GO:0006563 | L-serine metabolic process | 6 | 0 | 0.19 | 1 | 1 |
| GO:0006568 | tryptophan metabolic process | 16 | 0 | 0.5 | 1 | 1 |
| GO:0006569 | tryptophan catabolic process | 6 | 0 | 0.19 | 1 | 1 |
| GO:0006576 | cellular biogenic amine metabolic proces... | 26 | 0 | 0.81 | 1 | 1 |
| GO:0006586 | indolalkylamine metabolic process | 16 | 0 | 0.5 | 1 | 1 |
| GO:0006595 | polyamine metabolic process | 8 | 0 | 0.25 | 1 | 1 |
| GO:0006596 | polyamine biosynthetic process | 8 | 0 | 0.25 | 1 | 1 |
| GO:0006597 | spermine biosynthetic process | 7 | 0 | 0.22 | 1 | 1 |
| GO:0006605 | protein targeting | 24 | 0 | 0.75 | 1 | 1 |
| GO:0006612 | protein targeting to membrane | 18 | 0 | 0.56 | 1 | 1 |
| GO:0006613 | cotranslational protein targeting to mem... | 17 | 0 | 0.53 | 1 | 1 |
| GO:0006614 | SRP-dependent cotranslational protein ta... | 17 | 0 | 0.53 | 1 | 1 |
| GO:0006621 | protein retention in ER lumen | 14 | 0 | 0.44 | 1 | 1 |
| GO:0006626 | protein targeting to mitochondrion | 5 | 0 | 0.16 | 1 | 1 |
| GO:0006635 | fatty acid beta-oxidation | 11 | 0 | 0.34 | 1 | 1 |
| GO:0006720 | isoprenoid metabolic process | 29 | 0 | 0.91 | 1 | 1 |
| GO:0006721 | terpenoid metabolic process | 11 | 0 | 0.34 | 1 | 1 |
| GO:0006739 | NADP metabolic process | 16 | 0 | 0.5 | 1 | 1 |
| GO:0006741 | NADP biosynthetic process | 6 | 0 | 0.19 | 1 | 1 |
| GO:0006749 | glutathione metabolic process | 8 | 0 | 0.25 | 1 | 1 |
| GO:0006750 | glutathione biosynthetic process | 5 | 0 | 0.16 | 1 | 1 |
| GO:0006754 | ATP biosynthetic process | 37 | 0 | 1.16 | 1 | 1 |
| GO:0006760 | folic acid-containing compound metabolic... | 5 | 0 | 0.16 | 1 | 1 |
| GO:0006772 | thiamine metabolic process | 8 | 0 | 0.25 | 1 | 1 |
| GO:0006777 | Mo-molybdopterin cofactor biosynthetic p... | 5 | 0 | 0.16 | 1 | 1 |
| GO:0006778 | porphyrin-containing compound metabolic ... | 20 | 0 | 0.63 | 1 | 1 |
| GO:0006779 | porphyrin-containing compound biosynthet... | 12 | 0 | 0.38 | 1 | 1 |
| GO:0006783 | heme biosynthetic process | 5 | 0 | 0.16 | 1 | 1 |
| GO:0006787 | porphyrin-containing compound catabolic ... | 7 | 0 | 0.22 | 1 | 1 |
| GO:0006801 | superoxide metabolic process | 12 | 0 | 0.38 | 1 | 1 |
| GO:0006814 | sodium ion transport | 23 | 0 | 0.72 | 1 | 1 |
| GO:0006817 | phosphate ion transport | 7 | 0 | 0.22 | 1 | 1 |
| GO:0006821 | chloride transport | 13 | 0 | 0.41 | 1 | 1 |
| GO:0006825 | copper ion transport | 7 | 0 | 0.22 | 1 | 1 |
| GO:0006826 | iron ion transport | 5 | 0 | 0.16 | 1 | 1 |
| GO:0006835 | dicarboxylic acid transport | 16 | 0 | 0.5 | 1 | 1 |
| GO:0006839 | mitochondrial transport | 6 | 0 | 0.19 | 1 | 1 |
| GO:0006869 | lipid transport | 18 | 0 | 0.56 | 1 | 1 |
| GO:0006873 | cellular ion homeostasis | 9 | 0 | 0.28 | 1 | 1 |
| GO:0006875 | cellular metal ion homeostasis | 9 | 0 | 0.28 | 1 | 1 |
| GO:0006879 | cellular iron ion homeostasis | 9 | 0 | 0.28 | 1 | 1 |
| GO:0006888 | ER to Golgi vesicle-mediated transport | 15 | 0 | 0.47 | 1 | 1 |
| GO:0006897 | endocytosis | 5 | 0 | 0.16 | 1 | 1 |
| GO:0006904 | vesicle docking involved in exocytosis | 12 | 0 | 0.38 | 1 | 1 |
| GO:0006913 | nucleocytoplasmic transport | 7 | 0 | 0.22 | 1 | 1 |
| GO:0006915 | apoptotic process | 6 | 0 | 0.19 | 1 | 1 |
| GO:0006935 | chemotaxis | 7 | 0 | 0.22 | 1 | 1 |
| GO:0007005 | mitochondrion organization | 7 | 0 | 0.22 | 1 | 1 |
| GO:0007010 | cytoskeleton organization | 37 | 0 | 1.16 | 1 | 1 |
| GO:0007015 | actin filament organization | 15 | 0 | 0.47 | 1 | 1 |
| GO:0007031 | peroxisome organization | 8 | 0 | 0.25 | 1 | 1 |
| GO:0007034 | vacuolar transport | 15 | 0 | 0.47 | 1 | 1 |
| GO:0007059 | chromosome segregation | 13 | 0 | 0.41 | 1 | 1 |
| GO:0007155 | cell adhesion | 47 | 0 | 1.47 | 1 | 1 |
| GO:0007156 | homophilic cell adhesion via plasma memb... | 16 | 0 | 0.5 | 1 | 1 |
| GO:0007166 | cell surface receptor signaling pathway | 9 | 0 | 0.28 | 1 | 1 |
| GO:0007264 | small GTPase mediated signal transductio... | 20 | 0 | 0.63 | 1 | 1 |
| GO:0007265 | Ras protein signal transduction | 10 | 0 | 0.31 | 1 | 1 |
| GO:0007266 | Rho protein signal transduction | 5 | 0 | 0.16 | 1 | 1 |
| GO:0007267 | cell-cell signaling | 6 | 0 | 0.19 | 1 | 1 |
| GO:0007275 | multicellular organism development | 21 | 0 | 0.66 | 1 | 1 |
| GO:0008033 | tRNA processing | 43 | 0 | 1.35 | 1 | 1 |
| GO:0008064 | regulation of actin polymerization or de... | 13 | 0 | 0.41 | 1 | 1 |
| GO:0008154 | actin polymerization or depolymerization | 13 | 0 | 0.41 | 1 | 1 |
| GO:0008213 | protein alkylation | 13 | 0 | 0.41 | 1 | 1 |
| GO:0008215 | spermine metabolic process | 7 | 0 | 0.22 | 1 | 1 |
| GO:0008216 | spermidine metabolic process | 7 | 0 | 0.22 | 1 | 1 |
| GO:0008219 | cell death | 6 | 0 | 0.19 | 1 | 1 |
| GO:0008272 | sulfate transport | 24 | 0 | 0.75 | 1 | 1 |
| GO:0008295 | spermidine biosynthetic process | 7 | 0 | 0.22 | 1 | 1 |
| GO:0008299 | isoprenoid biosynthetic process | 29 | 0 | 0.91 | 1 | 1 |
| GO:0008380 | RNA splicing | 21 | 0 | 0.66 | 1 | 1 |
| GO:0008643 | carbohydrate transport | 60 | 0 | 1.88 | 1 | 1 |
| GO:0009060 | aerobic respiration | 16 | 0 | 0.5 | 1 | 1 |
| GO:0009062 | fatty acid catabolic process | 11 | 0 | 0.34 | 1 | 1 |
| GO:0009063 | cellular amino acid catabolic process | 16 | 0 | 0.5 | 1 | 1 |
| GO:0009064 | glutamine family amino acid metabolic pr... | 24 | 0 | 0.75 | 1 | 1 |
| GO:0009067 | aspartate family amino acid biosynthetic... | 25 | 0 | 0.78 | 1 | 1 |
| GO:0009069 | serine family amino acid metabolic proce... | 11 | 0 | 0.34 | 1 | 1 |
| GO:0009070 | serine family amino acid biosynthetic pr... | 6 | 0 | 0.19 | 1 | 1 |
| GO:0009071 | serine family amino acid catabolic proce... | 5 | 0 | 0.16 | 1 | 1 |
| GO:0009074 | aromatic amino acid family catabolic pro... | 7 | 0 | 0.22 | 1 | 1 |
| GO:0009084 | glutamine family amino acid biosynthetic... | 17 | 0 | 0.53 | 1 | 1 |
| GO:0009085 | lysine biosynthetic process | 7 | 0 | 0.22 | 1 | 1 |
| GO:0009086 | methionine biosynthetic process | 6 | 0 | 0.19 | 1 | 1 |
| GO:0009089 | lysine biosynthetic process via diaminop... | 7 | 0 | 0.22 | 1 | 1 |
| GO:0009094 | L-phenylalanine biosynthetic process | 5 | 0 | 0.16 | 1 | 1 |
| GO:0009116 | nucleoside metabolic process | 38 | 0 | 1.19 | 1 | 1 |
| GO:0009119 | ribonucleoside metabolic process | 17 | 0 | 0.53 | 1 | 1 |
| GO:0009124 | nucleoside monophosphate biosynthetic pr... | 41 | 0 | 1.28 | 1 | 1 |
| GO:0009127 | purine nucleoside monophosphate biosynth... | 41 | 0 | 1.28 | 1 | 1 |
| GO:0009142 | nucleoside triphosphate biosynthetic pro... | 37 | 0 | 1.16 | 1 | 1 |
| GO:0009145 | purine nucleoside triphosphate biosynthe... | 37 | 0 | 1.16 | 1 | 1 |
| GO:0009152 | purine ribonucleotide biosynthetic proce... | 47 | 0 | 1.47 | 1 | 1 |
| GO:0009156 | ribonucleoside monophosphate biosyntheti... | 41 | 0 | 1.28 | 1 | 1 |
| GO:0009163 | nucleoside biosynthetic process | 6 | 0 | 0.19 | 1 | 1 |
| GO:0009165 | nucleotide biosynthetic process | 76 | 0 | 2.38 | 1 | 1 |
| GO:0009168 | purine ribonucleoside monophosphate bios... | 41 | 0 | 1.28 | 1 | 1 |
| GO:0009187 | cyclic nucleotide metabolic process | 5 | 0 | 0.16 | 1 | 1 |
| GO:0009201 | ribonucleoside triphosphate biosynthetic... | 37 | 0 | 1.16 | 1 | 1 |
| GO:0009206 | purine ribonucleoside triphosphate biosy... | 37 | 0 | 1.16 | 1 | 1 |
| GO:0009225 | nucleotide-sugar metabolic process | 5 | 0 | 0.16 | 1 | 1 |
| GO:0009228 | thiamine biosynthetic process | 8 | 0 | 0.25 | 1 | 1 |
| GO:0009240 | isopentenyl diphosphate biosynthetic pro... | 5 | 0 | 0.16 | 1 | 1 |
| GO:0009245 | lipid A biosynthetic process | 9 | 0 | 0.28 | 1 | 1 |
| GO:0009253 | peptidoglycan catabolic process | 10 | 0 | 0.31 | 1 | 1 |
| GO:0009254 | peptidoglycan turnover | 5 | 0 | 0.16 | 1 | 1 |
| GO:0009260 | ribonucleotide biosynthetic process | 47 | 0 | 1.47 | 1 | 1 |
| GO:0009297 | pilus assembly | 20 | 0 | 0.63 | 1 | 1 |
| GO:0009306 | protein secretion | 14 | 0 | 0.44 | 1 | 1 |
| GO:0009308 | amine metabolic process | 54 | 0 | 1.69 | 1 | 1 |
| GO:0009309 | amine biosynthetic process | 10 | 0 | 0.31 | 1 | 1 |
| GO:0009310 | amine catabolic process | 6 | 0 | 0.19 | 1 | 1 |
| GO:0009312 | oligosaccharide biosynthetic process | 48 | 0 | 1.5 | 1 | 1 |
| GO:0009401 | phosphoenolpyruvate-dependent sugar phos... | 38 | 0 | 1.19 | 1 | 1 |
| GO:0009405 | pathogenesis | 5 | 0 | 0.16 | 1 | 1 |
| GO:0009435 | NAD biosynthetic process | 5 | 0 | 0.16 | 1 | 1 |
| GO:0009451 | RNA modification | 43 | 0 | 1.35 | 1 | 1 |
| GO:0009628 | response to abiotic stimulus | 8 | 0 | 0.25 | 1 | 1 |
| GO:0009664 | plant-type cell wall organization | 11 | 0 | 0.34 | 1 | 1 |
| GO:0009690 | cytokinin metabolic process | 9 | 0 | 0.28 | 1 | 1 |
| GO:0009832 | plant-type cell wall biogenesis | 9 | 0 | 0.28 | 1 | 1 |
| GO:0009891 | positive regulation of biosynthetic proc... | 10 | 0 | 0.31 | 1 | 1 |
| GO:0009893 | positive regulation of metabolic process | 10 | 0 | 0.31 | 1 | 1 |
| GO:0009966 | regulation of signal transduction | 24 | 0 | 0.75 | 1 | 1 |
| GO:0010035 | response to inorganic substance | 8 | 0 | 0.25 | 1 | 1 |
| GO:0010109 | regulation of photosynthesis | 5 | 0 | 0.16 | 1 | 1 |
| GO:0010207 | photosystem II assembly | 9 | 0 | 0.28 | 1 | 1 |
| GO:0010215 | cellulose microfibril organization | 9 | 0 | 0.28 | 1 | 1 |
| GO:0010256 | endomembrane system organization | 7 | 0 | 0.22 | 1 | 1 |
| GO:0010498 | proteasomal protein catabolic process | 7 | 0 | 0.22 | 1 | 1 |
| GO:0010557 | positive regulation of macromolecule bio... | 10 | 0 | 0.31 | 1 | 1 |
| GO:0010604 | positive regulation of macromolecule met... | 10 | 0 | 0.31 | 1 | 1 |
| GO:0010628 | positive regulation of gene expression | 10 | 0 | 0.31 | 1 | 1 |
| GO:0010638 | positive regulation of organelle organiz... | 5 | 0 | 0.16 | 1 | 1 |
| GO:0010639 | negative regulation of organelle organiz... | 9 | 0 | 0.28 | 1 | 1 |
| GO:0010646 | regulation of cell communication | 24 | 0 | 0.75 | 1 | 1 |
| GO:0010817 | regulation of hormone levels | 9 | 0 | 0.28 | 1 | 1 |
| GO:0012501 | programmed cell death | 6 | 0 | 0.19 | 1 | 1 |
| GO:0015693 | magnesium ion transport | 12 | 0 | 0.38 | 1 | 1 |
| GO:0015696 | ammonium transport | 15 | 0 | 0.47 | 1 | 1 |
| GO:0015718 | monocarboxylic acid transport | 23 | 0 | 0.72 | 1 | 1 |
| GO:0015725 | gluconate transport | 13 | 0 | 0.41 | 1 | 1 |
| GO:0015727 | lactate transport | 6 | 0 | 0.19 | 1 | 1 |
| GO:0015740 | C4-dicarboxylate transport | 13 | 0 | 0.41 | 1 | 1 |
| GO:0015743 | malate transport | 11 | 0 | 0.34 | 1 | 1 |
| GO:0015780 | nucleotide-sugar transport | 7 | 0 | 0.22 | 1 | 1 |
| GO:0015781 | pyrimidine nucleotide-sugar transport | 7 | 0 | 0.22 | 1 | 1 |
| GO:0015850 | organic hydroxy compound transport | 7 | 0 | 0.22 | 1 | 1 |
| GO:0015858 | nucleoside transport | 11 | 0 | 0.34 | 1 | 1 |
| GO:0015914 | phospholipid transport | 5 | 0 | 0.16 | 1 | 1 |
| GO:0015936 | coenzyme A metabolic process | 7 | 0 | 0.22 | 1 | 1 |
| GO:0015969 | guanosine tetraphosphate metabolic proce... | 5 | 0 | 0.16 | 1 | 1 |
| GO:0015977 | carbon fixation | 8 | 0 | 0.25 | 1 | 1 |
| GO:0015980 | energy derivation by oxidation of organi... | 31 | 0 | 0.97 | 1 | 1 |
| GO:0015985 | energy coupled proton transport, down el... | 35 | 0 | 1.1 | 1 | 1 |
| GO:0015986 | ATP synthesis coupled proton transport | 35 | 0 | 1.1 | 1 | 1 |
| GO:0015988 | energy coupled proton transmembrane tran... | 28 | 0 | 0.88 | 1 | 1 |
| GO:0015991 | ATP hydrolysis coupled proton transport | 28 | 0 | 0.88 | 1 | 1 |
| GO:0016042 | lipid catabolic process | 18 | 0 | 0.56 | 1 | 1 |
| GO:0016049 | cell growth | 9 | 0 | 0.28 | 1 | 1 |
| GO:0016054 | organic acid catabolic process | 33 | 0 | 1.03 | 1 | 1 |
| GO:0016055 | Wnt signaling pathway | 6 | 0 | 0.19 | 1 | 1 |
| GO:0016071 | mRNA metabolic process | 47 | 0 | 1.47 | 1 | 1 |
| GO:0016072 | rRNA metabolic process | 30 | 0 | 0.94 | 1 | 1 |
| GO:0016114 | terpenoid biosynthetic process | 11 | 0 | 0.34 | 1 | 1 |
| GO:0016197 | endosomal transport | 9 | 0 | 0.28 | 1 | 1 |
| GO:0016226 | iron-sulfur cluster assembly | 13 | 0 | 0.41 | 1 | 1 |
| GO:0016255 | attachment of GPI anchor to protein | 6 | 0 | 0.19 | 1 | 1 |
| GO:0016458 | gene silencing | 7 | 0 | 0.22 | 1 | 1 |
| GO:0016482 | cytosolic transport | 7 | 0 | 0.22 | 1 | 1 |
| GO:0016485 | protein processing | 11 | 0 | 0.34 | 1 | 1 |
| GO:0016559 | peroxisome fission | 6 | 0 | 0.19 | 1 | 1 |
| GO:0016569 | covalent chromatin modification | 17 | 0 | 0.53 | 1 | 1 |
| GO:0016570 | histone modification | 17 | 0 | 0.53 | 1 | 1 |
| GO:0016571 | histone methylation | 9 | 0 | 0.28 | 1 | 1 |
| GO:0016573 | histone acetylation | 5 | 0 | 0.16 | 1 | 1 |
| GO:0016579 | protein deubiquitination | 43 | 0 | 1.35 | 1 | 1 |
| GO:0017004 | cytochrome complex assembly | 11 | 0 | 0.34 | 1 | 1 |
| GO:0017006 | protein-tetrapyrrole linkage | 5 | 0 | 0.16 | 1 | 1 |
| GO:0017038 | protein import | 14 | 0 | 0.44 | 1 | 1 |
| GO:0018022 | peptidyl-lysine methylation | 9 | 0 | 0.28 | 1 | 1 |
| GO:0018193 | peptidyl-amino acid modification | 73 | 0 | 2.29 | 1 | 1 |
| GO:0018205 | peptidyl-lysine modification | 17 | 0 | 0.53 | 1 | 1 |
| GO:0018208 | peptidyl-proline modification | 46 | 0 | 1.44 | 1 | 1 |
| GO:0018393 | internal peptidyl-lysine acetylation | 5 | 0 | 0.16 | 1 | 1 |
| GO:0018394 | peptidyl-lysine acetylation | 5 | 0 | 0.16 | 1 | 1 |
| GO:0019184 | nonribosomal peptide biosynthetic proces... | 5 | 0 | 0.16 | 1 | 1 |
| GO:0019288 | isopentenyl diphosphate biosynthetic pro... | 5 | 0 | 0.16 | 1 | 1 |
| GO:0019310 | inositol catabolic process | 8 | 0 | 0.25 | 1 | 1 |
| GO:0019319 | hexose biosynthetic process | 17 | 0 | 0.53 | 1 | 1 |
| GO:0019344 | cysteine biosynthetic process | 6 | 0 | 0.19 | 1 | 1 |
| GO:0019359 | nicotinamide nucleotide biosynthetic pro... | 11 | 0 | 0.34 | 1 | 1 |
| GO:0019363 | pyridine nucleotide biosynthetic process | 11 | 0 | 0.34 | 1 | 1 |
| GO:0019395 | fatty acid oxidation | 11 | 0 | 0.34 | 1 | 1 |
| GO:0019400 | alditol metabolic process | 9 | 0 | 0.28 | 1 | 1 |
| GO:0019439 | aromatic compound catabolic process | 39 | 0 | 1.22 | 1 | 1 |
| GO:0019441 | tryptophan catabolic process to kynureni... | 6 | 0 | 0.19 | 1 | 1 |
| GO:0019674 | NAD metabolic process | 5 | 0 | 0.16 | 1 | 1 |
| GO:0019682 | glyceraldehyde-3-phosphate metabolic pro... | 15 | 0 | 0.47 | 1 | 1 |
| GO:0019684 | photosynthesis, light reaction | 12 | 0 | 0.38 | 1 | 1 |
| GO:0019720 | Mo-molybdopterin cofactor metabolic proc... | 5 | 0 | 0.16 | 1 | 1 |
| GO:0019751 | polyol metabolic process | 23 | 0 | 0.72 | 1 | 1 |
| GO:0022402 | cell cycle process | 16 | 0 | 0.5 | 1 | 1 |
| GO:0022406 | membrane docking | 13 | 0 | 0.41 | 1 | 1 |
| GO:0022411 | cellular component disassembly | 26 | 0 | 0.81 | 1 | 1 |
| GO:0022610 | biological adhesion | 47 | 0 | 1.47 | 1 | 1 |
| GO:0022613 | ribonucleoprotein complex biogenesis | 44 | 0 | 1.38 | 1 | 1 |
| GO:0022618 | ribonucleoprotein complex assembly | 5 | 0 | 0.16 | 1 | 1 |
| GO:0022900 | electron transport chain | 26 | 0 | 0.81 | 1 | 1 |
| GO:0022904 | respiratory electron transport chain | 16 | 0 | 0.5 | 1 | 1 |
| GO:0023051 | regulation of signaling | 24 | 0 | 0.75 | 1 | 1 |
| GO:0030003 | cellular cation homeostasis | 9 | 0 | 0.28 | 1 | 1 |
| GO:0030029 | actin filament-based process | 16 | 0 | 0.5 | 1 | 1 |
| GO:0030030 | cell projection organization | 21 | 0 | 0.66 | 1 | 1 |
| GO:0030031 | cell projection assembly | 21 | 0 | 0.66 | 1 | 1 |
| GO:0030036 | actin cytoskeleton organization | 16 | 0 | 0.5 | 1 | 1 |
| GO:0030041 | actin filament polymerization | 13 | 0 | 0.41 | 1 | 1 |
| GO:0030042 | actin filament depolymerization | 8 | 0 | 0.25 | 1 | 1 |
| GO:0030150 | protein import into mitochondrial matrix | 5 | 0 | 0.16 | 1 | 1 |
| GO:0030198 | extracellular matrix organization | 9 | 0 | 0.28 | 1 | 1 |
| GO:0030203 | glycosaminoglycan metabolic process | 15 | 0 | 0.47 | 1 | 1 |
| GO:0030258 | lipid modification | 25 | 0 | 0.78 | 1 | 1 |
| GO:0030832 | regulation of actin filament length | 13 | 0 | 0.41 | 1 | 1 |
| GO:0030833 | regulation of actin filament polymerizat... | 13 | 0 | 0.41 | 1 | 1 |
| GO:0030834 | regulation of actin filament depolymeriz... | 8 | 0 | 0.25 | 1 | 1 |
| GO:0030835 | negative regulation of actin filament de... | 8 | 0 | 0.25 | 1 | 1 |
| GO:0030837 | negative regulation of actin filament po... | 8 | 0 | 0.25 | 1 | 1 |
| GO:0030838 | positive regulation of actin filament po... | 5 | 0 | 0.16 | 1 | 1 |
| GO:0031047 | gene silencing by RNA | 7 | 0 | 0.22 | 1 | 1 |
| GO:0031123 | RNA 3'-end processing | 7 | 0 | 0.22 | 1 | 1 |
| GO:0031124 | mRNA 3'-end processing | 7 | 0 | 0.22 | 1 | 1 |
| GO:0031163 | metallo-sulfur cluster assembly | 13 | 0 | 0.41 | 1 | 1 |
| GO:0031325 | positive regulation of cellular metaboli... | 10 | 0 | 0.31 | 1 | 1 |
| GO:0031328 | positive regulation of cellular biosynth... | 10 | 0 | 0.31 | 1 | 1 |
| GO:0031333 | negative regulation of protein complex a... | 8 | 0 | 0.25 | 1 | 1 |
| GO:0031334 | positive regulation of protein complex a... | 5 | 0 | 0.16 | 1 | 1 |
| GO:0032011 | ARF protein signal transduction | 5 | 0 | 0.16 | 1 | 1 |
| GO:0032012 | regulation of ARF protein signal transdu... | 5 | 0 | 0.16 | 1 | 1 |
| GO:0032196 | transposition | 11 | 0 | 0.34 | 1 | 1 |
| GO:0032259 | methylation | 32 | 0 | 1 | 1 | 1 |
| GO:0032271 | regulation of protein polymerization | 13 | 0 | 0.41 | 1 | 1 |
| GO:0032272 | negative regulation of protein polymeriz... | 8 | 0 | 0.25 | 1 | 1 |
| GO:0032273 | positive regulation of protein polymeriz... | 5 | 0 | 0.16 | 1 | 1 |
| GO:0032502 | developmental process | 27 | 0 | 0.85 | 1 | 1 |
| GO:0032507 | maintenance of protein location in cell | 16 | 0 | 0.5 | 1 | 1 |
| GO:0032535 | regulation of cellular component size | 13 | 0 | 0.41 | 1 | 1 |
| GO:0032784 | regulation of DNA-templated transcriptio... | 5 | 0 | 0.16 | 1 | 1 |
| GO:0032879 | regulation of localization | 6 | 0 | 0.19 | 1 | 1 |
| GO:0032956 | regulation of actin cytoskeleton organiz... | 13 | 0 | 0.41 | 1 | 1 |
| GO:0032970 | regulation of actin filament-based proce... | 13 | 0 | 0.41 | 1 | 1 |
| GO:0032984 | macromolecular complex disassembly | 26 | 0 | 0.81 | 1 | 1 |
| GO:0033015 | tetrapyrrole catabolic process | 7 | 0 | 0.22 | 1 | 1 |
| GO:0033043 | regulation of organelle organization | 17 | 0 | 0.53 | 1 | 1 |
| GO:0033365 | protein localization to organelle | 40 | 0 | 1.25 | 1 | 1 |
| GO:0033865 | nucleoside bisphosphate metabolic proces... | 12 | 0 | 0.38 | 1 | 1 |
| GO:0033875 | ribonucleoside bisphosphate metabolic pr... | 12 | 0 | 0.38 | 1 | 1 |
| GO:0034032 | purine nucleoside bisphosphate metabolic... | 12 | 0 | 0.38 | 1 | 1 |
| GO:0034035 | purine ribonucleoside bisphosphate metab... | 5 | 0 | 0.16 | 1 | 1 |
| GO:0034219 | carbohydrate transmembrane transport | 15 | 0 | 0.47 | 1 | 1 |
| GO:0034314 | Arp2/3 complex-mediated actin nucleation | 5 | 0 | 0.16 | 1 | 1 |
| GO:0034440 | lipid oxidation | 11 | 0 | 0.34 | 1 | 1 |
| GO:0034470 | ncRNA processing | 73 | 0 | 2.29 | 1 | 1 |
| GO:0034655 | nucleobase-containing compound catabolic... | 22 | 0 | 0.69 | 1 | 1 |
| GO:0034754 | cellular hormone metabolic process | 9 | 0 | 0.28 | 1 | 1 |
| GO:0034968 | histone lysine methylation | 9 | 0 | 0.28 | 1 | 1 |
| GO:0035023 | regulation of Rho protein signal transdu... | 5 | 0 | 0.16 | 1 | 1 |
| GO:0035429 | gluconate transmembrane transport | 13 | 0 | 0.41 | 1 | 1 |
| GO:0035434 | copper ion transmembrane transport | 6 | 0 | 0.19 | 1 | 1 |
| GO:0035437 | maintenance of protein localization in e... | 14 | 0 | 0.44 | 1 | 1 |
| GO:0040007 | growth | 9 | 0 | 0.28 | 1 | 1 |
| GO:0040011 | locomotion | 17 | 0 | 0.53 | 1 | 1 |
| GO:0042147 | retrograde transport, endosome to Golgi | 7 | 0 | 0.22 | 1 | 1 |
| GO:0042168 | heme metabolic process | 9 | 0 | 0.28 | 1 | 1 |
| GO:0042180 | cellular ketone metabolic process | 7 | 0 | 0.22 | 1 | 1 |
| GO:0042219 | cellular modified amino acid catabolic p... | 5 | 0 | 0.16 | 1 | 1 |
| GO:0042254 | ribosome biogenesis | 40 | 0 | 1.25 | 1 | 1 |
| GO:0042278 | purine nucleoside metabolic process | 10 | 0 | 0.31 | 1 | 1 |
| GO:0042330 | taxis | 7 | 0 | 0.22 | 1 | 1 |
| GO:0042401 | cellular biogenic amine biosynthetic pro... | 10 | 0 | 0.31 | 1 | 1 |
| GO:0042402 | cellular biogenic amine catabolic proces... | 6 | 0 | 0.19 | 1 | 1 |
| GO:0042430 | indole-containing compound metabolic pro... | 16 | 0 | 0.5 | 1 | 1 |
| GO:0042436 | indole-containing compound catabolic pro... | 6 | 0 | 0.19 | 1 | 1 |
| GO:0042440 | pigment metabolic process | 13 | 0 | 0.41 | 1 | 1 |
| GO:0042445 | hormone metabolic process | 9 | 0 | 0.28 | 1 | 1 |
| GO:0042455 | ribonucleoside biosynthetic process | 6 | 0 | 0.19 | 1 | 1 |
| GO:0042537 | benzene-containing compound metabolic pr... | 6 | 0 | 0.19 | 1 | 1 |
| GO:0042546 | cell wall biogenesis | 16 | 0 | 0.5 | 1 | 1 |
| GO:0042548 | regulation of photosynthesis, light reac... | 5 | 0 | 0.16 | 1 | 1 |
| GO:0042549 | photosystem II stabilization | 5 | 0 | 0.16 | 1 | 1 |
| GO:0042558 | pteridine-containing compound metabolic ... | 9 | 0 | 0.28 | 1 | 1 |
| GO:0042723 | thiamine-containing compound metabolic p... | 8 | 0 | 0.25 | 1 | 1 |
| GO:0042724 | thiamine-containing compound biosyntheti... | 8 | 0 | 0.25 | 1 | 1 |
| GO:0042773 | ATP synthesis coupled electron transport | 9 | 0 | 0.28 | 1 | 1 |
| GO:0042775 | mitochondrial ATP synthesis coupled elec... | 8 | 0 | 0.25 | 1 | 1 |
| GO:0042873 | aldonate transport | 14 | 0 | 0.44 | 1 | 1 |
| GO:0043062 | extracellular structure organization | 9 | 0 | 0.28 | 1 | 1 |
| GO:0043085 | positive regulation of catalytic activit... | 5 | 0 | 0.16 | 1 | 1 |
| GO:0043161 | proteasome-mediated ubiquitin-dependent ... | 7 | 0 | 0.22 | 1 | 1 |
| GO:0043241 | protein complex disassembly | 25 | 0 | 0.78 | 1 | 1 |
| GO:0043242 | negative regulation of protein complex d... | 9 | 0 | 0.28 | 1 | 1 |
| GO:0043244 | regulation of protein complex disassembl... | 13 | 0 | 0.41 | 1 | 1 |
| GO:0043248 | proteasome assembly | 6 | 0 | 0.19 | 1 | 1 |
| GO:0043254 | regulation of protein complex assembly | 13 | 0 | 0.41 | 1 | 1 |
| GO:0043269 | regulation of ion transport | 5 | 0 | 0.16 | 1 | 1 |
| GO:0043414 | macromolecule methylation | 24 | 0 | 0.75 | 1 | 1 |
| GO:0043467 | regulation of generation of precursor me... | 5 | 0 | 0.16 | 1 | 1 |
| GO:0043543 | protein acylation | 6 | 0 | 0.19 | 1 | 1 |
| GO:0043545 | molybdopterin cofactor metabolic process | 8 | 0 | 0.25 | 1 | 1 |
| GO:0043623 | cellular protein complex assembly | 43 | 0 | 1.35 | 1 | 1 |
| GO:0043624 | cellular protein complex disassembly | 24 | 0 | 0.75 | 1 | 1 |
| GO:0043631 | RNA polyadenylation | 9 | 0 | 0.28 | 1 | 1 |
| GO:0043648 | dicarboxylic acid metabolic process | 16 | 0 | 0.5 | 1 | 1 |
| GO:0043650 | dicarboxylic acid biosynthetic process | 5 | 0 | 0.16 | 1 | 1 |
| GO:0043711 | pilus organization | 20 | 0 | 0.63 | 1 | 1 |
| GO:0044087 | regulation of cellular component biogene... | 14 | 0 | 0.44 | 1 | 1 |
| GO:0044089 | positive regulation of cellular componen... | 5 | 0 | 0.16 | 1 | 1 |
| GO:0044093 | positive regulation of molecular functio... | 5 | 0 | 0.16 | 1 | 1 |
| GO:0044106 | cellular amine metabolic process | 26 | 0 | 0.81 | 1 | 1 |
| GO:0044242 | cellular lipid catabolic process | 12 | 0 | 0.38 | 1 | 1 |
| GO:0044270 | cellular nitrogen compound catabolic pro... | 36 | 0 | 1.13 | 1 | 1 |
| GO:0044272 | sulfur compound biosynthetic process | 41 | 0 | 1.28 | 1 | 1 |
| GO:0044273 | sulfur compound catabolic process | 5 | 0 | 0.16 | 1 | 1 |
| GO:0044275 | cellular carbohydrate catabolic process | 10 | 0 | 0.31 | 1 | 1 |
| GO:0044282 | small molecule catabolic process | 43 | 0 | 1.35 | 1 | 1 |
| GO:0044419 | interspecies interaction between organis... | 6 | 0 | 0.19 | 1 | 1 |
| GO:0044743 | protein transmembrane import into intrac... | 6 | 0 | 0.19 | 1 | 1 |
| GO:0045010 | actin nucleation | 5 | 0 | 0.16 | 1 | 1 |
| GO:0045047 | protein targeting to ER | 17 | 0 | 0.53 | 1 | 1 |
| GO:0045185 | maintenance of protein location | 16 | 0 | 0.5 | 1 | 1 |
| GO:0045333 | cellular respiration | 30 | 0 | 0.94 | 1 | 1 |
| GO:0045892 | negative regulation of transcription, DN... | 6 | 0 | 0.19 | 1 | 1 |
| GO:0045893 | positive regulation of transcription, DN... | 6 | 0 | 0.19 | 1 | 1 |
| GO:0045934 | negative regulation of nucleobase-contai... | 7 | 0 | 0.22 | 1 | 1 |
| GO:0045935 | positive regulation of nucleobase-contai... | 6 | 0 | 0.19 | 1 | 1 |
| GO:0046128 | purine ribonucleoside metabolic process | 10 | 0 | 0.31 | 1 | 1 |
| GO:0046148 | pigment biosynthetic process | 6 | 0 | 0.19 | 1 | 1 |
| GO:0046164 | alcohol catabolic process | 8 | 0 | 0.25 | 1 | 1 |
| GO:0046165 | alcohol biosynthetic process | 6 | 0 | 0.19 | 1 | 1 |
| GO:0046168 | glycerol-3-phosphate catabolic process | 5 | 0 | 0.16 | 1 | 1 |
| GO:0046173 | polyol biosynthetic process | 6 | 0 | 0.19 | 1 | 1 |
| GO:0046174 | polyol catabolic process | 8 | 0 | 0.25 | 1 | 1 |
| GO:0046218 | indolalkylamine catabolic process | 6 | 0 | 0.19 | 1 | 1 |
| GO:0046351 | disaccharide biosynthetic process | 36 | 0 | 1.13 | 1 | 1 |
| GO:0046364 | monosaccharide biosynthetic process | 17 | 0 | 0.53 | 1 | 1 |
| GO:0046365 | monosaccharide catabolic process | 6 | 0 | 0.19 | 1 | 1 |
| GO:0046390 | ribose phosphate biosynthetic process | 47 | 0 | 1.47 | 1 | 1 |
| GO:0046395 | carboxylic acid catabolic process | 33 | 0 | 1.03 | 1 | 1 |
| GO:0046434 | organophosphate catabolic process | 12 | 0 | 0.38 | 1 | 1 |
| GO:0046451 | diaminopimelate metabolic process | 7 | 0 | 0.22 | 1 | 1 |
| GO:0046487 | glyoxylate metabolic process | 9 | 0 | 0.28 | 1 | 1 |
| GO:0046490 | isopentenyl diphosphate metabolic proces... | 5 | 0 | 0.16 | 1 | 1 |
| GO:0046493 | lipid A metabolic process | 9 | 0 | 0.28 | 1 | 1 |
| GO:0046500 | S-adenosylmethionine metabolic process | 15 | 0 | 0.47 | 1 | 1 |
| GO:0046578 | regulation of Ras protein signal transdu... | 10 | 0 | 0.31 | 1 | 1 |
| GO:0046700 | heterocycle catabolic process | 36 | 0 | 1.13 | 1 | 1 |
| GO:0046834 | lipid phosphorylation | 10 | 0 | 0.31 | 1 | 1 |
| GO:0046854 | phosphatidylinositol phosphorylation | 10 | 0 | 0.31 | 1 | 1 |
| GO:0046916 | cellular transition metal ion homeostasi... | 9 | 0 | 0.28 | 1 | 1 |
| GO:0048193 | Golgi vesicle transport | 26 | 0 | 0.81 | 1 | 1 |
| GO:0048278 | vesicle docking | 13 | 0 | 0.41 | 1 | 1 |
| GO:0048285 | organelle fission | 15 | 0 | 0.47 | 1 | 1 |
| GO:0048518 | positive regulation of biological proces... | 15 | 0 | 0.47 | 1 | 1 |
| GO:0048522 | positive regulation of cellular process | 15 | 0 | 0.47 | 1 | 1 |
| GO:0048856 | anatomical structure development | 25 | 0 | 0.78 | 1 | 1 |
| GO:0048869 | cellular developmental process | 5 | 0 | 0.16 | 1 | 1 |
| GO:0048870 | cell motility | 10 | 0 | 0.31 | 1 | 1 |
| GO:0048878 | chemical homeostasis | 9 | 0 | 0.28 | 1 | 1 |
| GO:0050801 | ion homeostasis | 9 | 0 | 0.28 | 1 | 1 |
| GO:0050992 | dimethylallyl diphosphate biosynthetic p... | 5 | 0 | 0.16 | 1 | 1 |
| GO:0050993 | dimethylallyl diphosphate metabolic proc... | 5 | 0 | 0.16 | 1 | 1 |
| GO:0051016 | barbed-end actin filament capping | 8 | 0 | 0.25 | 1 | 1 |
| GO:0051049 | regulation of transport | 5 | 0 | 0.16 | 1 | 1 |
| GO:0051052 | regulation of DNA metabolic process | 6 | 0 | 0.19 | 1 | 1 |
| GO:0051056 | regulation of small GTPase mediated sign... | 10 | 0 | 0.31 | 1 | 1 |
| GO:0051128 | regulation of cellular component organiz... | 24 | 0 | 0.75 | 1 | 1 |
| GO:0051129 | negative regulation of cellular componen... | 10 | 0 | 0.31 | 1 | 1 |
| GO:0051130 | positive regulation of cellular componen... | 9 | 0 | 0.28 | 1 | 1 |
| GO:0051156 | glucose 6-phosphate metabolic process | 11 | 0 | 0.34 | 1 | 1 |
| GO:0051169 | nuclear transport | 7 | 0 | 0.22 | 1 | 1 |
| GO:0051173 | positive regulation of nitrogen compound... | 10 | 0 | 0.31 | 1 | 1 |
| GO:0051187 | cofactor catabolic process | 7 | 0 | 0.22 | 1 | 1 |
| GO:0051189 | prosthetic group metabolic process | 8 | 0 | 0.25 | 1 | 1 |
| GO:0051253 | negative regulation of RNA metabolic pro... | 6 | 0 | 0.19 | 1 | 1 |
| GO:0051254 | positive regulation of RNA metabolic pro... | 6 | 0 | 0.19 | 1 | 1 |
| GO:0051258 | protein polymerization | 13 | 0 | 0.41 | 1 | 1 |
| GO:0051259 | protein oligomerization | 12 | 0 | 0.38 | 1 | 1 |
| GO:0051260 | protein homooligomerization | 7 | 0 | 0.22 | 1 | 1 |
| GO:0051261 | protein depolymerization | 8 | 0 | 0.25 | 1 | 1 |
| GO:0051301 | cell division | 14 | 0 | 0.44 | 1 | 1 |
| GO:0051336 | regulation of hydrolase activity | 6 | 0 | 0.19 | 1 | 1 |
| GO:0051493 | regulation of cytoskeleton organization | 15 | 0 | 0.47 | 1 | 1 |
| GO:0051494 | negative regulation of cytoskeleton orga... | 8 | 0 | 0.25 | 1 | 1 |
| GO:0051495 | positive regulation of cytoskeleton orga... | 5 | 0 | 0.16 | 1 | 1 |
| GO:0051604 | protein maturation | 15 | 0 | 0.47 | 1 | 1 |
| GO:0051640 | organelle localization | 13 | 0 | 0.41 | 1 | 1 |
| GO:0051651 | maintenance of location in cell | 16 | 0 | 0.5 | 1 | 1 |
| GO:0051674 | localization of cell | 10 | 0 | 0.31 | 1 | 1 |
| GO:0051693 | actin filament capping | 8 | 0 | 0.25 | 1 | 1 |
| GO:0052646 | alditol phosphate metabolic process | 5 | 0 | 0.16 | 1 | 1 |
| GO:0052803 | imidazole-containing compound metabolic ... | 14 | 0 | 0.44 | 1 | 1 |
| GO:0055065 | metal ion homeostasis | 9 | 0 | 0.28 | 1 | 1 |
| GO:0055072 | iron ion homeostasis | 9 | 0 | 0.28 | 1 | 1 |
| GO:0055076 | transition metal ion homeostasis | 9 | 0 | 0.28 | 1 | 1 |
| GO:0055080 | cation homeostasis | 9 | 0 | 0.28 | 1 | 1 |
| GO:0055082 | cellular chemical homeostasis | 9 | 0 | 0.28 | 1 | 1 |
| GO:0061024 | membrane organization | 9 | 0 | 0.28 | 1 | 1 |
| GO:0065002 | intracellular protein transmembrane tran... | 6 | 0 | 0.19 | 1 | 1 |
| GO:0070189 | kynurenine metabolic process | 6 | 0 | 0.19 | 1 | 1 |
| GO:0070271 | protein complex biogenesis | 55 | 0 | 1.72 | 1 | 1 |
| GO:0070585 | protein localization to mitochondrion | 5 | 0 | 0.16 | 1 | 1 |
| GO:0070646 | protein modification by small protein re... | 43 | 0 | 1.35 | 1 | 1 |
| GO:0070726 | cell wall assembly | 9 | 0 | 0.28 | 1 | 1 |
| GO:0070838 | divalent metal ion transport | 13 | 0 | 0.41 | 1 | 1 |
| GO:0070925 | organelle assembly | 6 | 0 | 0.19 | 1 | 1 |
| GO:0070972 | protein localization to endoplasmic reti... | 31 | 0 | 0.97 | 1 | 1 |
| GO:0071668 | plant-type cell wall assembly | 9 | 0 | 0.28 | 1 | 1 |
| GO:0071669 | plant-type cell wall organization or bio... | 11 | 0 | 0.34 | 1 | 1 |
| GO:0071806 | protein transmembrane transport | 8 | 0 | 0.25 | 1 | 1 |
| GO:0071822 | protein complex subunit organization | 72 | 0 | 2.26 | 1 | 1 |
| GO:0071826 | ribonucleoprotein complex subunit organi... | 6 | 0 | 0.19 | 1 | 1 |
| GO:0071973 | bacterial-type flagellum-dependent cell ... | 9 | 0 | 0.28 | 1 | 1 |
| GO:0072329 | monocarboxylic acid catabolic process | 17 | 0 | 0.53 | 1 | 1 |
| GO:0072348 | sulfur compound transport | 24 | 0 | 0.75 | 1 | 1 |
| GO:0072350 | tricarboxylic acid metabolic process | 11 | 0 | 0.34 | 1 | 1 |
| GO:0072511 | divalent inorganic cation transport | 13 | 0 | 0.41 | 1 | 1 |
| GO:0072522 | purine-containing compound biosynthetic ... | 58 | 0 | 1.82 | 1 | 1 |
| GO:0072525 | pyridine-containing compound biosyntheti... | 14 | 0 | 0.44 | 1 | 1 |
| GO:0072527 | pyrimidine-containing compound metabolic... | 21 | 0 | 0.66 | 1 | 1 |
| GO:0072528 | pyrimidine-containing compound biosynthe... | 18 | 0 | 0.56 | 1 | 1 |
| GO:0072531 | pyrimidine-containing compound transmemb... | 7 | 0 | 0.22 | 1 | 1 |
| GO:0072593 | reactive oxygen species metabolic proces... | 12 | 0 | 0.38 | 1 | 1 |
| GO:0072594 | establishment of protein localization to... | 26 | 0 | 0.81 | 1 | 1 |
| GO:0072595 | maintenance of protein localization in o... | 14 | 0 | 0.44 | 1 | 1 |
| GO:0072599 | establishment of protein localization to... | 17 | 0 | 0.53 | 1 | 1 |
| GO:0072655 | establishment of protein localization to... | 5 | 0 | 0.16 | 1 | 1 |
| GO:0072657 | protein localization to membrane | 18 | 0 | 0.56 | 1 | 1 |
| GO:0090066 | regulation of anatomical structure size | 13 | 0 | 0.41 | 1 | 1 |
| GO:0090150 | establishment of protein localization to... | 18 | 0 | 0.56 | 1 | 1 |
| GO:0090481 | pyrimidine nucleotide-sugar transmembran... | 7 | 0 | 0.22 | 1 | 1 |
| GO:0090662 | ATP hydrolysis coupled transmembrane tra... | 28 | 0 | 0.88 | 1 | 1 |
| GO:0097164 | ammonium ion metabolic process | 8 | 0 | 0.25 | 1 | 1 |
| GO:0097435 | supramolecular fiber organization | 15 | 0 | 0.47 | 1 | 1 |
| GO:0097588 | archaeal or bacterial-type flagellum-dep... | 9 | 0 | 0.28 | 1 | 1 |
| GO:0098609 | cell-cell adhesion | 16 | 0 | 0.5 | 1 | 1 |
| GO:0098657 | import into cell | 5 | 0 | 0.16 | 1 | 1 |
| GO:0098742 | cell-cell adhesion via plasma-membrane a... | 16 | 0 | 0.5 | 1 | 1 |
| GO:0098771 | inorganic ion homeostasis | 9 | 0 | 0.28 | 1 | 1 |
| GO:0098813 | nuclear chromosome segregation | 9 | 0 | 0.28 | 1 | 1 |
| GO:0099131 | ATP hydrolysis coupled ion transmembrane... | 28 | 0 | 0.88 | 1 | 1 |
| GO:0099132 | ATP hydrolysis coupled cation transmembr... | 28 | 0 | 0.88 | 1 | 1 |
| GO:0110053 | regulation of actin filament organizatio... | 13 | 0 | 0.41 | 1 | 1 |
| GO:0120009 | intermembrane lipid transfer | 8 | 0 | 0.25 | 1 | 1 |
| GO:0140014 | mitotic nuclear division | 7 | 0 | 0.22 | 1 | 1 |
| GO:0140029 | exocytic process | 12 | 0 | 0.38 | 1 | 1 |
| GO:0140056 | organelle localization by membrane tethe... | 13 | 0 | 0.41 | 1 | 1 |
| GO:0198738 | cell-cell signaling by wnt | 6 | 0 | 0.19 | 1 | 1 |
| GO:1901068 | guanosine-containing compound metabolic ... | 9 | 0 | 0.28 | 1 | 1 |
| GO:1901264 | carbohydrate derivative transport | 18 | 0 | 0.56 | 1 | 1 |
| GO:1901269 | lipooligosaccharide metabolic process | 9 | 0 | 0.28 | 1 | 1 |
| GO:1901271 | lipooligosaccharide biosynthetic process | 9 | 0 | 0.28 | 1 | 1 |
| GO:1901292 | nucleoside phosphate catabolic process | 6 | 0 | 0.19 | 1 | 1 |
| GO:1901293 | nucleoside phosphate biosynthetic proces... | 76 | 0 | 2.38 | 1 | 1 |
| GO:1901361 | organic cyclic compound catabolic proces... | 37 | 0 | 1.16 | 1 | 1 |
| GO:1901606 | alpha-amino acid catabolic process | 14 | 0 | 0.44 | 1 | 1 |
| GO:1901615 | organic hydroxy compound metabolic proce... | 27 | 0 | 0.85 | 1 | 1 |
| GO:1901616 | organic hydroxy compound catabolic proce... | 8 | 0 | 0.25 | 1 | 1 |
| GO:1901617 | organic hydroxy compound biosynthetic pr... | 9 | 0 | 0.28 | 1 | 1 |
| GO:1901642 | nucleoside transmembrane transport | 6 | 0 | 0.19 | 1 | 1 |
| GO:1901657 | glycosyl compound metabolic process | 38 | 0 | 1.19 | 1 | 1 |
| GO:1901659 | glycosyl compound biosynthetic process | 6 | 0 | 0.19 | 1 | 1 |
| GO:1901700 | response to oxygen-containing compound | 6 | 0 | 0.19 | 1 | 1 |
| GO:1901879 | regulation of protein depolymerization | 8 | 0 | 0.25 | 1 | 1 |
| GO:1901880 | negative regulation of protein depolymer... | 8 | 0 | 0.25 | 1 | 1 |
| GO:1902221 | erythrose 4-phosphate/phosphoenolpyruvat... | 6 | 0 | 0.19 | 1 | 1 |
| GO:1902223 | erythrose 4-phosphate/phosphoenolpyruvat... | 5 | 0 | 0.16 | 1 | 1 |
| GO:1902531 | regulation of intracellular signal trans... | 10 | 0 | 0.31 | 1 | 1 |
| GO:1902679 | negative regulation of RNA biosynthetic ... | 6 | 0 | 0.19 | 1 | 1 |
| GO:1902680 | positive regulation of RNA biosynthetic ... | 6 | 0 | 0.19 | 1 | 1 |
| GO:1902903 | regulation of supramolecular fiber organ... | 13 | 0 | 0.41 | 1 | 1 |
| GO:1902904 | negative regulation of supramolecular fi... | 8 | 0 | 0.25 | 1 | 1 |
| GO:1902905 | positive regulation of supramolecular fi... | 5 | 0 | 0.16 | 1 | 1 |
| GO:1903047 | mitotic cell cycle process | 8 | 0 | 0.25 | 1 | 1 |
| GO:1903507 | negative regulation of nucleic acid-temp... | 6 | 0 | 0.19 | 1 | 1 |
| GO:1903508 | positive regulation of nucleic acid-temp... | 6 | 0 | 0.19 | 1 | 1 |
| GO:1905114 | cell surface receptor signaling pathway ... | 6 | 0 | 0.19 | 1 | 1 |

**2-CC**

| GO ID | Term | Annotated nuigene number | Significant unigene number | Expected | Classic Fisher | FDR |
| --- | --- | --- | --- | --- | --- | --- |
| GO:0009349 | riboflavin synthase complex | 7 | 4 | 1.70E-01 | 1.10E-05 | 0.00264 |
| GO:0009538 | photosystem I reaction center | 13 | 3 | 0.31 | 0.0033 | 0.396 |
| GO:0009522 | photosystem I | 17 | 3 | 0.41 | 0.0073 | 0.584 |
| GO:1990234 | transferase complex | 75 | 5 | 1.81 | 0.0339 | 1 |
| GO:0009521 | photosystem | 41 | 3 | 0.99 | 0.0754 | 1 |
| GO:0034357 | photosynthetic membrane | 42 | 3 | 1.02 | 0.0799 | 1 |
| GO:0009579 | thylakoid | 43 | 3 | 1.04 | 0.0844 | 1 |
| GO:0044436 | thylakoid part | 43 | 3 | 1.04 | 0.0844 | 1 |
| GO:0005840 | ribosome | 392 | 14 | 9.48 | 0.0865 | 1 |
| GO:0030529 | intracellular ribonucleoprotein complex | 415 | 14 | 10.03 | 0.1228 | 1 |
| GO:1990904 | ribonucleoprotein complex | 415 | 14 | 10.03 | 0.1228 | 1 |
| GO:0046658 | anchored component of plasma membrane | 9 | 1 | 0.22 | 0.1978 | 1 |
| GO:0043232 | intracellular non-membrane-bounded organ... | 571 | 17 | 13.8 | 0.2097 | 1 |
| GO:0043228 | non-membrane-bounded organelle | 573 | 17 | 13.85 | 0.2139 | 1 |
| GO:0000786 | nucleosome | 49 | 2 | 1.18 | 0.3329 | 1 |
| GO:0032993 | protein-DNA complex | 49 | 2 | 1.18 | 0.3329 | 1 |
| GO:0044815 | DNA packaging complex | 50 | 2 | 1.21 | 0.3418 | 1 |
| GO:0031225 | anchored component of membrane | 18 | 1 | 0.44 | 0.3569 | 1 |
| GO:0048046 | apoplast | 18 | 1 | 0.44 | 0.3569 | 1 |
| GO:0000785 | chromatin | 52 | 2 | 1.26 | 0.3595 | 1 |
| GO:0032991 | macromolecular complex | 1043 | 27 | 25.21 | 0.375 | 1 |
| GO:0030880 | RNA polymerase complex | 21 | 1 | 0.51 | 0.4026 | 1 |
| GO:0005618 | cell wall | 22 | 1 | 0.53 | 0.4171 | 1 |
| GO:0031226 | intrinsic component of plasma membrane | 23 | 1 | 0.56 | 0.4313 | 1 |
| GO:0030312 | external encapsulating structure | 26 | 1 | 0.63 | 0.4718 | 1 |
| GO:0061695 | transferase complex, transferring phosph... | 27 | 1 | 0.65 | 0.4847 | 1 |
| GO:0000145 | exocyst | 30 | 1 | 0.73 | 0.5214 | 1 |
| GO:0005938 | cell cortex | 30 | 1 | 0.73 | 0.5214 | 1 |
| GO:0044448 | cell cortex part | 30 | 1 | 0.73 | 0.5214 | 1 |
| GO:0099568 | cytoplasmic region | 30 | 1 | 0.73 | 0.5214 | 1 |
| GO:0044427 | chromosomal part | 72 | 2 | 1.74 | 0.524 | 1 |
| GO:0031224 | intrinsic component of membrane | 1070 | 26 | 25.87 | 0.5272 | 1 |
| GO:0016020 | membrane | 2437 | 59 | 58.91 | 0.5381 | 1 |
| GO:0016021 | integral component of membrane | 1052 | 25 | 25.43 | 0.5798 | 1 |
| GO:0099023 | tethering complex | 37 | 1 | 0.89 | 0.5973 | 1 |
| GO:1902494 | catalytic complex | 216 | 5 | 5.22 | 0.6057 | 1 |
| GO:0071944 | cell periphery | 132 | 3 | 3.19 | 0.6255 | 1 |
| GO:0005694 | chromosome | 87 | 2 | 2.1 | 0.6278 | 1 |
| GO:0016459 | myosin complex | 41 | 1 | 0.99 | 0.6352 | 1 |
| GO:0005654 | nucleoplasm | 48 | 1 | 1.16 | 0.6933 | 1 |
| GO:0044451 | nucleoplasm part | 48 | 1 | 1.16 | 0.6933 | 1 |
| GO:0044425 | membrane part | 1278 | 29 | 30.89 | 0.6989 | 1 |
| GO:0005623 | cell | 1756 | 40 | 42.45 | 0.7302 | 1 |
| GO:0044464 | cell part | 1756 | 40 | 42.45 | 0.7302 | 1 |
| GO:0015629 | actin cytoskeleton | 55 | 1 | 1.33 | 0.7421 | 1 |
| GO:0005634 | nucleus | 402 | 8 | 9.72 | 0.7708 | 1 |
| GO:0044444 | cytoplasmic part | 716 | 15 | 17.31 | 0.7714 | 1 |
| GO:0005576 | extracellular region | 61 | 1 | 1.47 | 0.7778 | 1 |
| GO:0044459 | plasma membrane part | 64 | 1 | 1.55 | 0.7938 | 1 |
| GO:0031981 | nuclear lumen | 65 | 1 | 1.57 | 0.7989 | 1 |
| GO:0044430 | cytoskeletal part | 66 | 1 | 1.6 | 0.8038 | 1 |
| GO:0005737 | cytoplasm | 834 | 17 | 20.16 | 0.823 | 1 |
| GO:0005886 | plasma membrane | 76 | 1 | 1.84 | 0.8471 | 1 |
| GO:0031974 | membrane-enclosed lumen | 79 | 1 | 1.91 | 0.8581 | 1 |
| GO:0043233 | organelle lumen | 79 | 1 | 1.91 | 0.8581 | 1 |
| GO:0070013 | intracellular organelle lumen | 79 | 1 | 1.91 | 0.8581 | 1 |
| GO:0005856 | cytoskeleton | 81 | 1 | 1.96 | 0.865 | 1 |
| GO:0005622 | intracellular | 1604 | 34 | 38.77 | 0.8678 | 1 |
| GO:0043229 | intracellular organelle | 1178 | 24 | 28.48 | 0.8713 | 1 |
| GO:0043226 | organelle | 1180 | 24 | 28.52 | 0.8736 | 1 |
| GO:0098796 | membrane protein complex | 202 | 3 | 4.88 | 0.875 | 1 |
| GO:0044428 | nuclear part | 90 | 1 | 2.18 | 0.8922 | 1 |
| GO:0044424 | intracellular part | 1539 | 31 | 37.2 | 0.9244 | 1 |
| GO:0043234 | protein complex | 481 | 6 | 11.63 | 0.9816 | 1 |
| GO:0043231 | intracellular membrane-bounded organelle | 634 | 8 | 15.33 | 0.9911 | 1 |
| GO:0044446 | intracellular organelle part | 404 | 4 | 9.77 | 0.9913 | 1 |
| GO:0044422 | organelle part | 406 | 4 | 9.81 | 0.9916 | 1 |
| GO:0043227 | membrane-bounded organelle | 643 | 8 | 15.54 | 0.9924 | 1 |
| GO:0005575 | cellular_component | 3930 | 95 | 95 | 1 | 1 |
| GO:0000123 | histone acetyltransferase complex | 5 | 0 | 0.12 | 1 | 1 |
| GO:0000139 | Golgi membrane | 10 | 0 | 0.24 | 1 | 1 |
| GO:0000148 | 1,3-beta-D-glucan synthase complex | 20 | 0 | 0.48 | 1 | 1 |
| GO:0000159 | protein phosphatase type 2A complex | 17 | 0 | 0.41 | 1 | 1 |
| GO:0000228 | nuclear chromosome | 9 | 0 | 0.22 | 1 | 1 |
| GO:0000276 | mitochondrial proton-transporting ATP sy... | 10 | 0 | 0.24 | 1 | 1 |
| GO:0000428 | DNA-directed RNA polymerase complex | 19 | 0 | 0.46 | 1 | 1 |
| GO:0000502 | proteasome complex | 52 | 0 | 1.26 | 1 | 1 |
| GO:0000775 | chromosome, centromeric region | 6 | 0 | 0.15 | 1 | 1 |
| GO:0000793 | condensed chromosome | 6 | 0 | 0.15 | 1 | 1 |
| GO:0005635 | nuclear envelope | 8 | 0 | 0.19 | 1 | 1 |
| GO:0005643 | nuclear pore | 8 | 0 | 0.19 | 1 | 1 |
| GO:0005666 | DNA-directed RNA polymerase III complex | 8 | 0 | 0.19 | 1 | 1 |
| GO:0005667 | transcription factor complex | 12 | 0 | 0.29 | 1 | 1 |
| GO:0005681 | spliceosomal complex | 5 | 0 | 0.12 | 1 | 1 |
| GO:0005730 | nucleolus | 7 | 0 | 0.17 | 1 | 1 |
| GO:0005739 | mitochondrion | 80 | 0 | 1.93 | 1 | 1 |
| GO:0005740 | mitochondrial envelope | 55 | 0 | 1.33 | 1 | 1 |
| GO:0005741 | mitochondrial outer membrane | 20 | 0 | 0.48 | 1 | 1 |
| GO:0005742 | mitochondrial outer membrane translocase... | 6 | 0 | 0.15 | 1 | 1 |
| GO:0005743 | mitochondrial inner membrane | 31 | 0 | 0.75 | 1 | 1 |
| GO:0005746 | mitochondrial respiratory chain | 11 | 0 | 0.27 | 1 | 1 |
| GO:0005750 | mitochondrial respiratory chain complex ... | 7 | 0 | 0.17 | 1 | 1 |
| GO:0005753 | mitochondrial proton-transporting ATP sy... | 13 | 0 | 0.31 | 1 | 1 |
| GO:0005759 | mitochondrial matrix | 12 | 0 | 0.29 | 1 | 1 |
| GO:0005768 | endosome | 7 | 0 | 0.17 | 1 | 1 |
| GO:0005773 | vacuole | 5 | 0 | 0.12 | 1 | 1 |
| GO:0005777 | peroxisome | 18 | 0 | 0.44 | 1 | 1 |
| GO:0005778 | peroxisomal membrane | 8 | 0 | 0.19 | 1 | 1 |
| GO:0005779 | integral component of peroxisomal membra... | 8 | 0 | 0.19 | 1 | 1 |
| GO:0005783 | endoplasmic reticulum | 70 | 0 | 1.69 | 1 | 1 |
| GO:0005789 | endoplasmic reticulum membrane | 44 | 0 | 1.06 | 1 | 1 |
| GO:0005794 | Golgi apparatus | 47 | 0 | 1.14 | 1 | 1 |
| GO:0005795 | Golgi stack | 5 | 0 | 0.12 | 1 | 1 |
| GO:0005798 | Golgi-associated vesicle | 28 | 0 | 0.68 | 1 | 1 |
| GO:0005839 | proteasome core complex | 46 | 0 | 1.11 | 1 | 1 |
| GO:0005849 | mRNA cleavage factor complex | 6 | 0 | 0.15 | 1 | 1 |
| GO:0005852 | eukaryotic translation initiation factor... | 18 | 0 | 0.44 | 1 | 1 |
| GO:0005875 | microtubule associated complex | 11 | 0 | 0.27 | 1 | 1 |
| GO:0005885 | Arp2/3 protein complex | 5 | 0 | 0.12 | 1 | 1 |
| GO:0005887 | integral component of plasma membrane | 14 | 0 | 0.34 | 1 | 1 |
| GO:0005890 | sodium:potassium-exchanging ATPase compl... | 5 | 0 | 0.12 | 1 | 1 |
| GO:0005905 | clathrin-coated pit | 8 | 0 | 0.19 | 1 | 1 |
| GO:0005956 | protein kinase CK2 complex | 8 | 0 | 0.19 | 1 | 1 |
| GO:0008250 | oligosaccharyltransferase complex | 10 | 0 | 0.24 | 1 | 1 |
| GO:0008287 | protein serine/threonine phosphatase com... | 18 | 0 | 0.44 | 1 | 1 |
| GO:0008290 | F-actin capping protein complex | 8 | 0 | 0.19 | 1 | 1 |
| GO:0009289 | pilus | 17 | 0 | 0.41 | 1 | 1 |
| GO:0009507 | chloroplast | 11 | 0 | 0.27 | 1 | 1 |
| GO:0009523 | photosystem II | 22 | 0 | 0.53 | 1 | 1 |
| GO:0009536 | plastid | 11 | 0 | 0.27 | 1 | 1 |
| GO:0009654 | photosystem II oxygen evolving complex | 15 | 0 | 0.36 | 1 | 1 |
| GO:0012505 | endomembrane system | 140 | 0 | 3.38 | 1 | 1 |
| GO:0012506 | vesicle membrane | 28 | 0 | 0.68 | 1 | 1 |
| GO:0012507 | ER to Golgi transport vesicle membrane | 15 | 0 | 0.36 | 1 | 1 |
| GO:0012510 | trans-Golgi network transport vesicle me... | 8 | 0 | 0.19 | 1 | 1 |
| GO:0015630 | microtubule cytoskeleton | 12 | 0 | 0.29 | 1 | 1 |
| GO:0015934 | large ribosomal subunit | 8 | 0 | 0.19 | 1 | 1 |
| GO:0016272 | prefoldin complex | 7 | 0 | 0.17 | 1 | 1 |
| GO:0016469 | proton-transporting two-sector ATPase co... | 55 | 0 | 1.33 | 1 | 1 |
| GO:0016591 | DNA-directed RNA polymerase II, holoenzy... | 11 | 0 | 0.27 | 1 | 1 |
| GO:0016592 | mediator complex | 13 | 0 | 0.31 | 1 | 1 |
| GO:0017119 | Golgi transport complex | 5 | 0 | 0.12 | 1 | 1 |
| GO:0019012 | virion | 5 | 0 | 0.12 | 1 | 1 |
| GO:0019028 | viral capsid | 5 | 0 | 0.12 | 1 | 1 |
| GO:0019773 | proteasome core complex, alpha-subunit c... | 15 | 0 | 0.36 | 1 | 1 |
| GO:0019866 | organelle inner membrane | 31 | 0 | 0.75 | 1 | 1 |
| GO:0019867 | outer membrane | 49 | 0 | 1.18 | 1 | 1 |
| GO:0019898 | extrinsic component of membrane | 9 | 0 | 0.22 | 1 | 1 |
| GO:0022624 | proteasome accessory complex | 5 | 0 | 0.12 | 1 | 1 |
| GO:0030117 | membrane coat | 54 | 0 | 1.31 | 1 | 1 |
| GO:0030118 | clathrin coat | 13 | 0 | 0.31 | 1 | 1 |
| GO:0030119 | AP-type membrane coat adaptor complex | 6 | 0 | 0.15 | 1 | 1 |
| GO:0030120 | vesicle coat | 28 | 0 | 0.68 | 1 | 1 |
| GO:0030125 | clathrin vesicle coat | 8 | 0 | 0.19 | 1 | 1 |
| GO:0030126 | COPI vesicle coat | 5 | 0 | 0.12 | 1 | 1 |
| GO:0030127 | COPII vesicle coat | 15 | 0 | 0.36 | 1 | 1 |
| GO:0030130 | clathrin coat of trans-Golgi network ves... | 8 | 0 | 0.19 | 1 | 1 |
| GO:0030131 | clathrin adaptor complex | 5 | 0 | 0.12 | 1 | 1 |
| GO:0030132 | clathrin coat of coated pit | 8 | 0 | 0.19 | 1 | 1 |
| GO:0030133 | transport vesicle | 23 | 0 | 0.56 | 1 | 1 |
| GO:0030134 | COPII-coated ER to Golgi transport vesic... | 15 | 0 | 0.36 | 1 | 1 |
| GO:0030135 | coated vesicle | 28 | 0 | 0.68 | 1 | 1 |
| GO:0030136 | clathrin-coated vesicle | 8 | 0 | 0.19 | 1 | 1 |
| GO:0030137 | COPI-coated vesicle | 5 | 0 | 0.12 | 1 | 1 |
| GO:0030140 | trans-Golgi network transport vesicle | 8 | 0 | 0.19 | 1 | 1 |
| GO:0030176 | integral component of endoplasmic reticu... | 12 | 0 | 0.29 | 1 | 1 |
| GO:0030286 | dynein complex | 8 | 0 | 0.19 | 1 | 1 |
| GO:0030288 | outer membrane-bounded periplasmic space | 11 | 0 | 0.27 | 1 | 1 |
| GO:0030313 | cell envelope | 17 | 0 | 0.41 | 1 | 1 |
| GO:0030658 | transport vesicle membrane | 23 | 0 | 0.56 | 1 | 1 |
| GO:0030659 | cytoplasmic vesicle membrane | 28 | 0 | 0.68 | 1 | 1 |
| GO:0030660 | Golgi-associated vesicle membrane | 28 | 0 | 0.68 | 1 | 1 |
| GO:0030662 | coated vesicle membrane | 28 | 0 | 0.68 | 1 | 1 |
| GO:0030663 | COPI-coated vesicle membrane | 5 | 0 | 0.12 | 1 | 1 |
| GO:0030665 | clathrin-coated vesicle membrane | 8 | 0 | 0.19 | 1 | 1 |
| GO:0030684 | preribosome | 8 | 0 | 0.19 | 1 | 1 |
| GO:0030904 | retromer complex | 6 | 0 | 0.15 | 1 | 1 |
| GO:0030906 | retromer, cargo-selective complex | 6 | 0 | 0.15 | 1 | 1 |
| GO:0031012 | extracellular matrix | 12 | 0 | 0.29 | 1 | 1 |
| GO:0031090 | organelle membrane | 107 | 0 | 2.59 | 1 | 1 |
| GO:0031227 | intrinsic component of endoplasmic retic... | 12 | 0 | 0.29 | 1 | 1 |
| GO:0031231 | intrinsic component of peroxisomal membr... | 8 | 0 | 0.19 | 1 | 1 |
| GO:0031248 | protein acetyltransferase complex | 6 | 0 | 0.15 | 1 | 1 |
| GO:0031300 | intrinsic component of organelle membran... | 11 | 0 | 0.27 | 1 | 1 |
| GO:0031301 | integral component of organelle membrane | 11 | 0 | 0.27 | 1 | 1 |
| GO:0031410 | cytoplasmic vesicle | 37 | 0 | 0.89 | 1 | 1 |
| GO:0031903 | microbody membrane | 8 | 0 | 0.19 | 1 | 1 |
| GO:0031966 | mitochondrial membrane | 51 | 0 | 1.23 | 1 | 1 |
| GO:0031967 | organelle envelope | 63 | 0 | 1.52 | 1 | 1 |
| GO:0031968 | organelle outer membrane | 20 | 0 | 0.48 | 1 | 1 |
| GO:0031975 | envelope | 80 | 0 | 1.93 | 1 | 1 |
| GO:0031982 | vesicle | 37 | 0 | 0.89 | 1 | 1 |
| GO:0031984 | organelle subcompartment | 57 | 0 | 1.38 | 1 | 1 |
| GO:0032040 | small-subunit processome | 5 | 0 | 0.12 | 1 | 1 |
| GO:0033176 | proton-transporting V-type ATPase comple... | 13 | 0 | 0.31 | 1 | 1 |
| GO:0033177 | proton-transporting two-sector ATPase co... | 34 | 0 | 0.82 | 1 | 1 |
| GO:0033178 | proton-transporting two-sector ATPase co... | 18 | 0 | 0.44 | 1 | 1 |
| GO:0033180 | proton-transporting V-type ATPase, V1 do... | 6 | 0 | 0.15 | 1 | 1 |
| GO:0042175 | nuclear outer membrane-endoplasmic retic... | 44 | 0 | 1.06 | 1 | 1 |
| GO:0042579 | microbody | 18 | 0 | 0.44 | 1 | 1 |
| GO:0042597 | periplasmic space | 24 | 0 | 0.58 | 1 | 1 |
| GO:0042651 | thylakoid membrane | 16 | 0 | 0.39 | 1 | 1 |
| GO:0042765 | GPI-anchor transamidase complex | 8 | 0 | 0.19 | 1 | 1 |
| GO:0042995 | cell projection | 19 | 0 | 0.46 | 1 | 1 |
| GO:0043190 | ATP-binding cassette (ABC) transporter c... | 13 | 0 | 0.31 | 1 | 1 |
| GO:0044391 | ribosomal subunit | 8 | 0 | 0.19 | 1 | 1 |
| GO:0044421 | extracellular region part | 15 | 0 | 0.36 | 1 | 1 |
| GO:0044423 | virion part | 5 | 0 | 0.12 | 1 | 1 |
| GO:0044429 | mitochondrial part | 67 | 0 | 1.62 | 1 | 1 |
| GO:0044431 | Golgi apparatus part | 46 | 0 | 1.11 | 1 | 1 |
| GO:0044432 | endoplasmic reticulum part | 45 | 0 | 1.09 | 1 | 1 |
| GO:0044433 | cytoplasmic vesicle part | 35 | 0 | 0.85 | 1 | 1 |
| GO:0044438 | microbody part | 8 | 0 | 0.19 | 1 | 1 |
| GO:0044439 | peroxisomal part | 8 | 0 | 0.19 | 1 | 1 |
| GO:0044440 | endosomal part | 7 | 0 | 0.17 | 1 | 1 |
| GO:0044454 | nuclear chromosome part | 8 | 0 | 0.19 | 1 | 1 |
| GO:0044455 | mitochondrial membrane part | 35 | 0 | 0.85 | 1 | 1 |
| GO:0044798 | nuclear transcription factor complex | 7 | 0 | 0.17 | 1 | 1 |
| GO:0045259 | proton-transporting ATP synthase complex | 24 | 0 | 0.58 | 1 | 1 |
| GO:0045261 | proton-transporting ATP synthase complex... | 10 | 0 | 0.24 | 1 | 1 |
| GO:0045263 | proton-transporting ATP synthase complex... | 14 | 0 | 0.34 | 1 | 1 |
| GO:0045275 | respiratory chain complex III | 7 | 0 | 0.17 | 1 | 1 |
| GO:0048475 | coated membrane | 54 | 0 | 1.31 | 1 | 1 |
| GO:0048500 | signal recognition particle | 6 | 0 | 0.15 | 1 | 1 |
| GO:0055029 | nuclear DNA-directed RNA polymerase comp... | 19 | 0 | 0.46 | 1 | 1 |
| GO:0070069 | cytochrome complex | 11 | 0 | 0.27 | 1 | 1 |
| GO:0070469 | respiratory chain | 11 | 0 | 0.27 | 1 | 1 |
| GO:0090533 | cation-transporting ATPase complex | 5 | 0 | 0.12 | 1 | 1 |
| GO:0090575 | RNA polymerase II transcription factor c... | 7 | 0 | 0.17 | 1 | 1 |
| GO:0097708 | intracellular vesicle | 37 | 0 | 0.89 | 1 | 1 |
| GO:0098533 | ATPase dependent transmembrane transport... | 18 | 0 | 0.44 | 1 | 1 |
| GO:0098588 | bounding membrane of organelle | 76 | 0 | 1.84 | 1 | 1 |
| GO:0098589 | membrane region | 8 | 0 | 0.19 | 1 | 1 |
| GO:0098687 | chromosomal region | 7 | 0 | 0.17 | 1 | 1 |
| GO:0098791 | Golgi subcompartment | 13 | 0 | 0.31 | 1 | 1 |
| GO:0098797 | plasma membrane protein complex | 24 | 0 | 0.58 | 1 | 1 |
| GO:0098798 | mitochondrial protein complex | 34 | 0 | 0.82 | 1 | 1 |
| GO:0098799 | outer mitochondrial membrane protein com... | 7 | 0 | 0.17 | 1 | 1 |
| GO:0098800 | inner mitochondrial membrane protein com... | 27 | 0 | 0.65 | 1 | 1 |
| GO:0098803 | respiratory chain complex | 10 | 0 | 0.24 | 1 | 1 |
| GO:0098805 | whole membrane | 64 | 0 | 1.55 | 1 | 1 |
| GO:0098827 | endoplasmic reticulum subcompartment | 44 | 0 | 1.06 | 1 | 1 |
| GO:1902493 | acetyltransferase complex | 6 | 0 | 0.15 | 1 | 1 |
| GO:1902495 | transmembrane transporter complex | 25 | 0 | 0.6 | 1 | 1 |
| GO:1903293 | phosphatase complex | 18 | 0 | 0.44 | 1 | 1 |
| GO:1904949 | ATPase complex | 20 | 0 | 0.48 | 1 | 1 |
| GO:1905368 | peptidase complex | 55 | 0 | 1.33 | 1 | 1 |
| GO:1905369 | endopeptidase complex | 52 | 0 | 1.26 | 1 | 1 |
| GO:1990204 | oxidoreductase complex | 24 | 0 | 0.58 | 1 | 1 |
| GO:1990351 | transporter complex | 25 | 0 | 0.6 | 1 | 1 |

**3-MF**

| GO ID | Term | Annotated nuigene number | Significant unigene number | Expected | Classic Fisher | FDR |
| --- | --- | --- | --- | --- | --- | --- |
| GO:0004523 | RNA-DNA hybrid ribonuclease activity | 91 | 13 | 3.04 | 9.80E-06 | 0.003235 |
| GO:0004568 | chitinase activity | 27 | 7 | 0.9 | 2.20E-05 | 0.003235 |
| GO:0004521 | endoribonuclease activity | 113 | 14 | 3.78 | 2.40E-05 | 0.003235 |
| GO:0016891 | endoribonuclease activity, producing 5'-... | 99 | 13 | 3.31 | 2.50E-05 | 0.003235 |
| GO:0016893 | endonuclease activity, active with eithe... | 99 | 13 | 3.31 | 2.50E-05 | 0.003235 |
| GO:0004540 | ribonuclease activity | 124 | 14 | 4.14 | 6.80E-05 | 0.006839714 |
| GO:0004519 | endonuclease activity | 125 | 14 | 4.18 | 7.40E-05 | 0.006839714 |
| GO:0016798 | hydrolase activity, acting on glycosyl b... | 440 | 30 | 14.7 | 0.00018 | 0.0145575 |
| GO:0004518 | nuclease activity | 170 | 15 | 5.68 | 0.00059 | 0.042414444 |
| GO:0004553 | hydrolase activity, hydrolyzing O-glycos... | 399 | 26 | 13.33 | 0.00093 | 0.060171 |
| GO:0043531 | ADP binding | 362 | 24 | 12.1 | 0.00115 | 0.067640909 |
| GO:0003854 | 3-beta-hydroxy-delta5-steroid dehydrogen... | 10 | 3 | 0.33 | 0.00373 | 0.16822 |
| GO:0016229 | steroid dehydrogenase activity | 10 | 3 | 0.33 | 0.00373 | 0.16822 |
| GO:0033764 | steroid dehydrogenase activity, acting o... | 10 | 3 | 0.33 | 0.00373 | 0.16822 |
| GO:0008061 | chitin binding | 32 | 5 | 1.07 | 0.0039 | 0.16822 |
| GO:0016717 | oxidoreductase activity, acting on paire... | 24 | 4 | 0.8 | 0.00769 | 0.310964375 |
| GO:0016788 | hydrolase activity, acting on ester bond... | 402 | 23 | 13.43 | 0.00874 | 0.332634118 |
| GO:0051087 | chaperone binding | 14 | 3 | 0.47 | 0.01025 | 0.337087 |
| GO:0004489 | methylenetetrahydrofolate reductase (NAD... | 5 | 2 | 0.17 | 0.01042 | 0.337087 |
| GO:0004819 | glutamine-tRNA ligase activity | 5 | 2 | 0.17 | 0.01042 | 0.337087 |
| GO:0016614 | oxidoreductase activity, acting on CH-OH... | 152 | 11 | 5.08 | 0.01313 | 0.396901034 |
| GO:0099516 | ion antiporter activity | 6 | 2 | 0.2 | 0.01529 | 0.396901034 |
| GO:0004672 | protein kinase activity | 1103 | 50 | 36.86 | 0.01668 | 0.396901034 |
| GO:0046872 | metal ion binding | 1393 | 61 | 46.55 | 0.01688 | 0.396901034 |
| GO:0140098 | catalytic activity, acting on RNA | 380 | 21 | 12.7 | 0.01696 | 0.396901034 |
| GO:0003684 | damaged DNA binding | 17 | 3 | 0.57 | 0.01779 | 0.396901034 |
| GO:0004743 | pyruvate kinase activity | 17 | 3 | 0.57 | 0.01779 | 0.396901034 |
| GO:0030955 | potassium ion binding | 17 | 3 | 0.57 | 0.01779 | 0.396901034 |
| GO:0031420 | alkali metal ion binding | 17 | 3 | 0.57 | 0.01779 | 0.396901034 |
| GO:0043169 | cation binding | 1409 | 61 | 47.08 | 0.0209 | 0.423178438 |
| GO:0030597 | RNA glycosylase activity | 7 | 2 | 0.23 | 0.02093 | 0.423178438 |
| GO:0030598 | rRNA N-glycosylase activity | 7 | 2 | 0.23 | 0.02093 | 0.423178438 |
| GO:0016773 | phosphotransferase activity, alcohol gro... | 1256 | 55 | 41.97 | 0.0228 | 0.447018182 |
| GO:0016616 | oxidoreductase activity, acting on the C... | 126 | 9 | 4.21 | 0.02543 | 0.478040571 |
| GO:0004674 | protein serine/threonine kinase activity | 34 | 4 | 1.14 | 0.02586 | 0.478040571 |
| GO:0016799 | hydrolase activity, hydrolyzing N-glycos... | 20 | 3 | 0.67 | 0.0277 | 0.497830556 |
| GO:0016705 | oxidoreductase activity, acting on paire... | 334 | 18 | 11.16 | 0.03198 | 0.559217838 |
| GO:0043167 | ion binding | 4093 | 155 | 136.77 | 0.03544 | 0.603412632 |
| GO:0016301 | kinase activity | 1216 | 52 | 40.63 | 0.03849 | 0.638539231 |
| GO:0046914 | transition metal ion binding | 901 | 40 | 30.11 | 0.04024 | 0.650882 |
| GO:0004857 | enzyme inhibitor activity | 117 | 8 | 3.91 | 0.04237 | 0.653161905 |
| GO:0046983 | protein dimerization activity | 160 | 10 | 5.35 | 0.0424 | 0.653161905 |
| GO:0008138 | protein tyrosine/serine/threonine phosph... | 11 | 2 | 0.37 | 0.0502 | 0.738168182 |
| GO:0140102 | catalytic activity, acting on a rRNA | 11 | 2 | 0.37 | 0.0502 | 0.738168182 |
| GO:0016846 | carbon-sulfur lyase activity | 12 | 2 | 0.4 | 0.05894 | 0.847426222 |
| GO:0016702 | oxidoreductase activity, acting on singl... | 46 | 4 | 1.54 | 0.06683 | 0.919978936 |
| GO:0051213 | dioxygenase activity | 46 | 4 | 1.54 | 0.06683 | 0.919978936 |
| GO:0005488 | binding | 8045 | 285 | 268.83 | 0.07193 | 0.969556458 |
| GO:0005516 | calmodulin binding | 30 | 3 | 1 | 0.0774 | 0.981486792 |
| GO:0016646 | oxidoreductase activity, acting on the C... | 14 | 2 | 0.47 | 0.0778 | 0.981486792 |
| GO:0016787 | hydrolase activity | 2254 | 87 | 75.32 | 0.07879 | 0.981486792 |
| GO:0016759 | cellulose synthase activity | 49 | 4 | 1.64 | 0.0804 | 0.981486792 |
| GO:0016760 | cellulose synthase (UDP-forming) activit... | 49 | 4 | 1.64 | 0.0804 | 0.981486792 |
| GO:0043565 | sequence-specific DNA binding | 158 | 9 | 5.28 | 0.08323 | 0.997218704 |
| GO:0008270 | zinc ion binding | 458 | 21 | 15.3 | 0.08923 | 1 |
| GO:0019239 | deaminase activity | 16 | 2 | 0.53 | 0.09825 | 1 |
| GO:0020037 | heme binding | 389 | 18 | 13 | 0.10274 | 1 |
| GO:0046906 | tetrapyrrole binding | 392 | 18 | 13.1 | 0.10827 | 1 |
| GO:0032559 | adenyl ribonucleotide binding | 2104 | 80 | 70.31 | 0.11499 | 1 |
| GO:0030554 | adenyl nucleotide binding | 2107 | 80 | 70.41 | 0.11761 | 1 |
| GO:0003824 | catalytic activity | 7625 | 268 | 254.8 | 0.11945 | 1 |
| GO:0016881 | acid-amino acid ligase activity | 18 | 2 | 0.6 | 0.12 | 1 |
| GO:0016701 | oxidoreductase activity, acting on singl... | 59 | 4 | 1.97 | 0.13425 | 1 |
| GO:0004866 | endopeptidase inhibitor activity | 60 | 4 | 2 | 0.14029 | 1 |
| GO:0030414 | peptidase inhibitor activity | 60 | 4 | 2 | 0.14029 | 1 |
| GO:0061134 | peptidase regulator activity | 60 | 4 | 2 | 0.14029 | 1 |
| GO:0061135 | endopeptidase regulator activity | 60 | 4 | 2 | 0.14029 | 1 |
| GO:0097159 | organic cyclic compound binding | 4924 | 176 | 164.54 | 0.14243 | 1 |
| GO:1901363 | heterocyclic compound binding | 4924 | 176 | 164.54 | 0.14243 | 1 |
| GO:0004721 | phosphoprotein phosphatase activity | 20 | 2 | 0.67 | 0.14279 | 1 |
| GO:0008168 | methyltransferase activity | 254 | 12 | 8.49 | 0.14472 | 1 |
| GO:0008171 | O-methyltransferase activity | 21 | 2 | 0.7 | 0.1545 | 1 |
| GO:0016772 | transferase activity, transferring phosp... | 1491 | 57 | 49.82 | 0.15486 | 1 |
| GO:0003906 | DNA-(apurinic or apyrimidinic site) lyas... | 5 | 1 | 0.17 | 0.1563 | 1 |
| GO:0004143 | diacylglycerol kinase activity | 5 | 1 | 0.17 | 0.1563 | 1 |
| GO:0004418 | hydroxymethylbilane synthase activity | 5 | 1 | 0.17 | 0.1563 | 1 |
| GO:0015098 | molybdate ion transmembrane transporter ... | 5 | 1 | 0.17 | 0.1563 | 1 |
| GO:0017025 | TBP-class protein binding | 5 | 1 | 0.17 | 0.1563 | 1 |
| GO:0019900 | kinase binding | 5 | 1 | 0.17 | 0.1563 | 1 |
| GO:0019901 | protein kinase binding | 5 | 1 | 0.17 | 0.1563 | 1 |
| GO:0070403 | NAD+ binding | 5 | 1 | 0.17 | 0.1563 | 1 |
| GO:0016747 | transferase activity, transferring acyl ... | 182 | 9 | 6.08 | 0.15652 | 1 |
| GO:0016627 | oxidoreductase activity, acting on the C... | 63 | 4 | 2.11 | 0.15904 | 1 |
| GO:0016740 | transferase activity | 2669 | 98 | 89.19 | 0.16065 | 1 |
| GO:1901505 | carbohydrate derivative transmembrane tr... | 22 | 2 | 0.74 | 0.16639 | 1 |
| GO:0030234 | enzyme regulator activity | 186 | 9 | 6.22 | 0.1711 | 1 |
| GO:0001871 | pattern binding | 23 | 2 | 0.77 | 0.17842 | 1 |
| GO:0015932 | nucleobase-containing compound transmemb... | 23 | 2 | 0.77 | 0.17842 | 1 |
| GO:0016645 | oxidoreductase activity, acting on the C... | 23 | 2 | 0.77 | 0.17842 | 1 |
| GO:0030247 | polysaccharide binding | 23 | 2 | 0.77 | 0.17842 | 1 |
| GO:0004592 | pantoate-beta-alanine ligase activity | 6 | 1 | 0.2 | 0.18451 | 1 |
| GO:0033743 | peptide-methionine (R)-S-oxide reductase... | 6 | 1 | 0.2 | 0.18451 | 1 |
| GO:0050664 | oxidoreductase activity, acting on NAD(P... | 6 | 1 | 0.2 | 0.18451 | 1 |
| GO:0097367 | carbohydrate derivative binding | 2461 | 90 | 82.24 | 0.18489 | 1 |
| GO:0005506 | iron ion binding | 323 | 14 | 10.79 | 0.19431 | 1 |
| GO:0005515 | protein binding | 2183 | 80 | 72.95 | 0.19741 | 1 |
| GO:0005507 | copper ion binding | 94 | 5 | 3.14 | 0.20587 | 1 |
| GO:0016628 | oxidoreductase activity, acting on the C... | 7 | 1 | 0.23 | 0.21177 | 1 |
| GO:0016741 | transferase activity, transferring one-c... | 277 | 12 | 9.26 | 0.2177 | 1 |
| GO:0046527 | glucosyltransferase activity | 96 | 5 | 3.21 | 0.21787 | 1 |
| GO:0016758 | transferase activity, transferring hexos... | 360 | 15 | 12.03 | 0.22528 | 1 |
| GO:0004000 | adenosine deaminase activity | 8 | 1 | 0.27 | 0.23812 | 1 |
| GO:0004476 | mannose-6-phosphate isomerase activity | 8 | 1 | 0.27 | 0.23812 | 1 |
| GO:0004843 | thiol-dependent ubiquitin-specific prote... | 8 | 1 | 0.27 | 0.23812 | 1 |
| GO:0035251 | UDP-glucosyltransferase activity | 77 | 4 | 2.57 | 0.25627 | 1 |
| GO:0004045 | aminoacyl-tRNA hydrolase activity | 9 | 1 | 0.3 | 0.26359 | 1 |
| GO:0004555 | alpha,alpha-trehalase activity | 9 | 1 | 0.3 | 0.26359 | 1 |
| GO:0004571 | mannosyl-oligosaccharide 1,2-alpha-manno... | 9 | 1 | 0.3 | 0.26359 | 1 |
| GO:0015924 | mannosyl-oligosaccharide mannosidase act... | 9 | 1 | 0.3 | 0.26359 | 1 |
| GO:0015927 | trehalase activity | 9 | 1 | 0.3 | 0.26359 | 1 |
| GO:0015079 | potassium ion transmembrane transporter ... | 30 | 2 | 1 | 0.26513 | 1 |
| GO:0032555 | purine ribonucleotide binding | 2382 | 85 | 79.6 | 0.26775 | 1 |
| GO:0004601 | peroxidase activity | 104 | 5 | 3.48 | 0.2678 | 1 |
| GO:0017076 | purine nucleotide binding | 2396 | 85 | 80.07 | 0.28746 | 1 |
| GO:0008373 | sialyltransferase activity | 10 | 1 | 0.33 | 0.28822 | 1 |
| GO:0031625 | ubiquitin protein ligase binding | 10 | 1 | 0.33 | 0.28822 | 1 |
| GO:0033897 | ribonuclease T2 activity | 10 | 1 | 0.33 | 0.28822 | 1 |
| GO:0044389 | ubiquitin-like protein ligase binding | 10 | 1 | 0.33 | 0.28822 | 1 |
| GO:0045300 | acyl-[acyl-carrier-protein] desaturase a... | 10 | 1 | 0.33 | 0.28822 | 1 |
| GO:0016491 | oxidoreductase activity | 1582 | 57 | 52.86 | 0.29061 | 1 |
| GO:0032553 | ribonucleotide binding | 2407 | 85 | 80.43 | 0.30337 | 1 |
| GO:0098772 | molecular function regulator | 245 | 10 | 8.19 | 0.30412 | 1 |
| GO:0009678 | hydrogen-translocating pyrophosphatase a... | 11 | 1 | 0.37 | 0.31202 | 1 |
| GO:0016892 | endoribonuclease activity, producing 3'-... | 11 | 1 | 0.37 | 0.31202 | 1 |
| GO:0016894 | endonuclease activity, active with eithe... | 11 | 1 | 0.37 | 0.31202 | 1 |
| GO:0016684 | oxidoreductase activity, acting on perox... | 111 | 5 | 3.71 | 0.31333 | 1 |
| GO:0016831 | carboxy-lyase activity | 61 | 3 | 2.04 | 0.33387 | 1 |
| GO:0030246 | carbohydrate binding | 61 | 3 | 2.04 | 0.33387 | 1 |
| GO:0000062 | fatty-acyl-CoA binding | 12 | 1 | 0.4 | 0.33503 | 1 |
| GO:1901567 | fatty acid derivative binding | 12 | 1 | 0.4 | 0.33503 | 1 |
| GO:0000166 | nucleotide binding | 2665 | 93 | 89.05 | 0.33693 | 1 |
| GO:1901265 | nucleoside phosphate binding | 2665 | 93 | 89.05 | 0.33693 | 1 |
| GO:0003735 | structural constituent of ribosome | 394 | 15 | 13.17 | 0.33916 | 1 |
| GO:0008514 | organic anion transmembrane transporter ... | 36 | 2 | 1.2 | 0.33976 | 1 |
| GO:0016746 | transferase activity, transferring acyl ... | 254 | 10 | 8.49 | 0.34364 | 1 |
| GO:0043168 | anion binding | 2762 | 96 | 92.3 | 0.34945 | 1 |
| GO:0016671 | oxidoreductase activity, acting on a sul... | 13 | 1 | 0.43 | 0.35727 | 1 |
| GO:0071949 | FAD binding | 13 | 1 | 0.43 | 0.35727 | 1 |
| GO:0036094 | small molecule binding | 2830 | 98 | 94.57 | 0.36256 | 1 |
| GO:0003777 | microtubule motor activity | 67 | 3 | 2.24 | 0.3889 | 1 |
| GO:0016209 | antioxidant activity | 123 | 5 | 4.11 | 0.3931 | 1 |
| GO:0030170 | pyridoxal phosphate binding | 123 | 5 | 4.11 | 0.3931 | 1 |
| GO:0070279 | vitamin B6 binding | 123 | 5 | 4.11 | 0.3931 | 1 |
| GO:0016814 | hydrolase activity, acting on carbon-nit... | 15 | 1 | 0.5 | 0.39954 | 1 |
| GO:0140096 | catalytic activity, acting on a protein | 1737 | 60 | 58.04 | 0.41167 | 1 |
| GO:0015297 | antiporter activity | 98 | 4 | 3.27 | 0.4153 | 1 |
| GO:0004332 | fructose-bisphosphate aldolase activity | 16 | 1 | 0.53 | 0.41963 | 1 |
| GO:0004345 | glucose-6-phosphate dehydrogenase activi... | 16 | 1 | 0.53 | 0.41963 | 1 |
| GO:0005096 | GTPase activator activity | 16 | 1 | 0.53 | 0.41963 | 1 |
| GO:0033926 | glycopeptide alpha-N-acetylgalactosamini... | 16 | 1 | 0.53 | 0.41963 | 1 |
| GO:0034061 | DNA polymerase activity | 16 | 1 | 0.53 | 0.41963 | 1 |
| GO:0019899 | enzyme binding | 71 | 3 | 2.37 | 0.42498 | 1 |
| GO:0015399 | primary active transmembrane transporter... | 100 | 4 | 3.34 | 0.43035 | 1 |
| GO:0015405 | P-P-bond-hydrolysis-driven transmembrane... | 100 | 4 | 3.34 | 0.43035 | 1 |
| GO:0016762 | xyloglucan:xyloglucosyl transferase acti... | 18 | 1 | 0.6 | 0.45781 | 1 |
| GO:0008194 | UDP-glycosyltransferase activity | 163 | 6 | 5.45 | 0.46367 | 1 |
| GO:0016757 | transferase activity, transferring glyco... | 462 | 16 | 15.44 | 0.47815 | 1 |
| GO:0016830 | carbon-carbon lyase activity | 107 | 4 | 3.58 | 0.4821 | 1 |
| GO:0005198 | structural molecule activity | 434 | 15 | 14.5 | 0.48434 | 1 |
| GO:0000287 | magnesium ion binding | 78 | 3 | 2.61 | 0.48617 | 1 |
| GO:0017176 | phosphatidylinositol N-acetylglucosaminy... | 20 | 1 | 0.67 | 0.49349 | 1 |
| GO:0003774 | motor activity | 109 | 4 | 3.64 | 0.49655 | 1 |
| GO:0008509 | anion transmembrane transporter activity | 80 | 3 | 2.67 | 0.50308 | 1 |
| GO:0003968 | RNA-directed 5'-3' RNA polymerase activi... | 21 | 1 | 0.7 | 0.51044 | 1 |
| GO:0004427 | inorganic diphosphatase activity | 21 | 1 | 0.7 | 0.51044 | 1 |
| GO:0008017 | microtubule binding | 83 | 3 | 2.77 | 0.5279 | 1 |
| GO:0016307 | phosphatidylinositol phosphate kinase ac... | 23 | 1 | 0.77 | 0.54265 | 1 |
| GO:0016791 | phosphatase activity | 54 | 2 | 1.8 | 0.54299 | 1 |
| GO:0005509 | calcium ion binding | 208 | 7 | 6.95 | 0.54635 | 1 |
| GO:0042578 | phosphoric ester hydrolase activity | 86 | 3 | 2.87 | 0.55201 | 1 |
| GO:0050660 | flavin adenine dinucleotide binding | 117 | 4 | 3.91 | 0.55251 | 1 |
| GO:0008081 | phosphoric diester hydrolase activity | 24 | 1 | 0.8 | 0.55796 | 1 |
| GO:0008134 | transcription factor binding | 24 | 1 | 0.8 | 0.55796 | 1 |
| GO:0019843 | rRNA binding | 25 | 1 | 0.84 | 0.57276 | 1 |
| GO:0016820 | hydrolase activity, acting on acid anhyd... | 89 | 3 | 2.97 | 0.57538 | 1 |
| GO:0042626 | ATPase activity, coupled to transmembran... | 89 | 3 | 2.97 | 0.57538 | 1 |
| GO:0043492 | ATPase activity, coupled to movement of ... | 89 | 3 | 2.97 | 0.57538 | 1 |
| GO:0019842 | vitamin binding | 153 | 5 | 5.11 | 0.5833 | 1 |
| GO:0015291 | secondary active transmembrane transport... | 122 | 4 | 4.08 | 0.58576 | 1 |
| GO:0022804 | active transmembrane transporter activit... | 247 | 8 | 8.25 | 0.5862 | 1 |
| GO:0008047 | enzyme activator activity | 26 | 1 | 0.87 | 0.58706 | 1 |
| GO:0016832 | aldehyde-lyase activity | 26 | 1 | 0.87 | 0.58706 | 1 |
| GO:0140103 | catalytic activity, acting on a glycopro... | 26 | 1 | 0.87 | 0.58706 | 1 |
| GO:0016887 | ATPase activity | 310 | 10 | 10.36 | 0.59065 | 1 |
| GO:0016667 | oxidoreductase activity, acting on a sul... | 59 | 2 | 1.97 | 0.59131 | 1 |
| GO:0015631 | tubulin binding | 92 | 3 | 3.07 | 0.59798 | 1 |
| GO:0003676 | nucleic acid binding | 1898 | 62 | 63.42 | 0.59829 | 1 |
| GO:0004559 | alpha-mannosidase activity | 28 | 1 | 0.94 | 0.61425 | 1 |
| GO:0015923 | mannosidase activity | 28 | 1 | 0.94 | 0.61425 | 1 |
| GO:0030695 | GTPase regulator activity | 28 | 1 | 0.94 | 0.61425 | 1 |
| GO:0052689 | carboxylic ester hydrolase activity | 63 | 2 | 2.11 | 0.62715 | 1 |
| GO:0009055 | electron transfer activity | 129 | 4 | 4.31 | 0.62981 | 1 |
| GO:0008375 | acetylglucosaminyltransferase activity | 65 | 2 | 2.17 | 0.64414 | 1 |
| GO:0005524 | ATP binding | 1742 | 56 | 58.21 | 0.64467 | 1 |
| GO:0016861 | intramolecular oxidoreductase activity, ... | 31 | 1 | 1.04 | 0.65171 | 1 |
| GO:0003677 | DNA binding | 920 | 29 | 30.74 | 0.65682 | 1 |
| GO:0003924 | GTPase activity | 199 | 6 | 6.65 | 0.6579 | 1 |
| GO:0016879 | ligase activity, forming carbon-nitrogen... | 67 | 2 | 2.24 | 0.66051 | 1 |
| GO:0016462 | pyrophosphatase activity | 676 | 21 | 22.59 | 0.66752 | 1 |
| GO:0050662 | coenzyme binding | 330 | 10 | 11.03 | 0.66871 | 1 |
| GO:0003700 | DNA binding transcription factor activit... | 395 | 12 | 13.2 | 0.67344 | 1 |
| GO:0016651 | oxidoreductase activity, acting on NAD(P... | 33 | 1 | 1.1 | 0.67465 | 1 |
| GO:0017111 | nucleoside-triphosphatase activity | 648 | 20 | 21.65 | 0.67611 | 1 |
| GO:0008237 | metallopeptidase activity | 70 | 2 | 2.34 | 0.68392 | 1 |
| GO:0016818 | hydrolase activity, acting on acid anhyd... | 683 | 21 | 22.82 | 0.68562 | 1 |
| GO:0016817 | hydrolase activity, acting on acid anhyd... | 687 | 21 | 22.96 | 0.69572 | 1 |
| GO:0016860 | intramolecular oxidoreductase activity | 35 | 1 | 1.17 | 0.69608 | 1 |
| GO:0004185 | serine-type carboxypeptidase activity | 73 | 2 | 2.44 | 0.706 | 1 |
| GO:0048037 | cofactor binding | 503 | 15 | 16.81 | 0.71166 | 1 |
| GO:0004842 | ubiquitin-protein transferase activity | 75 | 2 | 2.51 | 0.71999 | 1 |
| GO:0019787 | ubiquitin-like protein transferase activ... | 75 | 2 | 2.51 | 0.71999 | 1 |
| GO:0015298 | solute:cation antiporter activity | 38 | 1 | 1.27 | 0.72561 | 1 |
| GO:0015299 | solute:proton antiporter activity | 38 | 1 | 1.27 | 0.72561 | 1 |
| GO:0004812 | aminoacyl-tRNA ligase activity | 113 | 3 | 3.78 | 0.73326 | 1 |
| GO:0140101 | catalytic activity, acting on a tRNA | 148 | 4 | 4.95 | 0.73342 | 1 |
| GO:0005088 | Ras guanyl-nucleotide exchange factor ac... | 39 | 1 | 1.3 | 0.7348 | 1 |
| GO:0005089 | Rho guanyl-nucleotide exchange factor ac... | 39 | 1 | 1.3 | 0.7348 | 1 |
| GO:0017048 | Rho GTPase binding | 39 | 1 | 1.3 | 0.7348 | 1 |
| GO:0060589 | nucleoside-triphosphatase regulator acti... | 39 | 1 | 1.3 | 0.7348 | 1 |
| GO:0004180 | carboxypeptidase activity | 78 | 2 | 2.61 | 0.73992 | 1 |
| GO:0140110 | transcription regulator activity | 418 | 12 | 13.97 | 0.74524 | 1 |
| GO:0070008 | serine-type exopeptidase activity | 79 | 2 | 2.64 | 0.7463 | 1 |
| GO:0016829 | lyase activity | 256 | 7 | 8.55 | 0.75653 | 1 |
| GO:0030599 | pectinesterase activity | 42 | 1 | 1.4 | 0.76058 | 1 |
| GO:0015035 | protein disulfide oxidoreductase activit... | 43 | 1 | 1.44 | 0.7686 | 1 |
| GO:0015036 | disulfide oxidoreductase activity | 43 | 1 | 1.44 | 0.7686 | 1 |
| GO:0042623 | ATPase activity, coupled | 121 | 3 | 4.04 | 0.77437 | 1 |
| GO:0051287 | NAD binding | 84 | 2 | 2.81 | 0.77617 | 1 |
| GO:0015103 | inorganic anion transmembrane transporte... | 44 | 1 | 1.47 | 0.77636 | 1 |
| GO:0005085 | guanyl-nucleotide exchange factor activi... | 45 | 1 | 1.5 | 0.78386 | 1 |
| GO:0015267 | channel activity | 125 | 3 | 4.18 | 0.79289 | 1 |
| GO:0022803 | passive transmembrane transporter activi... | 125 | 3 | 4.18 | 0.79289 | 1 |
| GO:0016810 | hydrolase activity, acting on carbon-nit... | 48 | 1 | 1.6 | 0.80487 | 1 |
| GO:0019783 | ubiquitin-like protein-specific protease... | 48 | 1 | 1.6 | 0.80487 | 1 |
| GO:0036459 | thiol-dependent ubiquitinyl hydrolase ac... | 48 | 1 | 1.6 | 0.80487 | 1 |
| GO:0101005 | ubiquitinyl hydrolase activity | 48 | 1 | 1.6 | 0.80487 | 1 |
| GO:1901681 | sulfur compound binding | 48 | 1 | 1.6 | 0.80487 | 1 |
| GO:0017016 | Ras GTPase binding | 50 | 1 | 1.67 | 0.81774 | 1 |
| GO:0031267 | small GTPase binding | 50 | 1 | 1.67 | 0.81774 | 1 |
| GO:0035639 | purine ribonucleoside triphosphate bindi... | 2017 | 61 | 67.4 | 0.82111 | 1 |
| GO:0016765 | transferase activity, transferring alkyl... | 52 | 1 | 1.74 | 0.82976 | 1 |
| GO:0046873 | metal ion transmembrane transporter acti... | 97 | 2 | 3.24 | 0.83986 | 1 |
| GO:0015238 | drug transmembrane transporter activity | 56 | 1 | 1.87 | 0.85147 | 1 |
| GO:0051020 | GTPase binding | 56 | 1 | 1.87 | 0.85147 | 1 |
| GO:0090484 | drug transporter activity | 56 | 1 | 1.87 | 0.85147 | 1 |
| GO:0016874 | ligase activity | 101 | 2 | 3.38 | 0.85587 | 1 |
| GO:0140097 | catalytic activity, acting on DNA | 101 | 2 | 3.38 | 0.85587 | 1 |
| GO:0005216 | ion channel activity | 57 | 1 | 1.9 | 0.85646 | 1 |
| GO:0022838 | substrate-specific channel activity | 57 | 1 | 1.9 | 0.85646 | 1 |
| GO:0008238 | exopeptidase activity | 104 | 2 | 3.48 | 0.86691 | 1 |
| GO:0008092 | cytoskeletal protein binding | 145 | 3 | 4.85 | 0.86744 | 1 |
| GO:0015077 | monovalent inorganic cation transmembran... | 147 | 3 | 4.91 | 0.87341 | 1 |
| GO:0050661 | NADP binding | 62 | 1 | 2.07 | 0.87897 | 1 |
| GO:0016835 | carbon-oxygen lyase activity | 81 | 1 | 2.71 | 0.93676 | 1 |
| GO:0034062 | 5'-3' RNA polymerase activity | 82 | 1 | 2.74 | 0.93888 | 1 |
| GO:0097747 | RNA polymerase activity | 82 | 1 | 2.74 | 0.93888 | 1 |
| GO:0008289 | lipid binding | 91 | 1 | 3.04 | 0.95507 | 1 |
| GO:0001883 | purine nucleoside binding | 275 | 5 | 9.19 | 0.95518 | 1 |
| GO:0005525 | GTP binding | 275 | 5 | 9.19 | 0.95518 | 1 |
| GO:0032549 | ribonucleoside binding | 275 | 5 | 9.19 | 0.95518 | 1 |
| GO:0032550 | purine ribonucleoside binding | 275 | 5 | 9.19 | 0.95518 | 1 |
| GO:0001882 | nucleoside binding | 276 | 5 | 9.22 | 0.95614 | 1 |
| GO:0022857 | transmembrane transporter activity | 1008 | 25 | 33.68 | 0.95727 | 1 |
| GO:0032561 | guanyl ribonucleotide binding | 278 | 5 | 9.29 | 0.958 | 1 |
| GO:0008234 | cysteine-type peptidase activity | 93 | 1 | 3.11 | 0.95804 | 1 |
| GO:0015075 | ion transmembrane transporter activity | 363 | 7 | 12.13 | 0.9616 | 1 |
| GO:0016853 | isomerase activity | 198 | 3 | 6.62 | 0.96366 | 1 |
| GO:0019001 | guanyl nucleotide binding | 289 | 5 | 9.66 | 0.967 | 1 |
| GO:0022890 | inorganic cation transmembrane transport... | 204 | 3 | 6.82 | 0.96886 | 1 |
| GO:0016779 | nucleotidyltransferase activity | 160 | 2 | 5.35 | 0.97219 | 1 |
| GO:0003723 | RNA binding | 383 | 7 | 12.8 | 0.97424 | 1 |
| GO:0015078 | hydrogen ion transmembrane transporter a... | 109 | 1 | 3.64 | 0.97574 | 1 |
| GO:0005215 | transporter activity | 1067 | 25 | 35.66 | 0.98009 | 1 |
| GO:0008236 | serine-type peptidase activity | 172 | 2 | 5.75 | 0.98041 | 1 |
| GO:0017171 | serine hydrolase activity | 172 | 2 | 5.75 | 0.98041 | 1 |
| GO:0022891 | substrate-specific transmembrane transpo... | 412 | 7 | 13.77 | 0.98591 | 1 |
| GO:0008324 | cation transmembrane transporter activit... | 247 | 3 | 8.25 | 0.99008 | 1 |
| GO:0022892 | substrate-specific transporter activity | 439 | 7 | 14.67 | 0.99215 | 1 |
| GO:0070011 | peptidase activity, acting on L-amino ac... | 417 | 5 | 13.93 | 0.9985 | 1 |
| GO:0008233 | peptidase activity | 437 | 5 | 14.6 | 0.99911 | 1 |
| GO:0003674 | molecular_function | 14454 | 483 | 483 | 1 | 1 |
| GO:0000049 | tRNA binding | 8 | 0 | 0.27 | 1 | 1 |
| GO:0000155 | phosphorelay sensor kinase activity | 35 | 0 | 1.17 | 1 | 1 |
| GO:0000988 | transcription factor activity, protein b... | 26 | 0 | 0.87 | 1 | 1 |
| GO:0000989 | transcription factor activity, transcrip... | 23 | 0 | 0.77 | 1 | 1 |
| GO:0001671 | ATPase activator activity | 5 | 0 | 0.17 | 1 | 1 |
| GO:0002161 | aminoacyl-tRNA editing activity | 7 | 0 | 0.23 | 1 | 1 |
| GO:0003678 | DNA helicase activity | 19 | 0 | 0.63 | 1 | 1 |
| GO:0003682 | chromatin binding | 17 | 0 | 0.57 | 1 | 1 |
| GO:0003690 | double-stranded DNA binding | 58 | 0 | 1.94 | 1 | 1 |
| GO:0003697 | single-stranded DNA binding | 11 | 0 | 0.37 | 1 | 1 |
| GO:0003712 | transcription cofactor activity | 22 | 0 | 0.74 | 1 | 1 |
| GO:0003725 | double-stranded RNA binding | 5 | 0 | 0.17 | 1 | 1 |
| GO:0003729 | mRNA binding | 9 | 0 | 0.3 | 1 | 1 |
| GO:0003743 | translation initiation factor activity | 49 | 0 | 1.64 | 1 | 1 |
| GO:0003746 | translation elongation factor activity | 17 | 0 | 0.57 | 1 | 1 |
| GO:0003747 | translation release factor activity | 7 | 0 | 0.23 | 1 | 1 |
| GO:0003755 | peptidyl-prolyl cis-trans isomerase acti... | 50 | 0 | 1.67 | 1 | 1 |
| GO:0003779 | actin binding | 51 | 0 | 1.7 | 1 | 1 |
| GO:0003830 | beta-1,4-mannosylglycoprotein 4-beta-N-a... | 7 | 0 | 0.23 | 1 | 1 |
| GO:0003843 | 1,3-beta-D-glucan synthase activity | 20 | 0 | 0.67 | 1 | 1 |
| GO:0003849 | 3-deoxy-7-phosphoheptulonate synthase ac... | 5 | 0 | 0.17 | 1 | 1 |
| GO:0003857 | 3-hydroxyacyl-CoA dehydrogenase activity | 8 | 0 | 0.27 | 1 | 1 |
| GO:0003860 | 3-hydroxyisobutyryl-CoA hydrolase activi... | 6 | 0 | 0.2 | 1 | 1 |
| GO:0003871 | 5-methyltetrahydropteroyltriglutamate-ho... | 6 | 0 | 0.2 | 1 | 1 |
| GO:0003872 | 6-phosphofructokinase activity | 9 | 0 | 0.3 | 1 | 1 |
| GO:0003879 | ATP phosphoribosyltransferase activity | 5 | 0 | 0.17 | 1 | 1 |
| GO:0003883 | CTP synthase activity | 7 | 0 | 0.23 | 1 | 1 |
| GO:0003887 | DNA-directed DNA polymerase activity | 14 | 0 | 0.47 | 1 | 1 |
| GO:0003899 | DNA-directed 5'-3' RNA polymerase activi... | 61 | 0 | 2.04 | 1 | 1 |
| GO:0003905 | alkylbase DNA N-glycosylase activity | 7 | 0 | 0.23 | 1 | 1 |
| GO:0003909 | DNA ligase activity | 5 | 0 | 0.17 | 1 | 1 |
| GO:0003916 | DNA topoisomerase activity | 26 | 0 | 0.87 | 1 | 1 |
| GO:0003918 | DNA topoisomerase type II (ATP-hydrolyzi... | 13 | 0 | 0.43 | 1 | 1 |
| GO:0003951 | NAD+ kinase activity | 6 | 0 | 0.2 | 1 | 1 |
| GO:0003954 | NADH dehydrogenase activity | 15 | 0 | 0.5 | 1 | 1 |
| GO:0003993 | acid phosphatase activity | 14 | 0 | 0.47 | 1 | 1 |
| GO:0003997 | acyl-CoA oxidase activity | 10 | 0 | 0.33 | 1 | 1 |
| GO:0004003 | ATP-dependent DNA helicase activity | 10 | 0 | 0.33 | 1 | 1 |
| GO:0004014 | adenosylmethionine decarboxylase activit... | 7 | 0 | 0.23 | 1 | 1 |
| GO:0004019 | adenylosuccinate synthase activity | 7 | 0 | 0.23 | 1 | 1 |
| GO:0004061 | arylformamidase activity | 6 | 0 | 0.2 | 1 | 1 |
| GO:0004066 | asparagine synthase (glutamine-hydrolyzi... | 11 | 0 | 0.37 | 1 | 1 |
| GO:0004089 | carbonate dehydratase activity | 10 | 0 | 0.33 | 1 | 1 |
| GO:0004096 | catalase activity | 21 | 0 | 0.7 | 1 | 1 |
| GO:0004129 | cytochrome-c oxidase activity | 18 | 0 | 0.6 | 1 | 1 |
| GO:0004144 | diacylglycerol O-acyltransferase activit... | 6 | 0 | 0.2 | 1 | 1 |
| GO:0004175 | endopeptidase activity | 202 | 0 | 6.75 | 1 | 1 |
| GO:0004177 | aminopeptidase activity | 18 | 0 | 0.6 | 1 | 1 |
| GO:0004181 | metallocarboxypeptidase activity | 5 | 0 | 0.17 | 1 | 1 |
| GO:0004190 | aspartic-type endopeptidase activity | 18 | 0 | 0.6 | 1 | 1 |
| GO:0004197 | cysteine-type endopeptidase activity | 10 | 0 | 0.33 | 1 | 1 |
| GO:0004222 | metalloendopeptidase activity | 53 | 0 | 1.77 | 1 | 1 |
| GO:0004252 | serine-type endopeptidase activity | 75 | 0 | 2.51 | 1 | 1 |
| GO:0004298 | threonine-type endopeptidase activity | 42 | 0 | 1.4 | 1 | 1 |
| GO:0004347 | glucose-6-phosphate isomerase activity | 6 | 0 | 0.2 | 1 | 1 |
| GO:0004356 | glutamate-ammonia ligase activity | 14 | 0 | 0.47 | 1 | 1 |
| GO:0004357 | glutamate-cysteine ligase activity | 5 | 0 | 0.17 | 1 | 1 |
| GO:0004359 | glutaminase activity | 5 | 0 | 0.17 | 1 | 1 |
| GO:0004367 | glycerol-3-phosphate dehydrogenase [NAD+... | 5 | 0 | 0.17 | 1 | 1 |
| GO:0004375 | glycine dehydrogenase (decarboxylating) ... | 5 | 0 | 0.17 | 1 | 1 |
| GO:0004386 | helicase activity | 45 | 0 | 1.5 | 1 | 1 |
| GO:0004402 | histone acetyltransferase activity | 8 | 0 | 0.27 | 1 | 1 |
| GO:0004421 | hydroxymethylglutaryl-CoA synthase activ... | 5 | 0 | 0.17 | 1 | 1 |
| GO:0004451 | isocitrate lyase activity | 14 | 0 | 0.47 | 1 | 1 |
| GO:0004470 | malic enzyme activity | 8 | 0 | 0.27 | 1 | 1 |
| GO:0004471 | malate dehydrogenase (decarboxylating) (... | 8 | 0 | 0.27 | 1 | 1 |
| GO:0004474 | malate synthase activity | 9 | 0 | 0.3 | 1 | 1 |
| GO:0004478 | methionine adenosyltransferase activity | 15 | 0 | 0.5 | 1 | 1 |
| GO:0004488 | methylenetetrahydrofolate dehydrogenase ... | 6 | 0 | 0.2 | 1 | 1 |
| GO:0004497 | monooxygenase activity | 33 | 0 | 1.1 | 1 | 1 |
| GO:0004499 | N,N-dimethylaniline monooxygenase activi... | 22 | 0 | 0.74 | 1 | 1 |
| GO:0004506 | squalene monooxygenase activity | 5 | 0 | 0.17 | 1 | 1 |
| GO:0004525 | ribonuclease III activity | 8 | 0 | 0.27 | 1 | 1 |
| GO:0004527 | exonuclease activity | 23 | 0 | 0.77 | 1 | 1 |
| GO:0004529 | exodeoxyribonuclease activity | 5 | 0 | 0.17 | 1 | 1 |
| GO:0004536 | deoxyribonuclease activity | 6 | 0 | 0.2 | 1 | 1 |
| GO:0004576 | oligosaccharyl transferase activity | 20 | 0 | 0.67 | 1 | 1 |
| GO:0004579 | dolichyl-diphosphooligosaccharide-protei... | 10 | 0 | 0.33 | 1 | 1 |
| GO:0004602 | glutathione peroxidase activity | 8 | 0 | 0.27 | 1 | 1 |
| GO:0004609 | phosphatidylserine decarboxylase activit... | 5 | 0 | 0.17 | 1 | 1 |
| GO:0004611 | phosphoenolpyruvate carboxykinase activi... | 13 | 0 | 0.43 | 1 | 1 |
| GO:0004612 | phosphoenolpyruvate carboxykinase (ATP) ... | 6 | 0 | 0.2 | 1 | 1 |
| GO:0004616 | phosphogluconate dehydrogenase (decarbox... | 6 | 0 | 0.2 | 1 | 1 |
| GO:0004618 | phosphoglycerate kinase activity | 8 | 0 | 0.27 | 1 | 1 |
| GO:0004620 | phospholipase activity | 8 | 0 | 0.27 | 1 | 1 |
| GO:0004645 | phosphorylase activity | 17 | 0 | 0.57 | 1 | 1 |
| GO:0004650 | polygalacturonase activity | 24 | 0 | 0.8 | 1 | 1 |
| GO:0004652 | polynucleotide adenylyltransferase activ... | 5 | 0 | 0.17 | 1 | 1 |
| GO:0004664 | prephenate dehydratase activity | 5 | 0 | 0.17 | 1 | 1 |
| GO:0004673 | protein histidine kinase activity | 37 | 0 | 1.24 | 1 | 1 |
| GO:0004725 | protein tyrosine phosphatase activity | 7 | 0 | 0.23 | 1 | 1 |
| GO:0004748 | ribonucleoside-diphosphate reductase act... | 6 | 0 | 0.2 | 1 | 1 |
| GO:0004779 | sulfate adenylyltransferase activity | 5 | 0 | 0.17 | 1 | 1 |
| GO:0004781 | sulfate adenylyltransferase (ATP) activi... | 5 | 0 | 0.17 | 1 | 1 |
| GO:0004784 | superoxide dismutase activity | 8 | 0 | 0.27 | 1 | 1 |
| GO:0004803 | transposase activity | 11 | 0 | 0.37 | 1 | 1 |
| GO:0004809 | tRNA (guanine-N2-)-methyltransferase act... | 6 | 0 | 0.2 | 1 | 1 |
| GO:0004813 | alanine-tRNA ligase activity | 7 | 0 | 0.23 | 1 | 1 |
| GO:0004814 | arginine-tRNA ligase activity | 5 | 0 | 0.17 | 1 | 1 |
| GO:0004834 | tryptophan synthase activity | 8 | 0 | 0.27 | 1 | 1 |
| GO:0004853 | uroporphyrinogen decarboxylase activity | 5 | 0 | 0.17 | 1 | 1 |
| GO:0004867 | serine-type endopeptidase inhibitor acti... | 8 | 0 | 0.27 | 1 | 1 |
| GO:0004869 | cysteine-type endopeptidase inhibitor ac... | 12 | 0 | 0.4 | 1 | 1 |
| GO:0004871 | signal transducer activity | 60 | 0 | 2 | 1 | 1 |
| GO:0004872 | receptor activity | 62 | 0 | 2.07 | 1 | 1 |
| GO:0004888 | transmembrane signaling receptor activit... | 17 | 0 | 0.57 | 1 | 1 |
| GO:0004930 | G-protein coupled receptor activity | 8 | 0 | 0.27 | 1 | 1 |
| GO:0004970 | ionotropic glutamate receptor activity | 6 | 0 | 0.2 | 1 | 1 |
| GO:0005048 | signal sequence binding | 17 | 0 | 0.57 | 1 | 1 |
| GO:0005086 | ARF guanyl-nucleotide exchange factor ac... | 5 | 0 | 0.17 | 1 | 1 |
| GO:0005092 | GDP-dissociation inhibitor activity | 12 | 0 | 0.4 | 1 | 1 |
| GO:0005102 | receptor binding | 14 | 0 | 0.47 | 1 | 1 |
| GO:0005201 | extracellular matrix structural constitu... | 8 | 0 | 0.27 | 1 | 1 |
| GO:0005230 | extracellular ligand-gated ion channel a... | 7 | 0 | 0.23 | 1 | 1 |
| GO:0005244 | voltage-gated ion channel activity | 13 | 0 | 0.43 | 1 | 1 |
| GO:0005247 | voltage-gated chloride channel activity | 13 | 0 | 0.43 | 1 | 1 |
| GO:0005253 | anion channel activity | 13 | 0 | 0.43 | 1 | 1 |
| GO:0005254 | chloride channel activity | 13 | 0 | 0.43 | 1 | 1 |
| GO:0005283 | sodium:amino acid symporter activity | 5 | 0 | 0.17 | 1 | 1 |
| GO:0005310 | dicarboxylic acid transmembrane transpor... | 5 | 0 | 0.17 | 1 | 1 |
| GO:0005315 | inorganic phosphate transmembrane transp... | 6 | 0 | 0.2 | 1 | 1 |
| GO:0005319 | lipid transporter activity | 14 | 0 | 0.47 | 1 | 1 |
| GO:0005337 | nucleoside transmembrane transporter act... | 11 | 0 | 0.37 | 1 | 1 |
| GO:0005338 | nucleotide-sugar transmembrane transport... | 7 | 0 | 0.23 | 1 | 1 |
| GO:0005342 | organic acid transmembrane transporter a... | 32 | 0 | 1.07 | 1 | 1 |
| GO:0005343 | organic acid:sodium symporter activity | 5 | 0 | 0.17 | 1 | 1 |
| GO:0005375 | copper ion transmembrane transporter act... | 6 | 0 | 0.2 | 1 | 1 |
| GO:0005416 | cation:amino acid symporter activity | 5 | 0 | 0.17 | 1 | 1 |
| GO:0005520 | insulin-like growth factor binding | 5 | 0 | 0.17 | 1 | 1 |
| GO:0005539 | glycosaminoglycan binding | 6 | 0 | 0.2 | 1 | 1 |
| GO:0005543 | phospholipid binding | 55 | 0 | 1.84 | 1 | 1 |
| GO:0005544 | calcium-dependent phospholipid binding | 20 | 0 | 0.67 | 1 | 1 |
| GO:0005548 | phospholipid transporter activity | 5 | 0 | 0.17 | 1 | 1 |
| GO:0008026 | ATP-dependent helicase activity | 15 | 0 | 0.5 | 1 | 1 |
| GO:0008028 | monocarboxylic acid transmembrane transp... | 22 | 0 | 0.74 | 1 | 1 |
| GO:0008066 | glutamate receptor activity | 6 | 0 | 0.2 | 1 | 1 |
| GO:0008079 | translation termination factor activity | 7 | 0 | 0.23 | 1 | 1 |
| GO:0008080 | N-acetyltransferase activity | 9 | 0 | 0.3 | 1 | 1 |
| GO:0008083 | growth factor activity | 9 | 0 | 0.3 | 1 | 1 |
| GO:0008094 | DNA-dependent ATPase activity | 24 | 0 | 0.8 | 1 | 1 |
| GO:0008107 | galactoside 2-alpha-L-fucosyltransferase... | 7 | 0 | 0.23 | 1 | 1 |
| GO:0008131 | primary amine oxidase activity | 19 | 0 | 0.63 | 1 | 1 |
| GO:0008135 | translation factor activity, RNA binding | 73 | 0 | 2.44 | 1 | 1 |
| GO:0008137 | NADH dehydrogenase (ubiquinone) activity | 15 | 0 | 0.5 | 1 | 1 |
| GO:0008144 | drug binding | 7 | 0 | 0.23 | 1 | 1 |
| GO:0008146 | sulfotransferase activity | 13 | 0 | 0.43 | 1 | 1 |
| GO:0008170 | N-methyltransferase activity | 20 | 0 | 0.67 | 1 | 1 |
| GO:0008172 | S-methyltransferase activity | 8 | 0 | 0.27 | 1 | 1 |
| GO:0008173 | RNA methyltransferase activity | 21 | 0 | 0.7 | 1 | 1 |
| GO:0008175 | tRNA methyltransferase activity | 8 | 0 | 0.27 | 1 | 1 |
| GO:0008184 | glycogen phosphorylase activity | 17 | 0 | 0.57 | 1 | 1 |
| GO:0008198 | ferrous iron binding | 6 | 0 | 0.2 | 1 | 1 |
| GO:0008199 | ferric iron binding | 10 | 0 | 0.33 | 1 | 1 |
| GO:0008235 | metalloexopeptidase activity | 6 | 0 | 0.2 | 1 | 1 |
| GO:0008276 | protein methyltransferase activity | 12 | 0 | 0.4 | 1 | 1 |
| GO:0008308 | voltage-gated anion channel activity | 13 | 0 | 0.43 | 1 | 1 |
| GO:0008312 | 7S RNA binding | 6 | 0 | 0.2 | 1 | 1 |
| GO:0008374 | O-acyltransferase activity | 19 | 0 | 0.63 | 1 | 1 |
| GO:0008378 | galactosyltransferase activity | 17 | 0 | 0.57 | 1 | 1 |
| GO:0008408 | 3'-5' exonuclease activity | 15 | 0 | 0.5 | 1 | 1 |
| GO:0008417 | fucosyltransferase activity | 9 | 0 | 0.3 | 1 | 1 |
| GO:0008443 | phosphofructokinase activity | 12 | 0 | 0.4 | 1 | 1 |
| GO:0008452 | RNA ligase activity | 5 | 0 | 0.17 | 1 | 1 |
| GO:0008483 | transaminase activity | 23 | 0 | 0.77 | 1 | 1 |
| GO:0008484 | sulfuric ester hydrolase activity | 15 | 0 | 0.5 | 1 | 1 |
| GO:0008519 | ammonium transmembrane transporter activ... | 15 | 0 | 0.5 | 1 | 1 |
| GO:0008536 | Ran GTPase binding | 11 | 0 | 0.37 | 1 | 1 |
| GO:0008565 | protein transporter activity | 13 | 0 | 0.43 | 1 | 1 |
| GO:0008641 | ubiquitin-like modifier activating enzym... | 15 | 0 | 0.5 | 1 | 1 |
| GO:0008658 | penicillin binding | 7 | 0 | 0.23 | 1 | 1 |
| GO:0008661 | 1-deoxy-D-xylulose-5-phosphate synthase ... | 7 | 0 | 0.23 | 1 | 1 |
| GO:0008686 | 3,4-dihydroxy-2-butanone-4-phosphate syn... | 5 | 0 | 0.17 | 1 | 1 |
| GO:0008725 | DNA-3-methyladenine glycosylase activity | 7 | 0 | 0.23 | 1 | 1 |
| GO:0008745 | N-acetylmuramoyl-L-alanine amidase activ... | 6 | 0 | 0.2 | 1 | 1 |
| GO:0008757 | S-adenosylmethionine-dependent methyltra... | 25 | 0 | 0.84 | 1 | 1 |
| GO:0008839 | 4-hydroxy-tetrahydrodipicolinate reducta... | 5 | 0 | 0.17 | 1 | 1 |
| GO:0008964 | phosphoenolpyruvate carboxylase activity | 7 | 0 | 0.23 | 1 | 1 |
| GO:0008982 | protein-N(PI)-phosphohistidine-sugar pho... | 25 | 0 | 0.84 | 1 | 1 |
| GO:0009001 | serine O-acetyltransferase activity | 6 | 0 | 0.2 | 1 | 1 |
| GO:0009002 | serine-type D-Ala-D-Ala carboxypeptidase... | 7 | 0 | 0.23 | 1 | 1 |
| GO:0009916 | alternative oxidase activity | 5 | 0 | 0.17 | 1 | 1 |
| GO:0009982 | pseudouridine synthase activity | 23 | 0 | 0.77 | 1 | 1 |
| GO:0010181 | FMN binding | 25 | 0 | 0.84 | 1 | 1 |
| GO:0010242 | oxygen evolving activity | 5 | 0 | 0.17 | 1 | 1 |
| GO:0010333 | terpene synthase activity | 33 | 0 | 1.1 | 1 | 1 |
| GO:0015002 | heme-copper terminal oxidase activity | 22 | 0 | 0.74 | 1 | 1 |
| GO:0015018 | galactosylgalactosylxylosylprotein 3-bet... | 15 | 0 | 0.5 | 1 | 1 |
| GO:0015020 | glucuronosyltransferase activity | 15 | 0 | 0.5 | 1 | 1 |
| GO:0015081 | sodium ion transmembrane transporter act... | 10 | 0 | 0.33 | 1 | 1 |
| GO:0015095 | magnesium ion transmembrane transporter ... | 12 | 0 | 0.4 | 1 | 1 |
| GO:0015108 | chloride transmembrane transporter activ... | 13 | 0 | 0.43 | 1 | 1 |
| GO:0015116 | sulfate transmembrane transporter activi... | 24 | 0 | 0.8 | 1 | 1 |
| GO:0015128 | gluconate transmembrane transporter acti... | 14 | 0 | 0.47 | 1 | 1 |
| GO:0015129 | lactate transmembrane transporter activi... | 6 | 0 | 0.2 | 1 | 1 |
| GO:0015144 | carbohydrate transmembrane transporter a... | 43 | 0 | 1.44 | 1 | 1 |
| GO:0015165 | pyrimidine nucleotide-sugar transmembran... | 7 | 0 | 0.23 | 1 | 1 |
| GO:0015171 | amino acid transmembrane transporter act... | 7 | 0 | 0.23 | 1 | 1 |
| GO:0015197 | peptide transporter activity | 16 | 0 | 0.53 | 1 | 1 |
| GO:0015276 | ligand-gated ion channel activity | 7 | 0 | 0.23 | 1 | 1 |
| GO:0015288 | porin activity | 32 | 0 | 1.07 | 1 | 1 |
| GO:0015293 | symporter activity | 18 | 0 | 0.6 | 1 | 1 |
| GO:0015294 | solute:cation symporter activity | 7 | 0 | 0.23 | 1 | 1 |
| GO:0015296 | anion:cation symporter activity | 6 | 0 | 0.2 | 1 | 1 |
| GO:0015370 | solute:sodium symporter activity | 5 | 0 | 0.17 | 1 | 1 |
| GO:0015473 | fimbrial usher porin activity | 20 | 0 | 0.67 | 1 | 1 |
| GO:0015562 | efflux transmembrane transporter activit... | 8 | 0 | 0.27 | 1 | 1 |
| GO:0015930 | glutamate synthase activity | 8 | 0 | 0.27 | 1 | 1 |
| GO:0016151 | nickel cation binding | 9 | 0 | 0.3 | 1 | 1 |
| GO:0016157 | sucrose synthase activity | 6 | 0 | 0.2 | 1 | 1 |
| GO:0016160 | amylase activity | 12 | 0 | 0.4 | 1 | 1 |
| GO:0016161 | beta-amylase activity | 11 | 0 | 0.37 | 1 | 1 |
| GO:0016211 | ammonia ligase activity | 15 | 0 | 0.5 | 1 | 1 |
| GO:0016278 | lysine N-methyltransferase activity | 10 | 0 | 0.33 | 1 | 1 |
| GO:0016279 | protein-lysine N-methyltransferase activ... | 10 | 0 | 0.33 | 1 | 1 |
| GO:0016289 | CoA hydrolase activity | 6 | 0 | 0.2 | 1 | 1 |
| GO:0016298 | lipase activity | 9 | 0 | 0.3 | 1 | 1 |
| GO:0016407 | acetyltransferase activity | 18 | 0 | 0.6 | 1 | 1 |
| GO:0016410 | N-acyltransferase activity | 13 | 0 | 0.43 | 1 | 1 |
| GO:0016411 | acylglycerol O-acyltransferase activity | 6 | 0 | 0.2 | 1 | 1 |
| GO:0016412 | serine O-acyltransferase activity | 6 | 0 | 0.2 | 1 | 1 |
| GO:0016413 | O-acetyltransferase activity | 6 | 0 | 0.2 | 1 | 1 |
| GO:0016423 | tRNA (guanine) methyltransferase activit... | 7 | 0 | 0.23 | 1 | 1 |
| GO:0016597 | amino acid binding | 5 | 0 | 0.17 | 1 | 1 |
| GO:0016615 | malate dehydrogenase activity | 9 | 0 | 0.3 | 1 | 1 |
| GO:0016620 | oxidoreductase activity, acting on the a... | 45 | 0 | 1.5 | 1 | 1 |
| GO:0016624 | oxidoreductase activity, acting on the a... | 7 | 0 | 0.23 | 1 | 1 |
| GO:0016634 | oxidoreductase activity, acting on the C... | 12 | 0 | 0.4 | 1 | 1 |
| GO:0016638 | oxidoreductase activity, acting on the C... | 34 | 0 | 1.14 | 1 | 1 |
| GO:0016641 | oxidoreductase activity, acting on the C... | 20 | 0 | 0.67 | 1 | 1 |
| GO:0016642 | oxidoreductase activity, acting on the C... | 5 | 0 | 0.17 | 1 | 1 |
| GO:0016655 | oxidoreductase activity, acting on NAD(P... | 16 | 0 | 0.53 | 1 | 1 |
| GO:0016675 | oxidoreductase activity, acting on a hem... | 18 | 0 | 0.6 | 1 | 1 |
| GO:0016676 | oxidoreductase activity, acting on a hem... | 18 | 0 | 0.6 | 1 | 1 |
| GO:0016679 | oxidoreductase activity, acting on diphe... | 8 | 0 | 0.27 | 1 | 1 |
| GO:0016682 | oxidoreductase activity, acting on diphe... | 7 | 0 | 0.23 | 1 | 1 |
| GO:0016703 | oxidoreductase activity, acting on singl... | 5 | 0 | 0.17 | 1 | 1 |
| GO:0016709 | oxidoreductase activity, acting on paire... | 27 | 0 | 0.9 | 1 | 1 |
| GO:0016721 | oxidoreductase activity, acting on super... | 8 | 0 | 0.27 | 1 | 1 |
| GO:0016725 | oxidoreductase activity, acting on CH or... | 14 | 0 | 0.47 | 1 | 1 |
| GO:0016726 | oxidoreductase activity, acting on CH or... | 5 | 0 | 0.17 | 1 | 1 |
| GO:0016728 | oxidoreductase activity, acting on CH or... | 6 | 0 | 0.2 | 1 | 1 |
| GO:0016742 | hydroxymethyl-, formyl- and related tran... | 14 | 0 | 0.47 | 1 | 1 |
| GO:0016743 | carboxyl- or carbamoyltransferase activi... | 9 | 0 | 0.3 | 1 | 1 |
| GO:0016744 | transferase activity, transferring aldeh... | 7 | 0 | 0.23 | 1 | 1 |
| GO:0016755 | transferase activity, transferring amino... | 9 | 0 | 0.3 | 1 | 1 |
| GO:0016763 | transferase activity, transferring pento... | 19 | 0 | 0.63 | 1 | 1 |
| GO:0016769 | transferase activity, transferring nitro... | 24 | 0 | 0.8 | 1 | 1 |
| GO:0016774 | phosphotransferase activity, carboxyl gr... | 11 | 0 | 0.37 | 1 | 1 |
| GO:0016775 | phosphotransferase activity, nitrogenous... | 37 | 0 | 1.24 | 1 | 1 |
| GO:0016780 | phosphotransferase activity, for other s... | 9 | 0 | 0.3 | 1 | 1 |
| GO:0016782 | transferase activity, transferring sulfu... | 16 | 0 | 0.53 | 1 | 1 |
| GO:0016790 | thiolester hydrolase activity | 10 | 0 | 0.33 | 1 | 1 |
| GO:0016796 | exonuclease activity, active with either... | 5 | 0 | 0.17 | 1 | 1 |
| GO:0016811 | hydrolase activity, acting on carbon-nit... | 28 | 0 | 0.94 | 1 | 1 |
| GO:0016833 | oxo-acid-lyase activity | 15 | 0 | 0.5 | 1 | 1 |
| GO:0016836 | hydro-lyase activity | 38 | 0 | 1.27 | 1 | 1 |
| GO:0016838 | carbon-oxygen lyase activity, acting on ... | 38 | 0 | 1.27 | 1 | 1 |
| GO:0016840 | carbon-nitrogen lyase activity | 15 | 0 | 0.5 | 1 | 1 |
| GO:0016841 | ammonia-lyase activity | 5 | 0 | 0.17 | 1 | 1 |
| GO:0016843 | amine-lyase activity | 8 | 0 | 0.27 | 1 | 1 |
| GO:0016844 | strictosidine synthase activity | 8 | 0 | 0.27 | 1 | 1 |
| GO:0016854 | racemase and epimerase activity | 18 | 0 | 0.6 | 1 | 1 |
| GO:0016855 | racemase and epimerase activity, acting ... | 7 | 0 | 0.23 | 1 | 1 |
| GO:0016857 | racemase and epimerase activity, acting ... | 11 | 0 | 0.37 | 1 | 1 |
| GO:0016859 | cis-trans isomerase activity | 50 | 0 | 1.67 | 1 | 1 |
| GO:0016866 | intramolecular transferase activity | 40 | 0 | 1.34 | 1 | 1 |
| GO:0016868 | intramolecular transferase activity, pho... | 16 | 0 | 0.53 | 1 | 1 |
| GO:0016872 | intramolecular lyase activity | 6 | 0 | 0.2 | 1 | 1 |
| GO:0016877 | ligase activity, forming carbon-sulfur b... | 17 | 0 | 0.57 | 1 | 1 |
| GO:0016880 | acid-ammonia (or amide) ligase activity | 15 | 0 | 0.5 | 1 | 1 |
| GO:0016884 | carbon-nitrogen ligase activity, with gl... | 13 | 0 | 0.43 | 1 | 1 |
| GO:0016886 | ligase activity, forming phosphoric este... | 10 | 0 | 0.33 | 1 | 1 |
| GO:0016895 | exodeoxyribonuclease activity, producing... | 5 | 0 | 0.17 | 1 | 1 |
| GO:0016903 | oxidoreductase activity, acting on the a... | 55 | 0 | 1.84 | 1 | 1 |
| GO:0017056 | structural constituent of nuclear pore | 5 | 0 | 0.17 | 1 | 1 |
| GO:0017150 | tRNA dihydrouridine synthase activity | 10 | 0 | 0.33 | 1 | 1 |
| GO:0018024 | histone-lysine N-methyltransferase activ... | 10 | 0 | 0.33 | 1 | 1 |
| GO:0019104 | DNA N-glycosylase activity | 8 | 0 | 0.27 | 1 | 1 |
| GO:0019139 | cytokinin dehydrogenase activity | 9 | 0 | 0.3 | 1 | 1 |
| GO:0019200 | carbohydrate kinase activity | 15 | 0 | 0.5 | 1 | 1 |
| GO:0019203 | carbohydrate phosphatase activity | 5 | 0 | 0.17 | 1 | 1 |
| GO:0019205 | nucleobase-containing compound kinase ac... | 6 | 0 | 0.2 | 1 | 1 |
| GO:0019207 | kinase regulator activity | 10 | 0 | 0.33 | 1 | 1 |
| GO:0019208 | phosphatase regulator activity | 21 | 0 | 0.7 | 1 | 1 |
| GO:0019829 | cation-transporting ATPase activity | 19 | 0 | 0.63 | 1 | 1 |
| GO:0019838 | growth factor binding | 6 | 0 | 0.2 | 1 | 1 |
| GO:0019887 | protein kinase regulator activity | 10 | 0 | 0.33 | 1 | 1 |
| GO:0019888 | protein phosphatase regulator activity | 19 | 0 | 0.63 | 1 | 1 |
| GO:0022824 | transmitter-gated ion channel activity | 6 | 0 | 0.2 | 1 | 1 |
| GO:0022829 | wide pore channel activity | 32 | 0 | 1.07 | 1 | 1 |
| GO:0022832 | voltage-gated channel activity | 13 | 0 | 0.43 | 1 | 1 |
| GO:0022834 | ligand-gated channel activity | 7 | 0 | 0.23 | 1 | 1 |
| GO:0022835 | transmitter-gated channel activity | 6 | 0 | 0.2 | 1 | 1 |
| GO:0022836 | gated channel activity | 20 | 0 | 0.67 | 1 | 1 |
| GO:0022853 | active ion transmembrane transporter act... | 25 | 0 | 0.84 | 1 | 1 |
| GO:0030145 | manganese ion binding | 10 | 0 | 0.33 | 1 | 1 |
| GO:0030151 | molybdenum ion binding | 5 | 0 | 0.17 | 1 | 1 |
| GO:0030545 | receptor regulator activity | 10 | 0 | 0.33 | 1 | 1 |
| GO:0030594 | neurotransmitter receptor activity | 6 | 0 | 0.2 | 1 | 1 |
| GO:0030976 | thiamine pyrophosphate binding | 26 | 0 | 0.87 | 1 | 1 |
| GO:0030983 | mismatched DNA binding | 15 | 0 | 0.5 | 1 | 1 |
| GO:0031072 | heat shock protein binding | 10 | 0 | 0.33 | 1 | 1 |
| GO:0031127 | alpha-(1,2)-fucosyltransferase activity | 7 | 0 | 0.23 | 1 | 1 |
| GO:0031369 | translation initiation factor binding | 6 | 0 | 0.2 | 1 | 1 |
| GO:0031406 | carboxylic acid binding | 14 | 0 | 0.47 | 1 | 1 |
| GO:0031683 | G-protein beta/gamma-subunit complex bin... | 11 | 0 | 0.37 | 1 | 1 |
| GO:0032296 | double-stranded RNA-specific ribonucleas... | 8 | 0 | 0.27 | 1 | 1 |
| GO:0032403 | protein complex binding | 21 | 0 | 0.7 | 1 | 1 |
| GO:0033218 | amide binding | 26 | 0 | 0.87 | 1 | 1 |
| GO:0033293 | monocarboxylic acid binding | 7 | 0 | 0.23 | 1 | 1 |
| GO:0034212 | peptide N-acetyltransferase activity | 8 | 0 | 0.27 | 1 | 1 |
| GO:0035091 | phosphatidylinositol binding | 15 | 0 | 0.5 | 1 | 1 |
| GO:0036361 | racemase activity, acting on amino acids... | 7 | 0 | 0.23 | 1 | 1 |
| GO:0038023 | signaling receptor activity | 58 | 0 | 1.94 | 1 | 1 |
| GO:0042054 | histone methyltransferase activity | 10 | 0 | 0.33 | 1 | 1 |
| GO:0042085 | 5-methyltetrahydropteroyltri-L-glutamate... | 6 | 0 | 0.2 | 1 | 1 |
| GO:0042277 | peptide binding | 17 | 0 | 0.57 | 1 | 1 |
| GO:0042393 | histone binding | 7 | 0 | 0.23 | 1 | 1 |
| GO:0042625 | ATPase coupled ion transmembrane transpo... | 19 | 0 | 0.63 | 1 | 1 |
| GO:0042879 | aldonate transmembrane transporter activ... | 14 | 0 | 0.47 | 1 | 1 |
| GO:0043015 | gamma-tubulin binding | 7 | 0 | 0.23 | 1 | 1 |
| GO:0043021 | ribonucleoprotein complex binding | 10 | 0 | 0.33 | 1 | 1 |
| GO:0043022 | ribosome binding | 6 | 0 | 0.2 | 1 | 1 |
| GO:0043177 | organic acid binding | 14 | 0 | 0.47 | 1 | 1 |
| GO:0043546 | molybdopterin cofactor binding | 8 | 0 | 0.27 | 1 | 1 |
| GO:0043733 | DNA-3-methylbase glycosylase activity | 7 | 0 | 0.23 | 1 | 1 |
| GO:0044769 | ATPase activity, coupled to transmembran... | 15 | 0 | 0.5 | 1 | 1 |
| GO:0044877 | macromolecular complex binding | 36 | 0 | 1.2 | 1 | 1 |
| GO:0045735 | nutrient reservoir activity | 20 | 0 | 0.67 | 1 | 1 |
| GO:0046912 | transferase activity, transferring acyl ... | 22 | 0 | 0.74 | 1 | 1 |
| GO:0046915 | transition metal ion transmembrane trans... | 11 | 0 | 0.37 | 1 | 1 |
| GO:0046923 | ER retention sequence binding | 14 | 0 | 0.47 | 1 | 1 |
| GO:0046933 | proton-transporting ATP synthase activit... | 12 | 0 | 0.4 | 1 | 1 |
| GO:0046943 | carboxylic acid transmembrane transporte... | 32 | 0 | 1.07 | 1 | 1 |
| GO:0047429 | nucleoside-triphosphate diphosphatase ac... | 5 | 0 | 0.17 | 1 | 1 |
| GO:0048018 | receptor ligand activity | 10 | 0 | 0.33 | 1 | 1 |
| GO:0048038 | quinone binding | 25 | 0 | 0.84 | 1 | 1 |
| GO:0050113 | inositol oxygenase activity | 8 | 0 | 0.27 | 1 | 1 |
| GO:0050136 | NADH dehydrogenase (quinone) activity | 15 | 0 | 0.5 | 1 | 1 |
| GO:0050308 | sugar-phosphatase activity | 5 | 0 | 0.17 | 1 | 1 |
| GO:0051015 | actin filament binding | 9 | 0 | 0.3 | 1 | 1 |
| GO:0051082 | unfolded protein binding | 48 | 0 | 1.6 | 1 | 1 |
| GO:0051536 | iron-sulfur cluster binding | 92 | 0 | 3.07 | 1 | 1 |
| GO:0051537 | 2 iron, 2 sulfur cluster binding | 14 | 0 | 0.47 | 1 | 1 |
| GO:0051539 | 4 iron, 4 sulfur cluster binding | 13 | 0 | 0.43 | 1 | 1 |
| GO:0051540 | metal cluster binding | 92 | 0 | 3.07 | 1 | 1 |
| GO:0051745 | 4-hydroxy-3-methylbut-2-en-1-yl diphosph... | 5 | 0 | 0.17 | 1 | 1 |
| GO:0051920 | peroxiredoxin activity | 7 | 0 | 0.23 | 1 | 1 |
| GO:0060089 | molecular transducer activity | 62 | 0 | 2.07 | 1 | 1 |
| GO:0060590 | ATPase regulator activity | 11 | 0 | 0.37 | 1 | 1 |
| GO:0061505 | DNA topoisomerase II activity | 13 | 0 | 0.43 | 1 | 1 |
| GO:0061630 | ubiquitin protein ligase activity | 13 | 0 | 0.43 | 1 | 1 |
| GO:0061659 | ubiquitin-like protein ligase activity | 13 | 0 | 0.43 | 1 | 1 |
| GO:0061731 | ribonucleoside-diphosphate reductase act... | 6 | 0 | 0.2 | 1 | 1 |
| GO:0061733 | peptide-lysine-N-acetyltransferase activ... | 8 | 0 | 0.27 | 1 | 1 |
| GO:0061783 | peptidoglycan muralytic activity | 10 | 0 | 0.33 | 1 | 1 |
| GO:0070001 | aspartic-type peptidase activity | 18 | 0 | 0.6 | 1 | 1 |
| GO:0070003 | threonine-type peptidase activity | 42 | 0 | 1.4 | 1 | 1 |
| GO:0070035 | purine NTP-dependent helicase activity | 15 | 0 | 0.5 | 1 | 1 |
| GO:0070566 | adenylyltransferase activity | 13 | 0 | 0.43 | 1 | 1 |
| GO:0070569 | uridylyltransferase activity | 7 | 0 | 0.23 | 1 | 1 |
| GO:0072509 | divalent inorganic cation transmembrane ... | 13 | 0 | 0.43 | 1 | 1 |
| GO:0099600 | transmembrane receptor activity | 18 | 0 | 0.6 | 1 | 1 |
| GO:0120013 | intermembrane lipid transfer activity | 8 | 0 | 0.27 | 1 | 1 |
| GO:1901618 | organic hydroxy compound transmembrane t... | 7 | 0 | 0.23 | 1 | 1 |
| GO:1901682 | sulfur compound transmembrane transporte... | 24 | 0 | 0.8 | 1 | 1 |
