# Supplementary material for: Comparative transcriptomic analysis reveals the cold acclimation during chilling stress in sensitive and resistant passion fruit (Passiflora edulis) cultivars
Source: PeerJ. 2021 Mar 3;9:e10977. doi: 10.7717/peerj.10977 (PMC7936571; doi:10.7717/peerj.10977)
Supplement: Supplemental Information 8 [file peerj-09-10977-s008.docx]

Supplementary table 8 Raw Ct value of RT-qPCR

| Gene rank | Gene Symbol | Assay Catalog | Repeat 1 | | | Repeat 2 | | | Repeat 3 | | | Repeat 1 | | | Repeat 2 | | | Repeat 3 | | |
| --- | --- | --- | --- | --- | --- | --- | --- | --- | --- | --- | --- | --- | --- | --- | --- | --- | --- | --- | --- | --- |
|  |  |  | HJB1-1 | HJB1-2 | HJB1-3 | HJB2-1 | HJB2-2 | HJB2-3 | HJB3-1 | HJB3-2 | HJB3-3 | TNB1-1 | TNB1-2 | TNB1-3 | TNB2-1 | TNB2-2 | TNB2-3 | TNB3-1 | TNB3-2 | TNB3-3 |
| 1 | *HIS* | HIS | 21.02 | 20.78 | 21.13 | 20.13 | 20.77 | 20.52 | 19.05 | 19.12 | 19.00 | 20.12 | 20.50 | 20.41 | 20.47 | 20.94 | 20.92 | 21.10 | 21.18 | 21.25 |
| 2 | *TRINITYDN8803_c0_g1_i1* | DN8803_c0_g1_i1 | 24.39 | 24.67 | 24.40 | 24.18 | 24.59 | 24.36 | 22.49 | 22.47 | 22.43 | 25.61 | 25.80 | 25.58 | 25.84 | 26.17 | 26.16 | 27.24 | 27.27 | 27.23 |
| 3 | *TRINITYDN39089_c1_g3_i1* | DN39089_c1_g3_i1 | 27.04 | 27.08 | 27.14 | 26.66 | 26.20 | 26.28 | 24.65 | 24.67 | 24.46 | 24.54 | 24.17 | 24.46 | 24.91 | 24.81 | 25.29 | 25.16 | 24.75 | 25.04 |
| 4 | *TRINITYDN19358_c0_g2_i1* | DN19358_c0_g2_i1 | 26.49 | 26.96 | 27.22 | 25.77 | 26.13 | 26.12 | 24.95 | 24.76 | 24.98 | 26.04 | 25.53 | 25.74 | 26.00 | 26.15 | 26.19 | 27.30 | 27.48 | 27.28 |
| 5 | *TRINITYDN25050_c0_g1_i1* | DN25050_c0_g1_i1 | 26.51 | 26.41 | 26.61 | 25.43 | 26.05 | 25.78 | 24.38 | 24.62 | 24.45 | 28.13 | 28.14 | 28.31 | 29.28 | 29.53 | 28.97 | 28.70 | 28.48 | 28.27 |
| 6 | *TRINITYDN26890_c0_g1_i3* | DN26890_c0_g1_i3 | 31.53 | 31.57 | 31.17 | 31.15 | 31.96 | 30.71 | 29.44 | 30.30 | 30.25 | 27.41 | 27.61 | 27.52 | 27.34 | 27.99 | 27.69 | 27.89 | 28.30 | 28.04 |
| 7 | *TRINITYDN38693_c3_g9_i1* | DN38693_c3_g9_i1 | 26.58 | 26.70 | 27.21 | 26.20 | 26.35 | 26.22 | 25.03 | 25.48 | 25.36 | 23.39 | 23.85 | 23.73 | 23.44 | 23.76 | 23.78 | 24.16 | 24.67 | 24.67 |
| 8 | *TRINITYDN35488_c0_g2_i1* | DN35488_c0_g2_i1 | 25.04 | 25.47 | 25.19 | 24.14 | 24.43 | 24.04 | 23.18 | 23.08 | 22.77 | 22.92 | 23.70 | 23.42 | 23.59 | 23.60 | 23.65 | 24.03 | 24.17 | 24.05 |
| 9 | *TRINITYDN30661_c0_g1_i2* | DN30661_c0_g1_i2 | 25.99 | 26.11 | 26.44 | 25.11 | 25.60 | 25.67 | 24.20 | 25.00 | 25.19 | 29.90 | 29.15 | 30.26 | 30.34 | 29.66 | 30.13 | 30.76 | 31.39 | 31.73 |
| 10 | *TRINITYDN36596_c0_g1_i2* | DN36596_c0_g1_i2 | 22.17 | 22.66 | 22.56 | 21.42 | 21.55 | 21.38 | 20.43 | 21.18 | 20.85 | 20.07 | 20.38 | 20.29 | 20.20 | 20.45 | 20.53 | 21.93 | 22.27 | 22.05 |
| 11 | *TRINITYDN38940_c0_g1_i1* | DN38940_c0_g1_i1 | 25.26 | 25.64 | 25.56 | 24.49 | 24.93 | 25.05 | 24.31 | 24.18 | 24.86 | 23.30 | 23.39 | 23.55 | 23.14 | 23.44 | 23.35 | 24.73 | 24.73 | 24.91 |
| 12 | *TRINITYDN24790_c0_g2_i2* | DN24790_c0_g2_i2 | 27.78 | 27.66 | 28.04 | 27.35 | 27.36 | 27.26 | 26.13 | 26.31 | 26.18 | 26.49 | 26.84 | 26.73 | 26.16 | 26.48 | 26.33 | 25.36 | 25.65 | 25.34 |
| 13 | *TRINITYDN30916_c0_g2_i3* | DN30916_c0_g2_i3 | 21.74 | 21.88 | 21.56 | 20.90 | 20.40 | 20.58 | 19.65 | 19.09 | 19.10 | 19.82 | 20.17 | 20.07 | 20.47 | 20.94 | 20.92 | 20.56 | 20.62 | 20.72 |
| 14 | *TRINITYDN38458_c1_g4_i1* | DN38458_c1_g4_i1 | 24.79 | 25.30 | 25.34 | 24.19 | 23.89 | 24.92 | 23.04 | 22.57 | 23.58 | 23.81 | 22.83 | 22.88 | 23.74 | 24.03 | 24.12 | 24.53 | 25.20 | 23.96 |
| 15 | *TRINITYDN21214_c0_g2_i2* | DN21214_c0_g2_i2 | 32.51 | 32.17 | 33.05 | 31.30 | 31.74 | 32.26 | 29.81 | 30.06 | 29.75 | 33.29 | 33.22 | 32.62 | 33.14 | 33.22 | 33.35 | 33.36 | 33.98 | 33.48 |
| 16 | *TRINITYDN26855_c1_g3_i1* | DN26855_c1_g3_i1 | 25.30 | 25.61 | 25.42 | 24.38 | 24.72 | 24.86 | 23.55 | 23.94 | 23.71 | 25.50 | 25.62 | 25.40 | 25.67 | 25.56 | 25.58 | 24.30 | 24.41 | 24.86 |
