# Supplementary figures and images for: Comparative transcriptomic analysis reveals the cold acclimation during chilling stress in sensitive and resistant passion fruit (Passiflora edulis) cultivars
Source: PeerJ. 2021 Mar 3;9:e10977. doi: 10.7717/peerj.10977 (PMC7936571; doi:10.7717/peerj.10977)

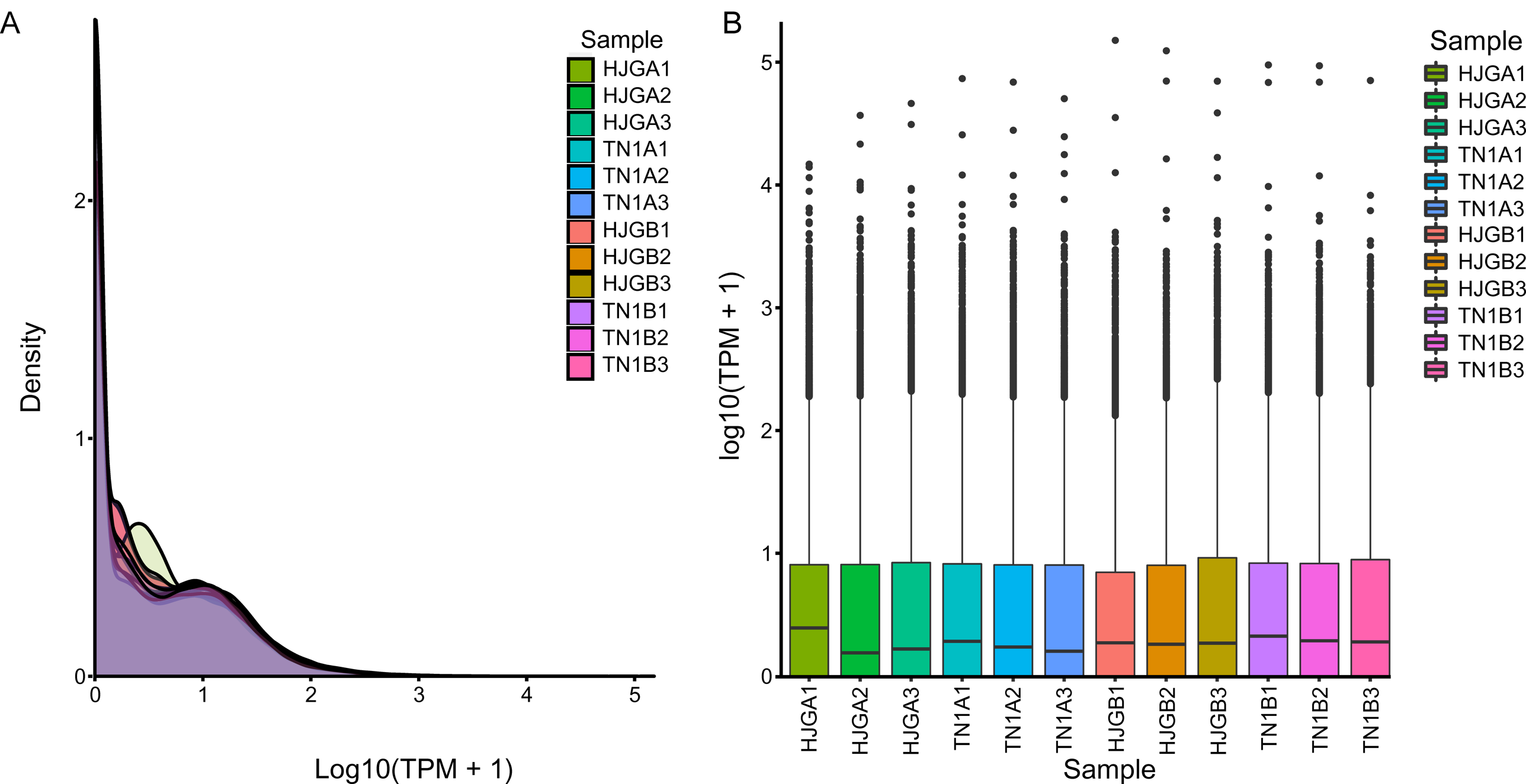

Supplement: Supplemental Information 9 — (A) Distribution curves of gene RPKM density in different samples. The x-axis indicates the log10 (FPKM) value of the genes; the y-axis indicates the density corresponding to log10 (FPKM). Different colors represent different samples. (B) Boxplot showing the distribution of the FPKM values of each sample. The x-axis in the boxplot shows the name of each sample; the y-axis represents the log10 (FPKM). [file peerj-09-10977-s009.png]

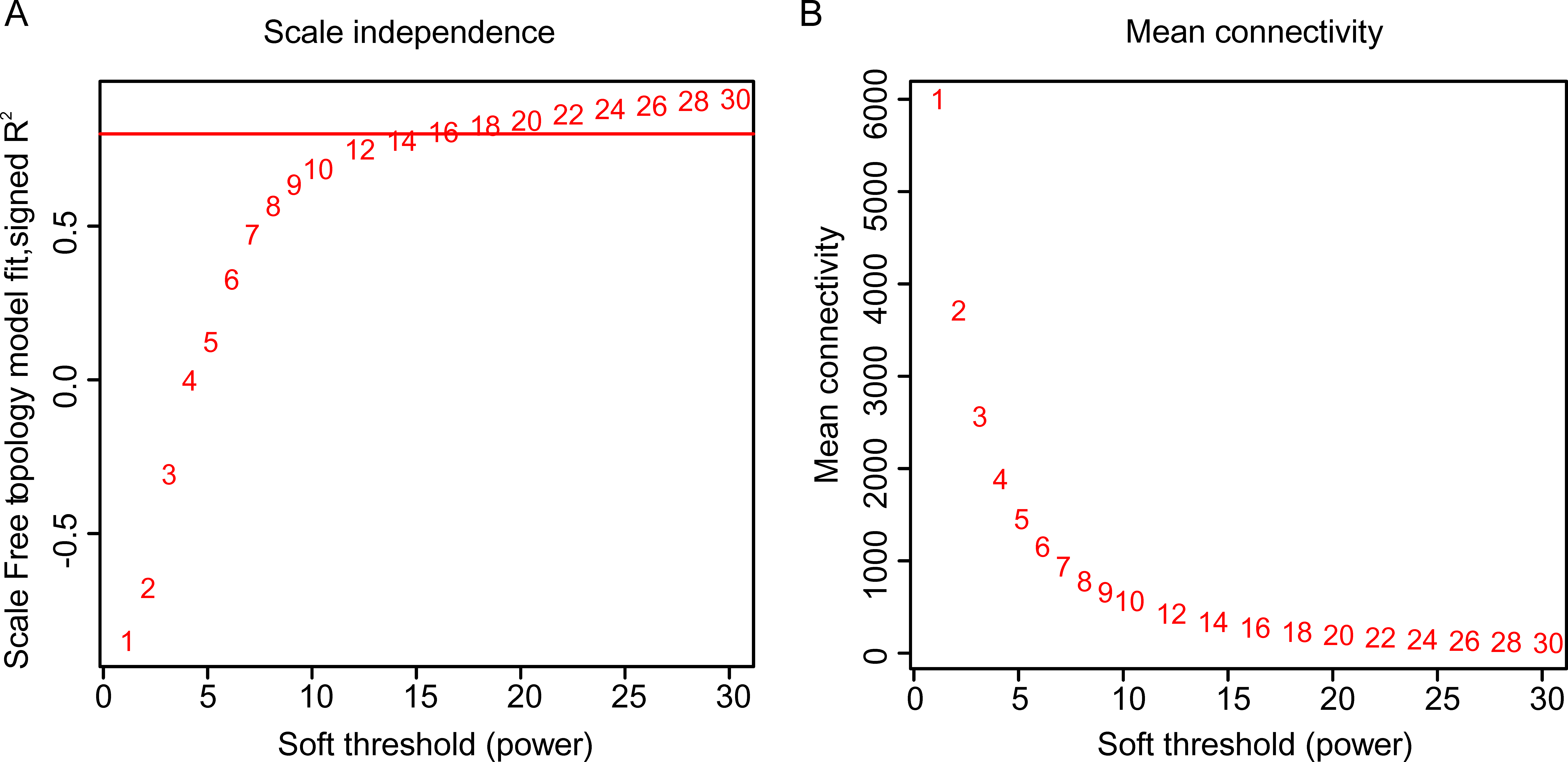

Supplement: Supplemental Information 11 — (A) Analysis of scale-free fit index of each β value from 1 to 30. (B) β =16 was chosen for subsequent analyses as it has the biggest mean connectivity. [file peerj-09-10977-s011.png]

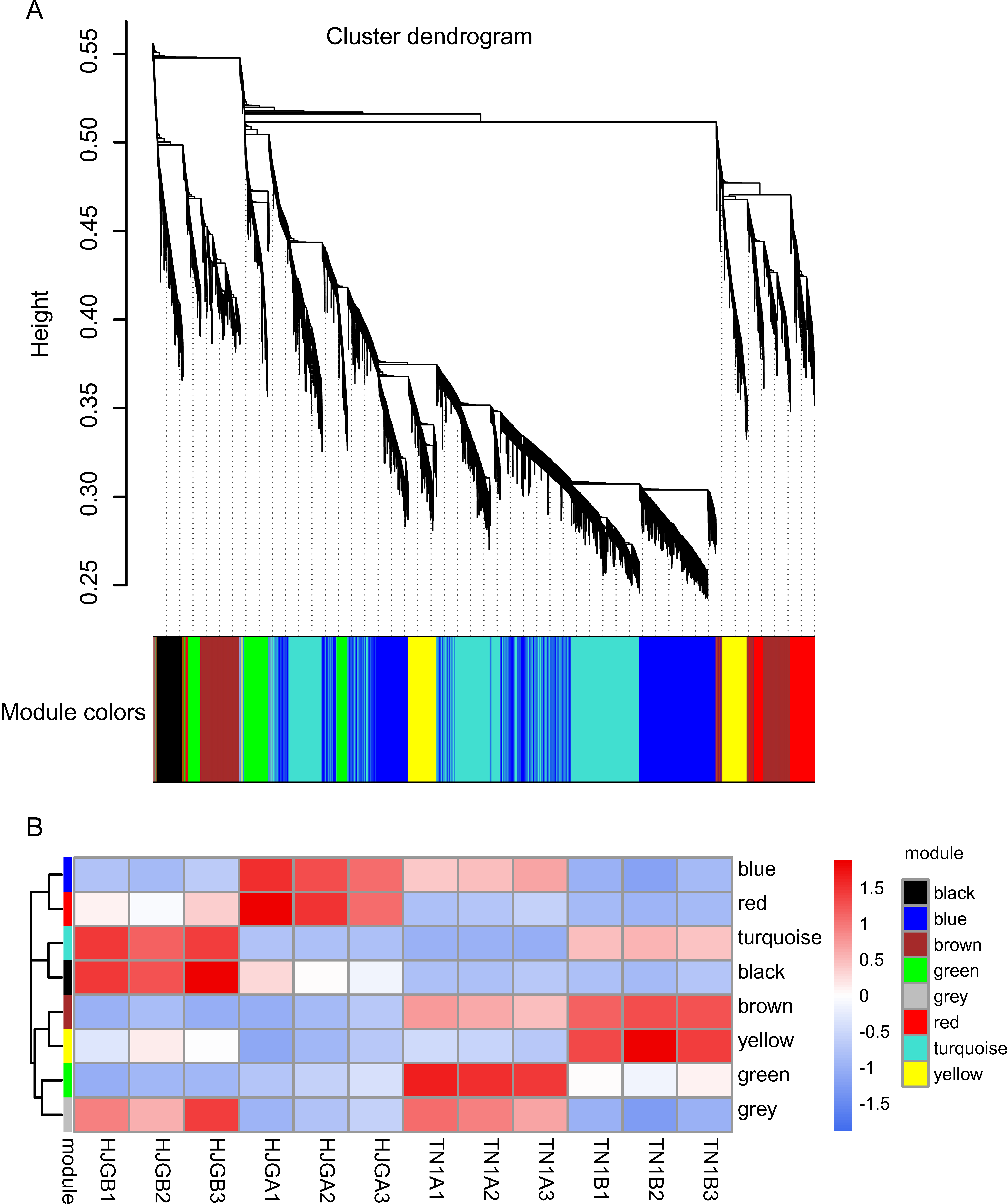

Supplement: Supplemental Information 12 — (A) Gene cluster dendrograms and module detecting. The top is the sample hierarchical clustering tree; the bottom is the result of merging similar modules according to the dynamic tree cut. (B) Correlation between gene modules and traits. [file peerj-09-10977-s012.png]
